# Isothiourea-catalysed enantioselective Michael addition of N-heterocyclic pronucleophiles to α,β-unsaturated aryl esters†

**DOI:** 10.1039/c9sc04303a

**Published:** 2019-10-23

**Authors:** Chang Shu, Honglei Liu, Alexandra M. Z. Slawin, Cameron Carpenter-Warren, Andrew D. Smith

**Affiliations:** EaStCHEM, School of Chemistry, University of St Andrews North Haugh, St Andrews Fife KY16 9ST UK ads10@st-andrews.ac.uk

## Abstract

The isothiourea-catalysed enantioselective Michael addition of 3-aryloxindole and 4-substituted-dihydropyrazol-3-one pronucleophiles to α,β-unsaturated *p*-nitrophenyl esters is reported. This process generates products containing two contiguous stereocentres, one quaternary, in good yields and excellent enantioselectivities (>30 examples, up to > 95 : 5 dr and 99 : 1 er). This protocol harnesses the multifunctional ability of *p*-nitrophenoxide to promote effective catalysis. In contrast to previous methodologies using tertiary amine Lewis bases, in which the pronucleophile was used as the solvent, this work allows bespoke pronucleophiles to be used in stoichiometric quantities.

## Abstract 2

The isothiourea-catalysed enantioselective Michael addition of 3-aryloxindole and 4-substituted-dihydropyrazol-3-one pronucleophiles to α,β-unsaturated *p*-nitrophenyl esters is reported.

## Graphical abstract

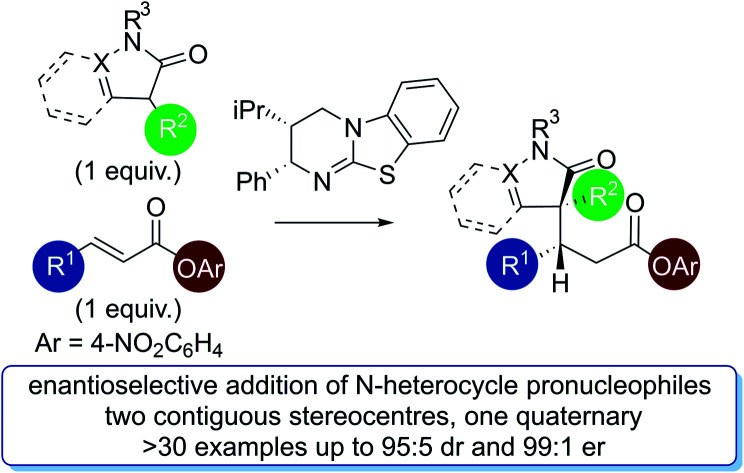

## Introduction

**Scheme 1:**
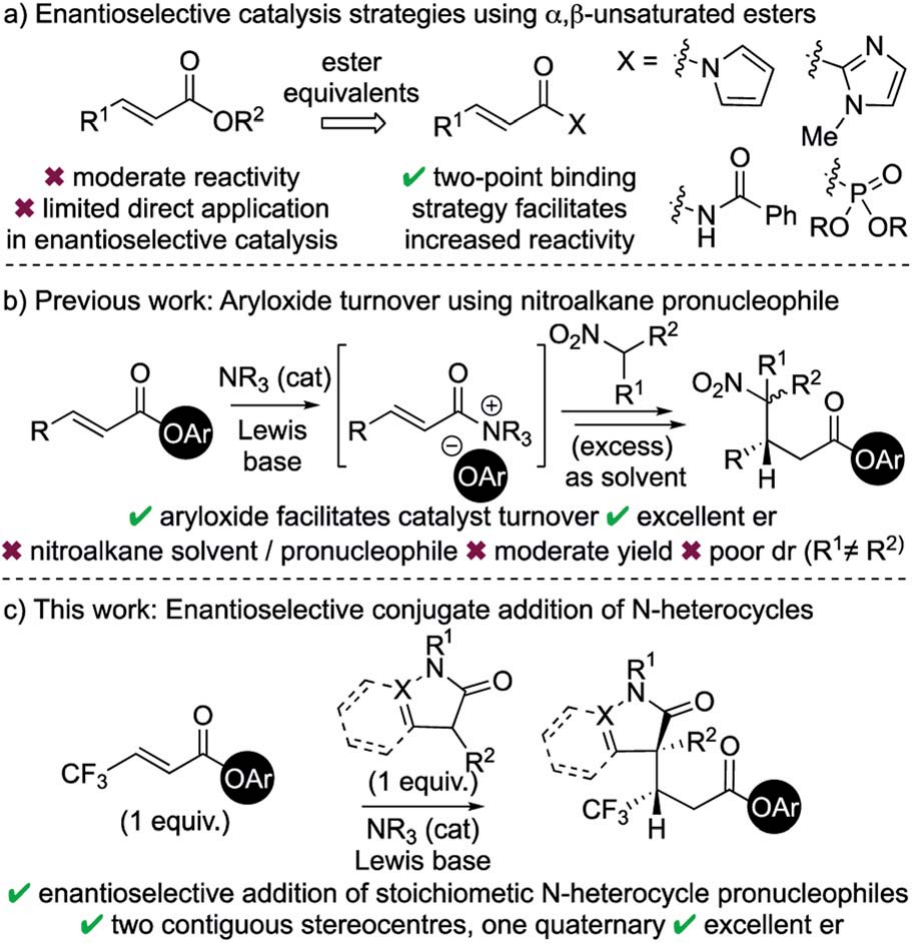
Enantioselective Michael addition to α,β-unsaturated esters.

Catalytic enantioselective Michael addition of enolate equivalents to α,β-unsaturated carbonyl compounds represents an efficient methodology for stereoselective C–C bond formation.^1^ Within this field, considerable advances in catalytic enantioselective Michael additions to enals and enones have been reported.^2^ Typical strategies involve activation of the Michael acceptor through iminium ion formation,^3^ H-bonding organocatalysis,^4^ or Lewis acid catalysis.^5^ In comparison to enals and enones, the intrinsic recalcitrance of α,β-unsaturated esters^6^ represents a significant challenge in enantioselective catalysis (Scheme 1a).^7^ Established metal-based catalytic systems allow, for example, conjugate additions of aryl boronic acids and Grignard reagents to α,β-unsaturated esters.^8^ Broader reactivity has been targeted through developing the use of ester surrogates such as *N*-acylpyrroles,^9^ 2-acyl imidazoles,^10^ activated imides^11^ and β,γ-unsaturated acyl phosphonates.^12^ Catalytic strategies using these motifs typically rely upon two-point binding between the enoyl substrate and either a Lewis acidic metal catalyst^13^ or a H-bond donor organocatalyst.^14^ Despite these advances, only limited organocatalytic strategies have been developed that allow activation of α,β-unsaturated ester substrates, with the current state-of-the-art strategies having been showcased by List (silylium catalysis)^15^ and Dixon (BIMP catalysis).^16^

Chiral α,β-unsaturated acyl ammonium intermediates are readily prepared *in situ* from α,β-unsaturated acyl halides and anhydrides using tertiary amine Lewis base catalysts.^17^ They have been utilised as convenient and powerful synthons in a number of organocascade reactions,^18^ yet the Lewis base catalysed activation of α,β-unsaturated esters are rare. One major limitation within this field is the requirement of the reactive partner to contain two distinct nucleophilic functionalities to facilitate conjugate addition and subsequent catalyst turnover.^18*a*^ In previous work we exploited aryloxide- facilitated catalyst turnover to show the compatibility of monofunctional nucleophilic reaction partners in this reaction manifold.^19^ This allowed the conjugate addition of nitronate anions to *in situ* generated α,β-unsaturated acyl ammonium intermediates. However, this transformation was inherently limited to the use of excess nitroalkane as both solvent and pronucleophile and was not applicable for the formation of quaternary stereogenic centres.

In the area of medicinal chemistry, the incorporation of N-heterocycles and fluorinated substituents into substrates are common strategies to improve physiochemical properties.^20^ In this context, the application of this aryloxide turnover strategy to incorporate these valuable motifs was targeted.^21^ The major challenges were to identify suitable pronucleophiles containing N-heterocycles that could (i) be used as stoichiometric reagents rather than solvent; (ii) lead to the formation of a quaternary stereocentre; and (iii) be compatible with a range of acyl ammonium precursors. In this manuscript, these challenges are met through the enantioselective conjugate addition of enolates derived from dihydropyrazol-3-ones and 3-substituted oxindoles to a range of α,β-unsaturated aryl esters, particularly those bearing a β-trifluoromethyl substituent.

## Results and discussion

**Fig. 1:**
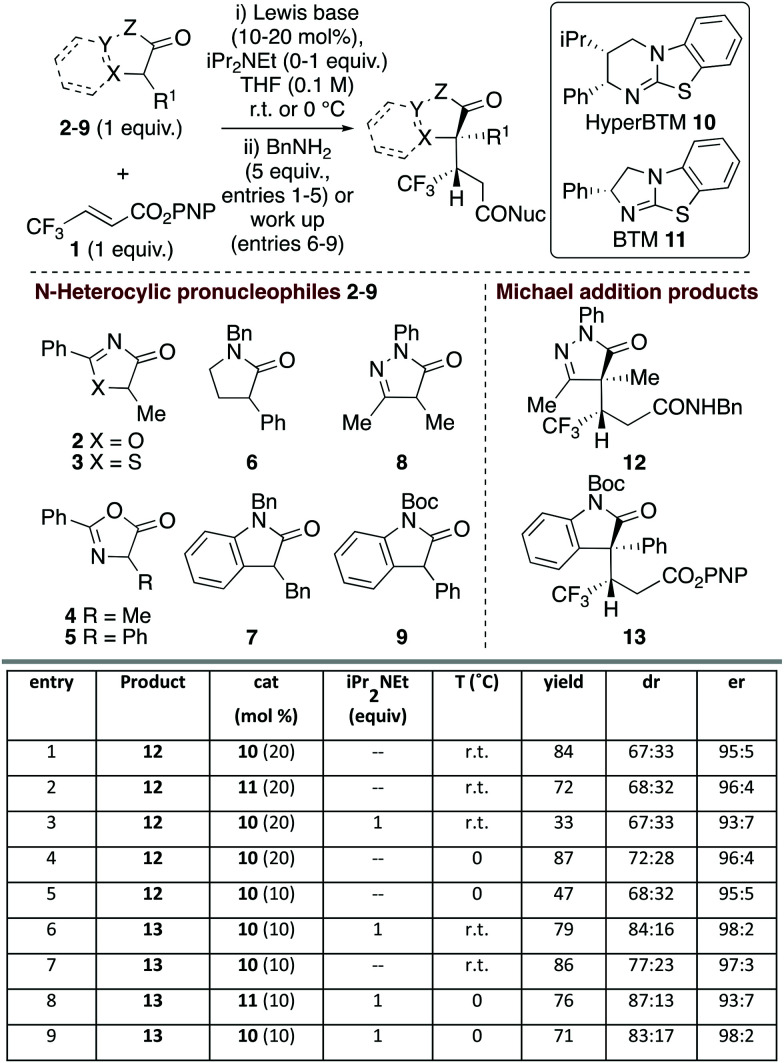
Screening of pronucleophiles. Isolated overall yields given; dr determined by ^1^H NMR spectroscopic analysis of crude mixture; er determined by chiral HPLC analysis of purified products and refers to er of major diastereoisomer.

Initial studies probed the addition of a range of model N-heterocycle containing pronucleophiles **2–9** (1 equiv.) to β-trifluoromethyl α,β-unsaturated *p*-nitrophenyl (PNP) ester **1** to assess the feasibility of this process (Fig. 1). Screening showed that oxazolones and thiazolones **2–5**, pyrrolidinone **6** and 3-benzyl oxindole **7** gave < 10% conversion to product using HyperBTM as the Lewis base catalyst (see ESI† for full details).

However dihydropyrazol-3-one **8** and 3-phenyl oxindole **9** pronucleophiles^22^ gave good reactivity, consistent with their expected lower p*K*_a_ and associated ease of enolate formation. Using dihydropyrazol-3-one **8**, followed by addition of benzylamine, gave isolable amide **12**,^23^ with HyperBTM **10** giving marginally improved yield over BTM **11** (entries 1 and 2). Consistent with the oxidative susceptibility of dihydropyrazol-3-ones in the presence of base,^24^ only low product yield was observed with iPr_2_NEt (entry 3). Variation of solvent and temperature showed that THF at 0 °C was optimal, giving the desired amide in 72 : 28 dr.^25^ Purification of the separable diastereoisomers provided 87% overall yield, with each diastereoisomer obtained with high enantioselectivity (96 : 4 er) (entry 4). Attempted reduction of the catalyst loading to 10 mol% gave **12** with high enantioselectivity but markedly reduced yield (entry 5). Moving to the oxindole series, *N*-Boc-3-phenyl-oxindole **9** gave isolable ester **13**, with iPr_2_NEt required for optimal diastereoselectivity (entries 6 and 7). Variation of Lewis base catalysts **10** and **11** at 0 °C showed that HyperBTM **10** was optimal, giving **13** in 71% yield, 83 : 17 dr and 98 : 2 er (entry 9).

Further studies focused on structural variations of the pronucleophile. In the dihydropyrazol-3-one series, variation of the N(2)-, C(4)- or C(5)-substituents was investigated (Table 1). Variation of the N(2)-substituent showed that N-Ph gave higher product yield than N-Me or N-Bn substitution. Although moderate diastereoselectivities were observed, in each case purification allowed separation of the diastereoisomers, which were all obtained with excellent enantioselectivity (products **12**, **25–26**). C(4)-Ethyl and allyl variants, as well as a range of substituted C(4)-benzyl^26^ derivatives, were also tolerated, giving good product yields with high enantioselectivity (products **27–34**). Using a C(4)-phenyl substituted pronucleophile with HyperBTM **10** gave **35** in moderate yield, while the use of (*R*)-BTM **11** gave improved yield and stereocontrol.

**Table 1:** Scope and limitations: dihydropyrazol-5-one pronucleophiles^a^

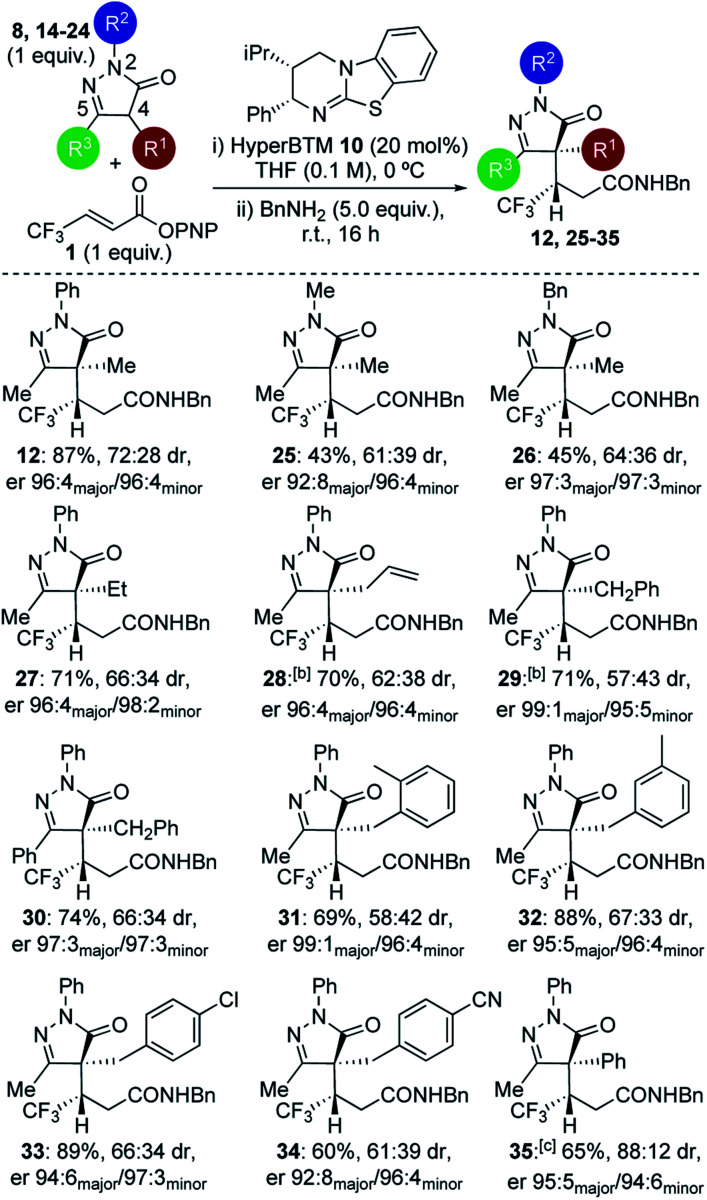

a Isolated overall yields given; dr determined by ^1^H NMR spectroscopic analysis of crude mixture; er determined by chiral HPLC analysis of purified products.

b Reaction at room temperature.

c 20 mol% of (*R*)-BTM use.

**Fig. 2:**
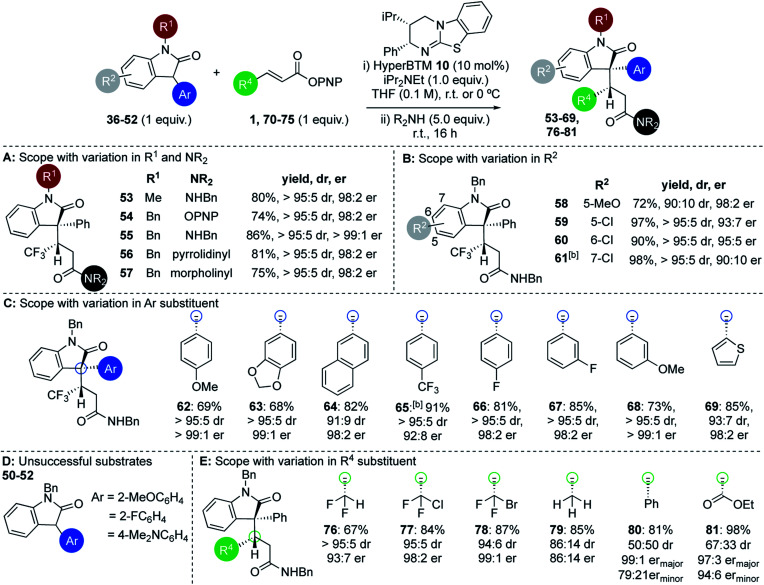
(A–E) Scope and limitations: 3-aryloxindole pronucleophiles and α,β-unsaturated aryl esters.^a^ Isolated overall yields given; dr determined by ^1^H NMR spectroscopic analysis of crude mixture; er determined by chiral HPLC analysis.^b^ Reaction carried out at −40 °C.

Within the 3-aryloxindole class, *N*-methyl substitution led to improved stereoselectivity over the N-Boc variant, giving amide **53** in 80% yield, >95 : 5 dr and 98 : 2 er (Fig. 2A). Using *N*-benzyl 3-phenyl oxindole led to similarly excellent stereoselectivity, giving ester **54** directly in 74% yield, >95 : 5 dr and 98 : 2 er,^26^ or the corresponding amides **55–57** (following addition of the appropriate amine) in excellent yield, >95 : 5 dr and >98 : 2 er in each case. Further investigation probed the scope and limitations of this transformation through subjecting a variety of substituted 3-aryloxindoles to this protocol. Notably, studies showed that 5-, 6-, and 7-substituents within the oxindole were readily tolerated (Fig. 2B). For example, 5-MeO substituted 3-phenyl oxindole gave **58** in 72% yield, 90 : 10 dr and 98 : 2 er, while 5-Cl-, 6-Cl and 7-Cl derivatives gave **59–61** respectively in excellent yield, >95 : 5 dr and up to 95 : 5 er. This protocol was extended to a range of 3-aryloxindole pronucleophiles bearing either extended π-systems, fluorinated substituents, electron-donating or electron-withdrawing substituents, as well as the heteroaryl 3-(2-thienyl) motif (Fig. 2C). Products **62–69** were isolated in good to excellent yields with 91 : 9 to >95 : 5 dr and 92 : 8 to >99 : 1 er. Either 2-substitution of the oxindole 3-aryl group (2-MeOC_6_H_4_, 2-FC_6_H_4_), or the incorporation of a strongly electron-donating 4-Me_2_NC_6_H_4_ substituent, led to no reactivity, presumably due to either additional steric encumberance or increased p*K*_a_ of the substrate disfavouring enolate formation (Fig. 2D). Extension of this procedure to a range of β-substituted α,β-unsaturated esters was also probed, using *N*-benzyl-3-phenyloxindole **37** as a representative pronucleophile. Electron-withdrawing groups at the β-position were readily tolerated, with C(3)-difluoromethyl-, C(3)-chlorodifluoromethyl-, and C(3)-bromodifluoromethyl-substituted esters giving the corresponding derivatives **76–78** in good to excellent yields and high diastereo- and enantiocontrol (Fig. 2E). Significantly, both crotonic and cinnamic PNP esters also proved compatible with this methodology. In our previous work crotonic esters proved low yielding (∼20%) while cinnamic derivatives gave no reaction,^19^ highlighting the significant potential of this approach. For example, addition of **37** to the crotonic PNP ester gave **79** in 85% yield with promising stereocontrol (86 : 14 dr and 86 : 14 er). Addition of **37** to the cinnamate PNP ester derivative gave **80** with poor diastereocontrol but high enantioselectivity in excellent yield.^27^ The incorporation of a β-ester substituent gave product **81** in moderate dr but excellent overall yield and enantioenrichment, indicating that the hybridisation of the β-substituent may be significant in determining diastereocontrol.

**Scheme 2:**
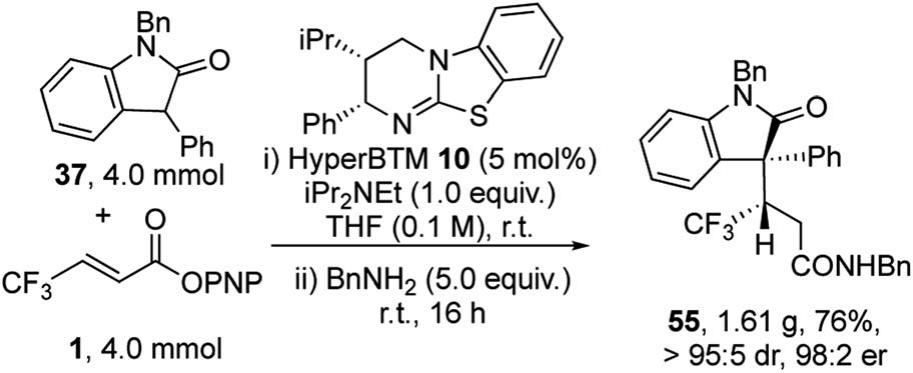
Gram-scale experiment.

To demonstrate the utility of this process a gram scale reaction was carried out (Scheme 2). At this practical scale the catalyst loading of HyperBTM **10** could be readily reduced to 5 mol%, giving product **55** in 76% yield, >95 : 5 dr and 98 : 2 er.

**Scheme 3:**
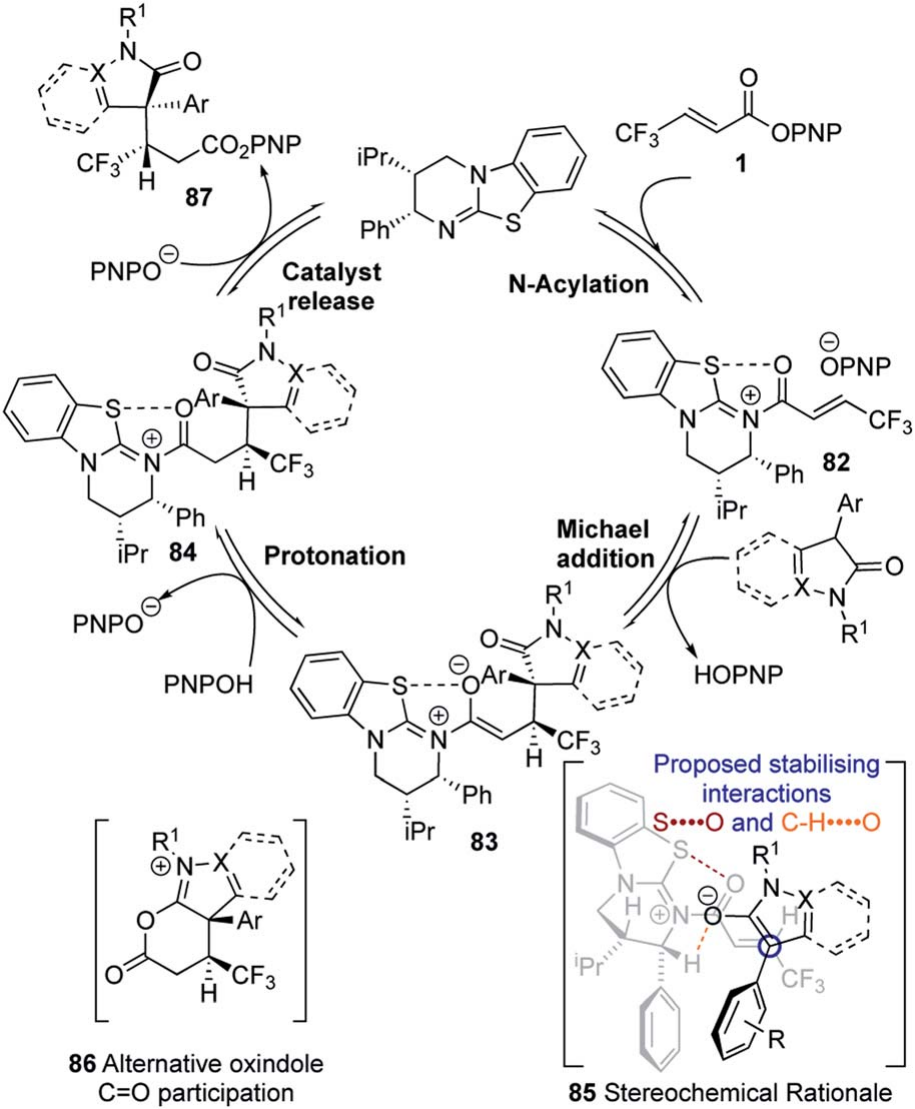
Proposed mechanism for the reaction.

Following our previous mechanistic investigations,^19^ a proposed catalytic cycle is outlined in Scheme 3. Catalysis is initiated through rapid and reversible catalyst acylation by the α,β-unsaturated PNP ester **1** to give α,β-unsaturated acyl isothiouronium ion pair **82**. Deprotonation of the pronucleophile by the released *p*-nitrophenoxide,^28^ followed by Michael addition of the resultant enolate to the α,β-unsaturated acyl isothiouronium **82**, in the assumed stereo-determining step, will generate isothiouronium enolate **83**. Subsequent protonation, presumably by the generated *p*-nitrophenol, gives acyl isothiouronium ion pair **84**. Finally, catalyst turnover either directly by *p*-nitrophenoxide, or by intramolecular participation from the oxindole C=O to give **86**, followed by addition of *p*-nitrophenoxide, gives the Michael addition product **87** and regenerates the isothiourea. The stereochemical outcome of the reaction can be rationalised by the α,β-unsaturated acyl isothiouronium **82** adopting an s-*cis* conformation, with a 1,5 S⋯O interaction between the acyl O and catalyst S providing a conformational lock.^17*p*,29^ Enantioselective conjugate addition of the N-heterocycle-derived enolate to the *Si*-face of the α,β-unsaturated acyl isothiouronium **82** takes place *anti*- to the stereodirecting pseudo-axial phenyl substituent of the acylated HyperBTM isothiourea catalyst.

The observed diastereoselectivity is consistent with the reaction proceeding through pre-transition state assembly **85**, in which non-bonding interactions around the prostereogenic centres are minimised while allowing for a potentially-stabilising C–H⋯O interaction^30^ between the enolate oxygen and acidic α-ammonium C–H of the acylated catalyst.

## Conclusions

In summary, we have developed an isothiourea-catalysed enantioselective protocol for the Michael addition of 3-aryloxindole and 4-substituted-dihydropyrazol-3-one pronucleophiles to a range of α,β-unsaturated *p*-nitrophenyl esters. This protocol allows the use of pronucleophiles as stoichiometric reagents rather than solvent, affording products containing N-heterocycles and fluorinated substituents bearing contiguous quaternary and tertiary stereocenters in moderate to high yield and with generally excellent diastereo- and enantioselectivity (>30 examples, up to >95 : 5 dr and 98 : 2 er). A broad range of substitution patterns within the heterocyclic pronucleophiles is tolerated, with 3-aryloxindoles leading to optimal diastereo- and enantiocontrol. Variation of the β-substituent within the α,β-unsaturated ester showed that electron-withdrawing β-substituents provided optimal stereocontrol. Notably, in contrast to our previous work, both crotonic and cinnamic esters gave high product yields, further demonstrating the generality of this process. This protocol enhances the utility of α,β-unsaturated acyl ammonium catalysis and uses the multifunctional ability of the aryloxide to act as a leaving group, a proton shuttle (through acting as a Brønsted base, then Brønsted acid as *p*-nitrophenol) and as a Lewis base to promote catalyst turnover.^31^

## Conflicts of interest

There are no conflicts to declare.

## Supplementary Material

SC-011-C9SC04303A-s001

SC-011-C9SC04303A-s002
