# Supplementary material for: Isothiourea-catalysed enantioselective Michael addition of N-heterocyclic pronucleophiles to α,β-unsaturated aryl esters
Source: Chem Sci. 2019 Oct 23;11(1):241–7. doi: 10.1039/c9sc04303a (PMC8133005; doi:10.1039/c9sc04303a)
Supplement: SC-011-C9SC04303A-s001 [file SC-011-C9SC04303A-s001.pdf]

Electronic Supplementary Material (ESI) for Chemical Science.
This journal is © The Royal Society of Chemistry 2019

Isothiourea-Catalysed Enantioselective Michael Addition of *N*-heterocyclic Pronucleophiles to α,β-Unsaturated Aryl Esters

Chang Shu, Honglei Liu, Alexandra. M. Z. Slawin, Cameron Carpenter-Warren and Andrew D. Smith*

EaStCHEM, School of Chemistry, University of St Andrews, North Haugh, St Andrews, Fife, KY16 9ST, UK

Supporting Information

Table of Contents

1. General Information S2
2. General Procedures S4
3. Preparation of Starting Materials S8
4. Michael Addition Products S19
5. Determination of Product Configuration by X-ray Crystallography S49
6. References S51

Appendix I: Reaction of Unsuccessful Pronucleophiles S52
Appendix II: Supplementary Optimisation Studies S53
Appendix III: NMR Spectra S55
Appendix IV: HPLC Spectra S131

1 General Information

Reactions involving moisture sensitive reagents were carried out in flame-dried glassware under an argon or nitrogen atmosphere using standard vacuum line techniques and using anhydrous solvents. Anhydrous solvents (THF, CH_2Cl_2 and toluene) were obtained from an anhydrous solvent system (purified using an alumina column, Mbraun SPS-800). All other reactions were performed in standard glassware with no precautions to exclude air or moisture. Solvents and commercial reagents were used as supplied without further purification unless otherwise stated.

Room temperature (r.t.) refers to 20-25 °C. Temperatures of 0 °C and −78 °C were obtained using ice/water and CO_2(s)/acetone baths, respectively. Temperatures of −40 °C for overnight reactions were obtained using an immersion cooler (HAAKE EK 90). Reflux conditions were obtained using a DrySyn, oil bath or sand bath equipped with a contact thermometer.

'*In vacuo*' refers to the use of either a Büchi Rotavapor R-200 with a Büchi V-491 heating bath and Büchi V-800 vacuum controller, a Büchi Rotavapor R-210 with a Büchi V-491 heating bath and Büchi V-850 vacuum controller, a Heidolph Laborota 4001 with vacuum controller, an IKA RV10 rotary evaporator with a IKA HB10 heating bath and ILMVAC vacuum controller, or an IKA RV10 rotary evaporator with a IKA HB10 heating bath and Vacuubrand CVC3000 vacuum controller. Rotary evaporator condensers are fitted to Julabo FL601 Recirculating Coolers filled with ethylene glycol and set to –5 °C.

Analytical thin layer chromatography was performed on pre-coated aluminium plates (Kieselgel 60 F_{254} silica). TLC visualisation was carried out with ultraviolet light (254 nm), followed by staining with a 1% aqueous $KMnO_4$ solution. Manual column chromatography was performed in glass columns fitted with porosity 3 sintered discs over Kieselgel 60 silica using the solvent system stated. Automated chromatography was performed on a Biotage Isolera Four running Biotage OS578 with a UV/Vis detector using the method stated and cartridges filled with Kieselgel 60 silica.

Melting points were recorded on an Electrothermal 9100 melting point apparatus and are uncorrected.

Optical rotations were measured on a PerkinElmer Precisly/Model-341 polarimeter operating at the sodium D line with a 100 mm path cell at 20 °C.

HPLC analyses were obtained using either a Shimadzu HPLC consisting of a DGU-20A5 degassing unit, LC-20AT liquid chromatography pump, SIL-20AHT autosampler, CMB-20A communications bus module, SPD-M20A diode array detector and a CTO-20A column oven; or a Shimadzu HPLC consisting of a DGU-20A5R degassing unit, LC-20AD liquid chromatography pump, SIL-20AHT autosampler, SPD-20A UV/Vis detector and a CTO-20A column oven. Separation was achieved using DAICEL CHIRALCEL OD-H and OJ-H columns or DAICEL CHIRALPAK AD-H, AS-H, IA, IB, IC and ID columns. All HPLC traces of enantiomerically-enriched compounds were compared with authentic racemic spectra.

^{1}H, ^{13}C, ^{19}F nuclear magnetic resonance (NMR) spectra were acquired on either a Bruker Avance 300 (^{1}H 300 MHz), Bruker Avance II 400 (^{1}H 400 MHz; ^{13}C 101 MHz; ^{19}F 376 MHz) or a Bruker Avance II 500 (^{1}H 500 MHz; ^{13}C 126 MHz) spectrometer at ambient temperature in the deuterated solvent stated. All chemical shifts are quoted in parts per million (ppm) and referenced to the residual solvent peak. All coupling constants, J, are quoted in Hz. Multiplicities are indicated by: s (singlet), d (doublet), t (triplet), q (quartet), sept (septet), dd (doublet of doublets), dt (doublet of triplets), dq (doublet of quartets), td

(triplet of doublets), tt (triplet of triplets), ddd (doublet of doublet of doublets), ddt (doublet of doublet of triplets) dddd (doublet of doublet of doublet of doublets), dddt (doublet of doublet of doublet of triplets), and m (multiplet). The abbreviation Ar is used to denote aromatic, Ph to denote phenyl, Bn to denote benzyl, br to denote broad and app to denote apparent.

Infrared spectra were recorded on a Shimadzu IRAffinity-1 Fourier transform IR spectrophotometer fitted with a Specac Quest ATR accessory (diamond puck). Spectra were recorded of either thin films or solids, with characteristic absorption wave numbers (max) reported in cm^{-1}.

Mass spectrometry (m/z) data were acquired by electrospray ionization (ESI), electron impact (EI), atmospheric pressure chemical ionization (APCI) or nanospray ionization (NSI) either at the University of St Andrews or the EPSRC National Mass Spectrometry Facility, Swansea.

2 General Procedures

General Procedure 1: *Synthesis of α,β-unsaturated aryl esters*

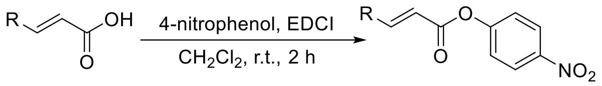

To a solution of α, β-unsaturated acid (1.0 equiv.) and EDCI (1.2 equiv.) in anhydrous dichloromethane (0.67 M) was added 4-nitrophenol (1.1 equiv.) at room temperature. The resulting solution was stirred at r.t. for 2 h till the total consumption of starting material. The solvent was removed *in vacuo*, and the yellow residue was purified by by flash silica column chromatography using a mixture of petroleum ether and ethyl acetate as eluent.

General Procedure 2: *Synthesis of 2-phenyloxazol-4(5H)-one* [1]

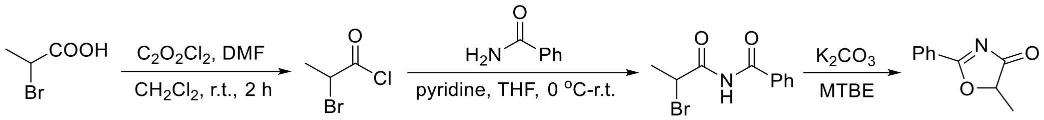

A solution of oxalyl chloride (4.1 mL, 48 mmol) in dry dichloromethane (24 mL) was added slowly to a stirred solution of the corresponding 2-bromopropanoic acid (6.12 g, 40 mmol) in dichloromethane (20 mL) at 0 °C, then 2 drops of DMF were added. Gas evolution was observed, and the system was allowed to stir at room temperature for 2 additional hours. Volatiles were removed under reduced pressure and the resulting crude 2-bromopropanoyl chloride was subject to next step.

To a solution of benzamide (4.8 g, 40 mmol) and pyridine (3.24 mL, 40 mmol) in THF (50 mL) was added the corresponding 2-bromopropanoyl chloride (6.86 g, 40 mmol) dropwise over 5 min at 0 °C. The resulting suspension was stirred overnight at room temperature and diluted with EtOAc. The mixture was acidified to ca. pH 2 with 1N HCl aq. and the phases were separated. The aqueous phase was extracted with EtOAc (3 × 80 mL) and the combined organic layers were washed with water (3 × 80 mL) and brine (80 mL), dried over $MgSO_4$, and filtered. Volatiles were removed under reduced pressure. The *N*-(2-bromopropanoyl) benzamide product was purified by flash chromatography on silica gel (eluting with hexane/ ethyl acetate 90/10).

A suspension of K_2CO_3 (4.0 g, 40 mmol) in methyl *tert*-butylether (MTBE) (40 mL) was refluxed for 2 h to remove water using a Dean-Stark trap. The suspension was cooled to room temperature and the corresponding imide (5.12 g, 20 mmol) was added in one portion. The resulting mixture was refluxed overnight and cooled to room temperature. Inorganic salts were filtered through a celite pad with suction and the filter cake was washed with EtOAc. The combined organic layers were concentrated *in vacuo* and purified by flash chromatography on silica gel (eluting with hexane/ ethyl acetate 80/20).

General Procedure 3: *Synthesis of 5-methyl-2-phenylthiazol-4(5H)-one*[2]

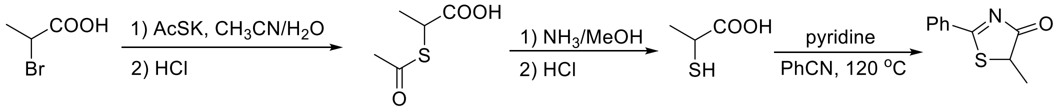

To a solution of the DL-2-bromopropionic acid (2.55 g, 16.7 mmol) in 40 mL of CH_3CN was added a solution of potassium thioacetate (3.8 g, 33.3 mmol) in H_2O (10 mL), and the mixture was stirred for 3 h. The organic solvent was evaporated and the mixture was diluted with H_2O and washed with CH_2Cl_2.

The aqueous phase was acidified with concentrated hydrochloric acid, extracted with CH_2Cl_2 (3 × 17 mL), dried over $MgSO_4$ and concentrated to afford the α-(acetylthio) carboxylic acid as an off white solid. Subsequently, the solid was dissolved in MeOH (10 mL) at 0 °C and ammnia (7N in MeOH, 18 mL) was added to the solution. The mixture was allowed to warm up to room temperature and stirred for 1 h. The organic solvent was then completely evaporated under reduced pressure and the residue was dissolved in a saturated aqueous solution of $NaHCO_3$. The solution was washed with EtOAc, the aqueous phase was acidified with concentrated hydrochloric acid and extracted with EtOAc. The organic layers were combined, dried over $MgSO_4$ and the solvent was evaporated under reduced pressure to afford the α-mercaptopropanoic acid without further purification.
Under the N_2 atmosphere, to a mixture of PhCN (612.6 μL, 6 mmol) and pyridine (97 μL, 1.2 mmol) was added the α-mercaptocarboxylic acid (636.8 mg, 6 mmol) at room temperature, and the mixture was stirred overnight at 120 °C. After cooling to room temperature, a yellow solid could be observed, and was collected by filtration and washed with cold methanol to give the pure product.

General Procedure 4: *Synthesis of oxazol-5-ones*[3]

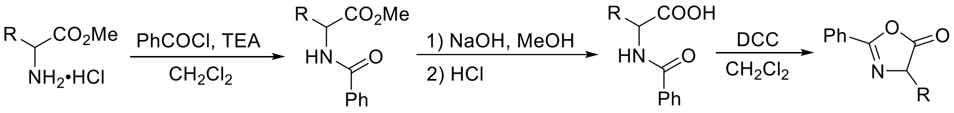

CH_2Cl_2 (90 mL) and TEA (7.6 mL, 54.7 mmol) were added to a flask containing Alanine methyl ester hydrochloride (23.75 mmol). The resulting slurry was cooled to 0 °C, and benzoyl chloride (2.68 mL, 23.3 mmol) in CH_2Cl_2 (7 mL) was added by cannula over 15 min. After 75 min, the ice bath was removed, and the mixture was stirred at room temperature for 4 h. The mixture was then washed with 1 N HCl (2 × 75 mL), saturated $NaHCO_3$ (2 × 75 mL) and saturated NaCl (75 mL). The CH_2Cl_2 layer was dried with $MgSO_4$, and the solvent was removed by rotary evaporation, providing amide as a white solid. The solid was dissolved in MeOH (40 mL), and 2 N aqueous NaOH (12.4 mL) was added. The resulting mixture was stirred for 20 min, and then the methanol was removed by rotary evaporation. Water was added until the aqueous solution was homogeneous, and then the aqueous solution was washed with CH_2Cl_2 (2 × 50 mL). The aqueous layer was made acidic with 1 N HCl, which resulted in the formatin of a white precipitate, which was filtered, washed with several portions of water, and dried with a flow of air through a fiter.
A solution of DCC (2.06 g, 10 mmol) in CH_2Cl_2 (10 mL) was added by cannula to a 0 °C slurry of substrate (10 mmol) in CH_2Cl_2 (60 mL). The resulting slurry was allowed to warm to room temperature overnight. The white precipitate was then removed by filtration, and the CH_2Cl_2 solution was washed with saturated $NaHCO_3$ (2 × 50 mL), followed by saturated NaCl (50 mL). The CH_2Cl_2 layer was dried with $MgSO_4$, and the CH_2Cl_2 was removed by rotary evaporation.

General Procedure 5: *Synthesis of 3-monosubstituted oxindoles*

The 3-monosubstituted oxindoles were synthesized by the reported methods.[4]

General Procedure 6: *Synthesis of dihydropyrazol-5-ones*[5]

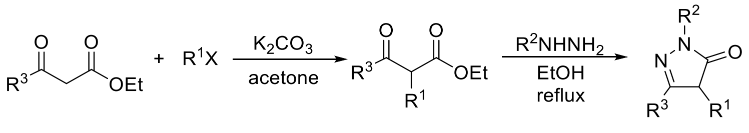

According a published procedure[5], a mixture of acetoacetate (1.0 equiv.) and anhydrous K_2CO_3 (1.3 equiv.) in dry acetone (1.0 M) was stirred under argon atmosphere for five minutes. Then, corresponding halide (1.3 equiv.) was added carefully. The reaction was refluxed overnight. After filtration, the solvent was evaporated. The crude mixture purified by flash chromatography on silica gel with mixture of hexane/ethyl acetate (20:1) affording corresponding pure α-substituted acetoacetate. Then, a mixture of α-substituted acetoacetate (1.0 equiv.) and hydrazine (1.1 equiv.) was refluxed in EtOH (1.0 M) until full conversion. The solvent was removed a residue was crystalized from Et_2O to give the corresponding dihydropyrazol-5-ones.

General Procedure 7: *Asymmetric Michael Addition of oxazol-4(5H)-ones, thiazol-4(5H)-one, oxazol-5-one and dihydropyrazol-5-ones, with 4-nitrophenyl (E)-4,4,4-trifluorobut-2-enoate*

Under N_2, dihydropyrazol-5-ones (0.2 mmol), 4-nitrophenyl (*E*)-4,4,4-trifluorobut-2-enoate (0.2 mmol) and (2*S*,3*R*)-HyperBTM (20 mol%) in THF were allowed to stir for 6–72 h at 0 ℃ or room temperature. Alternatively, benzylamine (1.0 mmol) was added and allowed to stir for a further 16 h at room temperature. Then, 30 mL Et_2O was added, the mixture was washed with 1N NaOH aq (3 × 30 mL) and 1N HCl aq (30 mL). After dried by $MgSO_4$, the mixture was concentrated *in vacuo*, and the residue purified by column chromatography using a mixture of petroleum ether and EtOAc as eluent.

General Procedure 8: *Unasymmetric Michael Addition of dihydropyrazol-5-ones with 4-nitrophenyl (E)-4,4,4-trifluorobut-2-enoate*

Under N_2, dihydropyrazol-5-ones (0.1 mmol), 4-nitrophenyl (*E*)-4,4,4-trifluorobut-2-enoate (0.1 mmol) and *rac*-HyperBTM (20 mol%) in THF were allowed to stir for 6–72 h at 0 ℃ or room temperature. Then, benzylamine (0.5 mmol) was added and allowed to stir for a further 16 h at room temperature. Then, 15 mL Et_2O was added, the mixture was washed with 1N NaOH aqueous solution (3 × 15 mL) and 1N HCl aq (15 mL). After dried by $MgSO_4$, the mixture was concentrated in vacuo, and the residue purified by column chromatography using a mixture of petroleum ether and EtOAc as eluent.

General Procedure 9: *Asymmetric Michael Addition of pyrrolidinone and 3-monosubstituted oxindoles with α,β-unsaturated aryl esters*

The *N*-heterocycle **1** (0.2 mmol), activated 4-nitolphenol ester **2** (0.2 mmol), (2*S*,3*R*)-HyperBTM (10 mol%) and $^{i}PrNEt_2$ (35.0 µL) in anhydrous THF (2.0 mL) was allowed to stir for 24–72 h at rt. For isolation of the PNP ester product, the reaction may be concentrated and purified by column chromatography. Alternatively, an appropriate amine (5.0 equiv.) may be added and allowed to stir for a further 16 h at room temperature. The crude mixture was then concentrated *in vacuo*, and the residue purified by column chromatography using a mixture of petroleum ether and ethyl acetate as eluent.

General Procedure 10: *Unasymmetric Michael Addition of 3-monosubstituted oxindoles with α,β-unsaturated aryl esters*

The *N*-heterocycle **1** (0.2 mmol), activated 4-nitolphenol ester **2** (0.2 mmol), *rac*-HyperBTM (10 mol%) and $^{i}PrNEt_2$ (35.0 µL) in anhydrous THF (2.0 mL) was allowed to stir for 24–72 h at rt. For isolation of the PNP ester product, the reaction may be concentrated and purified by column chromatography. Alternatively, an appropriate amine (5.0 equiv.) may be added and allowed to stir for a further 16 h at room temperature. The crude mixture was then concentrated *in vacuo*, and the residue purified by column chromatography using a mixture of petroleum ether and ethyl acetate as eluent.

General Procedure 11: Grams-scale preparation of **55**

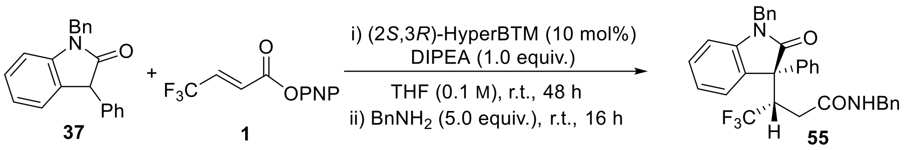

1-benzyl-3-phenylindolin-2-one **36** (1.20 g, 4.0 mmol), 4-nitrophenyl (*E*)-4,4,4-trifluorobut-2-enoate **1** (1.04 g, 4.0 mmol), (2*S*,3*R*)-HyperBTM (61.7 mg, 2.0 mmol) and DIPEA (0.7 mL, 4.0 mmol) in anhydrous THF (20.0 mL) at rt for 48 h. Benzyl amine (2.2 mL, 20.0 mmol) was added and the reaction was stirred at room temperature overnight. The yellow reaction mixture was diluted in diethyl ether (50 mL) and then washed with 1 N NaOH aqueous solution (50 mL×3) and 1 N HCl aqueous solution (100 mL). The organic layers were washed with brine, dried over $MgSO_4$ and concentrated under reduced pressure. The residue was purified by flash column chromatography (6:1 Petrol : EtOAc, R_f 0.13) to get **55** as a white solid (1.61 g, 76% yield).

3 Preparation of Starting Materials

3.1 Data for α,β-unsaturated aryl esters

4-Nitrophenyl (*E*)-4,4,4-trifluorobut-2-enoate (1)

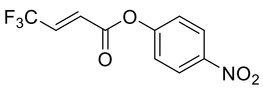

^{1}H NMR (400 MHz, $CDCl_3$) δ_H: 6.72–6.77 (1H, m, F_3CCH=C*H*), 7.00–7.09 (1H, m, F_3CC*H*=CH), 7.37–7.40 (2H, m, ArC(2,6)*H*), 8.31–8.35 (2H, m, ArC(3,5)*H*); **^{19}F NMR (376 MHz, $CDCl_3$) δ_F**: –65.76 (C*F_2*H); **^{13}C NMR (101 MHz, $CDCl_3$) δ_C**: 120.9 (q, *J* 269.0, *C*F_3), 122.2 (2×Ar*C*H), 125.4 (2×Ar*C*H), 127.4 (q, *J* 6.1, F_3CCH=*C*H), 134.1 (q, *J* 35.8, F_3C*C*H=CH), 145.8 (Ar*C*H), 154.6 (Ar*C*H), 161.4 (*C*O_2Ar).

4-Nitrophenyl (*E*)-4,4-difluorobut-2-enoate (70)

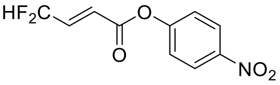

^{1}H NMR (400 MHz, $CDCl_3$) δ_H: 6.22–6.50 (1H, m, CF_2*H*), 6.52–6.57 (1H, m, F_2HCCH=C*H*), 7.03–7.14 (1H, m, F_2HCC*H*=CH), 7.36–7.40 (2H, m, ArC(2,6)*H*), 8.31–8.35 (2H, m, ArC(3,5)*H*); **^{19}F NMR (376 MHz, $CDCl_3$)** δ_F: –116.9 (C*F_2*H); **^{13}C NMR (101 MHz, $CDCl_3$) δ_C**: 111.9 (t, *J* 236.7, *C*F_2H), 122.3 (2×Ar*C*H), 125.4 (2×Ar*C*H), 125.7 (t, *J* 10.3, HF_2CCH=*C*H), 139.5 (t, *J* 24.0, HF_2C*C*H=CH), 145.7 (Ar*C*H), 154.8 (Ar*C*H), 162.1 (*C*O_2Ar).

4-Nitrophenyl (*E*)-4-chloro-4,4-difluorobut-2-enoate (71)

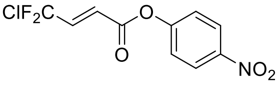

^{1}H NMR (400 MHz, $CDCl_3$) δ_H: 6.63 (1H, dt, *J* 15.6, 1.8, F_2ClCCH=C*H*), 7.18 (1H, dt, *J* 15.6, 9.0, F_2ClCC*H*=CH), 7.37 – 7.41 (2H, m, ArC(2,6)*H*), 8.32 – 8.36 (2H, m, ArC(3,5)*H*); **^{19}F NMR (376 MHz, $CDCl_3$)** δ_F: –54.5 (C*F_2*Cl); **^{13}C NMR (101 MHz, $CDCl_3$)** δ_C: 122.3 (2×Ar*C*H), 123.1 (t, *J* 287.9, F_2Cl*C*), 124.0 (t, *J* 6.5, F_2ClCCH=*C*H), 125.4 (2×Ar*C*H), 139.5 (t, *J* = 29.1 Hz, F_2ClC*C*H=CH), 145.8 (Ar*C*H), 154.6 (Ar*C*H), 161.7 (*C*O_2Ar).

4-Nitrophenyl (*E*)-4-bromo-4,4-difluorobut-2-enoate (72)

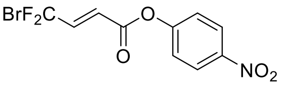

^{1}H NMR (400 MHz, $CDCl_3$) δ_H: 6.54 (1H, dt, *J* 15.6, 1.7, F_2ClCCH=C*H*), 7.24 (1H, dt, *J* 15.6, 9.9, F_2ClCC*H*=CH), 7.37 – 7.41 (2H, m, ArC(2,6)*H*), 8.31 – 8.35 (2H, m, ArC(3,5)*H*); **^{19}F NMR (376 MHz, $CDCl_3$)** δ_F: –50.6 (C*F_2*Cl); **^{13}C NMR (101 MHz, $CDCl_3$)** δ_C: 114.6 (t, *J* 301.8, F_2Cl*C*), 122.3 (2×Ar*C*H), 122.6 (t, *J* 6.8, F_2ClCCH=*C*H), 125.4 (2×Ar*C*H), 141.0 (t, *J* 25.9, F_2ClC*C*H=CH), 145.7 (Ar*C*H), 154.7 (Ar*C*H), 161.7 (*C*O_2Ar).

4-nitrophenyl (*E*)-but-2-enoate (73)

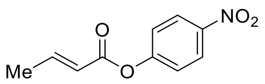

^{1}H NMR (500 MHz, $CDCl_3$) δ_H: 2.01 (3H, dd, *J* 6.9, 1.7, C*H*3), 6.03–6.08 (1H, m, CH=C*H*–CO_2PNP), 7.21–7.28 (1H, m, C*H*=CHCO$_2$PNP), 7.29–7.31 (2H, m, C*H*-Ar$_{PNP}$), 8.26–8.29 (2H, m, C*H*-Ar$_{PNP}$).

4-Nitrophenyl cinnamate (74)

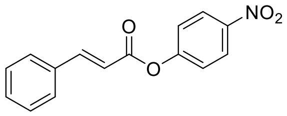

^{1}H NMR (400 MHz, $CDCl_3$) δ_H: 6.63 (1H, d, *J* 16.0, CH=C*H*– CO_2PNP), 7.37–7.39 (2H, m, C*H*-Ar$_{PNP}$), 7.44–7.47 (3H, m, C*H*-Ph), 7.60–7.62 (2H, m, C*H*Ph), 7.92 (1H, d, *J* 16.0, C*H*=CH-CO_2PNP), 8.30–8.32 (2H, m, C*H*-Ar$_{PNP}$).

4-Nitrophenyl (*E*)-3-(*p*-tolyl)acrylate (S1)

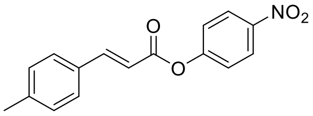

White solid. mp: 109–110°C; ν_{max} (film), 1736 (C=O); **^{1}H NMR (400 MHz, $CDCl_3$)** δ_H: 6.79 (1H, d, *J* 16.0, CH=C*H*–CO_2PNP), 7.40–7.42 (2H, m, C*H*-Ar), 7.78–7.80 (2H, m, C*H*-Ar), 7.79 (1H, d, *J* 16.0, C*H*=CH–CO_2PNP), 8.32–8.36 (4H, m, C*H*-Ar);**^{13}C NMR (101 MHz, $CDCl_3$)** δ_C: 120.5 (CH=*C*H–CO_2PNP), 122.4 (2×*C*H-Ar), 124.4 (2×*C*H-Ar), 125.4 (2×*C*H-Ar), 129.1 (2×*C*H-Ar), 139.7 (*C*H-Ar), 144.9 (*C*H=CH–CO_2PNP), 145.5 (*C*H-Ar), 149.0 (*C*H-Ar), 155.2 (*C*H-Ar), 163.5 (C=O); **HRMS (NSI$^+$)** exact mass calcd. For $C_{16}H_{13}NNaO_4^+$ (M+Na$^+$) requires m/z 306.0737, found m/z 306.0734 (–0.91 ppm).

4-Nitrophenyl (*E*)-3-(4-nitrophenyl)acrylate (S2)

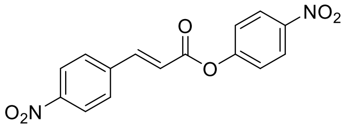

White solid. mp: 189–190°C; ν_{max} (film), 1736 (C=O); **^{1}H NMR (400 MHz, $CDCl_3$)** δ_H: 2.43 (3H, s), 6.60 (1H, d, *J* 16.0, CH=C*H*–CO_2PNP), 7.25–7.28 (2H, m, C*H*-Ar), 7.38–7.40 (2H, m, C*H*-Ar), 7.52–7.54 (2H, m, C*H*-Ar), 7.91 (1H, d, *J* 16.0, C*H*=CH–CO_2PNP), 8.31–8.33 (2H, m, C*H*-Ar);**^{13}C NMR (101 MHz, $CDCl_3$)** δ_C: 21.6 (CH3), 115.0 (CH=*C*H–CO_2PNP), 122.5 (2×*C*H-Ar), 125.2 (2×*C*H-Ar), 128.5 (2×*C*H-Ar), 129.9 (2×*C*H-Ar), 131.1 (*C*H-Ar), 141.9 (*C*H-Ar), 145.2 (*C*H-Ar), 148.1 (*C*H=CH–CO_2PNP), 155.7 (*C*H-Ar), 164.6 (*C*=O); **HRMS (NSI$^+$)** exact mass calcd. For $C_{15}H_{10}N_2NaO_6^+$ (M+Na$^+$) requires m/z 337.0431, found m/z 337.0428 (–0.91 ppm).

Ethyl (4-nitrophenyl) fumarate (75)

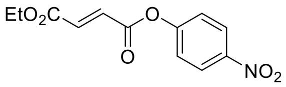

^{1}H NMR (400 MHz, CDCl$_3$) δ_H: 1.36 (3H, t, *J* 7.1, CH$_2$C*H*$_3$), 4.32 (2H, q, *J* 7.1, C*H*$_2$CH$_3$), 7.04 (1H, d, *J* 15.8, CH=C*H*CO$_2$Ar), 7.09 (1H, d, *J* 15.8, C*H*=CHCO$_2$Ar), 7.31–7.43 (2H, m, C*H*-Ar), 8.10–8.13 (2H, m, C*H*-Ar).

3.2 Data for oxazol-4(5*H*)-one

5-Methyl-2-phenyloxazol-4(5*H*)-one (2)

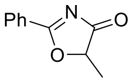

Following General Procedure **2**, the title compound was afforded as a white solid (1.65 g, 42% yield); **^{1}H NMR (400 MHz, CDCl$_3$)** δ_H: 1.49 (3H, d, *J* 7.2, C*H*$_3$), 4.14 (1H, q, *J* 7.2, CH$_3$C*H*), 7.48–7.51 (2H, m, C*H*Ph), 7.61–7.63 (1H, m, C*H*Ph), 8.04–8.06(2H, m, C*H*Ph).

3.3 Data for thiazol-4(5*H*)-one

5-Methyl-2-phenylthiazol-4(5*H*)-one (3)

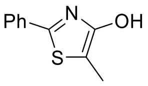

Following General Procedure **3**, the title compound was afforded as a yellow solid (0.42g, 37% yield); **^{1}H NMR (400 MHz, CDCl$_3$)** δ_H: 2.20 (3H, s, C*H*$_3$), 7.42–7.46 (3H, m, C*H*Ph), 7.83–7.85 (2H, m, C*H*Ph), 10.30 (1H, s, O*H*).

3.4 Data for oxazol-5(4*H*)-ones

4-Methyl-2-phenyloxazol-5(4*H*)-one (4)

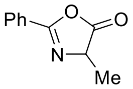

Following General Procedure **4**, the title compound was afforded from *N*-benzoyl-D,L-alanine (1.93 g, 10.0 mmol,) as a white solid (1.40 g, 80%); **^{1}H NMR (400 MHz, CDCl$_3$)** δ_H: 1.62 (3H, d, *J* 7.6, C*H*$_3$), 4.48 (1H, q, *J* 7.6, CH$_3$C*H*), 7.49–7.54 (2H, m, C*H*Ph), 7.59–7.63 (1H, m, C*H*Ph), 8.01–8.04 (2H, m, C*H*Ph).

2,4-Diphenyloxazol-5-(4*H*)-one (5)

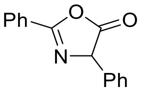

Following General Procedure **4**, the title compound was afforded from *N*-benzoyl-D,L-phenylglycine (2.55 g, 10.0 mmol,) as a yellow solid (1.21 g, 51%); **^{1}H NMR (400 MHz, CDCl$_3$)** δ_H: 5.32 (1H, s, CH), 7.44–7.60 (5H, m, C*H*Ph), 7.63–7.66 (3H, m, C*H*Ph), 7.89–7.91 (2H, m, C*H*Ph).

3.5 Data for 3-monosubstituted oxindoles

1-Benzyl-3-phenylpyrrolidin-2-one (6)

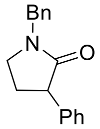

^{1}HNMR (CDCl$_3$, 400 MHz) δ_H: 2.08–2.18 (1H, m, C*H*$_{2(pyrr)}$), 2.48–2.56 (1H, m, C*H*$_{2(pyrr)}$), 3.30–3.41 (2H, m, C*H*$_{2(pyrr)}$), 3.76 (1H, t, *J* 8.8, PhC*H*$_{(pyrr)}$), 4.50 (1H, d, *J* 14.6, NC*H*$_2$Ph), 4.63 (1H, d, *J* 14.6, NC*H*$_2$Ph), 7.27–7.40 (10H, m, Ar-C*H*).

1,3-Dibenzylindolin-2-one (7)

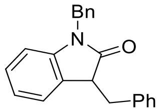

^{1}HNMR (CDCl$_3$, 400 MHz) δ_H: 3.17 (1H, dd, *J* 13.6, 8.1, C*H*$_2$Ph), 3.54 (1H, dd, J 13.6, 8.1, C*H*$_2$Ph), 3.89 (1H, q, *J* 4.3, C(3)*H*), 4.67 (1H, d, *J* 1.6, C*H*$_2$Ph), 5.07 (1H, d, *J* 1.6, C*H*$_2$Ph), 6.58 (1H, d, *J* 7.8, Ar-C*H*), 6.95–7.01 (4H, m, Ar-C*H*), 7.10–7.20 (4H, m, Ar-C*H*), 7.23–7.27 (5H, m, Ar-C*H*).

***tert*-Butyl 2-oxo-3-phenylindoline-1-carboxylate (9)**

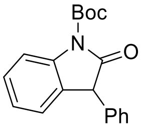

^{1}HNMR (CDCl$_3$, 400 MHz): δ 1.66 (9 H, s, (C*H*$_3$)$_3$), 4.76 (1 H, s, C(3)*H*), 7.18-7.24 (4 H, m, Ar-C*H*), 7.31-7.42 (4 H, m, Ar-C*H*), 7.96 (1 H, d, *J* 8.2, Ar-C*H*).

1-Methyl-3-phenylindolin-2-one (36)

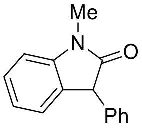

^{1}HNMR (CDCl$_3$, 400 MHz): δ 3.28 (3 H, s, C*H*$_3$), 4.64 (1 H, s, C(3)*H*), 6.93 (1 H, d, *J* 7.8, Ar-C*H*), 7.07-7.11 (1 H, m, Ar-C*H*), 7.19-7.24 (3 H, m, Ar-C*H*), 7.29-7.39 (4 H, m, Ar-C*H*).

1-Benzyl-3-phenylindolin-2-one (37)

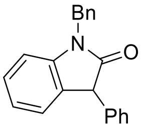

^{1}HNMR (CDCl$_3$, 400 MHz) δ_H: 4.75 (1H, s, C(3)*H*), 4.94 (1H, d, *J* 15.6, C*H*$_2$Ph), 5.04 (1H, d, *J* 15.6, C*H*$_2$Ph), 6.83 (1H, d, *J* 7.8, Ar-C*H*), 7.04–7.08 (1H, m, Ar-C*H*), 7.19–7.41 (12H, m, Ar-C*H*).

1-Benzyl-5-methoxy-3-phenylindolin-2-one (38)

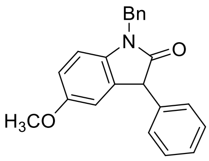

^{1}HNMR ($CDCl_3$, 400 MHz) δ_H: 3.74 (3H, s, OCH_3), 4.72 (1H, s, C(3)*H*), 4.91 (1H, d, *J* 15.6, CH_2Ph), 5.00 (1H, d, *J* 15.6, CH_2Ph), 6.70 (1H, d, *J* 8.5, Ar-C*H*), 6.75–6.78 (1H, m, Ar-C*H*), 6.81 (1H, dd, *J* 2.2, 0.8, Ar-C*H*), 7.25–7.41 (10H, m, Ar-C*H*).

1-Benzyl-5-chloro-3-phenylindolin-2-one (39)

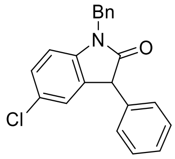

^{1}HNMR ($CDCl_3$, 400 MHz) δ_H: 4.72 (1H, s, C(3)*H*), 4.91 (1H, d, *J* 15.6 , CH_2Ph), 5.01 (1H, d, *J* 15.6, CH_2Ph), 6.72 (1H, d, *J* 8.3, Ar-C*H*), 7.16–7.24 (4H, m, Ar-C*H*), 7.29–7.42 (8H, m, Ar-C*H*).

1-Benzyl-6-chloro-3-phenylindolin-2-one (40)

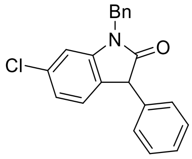

^{1}HNMR ($CDCl_3$, 400 MHz) δ_H: 4.70 (1H, s, C(3)*H*), 4.90 (1H, d, *J* 15.6, CH_2Ph), 5.00 (1H, d, *J* 15.6, CH_2Ph), 6.81 (1H, d, *J* 1.5, Ar-C*H*), 7.03 (1H, dd, *J* 7.9, 1.0, Ar-C*H*), 7.10 (1H, dd, *J* 7.9, 1.0, Ar-C*H*), 7.22–7.24 (2H, m, Ar-C*H*), 7.32–7.41 (8H, m, Ar-C*H*).

1-Benzyl-7-chloro-3-phenylindolin-2-one (41)

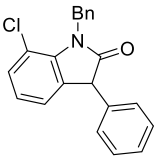

^{1}HNMR ($CDCl_3$, 400 MHz) δ_H: 4.75 (1H, s, C(3)*H*),5.39 (1H, d, *J* 15.6, CH_2Ph), 5.46 (1H, d, *J* 15.6, CH_2Ph), 6.97–7.01 (1H, m, Ar-C*H*), 7.08–7.10 (1 H, m, Ar-C*H*), 7.21–7.24 (3H, m, Ar-C*H*), 7.27–7.41 (8H, m, Ar-C*H*).

1-Benzyl-3-(4-methoxyphenyl)indolin-2-one (42)

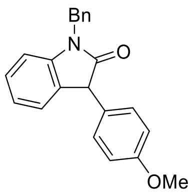

^{1}HNMR ($CDCl_3$, 400 MHz) δ_H: 3.83 (3H, s, OCH_3), 4.69 (1H, s, C(3)*H*), 4.92 (1H, d, *J* 15.6, CH_2Ph), 5.02 (1H, d, *J* 15.6, CH_2Ph), 6.81 (1H, d, *J* 7.8, Ar-C*H*), 6.90–6.93 (2H, m, Ar-C*H*), 7.03–7.07 (1H, m,

Ar-C*H*), 7.16–7.35 (9H, m, Ar-C*H*).

3-(Benzo[d][1,3]dioxol-5-yl)-1-benzylindolin-2-one (43)

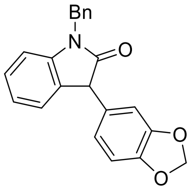

¹HNMR ($CDCl_3$, 400 MHz) δ_H: 4.64 (1H, s, C(3)*H*), 4.92 (1H, d, *J* 15.6, CH_2Ph), 5.01 (1H, d, *J* 15.6, CH_2Ph), 5.96 (2H, dd, *J* 3.1, 1.4, OCH_2O), 6.67 (1H, d, *J* 1.7, Ar-C*H*), 6.76 (1H, dd, *J* 8.0, 1.7, Ar-C*H*), 6.80-6.83 (2H, m, Ar-C*H*), 7.03–7.07 (1H, m, Ar-C*H*), 7.18–7.25 (2H, m, Ar-C*H*), 7.28–7.37 (5H, m, Ar-C*H*).

1-Benzyl-3-(naphthalen-2-yl)indolin-2-one (44)

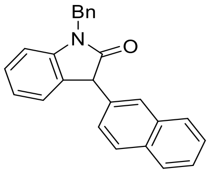

¹HNMR ($CDCl_3$, 400 MHz) δ_H: 4.91 (1H, s, C(3)*H*), 4.96 (1H, d, *J* 15.7, CH_2Ph), 5.08 (1H, d, *J* 15.7, CH_2Ph), 6.87 (1H, d, *J* 7.8, Ar-C*H*), 7.05–7.09 (1H, m, Ar-C*H*), 7.21–7.41 (8H, m, Ar-C*H*), 7.49–7.53 (2H, m, Ar-C*H*). 7.78 (1H, m, Ar-C*H*), 7.82–7.87 (3H, m, Ar-C*H*).

1-Benzyl-3-(4-(trifluoromethyl)phenyl)indolin-2-one (45)

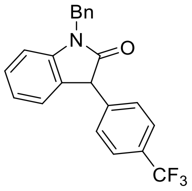

¹HNMR ($CDCl_3$, 400 MHz) δ_H: 4.80 (1H, s, C(3)*H*), 4.93 (1H, d, *J* 15.7, CH_2Ph), 5.03 (1H, d, *J* 15.7, CH_2Ph), 6.86 (1H, d, *J* 8.0, Ar-C*H*), 7.06–7.10 (1H, m, Ar-C*H*), 7.17-7.19 (1H, m, Ar-C*H*), 7.25-7.40 (8H, m, Ar-C*H*), 7.65 (2 H, d, *J* 8.0, Ar-C*H*).

1-Benzyl-3-(4-fluorophenyl)indolin-2-one (46)

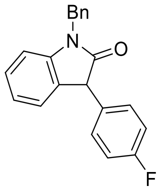

¹HNMR ($CDCl_3$, 400 MHz) δ_H: 4.72 (1H, s, C(3)*H*), 4.93 (1H, d, *J* 15.6, CH_2Ph), 5.02 (1H, d, *J* 15.6 ,

C*H*$_2$Ph), 6.83 (1H, d, *J* 7.8, Ar-C*H*), 7.05–7.10 (3H, m, Ar-C*H*), 7.18–7.38 (9H, m, Ar-C*H*); **^{19}F{^{1}H} NMR (376 MHz, CDCl$_3$)** δ_F: –114.8.

1-Benzyl-3-(3-fluorophenyl)indolin-2-one (47)

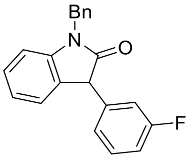

^{1}HNMR (CDCl$_3$, 400 MHz) δ_H: 4.73 (1H, s, C(3)*H*), 4.94 (1H, d, *J* 15.6, C*H*$_2$Ph), 5.02 (1H, d, *J* 15.6, C*H*$_2$Ph), 6.84 (1H, d, *J* 7.8, Ar-C*H*), 6.94–6.97 (1H, m, Ar-C*H*), 7.00–7.10 (3H, m, Ar-C*H*), 7.19–7.38 (8 H, m, Ar-C*H*); **^{19}F{^{1}H} NMR (376 MHz, CDCl$_3$)** δ_F: –112.4.

1-Benzyl-3-(3-methoxyphenyl)indolin-2-one (48)

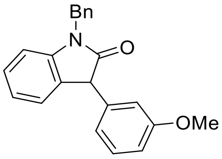

^{1}HNMR (CDCl$_3$, 400 MHz) δ_H: 3.81 (3H, s, OC*H*$_3$), 4.72 (1H, s, C(3)*H*), 4.93 (1H, d, *J* 15.6, C*H*$_2$Ph), 5.04 (1H, d, *J* 15.6, C*H*$_2$Ph), 6.80–6.90 (4H, m, Ar-C*H*), 7.03–7.07 (1H, m, Ar-C*H*), 7.20–7.38 (8H, m, Ar-C*H*).

1-Benzyl-3-(thiophen-2-yl)indolin-2-one (49)

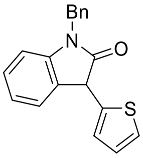

^{1}HNMR (CDCl$_3$, 400 MHz) δ_H: 4.93 (1H, d, *J* 15.6, C*H*$_2$Ph), 5.00 (1H, s, C(3)*H*), 5.02 (1H, d, *J* 15.7, C*H*$_2$Ph), 6.81 (1H, d, *J* 7.8, Ar-C*H*), 7.04 (1H, dd, *J* 5.1, 3.5, Ar-C*H*), 7.07-7.11 (2H, m, Ar-C*H*), 7.24–7.39 (8H, m, Ar-C*H*).

1-Benzyl-3-(2-methoxyphenyl)indolin-2-one (50)

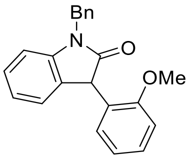

^{1}HNMR (CDCl$_3$, 400 MHz) δ_H: 3.66 (3H, s, OC*H*$_3$), 4.91 (1H, d, *J* 15.6, C*H*$_2$Ph), 4.95 (1H, s, C(3)H), 5.15 (1H, d, *J* 15.6, C*H*$_2$Ph), 6.80 (1H, d, *J* 7.8, Ar-C*H*), 6.92–7.00 (3H, m, Ar-C*H*), 7.07 (1H, d, *J* 7.4, Ar-C*H*), 7.16–7.21 (2H, m, Ar-C*H*), 7.28–7.39 (4H, m, Ar-C*H*), 7.43–7.45 (2H, m, Ar-C*H*).

1-Benzyl-3-(2-fluorophenyl)indolin-2-one (51)

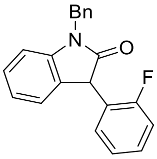

^{1}HNMR (CDCl$_3$, 400 MHz) δ$_H$: 4.96 (1H, d, *J* 15.7, C*H*$_2$Ph), 4.99 (1H, s, C(3)*H*), 5.11 (1H, d, *J* 15.7, C*H*$_2$Ph), 6.81 (1H, d, *J* 7.8, Ar-C*H*), 7.00–7.04 (1H, m, Ar-C*H*), 7.13–7.24 (5H, m, Ar-C*H*), 7.28–7.41 (6H, m, Ar-C*H*); **^{19}F{^{1}H} NMR (376 MHz, CDCl$_3$)** δ$_F$: –116.8.

1-Benzyl-3-(4-(dimethylamino)phenyl)indolin-2-one (52)

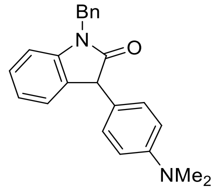

^{1}HNMR (CDCl$_3$, 400 MHz) δ$_H$: 2.96 (6H, s, N(C*H$_3$*)$_2$) 4.65 (1H, s, C(3)*H*), 4.92 (1H, d, *J* 15.7, C*H*$_2$Ph), 5.03 (1H, d, *J* 15.7, C*H*$_2$Ph), 6.74–6.81 (3H, m, Ar-C*H*), 7.02–7.06 (1H, m, Ar-C*H*), 7.10–7.14 (2H, m, Ar-C*H*), 7.20–7.22 (2H, m, Ar-C*H*), 7.28–7.37 (5H, m, Ar-C*H*).

3.6 Data for dihydropyrazol-5-ones

3,4-Dimethyl-1-phenyl-1,4-dihydro-5*H*-pyrazol-5-one (8)

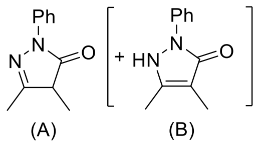

Following General Procedure **6**, the mixture of ethyl 2-methyl-3-oxobutanoate (3.60 g, 25.0 mmol), phenylhydrazine (2.97 g, 27.5 mmol) and EtOH (25 mL) were refluxed overnight to afford the title compound (3.09 g, 66% yield) as a pale-yellow solid; **^{1}H NMR (400 MHz, CDCl$_3$, 1.6:1 A:B)** δ$_H$: 1.44 (A, 3H, s, C(4)C*H$_3$*), 1.70 (B, 3H, s, C(4)C*H$_3$*), 2.07 (B, 3H, s, C(3)C*H$_3$*), 2.17 (A, 3H, s, C(3)C*H$_3$*), 3.24 (A, 1H, q, *J* 8.0, C*H*), 7.08–7.13 (B, 1H, m, PhC(4)*H*), 7.17–7.21 (A, 1H, m, PhC(4)*H*), 7.25–7.29 (B, 2H, m, PhC(3,5)*H*), 7.38–7.43 (A, 2H, m, PhC(3,5)*H*), 7.56–7.59 (B, 2H, m, PhC(2,6)*H*), 7.87–7.91 (A, 2H, m, PhC(2,6)*H*).

1,3,4-Trimethyl-1,4-dihydro-5*H*-pyrazol-5-one (14)

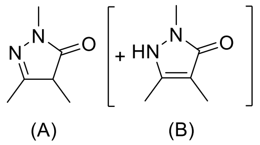

Following General Procedure **6**, the mixture of ethyl 2-methyl-3-oxobutanoate (2.05 g, 14.2 mmol), methylhydrazine (0.72 g, 15.6 mmol) and EtOH (15 mL) were refluxed for 1 d to afford the title compound (1.14 g, 64% yield) as a white solid;**^{1}H NMR (400 MHz, CDCl$_3$, 1:2.2 A:B)** δ$_H$: 1.34 (A, 3H, s, C(4)C*H$_3$*), 1.75 (B, 3H, s, C(4)C*H$_3$*), 2.06 (A, 3H, s, C(3)C*H$_3$*), 2.09 (B, 3H, s, C(3)C*H$_3$*), 2.99 (A, 1H, q, *J* 8.0, C*H*), 3.28 (A, 3H, s, NC*H$_3$*), 3.38 (B, 3H, s, NC*H$_3$*).

3,4-Dimethyl-1-benzyl-1,4-dihydro-5*H*-pyrazol-5-one (15)

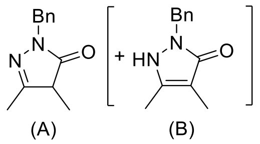

Following General Procedure **6**, the mixture of ethyl 2-methyl-3-oxobutanoate (0.72 g, 5 mmol), benzylhydrazine (1.46 g, 5.5 mmol) and EtOH (5 mL) were refluxed for 1 d to afford the title compound (0.63 g, 62% yield) as a white solid.**^{1}H NMR (400 MHz, CDCl$_3$, 1:1.2 A:B)** δ_H: 1.38 (A, 3H, d, *J* 8.0, C(4)C*H$_3$*), 1.47 (B, 3H, s, C(4)C*H$_3$*), 2.08 (B, 3H, s, C(3)C*H$_3$*), 2.20 (A, 3H, s, C(3)C*H$_3$*), 3.06 (A, 1H, q, *J* 7.9, C*H*), 4.75–4.88 (A, 2H, m, C*H$_2$* and B, 2H, m, C*H$_2$*), 7.30–7.38 (A, 5H, m, C$_6$*H$_5$* and B, 5H, m, C$_6$*H$_5$*).

3-Methyl-4-ethyl-1-phenyl-1,4-dihydro-5*H*-pyrazol-5-one (16)

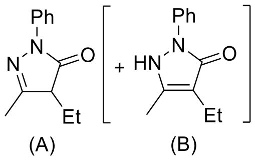

Following General Procedure **6**, ethyl acetoacetate (2.86 g, 22.0 mmol), bromoethane (2.76g, 28.6 mmol), potassium carbonate (3.96 g, 28.6 mmol) and acetone (22 mL) afforded ethyl 2-ethyl-3-oxobutanoate (3.49 g, quant.) as a colourless liquid; Following General Procedure, the mixture of ethyl 2-ethyl-3-oxobutanoate (3.49 g, 37.0 mmol), phenylhydrazine (4.43 g, 41.0 mmol) and EtOH (37 mL) were refluxed for 1 d to afford the title compound (1.96 g, 26% yield) as an orange solid; **^{1}H NMR (400 MHz, CDCl$_3$, 4.3:1 A:B)** δ_H: 0.83 (B, 3H, t, *J* 7.6, CH$_2$C*H$_3$*), 0.93 (A, 3H, t, *J* 7.6, CH$_2$C*H$_3$*), 1.84–2.14 (A, 2H, m, C*H$_2$*CH$_3$ and B, 2H, m, C*H$_2$*CH$_3$), 2.16 (B, 3H, s, C(3)C*H$_3$*), 2.18 (A, 3H, d, *J* 0.7, C(3)C*H$_3$*), 3.27 (A, 1H, tq, *J* 5.4, 0.8, C*H*), 7.17–7.22 (A, 1H, m, PhC(4)*H* and B, 1H, m, PhC(4)*H*), 7.39–7.44 (A, 2H, m, PhC(3,5)*H* and B, 2H, m, PhC(3,5)*H*), 7.87–7.92 (A, 2H, m, PhC(2,6)*H* and B, 2H, m, PhC(2,6)*H*).

3-Methyl-4-allyl-1-phenyl-1,4-dihydro-5*H*-pyrazol-5-one (17)

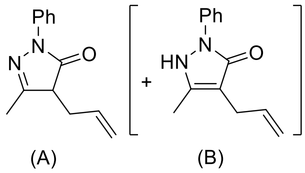

Following General Procedure **6**, ethyl acetoacetate (2.60 g, 20.0 mmol), 3-bromoprop-1-ene (3.40 g, 26.0 mmol), potassium carbonate (3.60 g, 26.0 mmol) and acetone (20 mL) afforded ethyl 2-acetylpent-4-enoate (1.20 g, 30% yield) as a colourless liquid; Following General Procedure, the mixture of ethyl 2-acetylpent-4-enoate (0.85 g, 5.0 mmol), phenylhydrazine (0.59 g, 5.5 mmol) and EtOH (5 mL) were refluxed for 1 d to afford the title compound (0.72 g, 68% yield) as an orange oil; **^{1}H NMR (400 MHz, CDCl$_3$, 5.5:1 A:B)** δ_H: 2.09 (A, 3H, s, C*H$_3$*), 2.67–2.81 (A, 2H, C*H$_2$*), 3.36 (A, 1H, t, *J* 5.7, C*H*), 5.11–5.24 (A, 2H, m, CH=C*H$_2$*), 5.64–5.74 (A, 1H, C*H*=CH$_2$), 7.18–7.22 (A, 1H, m, PhC(4)*H*), 7.39–7.44 (A, 2H, m, PhC(3,5)*H*), 7.89–7.92 (A, 2H, m, PhC(2,6)*H*).

3-Methyl-4-benzyl-1-phenyl-1,4-dihydro-5*H*-pyrazol-5-one (18)

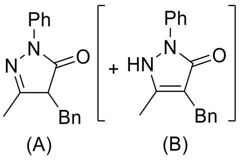

Following General Procedure **6**, ethyl 3-oxobutanoate (2.60 g, 20 mmol), benzyl bromide (4.45 g, 26 mmol), potassium carbonate (3.60 g, 26 mmol) and acetone (20 mL) afforded ethyl 2-benzyl-3-oxobutanoate (3.07 g, 70% yield) as a colourless liquid; Following General Procedure, the mixture of ethyl 2-benzyl-3-oxobutanoate (3.07 g, 14 mmol), phenylhydrazine (1.67 g, 15.4 mmol) and EtOH (14 mL) were refluxed overnight to afford the title compound (1.04 g, 28% yield) as a yellow solid; **^{1}H NMR (400 MHz, $CDCl_3$, 1:1.6 A:B)** δ_H: 1.90 (B, 3H, s, C(3)C*H$_3$*), 2.06 (A, 3H, s, C(3)C*H$_3$*), 3.24 (A, 1H, dd, *J* 14.3, 6.9, C*H^A*H^B), 3.31 (A, 1H, dd, *J* 14.3, 6.9, CH^A*H^B*), 3.46 (B, 2H, s, C*H$_2$*), 3.53–3.56 (A, 1H, m, C*H*), 7.03–7.07 (B, 1H, m, Ar*H*), 7.11–7.31 (A, 6H, m, Ar*H* and B, 7H, m, Ar*H*), 7.36–7.41 (A, 2H, m, Ar*H*), 7.48–7.51 (B, 2H, m, Ar*H*), 7.78–7.81 (A, 2H, m, Ar*H*).

4-Benzyl-1,3-diphenyl-1,4-dihydro-5*H*-pyrazol-5-one (19)

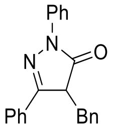

Following General Procedure **6**, ethyl 3-oxo-3-phenylpropanoate (3.84 g, 20 mmol), benzyl bromide (4.45 g, 26 mmol), potassium carbonate (3.60 g, 26 mmol) and acetone (20 mL) afforded ethyl 2-benzyl-3-oxo-3-phenylpropanoate (5.95 g, quant.) as a colourless liquid. Following General Procedure, the mixture of ethyl 2-benzyl-3-oxo-3-phenylpropanoate (5.95 g, 21.0 mmol), phenylhydrazine (2.50 g, 23.1 mmol) and EtOH (21 mL) were refluxed for 1 d to afford the title compound (1.61 g, 24 % yield) as a yellow solid; **^{1}H NMR (400 MHz, $CDCl_3$)** δ_H: 3.38 (1H, dd, *J* 13.8, 5.5, C*H^A*H^B), 3.51 (1H, dd, *J* 13.8, 5.5, CH^A*H^B*), 4.14 (1H, dd, *J* 5.5, 4.8, C*H*), 6.92–6.94 (2H, m, C(4)PhC(2,6)*H*), 7.09–7.14 (3H, m, C(4)PhC(3,4,5)*H*), 7.18–7.22 (1H, m, N(1)PhC(4)*H*), 7.37–7.42 (2H, m, N(1)PhC(3,5)*H*), 7.48–7.52 (3H, m, C(3)PhC(3,4,5)*H*), 7.72–7.74 (2H, m, N(1)PhC(2,6)*H*), 7.76–7.79 (2H, m, C(3)PhC(2,6)*H*).

3-Methyl-1-(2-methylbenzyl)-1-phenyl-1,4-dihydro-5*H*-pyrazol-5-one (20)

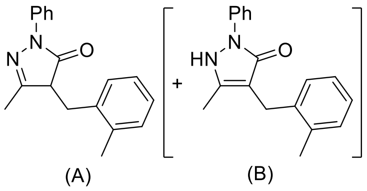

Following General Procedure **6**, ethyl acetoacetate (2.60 g, 20.0 mmol), 1-(bromomethyl)-2-methylbenzene (4.81 g, 26.0 mmol), potassium carbonate (3.60 g, 26.0 mmol) and acetone (20 mL) afforded ethyl 2-(2-methylbenzyl)-3-oxobutanoate (4.69 g, quant.) as a colourless liquid; Following General Procedure, the mixture of ethyl 2-(2-methylbenzyl)-3-oxobutanoate (4.69 g, 20.0 mmol), phenylhydrazine (2.69 g, 22.0 mmol) and EtOH (20 mL) were refluxed for 1 d to afford the title compound (2.5 g, 45% yield) as a yellow solid; **^{1}H NMR (400 MHz, $CDCl_3$, 1:4.2 A:B)** δ_H: 1.78 (B, 3H, s, ArC*H$_3$*), 1.95 (A, 3H, s, ArC*H$_3$*), 2.22 (B, 3H, s, C(3)C*H$_3$*), 2.41 (A, 3H, s, C(3)C*H$_3$*), 3.02–3.08 (A, 1H, m, C*H^A*H^B), 3.34–3.39 (A, 1H, m, CH^A*H^B*), 3.39 (B, 2H, s, C*H$_2$*), 3.54 (A, 1H, dd, *J* 14.8, 9.1, C*H*), 6.82–6.88 (A, 1H, m, Ar*H* and B, 1H, m, Ar*H*), 6.95–6.97 (B, 1H, m, Ar*H*), 7.01–7.29 (A, 4H, m,

Ar*H* and B, 5H, m, Ar*H*), 7.41–7.45 (A, 2H, m, Ar*H*), 7.51–7.53 (B, 2H, m, Ar*H*), 7.89–7.92 (A, 2H, m, Ar*H*).

3-Methyl-1-(3-methylbenzyl)-1-phenyl-1,4-dihydro-5*H*-pyrazol-5-one (21)

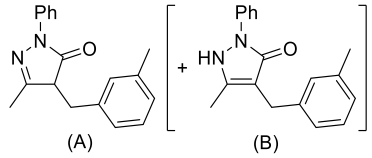

Following General Procedure **6**, ethyl acetoacetate (1.30 g, 10.0 mmol), 1-(bromomethyl)-3-methylbenzene (2.41 g, 13.0 mmol), potassium carbonate (1.80 g, 13.0 mmol) and acetone (10 mL) afforded ethyl 2-(3-methylbenzyl)-3-oxobutanoate (2.07 g, 88% yield) as a colourless liquid; Following General Procedure, the mixture of ethyl 2-(3-methylbenzyl)-3-oxobutanoate (2.11 g, 9.0 mmol), phenylhydrazine (1.21 g, 9.9 mmol) and EtOH (9 mL) were refluxed for 1 d to afford the title compound (1.44 g, 57% yield) as an orange solid; **^{1}H NMR (400 MHz, CDCl$_3$, 3.4:1 A:B)** δ_H: 1.95 (A, 3H, s, ArC*H*$_3$), 2.05 (B, 3H, s, ArC*H*$_3$), 2.21 (A, 3H, s, C(3)C*H*$_3$), 2.23 (B, 3H, s, C(3)C*H*$_3$), 3.11 (A, 1H, dd, *J* 14.3, 7.1, C$H^A$$H^B$), 3.19 (A, 1H, dd, *J* 14.3, 5.3, C$H^A$$H^B$), 3.46 (A, 1H, t, *J* 6.0, C*H*), 3.55 (B, 2H, s, C*H*$_2$), 6.93–7.11 (A, 5H, m, Ar*H* and B, 5H, m, Ar*H*), 7.28–7.32 (A, 2H, m, Ar*H* and B, 2H, m, Ar*H*), 7.61–7.63 (B, 2H, m, Ar*H*), 7.70–7.73 (A, 2H, m, Ar*H*).

3-Methyl-1-(4-chlorobenzyl)-1-phenyl-1,4-dihydro-5*H*-pyrazol-5-one (22)

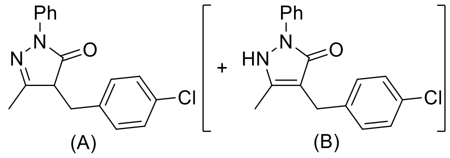

Following General Procedure **6**, ethyl acetoacetate (1.30 g, 10.0 mmol), 1-(bromomethyl)-4-chlorobenzene (2.67 g, 13.0 mmol), potassium carbonate (1.82 g, 13.0 mmol) and acetone (10 mL) afforded ethyl 2-(4-chlorobenzyl)-3-oxobutanoate (1.02 g, 40% yield) as a colourless liquid; Following General Procedure, the mixture of ethyl 2-(4-chlorobenzyl)-3-oxobutanoate (0.61 g, 2.40 mmol), phenylhydrazine (0.32 g, 2.64 mmol) and EtOH (3 mL) were refluxed for 1 d to afford the title compound (0.52 g, 73% yield) as an orange solid; **^{1}H NMR (400 MHz, CDCl$_3$, 2.5:1 A:B)** δ_H: 2.00 (A, 3H, s, C*H*$_3$) 2.08 (B, 3H, s, C*H*$_3$), 3.13–3.22 (A, 2H, m, C*H*$_2$), 3.46 (A, 1H, t, *J* 6.0, C*H*), 3.55 (B, 2H, s, C*H*$_2$), 7.07–7.18 (A, 5H, m, Ar*H* and B, 5H, m, Ar*H*), 7.28–7.34 (A, 2H, m, Ar*H* and B, 2H, m, Ar*H*), 7.59–7.62 (B, 2H, m, Ar*H*), 7.69–7.72 (A, 2H, m, Ar*H*).

3-Methyl-1-(4-cyanobenzyl)-1-phenyl-1,4-dihydro-5*H*-pyrazol-5-one (23)

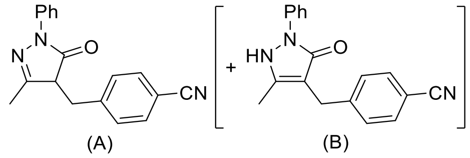

Following General Procedure **6**, ethyl acetoacetate (0.65 g, 5.0 mmol), 4-(bromomethyl)benzonitrile (1.27 g, 6.5 mmol), potassium carbonate (0.90 g, 6.5 mmol) and acetone (5 mL) afforded ethyl 2-(4-cyanobenzyl)-3-oxobutanoate (0.17 g, 14% yield) as a colourless liquid; Following General Procedure, the mixture of ethyl 2-(4-cyanobenzyl)-3-oxobutanoate (0.17 g, 0.7 mmol), phenylhydrazine (0.23 g, 2.1 mmol) and EtOH (2 mL) were refluxed for 1 d to afford the title compound (0.18 g, 89% yield) as an

orange solid; **^{1}H NMR (400 MHz, $CDCl_3$, 1:2.7 A:B)** δ_H: 2.05 (B, 3H, s, C*H₃*) 2.12 (A, 3H, s, CH_3), 3.27–3.41 (A, 2H, m, CH_2), 3.59 (br, A, 1H, C*H* and B, 2H, CH_2), 7.14–7.60 (A, 7H, m, Ar*H* and B, 9H, m, Ar*H*), 7.75–7.77 (A, 2H, m, Ar*H*).

3-Methyl-1,4-diphenyl-1,4-dihydro-5*H*-pyrazol-5-one (24)

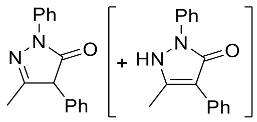

(A) (B)

Following General Procedure **6**, the mixture of ethyl 3-oxo-2-phenylbutanoate (1.03 g, 5 mmol), phenylhydrazine (0.59 g, 5.5 mmol) and EtOH (5 mL) were refluxed for 1 d to afford the title compound (0.70 g, 56% yield) as a white solid. **^{1}H NMR (400 MHz, $CDCl_3$, 1:2.7 A:B)** δ_H: 2.12 (A, 3H, s, C(3)CH_3), 2.30 (B, 3H, s, C(3)CH_3), 4.39 (A, 1H, s, C*H*), 7.18–7.24 (A, 3H, m, Ar*H* and B, 3H, m, Ar*H*), 7.31–7.45 (A, 5H, m, Ar*H* and B, 3H, m, Ar*H*), 7.50–7.52 (B, 2H, m, Ar*H*), 7.68–7.70 (B, 2H, m, Ar*H*), 7.95–7.97 (A, 2H, m, Ar*H*).

4 Michael Addition Products

4.1 Data for Michael adducts with dihydropyrazol-5-ones

(*S*)-*N*-benzyl-3-((*S*)-3,4-dimethyl-5-oxo-1-phenyl-4,5-dihydro-1*H*-pyrazol-4-yl)-4,4,4-trifluorobutanamide (12_{major}) and (*S*)-*N*-benzyl-3-((*R*)-3,4-dimethyl-5-oxo-1-phenyl-4,5-dihydro-1*H*-pyrazol-4-yl)-4,4,4-trifluorobutanamide (12_{minor})

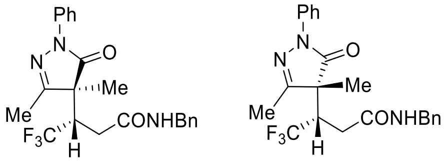

Following General Procedure **7**, 4,5-dimethyl-2-phenyl-2,4-dihydro-3*H*-pyrazol-3-one (37.6 mg, 0.2 mmol), 4-nitrophenyl (*E*)-4,4,4-trifluorobut-2-enoate (52.2 mg, 0.2 mmol) and (2*S*,3*R*)-HyperBTM (12.3 mg, 0.04 mmol) in THF at 0 °C for 24 h. Benzyl amine (109 µL, 1.0 mmol) was added and the reaction stirred at room temperature for 16 h. The crude product (71:29 dr) was purified by flash silica chromatography (67:33 Petrol : EtOAc, R_f 0.21_{major} and 0.33_{minor}) to give:

12_{major} (50.9 mg, 61 %) as a colourless oil. $[\alpha]_D^{20}$ = +31.0 (*c* 1.5, $CHCl_3$); Chiral HPLC analysis, Chiralcel OD-H (95:5 hexane : IPA, flow rate 1 $mLmin^{-1}$, 254 nm, 30 °C) t_R (minor): 31.3 min, t_R (major): 38.3 min, 96:4 er; ν_{max} (film) 3310 (N-H), 2957, 2928 (C-H), 1699 (C=O, amide), 1684 (C=N), 1653 (C=O, lactam), 1172, 1148, 1126 (C-F); **^{1}H NMR (500 MHz, $CDCl_3$)** δ_H: 1.38 (3H, s, C(4)C*H₃*), 2.16 (3H, s, C(3)C*H₃*), 2.51 (1H, dd, *J* 16.2, 5.0, CH^AH^BCO), 2.60 (1H, dd, *J* 16.2, 6.6, CH^AH^BCO), 3.63–3.71 (1H, m, CF_3C*H*), 4.38 (1H, dd, *J* 14.7, 5.8, CH^AH^BPh), 4.44 (1H, dd, *J* 14.7, 5.8, CH^AH^BPh), 6.63 (1H, t, *J* 5.8, N*H*), 7.20–7.34 (6H, m, C*H*-Ar), 7.38–7.41 (2H, m, C*H*-Ar), 7.85–7.87 (2H, m, C*H*-Ar); **^{19}F{^{1}H} NMR (376 MHz, $CDCl_3$)** δ_F: –66.5 (s, C*F₃*); **^{13}C{^{1}H} NMR (101 MHz, $CDCl_3$)** δ_C: 14.9 (C(3)*C*H₃), 19.5 (C(4)*C*H₃), 31.5 (*C*H₂CO), 43.4 (q, $^2J_{CF}$ 26.4, CF_3CH), 44.0 (*C*H₂Ph), 53.3 (*C*(4)), 119.1 (2×*C*H-Ar), 125.5 (*C*H-Ar), 126.6 (q, $^1J_{CF}$ 281.6, *C*F₃), 127.6 (*C*H-Ar), 127.8 (2×*C*H-Ar),128.7 (2×*C*H-Ar), 128.9 (2×*C*H-Ar), 137.7 (*C*-Ar), 137.8 (*C*-Ar), 161.3 (*C*(3)), 168.8 (NH*C*=O), 174.1 (*C*(5)=O); **HRMS (NSI$^+$)** $C_{22}H_{23}F_3N_3O_2$ $[M+H]^+$ found 418.1732, requires 418.1737 (–1.2 ppm).

12_{minor} (21.9 mg, 26 %) as a white solid. mp 145–146 °C; $[\alpha]_D^{20}$ = +29.0 (*c* 1.4, $CHCl_3$); Chiral HPLC analysis, Chiralpak AD-H (95:5 hexane : IPA, flow rate 1 $mLmin^{-1}$, 254 nm, 30 °C) t_R (minor): 17.1 min, t_R (major): 23.1 min, 96:4 er; ν_{max} (film) 3310 (N-H), 2926 (C-H), 1701 (C=O, amide), 1684 (C=N), 1647 (C=O, lactam), 1165, 1124 , 1113 (C-F); **^{1}H NMR (500 MHz, $CDCl_3$)** δ_H: 1.38 (3H, s, C(4)C*H₃*), 2.21 (3H, s, C(3)C*H₃*), 2.68 (1H, dd, *J* 16.9, 5.4, CH^AH^BCO), 3.30 (1H, dd, *J* 16.9, 5.4, CH^AH^BCO), 3.58–3.63 (1H, m, CF_3C*H*), 4.40 (1H, dd, *J* 14.8, 5.8, CH^AH^BPh), 4.48 (1H, dd, *J* 14.8, 5.8, CH^AH^BPh), 6.38 (1H, t, *J* 5.8, N*H*), 7.21–7.35 (6H, m, C*H*-Ar), 7.39–7.42 (2H, m, C*H*-Ar), 7.82–7.84 (2H, m, C*H*-Ar); **^{19}F{^{1}H} NMR (376 MHz, $CDCl_3$)** δ_F: –67.4 (s, C*F₃*); **^{13}C{^{1}H} NMR (101 MHz, $CDCl_3$)** δ_C: 13.7 (C(3)*C*H₃), 20.2 (C(4)*C*H₃), 29.6 (*C*H₂CO), 43.9 (q, $^2J_{CF}$ 25.9, CF_3CH), 44.0 (*C*H₂Ph), 51.2 (*C*(4)), 119.4 (2×*C*H-Ar), 125.6 (*C*H-Ar), 126.3 (q, $^1J_{CF}$ 281.2, *C*F₃), 127.6 (*C*H-Ar), 127.7 (2×*C*H-Ar), 128.7 (2×*C*H-Ar), 128.9 (2×*C*H-Ar), 137.7 (*C*-Ar), 137.8 (*C*-Ar), 162.9 (*C*(3)), 169.7 (NH*C*=O), 173.7 (*C*(5)=O); **HRMS (NSI$^+$)** $C_{22}H_{23}F_3N_3O_2$ $[M+H]^+$ found 418.1735, requires 418.1737 (–0.4 ppm).

(*S*)-*N*-benzyl-4,4,4-trifluoro-3-((*S*)-1,3,4-trimethyl-5-oxo-4,5-dihydro-1*H*-pyrazol-4-yl)butanamide (25_{major}) and (*S*)-*N*-benzyl-4,4,4-trifluoro-3-((*R*)-1,3,4-trimethyl-5-oxo-4,5-dihydro-1*H*-pyrazol-4-yl)butanamide (25_{minor})

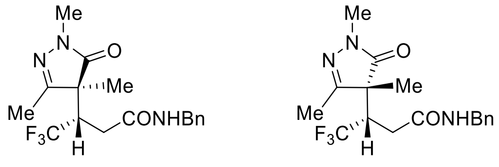

Following General Procedure **7**, pyrazol-one (25.2 mg, 0.2 mmol), 4-nitrophenyl (*E*)-4,4,4-trifluorobut-2-enoate (52.2 mg, 0.2 mmol) and (2*S*,3*R*)-HyperBTM (12.3 mg, 0.04 mmol) in THF at 0 °C for 72 h. Benzyl amine (109 µL, 1.0 mmol) was added and the reaction stirred at room temperature overnight. The crude product (61:39 dr) was purified by flash silica chromatography (50:50 Petrol : EtOAc, R_f 0.26_{major} and 0.41_{minor}) to give:

25_{major} (20.1 mg, 28 %) as a colourless oil. $[\alpha]_D^{20}$ = +21.4 (*c* 0.8, $CHCl_3$); Chiral HPLC analysis, Chiralcel OJ-H (93:7 hexane : IPA, flow rate 1 $mLmin^{-1}$, 254 nm, 30 °C) t_R (minor): 15.8 min, t_R (major): 19.5 min, 92:8 er; ν_{max} (film) 3310 (N-H), 2970, 2932 (C-H), 1697 (C=O, amide), 1684 (C=N), 1647 (C=O, lactam), 1165, 1126 (C-F); **^{1}H NMR (400 MHz, $CDCl_3$)** δ_H: 1.31 (3H, s, C(4)CH_3), 2.09 (3H, s, C(3)CH_3), 2.35 (1H, dd, *J* 16.0, 4.7, C$H^A$$H^B$CO), 2.51 (1H, dd, *J* 16.0, 7.1, C$H^A$$H^B$CO), 3.26 (3H, s, N(1)C$H_3$), 3.47–3.57 (1H, m, CF_3C*H*), 4.41–4.51 (2H, m, CH_2Ph), 6.10 (1H, br, N*H*), 7.27–7.39 (5H, m, C*H*-Ar); **^{19}F{^{1}H} NMR (376 MHz, $CDCl_3$)** δ_F: –66.1 (s, CF_3); **^{13}C{^{1}H} NMR (101 MHz, $CDCl_3$)** δ_C: 14.7 (C(3)*C*H_3), 19.1 (C(4)*C*H_3), 31.2 (N(1)*C*H_3), 31.9 (*C*H_2CO), 43.4 (q, $^2J_{CF}$ 26.4, CF_3*C*H), 44.1 (*C*H_2Ph), 51.7 (*C*(4)), 126.5 (q, $^1J_{CF}$ 281.8, *C*F_3), 127.8 (*C*H-Ar), 127.9 (2×*C*H-Ar), 128.8 (2×*C*H-Ar), 137.7 (*C*-Ar), 160.5 (*C*(3)), 168.5 (NH*C*=O), 175.4 (*C*(5)=O); **HRMS (NSI$^+$)** $C_{17}H_{21}F_3N_3O_2$ $[M+H]^+$ found 356.1583, requires 356.1580 (+0.7 ppm).

25_{minor} (10.4 mg, 15 %) as a white solid. mp 113–114 °C; $[\alpha]_D^{20}$ = +8.9 (*c* 0.5, $CHCl_3$); Chiral HPLC analysis, Chiralpak AD-H (97:3 hexane : IPA, flow rate 1 $mLmin^{-1}$, 254 nm, 30 °C) t_R (minor): 22.6 min, t_R (major): 29.5 min, 96:4 er; ν_{max} (film) 3304 (N-H), 2965, 2932 (C-H), 1699 (C=O, amide), 1684 (C=N), 1653 (C=O, lactam), 1273, 1155, 1126 (C-F); **^{1}H NMR (400 MHz, $CDCl_3$)** δ_H: 1.27 (3H, s, C(4)CH_3), 2.09 (3H, s, C(3)CH_3), 2.61 (1H, dd, *J* 17.0, 5.3, C$H^A$$H^B$CO), 3.15 (3H, s, N(1)C$H_3$), 3.27 (1H, dd, *J* 17.0, 5.6, C$H^A$$H^B$CO), 3.47–3.57 (1H, m, CF_3C*H*), 4.43–4.54 (2H, m, CH_2Ph), 6.68 (1H, *J* 5.5, N*H*), 7.27–7.36 (5H, m, C*H*-Ar); **^{19}F{^{1}H} NMR (376 MHz, $CDCl_3$)** δ_F: –67.7 (s, CF_3); **^{13}C{^{1}H} NMR (101 MHz, $CDCl_3$)** δ_C: 13.5 (C(3)*C*H_3), 19.9 (C(4)*C*H_3), 29.4 (N(1)*C*H_3), 31.0 (*C*H_2CO), 43.5 (q, $^2J_{CF}$ 26.0, CF_3*C*H), 43.9 (*C*H_2Ph), 49.7 (*C*(4)), 126.3 (q, $^1J_{CF}$ 281.3, *C*F_3), 127.6 (*C*H-Ar), 127.7 (2×*C*H-Ar), 128.7 (2×*C*H-Ar), 138.0 (*C*-Ar), 162.4 (*C*(3)), 169.9 (NH*C*=O), 175.1 (*C*(5)=O); **HRMS (NSI$^+$)** $C_{17}H_{21}F_3N_3O_2$ $[M+H]^+$ found 356.1584, requires 356.1580 (+1.0 ppm).

(*S*)-*N*-benzyl-3-((*S*)-1-benzyl-3,4-dimethyl-5-oxo-4,5-dihydro-1*H*-pyrazol-4-yl)-4,4,4-trifluorobutanamide ($\mathbf{26_{major}}$) and (*S*)-*N*-benzyl-3-((*R*)-1-benzyl-3,4-dimethyl-5-oxo-4,5-dihydro-1*H*-pyrazol-4-yl)-4,4,4-trifluorobutanamide ($\mathbf{26_{minor}}$)

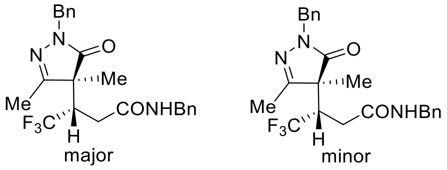

Following General Procedure **7**, pyrazol-one (40.5 mg, 0.2 mmol), 4-nitrophenyl (*E*)-4,4,4-trifluorobut-2-enoate (52.2 mg, 0.2 mmol) and (2*S*,3*R*)-HyperBTM (12.3 mg, 0.04 mmol) in THF at 0 °C for 72 h. Benzyl amine (109 µL, 1.0 mmol) was added and the reaction stirred at room temperature for 16 h. The crude product (64:36 dr) was purified by flash silica chromatography (50:50 Petrol : EtOAc, R_f 0.44_{major} and 0.52_{minor}) to give:

$\mathbf{26_{major}}$ (23.1 mg, 27 %) as a colourless oil. $[\alpha]_D^{20}$ = +20.6 (*c* 0.5, $CHCl_3$); Chiral HPLC analysis, Chiralpak AD-H (90:10 hexane : IPA, flow rate 1 mLmin^{-1}, 254 nm, 30 °C) t_R (major): 16.3 min, t_R (minor): 41.8 min, 97:3 er; ν_{max} (film) 3310 (N-H), 2968, 2934 (C-H), 1697 (C=O, amide), 1684 (C=N), 1647 (C=O, lactam), 1165, 1130 (C-F); **^{1}H NMR (400 MHz, CDCl$_3$)** δ_H: 1.34 (3H, s, C(4)C*H$_3$*), 2.05 (3H, s, C(3)C*H$_3$*), 2.23 (1H, dd, *J* 16.1, 4.7, CH^AH^BCO), 2.45 (1H, dd, *J* 16.1, 6.9, CH^AH^BCO), 3.47–3.57 (1H, m, CF_3CH), 4.38 (1H, dd, *J* 14.7, 5.7, CONHCH^AH^BPh), 4.46 (1H, dd, *J* 14.7, 5.7, CONHCH^AH^BPh), 4.77 (1H, d, *J* 14.9, N(1)CH^AH^BPh), 4.84 (1H, d, *J* 14.9, N(1)CH^AH^BPh), 5.76 (1H, t, *J* 5.7, N*H*), 7.25–7.40 (10H, m, C*H*-Ar); **^{19}F{^{1}H} NMR (376 MHz, CDCl$_3$)** δ_F: –65.6 (s, C*F$_3$*); **^{13}C{^{1}H} NMR (101 MHz, CDCl$_3$)** δ_C: 14.6 (C(3)*C*H$_3$), 19.2 (C(4)*C*H$_3$), 31.9 (*C*H$_2$CO), 42.7 (q, *J* 26.6, CF$_3$*C*H), 44.1 (CONH*C*H$_2$Ph), 48.0 (N*C*H$_2$Ph), 51.9 (*C*(4)), 125.1 (*C*F$_3$), 127.77 (*C*H-Ar), 127.81 (*C*H-Ar), 127.9 (2×*C*H-Ar), 128.3 (2×*C*H-Ar), 128.7 (2×*C*H-Ar), 128.8 (2×*C*H-Ar), 136.5 (*C*-Ar), 137.6 (*C*-Ar), 160.7 (*C*(3)), 168.4 (NH*C*=O), 175.0 (*C*(5)=O); **HRMS (NSI$^+$)** $C_{23}H_{25}F_3N_3O_2$ $[M+H]^+$ found 432.1891, requires 432.1893 (–0.6 ppm).

$\mathbf{26_{minor}}$ (15.3 mg, 18 %) as a colourless oil. $[\alpha]_D^{20}$ = +11.5 (*c* 0.5, $CHCl_3$); Chiral HPLC analysis, Chiralpak AD-H (93:7 hexane : IPA, flow rate 1 mLmin^{-1}, 254 nm, 30 °C) t_R (minor): 19.0 min, t_R (major): 39.7 min, 97:3 er; ν_{max} (film) 3310 (N-H), 2926 (C-H), 1699 (C=O, amide), 1684 (C=N), 1645 (C=O, lactam), 1271, 1155, 1126 (C-F); **^{1}H NMR (400 MHz, CDCl$_3$)** δ_H: 1.30 (3H, s, C(4)C*H$_3$*), 2.09 (3H, s, C(3)C*H$_3$*), 2.60 (1H, dd, *J* 16.9, 5.2, CH^AH^BCO), 3.29 (1H, dd, *J* 16.9, 5.7, CH^AH^BCO), 3.48–3.57 (1H, m, CF_3CH), 4.50 (2H, d, *J* 5.7, NC*H$_2$*Ph), 4.76 (1H, d, *J* 15.2, NCH^AH^BPh), 4.83 (1H, d, *J* 15.2, NCH^AH^BPh), 6.06 (1H, t, *J* 5.1, N*H*), 7.27–7.39 (10H, m, C*H*-Ar); **^{19}F{^{1}H} NMR (376 MHz, CDCl$_3$)** δ_F: –67.4 (s, C*F$_3$*); **^{13}C{^{1}H} NMR (101 MHz, CDCl$_3$)** δ_C: 13.6 (C(3)*C*H$_3$), 20.2 (C(4)*C*H$_3$), 29.6 (*C*H$_2$CO), 43.4 (q, *J* 26.1, CF$_3$*C*H), 44.1 (CONH*C*H$_2$Ph), 47.8 (N*C*H$_2$Ph), 49.8 (*C*(4)), 124.9 (*C*F$_3$), 127.70 (*C*H-Ar), 127.74 (*C*H-Ar), 127.8 (C×*C*H-Ar), 127.9 (2×*C*H-Ar), 128.6 (2×*C*H-Ar), 128.8 (2×*C*H-Ar), 136.2 (*C*-Ar), 137.7 (*C*-Ar), 162.4 (*C*(3)), 169.6 (NH*C*=O), 174.9 (*C*(5)=O); **HRMS (NSI$^+$)** $C_{23}H_{25}F_3N_3O_2$ $[M+H]^+$ found 432.1892, requires 432.1893 (–0.3 ppm).

(*S*)-*N*-benzyl-3-((*S*)-4-ethyl-3-methyl-5-oxo-1-phenyl-4,5-dihydro-1*H*-pyrazol-4-yl)-4,4,4-trifluorobutanamide (27_{major}) and (*S*)-*N*-benzyl-3-((*R*)-4-ethyl-3-methyl-5-oxo-1-phenyl-4,5-dihydro-1*H*-pyrazol-4-yl)-4,4,4-trifluorobutanamide (27_{minor})

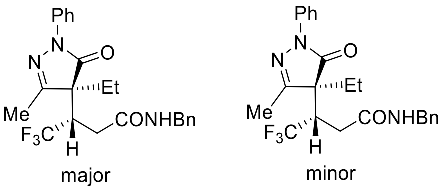

Following General Procedure **7**, 4-ethyl-5-methyl-2-phenyl-2,4-dihydro-3*H*-pyrazol-3-one (40.5 mg, 0.2 mmol), 4-nitrophenyl (*E*)-4,4,4-trifluorobut-2-enoate (52.2 mg, 0.2 mmol) and (2*S*,3*R*)-HyperBTM (12.3 mg, 0.04 mmol) in THF at 0 °C for 72 h. Benzyl amine (109 µL, 1.0 mmol) was added and the reaction stirred at room temperature for 16 h. The crude product (66:34 dr) was purified by flash silica chromatography (67:33 Petrol : EtOAc, R_f 0.29_{major} and 0.43_{minor}) to give:

27_{major} (38.3 mg, 44 %) as a colourless oil. $[\alpha]_D^{20}$ = –4.9 (*c* 1.7, $CHCl_3$); Chiral HPLC analysis, Chiralpak AD-H (90:10 hexane : IPA, flow rate 1 mLmin^{-1}, 254 nm, 30 °C) t_R (major): 15.0 min, t_R (minor): 41.9 min, 96:4 er; ν_{max} (film) 3310 (N-H), 2959, 2930 (C-H), 1699 (C=O, amide), 1684 (C=N), 1653 (C=O, lactam), 1171, 1142, 1121 (C-F); **^{1}H NMR (400 MHz, $CDCl_3$)** δ_H: 0.73 (3H, t, *J* 7.4, CH_2CH_3), 1.78 (1H, dq, *J* 14.4, 7.4, $CH^AH^BCH_3$), 1.97 (1H, dq, *J* 14.4, 7.4, $CH^AH^BCH_3$), 2.14 (3H, s, C(3)CH_3), 2.48–2.60 (2H, m, CH_2CO), 3.65–3.74 (1H, m, CF_3CH), 4.40–4.49 (2H, m, CH_2Ph), 6.29 (1H, t, *J* 5.8, N*H*), 7.19–7.36 (6H, m, C*H*-Ar), 7.38–7.43 (2H, m, C*H*-Ar), 7.84–7.87 (2H, m, C*H*-Ar); **^{19}F{^{1}H} NMR (376 MHz, $CDCl_3$)** δ_F: –66.0 (s, CF_3); **^{13}C{^{1}H} NMR (101 MHz, $CDCl_3$)** δ_C: 7.5 (C(4)CH_2CH_3), 15.1 (C(3)CH_3), 25.8 (C(4)CH_2CH_3), 31.6 (CH_2CO), 43.8 (q, $^2J_{CF}$ 26.2, CF_3CH), 44.0 (CH_2Ph), 58.2 (*C*(4)), 119.1 (2×*C*H-Ar), 125.5 (*C*H-Ar), 126.6 (q, $^1J_{CF}$ 281.7, CF_3), 127.7 (*C*H-Ar), 127.8 (2×*C*H-Ar), 128.79 (2×*C*H-Ar), 128.9 (2×*C*H-Ar), 137.5 (*C*-Ar), 137.7 (*C*-Ar), 159.5 (*C*(3)), 168.6 (NH*C*=O), 173.3 (*C*(5)=O); **HRMS (NSI$^+$)** $C_{23}H_{25}F_3N_3O_2$ [M+H]+ found 432.1890, requires 432.1893 (–0.8 ppm).

27_{minor} (23.4 mg, 27 %) as a colourless oil. $[\alpha]_D^{20}$ = +47.7 (*c* 1.0, $CHCl_3$); Chiral HPLC analysis, Chiralpak AD-H (97:3 hexane : IPA, flow rate 1 mLmin^{-1}, 254 nm, 30 °C) t_R (major): 30.9 min, t_R (minor): 36.4 min, 98:2 er; ν_{max} (film) 3310 (N-H), 2963, 2926 (C-H), 1699 (C=O, amide), 1684 (C=N), 1645 (C=O, lactam), 1267, 1165, 1117 (C-F); **^{1}H NMR (400 MHz, $CDCl_3$)** δ_H: 0.68 (3H, t, *J* 7.4, CH_2CH_3), 1.76 (1H, dq, *J* 13.4, 7.4, $CH^AH^BCH_3$), 1.95 (1H, dq, *J* 13.4, 7.4, $CH^AH^BCH_3$), 2.18 (3H, s, C(3)CH_3), 2.62 (1H, dd, *J* 17.0, 5.4, CH^AH^BCO), 3.36 (1H, dd, *J* 17.0, 5.4, CH^AH^BCO), 3.58–3.68 (1H, m, CF_3CH), 4.42 (1H, dd, *J* 14.8, 5.8, CH^AH^BPh), 4.50 (1H, dd, *J* 14.8, 5.8, C^AH^BPh), 6.25 (1H, t, *J* 5.9, N*H*), 7.21–7.37 (6H, m, C*H*-Ar), 7.39–7.44 (2H, m, C*H*-Ar), 7.80–7.83 (2H, m, C*H*-Ar); **^{19}F{^{1}H} NMR (376 MHz, $CDCl_3$)** δ_F: –67.0 (s, CF_3); **^{13}C{^{1}H} NMR (101 MHz, $CDCl_3$)** δ_C: 7.5 (C(4)CH_2CH_3), 14.1 (C(3)CH_3), 26.4 (C(4)CH_2CH_3), 29.6 (CH_2CO), 43.9 (q, $^2J_{CF}$ 26.2, CF_3CH), 44.0 (CH_2Ph), 56.4 (*C*(4)), 119.5 (2×*C*H-Ar), 125.6 (*C*H-Ar), 126.4 (q, $^1J_{CF}$ 281.3, CF_3), 127.7 (*C*H-Ar), 127.8 (2×*C*H-Ar), 128.8 (2×*C*H-Ar), 128.9 (2×*C*H-Ar), 137.5 (*C*-Ar), 137.8 (*C*-Ar), 161.2 (*C*(3)), 169.7 (NH*C*=O), 173.0 (*C*(5)=O); **HRMS (NSI$^+$)** $C_{23}H_{25}F_3N_3O_2$ [M+H]$^+$ found 432.1891, requires 432.1893 (–0.6 ppm).

(*S*)-3-((*S*)-4-allyl-3-methyl-5-oxo-1-phenyl-4,5-dihydro-1*H*-pyrazol-4-yl)-N-benzyl-4,4,4-trifluorobutanamide (28$_{major}$) and (*S*)-3-((*R*)-4-allyl-3-methyl-5-oxo-1-phenyl-4,5-dihydro-1*H*-pyrazol-4-yl)-N-benzyl-4,4,4-trifluorobutanamide (28$_{minor}$)

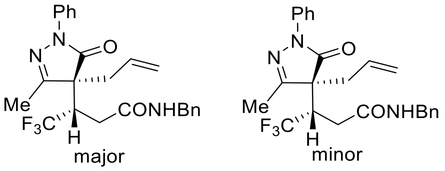

Following General Procedure **7**, 4-allyl-5-methyl-2-phenyl-2,4-dihydro-3*H*-pyrazol-3-one (42.8 mg, 0.2 mmol), 4-nitrophenyl (*E*)-4,4,4-trifluorobut-2-enoate (52.2 mg, 0.2 mmol) and (2*S*,3*R*)-HyperBTM (12.3 mg, 0.04 mmol) in THF at room temperature for 6 h. Benzyl amine (109 µL, 1.0 mmol) was added and the reaction stirred at room temperature for 16 h. The crude product (62:38 dr) was purified by flash silica chromatography (67:33 Petrol : EtOAc, R_f 0.20$_{major}$ and 0.40$_{minor}$) to give:

28$_{major}$ (39.1 mg, 44 %) as a pale-yellow oil. $[\alpha]_D^{20}$ = –29.4 (*c* 1.7, $CHCl_3$); Chiral HPLC analysis, Chiralpak AD-H (90:10 hexane : IPA, flow rate 1 mLmin^{-1}, 254 nm, 30 °C) t_R (major): 16.1 min, t_R (minor): 21.9 min, 96:4 er; ν_{max} (film) 3312 (N-H), 2967, 2932 (C-H), 1701 (C=O, amide), 1684 (C=N), 1651 (C=O, lactam), 1169, 1121 (C-F); **^{1}H NMR (400 MHz, CDCl$_3$)** δ_H: 2.19 (3H, s, C(3)C*H$_3$*), 2.44–2.60 (3H, m, C(4)C*H$_2$*CH=CH$_2$ and CH^A*H^B*CO), 2.75 (1H, dd, *J* 13.4, 6.9, CH^A*H^B*CO), 3.65–3.75 (1H, m, CF$_3$C*H*), 4.42–4.51 (2H, m, C*H$_2$*Ph), 5.08–5.11 (1H, m, CH=C*H^A*H^B), 5.15–5.19 (1H, m, CH=CH^A*H^B*), 5.33–5.44 (1H, m, C*H*=CH$_2$), 5.99 (1H, br, N*H*), 7.19–7.42 (8H, m, C*H*-Ar), 7.83–7.85 (2H, m, C*H*-Ar); **^{19}F{^{1}H} NMR (376 MHz, CDCl$_3$)** δ_F: –65.7 (s, C*F$_3$*); **^{13}C{^{1}H} NMR (101 MHz, CDCl$_3$)** δ_C: 15.3 (C(3)*C*H$_3$), 31.8 (C(4)*C*H$_2$CH=CH$_2$), 36.9 (*C*H$_2$CO), 43.3 (q, $^2J_{CF}$ 26.5, CF$_3$*C*H), 44.2 (*C*H$_2$Ph), 57.8 (*C*(4)), 119.1 (2×*C*H-Ar), 121.2 (CH=*C*H$_2$), 125.4 (*C*H-Ar), 126.5 (q, $^1J_{CF}$ 281.6, *C*F$_3$), 127.8 (*C*H-Ar), 127.9 (2×*C*H-Ar), 128.8 (2×*C*H-Ar), 128.9 (2×*C*H-Ar), 129.1 (*C*H=CH$_2$), 137.5 (*C*-Ar), 137.6 (*C*-Ar), 159.0 (*C*(3)), 168.3 (NH*C*=O), 172.6 (*C*(5)=O); **HRMS (NSI$^+$)** $C_{24}H_{25}F_3N_3O_2$ [M+H]+ found 444.1891, requires 444.1893 (–0.5 ppm).

28$_{minor}$ (23.1 mg, 26 %) as a pale-yellow oil. $[\alpha]_D^{20}$ = +72.5 (*c* 0.6, $CHCl_3$); Chiral HPLC analysis, Chiralcel OD-H (97:3 hexane : IPA, flow rate 1 mLmin^{-1}, 254 nm, 30 °C) t_R (major): 15.2 min, t_R (minor): 24.7 min, 96:4 er; ν_{max} (film) 3308 (N-H), 2926 (C-H), 1699 (C=O, amide), 1684 (C=N), 1651 (C=O, lactam), 1261, 1165, 1115 (C-F); **^{1}H NMR (400 MHz, CDCl$_3$)** δ_H: 2.19 (3H, s, C(3)C*H$_3$*), 2.52 (1H, dd, *J* 13.4, 7.7, C*H^A*H^{B}CO), 2.62–2.68 (2H, m, C(4)C*H$_2$*CH=CH$_2$), 3.39 (1H, dd, *J* 17.0, 5.5, CH^A*H^B*CO), 3.62–3.67 (1H, m, CF$_3$C*H*), 4.42 (1H, dd, *J* 14.8, 5.8, C*H^A*H^{B}Ph), 4.51 (1H, dd, *J* 14.8, 5.8, CH^A*H^B*Ph), 5.08–5.11 (1H, m, CH=C*H^A*H^B), 5.12–5.17 (1H, m, CH=CH^A*H^B*), 5.28–5.38 (1H, m, C*H*=CH$_2$), 6.23 (1H, t, *J* 5,8, N*H*), 7.20–7.43 (8H, m, C*H*-Ar), 7.78–7.81 (2H, m, C*H*-Ar); **^{19}F{^{1}H} NMR (376 MHz, CDCl$_3$)** δ_F: –66.9 (s, C*F$_3$*); **^{13}C{^{1}H} NMR (101 MHz, CDCl$_3$)** δ_C: 14.4 (C(3)*C*H$_3$), 29.5 (C(4)*C*H$_2$CH=CH$_2$), 37.6 (*C*H$_2$CO), 43.5 (q, $^2J_{CF}$ 26.1, CF$_3$*C*H), 44.1 (*C*H$_2$Ph), 55.7 (*C*(4)), 119.6 (2×*C*H-Ar), 121.2 (CH=*C*H$_2$), 125.6 (*C*H-Ar), 126.3 (q, $^1J_{CF}$ 281.3, *C*F$_3$), 127.7 (*C*H-Ar), 127.8 (2×*C*H-Ar), 128.8 (2×*C*H-Ar), 128.9 (2×*C*H-Ar), 129.0 (*C*H=CH$_2$), 137.4 (*C*-Ar), 137.7 (*C*-Ar), 160.8 (*C*(3)), 169.5 (NH*C*=O), 172.7 (*C*(5)=O); **HRMS (NSI$^+$)** $C_{24}H_{25}F_3N_3O_2$ $[M+H]^+$ found 444.1890, requires 444.1893 (–0.8 ppm).

(*S*)-*N*-benzyl-3-((*S*)-4-benzyl-3-methyl-5-oxo-1-phenyl-4,5-dihydro-1*H*-pyrazol-4-yl)-4,4,4-trifluorobutanamide ($\mathbf{29_{major}}$) and (*S*)-*N*-benzyl-3-((*R*)-4-benzyl-3-methyl-5-oxo-1-phenyl-4,5-dihydro-1*H*-pyrazol-4-yl)-4,4,4-trifluorobutanamide ($\mathbf{29_{minor}}$)

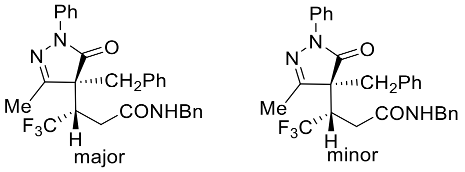

Following General Procedure **7**, 4-benzyl-5-methyl-2-phenyl-2,4-dihydro-3*H*-pyrazol-3-one (52.9 mg, 0.2 mmol), 4-nitrophenyl (*E*)-4,4,4-trifluorobut-2-enoate (52.2 mg, 0.2 mmol) and (2*S*,3*R*)-HyperBTM (12.3 mg, 0.04 mmol) in THF at room temperature for 24 h. Benzyl amine (109 µL, 1.0 mmol) was added and the reaction stirred at room temperature for 16 h. The crude product (57:43 dr) was purified by flash silica chromatography (67:33 Petrol : EtOAc, R_f 0.33_{major} and 0.55_{minor}) to give:

$\mathbf{29_{major}}$ (44.4 mg, 45 %) as a white solid. mp 158–159 °C; $[\alpha]_D^{20}$ = –80.2 (*c* 1.0, $CHCl_3$); Chiral HPLC analysis, Chiralpak AD-H (85:15 hexane : IPA, flow rate 1 $mLmin^{-1}$, 254 nm, 30 °C) t_R (major): 11.7 min, t_R (minor): 21.2 min, 99:1 er; ν_{max} (film) 3294 (N-H), 2957, 2928 (C-H), 1699 (C=O, amide), 1684 (C=N), 1645 (C=O, lactam), 1263, 1165, 1126 (C-F); **^{1}H NMR (400 MHz, $CDCl_3$)** δ_H: 2.17 (3H, s, C(3)CH_3), 2.52 (1H, dd, *J* 16.2, 4.8, CH^AH^BCO), 2.58 (1H, dd, *J* 16.2, 6.6, CH^AH^BCO), 2.96 (1H, d, *J* 13.0, C(4)CH^AH^BPh), 3.16 (1H, d, *J* 13.0, C(4)CH^AH^BPh), 3.72–3.82 (1H, m, CF_3C*H*), 4.31–4.41 (2H, m, CONHCH_2Ph), 6.33 (1H, t, *J* 5.7, N*H*), 6.94–6.97 (2H, m, C*H*-Ar), 7.01–7.05 (4H, m, C*H*-Ar), 7.14–7.23 (7H, m, C*H*-Ar), 7.27–7.30 (2H, m, C*H*-Ar); **^{19}F{^{1}H} NMR (376 MHz, $CDCl_3$)** δ_F: –65.5 (s, CF_3); **^{13}C{^{1}H} NMR (101 MHz, $CDCl_3$)** δ_C: 15.9 (C(3)*C*H_3), 31.8 (*C*H_2CO), 38.8 (C(4)*C*H_2Ph), 43.7 (q, $^2J_{CF}$ 26.4, CF_3*C*H), 44.1 (CONH*C*H_2Ph), 59.5 (*C*(4)), 119.9 (2×*C*H-Ar), 125.7 (*C*H-Ar), 126.6 (q, $^1J_{CF}$ 281.8, *C*F_3), 127.7 (2×*C*H-Ar), 127.8 (2×*C*H-Ar), 128.3 (2×*C*H-Ar), 128.7 (2×*C*H-Ar), 128.8 (2×*C*H-Ar), 129.4 (2×*C*H-Ar), 132.6 (*C*-Ar), 136.9 (*C*-Ar), 137.7 (*C*-Ar), 158.7 (*C*(3)), 168.6 (NH*C*=O), 172.8 (*C*(5)=O); **HRMS (NSI$^+$)** $C_{28}H_{27}F_3N_3O_2$ $[M+H]^+$ found 494.2041, requires 494.2050 (–1.8 ppm).

$\mathbf{29_{minor}}$ (25.5 mg, 26 %) as a white solid. mp 113–115 °C; $[\alpha]_D^{20}$ = +138.9 (*c* 1.2, $CHCl_3$); Chiral HPLC analysis, Chiralpak AD-H (90:10 hexane : IPA, flow rate 1 $mLmin^{-1}$, 254 nm, 30 °C) t_R (minor): 23.4 min, t_R (major): 28.4 min, 95:5 er; ν_{max} (film) 3310 (N-H), 2924 (C-H), 1699 (C=O, amide), 1684 (C=N), 1653 (C=O, lactam), 1263, 1165, 1117 (C-F); **^{1}H NMR (500 MHz, $CDCl_3$)** δ_H: 2.22 (3H, s, C(3)CH_3), 2.75 (1H, dd, *J* 17.1, 5.0, CH^AH^BCO), 3.01 (1H, d, *J* 13.2, C(4)CH^AH^BPh), 3.33 (1H, d, *J* 13.2, C(4)CH^AH^BPh), 3.57 (1H, dd, *J* 17.1, 5.6, CH^AH^BCO), 3.75–3.85 (1H, m, CF_3C*H*), 4.43–4.53 (2H, m, CONHCH_2Ph), 6.35 (1H, t, *J* 5.8, N*H*), 7.04–7.06 (2H, m, C*H*-Ar), 7.14–7.36 (11H, m, C*H*-Ar), 7.45–7.48 (2H, m, C*H*-Ar); **^{19}F{^{1}H} NMR (376 MHz, $CDCl_3$)** δ_F: –66.6 (s, CF_3); **^{13}C{^{1}H} NMR (101 MHz, $CDCl_3$)** δ_C: 14.8 (C(3)*C*H_3), 29.8 (*C*H_2CO), 39.4 (C(4)*C*H_2Ph), 43.9 (q, $^2J_{CF}$ 26.1, CF_3*C*H), 44.1 (CONH*C*H_2Ph), 57.2 (*C*(4)), 120.4 (2×*C*H-Ar), 125.5 (q, $^1J_{CF}$ 281.4, *C*F_3), 125.9 (*C*H-Ar), 127.66 (*C*H-Ar), 127.72 (2×*C*H-Ar), 127.8 (*C*H-Ar), 128.4 (2×*C*H-Ar), 128.8 (4×*C*H-Ar), 129.2 (2×*C*H-Ar), 132.8 (*C*-Ar), 137.0 (*C*-Ar), 137.7 (*C*-Ar), 160.5 (*C*(3)), 169.7 (NH*C*=O), 172.9 (*C*(5)=O); **HRMS (NSI$^+$)** $C_{28}H_{27}F_3N_3O_2$ $[M+H]^+$ found 494.2044, requires 494.2050 (–1.2 ppm).

(*S*)-*N*-benzyl-3-((*S*)-4-benzyl-5-oxo-1,3-diphenyl-4,5-dihydro-1*H*-pyrazol-4-yl)-4,4,4-trifluorobutanamide (30major) and (*S*)-*N*-benzyl-3-((*R*)-4-benzyl-5-oxo-1,3-diphenyl-4,5-dihydro-1*H*-pyrazol-4-yl)-4,4,4-trifluorobutanamide (30minor)

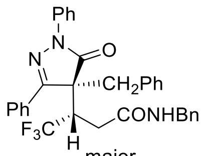

major

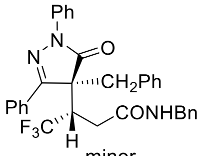

minor

Following General Procedure **7**, pyrazol-one (65.3 mg, 0.2 mmol), 4-nitrophenyl (*E*)-4,4,4-trifluorobut-2-enoate (52.2 mg, 0.2 mmol) and (2*S*,3*R*)-HyperBTM (12.3 mg, 0.04 mmol) in THF at 0 °C for 72 h. Benzyl amine (109 µL, 1.0 mmol) was added and the reaction stirred at room temperature for 16 h. The crude product (66:34 dr) was purified by flash silica chromatography (67:33 Petrol : EtOAc, R_f 0.42$_{major}$ and 0.61$_{minor}$) to give:

30$_{major}$ (57.2 mg, 51 %) as a white solid. mp 157–158 °C; $[\alpha]_D^{20}$ = +15.3 (*c* 0.7, $CHCl_3$); Chiral HPLC analysis, Chiralpak AD-H (85:15 hexane : IPA, flow rate 1 mLmin^{-1}, 254 nm, 30 °C) t_R (major): 10.3 min, t_R (minor): 24.3 min, 97:3 er; ν_{max} (film) 3310 (N-H), 2924 (C-H), 1701 (C=O, amide), 1684 (C=N), 1653 (C=O, lactam), 1165, 1138, 1124 (C-F); **^{1}H NMR (400 MHz, $CDCl_3$)** δ_H: 2.50 (2H, d, *J* 6.5, 2H, C*H*$_2$CO), 3.52 (1H, d, *J* 13.1, C(4)C*H*^{A}H^{B}Ph), 3.69 (1H, d, *J* 13.1, C(4)CH^A*H*^{B}Ph), 4.08–4.18 (1H, m, CF_3C*H*), 4.32 (1H, dd, *J* 14.6, 5.6, CONHC*H*^{A}H^{B}Ph), 4.45(1H, dd, *J* 14.6, 5.6, CONHCH^A*H*^{B}Ph), 5.81 (1H, t, *J* 6.6, N*H*), 7.00–7.12 (5H, m, C*H*-Ar), 7.17–7.23 (3H, m, C*H*-Ar), 7.28–7.36 (6H, m, C*H*-Ar), 7.54–7.57 (4H, m, C*H*-Ar), 7.95–7.98 (2H, m, C*H*-Ar); **^{19}F{^{1}H} NMR (376 MHz, $CDCl_3$)** δ_F: –63.9 (s, C*F*$_3$); **^{13}C{^{1}H} NMR (101 MHz, $CDCl_3$)** δ_C: 32.9 (*C*H$_2$CO), 40.0 (C(4)*C*H$_2$Ph), 44.0 (q, *J* 26.7, CF_3*C*H), 44.1 (CONH*C*H$_2$Ph), 59.8 (*C*(4)), 119.9 (2×*C*H-Ar), 125.2 (*C*F$_3$), 125.9 (*C*H-Ar), 127.1 (2×*C*H-Ar), 127.6 (*C*H-Ar), 127.7 (*C*H-Ar), 127.9 (2×*C*H-Ar), 128.1 (2×*C*H-Ar), 128.7 (2×*C*H-Ar), 128.8 (2×*C*H-Ar), 129.0 (2×*C*H-Ar), 129.7 (2×*C*H-Ar), 130.7 (*C*H-Ar), 131.6 (*C*-Ar), 132.8 (*C*-Ar), 137.0 (*C*-Ar), 137.6 (*C*-Ar), 157.1 (*C*(3)), 168.2 (NH*C*=O), 172.9 (*C*(5)=O); **HRMS (NSI$^+$)** $C_{33}H_{29}F_3N_3O_2$ [M+H]$^+$ found 556.2199, requires 556.2206 (–1.3 ppm).

30$_{minor}$ (26.0 mg, 23 %) as a colourless oil. $[\alpha]_D^{20}$ = +36.8 (*c* 1.1, $CHCl_3$); Chiral HPLC analysis, Chiralcel OD-H (97:3 hexane : IPA, flow rate 1 mLmin^{-1}, 254 nm, 30 °C) t_R (major): 16.2 min, t_R (minor): 26.1 min, 97:3 er; ν_{max} (film) 3310 (N-H), 3032, 2934 (C-H), 1701 (C=O, amide), 1684 (C=N), 1653 (C=O, lactam), 1165, 1117 (C-F); **^{1}H NMR (400 MHz, $CDCl_3$)** δ_H: 2.80 (1H, dd, *J* 17.2, 4.0, C*H*^{A}H^{B}CO), 3.40 (1H, d, *J* 13.0, C(4)C*H*^{A}H^{B}Ph), 3.59 (1H, d, *J* 13.0, C(4)CH^A*H*^{B}Ph), 3.84 (1H, dd, *J* 17.2, 6.5, CH^A*H*^{B}CO), 4.38–4.47 (1H, m, CF_3C*H*), 4.55 (2H, d, *J* 5.7, CONHC*H*$_2$Ph), 6.33 (1H, t, *J* 6.6, N*H*), 6.82–6.84 (2H, m, C*H*-Ar), 6.98–7.02 (2H, m, C*H*-Ar), 7.08–7.12 (1H, m, C*H*-Ar), 7.21–7.25 (1H, m, C*H*-Ar), 7.29–7.39 (7H, m, C*H*-Ar), 7.51–7.57 (5H, m, C*H*-Ar), 8.04–8.06 (2H, m, C*H*-Ar); **^{19}F{^{1}H} NMR (376 MHz, $CDCl_3$)** δ_F: –66.2 (s, C*F*$_3$); **^{13}C{^{1}H} NMR (101 MHz, $CDCl_3$)** δ_C: 29.9 (*C*H$_2$CO), 40.4 (C(4)*C*H$_2$Ph), 44.1 (CONH*C*H$_2$Ph), 44.4 (q, $^2J_{CF}$ 25.9, CF_3*C*H), 56.6 (*C*(4)), 120.8 (2×*C*H-Ar), 126.2 (*C*H-Ar), 126.4 (q, $^1J_{CF}$ 281.7, *C*F$_3$), 126.5 (2×*C*H-Ar), 127.6 (*C*H-Ar), 127.7 (3×*C*H-Ar), 128.0 (2×*C*H-Ar), 128.80 (2×*C*H-Ar), 128.84 (2×*C*H-Ar), 129.1 (2×*C*H-Ar), 129.6 (2×*C*H-Ar), 130.5 (*C*H-Ar), 131.1 (*C*-Ar), 132.6 (*C*-Ar), 137.0 (*C*-Ar), 137.7 (*C*-Ar), 157.9 (*C*(3)), 170.1 (NH*C*=O), 173.4 (*C*(5)=O); **HRMS (NSI$^+$)** $C_{33}H_{29}F_3N_3O_2$ [M+H]$^+$ found 556.2202, requires 556.2206 (–0.8 ppm).

(*S*)-*N*-benzyl-4,4,4-trifluoro-3-((*S*)-3-methyl-4-(2-methylbenzyl)-5-oxo-1-phenyl-4,5-dihydro-1*H*-pyrazol-4-yl)butanamide (31_{major}) and (*S*)-*N*-benzyl-4,4,4-trifluoro-3-((*R*)-3-methyl-4-(2-methylbenzyl)-5-oxo-1-phenyl-4,5-dihydro-1*H*-pyrazol-4-yl)butanamide (31_{minor})

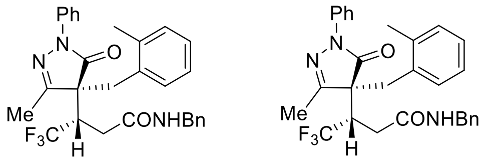

major minor

Following General Procedure **7**, pyrazol-one (55.6 mg, 0.2 mmol), 4-nitrophenyl (*E*)-4,4,4-trifluorobut-2-enoate (52.2 mg, 0.2 mmol) and (2*S*,3*R*)-HyperBTM (12.3 mg, 0.04 mmol) in THF at 0 °C for 72 h. Benzyl amine (109 µL, 1.0 mmol) was added and the reaction stirred at room temperature for 16 h. The crude product (58:42 dr) was purified by flash silica chromatography (67:33 Petrol : EtOAc, R_f 0.43_{major} and 0.64_{minor}) to give:

31_{major} (40.6 mg, 40 %) as a a pale-yellow solid. mp 143–144 °C;$[\alpha]_D^{20}$ = –59.2 (*c* 0.5, $CHCl_3$); Chiral HPLC analysis, Chiralcel AD-H (90:10 hexane : IPA, flow rate 1 $mLmin^{-1}$, 254 nm, 30 °C) t_R (major): 11.2 min, t_R (minor): 22.8 min, 99:1 er; ν_{max} (film) 3319 (N-H), 2957, 2928 (C-H), 1699 (C=N), 1684 (C=O, amide), 1653 (C=O, lactam), 1167, 1134, 1121 (C-F); **^{1}H NMR (400 MHz, $CDCl_3$)** δ_H: 2.33 (3H, s, $C_6H_4CH_3$), 2.40 (3H, s, C(3)CH_3), 2.63–2.65 (2H, m, CH_2CO), 3.03 (1H, d, *J* 13.6, C(4)CH^AH^BAr), 3.47 (1H, d, *J* 13.6, C(4)CH^AH^BAr), 3.85–3.95 (1H, m, CF_3CH), 4.45–4.57 (2H, m, CONHCH_2Ph), 5.93 (1H, t, *J* 5.6, N*H*), 6.90–6.93 (1H, m, C*H*-Ar), 6.96–7.00 (1H, m, C*H*-Ar), 7.03–7.07 (1H, m, C*H*-Ar), 7.09–7.16 (2H, m, C*H*-Ar), 7.26–7.36 (7H, m, C*H*-Ar), 7.37–7.40 (2H, m, C*H*-Ar); **^{19}F{^{1}H} NMR (376 MHz, $CDCl_3$)** δ_F: –65.5 (s, CF_3); **^{13}C{^{1}H} NMR (101 MHz, $CDCl_3$)** δ_C: 16.0 (C(3)CH_3), 20.3 ($C_6H_4CH_3$), 32.0 (CH_2CO), 34.2 (C(4)CH_2Ph), 44.20 (q, $^2J_{CF}$ 26.8, CF_3CH), 44.24 (CONHCH_2Ph), 58.9 (*C*(4)), 119.9 (2×*C*H-Ar), 125.6 (2×*C*H-Ar), 126.5 (q, $^1J_{CF}$ 282.2, CF_3), 127.6 (*C*H-Ar), 127.8 (*C*H-Ar), 127.9 (2×*C*H-Ar), 128.9 (2×*C*H-Ar), 128.8 (*C*H-Ar), 128.9 (2×*C*H-Ar), 130.9 (*C*H-Ar), 131.5 (*C*-Ar), 137.1 (*C*-Ar), 137.3 (*C*-Ar), 137.6 (*C*-Ar), 158.5 (*C*(3)), 168.5 (NH*C*=O), 172.6 (*C*(5)=O); **HRMS (NSI$^+$)** $C_{29}H_{29}F_3N_3O_2$ $[M+H]^+$ found 508.2201, requires 508.2206 (–1.1 ppm).

31_{minor} (29.4 mg, 29 %) as a pale-yellow oil. $[\alpha]_D^{20}$ = +125.0 (*c* 1.4, $CHCl_3$); Chiral HPLC analysis, Chiralcel OD-H (95:5 hexane : IPA, flow rate 1 $mLmin^{-1}$, 254 nm, 30 °C) t_R (major): 11.8 min, t_R (minor): 20.8 min, 96:4 er; ν_{max} (film) 3310 (N-H), 2965, 2963 (C-H), 1699 (C=N), 1684 (C=O, amide), 1653 (C=O, lactam), 1263, 1165, 1117 (C-F); **^{1}H NMR (400 MHz, $CDCl_3$)** δ_H: 2.17 (3H, s, $C_6H_4CH_3$), 2.32 (3H, s, C(3)CH_3), 2.78 (1H, dd, *J* 17.2, 5.5, CH_AH_BCO), 3.13 (1H, d, *J* 14.1, C(4)CH^AH^BAr), 3.34 (1H, d, *J* 14.1, C(4)CH^AH^BAr), 3.55 (1H, dd, *J* 17.2, 4.9, CH_AH_BCO), 3.80–3.89 (1H, m, CF_3CH), 4.35 (1H, dd, *J* 14.8, 5.8, CONHCH^AH^BPh), 4.50 (1H, dd, *J* 14.8, 5.8, CONHCH^AH^BPh), 6.51 (1H, t, *J* 5.8, N*H*), 6.91–6.93 (1H, m, C*H*-Ar), 6.96–7.00 (1H, m, C*H*-Ar), 7.09–7.11 (2H, m, C*H*-Ar), 7.18–7.36 (8H, m, C*H*-Ar), 7.46–7.49 (2H, m, C*H*-Ar); **^{19}F{^{1}H} NMR (376 MHz, $CDCl_3$)** δ_F: –66.5 (s, CF_3); **^{13}C{^{1}H} NMR (101 MHz, $CDCl_3$)** δ_C: 14.9 (C(3)CH_3), 20.2 ($C_6H_4CH_3$), 30.0 (CH_2CO), 34.8 (C(4)CH_2Ph), 44.3 (q, $^2J_{CF}$ 26.8, CF_3CH), 44.0 (CONHCH_2Ph), 56.9 (*C*(4)), 120.5 (2×*C*H-Ar), 125.99 (*C*H-Ar), 126.0 (*C*H-Ar), 126.3 (q, $^1J_{CF}$ 282.0, CF_3), 127.6 (*C*H-Ar), 127.7 (3×*C*H-Ar), 128.6 (*C*H-Ar), 128.7 (2×*C*H-Ar), 128.9 (2×*C*H-Ar), 130.9 (*C*H-Ar), 131.8 (*C*-Ar), 136.5 (*C*-Ar), 137.1 (*C*-Ar), 137.9 (*C*-Ar), 160.8 (*C*(3)), 169.8 (NH*C*=O), 173.3 (*C*(5)=O); **HRMS (NSI$^+$)** $C_{29}H_{29}F_3N_3O_2$ $[M+H]^+$ found 508.2201, requires 508.2206 (–1.1 ppm).

(*S*)-*N*-benzyl-4,4,4-trifluoro-3-((*S*)-3-methyl-4-(3-methylbenzyl)-5-oxo-1-phenyl-4,5-dihydro-1*H*-pyrazol-4-yl)butanamide (32_{major}) and (*S*)-*N*-benzyl-4,4,4-trifluoro-3-((*R*)-3-methyl-4-(3-methylbenzyl)-5-oxo-1-phenyl-4,5-dihydro-1*H*-pyrazol-4-yl)butanamide (32_{minor})

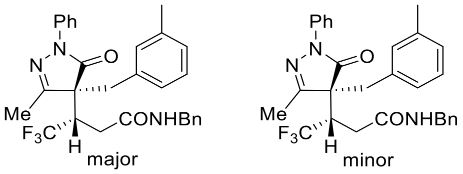

Following General Procedure **7**, pyrazol-one (55.6 mg, 0.2 mmol), 4-nitrophenyl (*E*)-4,4,4-trifluorobut-2-enoate (52.2 mg, 0.2 mmol) and (2*S*,3*R*)-HyperBTM (12.3 mg, 0.04 mmol) in THF at 0 °C for 48 h. Benzyl amine (109 µL, 1.0 mmol) was added and the reaction stirred at room temperature for 16 h. The crude product (67:33 dr) was purified by flash silica chromatography (67:33 Petrol : EtOAc, R_f 0.43_{major} and 0.67_{minor}) to give:

$\mathbf{32_{major}}$ (58.0 mg, 57 %) as a pale-yellow solid. mp 127–128 °C;$[\alpha]_D^{20}$ = –86.8 (*c* 1.0, $CHCl_3$); Chiral HPLC analysis, Chiralpak AD-H (90:10 hexane : IPA, flow rate 1 mLmin^{-1}, 254 nm, 30 °C) t_R (major): 15.8 min, t_R (minor): 21.4 min, 95:5 e5; ν_{max} (film) 3310 (N-H), 2957, 2926 (C-H), 1699 (C=O, amide), 1684 (C=N), 1647 (C=O, lactam), 1167, 1134, 1121 (C-F); **^{1}H NMR (400 MHz, CDCl$_3$)** δ_H: 2.14 (3H, s, $C_6H_4CH_3$), 2.26 (3H, s, C(3)C*H*$_3$), 2.62 (1H, dd, *J* 16.2, 4.8, CH^AH^BCO), 2.70 (1H, dd, *J* 16.2, 6.6, CH^AH^BCO), 3.04 (1H, d, *J* 13.0, C(4)CH^AH^BAr), 3.25 (1H, d, *J* 13.0, C(4)CH^AH^BAr), 3.83–3.92 (1H, m, CF_3CH), 4.41–4.52 (2H, m, CONHC*H*$_2$Ph), 6.52 (1H, t, *J* 5.7, N*H*), 6.85–6.88 (2H, m, C*H*-Ar), 6.95–6.97 (1H, m, C*H*-Ar), 7.02–7.06 (1H, m, C*H*-Ar), 7.13–7.17 (1H, m, C*H*-Ar), 7.25–7.33 (7H, m, C*H*-Ar), 7.43–7.46 (2H, m, C*H*-Ar); **^{19}F{^{1}H} NMR (376 MHz, CDCl$_3$)** δ_F: –65.6 (s, C*F*$_3$); **^{13}C{^{1}H} NMR (101 MHz, CDCl$_3$)** δ_C: 15.8 (C(3)*C*H$_3$), 21.2 (Ar*C*H$_3$), 31.8 (*C*H$_2$CO), 38.9 (C(4)*C*H$_2$Ph), 43.6 (q, $^2J_{CF}$ 26.3, CF$_3$*C*H), 44.1 (CONH*C*H$_2$Ph), 59.5 (*C*(4)), 119.8 (2×*C*H-Ar), 125.6 (N(1)Ph*C*(4)), 126.4 (C(4)CH$_2$Ar*C*(2)), 126.6 (q, $^1J_{CF}$ 281.7, *C*F$_3$), 127.7 (C(4)CH$_2$Ar*C*(5)), 127.8 (2×*C*H-Ar), 128.1 (*C*H-Ar), 128.5 (*C*H-Ar), 128.7 (2×*C*H-Ar), 128.8 (2×*C*H-Ar), 130.1 (*C*H-Ar), 132.6 (*C*-Ar), 137.0 (*C*-Ar), 137.7 (*C*-Ar), 137.9 (*C*-Ar), 158.8 (*C*(3)), 168.7 (NH*C*=O), 172.9 (*C*(5)=O); **HRMS (NSI$^+$)** $C_{29}H_{29}F_3N_3O_2$ $[M+H]^+$ found 508.2199, requires 508.2206 (–1.5 ppm).

$\mathbf{32_{minor}}$ (31.7 mg, 31 %) as a pale-yellow solid. mp 153–154 °C;$[\alpha]_D^{20}$ = +142.4 (*c* 1.5, $CHCl_3$); Chiral HPLC analysis, Chiralpak AD-H (93:7 hexane : IPA, flow rate 1 mLmin^{-1}, 254 nm, 30 °C) t_R (minor): 22.3 min, t_R (major): 26.1 min, 96:4 er; ν_{max} (film) 3310 (N-H), 2926 (C-H), 1701 (C=O, amide), 1684 (C=N), 1645 (C=O, lactam), 1265, 1165, 1117 (C-F); **^{1}H NMR (400 MHz, CDCl$_3$)** δ_H: 2.15 (3H, s, $C_6H_4CH_3$), 2.19 (3H, s, C(3)C*H*$_3$), 2.73 (1H, dd, *J* 17.1, 5.6, CH^AH^BCO), 2.96 (1H, d, *J* 13.1, C(4)CH^AH^BAr), 3.30 (1H, d, *J* 13.1, C(4)CH^AH^BAr), 3.55 (1H, dd, *J* 17.1, 5.6, CH^AH^BCO), 3.74–3.83 (1H, m, CF_3CH), 4.45–4.55 (2H, m, CONHC*H*$_2$Ph), 6.13 (1H, t, *J* 5.7, N*H*), 6.85–6.87 (2H, m, C*H*-Ar), 6.99–7.00 (1H, m, C*H*-Ar), 7.05–7.08 (1H, m, C*H*-Ar), 7.17–7.21 (1H, m, C*H*-Ar), 7.27–7.37 (7H, m, C*H*-Ar), 7.51–7.53 (2H, m, C*H*-Ar); **^{19}F{^{1}H} NMR (376 MHz, CDCl$_3$)** δ_F: –66.6 (s, C*F*$_3$); **^{13}C{^{1}H} NMR (101 MHz, CDCl$_3$)** δ_C: 14.9 (C(3)*C*H$_3$), 21.2 (Ar*C*H$_3$), 29.8 (*C*H$_2$CO), 39.5 (C(4)*C*H$_2$Ph), 43.9 (q, $^2J_{CF}$ 26.1, CF$_3$*C*H), 44.2 (CONH*C*H$_2$Ph), 57.1 (*C*(4)), 120.1 (2×*C*H-Ar), 125.7 (*C*H-Ar), 126.2 (*C*H-Ar), 126.3 (q, $^1J_{CF}$ 281.1, *C*F$_3$), 127.7 (*C*H-Ar), 127.8 (2×*C*H-Ar), 128.3 (*C*H-Ar), 128.5 (*C*H-Ar), 128.7 (2×*C*H-Ar), 128.8 (2×*C*H-Ar), 129.8 (*C*H-Ar), 132.7 (*C*-Ar), 137.1 (*C*-Ar), 137.6 (*C*-Ar), 138.1 (*C*-Ar), 160.5 (*C*(3)), 169.6 (NH*C*=O), 172.9 (*C*(5)=O); **HRMS (NSI$^+$)** $C_{29}H_{29}F_3N_3O_2$ $[M+H]^+$ found 508.2201, requires 508.2206 (–1.1 ppm).

(*S*)-*N*-benzyl-4,4,4-trifluoro-3-((*S*)-4-(4-chlorobenzyl)-3-methyl-5-oxo-1-phenyl-4,5-dihydro-1*H*-pyrazol-4-yl)butanamide (33_{major}) and (*S*)-*N*-benzyl-4,4,4-trifluoro-3-((*R*)-4-(4-chlorobenzyl)-3-methyl-5-oxo-1-phenyl-4,5-dihydro-1*H*-pyrazol-4-yl)butanamide (33_{minor})

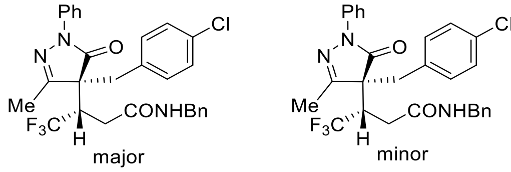

Following General Procedure **7**, 4-(4-chlorobenzyl)-5-methyl-2-phenyl-2,4-dihydro-3*H*-pyrazol-3-one (59.8 mg, 0.2 mmol), 4-nitrophenyl (*E*)-4,4,4-trifluorobut-2-enoate (52.2 mg, 0.2 mmol) and (2*S*,3*R*)-HyperBTM (12.3 mg, 0.04 mmol) in THF at 0 °C for 24 h. Benzyl amine (109 µL, 1.0 mmol) was added and the reaction stirred at room temperature for 16 h. The crude product (66:34 dr) was purified by flash silica chromatography (67:33 Petrol : EtOAc, R_f 0.32_{major} and 0.60_{minor}) to give:

33_{major} (56.7 mg, 54 %) as a white solid. mp 119–120 °C;$[\alpha]_D^{20}$ = –99.3 (*c* 1.0, $CHCl_3$); Chiral HPLC analysis, Chiralcel OD-H (90:10 hexane : IPA, flow rate 1 $mLmin^{-1}$, 254 nm, 30 °C) t_R (minor): 19.0 min, t_R (major): 34.1 min, 94:6 er; ν_{max} (film) 3310 (N-H), 2959, 2928 (C-H), 1697 (C=O, amide), 1684 (C=N), 1647 (C=O, lactam), 1167, 1135, 1123 (C-F); **^{1}H NMR (400 MHz, $CDCl_3$)** δ_H: 2.27 (3H, s, C(3)CH_3), 2.58 (1H, dd, *J* 16.2, 4.8, CH^AH^BCO), 2.68 (1H, dd, *J* 16.2, 6.5, CH^AH^BCO), 3.03 (1H, d, *J* 13.1, C(4)CH^AH^BAr), 3.25 (1H, d, *J* 13.1, C(4)CH^AH^BAr), 3.80–3.90 (1H, m, CF_3C*H*), 4.39–4.55 (2H, m, CONHCH_2Ph), 6.30 (1H, t, *J* 5.7, N*H*), 6.97–7.01 (2H, m, C*H*-Ar), 7.11–7.14 (2H, m, C*H*-Ar), 7.15–7.19 (1H, m, C*H*-Ar), 7.25–7.35 (7H, m, C*H*-Ar), 7.42–7.45 (2H, m, C*H*-Ar); **^{19}F{^{1}H} NMR (376 MHz, $CDCl_3$)** δ_F: –65.5 (s, CF_3); **^{13}C{^{1}H} NMR (101 MHz, $CDCl_3$)** δ_C: 15.8 (C(3)CH_3), 31.8 (CH_2CO), 38.0 (C(4)CH_2Ph), 43.6 (q, $^2J_{CF}$ 26.4, CF_3CH), 44.2 (CONHCH_2Ph), 59.4 (*C*(4)), 119.7 (2×*C*H-Ar), 125.9 (*C*H-Ar), 126.5 (q, $^1J_{CF}$ 281.7, *C*F_3), 127.81 (*C*H-Ar), 127.84 (2×*C*H-Ar), 128.5 (2×*C*H-Ar), 128.8 (2×*C*H-Ar), 128.9 (2×*C*H-Ar), 130.8 (2×*C*H-Ar), 131.2 (*C*-Ar), 133.8 (*C*-Ar), 136.8 (*C*-Ar), 137.6 (*C*-Ar), 158.4 (*C*(3)), 168.4 (NH*C*=O), 172.5 (*C*(5)=O); **HRMS (NSI$^+$)** $C_{28}H_{26}ClF_3N_3O_2$ $[M+H]^+$ found 528.1654, requires 528.1660 (–1.2 ppm).

33_{minor} (36.5 mg, 35 %) as a white solid. mp 90–92 °C;$[\alpha]_D^{20}$ = +50.8 (*c* 0.5, $CHCl_3$); Chiral HPLC analysis, Chiralcel OD-H (97:3 hexane : IPA, flow rate 1 $mLmin^{-1}$, 254 nm, 30 °C) t_R (major): 23.6 min, t_R (minor): 47.2 min, 97:3 er; ν_{max} (film) 3308 (N-H), 2959, 2926 (C-H), 1699 (C=O, amide), 1684 (C=N), 1645 (C=O, lactam), 1265, 1167, 1119 (C-F); **^{1}H NMR (400 MHz, $CDCl_3$)** δ_H: 2.21 (3H, s, C(3)CH_3), 2.72 (1H, dd, *J* 17.1, 4.7, CH^AH^BCO), 2.98 (1H, d, *J* 13.3, C(4)CH^AH^BAr), 3.25 (1H, d, *J* 13.3, C(4)CH^AH^BAr), 3.54 (1H, dd, *J* 17.1, 5.8, CH^AH^BCO), 3.73–3.83 (1H, m, CF_3C*H*), 4.49 (2H, d, *J* 5.7, CONHCH_2Ph), 6.22 (1H, t, *J* 5.6, N*H*), 6.96–6.99 (2H, m, C*H*-Ar), 7.13–7.16 (2H, m, C*H*-Ar), 7.19–7.23 (1H, m, C*H*-Ar), 7.25–7.39 (7H, m, C*H*-Ar), 7.48–7.51 (2H, m, C*H*-Ar); **^{19}F{^{1}H} NMR (376 MHz, $CDCl_3$)** δ_F: –66.6 (s, CF_3); **^{13}C{^{1}H} NMR (101 MHz, $CDCl_3$)** δ_C: 14.8 (C(3)CH_3), 29.7 (CH_2CO), 38.6 (C(4)CH_2Ph), 43.8 (q, $^2J_{CF}$ 26.3, CF_3CH), 44.1 (CONHCH_2Ph), 57.1 (*C*(4)), 120.1 (2×*C*H-Ar), 126.0 (*C*H-Ar), 126.2 (q, $^1J_{CF}$ 281.4, *C*F_3), 127.8 (3×*C*H-Ar), 128.6 (2×*C*H-Ar), 128.8 (2×*C*H-Ar), 128.9 (2×*C*H-Ar), 130.5 (2×*C*H-Ar), 131.4 (*C*-Ar), 133.8 (*C*-Ar), 136.9 (*C*-Ar), 137.6, (*C*-Ar) 160.2 (*C*(3)), 169.5 (NH*C*=O), 172.6 (*C*(5)=O); **HRMS (NSI$^+$)** $C_{28}H_{26}ClF_3N_3O_2$ $[M+H]^+$ found 528.1654, requires 528.1660 (–1.2 ppm).

(*S*)-*N*-benzyl-3-((*S*)-4-(4-cyanobenzyl)-3-methyl-5-oxo-1-phenyl-4,5-dihydro-1*H*-pyrazol-4-yl)-4,4,4-trifluorobutanamide (34_{major}) and (*S*)-*N*-benzyl-3-((*R*)-4-(4-cyanobenzyl)-3-methyl-5-oxo-1-phenyl-4,5-dihydro-1*H*-pyrazol-4-yl)-4,4,4-trifluorobutanamide (34_{minor})

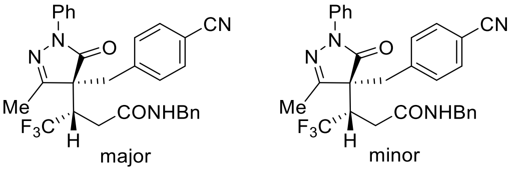

Following General Procedure **7**, 4-((3-methyl-5-oxo-1-phenyl-4,5-dihydro-1*H*-pyrazol-4-yl)methyl)benzonitrile (57.9 mg, 0.2 mmol), 4-nitrophenyl (*E*)-4,4,4-trifluorobut-2-enoate (52.2 mg, 0.2 mmol) and (2*S*,3*R*)-HyperBTM (12.3 mg, 0.04 mmol) in THF at 0 °C for 24 h. Benzyl amine (109 µL, 1.0 mmol) was added and the reaction stirred at room temperature for 16 h. The crude product (61:39 dr) was purified by flash silica chromatography (67:33 Petrol : EtOAc, R_f 0.13_{major} and 0.22_{minor}) to give:

34_{major} (40.1 mg, 39 %) as a pale-yellow oil. $[\alpha]_D^{20}$ = –99.8 (*c* 0.9, $CHCl_3$); Chiral HPLC analysis, Chiralcel OD-H (90:10 hexane : IPA, flow rate 1 mLmin^{-1}, 254 nm, 30 °C) t_R (minor): 41.3 min, t_R (major): 91.5 min, 92:8 er; ν_{max} (film) 3335 (N-H), 2967, 2934 (C-H), 1699 (C=O, amide), 1684 (C=N), 1647 (C=O, lactam), 1167, 1134, 1121 (C-F); **^{1}H NMR (400 MHz, $CDCl_3$)** δ_H: 2.21 (3H, s, C(3)CH_3), 2.46 (1H, dd, *J* 16.2, 4.7, CH^AH^BCO), 2.59 (1H, dd, *J* 16.2, 6.5, CH^AH^BCO), 3.00 (1H, d, *J* 13.0, C(4)CH^AH^BAr), 3.25 (1H, d, *J* 13.0, C(4)CH^AH^BAr), 3.71–3.81 (1H, m, CF_3C*H*), 4.33–4.44 (2H, m, CONHCH_2Ph), 6.01 (1H, t, *J* 5.7, N*H*), 7.06–7.10 (3H, m, C*H*-Ar), 7.17–7.26 (7H, m, C*H*-Ar), 7.31–7.37 (4H, m, C*H*-Ar); **^{19}F{^{1}H} NMR (376 MHz, $CDCl_3$)** δ_F: –65.4 (s, CF_3); **^{13}C{^{1}H} NMR (101 MHz, $CDCl_3$)** δ_C: 15.9 (C(3)*C*H_3), 31.9 (*C*H_2CO), 38.6 (C(4)*C*H_2Ph), 43.5 (q, $^2J_{CF}$ 26.8, CF_3*C*H), 44.3 (CONH*C*H_2Ph), 59.3 (*C*(4)), 111.8 (*C*N), 118.4 (*C*-Ar), 119.4 (2×*C*H-Ar), 126.4 (q, $^1J_{CF}$ 281.9, *C*F_3), 126.0 (N(1)Ph*C*(4)), 127.9 (3×*C*H-Ar), 128.9 (4×*C*H-Ar), 130.3 (2×*C*H-Ar), 132.0 (2×*C*H-Ar), 136.7 (*C*-Ar), 137.4 (*C*-Ar), 138.4 (*C*-Ar), 158.0 (*C*(3)), 168.2 (NH*C*=O), 171.9 (*C*(5)=O); **HRMS (NSI$^+$)** $C_{29}H_{26}F_3N_4O_2$ $[M+H]^+$ found 519.1996, requires 519.2002 (–1.2 ppm).

34_{minor} (22.0 mg, 21 %) as a pale-yellow oil. $[\alpha]_D^{20}$ = +171.9 (*c* 1.1, $CHCl_3$); Chiral HPLC analysis, Chiralcel OD-H (90:10 hexane : IPA, flow rate 1 mLmin^{-1}, 254 nm, 30 °C) t_R (major): 14.1 min, t_R (minor): 20.4 min, 96:4 er; ν_{max} (film) 3327 (N-H), 2967, 2934 (C-H), 1699 (C=O, amide), 1684 (C=N), 1653 (C=O, lactam), 1263, 1165, 1117 (C-F); **^{1}H NMR (400 MHz, $CDCl_3$)** δ_H: 2.12 (3H, s, C(3)CH_3), 2.64 (1H, dd, *J* 17.1, 4.6, CH^AH^BCO), 2.97 (1H, d, *J* 13.2, C(4)CH^AH^BPh), 3.20 (1H, d, *J* 13.2, C(4)CH^AH^BPh), 3.44 (1H, dd, *J* 17.1, 5.8, CH^AH^BCO), 3.67–3.75 (1H, m, CF_3C*H*), 4.35 (1H, dd, *J* 14.7, 5.8, CONHCH^AH^BPh), 4.42 (1H, dd, *J* 14.7, 5.8, CONHCH^AH^BPh), 6.26 (1H, t, *J* 5.8, N*H*), 7.04–7.07 (2H, m, C*H*-Ar), 7.11–7.23 (6H, m, C*H*-Ar), 7.24–7.29 (2H, m, C*H*-Ar), 7.35–7.37 (4H, m, C*H*-Ar); **^{19}F{^{1}H} NMR (376 MHz, $CDCl_3$)** δ_F: –66.5 (s, CF_3); **^{13}C{^{1}H} NMR (101 MHz, $CDCl_3$)** δ_C: 14.9 (C(3)*C*H_3), 29.7 (*C*H_2CO), 39.1 (C(4)*C*H_2Ph), 43.8 (q, $^2J_{CF}$ 26.3, CF_3*C*H), 44.2 (CONH*C*H_2Ph), 57.0 (*C*(4)), 111.9 (*C*N), 118.4 (*C*-Ar), 119.9 (2×*C*H-Ar), 126.1 (q, $^1J_{CF}$ 281.6, *C*F_3), 126.2 (*C*H-Ar), 127.77 (2×*C*H-Ar), 127.80 (*C*H-Ar), 128.8 (2×*C*H-Ar), 129.0 (2×*C*H-Ar), 130.1 (2×*C*H-Ar), 132.2 (2×*C*H-Ar), 136.7 (*C*-Ar), 137.6 (*C*-Ar), 138.5 (*C*-Ar), 159.8 (*C*(3)), 169.4 (NH*C*=O), 172.2 (*C*(5)=O); **HRMS (NSI$^+$)** $C_{29}H_{26}F_3N_4O_2$ $[M+H]^+$ found 519.1996, requires 519.2002 (–1.2 ppm).

(*S*)-*N*-benzyl-4,4,4-trifluoro-3-((*R*)-3-methyl-5-oxo-1,4-diphenyl-4,5-dihydro-1*H*-pyrazol-4-yl)butanamide (35$_{major}$) and (*S*)-*N*-benzyl-4,4,4-trifluoro-3-((*S*)-3-methyl-5-oxo-1,4-diphenyl-4,5-dihydro-1*H*-pyrazol-4-yl)butanamide (35$_{minor}$)

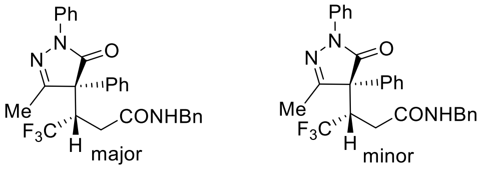

Following General Procedure **7**, pyrazol-one (50.0 mg, 0.2 mmol), 4-nitrophenyl (*E*)-4,4,4-trifluorobut-2-enoate (52.2 mg, 0.2 mmol) and (*R*)-BTM (10.7 mg, 0.04 mmol) in THF at 0 °C for 48 h. Benzyl amine (109 µL, 1.0 mmol) was added and the reaction stirred at room temperature for 16 h. The crude product (88:12 dr) was purified by flash silica chromatography (67:33 Petrol : EtOAc, R_f 0.28$_{major}$ and 0.39$_{minor}$) to give:

35$_{major}$ (59.8 mg, 54 %) as a white solid. mp 161–163 °C; $[\alpha]_D^{20}$ = +157.4 (*c* 1.0, $CHCl_3$); Chiral HPLC analysis, Chiralcel OD-H (90:10 hexane : IPA, flow rate 1 mLmin^{-1}, 254 nm, 30 °C) t_R (major): 14.9 min, t_R (minor): 67.9 min, 95:5 er; ν_{max} (film) 3327 (N-H), 2957, 2930 (C-H), 1699 (C=O, amide), 1684 (C=N), 1645 (C=O, lactam), 1161, 1132, 1119 (C-F); **^{1}H NMR (400 MHz, $CDCl_3$)** δ_H: 2.31 (3H, s, C(3)CH_3), 2.52 (1H, br, CH^AH^{B}CO), 2.54 (1H, br, CH^AH^BCO), 4.41 (1H, dd, *J* 14.6, 5.6, CONHCH^AH^{B}Ph), 4.52 (1H, dd, *J* 14.6, 5.6, CONHCH^AH^BPh), 4.55–4.62 (1H, m, CF_3C*H*), 5.74 (1H, t, *J* 5.7, N*H*), 7.17–7.25 (3H, m, C*H*-Ar), 7.30–7.46 (10H, m, C*H*-Ar), 7.84–7.86 (2H, m, C*H*-Ar); **^{19}F{^{1}H} NMR (376 MHz, $CDCl_3$)** δ_F: –66.6 (s, CF_3); **^{13}C{^{1}H} NMR (101 MHz, $CDCl_3$)** δ_C: 17.0 (C(3)*C*H_3), 32.3 (*C*H_2CO), 43.4 (q, $^2J_{CF}$ 26.0, CF_3*C*H), 44.1 (CONH*C*H_2Ph), 61.6 (*C*(4)), 119.0 (2×*C*H-Ar), 126.7 (q, $^1J_{CF}$ 282.3, *C*F_3), 125.4 (*C*H-Ar), 126.8 (2×*C*H-Ar), 127.8 (*C*H-Ar), 127.9 (2×*C*H-Ar), 128.8 (2×*C*H-Ar), 128.87 (2×*C*H-Ar), 128.93 (*C*H-Ar), 130.0 (2×*C*H-Ar), 133.3 (*C*-Ar), 137.6(*C*-Ar), 137.8 (*C*-Ar), 158.9(*C*(3)), 168.4(NH*C*=O), 171.8 (*C*(5)=O); **HRMS (NSI$^+$)** $C_{27}H_{25}F_3N_3O_2$ [M+H]$^+$ found 480.1881, requires 480.1893 (–2.6 ppm).

35$_{minor}$ (12.6 mg, 11 %) as a white solid. mp 182–184 °C; $[\alpha]_D^{20}$ = +112.3 (*c* 0.5, $CHCl_3$); Chiral HPLC analysis, Chiralpak AD-H (95:5 hexane : IPA, flow rate 1 mLmin^{-1}, 254 nm, 30 °C) t_R (major): 29.0 min, t_R (minor): 51.1 min, 94:6 er; ν_{max} (film) 3292 (N-H), 2926 (C-H), 1684 (C=N), 1653 (C=O, amide), 1647 (C=O, lactam), 1271, 1130, 1121 (C-F); **^{1}H NMR (400 MHz, $CDCl_3$)** δ_H: 2.09 (3H, s, C(3)CH_3), 2.77–2.88 (2H, m, CH_2CO), 4.30–4.42 (2H, m, CONHCH^AH^{B}Ph and CF_3C*H*), 4.48 (1H, dd, *J* 14.8, 5.9, CONHCH^AH^BPh), 5.80 (1H, t, *J* 5.8, N*H*), 7.18–7.20 (2H, m, C*H*-Ar), 7.25–7.49 (11H, m, C*H*-Ar), 7.92–7.95 (2H, m, C*H*-Ar); **^{19}F{^{1}H} NMR (376 MHz, $CDCl_3$)** δ_F: –66.7 (s, CF_3); **^{13}C{^{1}H} NMR (101 MHz, $CDCl_3$)** δ_C: 14.3 (C(3)*C*H_3), 31.1 (*C*H_2CO), 42.8 (q, $^2J_{CF}$ 26.1, CF_3*C*H), 43.9 (CONH*C*H_2Ph), 61.3 (*C*(4)), 119.5 (2×*C*H-Ar), 126.6 (q, $^1J_{CF}$ 281.7, *C*F_3), 125.7 (*C*H-Ar), 127.0 (2×*C*H-Ar), 127.6 (3×*C*H-Ar), 128.7 (2×*C*H-Ar), 128.8 (3×*C*H-Ar), 129.9 (2×*C*H-Ar), 133.8 (*C*-Ar), 137.6 (*C*-Ar), 137.7 (*C*-Ar), 161.0 (*C*(3)), 169.6 (NH*C*=O), 172.9 (*C*(5)=O); **HRMS (NSI$^+$)** $C_{27}H_{25}F_3N_3O_2$ [M+H]$^+$ found 480.1886, requires 480.1893 (–1.5 ppm).

4.2 Data for Michael adducts with 3-monosubstituted oxindoles

***tert*-Butyl (*R*)-2-oxo-3-phenyl-3-((*S*)-1,1,1-trifluoro-4-(4-nitrophenoxy)-4-oxobutan-2-yl)indoline-1-carboxylate (13)**

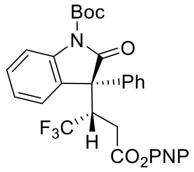

Following General Procedure **9**, *tert*-butyl 2-oxo-3-phenylindoline-1-carboxylate (61.9 mg, 0.2 mmol), 4-nitrophenyl (*E*)-4,4,4-trifluorobut-2-enoate (52.2 mg, 0.2 mmol), (2*S*,3*R*)-HyperBTM (6.2 mg, 0.02 mmol) and DIPEA (35.0 µL, 0.2 mmol) in anhydrous THF (2.0 mL) at room temperature for 24 h. The reaction mixture was concentrated and the crude residue (86:14 dr) was purified by column chromatography (95:5 Petrol : EtOAc, R_f 0.14) afforded the desired compound (70.0 mg, 61%) as white powder. mp: 136–137°C; $[\alpha]_D^{20}$ = +161.2 (*c* 0.5, $CHCl_3$); Chiral HPLC analysis, Chiralcel OD-H (95:5 hexane : IPA, flow rate 1 mL/min^{-1}, 254 nm, 30 °C) t_R (major): 9.7 min, t_R (minor): 13.6 min, 98:2 er; ν_{max} (film), 1767 (C=O), 1733 (C=O), 1724 (C=O), 1134 (C-F), 1117 (C-F), 1080 (C-F); **^{1}H NMR ($CDCl_3$, 400 MHz)** δ_H: 1.63 (9H, s, $CO_2C(CH_3)_3$), 2.90 (1H, dd, *J* 16.9, 2.3, CH^AH^BCO), 3.10 (1H, dd, *J* 16.9, 9.4, CH^AH^BCO), 4.45–4.54 (1H, m, C*H*CF_3), 7.20–7.24 (2H, m, C*H*-Ar_{PNP}), 7.33–7.41 (7H, m, C*H*-Ar), 7.50–7.55 (1H, m, C*H*-Ar), 8.07 (1H, d, *J* 8.2, C*H*-Ar), 8.27–8.31 (2H, m, C*H*-Ar_{PNP}); **$^{19}F\{^1H\}$ NMR (376 MHz, $CDCl_3$)** δ_F: –65.9 (s, C*F*$_3$); **^{13}C NMR ($CDCl_3$, 100 MHz)** δ_C: 28.0 ($CO_2C(CH_3)_3$), 31.3 (CH_2CO), 47.0 (q, $^2J_{C-F}$ 25.1, *C*HCF_3), 55.8 (*C*(3)), 84.9 ($CO_2C(CH_3)_3$), 116.1 (*C*H-Ar), 122.3 (2×*C*H-Ar), 124.3 (*C*H-Ar), 125.3 (3×*C*H-Ar), 126.1 (*C*H-Ar), 126.2 (q, $^1J_{C-F}$ 280.6, *C*F_3), 127.8 (2×*C*H-Ar), 128.9 (*C*H-Ar), 129.4 (2×*C*H-Ar), 129.8 (*C*H-Ar), 136.1 (*C*-Ar), 140.1 (*C*-Ar), 145.6 (*C*-NO_2), 148.9 ($CO_2C(CH_3)_3$), 154.9 (CO_2C-Ar_{PNP}), 168.2 (*C*ON), 172.3 (*C*O_2PNP); **HRMS (NSI$^+$)** exact mass calcd. For $C_{29}H_{29}F_3N_3O_7^+$ (M+NH_4^+) requires m/z 588.1952, found m/z 588.1947 (–0.9 ppm).

(*S*)-*N*-benzyl-4,4,4-trifluoro-3-((*R*)-1-methyl-2-oxo-3-phenylindolin-3-yl)butanamide (53)

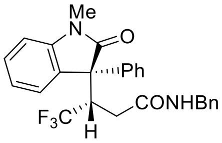

Following General Procedure, 1-methyl-3-phenylindolin-2-one (44.7 mg, 0.2 mmol), 4-nitrophenyl (*E*)-4,4,4-trifluorobut-2-enoate (52.2 mg, 0.2 mmol), (2*S*,3*R*)-HyperBTM (6.2 mg, 0.02 mmol) and DIPEA (35.0 µL, 0.2 mmol) in anhydrous THF (2.0 mL) at room temperature for 48 h. Benzyl amine (109 µL, 5.0 mmol) was added and the reaction was stirred at room temperature overnight. The crude residue (>95:5 dr) was purified by flash silica chromatography (8:1 Petrol : EtOAc, R_f 0.12) to give desired compound (72.0 mg, 80%) as a white solid. mp: 68–70°C; $[\alpha]_D^{20}$ = +229.8 (*c* 0.5, $CHCl_3$); Chiral HPLC analysis, Chiralcel OD-H (93:7 hexane : IPA, flow rate 1 mL/min^{-1}, 254 nm, 30 °C) t_R (major): 26.1 min, t_R (minor): 39.4 min, 98:2 er; ν_{max} (film), 3300 (N-H), 1713 (C=O), 1643 (C=O), 1150 (C-F), 1132 (C-F), 1107 (C-F); **^{1}HNMR ($CDCl_3$, 400 MHz)** δ_H: 2.47–2.58 (2H, m, C*H*$_2$CO), 3.11 (3H, s, NC*H*$_3$), 4.24 (1H, dd, *J* 14.7, 5.8, NHC*H*$_2$Ph), 4.39 (1H, dd, *J* 14.7, 5.8, NHC*H*$_2$Ph), 4.43–4.51 (1H, m , C*H*CF_3), 5.65 (1H, t, *J* 5.1 N*H*CH_2Ph), 6.91 (1H, d, *J* 7.8, C*H*-Ar), 7.10 (1H, t, *J* 7.6, C*H*-Ar), 7.14–7.16 7 (2H, m, C*H*-Ar), 7.20–7.29 (7H, m, C*H*-Ar), 7.37–7.44 (3H, m, C*H*-Ar); **$^{19}F\{^1H\}$ NMR (376 MHz, $CDCl_3$)** δ_F: –66.0

(s, CF_3); **^{13}C NMR (CDCl$_3$, 100 MHz)** δ_C:, 26.7 (NCH_3), 32.8 (CH_2CO), 44.0 (CH_2Ph), 46.2 (q, $^2J_{C\text{-}F}$ 24.6, CHCF$_3$), 55.7 (C(3)), 109.1 (CH-Ar), 122.2 (CH-Ar), 126.3 (CH-Ar), 126.6 (CH-Ar), 125.2 (q, $^1J_{C\text{-}F}$ 280.5, CF$_3$), 127.3 (CH-Ar), 127.6 (CH-Ar), 127.7 (2×CH-Ar), 127.9 (2×CH-Ar), 128.3 (CH-Ar), 128.7 (2×CH-Ar), 129.0 (2×CH-Ar), 129.2 (CH-Ar), 129.3 (C-Ar), 136.8 (C-Ar), 137.8 (C-Ar), 144.1 (C-Ar), 169.0 (CON), 176.3 (CONHBn); **HRMS (NSI$^+$)** exact mass calcd. For $C_{26}H_{24}F_3N_2O_2^+$ (M+H$^+$) requires m/z 453.1784, found m/z 453.1779 (–1.1 ppm).

4-Nitrophenyl (*S*)-3-((*R*)-1-benzyl-2-oxo-3-phenylindolin-3-yl)-4,4,4-trifluorobutanoate (54)

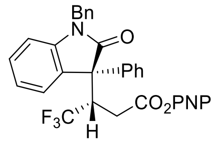

Following General Procedure **9**, 1-benzyl-3-phenylindolin-2-one (59.9 mg, 0.2 mmol), 4-nitrophenyl (*E*)-4,4,4-trifluorobut-2-enoate (52.2 mg, 0.2 mmol), (2*S*,3*R*)-HyperBTM (6.2 mg, 0.02 mmol) and DIPEA (35.0 µL, 0.2 mmol) in anhydrous THF (2.0 mL) at room temperature for 48 h. The reaction mixture was concentrated and the crude residue (>95:5 dr) was purified by column chromatography (95:5 Petrol : EtOAc, R$_f$ 0.12) afforded the desired compound (82.8 mg, 74%) as a white solid. mp: 156–157°C; $[\alpha]_D^{20}$ = +224.8 (*c* 0.5, CHCl$_3$); Chiral HPLC analysis, Chiralcel AS-H (96.5:3.5 hexane : IPA, flow rate 1 mL/min^{-1}, 254 nm, 30 °C) t$_R$ (major): 20.2 min, t$_R$ (minor): 26.6 min, 98.5:1.5 er; ν_{max} (film), 2924 (C-H), 1763 (C=O), 1718 (C=O), 1140 (C-F), 1121 (C-F); **^{1}HNMR (CDCl$_3$, 400 MHz)** δ_H: 2.95 (1H, dd, *J* 13.5, 2.0, CH^AH^BCO), 3.13 (1H, dd, *J* 13.4, 7.5, CH^AH^BCO), 4.49–4.53 (1H, m, CHCF$_3$), 4.88 (1H, d, *J* 12.6, CH_2Ph), 4.93 (1H, d, *J* 12.6, CH_2Ph), 6.88 (1H, d, *J* 6.3, CH-Ar), 7.19–7.25 (5H, m, CH-Ar), 7.26–7.31 (3H, m, CH-Ar), 7.35–7.42 (5H, m, CH-Ar), 7.50 (2H, d, *J* 5.0, CH-Ar), 8.28–8.31 (2H, m, CH-Ar$_{PNP}$); **^{19}F{^{1}H} NMR (376 MHz, CDCl$_3$)** δ_F: –65.9 (s, CF_3); **^{13}C NMR (CDCl$_3$, 100 MHz)** δ_C:, 31.3 (CH$_2$CO), 44.4 (CH$_2$Ph), 46.4 (q, $^2J_{C\text{-}F}$ 20.3, CHCF$_3$), 55.5 (C(3)), 110.5 (CH-Ar), 122.3 (2×CH-Ar), 122.5 (CH-Ar), 125.3 (2×CH-Ar), 126.3 (q, $^1J_{C\text{-}F}$ 224.4, CF$_3$), 126.5 (2×CH-Ar), 127.2 (CH-Ar), 127.4 (CH-Ar), 127.6 (2×CH-Ar), 127.7 (CH-Ar), 128.7 (CH-Ar), 128.8 (2×CH-Ar), 129.3 (2×CH-Ar), 129.5 (CH-Ar), 135.3 (C-Ar), 136.7 (C-Ar), 143.1 (C-Ar), 145.6 (C-NO$_2$), 154.9 (CO$_2$$C$-Ar$_{PNP}$), 168.3 ($C$ON), 176.1 ($C$OOPNP); **HRMS (NSI$^+$)** exact mass calcd. For $C_{31}H_{24}F_3N_2O_5^+$ (M+H$^+$) requires m/z 561.1632, found m/z 561.1622 (–17.8 ppm).

(*S*)-*N*-benzyl-3-((*R*)-1-benzyl-2-oxo-3-phenylindolin-3-yl)-4,4,4-trifluorobutanamide (55)

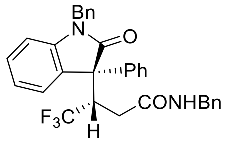

Following General Procedure **9**, 1-benzyl-3-phenylindolin-2-one (59.9 mg, 0.2 mmol), 4-nitrophenyl (*E*)-4,4,4-trifluorobut-2-enoate (52.2 mg, 0.2 mmol), (2*S*,3*R*)-HyperBTM (6.2 mg, 0.02 mmol) and DIPEA (35.0 µL, 0.2 mmol) in anhydrous THF (2.0 mL) at room temperature for 48 h. Benzyl amine (109 µL, 1.0 mmol) was added and the reaction was stirred at room temperature overnight. The crude residue (>95:5 dr) was purified by flash silica chromatography (6:1 Petrol : EtOAc, R$_f$ 0.13) to give desired compound (91.0 mg, 86%) as a white solid. mp: 62–63°C; $[\alpha]_D^{20}$ = +147.0 (*c* 0.5, CHCl$_3$); Chiral HPLC analysis, Chiralcel OD-H (93:7 hexane : IPA, flow rate 1 mL/min^{-1}, 254 nm, 30 °C) t$_R$ (major): 34.5 min, t$_R$ (minor): 48.7 min, >99:1 er; ν_{max} (film), 3314 (N-H), 1715 (C=O), 1651 (C=O), 1188 (C-F),

1149 (C-F), 1115 (C-F); **^{1}HNMR ($CDCl_3$, 400 MHz)** δ_H: 2.57–2.67 (2H, m, C*H*$_2$CO), 4.32 (1H, dd, *J* 14.6, 5.8, NHC*H*$_2$Ph), 4.47 (1H, dd, *J* 14.6, 5.8, NHC*H*$_2$Ph), 4.59–4.67 (1H, m , C*H*CF_3), 4.87 (2H, s, C*H*$_2$Ph), 5.60–5.63 (1H, m, N*H*CH_2Ph), 6.83 (1H, d, *J* 7.8, C*H*-Ar), 7.17–7.37 (17H, m, C*H*-Ar), 7.46 (2H, d, *J* 6.6, C*H*-Ar); **^{19}F{^{1}H} NMR (376 MHz, $CDCl_3$)** δ_F: –65.6 (s, C*F*$_3$); **^{13}C NMR ($CDCl_3$, 100 MHz)** δ_C:, 32.9 (*C*H_2CO), 44.0 (*C*H_2Ph), 44.3 (*C*H_2Ph), 45.9 (q, $^2J_{C-F}$ 24.7, *C*HCF_3), 55.8 (*C*(3)), 110.2 (*C*H-Ar), 122.2 (2×*C*H-Ar), 126.3 (*C*H-Ar), 126.7 (q, $^1J_{C-F}$ 280.5, *C*F_3), 127.2 (2×*C*H-Ar), 127.4 (*C*H-Ar), 127.6 (*C*H-Ar), 127.7 (2×*C*H-Ar), 127.9 (2×*C*H-Ar), 128.3 (*C*H-Ar), 128.7 (4×*C*H-Ar), 129.0 (2×*C*H-Ar), 129.2 (*C*H-Ar), 135.5 (*C*-Ar), 137.2 (*C*-Ar), 137.7 (*C*-Ar), 143.2 (*C*-Ar), 168.9 (*C*ON), 176.4 (*C*ONHBn); **HRMS (NSI$^+$)** exact mass calcd. For $C_{32}H_{28}F_3N_2O_2^+$ (M+H$^+$) requires m/z 529.2097, found m/z 529.2086 (–2.1 ppm).

(*R*)-1-Benzyl-3-phenyl-3-((*S*)-1,1,1-trifluoro-4-oxo-4-(pyrrolidin-1-yl)butan-2-yl)indolin-2-one (56)

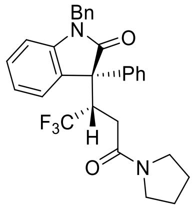

Following General Procedure **9**, 1-benzyl-3-phenylindolin-2-one (59.9 mg, 0.2 mmol), 4-nitrophenyl (*E*)-4,4,4-trifluorobut-2-enoate (52.2 mg, 0.2 mmol), (2*S*,3*R*)-HyperBTM (6.2 mg, 0.02 mmol) and DIPEA (35.0 µL, 0.2 mmol) in anhydrous THF (2.0 mL) at room temperature for 48 h. Pyrrolidine (82 µL, 1.0 mmol) was added and the reaction was stirred at room temperature overnight. The crude residue (>95:5 dr) was purified by flash silica chromatography (6:1 to 2:1 Petrol : EtOAc, R_f 0.12) to give desired compound (80.0 mg, 81%) as a white solid. mp: 125–126°C; $[\alpha]_D^{20}$ = +145.4 (*c* 0.5, $CHCl_3$); Chiral HPLC analysis, Chiralcel OD-H (90:10 hexane : IPA, flow rate 1 mL/min^{-1}, 254 nm, 30 °C) t_R (major): 22.2 min, t_R (minor): 14.3 min, 98:2 er; ν_{max} (film), 2876 (C-H), 1715 (C=O), 1639 (C=O), 1188 (C-F), 1150 (C-F), 1113 (C-F); **^{1}HNMR ($CDCl_3$, 400 MHz)** δ_H: 1.74–1.92 (4H, m, C*H*$_{2(pyrr)}$), 2.53 (1H, dd, *J* 16.1, 2.7, C*H*$_2$CO), 2.77 (1H, q, *J* 7.7, C*H*$_2$CO), 3.05–3.11 (1H, m, C*H*$_{2(pyrr)}$), 3.32–3.39 (2H, m, C*H*$_{2(pyrr)}$), 3.44–3.50 (1H, m, C*H*$_{2(pyrr)}$), 4.74–4.79 (1H, m , C*H*CF_3), 4.86 (1H, d, *J* 15.8, C*H*$_2$Ph), 4.91 (1H, d, *J* 15.8, C*H*$_2$Ph), 6.83 (1H, d, *J* 7.8, C*H*-Ar), 7.13–7.19 (3H, m, C*H*-Ar), 7.23–7.37 (8H, m, C*H*-Ar), 7.46 (2H, d, *J* 6.9, C*H*-Ar); **^{19}F{^{1}H} NMR (376 MHz, $CDCl_3$)** δ_F: –65.8 (s, C*F*$_3$); **^{13}C NMR ($CDCl_3$, 100 MHz)** δ_C: 24.3 (*C*$H_{2(pyrr)}$), 25.9 (*C*$H_{2(pyrr)}$), 30.7 (*C*H_2CO), 44.3 (*C*H_2Ph), 45.4 (q, $^2J_{C-F}$ 25.2, *C*HCF_3), 46.0 (*C*$H_{2(pyrr)}$), 46.4 (*C*$H_{2(pyrr)}$), 56.0 (*C*(3)), 110.1 (*C*H-Ar), 122.2 (*C*H-Ar), 126.2 (*C*H-Ar), 126.9 (q, $^1J_{C-F}$ 280.3, *C*F_3), 127.2 (2×*C*H-Ar), 127.6 (*C*H-Ar), 127.7 (*C*H-Ar), 127.9 (*C*H-Ar), 128.2 (*C*H-Ar), 128.7 (2×*C*H-Ar), 128.8 (2×*C*H-Ar), 129.1 (*C*H-Ar), 135.6 (*C*-Ar), 137.2 (*C*-Ar), 137.7 (*C*-Ar), 143.2 (*C*-Ar), 167.4 (*C*ON), 176.5 (*C*ONHBn); **HRMS (NSI$^+$)** exact mass calcd. For $C_{29}H_{28}F_3N_2O_2^+$ (M+H$^+$) requires m/z 493.2097, found m/z 493.2085 (–2.4 ppm).

(*R*)-1-Benzyl-3-phenyl-3-((*S*)-1,1,1-trifluoro-4-morpholino-4-oxobutan-2-yl)indolin-2-one (57)

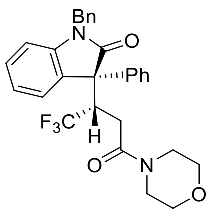

Following General Procedure **9**, 1-benzyl-3-phenylindolin-2-one (59.9 mg, 0.2 mmol), 4-nitrophenyl (*E*)-4,4,4-trifluorobut-2-enoate (52.2 mg, 0.2 mmol), (2*S*,3*R*)-HyperBTM (6.2 mg, 0.02 mmol) and DIPEA (35.0 µL, 0.2 mmol) in anhydrous THF (2.0 mL) at room temperature for 48 h. Morpholine (87 µL, 1.0 mmol) was added and the reaction was stirred at room temperature overnight. The crude residue (>95:5 dr) was purified by flash silica chromatography (5:1 to 2:1 Petrol : EtOAc, R_f 0.16) to give desired compound (76.0 mg, 75%) as a white solid. mp: 145–146°C; $[\alpha]_D^{20}$ = +220.4 (*c* 0.5, $CHCl_3$); Chiral HPLC analysis, Chiralcel OD-H (93:7 hexane : IPA, flow rate 1 mL/min^{-1}, 254 nm, 30 °C) t_R (major): 34.3 min, t_R (minor): 30.5 min, 98.5:1.5 er; ν_{max} (film), 2852 (C-H), 1713 (C=O), 1643 (C=O), 1188 (C-F), 1152 (C-F), 1111 (C-F); **^{1}HNMR ($CDCl_3$, 400 MHz)** δ_H: 2.46 (1H, dd, *J* 16.2, 2.0, CH_2CO), 2.81 (1H, q, *J* 8.0, CH_2CO), 3.17–3.61 (8H, m, C$H_{2(morph)}$), 4.69-4.78 (1H, m, C*H*CF$_3$), 4.81 (1H, d, *J* 15.9, CH_2Ph), 4.86 (1H, d, *J* 15.8, CH_2Ph), 6.79 (1H, d, *J* 7.8, C*H*-Ar), 7.09–7.15 (3H, m, C*H*-Ar), 7.19–7.32 (8H, m, C*H*-Ar), 7.40 (2H, d, *J* 6.0, C*H*-Ar); **^{19}F{^{1}H} NMR (376 MHz, $CDCl_3$)** δ_F: –65.6 (s, CF_3); **^{13}C NMR ($CDCl_3$, 100 MHz)** δ_C:, 28.6 (CH_2CO), 42.6 (*C*$H_{2(morph)}$), 44.3 (*C*H_2Ph), 45.5 (q, $^2J_{C-F}$ 24.8, *C*HCF$_3$), 45.9 (*C*$H_{2(morph)}$), 56.0 (*C*(3)), 66.3 (*C*$H_{2(morph)}$), 66.8 (*C*$H_{2(morph)}$), 110.2 (*C*H-Ar), 122.2 (*C*H-Ar), 126.8 (q, $^1J_{C-F}$ 280.3, *C*F$_3$), 126.2 (*C*H-Ar), 127.2 (2×*C*H-Ar), 127.5 (*C*H-Ar), 127.6 (*C*H-Ar), 127.8 (2×*C*H-Ar), 128.3 (*C*H-Ar), 128.7 (2×*C*H-Ar), 129.0 (*CH*-Ar), 129.2 (*C*H-Ar), 135.5 (*C*-Ar), 137.2 (*C*-Ar), 137.7 (*C*-Ar), 143.2 (*C*-Ar), 167.9 (*C*ON), 176.3 (*C*ONHBn); **HRMS (NSI$^+$)** exact mass calcd. For $C_{29}H_{28}F_3N_2O_3^+$ (M+H$^+$) requires m/z 509.2047, found m/z 509.2034 (–2.6 ppm).

(*S*)-*N*-benzyl-3-((*R*)-1-benzyl-5-methoxy-2-oxo-3-phenylindolin-3-yl)-4,4,4-trifluorobutanamide (58)

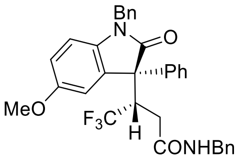

Following General Procedure **9**, 1-benzyl-5-methoxy-3-phenylindolin-2-one (65.9 mg, 0.2 mmol), 4-nitrophenyl (*E*)-4,4,4-trifluorobut-2-enoate (52.2 mg, 0.2 mmol), (2*S*,3*R*)-HyperBTM (6.2 mg, 0.02 mmol) and DIPEA (35.0 µL, 0.2 mmol) in anhydrous THF (2.0 mL) at room temperature for 48 h. Benzyl amine (109 µL, 1.0 mmol) was added and the reaction was stirred at room temperature overnight. The crude residue (90:10 dr) was purified by flash silica chromatography (6:1 Petrol : EtOAc, R_f 0.16) to give desired compound (80.0 mg, 72%) as a white solid. mp: 79–81°C; $[\alpha]_D^{20}$ = +213.0 (*c* 0.5, $CHCl_3$); Chiral HPLC analysis, Chiralcel AS-H (90:10 hexane : IPA, flow rate 1.0 mL/min^{-1}, 254 nm, 30 °C) t_R (major): 19.5 min, t_R (minor): 15.8 min, 98:2 er; ν_{max} (film), 3310 (N-H), 1724 (C=O), 1649 (C=O), 1180 (C-F), 1153 (C-F), 1121 (C-F); **^{1}HNMR ($CDCl_3$, 400 MHz)** δ_H: 2.54–2.65 (2H, m, CH_2CO), 3.79 (3H, s, OCH_3), 4.32 (1H, dd, *J* 14.6, 5.8, NHCH_2Ph), 4.47 (1H, dd, *J* 14.6, 5.8, NHCH_2Ph), 4.59–4.64 (1H, m , C*H*CF$_3$), 4.82 (1H, d, *J* 15.8, CH_2Ph), 4.87 (1H, d, *J* 15.8, CH_2Ph), 5.65 (1H, t, *J* 5.4, N*H*CH$_2$Ph), 6.71 (1H, d, *J* 8.6, C*H*-Ar), 6.82 (1H, dd, *J* 8.6, 2.5, C*H*-Ar), 6.89 (1H, d, *J* 2.4, C*H*-Ar), 7.16–7.39 (12H, m, C*H*-Ar), 7.47 (2H, d, *J* 6.9, C*H*-Ar); **^{19}F{^{1}H} NMR (376 MHz, $CDCl_3$)** δ_F: –65.5 (s, CF_3); **^{13}C NMR ($CDCl_3$, 100 MHz)** δ_C: 32.8 (*C*H$_2$CO), 44.0 (*C*H$_2$Ph), 44.4 (*C*H$_2$Ph), 45.7 (q, $^2J_{C-F}$ 25.0, *C*HCF$_3$), 55.8 (O*C*H$_3$), 56.0 (*C*(3)), 110.3 (*C*H-Ar), 112.6 (*C*H-Ar), 114.5 (*C*H-Ar), 126.8 (q, $^1J_{C-F}$ 280.5, *C*F$_3$), 127.2 (2×*C*H-Ar), 127.6 (2×*C*H-Ar), 127.7 (2×*C*H-Ar), 127.9 (2×*C*H-Ar), 128.3 (*C*H-Ar), 128.7 (4×*C*H-Ar), 128.9 (*C*H-Ar), 129.0 (*C*-Ar) , 135.6 (*C*-Ar), 136.8 (*C*-Ar), 137.1 (*C*-Ar), 137.8 (*C*-Ar), 141.8 (*C*-Ar), 155.5 (*C*-Ar), 168.9 (*C*ON), 176.0 (*C*ONHBn); **HRMS (NSI$^+$)** exact mass calcd. For $C_{33}H_{30}F_3N_2O_3^+$ (M+H$^+$) requires m/z 559.2203, found m/z 559.2191 (–2.2 ppm).

(*S*)-*N*-benzyl-3-((*R*)-1-benzyl-5-chloro-2-oxo-3-phenylindolin-3-yl)-4,4,4-trifluorobutanamide (59)

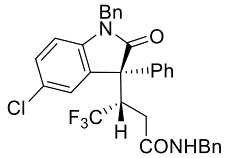

Following General Procedure **9**, 1-benzyl-5-chloro-3-phenylindolin-2-one (66.7 mg, 0.2 mmol), 4-nitrophenyl (*E*)-4,4,4-trifluorobut-2-enoate (52.2 mg, 0.2 mmol), (2*S*,3*R*)-HyperBTM (6.2 mg, 0.02 mmol) and DIPEA (35.0 µL, 0.2 mmol) in anhydrous THF (2.0 mL) at room temperature for 48 h. Benzyl amine (109 µL, 1.0 mmol) was added and the reaction was stirred at room temperature overnight. The crude residue (>95:5 dr) was purified by flash silica chromatography (6:1 Petrol : EtOAc, R_f 0.16) to give desired compound (109.0 mg, 97%) as a white solid. mp: 94–95°C; $[\alpha]_D^{20}$ = +209.8 (*c* 0.5, $CHCl_3$); Chiral HPLC analysis, Chiralcel IC (93:7 hexane : IPA, flow rate 1 mL/min^{-1}, 254 nm, 50 °C) t_R (major): 52.7 min, t_R (minor): 67.8 min, 93:7 er; ν_{max} (film), 3337 (N-H), 1717 (C=O), 1651 (C=O), 1186 (C-F), 1153 (C-F), 1119 (C-F); **^{1}HNMR ($CDCl_3$, 400 MHz)** δ_H: 2.54–2.65 (2H, m, C*H*$_2$CO), 4.34 (1H, dd, *J* 14.6, 5.8, NHC*H*$_2$Ph), 4.48 (1H, dd, *J* 14.6, 5.8, NHC*H*$_2$Ph), 4.61–4.66 (1H, m , C*H*CF$_3$), 4.84 (1H, d, *J* 15.8, C*H*$_2$Ph), 4.89 (1H, d, *J* 15.8, C*H*$_2$Ph), 5.62 (1H, t, *J* 5.3, N*H*CH$_2$Ph), 6.74 (1H, d, *J* 8.3, C*H*-Ar), 7.14–7.16 (2H, m, C*H*-Ar), 7.21–7.23 (2H, m, C*H*-Ar), 7.25–7.40 (11H, m, C*H*-Ar), 7.43–7.47 (2H, m, C*H*-Ar); **^{19}F{^{1}H} NMR (376 MHz, $CDCl_3$)** δ_F: –65.5 (s, C*F*$_3$); **^{13}C NMR ($CDCl_3$, 100 MHz)** δ_C: 32.7 (*C*H$_2$CO), 44.1 (*C*H$_2$Ph), 44.4 (*C*H$_2$Ph), 45.7 (q, $^2J_{C-F}$ 25.1, *C*HCF$_3$), 56.0 (*C*(3)), 111.1 (*C*H-Ar), 126.4 (*C*H-Ar), 126.6 (q, $^1J_{C-F}$ 280.9, *C*F$_3$), 127.2 (2×*C*H-Ar), 127.6 (2×*C*H-Ar), 127.7 (2×*C*H-Ar), 127.8 (*C*H-Ar), 127.9 (2×*C*H-Ar), 128.6 (*C*H-Ar), 128.8 (4×*C*H-Ar), 129.2 (4×*C*H-Ar), 135.0 (*C*-Ar), 136.5 (*C*-Ar), 137.6 (*C*-Ar), 141.8 (*C*-Ar), 168.6 (*C*ON), 175.9 (*C*ONHBn); **HRMS (NSI$^+$)** exact mass calcd. For $C_{32}H_{27}ClF_3N_2O_2^+$ (M+H$^+$) requires m/z 563.1708, found m/z 563.1701 (–1.3 ppm).

(*S*)-*N*-benzyl-3-((*R*)-1-benzyl-6-chloro-2-oxo-3-phenylindolin-3-yl)-4,4,4-trifluorobutanamide (60)

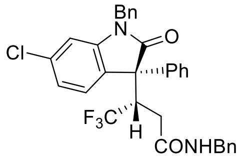

Following General Procedure **9**, 1-benzyl-6-chloro-3-phenylindolin-2-one (66.7 mg, 0.2 mmol), 4-nitrophenyl (*E*)-4,4,4-trifluorobut-2-enoate (52.2 mg, 0.2 mmol), (2*S*,3*R*)-HyperBTM (6.2 mg, 0.02 mmol) and DIPEA (35.0 µL, 0.2 mmol) in anhydrous THF (2.0 mL) at room temperature for 48 h. Benzyl amine (109 µL, 1.0 mmol) was added and the reaction was stirred at room temperature overnight. The crude residue (>95:5 dr) was purified by flash silica chromatography (6:1 Petrol : EtOAc, R_f 0.15) to give desired compound (101.0 mg, 90%) as a white solid. mp: 98–99°C; $[\alpha]_D^{20}$ = +185.2 (*c* 0.5, $CHCl_3$); Chiral HPLC analysis, Chiralcel OD-H (93:7 hexane : IPA, flow rate 1 mL/min^{-1}, 254 nm, 30 °C) t_R (major): 29.6 min, t_R (minor): 53.2 min, 95:5 er; ν_{max} (film), 3345 (N-H), 1717 (C=O), 1651 (C=O), 1188 (C-F), 1152 (C-F), 1119 (C-F); **^{1}HNMR ($CDCl_3$, 400 MHz)** δ_H: 2.54–2.63 (2H, m, C*H*$_2$CO), 4.31 (1H, dd, *J* 14.6, 5.8, NHC*H*$_2$Ph), 4.46 (1H, dd, *J* 14.6, 5.8, NHC*H*$_2$Ph), 4.59–4.65 (1H, m , C*H*CF$_3$), 4.84 (2H, s, C*H*$_2$Ph), 5.60 (1H, t, *J* 5.4, N*H*CH$_2$Ph), 6.82 (1H, d, *J* 1.8, C*H*-Ar), 7.10 (1H, dd, *J* 8.0, 1.9, C*H*-Ar), 7.15–7.22 (5H, m, C*H*-Ar), 7.24–7.38 (9H, m, C*H*-Ar), 7.43 (2H, d, *J* 6.2, C*H*-Ar); **^{19}F{^{1}H} NMR (376 MHz, $CDCl_3$)** δ_F: –65.6 (s, C*F*$_3$); **^{13}C NMR ($CDCl_3$, 100 MHz)** δ_C: 32.8 (*C*H$_2$CO), 44.1 (*C*H$_2$Ph), 44.4 (*C*H$_2$Ph), 45.8 (q, $^2J_{C-F}$ 25.2, *C*HCF$_3$), 55.6 (*C*(3)), 110.7 (*C*H-Ar), 122.3 (*C*H-Ar), 125.8 (*C*H-Ar), 126.6

(q, $^1J_{C\text{-}F}$ 280.6, CF_3), 127.1 (*C*H-Ar), 127.2 (2×*C*H-Ar), 127.6 (2×*C*H-Ar), 127.7 (*C*H-Ar), 127.8 (*C*H-Ar), 127.9 (2×*C*H-Ar), 128.6 (*C*H-Ar), 128.8 (2×*C*H-Ar), 128.9 (2×*C*H-Ar), 129.2 (2×*C*H-Ar), 134.9(*C*-Ar), 135.1 (*C*-Ar), 136.6 (*C*-Ar), 137.7 (*C*-Ar), 144.5 (*C*-Ar), 168.7 (*C*ON), 176.4 (*C*ONHBn); **HRMS (NSI$^+$)** exact mass calcd. For $C_{32}H_{27}ClF_3N_2O_2^+$ (M+H$^+$) requires m/z 563.1708, found m/z 563.1698 (–1.8 ppm).

(*S*)-*N*-benzyl-3-((*R*)-1-benzyl-7-chloro-2-oxo-3-phenylindolin-3-yl)-4,4,4-trifluorobutanamide (61)

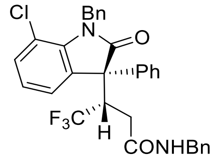

Following General Procedure **9**, 1-benzyl-7-chloro-3-phenylindolin-2-one (66.7 mg, 0.2 mmol), 4-nitrophenyl (*E*)-4,4,4-trifluorobut-2-enoate (52.2 mg, 0.2 mmol), (2*S*,3*R*)-HyperBTM (6.2 mg, 0.02 mmol) and DIPEA (35.0 µL, 0.2 mmol) in anhydrous THF (2.0 mL) at –40 °C for 48 h. Benzyl amine (109 µL, 1.0 mmol) was added and the reaction was stirred at room temperature overnight. The crude residue (>95:5 dr) was purified by flash silica chromatography (6:1 Petrol : EtOAc, R_f 0.15) to give desired compound (110.0 mg, 98%) as a white solid. mp: 97–98°C; $[\alpha]_D^{20}$ = +209.8 (*c* 0.6, $CHCl_3$); Chiral HPLC analysis, Chiralcel OD-H (90:10 hexane : IPA, flow rate 1 mL/min^{-1}, 254 nm, 30 °C) t_R (major): 15.0 min, t_R (minor): 34.1 min, 90:10 er; ν_{max} (film), 3323 (N-H), 1699 (C=O), 1651 (C=O), 1179 (C-F), 1148 (C-F), 1115 (C-F); **^{1}HNMR ($CDCl_3$, 400 MHz)** δ_H: 2.55–2.65 (2H, m, C*H*$_2$CO), 4.32 (1H, dd, *J* 14.6, 5.8, NHC*H*$_2$Ph), 4.47 (1H, dd, *J* 14.6, 5.8, NHC*H*$_2$Ph), 4.60–4.69 (1H, m , C*H*CF$_3$), 5.24 (1H, d, *J* 16.3, C*H*$_2$Ph), 5.37 (1H, d, *J* 16.3, C*H*$_2$Ph), 5.60 (1H, t, *J* 5.1, N*H*CH$_2$Ph), 7.04–7.10 (3H, m, C*H*-Ar), 7.19–7.22 (6H, m, C*H*-Ar), 7.27–7.38 (7H, m, C*H*-Ar), 7.42 (2H, d, *J* 6.0, C*H*-Ar); **^{19}F{^{1}H} NMR (376 MHz, $CDCl_3$)** δ_F: –65.4 (s, C*F$_3$*); **^{13}C NMR ($CDCl_3$, 100 MHz)** δ_C: 32.8 (*C*H$_2$CO), 44.1 (*C*H$_2$Ph), 45.4 (*C*H$_2$Ph), 45.8 (q, $^2J_{C\text{-}F}$ 24.8, *C*HCF$_3$), 55.5 (*C*(3)), 116.4 (*C*H-Ar), 123.1 (*C*H-Ar), 124.9 (*C*H-Ar), 126.3 (2×*C*H-Ar), 126.6 (q, $^1J_{C\text{-}F}$ 280.6, *C*F$_3$), 127.0 (*C*H-Ar), 127.7 (2×*C*H-Ar), 127.9 (2×*C*H-Ar), 128.4 (2×*C*H-Ar), 128.6 (*C*H-Ar), 128.8 (2×*C*H-Ar), 129.2 (2×*C*H-Ar), 130.4 (*C*-Ar), 131.9 (*C*-Ar), 136.8 (*C*-Ar), 137.4 (*C*-Ar), 137.7 (*C*-Ar), 139.6 (*C*-Ar), 144.5 (*C*-Ar), 168.7 (*C*ON), 177.0 (*C*ONHBn); **HRMS (NSI$^+$)** exact mass calcd. For $C_{32}H_{27}ClF_3N_2O_2^+$ (M+H$^+$) requires m/z 563.1708, found m/z 563.1698 (–1.8 ppm).

(*S*)-*N*-benzyl-3-((*R*)-1-benzyl-3-(4-methoxyphenyl)-2-oxoindolin-3-yl)-4,4,4-trifluorobutanamide (62)

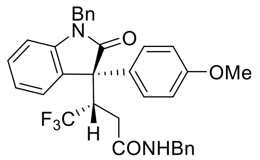

Following General Procedure **9**, 1-benzyl-3-(4-methoxyphenyl)indolin-2-one (65.9 mg, 0.2 mmol), 4-nitrophenyl (*E*)-4,4,4-trifluorobut-2-enoate (52.2 mg, 0.2 mmol), (2*S*,3*R*)-HyperBTM (6.2 mg, 0.02 mmol) and DIPEA (35.0 µL, 0.2 mmol) in anhydrous THF (2.0 mL) at room temperature for 48 h. Benzyl amine (109 µL, 1.0 mmol) was added and the reaction was stirred at room temperature overnight. The crude residue (>95:5 dr) was purified by flash silica chromatography (95:5 Petrol : EtOAc, R_f 0.15) to give desired compound (77.0 mg, 69%) as a white solid. mp: 74–75°C; $[\alpha]_D^{20}$ = +194.6 (*c* 0.5, $CHCl_3$);

Chiral HPLC analysis, Chiralcel OD-H (90:10 hexane : IPA, flow rate 1 mL/min^{-1}, 254 nm, 30 °C) t_R (major): 20.2 min, t_R (minor): 27.7 min, >99:1 er; ν_{max} (film), 3321 (N-H), 1707 (C=O), 1607 (C=O), 1184 (C-F), 1149 (C-F), 1111 (C-F); **^{1}HNMR (CDCl$_3$, 400 MHz)** δ_H: 2.57-2.66 (2H, m, C*H*$_2$CO), 3.80 (3H, s, C*H*$_3$O), 4.33 (1H, dd, *J* 14.6, 5.7, NHC*H*$_2$Ph), 4.48 (1H, dd, *J* 14.6, 5.7, NHC*H*$_2$Ph), 4.53–4.58 (1H, m , C*H*CF$_3$), 4.88 (2H, s, C*H*$_2$Ph), 5.55 (1H, br, N*H*CH$_2$Ph), 6.81 (1H, d, *J* 7.7, C*H*-Ar), 6.87–6.91 (2H, m, C*H*-Ar), 7.09–7.13 (1H, m, C*H*-Ar), 7.16-7.39 (14H, m, C*H*-Ar); **^{19}F{^{1}H} NMR (376 MHz, CDCl$_3$)** δ_F: –65.7 (s, C*F$_3$*); **^{13}C NMR (CDCl$_3$, 100 MHz)** δ_C: 32.9 (*C*H$_2$CO), 44.1 (*C*H$_2$Ph), 44.2 (*C*H$_2$Ph), 45.9 (q, $^2J_{C\text{-}F}$ 24.5, *C*HCF$_3$), 55.1 (*C*(3)), 55.3 (*C*H$_3$O), 110.2 (*C*H-Ar), 114.3 (2×*C*H-Ar), 122.1 (2×*C*H-Ar), 126.6 (q, $^1J_{C\text{-}F}$ 280.5, *C*F$_3$), 127.2 (2×*C*H-Ar), 127.6 (2×*C*H-Ar), 127.7 (*C*H-Ar), 127.9 (2×*C*H-Ar), 128.7 (2×*C*H-Ar), 128.8 (2×*C*H-Ar), 128.9 (*C*H-Ar), 129.0 (2×*C*H-Ar), 129.1 (*C*H-Ar), 135.5 (*C*-Ar), 137.7 (*C*-Ar), 143.2 (*C*-Ar), 159.5 (MeO*C*-Ar), 169.0 (*C*ON), 176.7 (*C*ONHBn); **HRMS (NSI$^+$)** exact mass calcd. For $C_{33}H_{30}F_3N_2O_3^+$ (M+H$^+$) requires m/z 559.2203, found m/z 559.2195 (–1.4 ppm).

(*S*)-3-((*R*)-3-(Benzo[*d*][1,3]dioxol-5-yl)-1-benzyl-2-oxoindolin-3-yl)-*N*-benzyl-4,4,4-trifluorobutanamide (63)

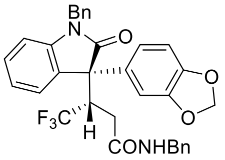

Following General Procedure **9**, 3-(benzo[*d*][1,3]dioxol-5-yl)-1-benzylindolin-2-one (68.7 mg, 0.2 mmol), 4-nitrophenyl (*E*)-4,4,4-trifluorobut-2-enoate (52.2 mg, 0.2 mmol), (2*S*,3*R*)-HyperBTM (6.2 mg, 0.02 mmol) and DIPEA (35.0 µL, 0.2 mmol) in anhydrous THF (2.0 mL) at room temperature for 48 h. Benzyl amine (109 µL, 1.0 mmol) was added and the reaction was stirred at room temperature overnight. The crude residue (>95:5 dr) was purified by flash silica chromatography (6:1 Petrol : EtOAc, R_f 0.14) to give desired compound (78.0 mg, 68%) as a white solid. mp: 83–85°C; $[\alpha]_D^{20}$ = +182.4 (*c* 0.5, CHCl$_3$); Chiral HPLC analysis, Chiralcel OD-H (90:10 hexane : IPA, flow rate 1 mL/min^{-1}, 254 nm, 30 °C) t_R (major): 30.2 min, t_R (minor): 52.1 min, 99:1 er; ν_{max} (film), 3209 (N-H), 1701 (C=O), 1655 (C=O), 1151 (C-F), 1113 (C-F); **^{1}HNMR (CDCl$_3$, 400 MHz)** δ_H: 2.58–2.69 (2H, m, C*H*$_2$CO), 4.36 (1H, dd, *J* 14.6, 5.3, NHC*H*$_2$Ph), 4.45–4.53 (2H, m, NHC*H*$_2$Ph, C*H*CF$_3$), 4.59–4.67 (1H, m , C*H*CF$_3$), 4.86 (1H, d, *J* 16.6, C*H*$_2$Ph), 4.90 (1H, d, *J* 16.6, C*H*$_2$Ph), 5.62 (1H, t, *J* 5.4, N*H*CH$_2$Ph), 5.95 (1H, d, *J* 1.4, OC*H*$_2$O), 5.97 (1H, t, *J* 1.4, OC*H*$_2$O), 6.81 (1H, d, *J* 7.8, C*H*-Ar), 6.87 (1H, d, *J* 7.8, C*H*-Ar), 7.07–7.13 (2H, m, C*H*-Ar), 7.19–7.21 (2H, m, C*H*-Ar), 7.23–7.37 (10H, m, C*H*-Ar); **^{19}F{^{1}H} NMR (376 MHz, CDCl$_3$)** δ_F: –65.6 (s, C*F$_3$*); **^{13}C NMR (CDCl$_3$, 100 MHz)** δ_C: 32.9 (*C*H$_2$CO), 44.1 (*C*H$_2$Ph), 44.3 (*C*H$_2$Ph), 46.1 (q, $^2J_{C\text{-}F}$ 24.6, *C*HCF$_3$), 55.4 (*C*(3)), 101.4 (O*C*H$_2$O), 101.4 (*C*H-Ar), 108.3 (*C*H-Ar), 108.4 (*C*H-Ar), 110.2 (*C*H-Ar), 121.5 (*C*H-Ar), 122.2 (*C*H-Ar), 126.2 (*C*H-Ar), 126.6 (q, $^1J_{C\text{-}F}$ 280.7, *C*F$_3$), 127.2 (2×*C*H-Ar), 127.4 (*C*H-Ar), 127.6 (*C*H-Ar), 127.9 (2×*C*H-Ar), 128.7 (2×*C*H-Ar), 128.8 (2×*C*H-Ar), 129.2 (*C*-Ar), 130.7 (*C*-Ar), 135.5 (*C*-Ar), 137.7 (*C*-Ar), 143.1 (*C*-Ar), 147.7 (*C*-Ar), 148.3 (*C*-Ar), 168.9 (*C*ON), 176.4 (*C*ONHBn); **HRMS (NSI$^+$)** exact mass calcd. For $C_{33}H_{28}F_3N_2O_4S^+$ (M+H$^+$) requires m/z 573.1996, found m/z 573.1985 (–1.9 ppm).

(*S*)-*N*-benzyl-3-((*R*)-1-benzyl-3-(naphthalen-2-yl)-2-oxoindolin-3-yl)-4,4,4-trifluorobutanamide (64)

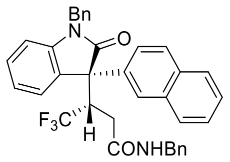

Following General Procedure **9**, 1-benzyl-3-(naphthalen-2-yl)indolin-2-one (69.9 mg, 0.2 mmol), 4-nitrophenyl (*E*)-4,4,4-trifluorobut-2-enoate (52.2 mg, 0.2 mmol), (2*S*,3*R*)-HyperBTM (6.2 mg, 0.02 mmol) and DIPEA (35.0 µL, 0.2 mmol) in anhydrous THF (2.0 mL) at room temperature for 48 h. Benzyl amine (109 µL, 1.0 mmol) was added and the reaction was stirred at room temperature overnight. The crude residue (>95:5 dr) was purified by flash silica chromatography (5:1 Petrol : EtOAc, R_f 0.15) to give desired compound (95.0 mg, 82%) as a white solid. mp: 88–89°C; $[\alpha]_D^{20}$ = +176.2 (*c* 0.5, $CHCl_3$); Chiral HPLC analysis, Chiralcel OD-H (90:10 hexane : IPA, flow rate 1 mL/min^{-1}, 254 nm, 30 °C) t_R (major): 22.2 min, t_R (minor): 46.6 min, 98:2 er; ν_{max} (film), 3319 (N-H), 1715 (C=O), 1651 (C=O), 1184 (C-F), 1150 (C-F), 1109 (C-F); **^{1}HNMR ($CDCl_3$, 400 MHz)** δ_H: 2.57–2.68 (2H, m, C*H*$_2$CO), 4.17 (1H, dd, *J* 14.5, 5.3, NC*H*$_2$Ph), 4.39 (1H, dd, *J* 14.5, 5.7, NHC*H*$_2$Ph), 4.71–4.80 (1H, m , C*H*CF$_3$), 4.86 (1H, d, *J* 15.9, C*H*$_2$Ph), 4.86 (1H, d, *J* 15.9, C*H*$_2$Ph), 5.45 (1H, t, *J* 5.4, N*H*CH$_2$Ph), 6.87 (1H, d, *J* 7.8, C*H*-Ar), 7.07–7.10 (2H, m, C*H*-Ar), 7.16–7.28 (9H, m, C*H*-Ar), 7.34–7.39 (2H, m, C*H*-Ar), 7.45–7.54 (2H, m, C*H*-Ar), 7.66 (1H, s, C*H*-Ar), 7.74 (1H, d, *J* 7.8, C*H*-Ar), 7.85 (1H, d, *J* 8.0, C*H*-Ar), 7.90 (2H, s, C*H*-Ar); **^{19}F{^{1}H} NMR (376 MHz, $CDCl_3$)** δ_F: –65.6 (s, C*F*$_3$); **^{13}C NMR ($CDCl_3$, 100 MHz)** δ_C: 33.0 (*C*H$_2$CO), 44.0 (*C*H$_2$Ph), 44.3 (*C*H$_2$Ph), 45.9 (q, $^2J_{C\text{-}F}$ 24.8, *C*HCF$_3$), 55.9 (*C*(3)), 110.3 (*C*H-Ar), 122.3 (2×*C*H-Ar), 125.1 (*C*H-Ar), 126.4 (2×*C*H-Ar), 126.6 (q, $^1J_{C\text{-}F}$ 271.4, *C*F$_3$), 126.8 (*C*H-Ar), 127.3 (3×*C*H-Ar), 127.5 (*C*H-Ar), 127.6 (2×*C*H-Ar), 127.8 (2×*C*H-Ar), 128.4 (*C*H-Ar), 128.7 (4×*C*H-Ar), 129.1 (*C*H-Ar), 129.3 (*C*H-Ar), 132.9 (*C*-Ar), 133.0 (*C*-Ar), 134.6 (*C*-Ar), 135.5 (*C*-Ar), 137.6 (*C*-Ar), 143.3 (*C*-Ar), 168.8 (*C*ON), 176.3 (*C*ONHBn); **HRMS (NSI$^+$)** exact mass calcd. For $C_{36}H_{30}F_3N_2O_2^+$ (M+H$^+$) requires m/z 579.2254, found m/z 579.2243 (–1.9 ppm).

(*S*)-*N*-benzyl-3-((*R*)-1-benzyl-2-oxo-3-(4-(trifluoromethyl)phenyl)indolin-3-yl)-4,4,4 trifluorobutanamide (65)

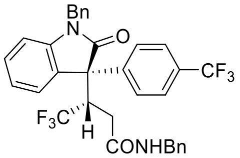

Following General Procedure **9**, 1-methyl-3-(4-(trifluoromethyl)phenyl)indolin-2-one (58.2 mg, 0.2 mmol), 4-nitrophenyl (*E*)-4,4,4-trifluorobut-2-enoate (52.2 mg, 0.2 mmol), (2*S*,3*R*)-HyperBTM (6.2 mg, 0.02 mmol) and DIPEA (35.0 µL, 0.2 mmol) in anhydrous THF (2.0 mL) at –40 °C for 48 h. Benzyl amine (109 µL, 1.0 mmol) was added and the reaction was stirred at room temperature overnight. The crude residue (>95:5 dr) was purified by flash silica chromatography (95:5 Petrol : EtOAc, R_f 0.15) to give desired compound (108.0 mg, 91%) as a white solid. mp: 192–193°C; $[\alpha]_D^{20}$ = +174.0 (*c* 0.5, $CHCl_3$); Chiral HPLC analysis, Chiralcel OD-H (95:5 hexane : IPA, flow rate 1 mL/min^{-1}, 254 nm, 30 °C) t_R (major): 136.9 min, t_R (minor): 201.3 min, 92:8 er; ν_{max} (film), 3308 (N-H), 1713 (C=O), 1647 (C=O), 1157 (C-F), 1122 (C-F), 1107 (C-F); **^{1}HNMR ($CDCl_3$, 400 MHz)** δ_H: 2.51 (1H, d, *J* 14.2, C*H*$_2$CO), 2.64 (1H, dd, *J* 15.7, 8.8, C*H*$_2$CO), 4.30 (1H, dd, *J* 13.4, 4.3, NHC*H*$_2$Ph), 4.45 (1H, dd, *J* 13.4, 4.3, NHC*H*$_2$Ph), 4.62–4.71 (1H, m , C*H*CF$_3$), 4.88 (2H, s, *J* 16.1, C*H*$_2$Ph), 5.61 (1H, t, *J* 5.4, N*H*CH$_2$Ph), 6.87 (1H, d, *J* 7.7, C*H*-Ar), 7.13–7.36 (13H, m, C*H*-Ar), 7.61 (4H, m, C*H*-Ar) ppm; **^{19}F{^{1}H} NMR (376 MHz, $CDCl_3$)** δ_F: –65.7 (s, C*F*$_3$), –62.7 (s, Ar-C*F*$_3$); **^{13}C NMR ($CDCl_3$, 100 MHz)** δ_C: 32.8 (*C*H$_2$CO), 44.1 (*C*H$_2$Ph),

44.4 (CH_2Ph), 45.7 (q, ${}^2J_{C\text{-}F}$ 25.1, *C*HCF$_3$), 55.9 (*C*(3)), 110.5 (*C*H-Ar), 122.6 (*C*H-Ar), 125.9 (2×*C*H-Ar), 126.1 (*C*H-Ar), 126.5 (q, ${}^1J_{C\text{-}F}$ 279.8, *C*F$_3$), 126.6 (q, ${}^1J_{C\text{-}F}$ 271.4, *C*F$_3$), 126.8 (*C*H-Ar), 127.2 (2×*C*H-Ar), 127.7 (2×*C*H-Ar), 127.8 (2×*C*H-Ar), 127.9 (*C*H-Ar), 128.3 (*C*H-Ar), 128.8 (4×*C*H-Ar), 129.6 (*C*-Ar), 130.5 (q, ${}^2J_{C\text{-}F}$ 32.2, CF$_3$-*C*-Ar), 135.2 (*C*-Ar), 137.6 (*C*-Ar), 141.3 (*C*-Ar), 143.2 (*C*-Ar), 168.6 (*C*ON), 175.6 (*C*ONHBn); **HRMS (NSI$^+$)** exact mass calcd. For $C_{33}H_{27}F_6N_2O_2^+$ (M+H$^+$) requires m/z 597.1971, found m/z 597.1960 (–1.8 ppm).

(*S*)-*N*-benzyl-3-((*R*)-1-benzyl-3-(4-fluorophenyl)-2-oxoindolin-3-yl)-4,4,4-trifluorobutanamide (66)

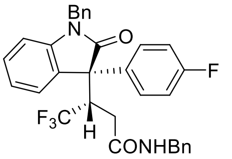

Following General Procedure **9**, 1-benzyl-3-(4-fluorophenyl)indolin-2-one (63.5 mg, 0.2 mmol), 4-nitrophenyl (*E*)-4,4,4-trifluorobut-2-enoate (52.2 mg, 0.2 mmol), (2*S*,3*R*)-HyperBTM (6.2 mg, 0.02 mmol) and DIPEA (35.0 μL, 0.2 mmol) in anhydrous THF (2.0 mL) at room temperature for 48 h. Benzyl amine (109 μL, 1.0 mmol) was added and the reaction was stirred at room temperature overnight. The crude residue (>95:5 dr) was purified by flash silica chromatography (95:5 Petrol : EtOAc, R$_f$ 0.15) to give desired compound (89.0 mg, 81%) as a white solid. mp: 90–91℃; $[\alpha]_D^{20}$ = +198.2 (*c* 0.5, CHCl$_3$); Chiral HPLC analysis, Chiralcel OD-H (95:5 hexane : IPA, flow rate 1 mL/min^{-1}, 254 nm, 30 °C) t$_R$ (major): 49.5 min, t$_R$ (minor): 62.2 min, 98:2 er; ν$_{max}$ (film), 3280 (N-H), 1697 (C=O), 1645 (C=O), 1188 (C-F), 1152 (C-F), 1119 (C-F), 1107 (C-F); **^{1}HNMR (CDCl$_3$, 400 MHz)** δ$_H$: 2.54–2.67 (2H, m, C*H$_2$*CO), 4.34 (1H, dd, *J* 14.6, 5.7, NHC*H$_2$*Ph), 4.47 (1H, dd, *J* 14.6, 5.7, NHC*H$_2$*Ph), 4.54–4.63 (1H, m , C*H*CF$_3$), 4.85 (1H, d, *J* 16.1, C*H$_2$*Ph), 4.90 (1H, d, *J* 16.1, C*H$_2$*Ph), 5.64 (1H, t, *J* 5.3, N*H*CH$_2$Ph), 6.84 (1H, d, *J* 7.8, C*H*-Ar), 7.03 (2H, t, *J* 8.4, C*H*-Ar), 7.11–7.36 (13H, m, C*H*-Ar), 7.41–7.47 (2H, m, C*H*-Ar); **^{19}F{^{1}H} NMR (376 MHz, CDCl$_3$)** δ$_F$: –65.7 (s, C*F$_3$*), –113.6 (s, *F*-Ph); **^{13}C NMR (CDCl$_3$, 100 MHz)** δ$_C$: 32.8 (*C*H$_2$CO), 44.1 (*C*H$_2$Ph), 44.3 (*C*H$_2$Ph), 45.9 (q, ${}^2J_{C\text{-}F}$ 25.2, *C*HCF$_3$), 55.2 (*C*(3)), 110.3 (*C*H-Ar), 115.8 (*C*H-Ar), 116.0 (*C*H-Ar), 122.4 (*C*H-Ar), 126.1 (*C*H-Ar), 126.7 (q, ${}^1J_{C\text{-}F}$ 280.3, *C*F$_3$), 127.2 (2×*C*H-Ar), 127.7 (2×*C*H-Ar), 127.9 (2×*C*H-Ar), 128.7 (3×*C*H-Ar), 128.8 (2×*C*H-Ar), 129.4 (*C*H-Ar), 129.7 (*C*H-Ar), 129.7 (*C*-Ar), 132.8 (*C*-Ar), 135.4 (*C*-Ar), 137.7 (*C*-Ar), 143.2 (*C*-Ar), 162.6 (d, ${}^1J_{C\text{-}F}$ 246.9, F-*C*-Ar), 168.8 (*C*ON), 176.3 (*C*ONHBn); **HRMS (NSI$^+$)** exact mass calcd. For $C_{32}H_{27}F_4N_2O_2^+$ (M+H$^+$) requires m/z 547.2003, found m/z 547.1990 (–2.3 ppm).

(*S*)-*N*-benzyl-3-((*R*)-1-benzyl-3-(3-fluorophenyl)-2-oxoindolin-3-yl)-4,4,4-trifluorobutanamide (67)

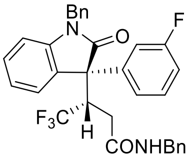

Following General Procedure **9**, 1-benzyl-3-(4-fluorophenyl)indolin-2-one (63.5 mg, 0.2 mmol), 4-nitrophenyl (*E*)-4,4,4-trifluorobut-2-enoate (52.2 mg, 0.2 mmol), (2*S*,3*R*)-HyperBTM (6.2 mg, 0.02 mmol) and DIPEA (35.0 μL, 0.2 mmol) in anhydrous THF (2.0 mL) at room temperature for 48 h. Benzyl amine (109 μL, 1.0 mmol) was added and the reaction was stirred at room temperature overnight. The crude residue (>95:5 dr) was purified by flash silica chromatography (95:5 Petrol : EtOAc, R$_f$ 0.15) to give desired compound (93.0 mg, 85%) as a white solid. mp: 88–90℃; $[\alpha]_D^{20}$ = +198.2 (*c* 0.5, CHCl$_3$); Chiral HPLC analysis, Chiralcel OD-H (90:10 hexane : IPA, flow rate 1 mL/min^{-1}, 254 nm, 30 °C) t$_R$

(major): 19.2 min, t_R (minor): 26.1 min, 98:2 er; ν_{max} (film), 3276 (N-H), 1712 (C=O), 1703 (C=O), 1182 (C-F), 1155 (C-F), 1115 (C-F); **^{1}HNMR ($CDCl_3$, 400 MHz)** δ_H: 2.55–2.69 (2H, m, C*H*$_2$CO), 4.32 (1H, dd, *J* 14.6, 5.8, NHC*H*$_2$Ph), 4.47 (1H, dd, *J* 14.6, 5.8, NHC*H*$_2$Ph), 4.55–4.63 (1H, m , C*H*CF$_3$), 4.85 (1H, d, *J* 16.0, C*H*$_2$Ph), 4.89 (1H, d, *J* 16.0, C*H*$_2$Ph), 5.81 (1H, t, *J* 5.5, N*H*CH$_2$Ph), 6.85 (1H, d, *J* 7.8, C*H*-Ar), 6.99–7.37 (16H, m, C*H*-Ar), 7.42 (1H, d, *J* 7.8, C*H*-Ar); **^{19}F{^{1}H} NMR (376 MHz, $CDCl_3$)** δ_F: –65.6 (s, C*F$_3$*), –111.1 (s, *F*-Ph); **^{13}C NMR ($CDCl_3$, 100 MHz):** δ_C, 32.8 (*C*H$_2$CO), 44.0 (*C*H$_2$Ph), 44.4 (*C*H$_2$Ph), 45.9 (q, $^2J_{C\text{-}F}$ 24.9, *C*HCF$_3$), 55.7 (*C*(3)), 110.4 (*C*H-Ar), 115.1 (d, $^2J_{C\text{-}F}$ 23.6, *C*H-Ar), 115.4 (d, $^2J_{C\text{-}F}$ 20.8, *C*H-Ar), 122.5 (*C*H-Ar), 123.6 (*C*H-Ar), 123.7 (*C*H-Ar), 126.2 (*C*H-Ar), 126.7 (q, $^1J_{C\text{-}F}$ 280.6, *C*F$_3$), 126.9 (*C*H-Ar), 127.2 (2×*C*H-Ar), 127.7 (2×*C*H-Ar), 127.9 (2×*C*H-Ar), 128.7 (2×*C*H-Ar), 128.8 (2×*C*H-Ar), 129.5 (*C*H-Ar), 130.6 (d, ^3J 7.1, *C*H-Ar), 135.3 (*C*-Ar), 137.7 (*C*-Ar), 139.7 (d, ^3J 7.1, *C*-Ar), 143.1 (*C*-Ar), 162.9 (d, $^1J_{C\text{-}F}$ 245.2, F-*C*-Ar), 168.8 (*C*ON), 175.9 (*C*ONHBn); **HRMS (NSI$^+$)** exact mass calcd. For $C_{32}H_{27}F_4N_2O_2^+$ (M+H$^+$) requires m/z 547.2003, found m/z 547.1989 (–2.6 ppm).

(*S*)-*N*-benzyl-3-((*R*)-1-benzyl-3-(3-methoxyphenyl)-2-oxoindolin-3-yl)-4,4,4-trifluorobutanamide (68)

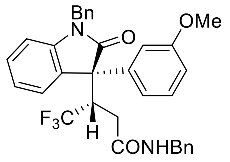

Following General Procedure **9**, 1-benzyl-3-(3-methoxyphenyl)indolin-2-one (65.9 mg, 0.2 mmol), 4-nitrophenyl (*E*)-4,4,4-trifluorobut-2-enoate (52.2 mg, 0.2 mmol), (2*S*,3*R*)-HyperBTM (6.2 mg, 0.02 mmol) and DIPEA (35.0 µL, 0.2 mmol) in anhydrous THF (2.0 mL) at room temperature for 48 h. Benzyl amine (109 µL, 1.0 mmol) was added and the reaction was stirred at room temperature overnight. The crude residue (>95:5 dr) was purified by flash silica chromatography (95:5 Petrol : EtOAc, R_f 0.14) to give desired compound (82.0 mg, 73%) as a white solid. mp: 79–80°C; $[\alpha]_D^{20}$ = +215.4 (*c* 0.5, $CHCl_3$); Chiral HPLC analysis, Chiralcel OD-H (90:10 hexane : IPA, flow rate 1 mL/min^{-1}, 254 nm, 30 °C) t_R (major): 20.3 min, t_R (minor): 27.5 min, 99:1 er; ν_{max} (film), 3338 (N-H), 1699 (C=O), 1648 (C=O), 1182 (C-F), 1152 (C-F), 1115 (C-F); **^{1}HNMR ($CDCl_3$, 400 MHz)** δ_H: 2.59–2.67 (2H, m, C*H*$_2$CO), 3.81 (3H, s, C*H*$_3$O), 4.31 (1H, dd, *J* 14.6, 5.8, NHC*H*$_2$Ph), 4.47 (1H, dd, *J* 14.6, 5.8, NHC*H*$_2$Ph), 4.59–4.64 (1H, m , C*H*CF$_3$), 4.87 (2H, s, C*H*$_2$Ph), 4.84 (1H, d, *J* 15.9, C*H*$_2$Ph), 4.90 (1H, d, *J* 15.8, C*H*$_2$Ph), 5.70 (1H, t, *J* 5.4, N*H*CH$_2$Ph), 6.82 (1H, d, *J* 7.8, C*H*-Ar), 6.84–6.87 (1H, m, C*H*-Ar), 6.95 (1H, d, *J* 7.4, CH-Ar), 7.08–7.12 (2H, m, C*H*-Ar), 7.19–7.35 (11H, m, C*H*-Ar); **^{19}F{^{1}H} NMR (376 MHz, $CDCl_3$)** δ_F: –65.7 (s, C*F$_3$*); **^{13}C NMR ($CDCl_3$, 100 MHz)** δ_C:, 32.9 (*C*H$_2$CO), 44.0 (*C*H$_2$Ph), 44.3 (*C*H$_2$Ph), 45.8 (q, $J_{C\text{-}F}$ 24.5, *C*HCF$_3$), 55.3 (*C*(3)), 55.8 (*C*H$_3$O), 110.2 (*C*H-Ar), 113.3 (CH-Ar), 114.3 (*C*H-Ar), 119.9 (2×CH-Ar), 122.3 (2×*C*H-Ar), 126.2 (*C*H-Ar), 126.7 (q, $^1J_{C\text{-}F}$ 280.3, *C*F$_3$), 127.2 (2×*C*H-Ar), 127.4 (*C*H-Ar), 127.6 (2×*C*H-Ar), 127.8 (2×*C*H-Ar), 128.7 (2×*C*H-Ar), 129.2 (*C*H-Ar), 129.8 (*C*H-Ar), 135.5 (*C*-Ar), 137.8 (*C*-Ar), 138.7 (C-Ar), 143.2 (*C*-Ar), 159.9 (MeO*C*-Ar), 169.0 (*C*ON), 176.3 (*C*ONHBn); **HRMS (NSI$^+$)** exact mass calcd. For $C_{33}H_{30}F_3N_2O_3^+$ (M+H$^+$) requires m/z 559.2203, found m/z 559.2193 (–1.8 ppm).

(*S*)-*N*-benzyl-3-((*R*)-1-benzyl-2-oxo-3-(thiophen-2-yl)indolin-3-yl)-4,4,4-trifluorobutanamide (69)

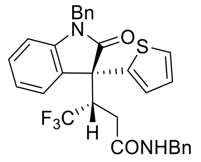

Following General Procedure **9**, 1-benzyl-3-(thiophen-2-yl)indolin-2-one (59.9 mg, 0.2 mmol), 4-nitrophenyl (*E*)-4,4,4-trifluorobut-2-enoate (52.2 mg, 0.2 mmol), (2*S*,3*R*)-HyperBTM (6.2 mg, 0.02 mmol) and DIPEA (35.0 µL, 0.2 mmol) in anhydrous THF (2.0 mL) at room temperature for 48 h. Benzyl amine (109 µL, 1.0 mmol) was added and the reaction was stirred at room temperature overnight. The crude residue (>95:5 dr) was purified by flash silica chromatography (5:1 Petrol : EtOAc, R_f 0.14) to give desired compound (91.0 mg, 85%) as a white solid. mp: 136–138°C; $[\alpha]_D^{20}$ = +215.0 (*c* 0.5, $CHCl_3$); Chiral HPLC analysis, Chiralcel OD-H (93:7 hexane : IPA, flow rate 1 mL/min^{-1}, 254 nm, 30 °C) t_R (major): 45.2 min, t_R (minor): 57.4 min, 98:2 er; ν_{max} (film), 3280 (N-H), 1709 (C=O), 1647 (C=O), 1182 (C-F), 1151 (C-F), 1113 (C-F); **^{1}HNMR ($CDCl_3$, 400 MHz)** δ_H: 2.66–2.82 (2H, m, CH_2CO), 4.33 (1H, dd, *J* 14.6, 5.4, NHCH_2Ph), 4.45–4.50 (2H, m, NHCH_2Ph, C*H*CF$_3$), 4.85 (1H, d, *J* 15.8, CH_2Ph), 4.94 (1H, d, *J* 15.8, CH_2Ph), 5.87 (1H, t, *J* 5.4, N*H*CH$_2$Ph), 6.81 (1H, d, *J* 7.8, C*H*-Ar), 7.00 (1H, dd, *J* 5.2, 3.7, C*H*-Ar), 7.10–7.12 (1H, m, C*H*-Ar), 7.20–7.36 (13H, m, C*H*-Ar), 7.41 (1H, d, *J* 7.1, C*H*-Ar); **^{19}F{^{1}H} NMR (376 MHz, $CDCl_3$)** δ_F: –65.8 (s, CF_3); **^{13}C NMR ($CDCl_3$, 100 MHz)** δ_C: 32.8 (CH$_2$CO), 44.0 (CH$_2$Ph), 44.4 (CH$_2$Ph), 47.1 (q, $^2J_{C-F}$ 24.9, *C*HCF$_3$), 53.6 (*C*(3)), 110.2 (*C*H-Ar), 122.4 (*C*H-Ar), 125.6 (*C*H-Ar), 126.6 (q, $^1J_{C-F}$ 280.8, *C*F$_3$), 126.3 (*C*H-Ar), 127.2 (2×*C*H-Ar), 127.6 (*C*H-Ar), 127.7 (3×*C*H-Ar), 127.9 (2×*C*H-Ar), 128.0 (*C*H-Ar), 128.7 (2×*C*H-Ar), 128.8 (2×*C*H-Ar), 129.6 (*C*-Ar), 135.3 (*C*-Ar), 137.8 (*C*-Ar), 141.0 (*C*-Ar), 142.9 (*C*-Ar), 169.0 (*C*ON), 175.5 (*C*ONHBn); **HRMS (NSI$^+$)** exact mass calcd. For $C_{30}H_{25}F_3N_2NaO_2S^+$ (M+Na$^+$) requires m/z 535.1481, found m/z 535.1468 (–2.2 ppm)

(*S*)-*N*-benzyl-3-((*R*)-1-benzyl-2-oxo-3-phenylindolin-3-yl)-4,4-difluorobutanamide (76)

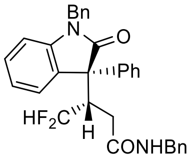

Following General Procedure **9**, 1-benzyl-3-phenylindolin-2-one (59.9 mg, 0.2 mmol), 4-nitrophenyl 4-nitrophenyl (*E*)-4,4-difluorobut-2-enoate (48.6 mg, 0.2 mmol), (2*S*,3*R*)-HyperBTM (6.2 mg, 0.02 mmol) and DIPEA (35.0 µL, 0.2 mmol) in anhydrous THF (2.0 mL) at room temperature for 48 h. Benzyl amine (109 µL, 1.0 mmol) was added and the reaction was stirred at room temperature overnight. The crude residue (>95:5 dr) was purified by flash silica chromatography (5:1 Petrol : EtOAc, R_f 0.15) to give desired compound (68.0 mg, 67%) as a white solid. mp: 61–63°C; $[\alpha]_D^{20}$ = +132.1 (*c* 0.6, $CHCl_3$); Chiral HPLC analysis, Chiralcel IC (80:20 hexane : IPA, flow rate 1.5 mL/min^{-1}, 254 nm, 40 °C) t_R (major): 34.6 min, t_R (minor): 41.7 min, 93:7 er; ν_{max} (film) , 2862 (C-H), 1748 (C=O), 1734 (C=O); **^{1}HNMR ($CDCl_3$, 400 MHz)** δ_H: 2.37–2.52 (2H, m, CH_2CO), 4.11–4.22 (1H, m, C*H*CHF$_2$), 4.30 (1H, dd, *J* 14.6, 5.8, NHCH_2Ph), 4.45 (1H, dd, *J* 14.6, 5.8, NHCH_2Ph), 4.91 (2H, s, CH_2Ph), 5.60–5.89 (2H, m, N*H*CH$_2$Ph, CHC*H*F$_2$), 6.84 (1H, d, *J* 7.8, C*H*-Ar), 7.09–7.13 (1H, m, C*H*-Ar), 7.21–7.38 (15H, m, C*H*-Ar), 7.47–7.49 (2H, m, C*H*-Ar) ppm; **^{19}F{^{1}H} NMR (376 MHz, $CDCl_3$)** δ_F: –114.6 (d, *J* 284.9, CHCHF_2), -121.2 (d, *J* 284.5 Hz, CHCHF_2); **^{13}C NMR ($CDCl_3$, 100 MHz)** δ_C: 32.2 (CH$_2$CO), 43.9 (CH$_2$Ph), 44.2 (CH$_2$Ph), 45.7 (t, $^2J_{C-F}$ 19.3, *C*HCHF$_2$), 56.4 (*C*(3)), 110.0 (*C*H-Ar), 116.9 (t, $^1J_{C-F}$ 243.2, *C*HF$_2$), 122.6 (*C*H-Ar), 125.9 (*C*H-Ar), 127.3 (2×*C*H-Ar), 127.5 (2×*C*H-Ar), 127.6 (*C*H-Ar), 127.7 (*C*H-Ar), 127.8 (2×*C*H-Ar), 128.1 (*C*H-Ar), 128.6 (*C*H-Ar), 128.7 (2×*C*H-Ar), 128.7 (2×*C*H-Ar), 129.0 (2×*C*H-Ar), 129.0 (*C*-Ar), 135.6 (*C*-Ar), 137.6 (*C*-Ar), 137.9 (*C*-Ar), 143.0 (*C*-Ar), 169.9 (*C*ON), 176.8 (*C*ONHBn); **HRMS (NSI$^+$)** exact mass calcd. For $C_{32}H_{29}F_2N_2O_2^+$ (M+H$^+$) requires m/z 511.2192, found m/z 511.2182 (–1.9 ppm).

(*S*)-*N*-benzyl-3-((*R*)-1-benzyl-2-oxo-3-phenylindolin-3-yl)-4-chloro-4,4-difluorobutanamide (77)

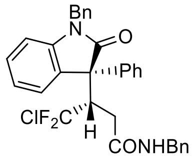

Following General Procedure **9**, 1-benzyl-3-phenylindolin-2-one (59.9 mg, 0.2 mmol), 4-nitrophenyl (*E*)-4-chloro-4,4-difluorobut-2-enoate (55.4 mg, 0.2 mmol), (2*S*,3*R*)-HyperBTM (6.2 mg, 0.02 mmol) and DIPEA (35.0 µL, 0.2 mmol) in anhydrous THF (2.0 mL) at room temperature for 48 h. Benzyl amine (109 µL, 1.0 mmol) was added and the reaction was stirred at room temperature overnight. The crude residue (95:5 dr) was purified by flash silica chromatography (5:1 Petrol : EtOAc, R_f 0.14) to give desired compound (92.0 mg, 84%) as a white solid. mp: 78–79°C; $[\alpha]_D^{20}$ = +138.3 (*c* 1.6, $CHCl_3$); Chiral HPLC analysis, Chiralcel OD-H (93:7 hexane : IPA, flow rate 1.0 mL/min^{-1}, 254 nm, 30 °C) t_R (major): 34.4 min, t_R (minor): 47.4 min, 98:2 er; ν_{max} (film), 3316 (N-H), 1701 (C=O), 1655 (C=O), 1192 (C-F), 959 (C-Cl); **^{1}HNMR ($CDCl_3$, 400 MHz)** δ_H: 2.64–2.77 (2H, m, C*H*$_2$CO), 4.11–4.22 (1H, m, C*H*CHF_2), 4.25 (1H, dd, *J* 14.6, 5.3, NHC*H*$_2$Ph), 4.44 (1H, dd, *J* 14.6, 5.9, NHC*H*$_2$Ph), 4.78–4.90 (3H, m, C*H*$_2$Ph, C*H*CF_2Cl), 5.72 (1H, t, *J* 5.4, N*H*CH_2Ph), 6.82 (1H, d, *J* 7.8, C*H*-Ar), 7.09–7.13 (1H, m, C*H*-Ar), 7.16–7.21 (4H, m, C*H*-Ar), 7.23–7.35 (11H, m, C*H*-Ar), 7.49–7.54 (2H, m, C*H*-Ar); **^{19}F{^{1}H} NMR (376 MHz, $CDCl_3$)** δ_F: –49.3 (d, *J* 163.0, CHCCl*F*$_2$), –51.9 (d, *J* 163.0, CHCCl*F*$_2$); **^{13}C NMR ($CDCl_3$, 100 MHz)** δ_C: 34.3 (*C*H_2CO), 44.1 (*C*H_2Ph), 44.3 (*C*H_2Ph), 51.8 (t, $^2J_{C\text{-}F}$ 20.5, *C*HF_2Cl), 56.8 (*C*(3)), 110.2 (*C*H-Ar), 122.2 (*C*H-Ar), 126.7 (*C*H-Ar), 127.2 (3×*C*H-Ar), 127.6 (2×*C*H-Ar), 127.9 (2×*C*H-Ar), 128.0 (2×*C*H-Ar), 128.4 (*C*H-Ar), 128.7 (4×*C*H-Ar), 128.9 (2×*C*H-Ar), 129.1 (*C*-Ar), 130.3 (t, $^1J_{C\text{-}F}$ 296.1, *C*ClF_2), 135.5 (*C*-Ar), 137.4 (*C*-Ar), 137.8 (*C*-Ar), 143.2 (*C*-Ar), 169.1 (*C*ON), 176.6 (*C*ONHBn); **HRMS (NSI$^+$)** exact mass calcd. For $C_{32}H_{28}ClF_2N_2O_2^+$ (M+H$^+$) requires m/z 545.1802, found m/z 545.1792 (–1.8 ppm).

(*S*)-*N*-benzyl-3-((*R*)-1-benzyl-2-oxo-3-phenylindolin-3-yl)-4-bromo-4,4-difluorobutanamide (78)

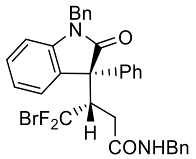

Following General Procedure **9**, 1-benzyl-3-phenylindolin-2-one (59.9 mg, 0.2 mmol), 4-nitrophenyl (*E*)-4-bromo-4,4-difluorobut-2-enoate (64.4 mg, 0.2 mmol), (2*S*,3*R*)-HyperBTM (6.2 mg, 0.02 mmol) and DIPEA (35.0 µL, 0.2 mmol) in anhydrous THF (2.0 mL) at room temperature for 48 h. Benzyl amine (109 µL, 1.0 mmol) was added and the reaction was stirred at room temperature overnight. The crude residue (95:5 dr) was purified by flash silica chromatography (5:1 Petrol : EtOAc, R_f 0.15) to give desired compound (99.0 mg, 84%) as a white solid. mp: 86–87°C; $[\alpha]_D^{20}$ = +170.1 (*c* 1.6, $CHCl_3$); Chiral HPLC analysis, Chiralcel OD-H (93:7 hexane : IPA, flow rate 1.0 mL/min^{-1}, 254 nm, 30 °C) t_R (major): 35.7 min, t_R (minor): 47.5 min, 99:1 er; ν_{max} (film), 3312 (N-H), 1699 (C=O), 1653 (C=O), 1190 (C-F), 889 (C-Br); **^{1}HNMR ($CDCl_3$, 400 MHz):** δ_H 2.63–2.78 (2H, m, C*H*$_2$CO), 4.25 (1H, dd, *J* 14.5, 5.2, NHC*H*$_2$Ph), 4.44 (1H, dd, *J* 14.5, 5.8, NHC*H*$_2$Ph), 4.82–4.91 (3H, m, C*H*$_2$Ph, C*H*CF_2Br), 5.57 (1H, t, *J* 5.4, N*H*CH_2Ph), 6.81 (1H, d, *J* 7.8, C*H*-Ar), 7.09–7.13 (1H, m, C*H*-Ar), 7.15–7.34 (15H, m, C*H*-Ar), 7.51 (2H, d, *J* 5.5, C*H*-Ar); **^{19}F{^{1}H} NMR (376 MHz, $CDCl_3$)** δ_F: –42.75 (d, *J* 158.7, CHC*F*$_2$Br), -45.67 (d, *J* 159.2, CHC*F*$_2$Br); **^{13}C NMR ($CDCl_3$, 100 MHz):** δ_C, 34.7 (*C*H_2CO), 44.1 (*C*H_2Ph), 44.3 (*C*H_2Ph),

53.4 (t, $^2J_{C-F}$ 20.5, *C*HF$_2$Br), 56.9 (*C*(3)), 110.2 (*C*H-Ar), 123.3 (t, $^1J_{C-F}$ 308.4, *C*BrF$_2$), 122.1 (*C*H-Ar), 126.8 (*C*H-Ar), 127.1 (*C*H-Ar), 127.2 (2×*C*H-Ar), 127.6 (2×*C*H-Ar), 128.0 (4×*C*H-Ar), 128.4 (*C*H-Ar), 128.7 (4×*C*H-Ar), 128.9 (2×*C*H-Ar), 129.1 (*C*-Ar), 135.5 (*C*-Ar), 137.3 (*C*-Ar), 137.7 (*C*-Ar), 143.2 (*C*-Ar), 169.1 (*C*ON), 176.6 (*C*ONHBn); **HRMS (NSI$^+$)** exact mass calcd. For $C_{32}H_{28}BrF_2N_2O_2^+$ (M+H$^+$) requires m/z 589.1297, found m/z 589.1282 (–2.6 ppm).

(*S*)-*N*-benzyl-3-((*R*)-1-benzyl-2-oxo-3-phenylindolin-3-yl)butanamide (79)

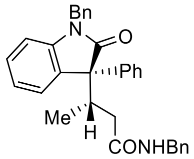

Following General Procedure **9**, 1-benzyl-3-phenylindolin-2-one (59.9 mg, 0.2 mmol), 4-nitrophenyl (E)-but-2-enoate (41.4 mg, 0.2 mmol), (2*S*,3*R*)-HyperBTM (12.3 mg, 0.04 mmol) and DIPEA (35.0 µL, 0.2 mmol) in anhydrous THF (2.0 mL) at room temperature for 72 h. Benzyl amine (109 µL, 1.0 mmol) was added and the reaction was stirred at room temperature overnight. The crude residue (86:14 dr$_{\mathbf{5ah:5ah'}}$) was purified by flash silica chromatography (4:1 Petrol : EtOAc, R$_f$ 0.14) to give desired compound of a mixture of isomers (81.0 mg, 85%) as a white solid. mp: 123–124°C; $[\alpha]_D^{20}$ = +29.6 (*c* 0.6, CHCl$_3$); Chiral HPLC analysis, Chiralcel OD-H (93:7 hexane : IPA, flow rate 1.0 mL/min^{-1}, 254 nm, 30 °C) t$_R$ 51.8, 66.3 min, 86:14 er; νmax (film), 2862 (C-H), 1748 (C=O), 1734 (C=O); **^{1}HNMR (CDCl$_3$, 400 MHz)** δ$_H$: 0.85 (3H, d , *J* 6.6, C*H*$_3$), 2.02 (1H, dd, *J* 13.9, 11.3, C*H*$_2$CO), 3.44-3.51 (1H, m, C*H*), 4.40 (2H, dd, *J* 8.9, 5.7, NHC*H*$_2$Ph), 4.89 (1H, d, *J* 15.6, NC*H*$_2$Ph), 4.96 (1H, d, *J* 15.6, NC*H*$_2$Ph), 5.62 (1H, t, *J* 5.4, N*H*CH$_2$Ph), 5.74 (1H, t, *J* 5.5, N*H*CH$_2$Ph), 6.84 (1H, d, *J* 7.8, C*H*-Ar), 7.08-7.12 (1H, m, C*H*-Ar), 7.23–7.37 (15H, m, C*H*-Ar), 7.51–7.53 (2H, m, C*H*-Ar); **^{13}C NMR (CDCl$_3$, 100 MHz)** δ$_C$: 15.1 (*C*H$_3$), 38.3 (*C*H), 39.2 (*C*H$_2$CO), 43.7 (*C*H$_2$Ph), 44.0 (*C*H$_2$Ph), 60.3 (*C*(3)), 109.5 (*C*H-Ar), 122.4 (*C*H-Ar), 125.7 (*C*H-Ar), 127.4 (2×*C*H-Ar), 127.5 (2×*C*H-Ar), 127.6 (2×*C*H-Ar), 127.8 (2×*C*H-Ar), 128.4 (*C*H-Ar), 128.7 (2×*C*H-Ar), 128.8 (4×*C*H-Ar), 129.7 (*C*-Ar), 135.9 (*C*-Ar), 138.2 (*C*-Ar), 138.7 (*C*-Ar), 143.4 (*C*-Ar), 171.3 (*C*ON), 177.7 (*C*ONHBn), 177.5 (*C*ONHBn); **HRMS (NSI$^+$)** exact mass calcd. For $C_{32}H_{31}N_2O_2^+$ (M+H$^+$) requires m/z 475.2380, found m/z 475.2373 (–1.5 ppm).

(*R*)-*N*-benzyl-3-((*R*)-1-benzyl-2-oxo-3-phenylindolin-3-yl)-3-phenylpropanamide (80major) and (*S*)-*N*-benzyl-3-((*R*)-1-benzyl-2-oxo-3-phenylindolin-3-yl)-3-phenylpropanamide (80minor)

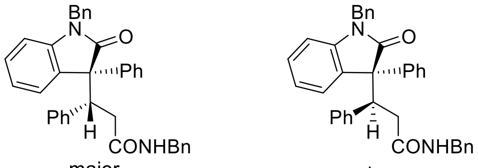

major minor

Following General Procedure **9**, 1-benzyl-3-phenylindolin-2-one (59.9 mg, 0.2 mmol), 4-nitrophenyl cinnamate (53.8 mg, 0.2 mmol), (2*S*,3*R*)-HyperBTM (12.3 mg, 0.04 mmol) and DIPEA (35.0 µL, 0.2 mmol) in anhydrous THF (2.0 mL) at room temperature for 72 h. Benzyl amine (109 µL, 1.0 mmol) was added and the reaction was stirred at room temperature overnight. The crude residue (50:50 dr) was purified by flash silica chromatography (4:1 Petrol : EtOAc, R$_f$ 0.12) to give desired compound of a mixture of isomers (87.0 mg, 81%) as a white solid. mp: 123–124°C; $[\alpha]_D^{20}$ = +29.6 (*c* 0.6, CHCl$_3$).

80$_{major}$: Chiral HPLC analysis, Chiralcel IC (90:10 hexane : IPA, flow rate 0.5 mL/min^{-1}, 254 nm, 40 °C) t$_R$ 49.1 min, 128.1, 99:1 er; ν$_{max}$ (film) , 3325 (N-H), 1701 (C=O), 1647 (C=O); **^{1}HNMR (CDCl$_3$, 400**

MHz) δ_H: 2.77–2.92 (1H, m, C*H*$_2$CO), 3.23 (1H, dd, *J* 15.0, 11.5, C*H*$_2$CO), 4.25–4.38 (2H, m, NHC*H*$_2$Ph), 4.57 (1H, dd, *J* 11.3, 3.4, C*H*Ph), 4.81 (1H, d, *J* 16.0, NC*H*$_2$Ph), 4.94 (1H, d, *J* 15.8, NC*H*$_2$Ph), 5.74 (1H, t, *J* 5.5, N*H*CH$_2$Ph), 6.43 (2H, d, *J* 7.3, C*H*-Ar), 6.80 (2H, dd, *J* 6.6, 1.6, C*H*-Ar), 6.94-7.29 (14H, m, C*H*-Ar), 7.35-7.36 (1H, m, C*H*-Ar), 7.39-7.43 (2H, m, C*H*-Ar), 7.54-7.56 (1H, m, C*H*-Ar), 7.69-7.73 (2H, m, C*H*-Ar); **^{13}C NMR (CDCl$_3$, 100 MHz)** δ_C: 38.5 (*C*H$_2$CO), 43.2 (*C*H$_2$Ph), 43.8 (*C*H$_2$Ph), 49.6 (*C*HPh), 60.9 (*C*(3)), 110.0 (*C*H-Ar), 122.6 (*C*H-Ar), 124.5 (*C*H-Ar), 126.5 (*C*H-Ar), 126.9 (*C*H-Ar), 127.0 (2×*C*H-Ar), 127.0 (2×*C*H-Ar), 127.2 (2×*C*H-Ar), 127.5 (*C*H-Ar), 127.9 (2×*C*H-Ar), 128.4 (2×*C*H-Ar), 128.5 (2×*C*H-Ar), 128.7 (2×*C*H-Ar), 128.9 (*C*H-Ar), 129.1 (2×*C*H-Ar), 129.6 (*C*H-Ar), 129.6 (*C*-Ar), 135.1 (*C*-Ar), 137.8 (*C*-Ar), 137.9 (*C*-Ar), 139.3 (*C*-Ar), 144.0 (*C*-Ar), 171.5(*C*ON), 177.5 (*C*ONHBn); **HRMS (NSI$^+$)** exact mass calcd. For $C_{37}H_{33}N_2O_2^+$ (M+H$^+$) requires m/z 537.2537, found m/z 537.2527 (–1.8 ppm).

80$_{minor}$: Chiral HPLC analysis, Chiralcel IC (90:10 hexane : IPA, flow rate 0.5 mL/min^{-1}, 254 nm, 40 °C) t_R 173.9 min, 185.9, 79:21 er; ν_{max} (film) , 3304 (N-H), 1701 (C=O), 1647 (C=O); **^{1}HNMR (CDCl$_3$, 400 MHz)** δ_H: 2.77–2.92 (2H, m, C*H*$_2$CO), 3.23 (1H, dd, *J* 15.0, 11.5, C*H*$_2$CO), 4.01 (1H, dd, *J* 15.0, 4.9, NHC*H*$_2$Ph), 4.09 (1H, dd, *J* 14.9, 5.0, NHC*H*$_2$Ph), 4.25–4.38 (1H, m, C*H*Ph), 4.66-4.71 (2H, m, NC*H*$_2$Ph), 5.46 (1H, t, *J* 5.5, N*H*CH$_2$Ph), 6.36-6.40 (1H, m, C*H*-Ar), 6.47 (1H, d, *J* 7.2, C*H*-Ar), 6.75 (2H, dd, *J* 6.9, 1.4, C*H*-Ar), 6.94-7.29 (14H, m, C*H*-Ar), 7.30-7.32 (1H, m, C*H*-Ar), 7.39-7.43 (2H, m, C*H*-Ar), 7.46-7.48 (1H, m, C*H*-Ar), 7.69-7.73 (2H, m, C*H*-Ar); **^{13}C NMR (CDCl$_3$, 100 MHz)** δ_C: 36.8 (*C*H$_2$CO), 43.2 (*C*H$_2$Ph), 43.7 (*C*H$_2$Ph), 49.0 (*C*HPh), 60.8 (*C*(3)), 109.1 (*C*H-Ar), 122.0 (*C*H-Ar), 124.5 (*C*H-Ar), 126.5 (*C*H-Ar), 126.6 (*C*H-Ar), 127.0 (2×*C*H-Ar), 127.0 (2×*C*H-Ar), 127.2 (2×*C*H-Ar), 127.4 (*C*H-Ar), 127.7 (*C*H-Ar), 127.8 (2×*C*H-Ar), 128.3 (2×*C*H-Ar), 128.4 (2×*C*H-Ar), 128.7 (2×*C*H-Ar), 128.9 (2×*C*H-Ar), 129.4 (2×*C*H-Ar), 132.5 (*C*-Ar), 135.4 (*C*-Ar), 137.8 (*C*-Ar), 138.1 (*C*-Ar), 141.4 (*C*-Ar), 170.6 (*C*ON), 176.7 (*C*ONHBn); **HRMS (NSI$^+$)** exact mass calcd. For $C_{37}H_{33}N_2O_2^+$ (M+H$^+$) requires m/z 537.2537, found m/z 537.2527 (–1.8 ppm).

(*R*)-*N*-benzyl-3-((*R*)-1-benzyl-2-oxo-3-phenylindolin-3-yl)-3-(*p*-tolyl)propanamide (S3$_{major}$) and (*S*)-*N*-benzyl-3-((*R*)-1-benzyl-2-oxo-3-phenylindolin-3-yl)-3-(*p*-tolyl)propanamide (S3$_{minor}$)

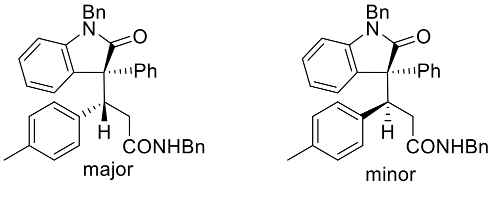

Following General Procedure **9**, 1-benzyl-3-phenylindolin-2-one (59.9 mg, 0.2 mmol), 4-nitrophenyl (*E*)-3-(*p*-tolyl)acrylate (56.7 mg, 0.2 mmol), (2*S*,3*R*)-HyperBTM (12.3 mg, 0.04 mmol) and DIPEA (35.0 µL, 0.2 mmol) in anhydrous THF (2.0 mL) at room temperature for 72 h. Benzyl amine (109 µL, 1.0 mmol) was added and the reaction was stirred at room temperature overnight. The crude residue (59:41 dr) was purified by flash silica chromatography (67:33 Petrol : EtOAc, R_f 0.21$_{major}$ and 0.29$_{minor}$) to give:

S3$_{major}$ (16.4 mg, 15%) as a white solid. mp: 172–173°C; $[\alpha]_D^{20}$ = +42.3 (*c* 0.8, CHCl$_3$); ν_{max} (film), 1711 (C=O), 1647 (C=O); **^{1}HNMR (CDCl$_3$, 400 MHz)** δ_H: 2.34 (3H, s, C*H*$_3$), 2.74–2.86 (2H, m, C*H*$_2$CO), 4.09 (1H, dd, *J* 5.1, 14.9, NHC*H*$_2$Ph), 4.24 (1H, d, *J* 16.1, NC*H*$_2$Ph), 4.34 (1H, dd, *J* 14.9, 5.0, NHC*H*$_2$Ph), 4.60 (1H, dd, *J* 3.3, 11.8, C*H*Ar),4.89 (1H, d, *J* 16.1, NC*H*$_2$Ph), 5.34 (1H, t, *J* 5.4, N*H*CH$_2$Ph), 6.45–6.49 (3H, m, C*H*-Ar), 6.75–6.77 (2H, m, C*H*-Ar), 6.80–6.82 (2H, m, C*H*-Ar), 6.89–6.91 (2H, m, C*H*-Ar), 7.06–7.09 (2H, m, C*H*-Ar), 7.14–7.24 (6H, m, C*H*-Ar), 7.34–7.36 (1H, m, C*H*-Ar), 7.39–7.43 (2H, m,

C*H*-Ar), 7.53–7.55 (1H, m, C*H*-Ar), 7.69–7.71 (1H, m, C*H*-Ar); **^{13}C NMR (CDCl$_3$, 100 MHz)** δ_C: 21.3 (*C*H$_3$), 38.6 (*C*H$_2$CO), 43.3 (*C*H$_2$Ph), 43.8 (*C*H$_2$Ph), 49.2 (*C*HAr), 60.9 (*C*(3)), 109.9 (*C*H-Ar), 121.9 (*C*H-Ar), 126.6 (2×*C*H-Ar), 127.0 (*C*H-Ar), 127.2 (*C*H-Ar), 127.3 (*C*H-Ar), 127.8 (*C*H-Ar), 127.9 (2×*C*H-Ar), 128.36 (2×*C*H-Ar), 128.40 (2×*C*H-Ar), 128.8 (*C*H-Ar), 128.9 (2×*C*H-Ar), 129.0 (2×*C*H-Ar), 129.4 (2×*C*H-Ar), 134.7 (*C*-Ar), 135.1 (*C*-Ar), 136.9 (*C*-Ar), 137.8 (*C*-Ar), 137.9 (*C*-Ar), 144.0 (*C*-Ar), 170.7 (*C*ON), 176.7 (*C*ONHBn); **HRMS (NSI$^+$)** exact mass calcd. For $C_{38}H_{34}N_2NaO_2^+$ (M+Na$^+$) requires m/z 537.2512, found m/z 537.2502 (–1.8 ppm).

S3$_{minor}$ (12.1 mg, 11%) as a white solid. mp: 172–173°C; $[\alpha]_D^{20}$ = +126.0 (*c* 0.1, CHCl$_3$); ν_{max} (film), 1690 (C=O), 1647 (C=O); **^{1}HNMR (CDCl$_3$, 400 MHz)** δ_H: 2.25 (3H, s, C*H*$_3$), 2.83 (1H, dd, *J* 3.5, 14.8 Hz, C*H*$_2$CO), 3.19 (1H, dd, *J* 11.6, 14.8, C*H*$_2$CO), 4.03 (1H, dd, *J* 4.9, 15.1 Hz, NHC*H*$_2$Ph), 4.37 (1H, dd, *J* 6.7, 15.1 Hz, NHC*H*$_2$Ph), 4.52 (1H, dd, *J* 3.5, 11.6, C*H*Ar), 4.77 (1H, d, *J* 15.9, NC*H*$_2$Ph), 4.89 (1H, d, *J* 15.9, NC*H*$_2$Ph), 5.66 (1H, br, N*H*CH$_2$Ph), 6.38–6.40 (1H, m, C*H*-Ar), 6.79–6.83 (4H, m, C*H*-Ar), 6.97–7.04 (5H, m, C*H*-Ar), 7.15–7.19 (3H, m, C*H*-Ar), 7.24–7.31 (5H, m, C*H*-Ar), 7.38–7.42 (2H, m, C*H*-Ar), 7.45–7.47 (1H, m, C*H*-Ar), 7.67–7.69 (2H, m, C*H*-Ar); **^{13}C NMR (CDCl$_3$, 100 MHz)** δ_C: 21.1 (*C*H$_3$), 36.9 (*C*H$_2$CO), 43.2 (*C*H$_2$Ph), 43.7 (*C*H$_2$Ph), 48.6 (*C*HAr), 60.9 (*C*(3)), 109.1 (*C*H-Ar), 122.5 (*C*H-Ar), 124.5 (*C*H-Ar), 127.03 (2×*C*H-Ar), 127.05 (*C*H-Ar), 127.1 (2×*C*H-Ar), 127.2 (2×*C*H-Ar), 127.3 (*C*H-Ar), 127.4 (*C*H-Ar), 127.6 (*C*H-Ar), 128.4 (2×*C*H-Ar), 128.5 (2×*C*H-Ar), 128.6 (2×*C*H-Ar), 129.1 (2×*C*H-Ar), 129.2 (2×*C*H-Ar), 132.5 (*C*-Ar), 134.6 (*C*-Ar), 135.4 (*C*-Ar), 136.3 (*C*-Ar), 138.1 (*C*-Ar), 139.4 (*C*-Ar), 141.5 (*C*-Ar), 171.6(*C*ON), 177.6 (*C*ONHBn); **HRMS (NSI$^+$)** exact mass calcd. For $C_{38}H_{34}N_2N\ aO_2^+$ (M+Na$^+$) requires m/z 537.2512, found m/z 537.2505 (–1.3 ppm).

(*R*)-*N*-benzyl-3-((*R*)-1-benzyl-2-oxo-3-phenylindolin-3-yl)-3-(4-nitrophenyl)propanamide (S4$_{major}$) and (*S*)-*N*-benzyl-3-((*R*)-1-benzyl-2-oxo-3-phenylindolin-3-yl)-3-(4-nitrophenyl)propanamide (S4$_{minor}$)

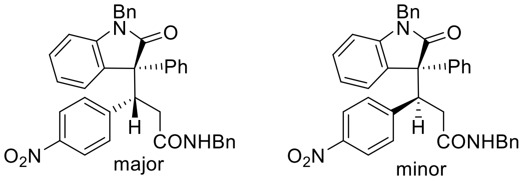

Following General Procedure **9**, 1-benzyl-3-phenylindolin-2-one (59.9 mg, 0.2 mmol), 4-nitrophenyl (*E*)-3-(4-nitrophenyl)acrylate (62.9 mg, 0.2 mmol), (2*S*,3*R*)-HyperBTM (12.3 mg, 0.04 mmol) and DIPEA (35.0 µL, 0.2 mmol) in anhydrous THF (2.0 mL) at room temperature for 72 h. Benzyl amine (109 µL, 1.0 mmol) was added and the reaction was stirred at room temperature overnight. The crude residue (52:48 dr) was purified by flash silica chromatography (67:33 Petrol : EtOAc, R_f 0.19$_{major}$ and 0.24$_{minor}$) to give:

S4$_{major}$ (31.8 mg, 30%) as a white solid. mp: 209–210°C; $[\alpha]_D^{20}$ = +93.6 (*c* 0.5, CHCl$_3$); ν_{max} (film), 1705 (C=O), 1647 (C=O); **^{1}HNMR (CDCl$_3$, 400 MHz)** δ_H: 2.72 (1H, dd, *J* 12.3, 14.3 Hz, C*H*$_2$CO), 2.86 (1H, dd, *J* 2.8, 14.3 Hz, C*H*$_2$CO), 4.10 (1H, dd, *J* 5.1, 14.7, NHC*H*$_2$Ph), 4.27–4.33 (2H, m, NC*H*$_2$Ph+NHC*H*$_2$Ph), 4.73–4.81 (2H, m, NC*H*$_2$Ph+ C*H*Ar), 5.42 (1H, br, N*H*CH$_2$Ph), 6.63–6.68 (3H, m, C*H*-Ar), 6.84–6.86 (2H, m, C*H*-Ar), 7.00–7.06 (4H, m, C*H*-Ar), 7.12–7.20 (4H, m, C*H*-Ar), 7.22–7.26 (1H, m, C*H*-Ar), 7.32–7.39 (2H, m, C*H*-Ar), 7.41–7.45(2H, m, C*H*-Ar); 7.74–7.75 (1H, m, C*H*-Ar), 7.67–7.69 (2H, m, C*H*-Ar), 7.79–7.81 (2H, m, C*H*-Ar); **^{13}C NMR (CDCl$_3$, 100 MHz)** δ_C: 37.8 (*C*H$_2$CO), 43.5 (*C*H$_2$Ph), 43.9 (*C*H$_2$Ph), 49.1 (*C*HPh), 60.2(*C*(3)), 110.0 (*C*H-Ar), 122.3 (*C*H-Ar), 123.0 (2×*C*H-

Ar), 126.6 (CH-Ar), 127.0 (2×CH-Ar), 127.5 (2× CH-Ar), 127.57 (CH-Ar), 127.62 (CH-Ar), 127.8 (2×CH-Ar), 128.0 (CH-Ar), 128.1 (CH-Ar), 128.4 (2×CH-Ar), 128.6 (2×CH-Ar), 129.1 (2×CH-Ar), 129.4(CH-Ar), 130.3 (2×CH-Ar); **HRMS (NSI$^+$)** exact mass calcd. For $C_{37}H_{31}N_3NaO_4^+$ (M+Na$^+$) requires m/z 604.2207, found m/z 604.2200 (–1.1 ppm).

S4$_{minor}$ (31.4 mg, 27%) as a white solid. mp: 192–193°C; $[\alpha]_D^{20}$ = +115.6 (*c* 0.1, $CHCl_3$); ν_{max} (film), 1697 (C=O), 1645 (C=O); **^{1}HNMR ($CDCl_3$, 400 MHz)** δ_H: 2.86 (1H, dd, *J* 3.6, 15.5, CH_2CO), 3.25 (1H, dd, *J* 11.4, 15.5, CH_2CO), 4.03 (1H, dd, *J* 5.0, 14.8, NHCH_2Ph), 4.32 (1H, dd, *J* 6.6, 14.8, NHCH_2Ph), 4.67 (1H, dd, *J* 3.6, 11.4, C*H*Ph), 4.75 (1H, d, *J* 15.5, NCH_2Ph), 4.87 (1H, d, *J* 15.5, NCH_2Ph), 5.71 (1H, br, *J* 5.5, N*H*CH$_2$Ph), 6.51–6.53 (1H, m, C*H*-Ar), 6.87–6.89 (2H, m, C*H*-Ar), 7.02–7.05 (2H, m, C*H*-Ar), 7.09–7.10 (2H, m, C*H*-Ar), 7.16–7.21 (4H, m, C*H*-Ar), 7.24–7.35 (5H, m, C*H*-Ar), 7.41–7.45 (2H, m, C*H*-Ar), 7.48–7.50 (1H, m, C*H*-Ar), 7.65–7.68 (2H, m, C*H*-Ar), 7.72–7.75 (2H, m, C*H*-Ar); **^{13}C NMR ($CDCl_3$, 100 MHz)** δ_C: 36.1 (CH$_2$CO), 43.4 (CH$_2$Ph), 43.8 (CH$_2$Ph), 48.6 (CHPh), 60.1 (C(3)), 109.3 (CH-Ar), 122.7 (2×CH-Ar), 122.9 (CH-Ar), 124.3 (CH-Ar), 126.9 (2×CH-Ar), 127.4 (2×CH-Ar), 127.5 (2×CH-Ar), 127.8 (CH-Ar), 127.9 (CH-Ar), 128.3 (CH-Ar), 128.5 (2×CH-Ar), 128.7 (2×CH-Ar), 129.3 (2×CH-Ar), 130.1 (2×CH-Ar), 131.7 (C-Ar), 135.1 (C-Ar), 137.8 (C-Ar), 138.5 (C-Ar), 141.2 (C-Ar), 145.9 (C-Ar), 146.7 (C-Ar), 170.6(CON), 176.9 (CONHBn); **HRMS (NSI$^+$)** exact mass calcd. For $C_{37}H_{31}N_3NaO_4^+$ (M+Na$^+$) requires m/z 604.2207, found m/z 604.2198 (–1.4 ppm).

Ethyl (*S*)-2-((*R*)-1-benzyl-2-oxo-3-phenylindolin-3-yl)-4-(benzylamino)-4-oxobutanoate (81$_{major}$) and ethyl (*R*)-2-((*R*)-1-benzyl-2-oxo-3-phenylindolin-3-yl)-4-(benzylamino)-4-oxobutanoate (81$_{minor}$)

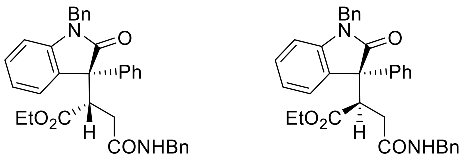

Following General Procedure **9**, 1-benzyl-3-phenylindolin-2-one (59.9 mg, 0.2 mmol), ethyl (4-nitrophenyl) fumarate (53.0 mg, 0.2 mmol), (2*S*,3*R*)-HyperBTM (6.2 mg, 0.02 mmol) and DIPEA (35.0 µL, 0.2 mmol) in anhydrous THF (2.0 mL) at –40 °C for 48 h. Benzyl amine (109 µL, 1.0 mmol) was added and the reaction was stirred at room temperature overnight. The crude residue (67:33 dr) was purified by flash silica chromatography (5:1 to 2:1 Petrol : EtOAc, R_f 0.16$_{major}$ and R_f 0.14$_{minor}$) to give:

81$_{major}$: (74.0 mg, 70%) as a white solid. mp: 83–85°C; $[\alpha]_D^{20}$ = +202.3 (*c* 0.8, $CHCl_3$); Chiral HPLC analysis, Chiralcel OD-H (85:15 hexane : IPA, flow rate 1 mL/min^{-1}, 254 nm, 30 °C) t_R (major): 23.5 min, t_R (minor): 31.1 min, 97:3 er; ν_{max} (film), 3304 (N-H), 1721 (C=O), 1705 (C=O), 1649 (C=O); **^{1}HNMR ($CDCl_3$, 400 MHz)** δ_H: 0.73 (3H, t, *J* 7.1, COOCH$_2$CH_3), 2.37 (1H, dd, *J* 14.8, 2.5, CH_2CO), 2.81 (1H, dd, *J* 14.8, 11.3, CH_2CONHBn), 3.67–3.84 (2H, m, COOCH_2CH$_3$), 4.38 (1H, dd, *J* 14.7, 5.6, NHCH_2Ph), 4.45 (1H, dd, *J* 14.6, 5.6, NHCH_2Ph), 4.55 (1H, dd , C*H*COOEt), 4.71 (1H, d, *J* 15.6, CH_2Ph), 5.06 (1H, d, *J* 15.6, CH_2Ph), 5.76 (1H, t, *J* 5.4, N*H*CH$_2$Ph), 6.81 (1H, d, *J* 15.6, C*H*-Ar), 7.07–7.11 (1H, m, C*H*-Ar), 7.21–7.36 (15H, m, C*H*-Ar), 7.45–7.47 (2H, m, C*H*-Ar);h32 **^{13}C NMR ($CDCl_3$, 100 MHz)** δ_C: 13.5 (COOCH$_2$$CH_3$), 34.5 ($CH_2$CO), 43.8 ($CH_2$Ph), 44.2 ($CH_2$Ph), 47.8 ($C$HCOOCH$_2CH_3$), 57.3 ($C$(3)), 61.0 (CHCOO$CH_2CH_3$), 109.5 ($C$H-Ar), 122.2 ($C$H-Ar), 125.8 ($C$H-Ar), 127.5 (4×$C$H-Ar), 127.6 (2×$C$H-Ar), 127.7 (2×$C$H-Ar), 127.8 (2×$C$H-Ar), 127.9 ($C$H-Ar), 128.7 (4×$C$H-Ar), 128.9 (2×$C$H-Ar), 135.8 ($C$-Ar), 137.7 ($C$-Ar), 138.2 ($C$-Ar), 143.6 ($C$-Ar), 170.7 ($C$ON), 171.4 ($C$ONHBn), 177.4

(COOCH$_2$CH$_3$); c34**HRMS (NSI$^+$)** exact mass calcd. For $C_{34}H_{33}N_2O_4^+$ (M+H$^+$) requires m/z 533.2435, found m/z 533.2422 (–2.4 ppm).

81$_{minor}$: (30.0 mg, 28%) as a white solid. mp: 82-83°C; $[\alpha]_D^{20}$ = +165.3 (*c* 0.8, CHCl$_3$); Chiral HPLC analysis, Chiralcel OD-H (85:15 hexane : IPA, flow rate 1 mL/min^{-1}, 254 nm, 30 °C) t_R (major): 14.7 min, t_R (minor): 11.8 min, 94:6 er; ν_{max} (film), 3307 (N-H), 1719 (C=O), 1706 (C=O), 1650 (C=O); **^{1}HNMR (CDCl$_3$, 400 MHz)** δ_H: 0.78 (3H, t, *J* 7.2, COOCH$_2$CH_3), 2.33 (1H, dd, *J* 15.3, 3.5, CH_2CONHBn), 2.80 (1H, dd, *J* 15.3, 10.8, CH_2CO), 3.75–3.87 (2H, m, COOCH_2CH$_3$), 4.24–4.30 (2H, m, CHCOOEt, CONHCH_2Ph), 4.36 (1H, dd, *J* 14.7, 5.8, CONHCH_2Ph), 4.93 (1H, d, *J* 15.6, CH_2Ph), 5.00 (1H, d, *J* 15.6, CH_2Ph), 5.71 (1H, d, *J* 5.5, CONHCH_2Ph), 6.75 (1H, d, *J* 7.5, CH-Ar), 7.06–7.10 (1H, m, CH-Ar), 7.17–7.38 (15H, m, CH-Ar), 7.54–7.58 (3H, m, CH-Ar); **^{13}C NMR (CDCl$_3$, 100 MHz)** δ_C: 13.5 (COOCH$_2$$CH_3$), 34.7 ($CH_2$CONHBn), 43.6 ($CH_2$Ph), 44.1 ($CH_2$Ph), 48.5 ($C$HCOOCH$_2$$CH_3$), 57.0 ($C$(3)), 60.7 ($C$HCOO$CH_2$$CH_3$), 109.4 ($C$H-Ar), 122.6 ($C$H-Ar), 126.3 ($C$H-Ar), 127.3 (2×$C$H-Ar), 127.5 (4×$C$H-Ar), 127.7 (2×$C$H-Ar), 127.8 (2×$C$H-Ar), 128.5 ($C$H-Ar), 128.6 (2×$C$H-Ar), 128.7 (2×$C$H-Ar), 128.8 ($C$H-Ar), 129.8 ($C$-Ar), 135.8 ($C$-Ar), 137.5 ($C$-Ar), 138.0 ($C$-Ar), 142.3 ($C$-Ar), 170.3 ($C$ON), 172.4 ($C$ONHBn), 176.7 ($C$OOCH$_2CH_3$); **HRMS (NSI$^+$)** exact mass calcd. For $C_{34}H_{33}N_2O_4^+$ (M+H$^+$) requires m/z 533.2435, found m/z 533.2425 (–1.8 ppm).

5 Determination of Product Configuration

5.1 X-ray crystal structures of 54, 29 and 29'.

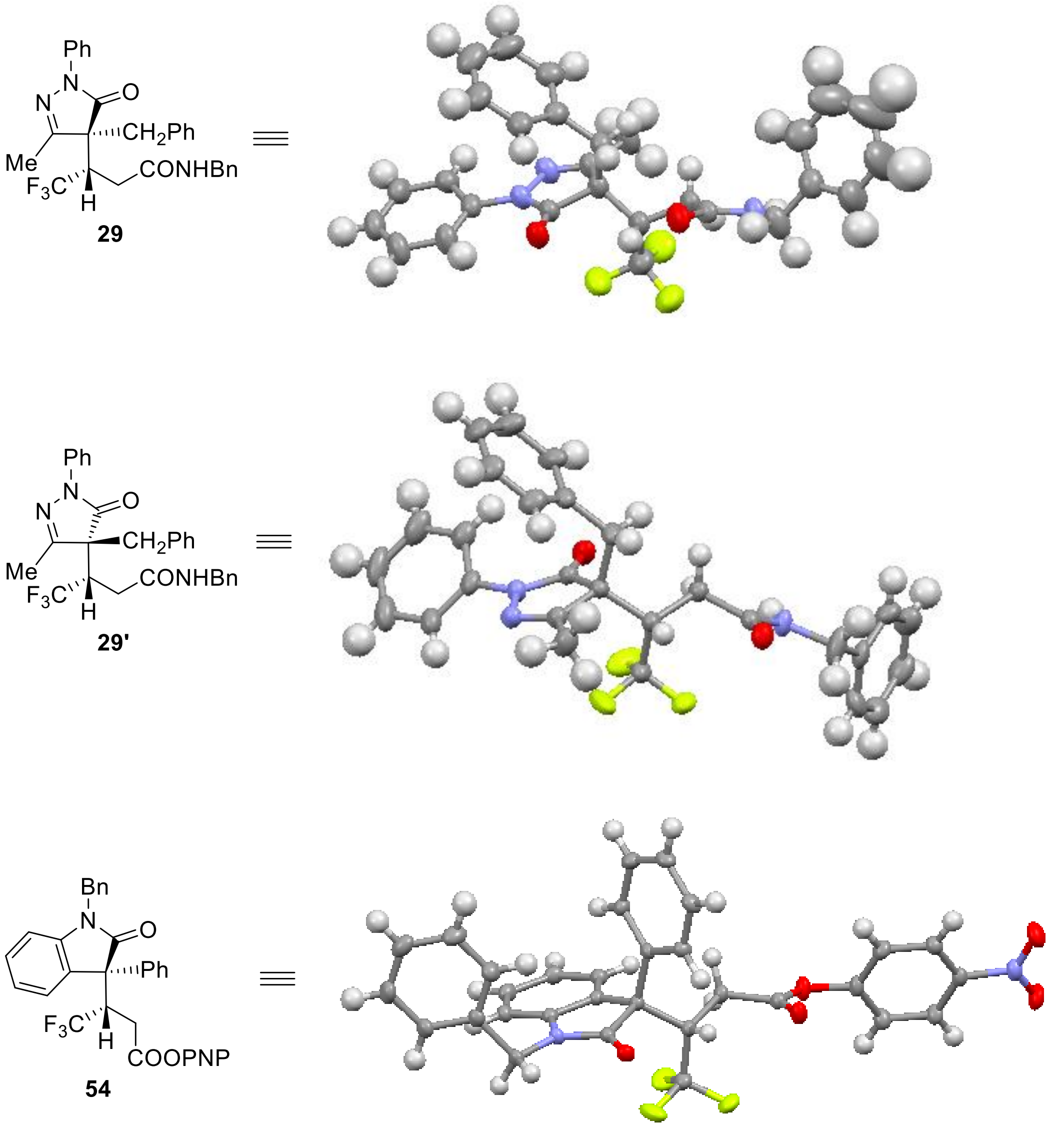

Figure S1: X-Ray crystal structures confirming the relative and absolute stereochemistry of **29**, **29'** and **54**.

5.1 Crystal data of 29, 29' and 54.

Crystal data for **29**: $C_{28}H_{26}F_3N_3O_2$, M = 493.52, colourless prism, orthorhombic space group *P*1 21 1; *a* = 13.5575(2) Å, *b* = 23.9425(3) Å, *c* = 19.8418(3) Å, α = 90°, β = 93.584°, γ = 90°, *V* = 6428.06(16) Å^3, *Z* = 10, D_x = 1.275 g cm^{-3}, *R* = 0.0406. Data were recorded at 173 K on Rigaku XtaLAB P100 diffractometer using multi-layer mirror monochromated Cu-Kσ radiation and the structures were solved by direct methods and refined using full-matrix least square analysis.

Crystal data for **29'**: $C_{28}H_{26}F_3N_3O_2$, M = 493.53, colourless prism, orthorhombic space group *P*1 21/c 1; *a* = 19.753(4) Å, *b* = 13.427(3) Å, *c* = 9.3303(18) Å, α = 90°, β = 93.119(8)°, γ = 90°, *V* = 2470.9(9) Å^3, *Z* = 4, D_x = 1.327 g cm^{-3}, *R* = 0.0480. Data were recorded at 173 K on Rigaku XtaLAB P100

diffractometer using multi-layer mirror monochromated Cu-Kσ radiation and the structures were solved by direct methods and refined using full-matrix least square analysis.

Crystal data for **54**: $C_{31}H_{23}F_3N_2O_5$, M = 560.53, colourless prism, orthorhombic space group *P*1 21 1; *a* = 8.97942(5) Å, *b* = 10.93580(6) Å, *c* = 27.05100(16) Å, α = 90°, β = 90°, γ = 90°, *V* = 2656.33(3) $Å^3$, *Z* = 4, D_x = 1.401 g cm^{-3}, *R* = 0.0268. Data were recorded at 173 K on Rigaku XtaLAB P100 diffractometer using multi-layer mirror monochromated Cu-Kσ radiation and the structures were solved by direct methods and refined using full-matrix least square analysis.

6 References

1. Badiola, Eider; Fiser, Béla; Gómez-Bengoa, Enrique; Mielgo, Antonia; Olaizola, Iurre; Urruzuno, Iñaki; García, Jesús M.; Odriozola, José M.; Razkin, Jesús; Oiarbide, Mikel; Palomo, Claudio. *J. Am. Chem. Soc.*, **2014**, *136*, 17869–17881.
2. Wang, Tianli; Yu, Zhaoyuan; Hoon, Ding Long; Huang, Kuo-Wei; Lan, Yu; Lu, Yixin. *Chem. Sci.*, **2015**, *6*, 4912–4922.
3. Ruble, J. Craig; Fu, Gregory C. *J. Am. Chem. Soc.*, **1998**, *120*, 11532–11533.
4. Huang, Audris; Kodanko, Jeremy J.; Overman, Larry E. *J. Am. Chem. Soc*, **2004**, 126, 14043–14053.
5. Sheng, Xuguang; Zhang, Jinlong; Yang, Huameng; Jiang, Gaoxi. *Org. Lett.*, **2017**, *19*, 2618–2621.

Appendix I: Reaction of Unsuccessful Pronucleophiles

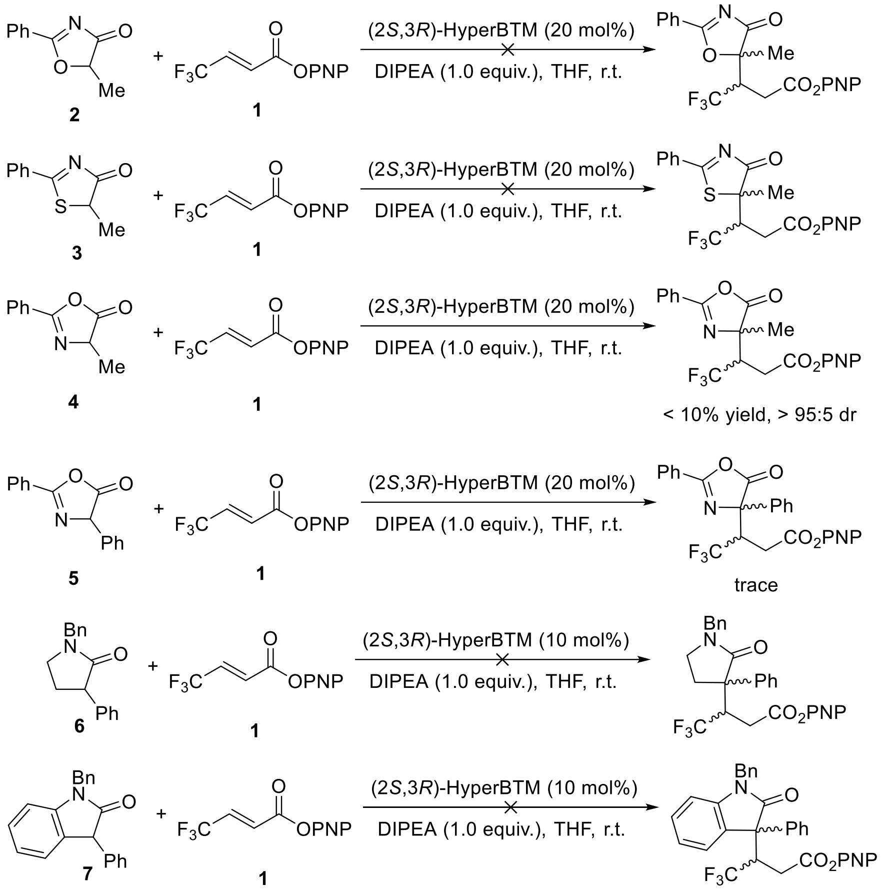

Appendix II: Supplementary Optimisation Studies

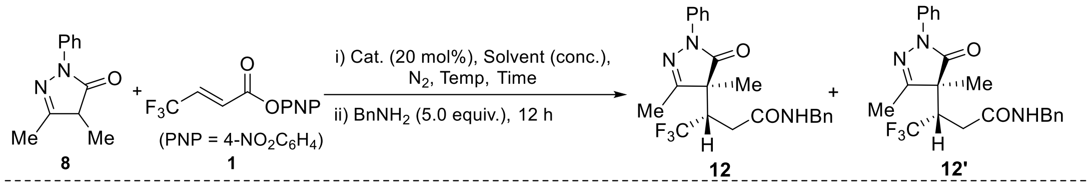

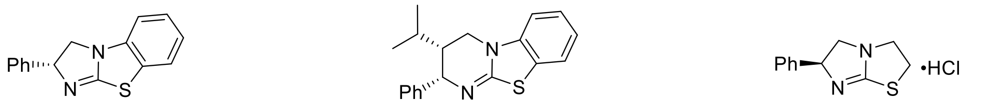

(*R*)-BTM **11** (2*S*,3*R*)-HyperBTM **10** (-)-Tetramisole hydrochloride **S5**

Entry	Cat.	Solvent	Temp [°C]	Time [h]	Yield [%][a]	dr[b]	**12**, *ee* [%][c]	**12'**, *ee* [%][c]
1	**11**	THF	r.t.	6	84	67:33	91	91
2	**10**	THF	r.t.	6	72	68:32	93	91
3[d]	**S5**	THF	r.t.	6	64	72:28	−82	−56
4	**10**	CH_3CN	r.t.	6	64	63:37	88	87
5	**10**	DMF	r.t.	6	74	66:34	78	86
6	**10**	DCM	r.t.	24	42	58:42	92	90
7	**10**	toluene	r.t.	24	36	67:33	92	89
8	**10**	Et_2O	r.t.	6	45	53:47	90	93
9[e]	**10**	THF	r.t.	6	53	68:32	89	89
10[f]	**10**	THF	r.t.	6	48	74:26	88	89
11[g]	**10**	THF	r.t.	6	61	67:33	86	88
12[h]	**10**	THF	r.t.	6	63	67:33	89	88
13	**10**	THF	0 °C	24	87	72:28	92	92
14	**11**	THF	0 °C	24	77	70:30	92	91
15	**10**	THF	– 10 °C	24	82	75:25	91	93
16	**10**	THF	– 40 °C	48	36	50:50	94	94
17[j]	**10**	THF	r.t.	72	47	68:32	91	90

[a] Isolated overall yields given.

[b] dr of crude product determined by ^{1}H NMR spectroscopic analysis.

[c] *ee* determined by chiral HPLC analysis.

[d] 0.2 mmol of ^{i}Pr$_2$NEt was added.

[e] 1 mL of solvent was used.

[f] 4 mL of solvent was used.

[g] 0.24 mmol of **5a** was used.

[h] 0.24 mmol of **2a** was used.

[i] 0.02 mmol of catalyst was used.

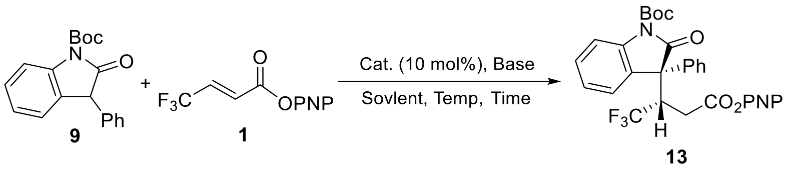

Entry	Cat.	Base	Solvent	Temp [°C]	Time [h]	Yield [%][a]	dr[b]	**13**, *ee* [%][c]
1	**11**	DIPEA (1.5 equiv.)	THF	0	16	71	87:13	93:7
2	**11**	DABCO (1.5 equiv.)	THF	0	16	60	86:14	92:8
3	**10**	DIPEA (1.5 equiv.)	THF	0	16	76	83:17	98:2
4	**10**	DIPEA (1.5 equiv.)	CH_2Cl_2	0	16	78	83:17	95:5
5	**10**	DIPEA (1.5 equiv.)	$CHCl_3$	0	48	23	71:29	91:9
6	**10**	DIPEA (1.5 equiv.)	toluene	0	48	58	75:25	95:5
7	**10**	DIPEA (1.5 equiv.)	CH_3CN	0	16	88	83:17	96.5:3.5
8	**10**	DIPEA (1.5 equiv.)	DMF	0	16	54	78:22	90:10
9	**10**	DIPEA (1.5 equiv.)	acetone	0	16	60	83:17	98:2
10	**10**	DIPEA (1.0 equiv.)	THF	0	16	79	83:17	97.5:2.5
11	**10**	DIPEA (0.5 equiv.)	THF	0	16	78	86:14	97.5:2.5
12	**10**	DIPEA (1.0 equiv.)	THF	r.t.	16	79	84:16	98:2

[a] Isolated overall yields given; [b] dr of crude product determined by ^{1}H NMR spectroscopic analysis; [c] *ee* determined by chiral HPLC analysis.

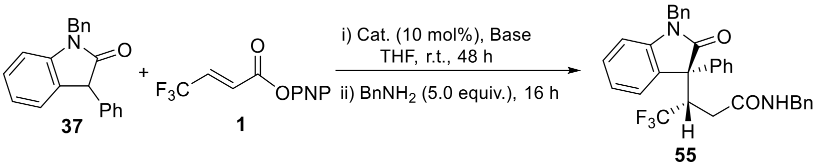

Entry	Cat.	Base	Yield [%][a]	dr[b]	**55**, *ee* [%][c]
1	**10**	DIPEA (0.2 equiv.)	69	>99:1	99.5:0.5
2	**10**	DIPEA (0.5 equiv.)	62	>99:1	98.5:1.5
3	**10**	DIPEA (0.75 equiv.)	75	>99:1	98.5:1.5
4	**10**	DIPEA (1.0 equiv.)	86	>99:1	99.5:0.5
5	**10**	-	59	>99:1	99:1
6	-	DIPEA (1.0 equiv.)	NR	-	-

[a] Isolated overall yields given; [b] dr of crude product determined by ^{1}H NMR spectroscopic analysis; [c] *ee* determined by chiral HPLC analysis.

Appendix III: NMR Spectra

(*S*)-*N*-benzyl-3-((*S*)-1-benzyl-3,4-dimethyl-5-oxo-4,5-dihydro-1*H*-pyrazol-4-yl)-4,4,4-trifluorobutanamide (12_{major})

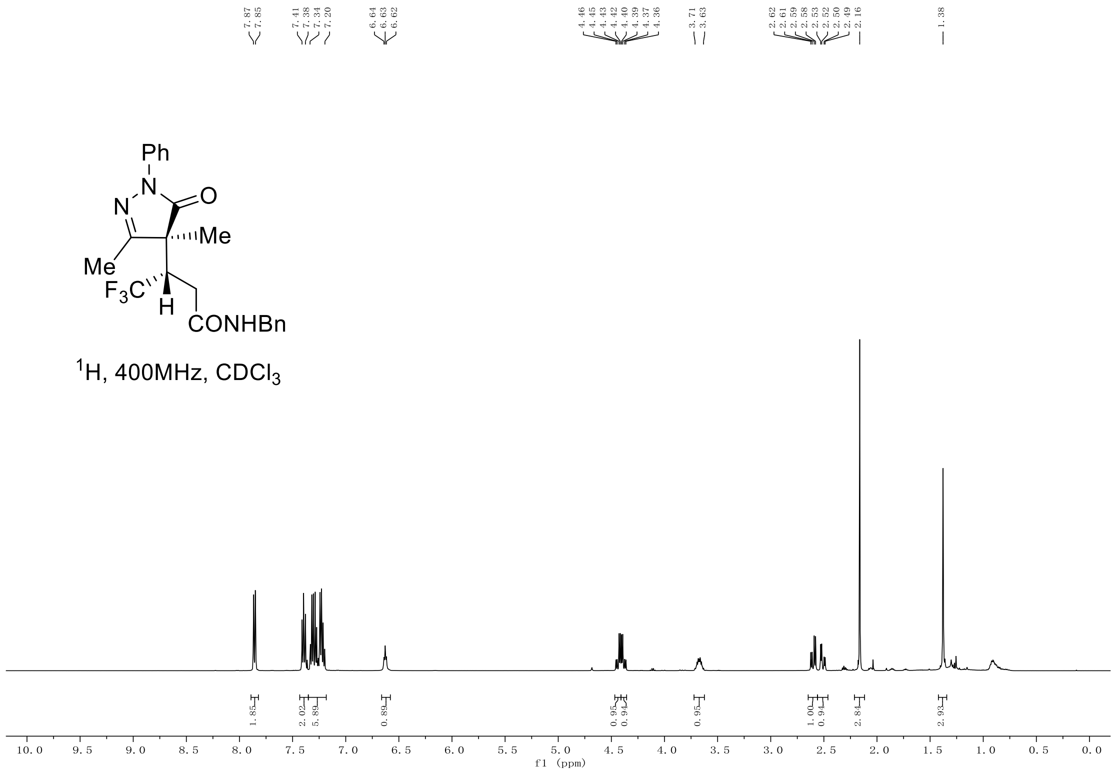

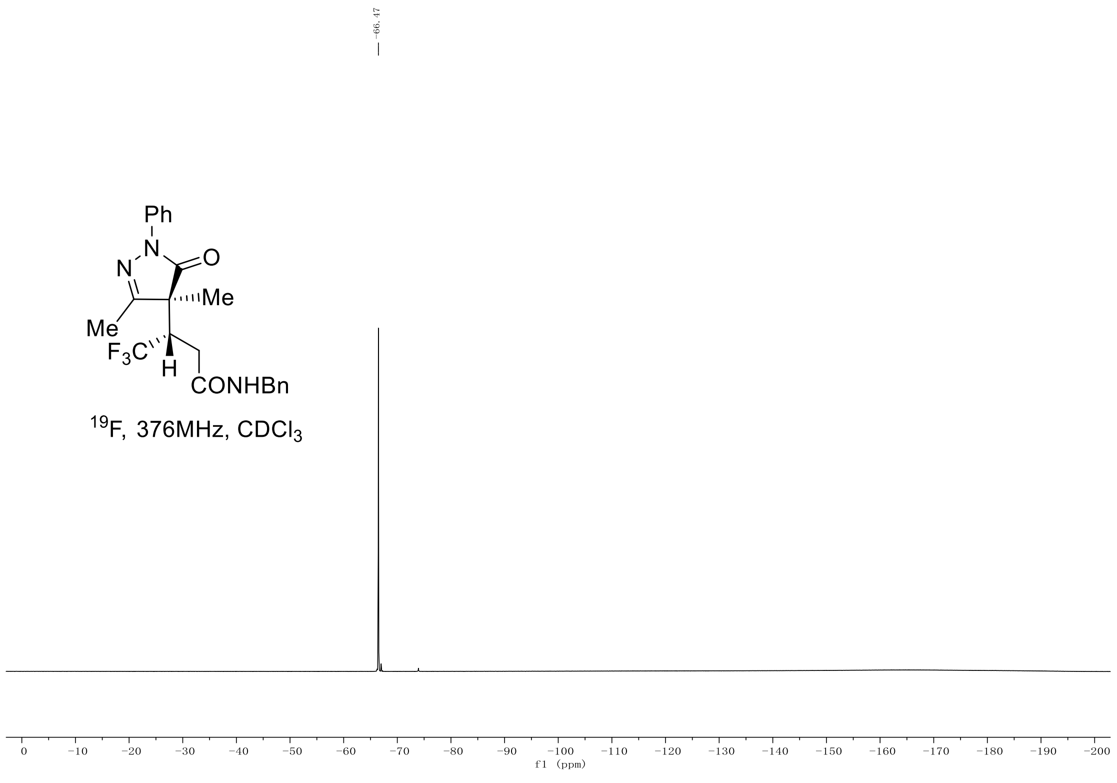

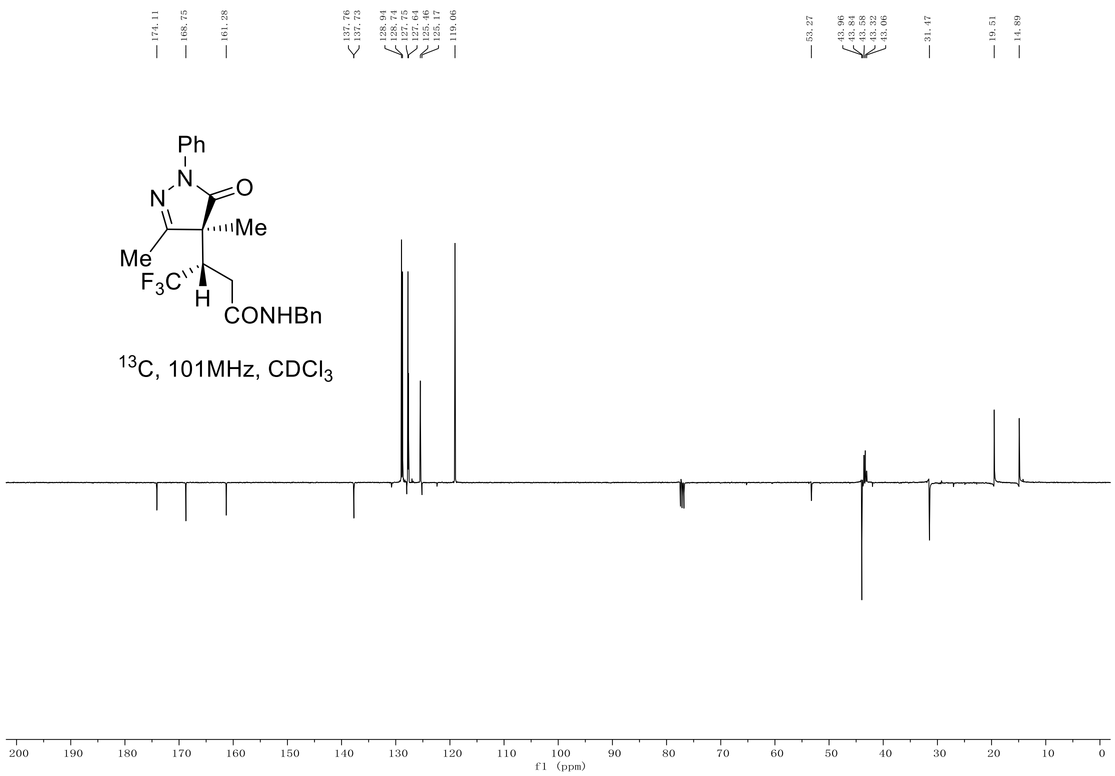

(*S*)-*N*-benzyl-3-((*R*)-3,4-dimethyl-5-oxo-1-phenyl-4,5-dihydro-1*H*-pyrazol-4-yl)-4,4,4-trifluorobutanamide (12$_{\mathbf{minor}}$)

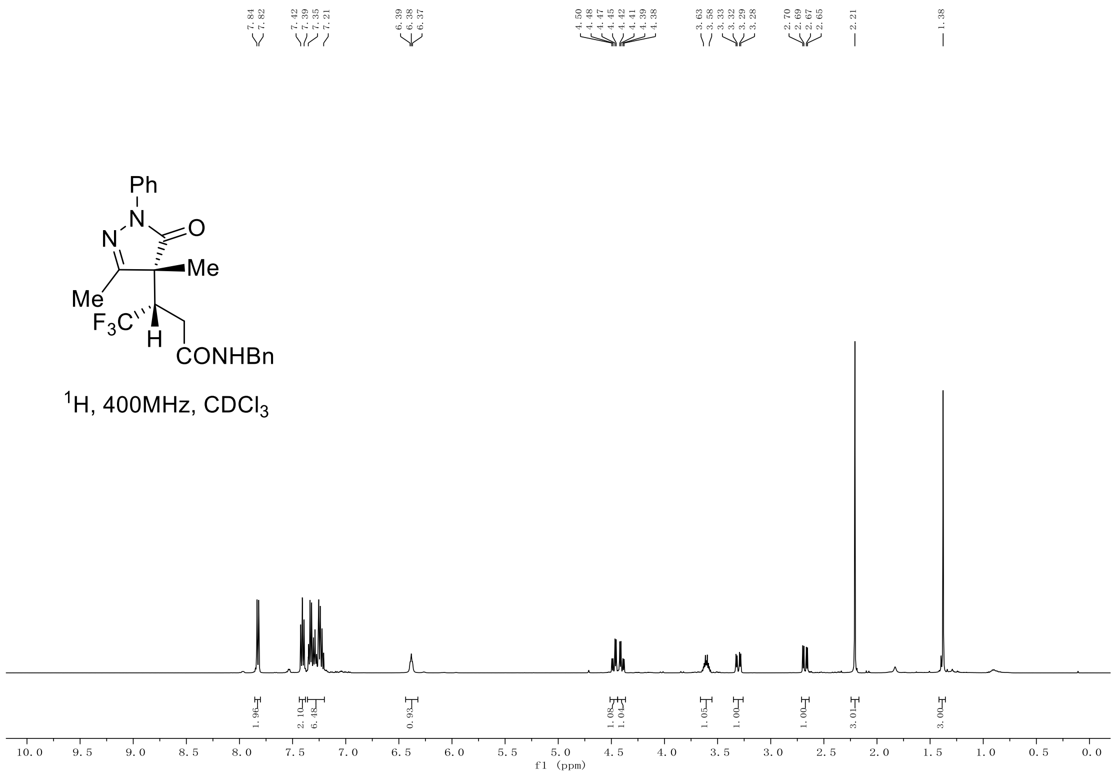

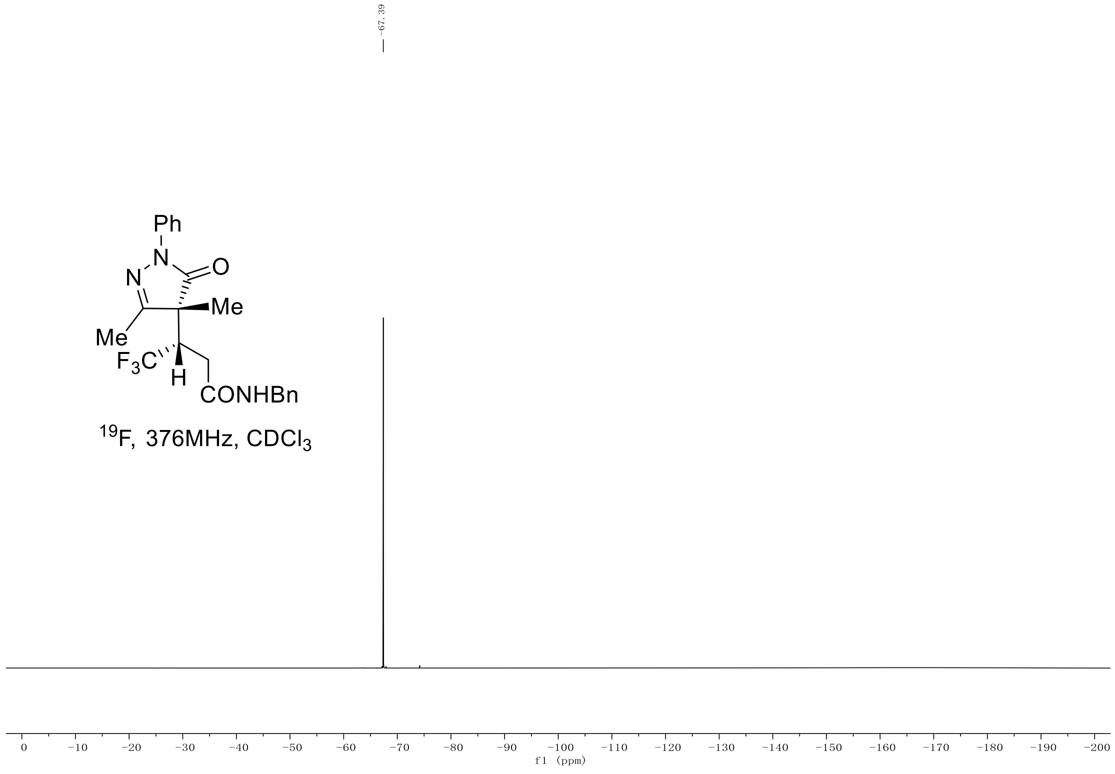

-67.39
Ph
N
N
O
Me
Me
F3C
H
CONHBn
19F, 376MHz, CDCl3
0 −10 −20 −30 −40 −50 −60 −70 −80 −90 −100 −110 −120 −130 −140 −150 −160 −170 −180 −190 −200
f1 (ppm)

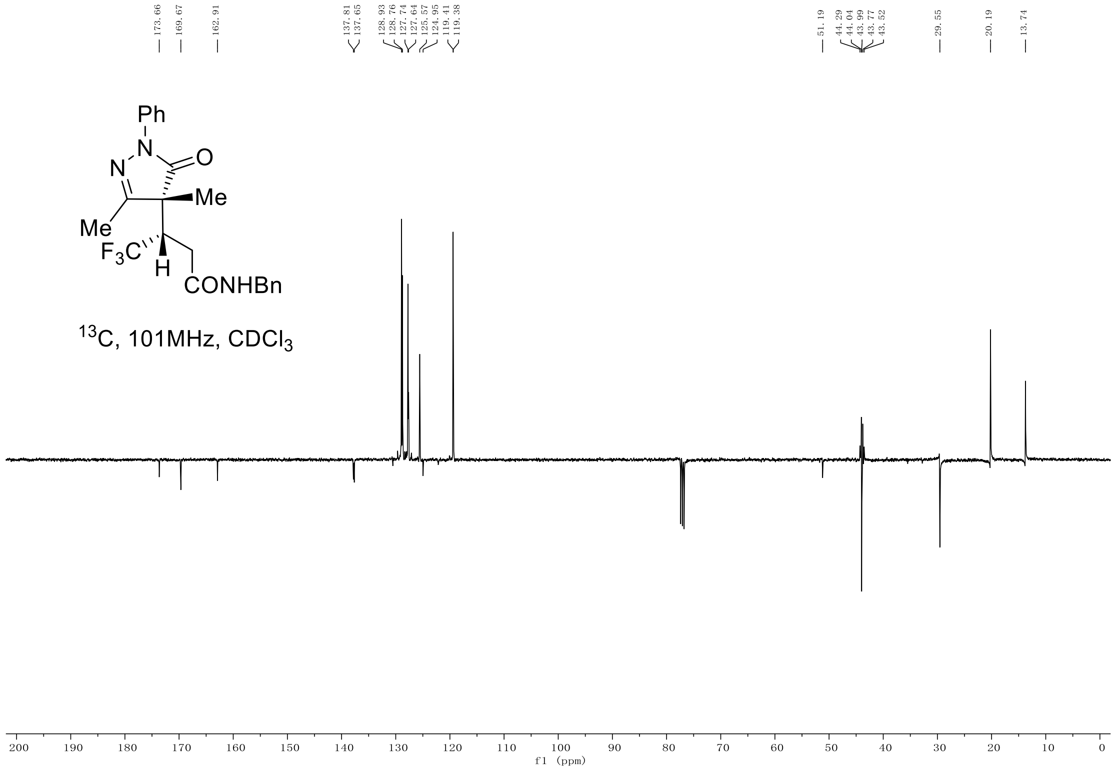

173.66
169.67
162.91
137.81
137.65
128.93
128.76
127.74
127.64
125.57
124.95
119.41
119.38
51.19
44.29
44.04
43.99
43.77
43.52
29.55
20.19
13.74
Ph
N
N
O
Me
Me
F3C
H
CONHBn
13C, 101MHz, CDCl3
200 190 180 170 160 150 140 130 120 110 100 90 80 70 60 50 40 30 20 10 0
f1 (ppm)

(*S*)-*N*-benzyl-4,4,4-trifluoro-3-((*S*)-1,3,4-trimethyl-5-oxo-4,5-dihydro-1*H*-pyrazol-4-yl)butanamide (25_{major})

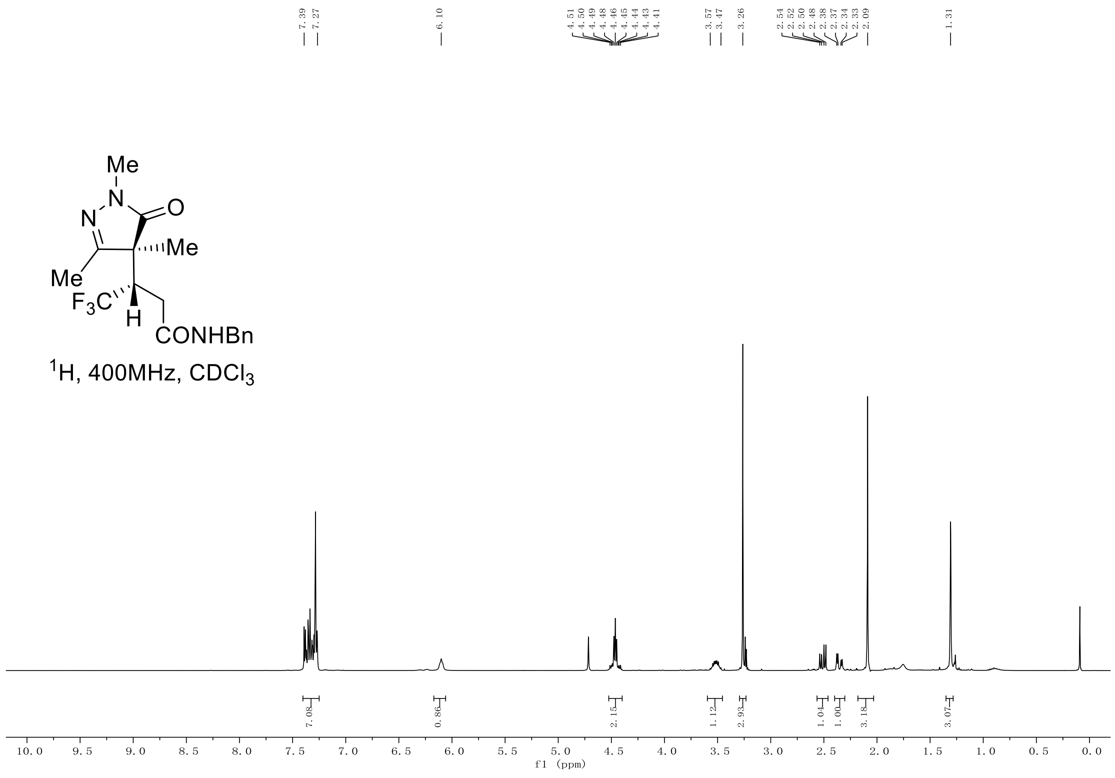

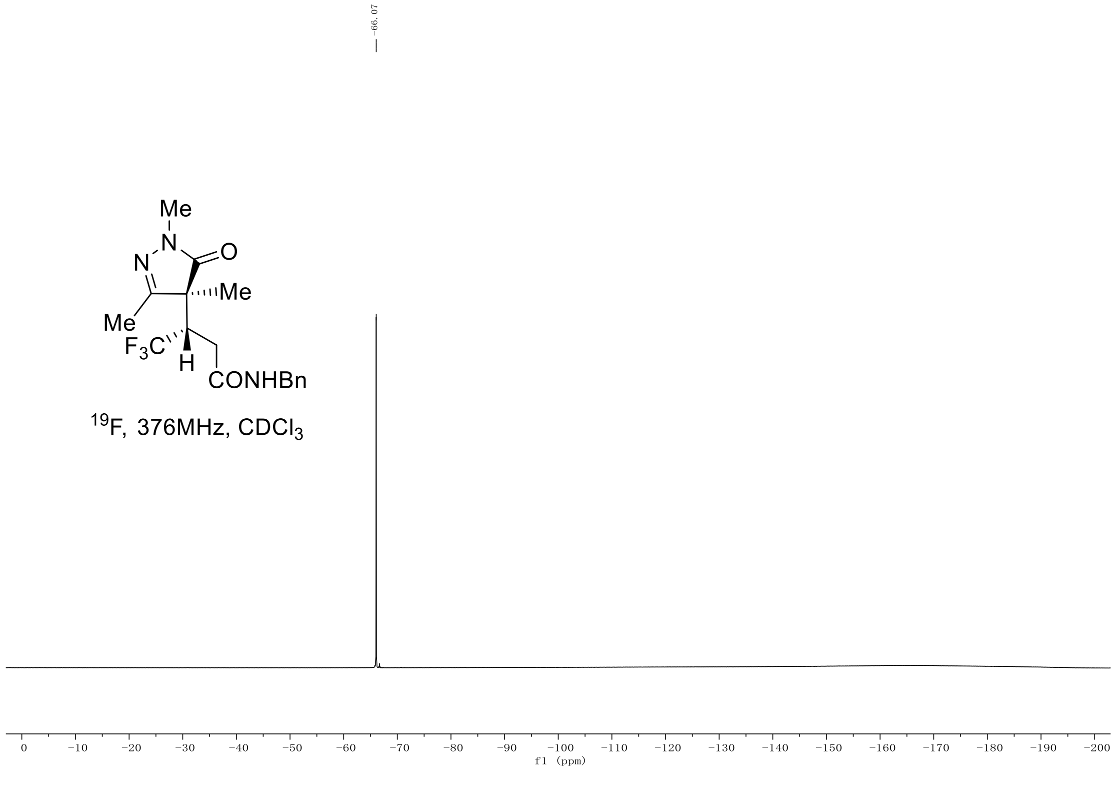

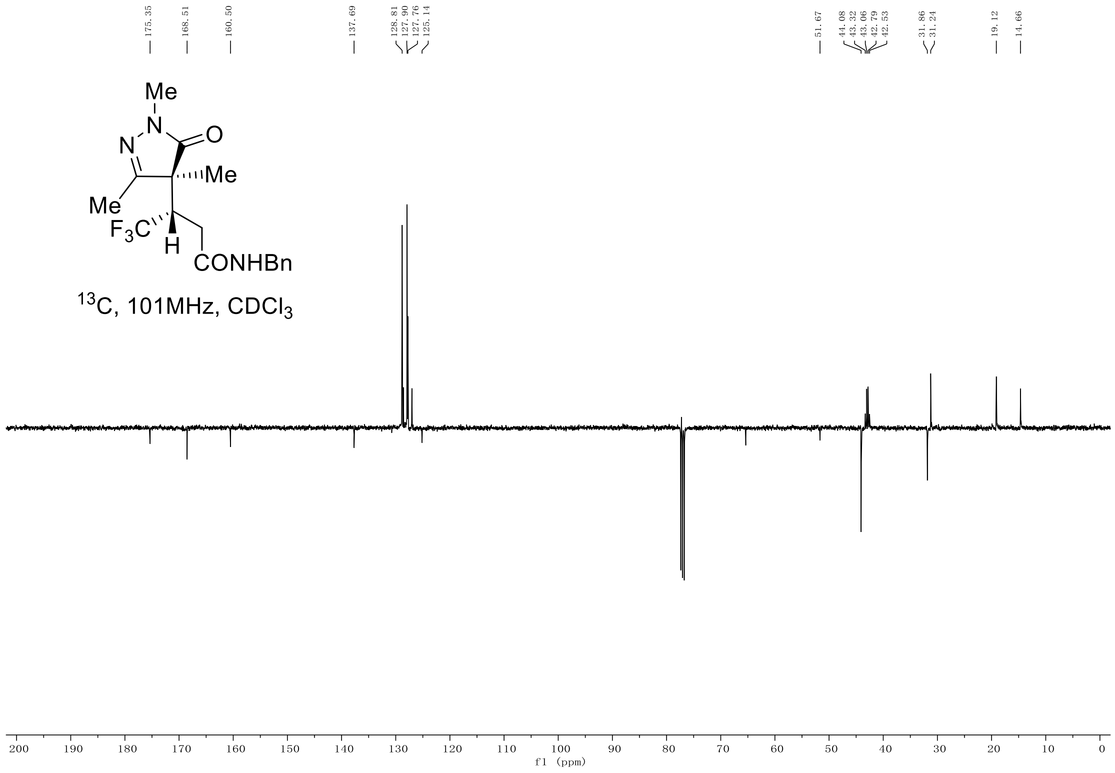

(*S*)-*N*-benzyl-4,4,4-trifluoro-3-((*R*)-1,3,4-trimethyl-5-oxo-4,5-dihydro-1*H*-pyrazol-4-yl)butanamide (**25$_{minor}$**)

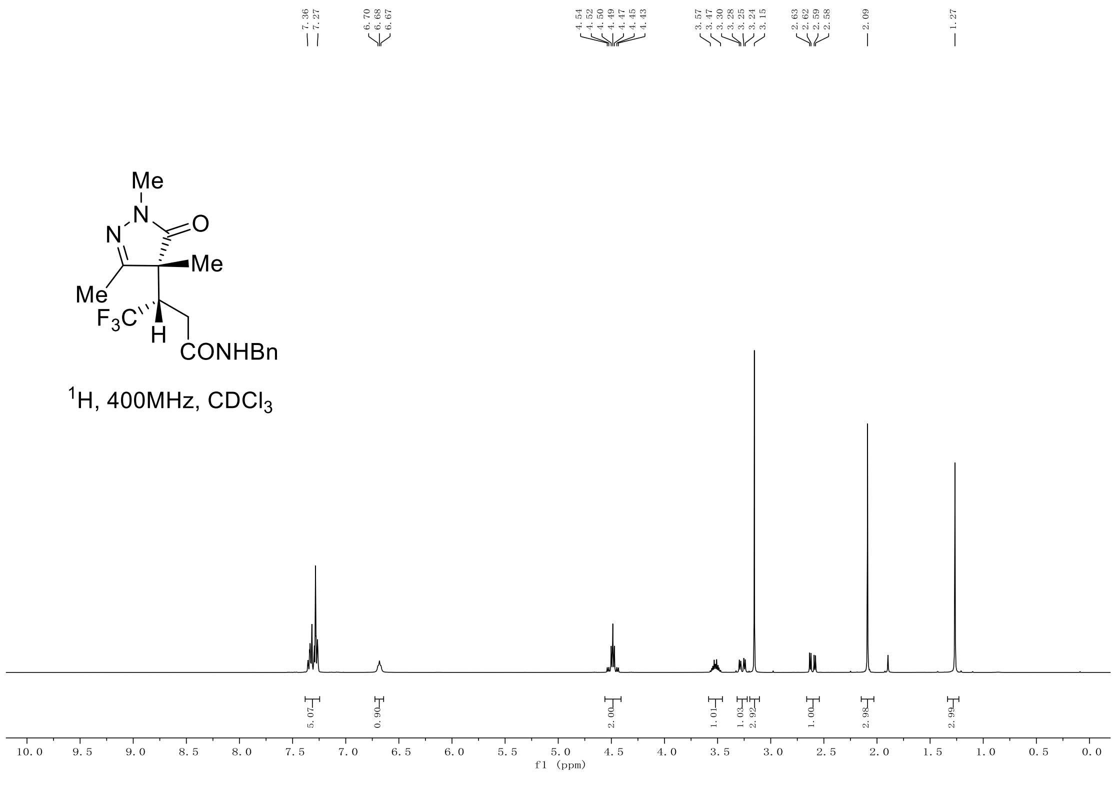

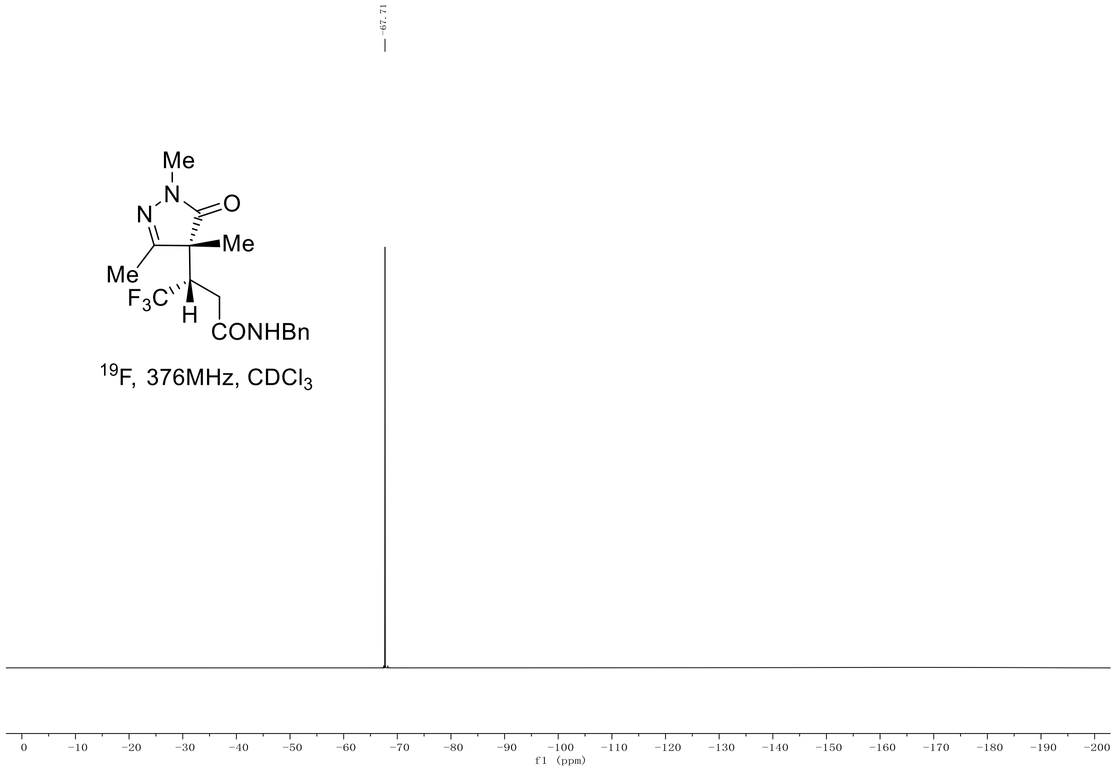

-67.71
Me
N
N
O
Me
Me
F3C
H
CONHBn
19F, 376MHz, CDCl3
0 -10 -20 -30 -40 -50 -60 -70 -80 -90 -100 -110 -120 -130 -140 -150 -160 -170 -180 -190 -200
f1 (ppm)

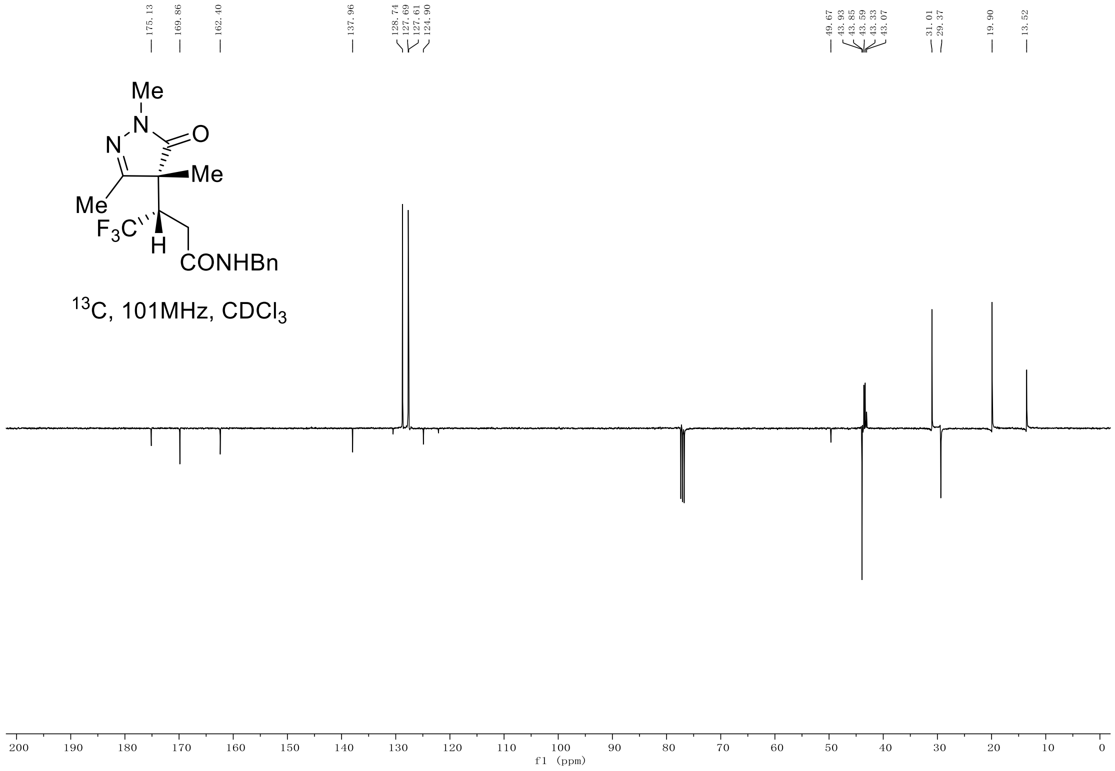

175.13
169.86
162.40
137.96
128.74
127.69
127.61
124.90
49.67
43.93
43.85
43.59
43.33
43.07
31.01
29.37
19.90
13.52
Me
N
N
O
Me
Me
F3C
H
CONHBn
13C, 101MHz, CDCl3
200 190 180 170 160 150 140 130 120 110 100 90 80 70 60 50 40 30 20 10 0
f1 (ppm)

(*S*)-*N*-benzyl-3-((*S*)-1-benzyl-3,4-dimethyl-5-oxo-4,5-dihydro-1*H*-pyrazol-4-yl)-4,4,4-trifluorobutanamide (26$_{major}$)

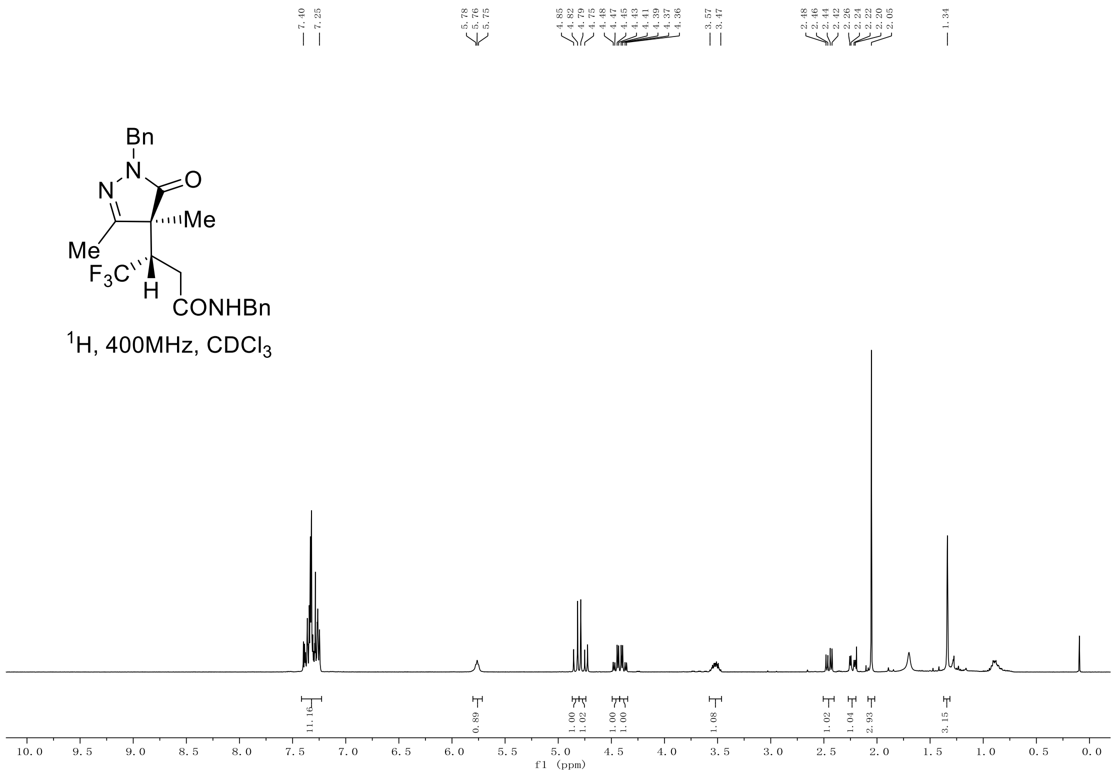

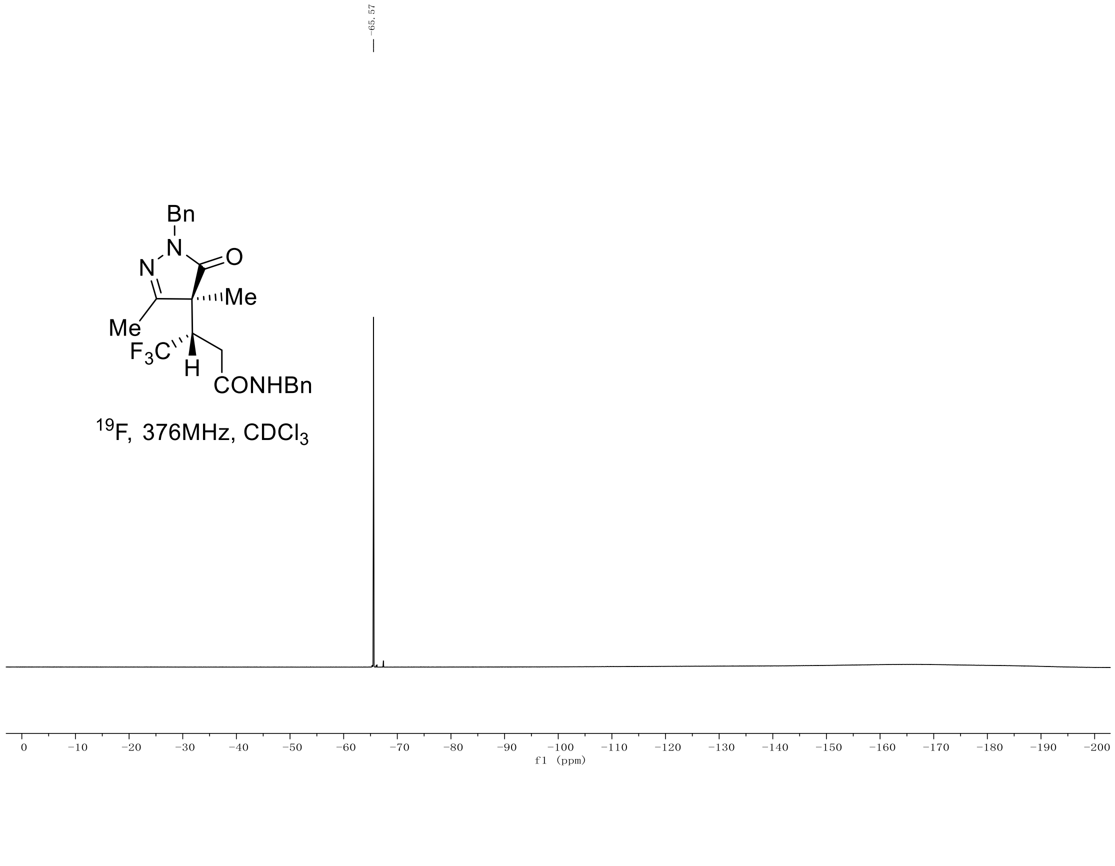

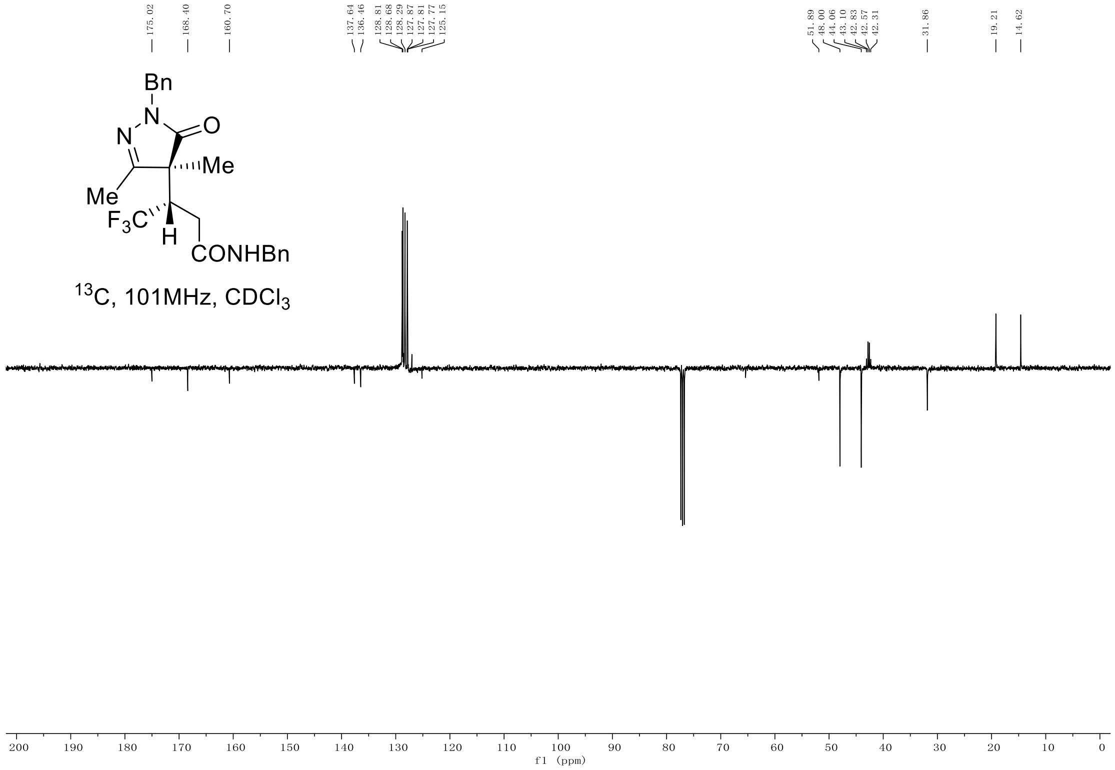

(*S*)-*N*-benzyl-3-((*R*)-1-benzyl-3,4-dimethyl-5-oxo-4,5-dihydro-1*H*-pyrazol-4-yl)-4,4,4-trifluorobutanamide (26_{minor})

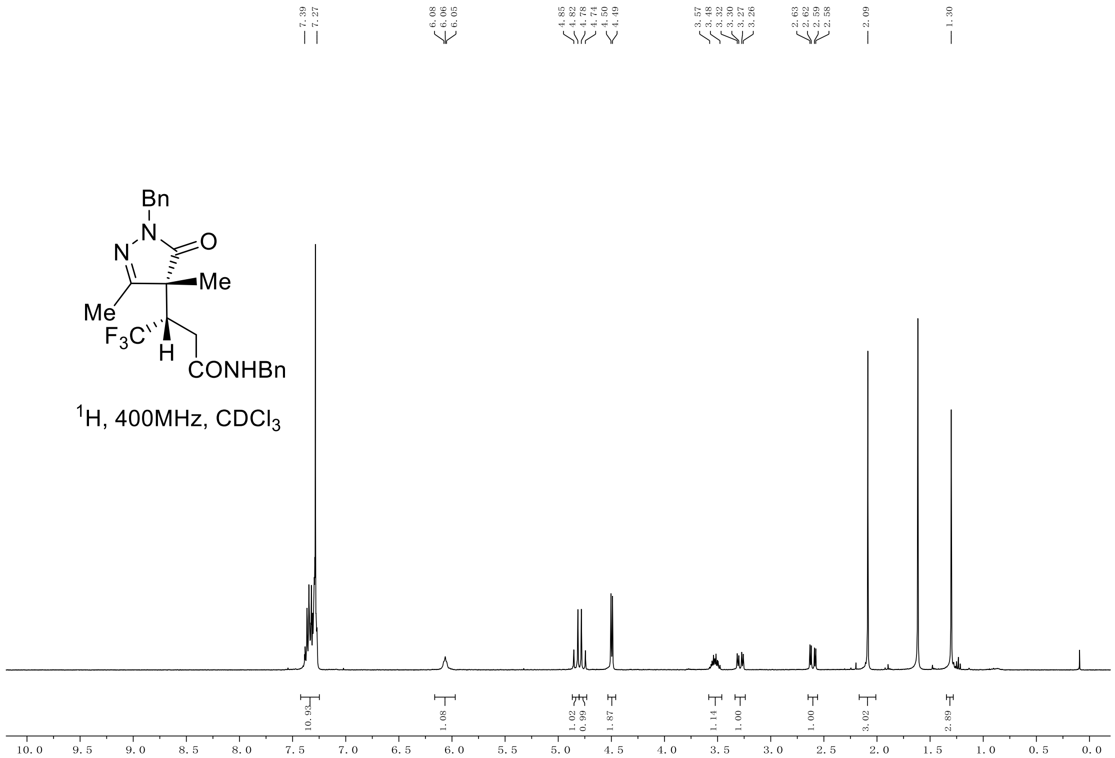

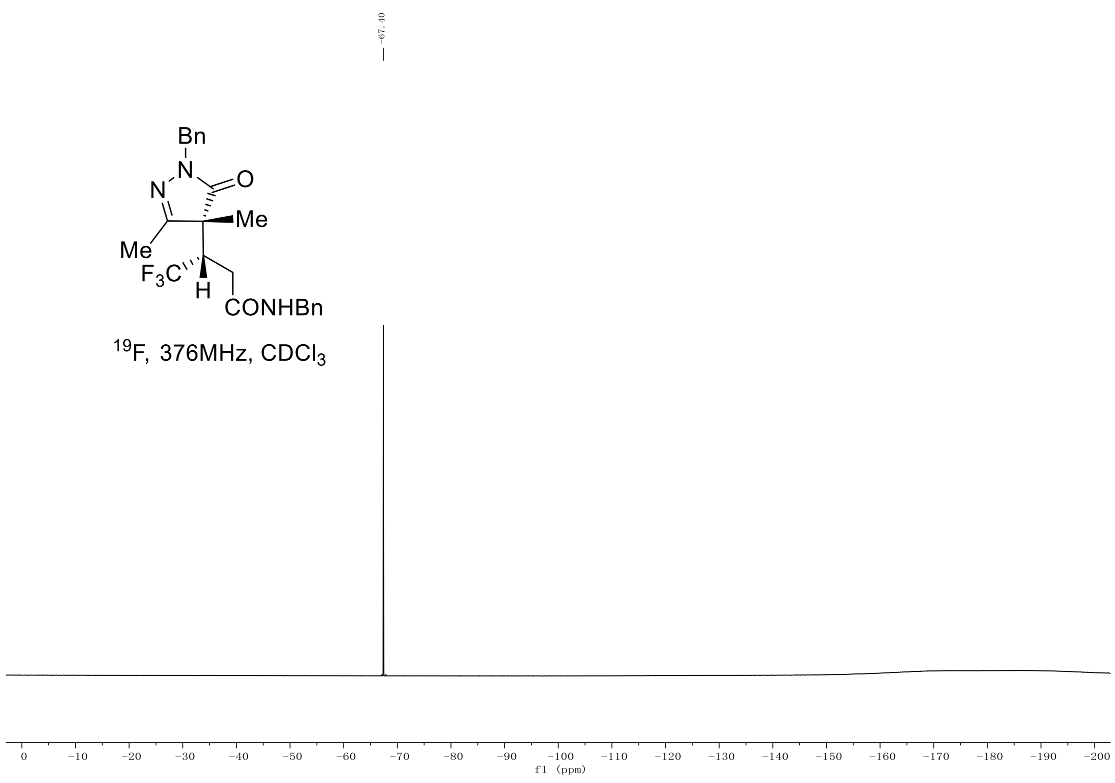
-67.40
Bn
N
N
O
Me
Me
F3C
H
CONHBn
19F, 376MHz, CDCl3
f1 (ppm)

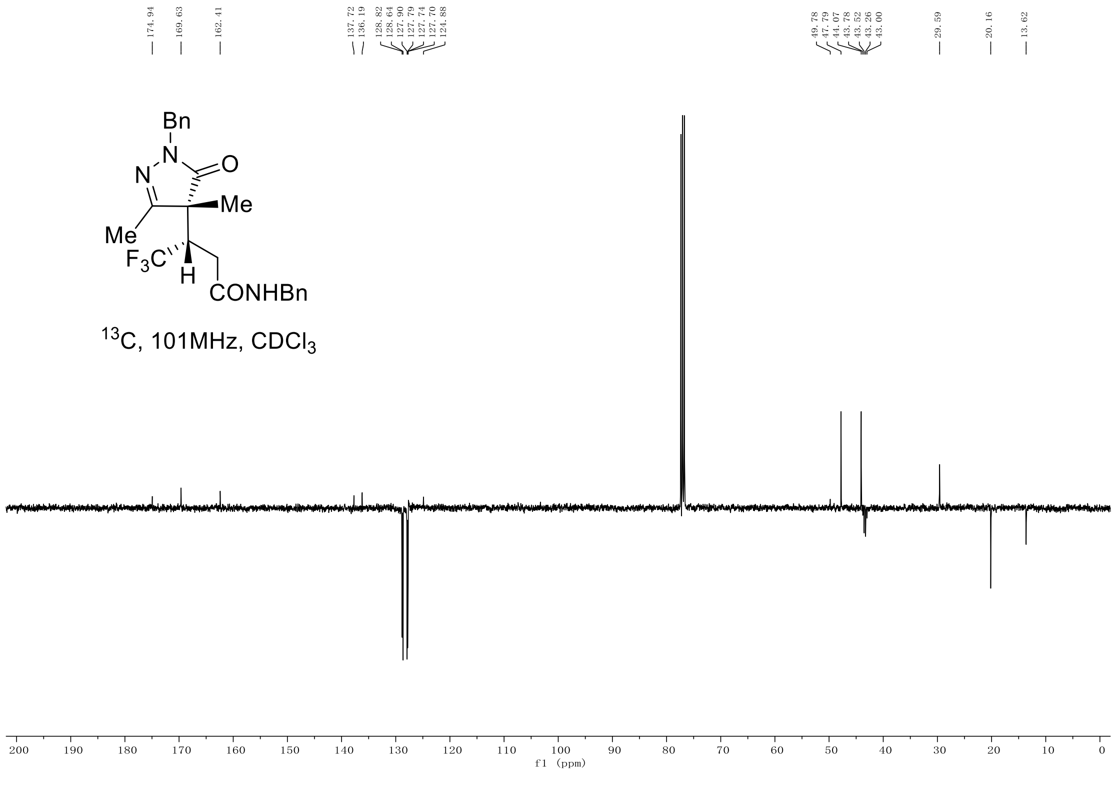
174.94
169.63
162.41
137.72
136.19
128.82
128.64
127.90
127.79
127.74
127.70
124.88
49.78
47.79
44.07
43.78
43.52
43.26
43.00
29.59
20.16
13.62
Bn
N
N
O
Me
Me
F3C
H
CONHBn
13C, 101MHz, CDCl3
f1 (ppm)

(*S*)-*N*-benzyl-3-((*S*)-4-ethyl-3-methyl-5-oxo-1-phenyl-4,5-dihydro-1*H*-pyrazol-4-yl)-4,4,4-trifluorobutanamide (27_{major})

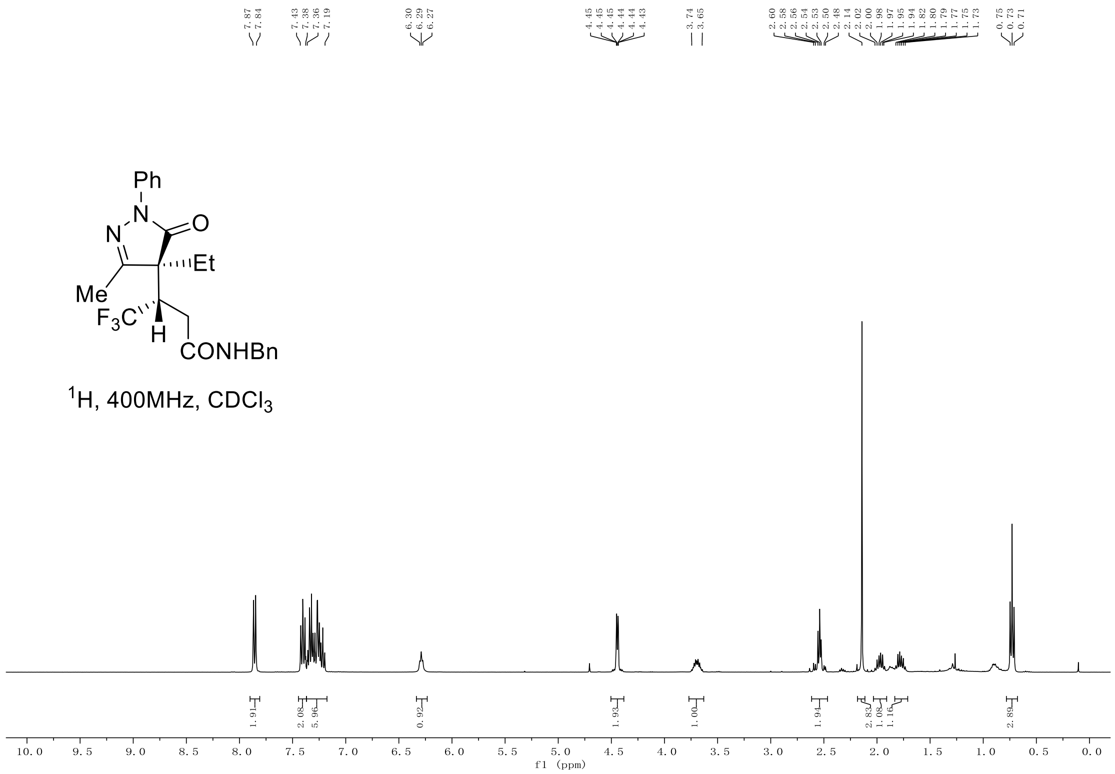

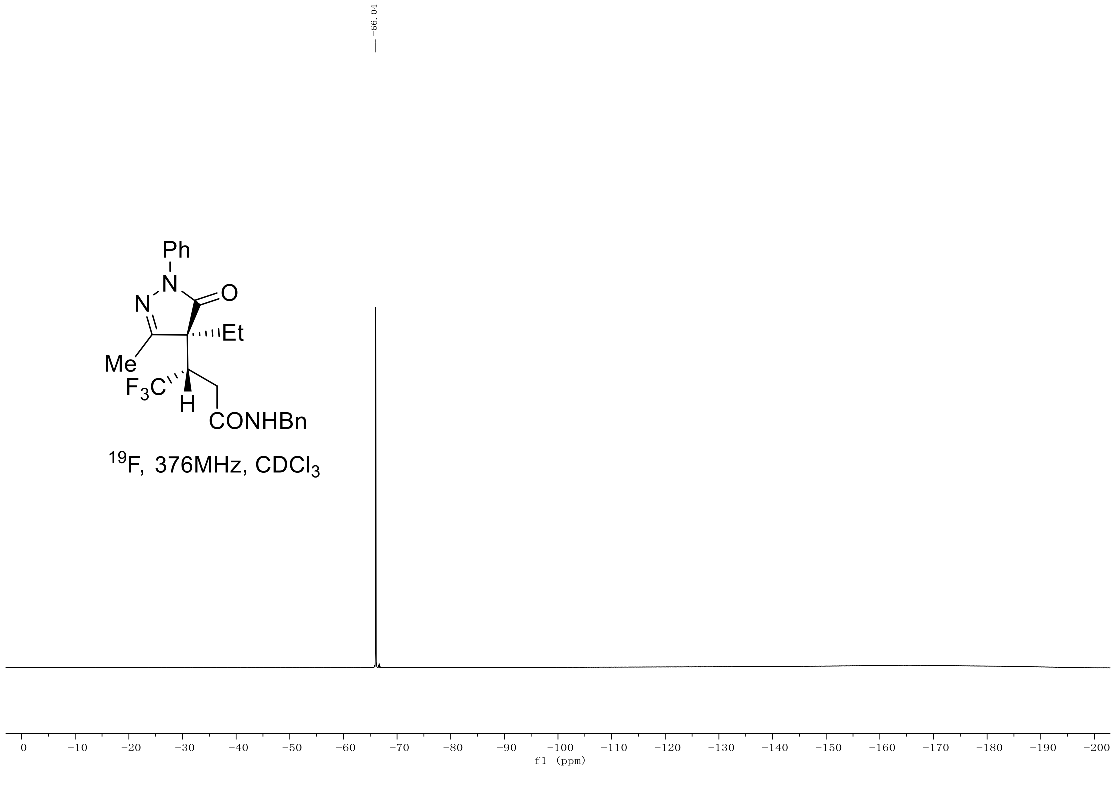

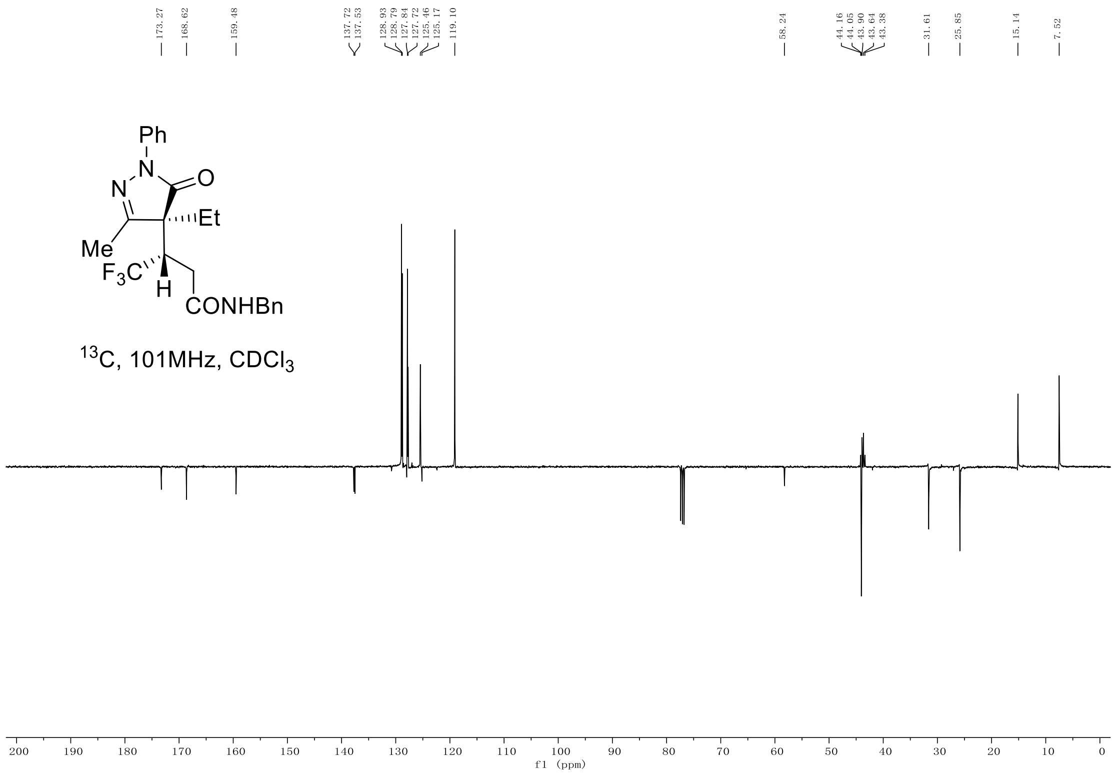

(*S*)-*N*-benzyl-3-((*R*)-4-ethyl-3-methyl-5-oxo-1-phenyl-4,5-dihydro-1*H*-pyrazol-4-yl)-4,4,4-trifluorobutanamide (27$_{minor}$)

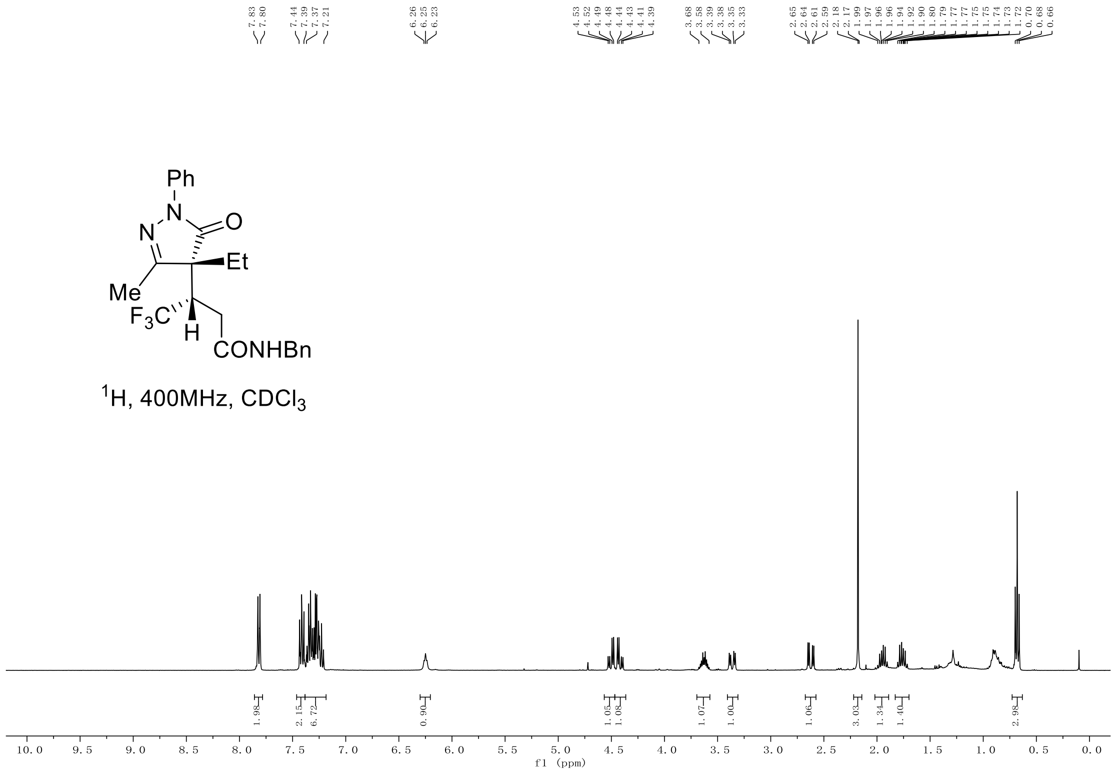

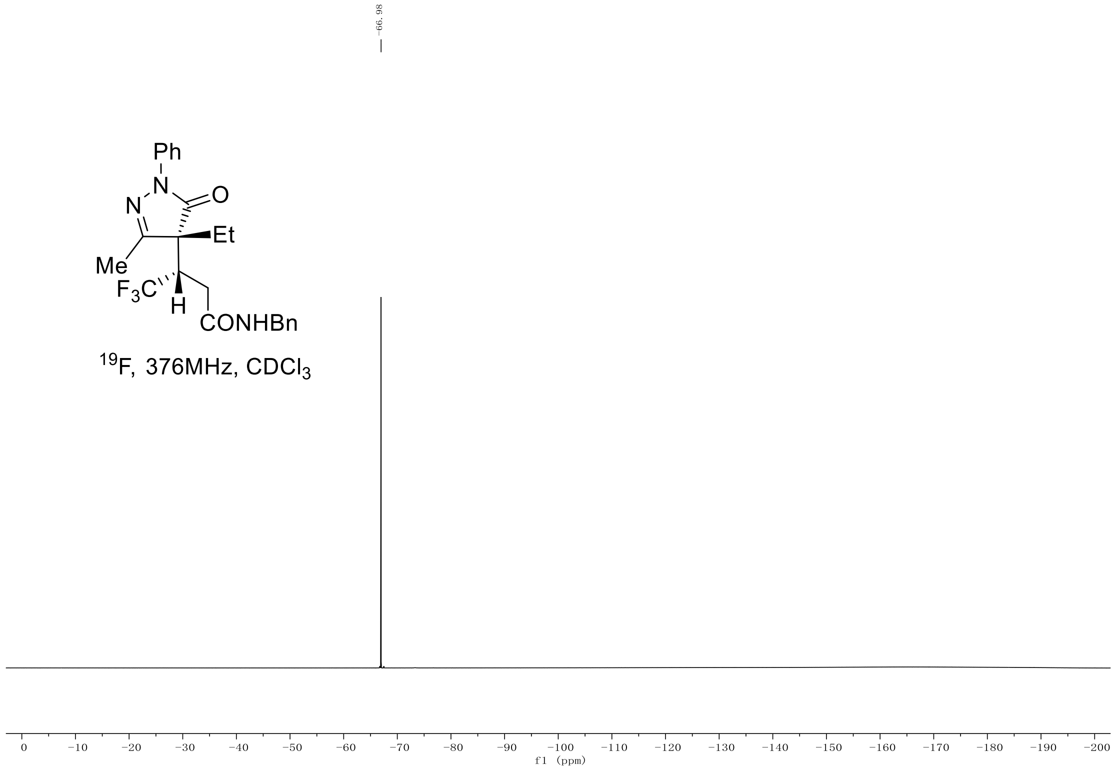

-66.98
Ph
N
N
O
Et
Me
F3C
H
CONHBn
19F, 376MHz, CDCl3
f1 (ppm)

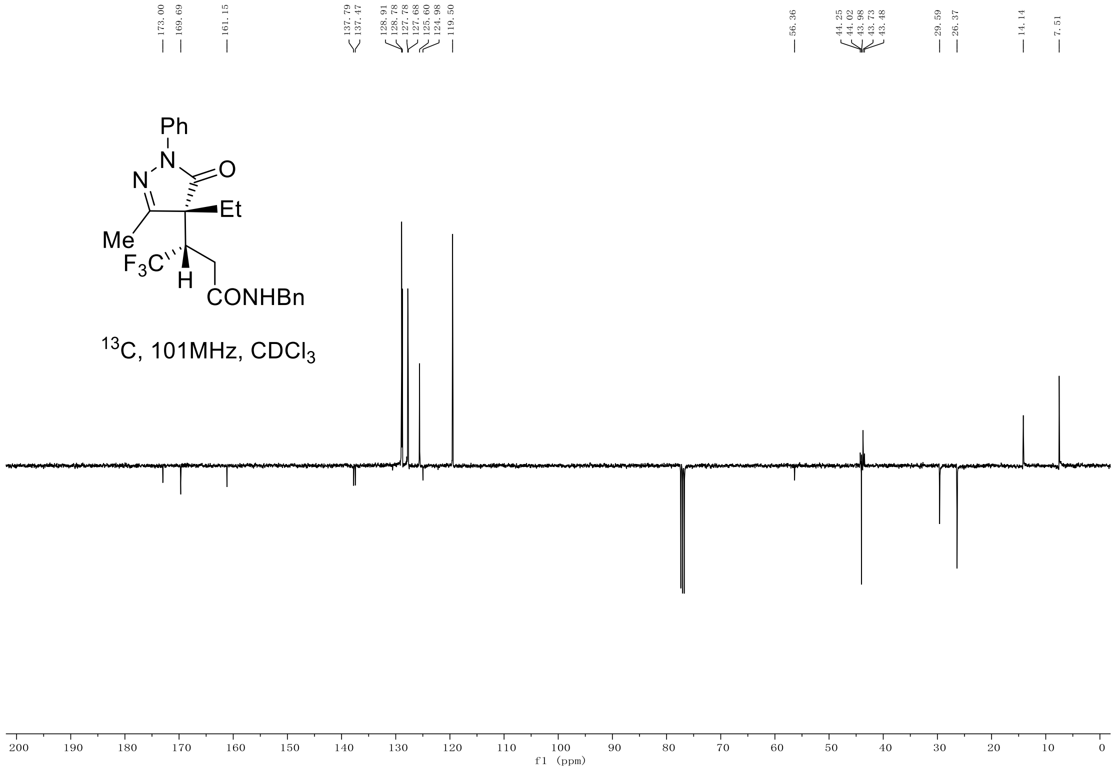

173.00
169.69
161.15
137.79
137.47
128.91
128.78
127.78
127.68
125.60
124.98
119.50
56.36
44.25
44.02
43.98
43.73
43.48
29.59
26.37
14.14
7.51
Ph
N
N
O
Et
Me
F3C
H
CONHBn
13C, 101MHz, CDCl3
f1 (ppm)

(*S*)-3-((*S*)-4-allyl-3-methyl-5-oxo-1-phenyl-4,5-dihydro-1*H*-pyrazol-4-yl)-N-benzyl-4,4,4-trifluorobutanamide (28$_{major}$)

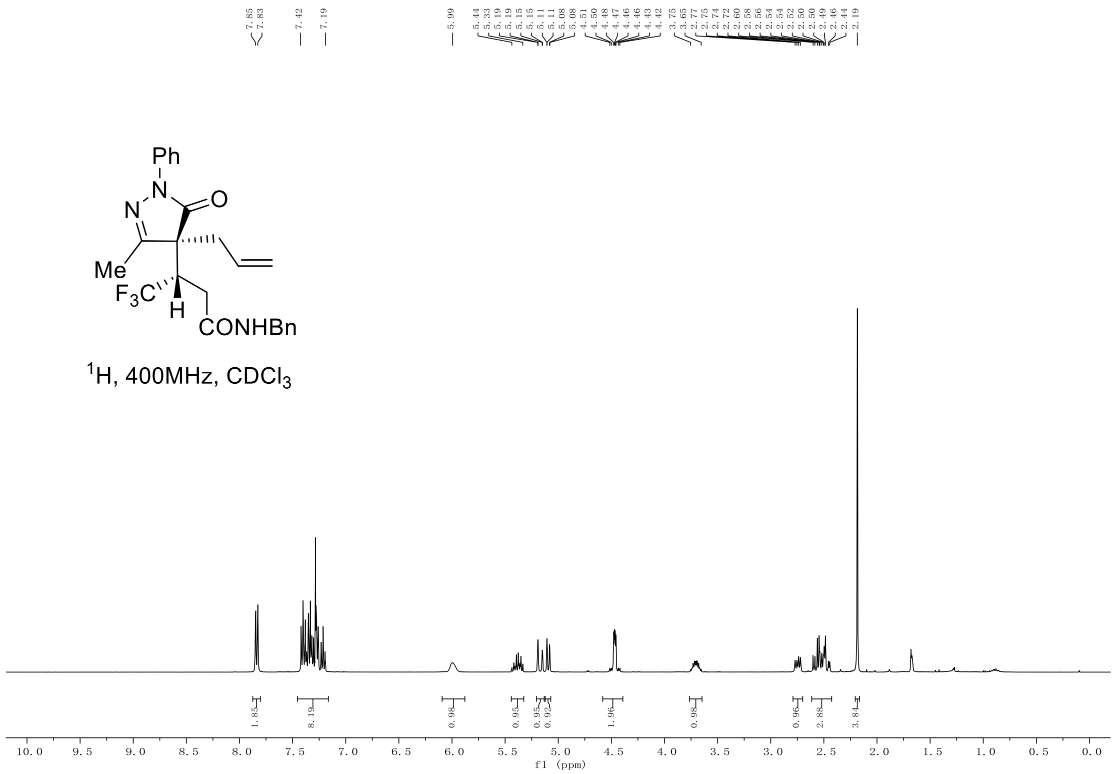

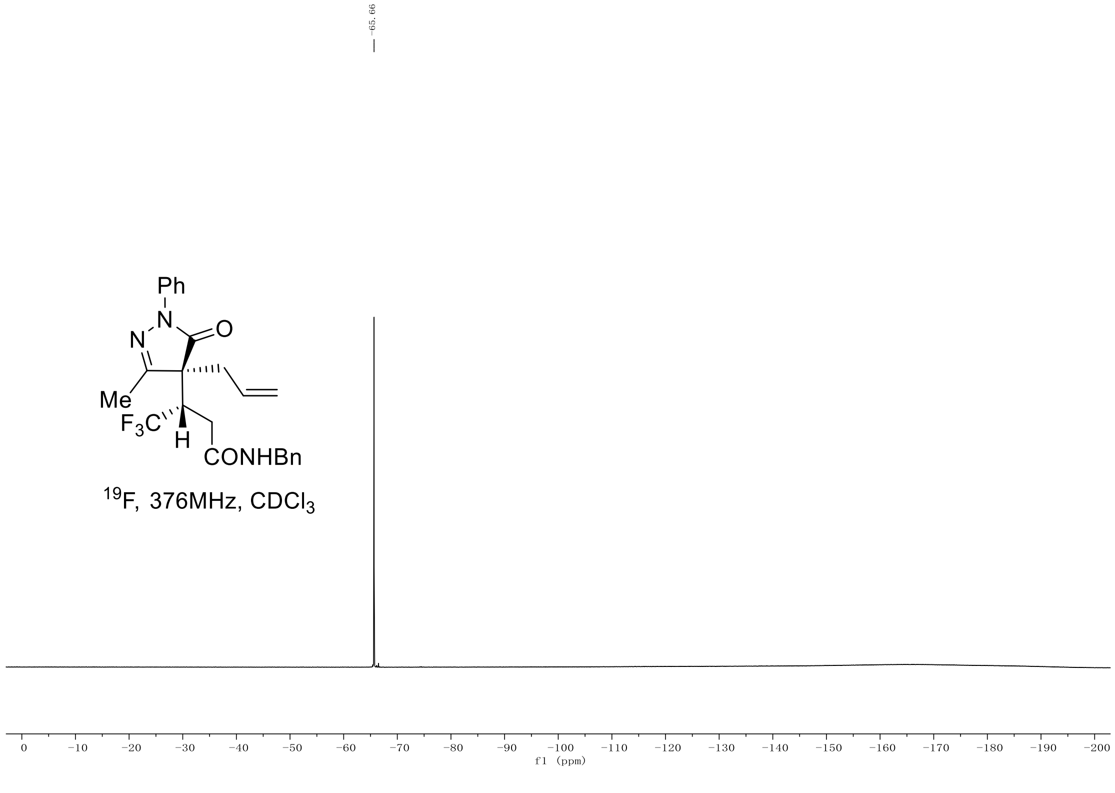

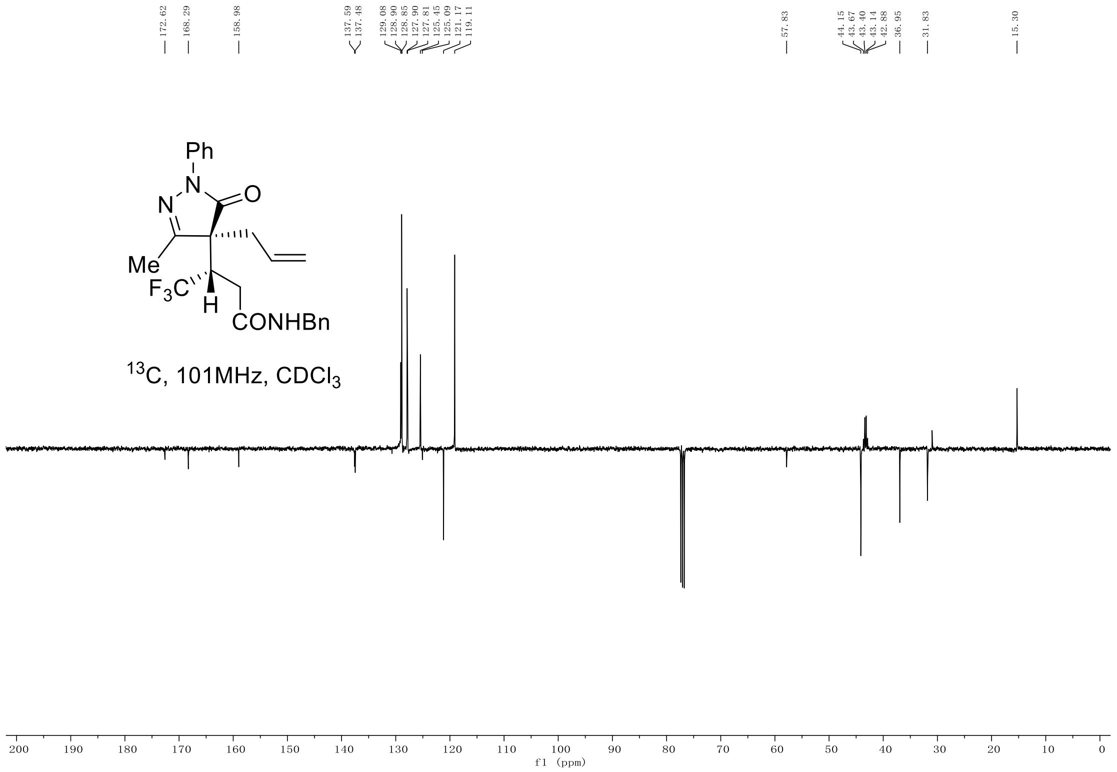

(*S*)-3-((*R*)-4-allyl-3-methyl-5-oxo-1-phenyl-4,5-dihydro-1*H*-pyrazol-4-yl)-N-benzyl-4,4,4-trifluorobutanamide (28$_{minor}$)

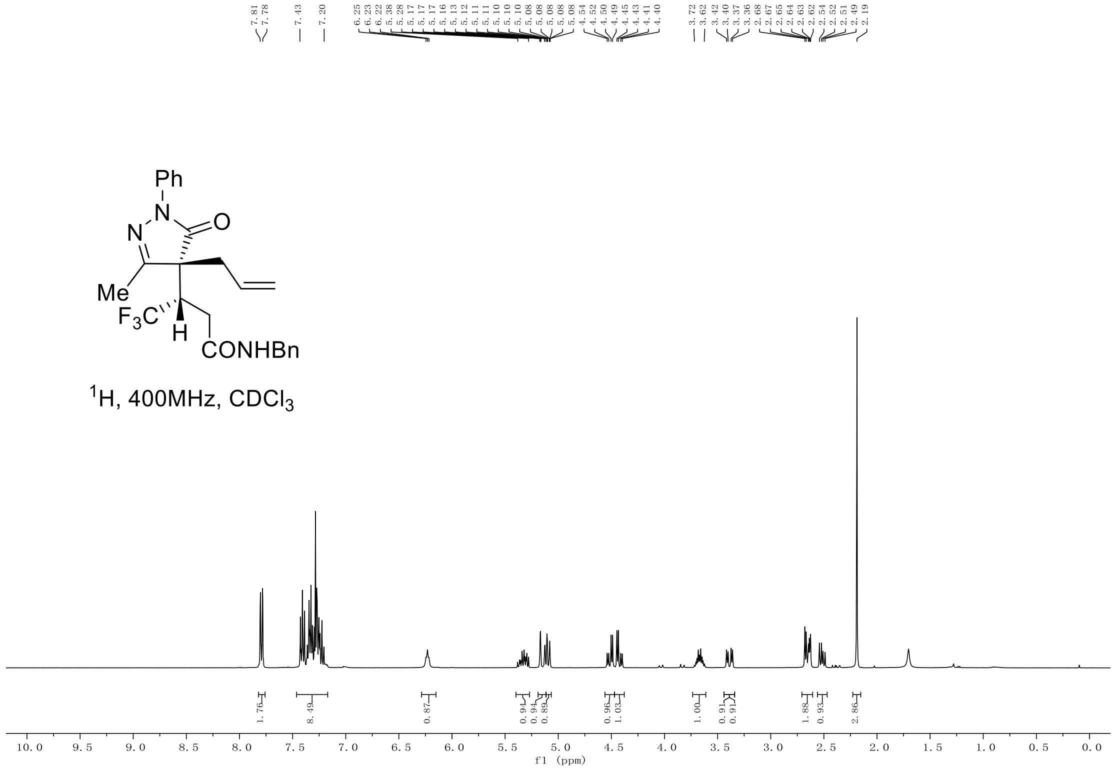

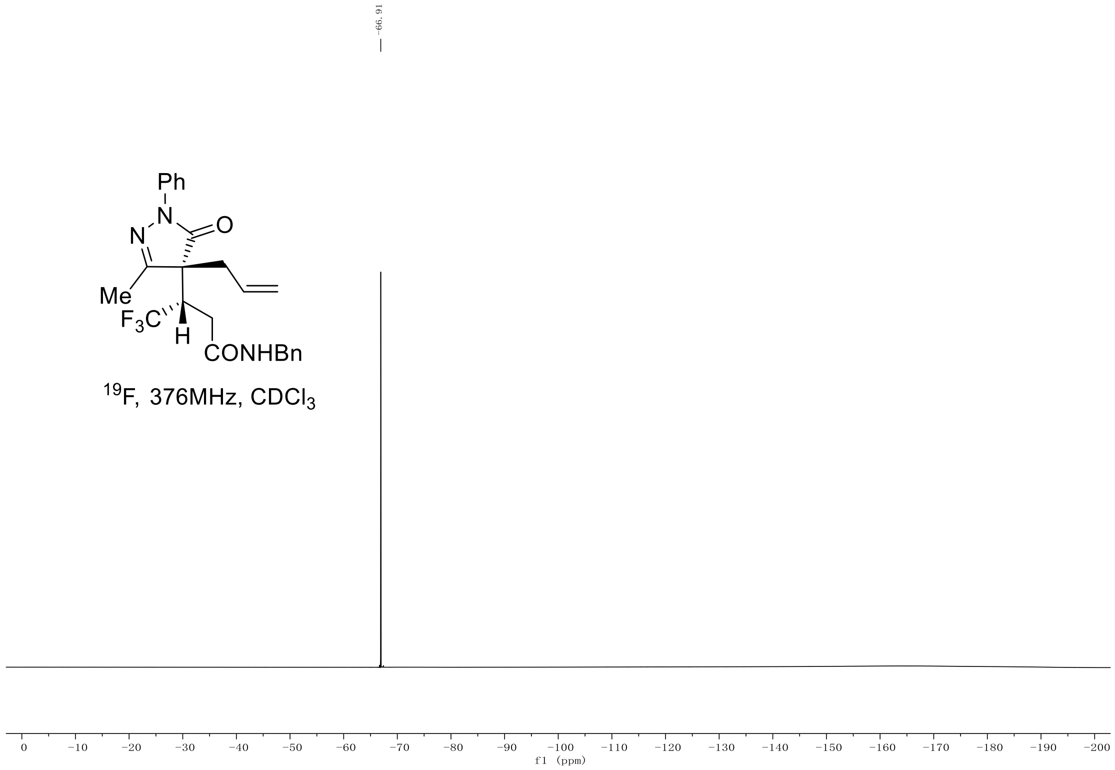
-66.91
Ph
N
N
O
Me
F_3C
H
CONHBn
^{19}F, 376MHz, $CDCl_3$
f1 (ppm)

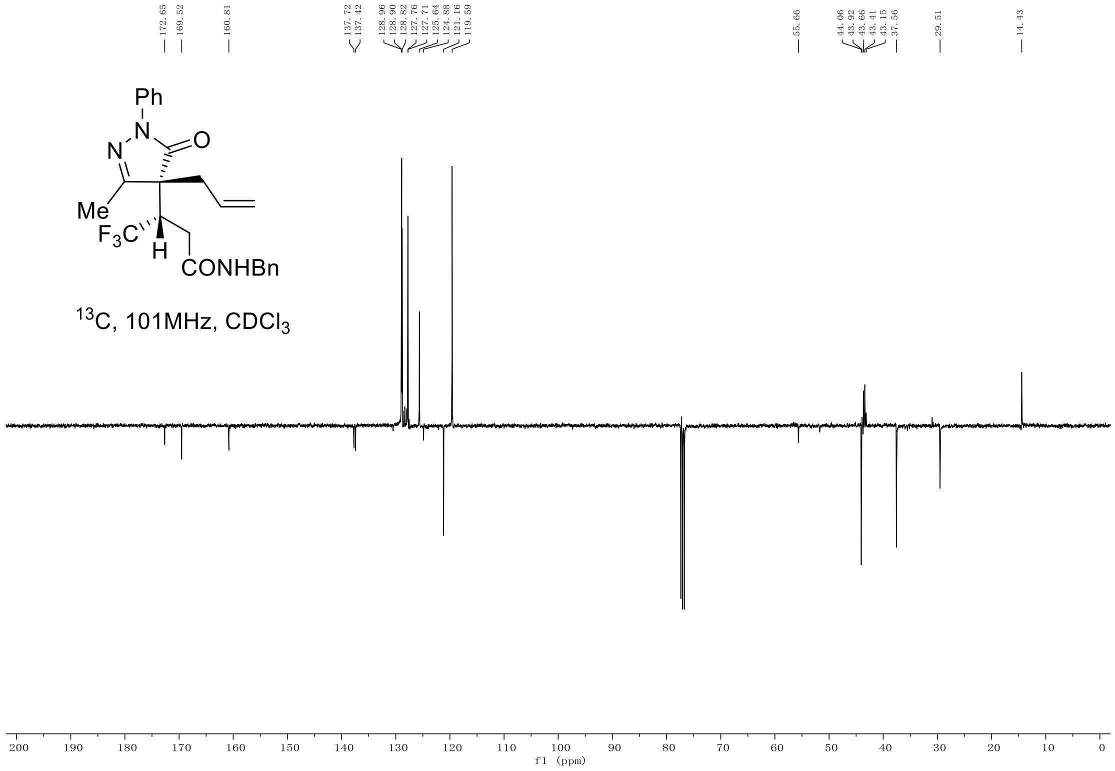
172.65
169.52
160.81
137.72
137.42
128.96
128.90
128.82
127.76
127.71
125.64
124.88
121.16
119.59
55.66
44.06
43.92
43.66
43.41
43.15
37.56
29.51
14.43
Ph
N
N
O
Me
F_3C
H
CONHBn
^{13}C, 101MHz, $CDCl_3$
f1 (ppm)

(*S*)-*N*-benzyl-3-((*S*)-4-benzyl-3-methyl-5-oxo-1-phenyl-4,5-dihydro-1*H*-pyrazol-4-yl)-4,4,4-trifluorobutanamide (29$_{major}$)

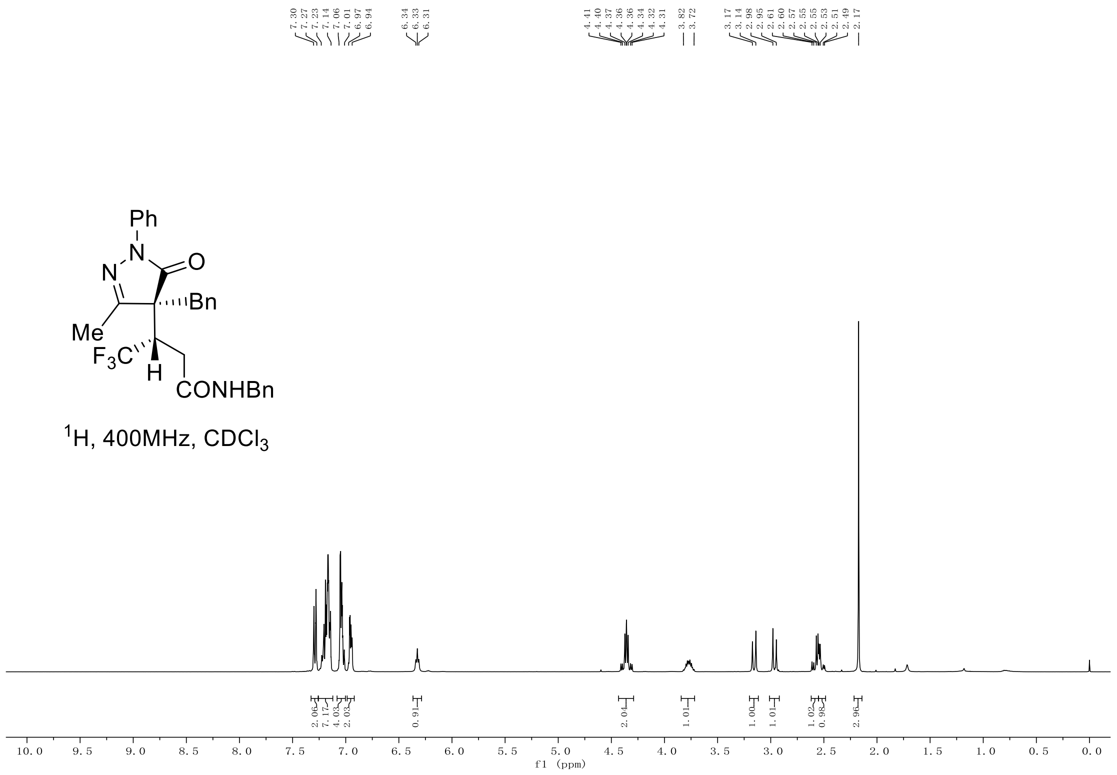

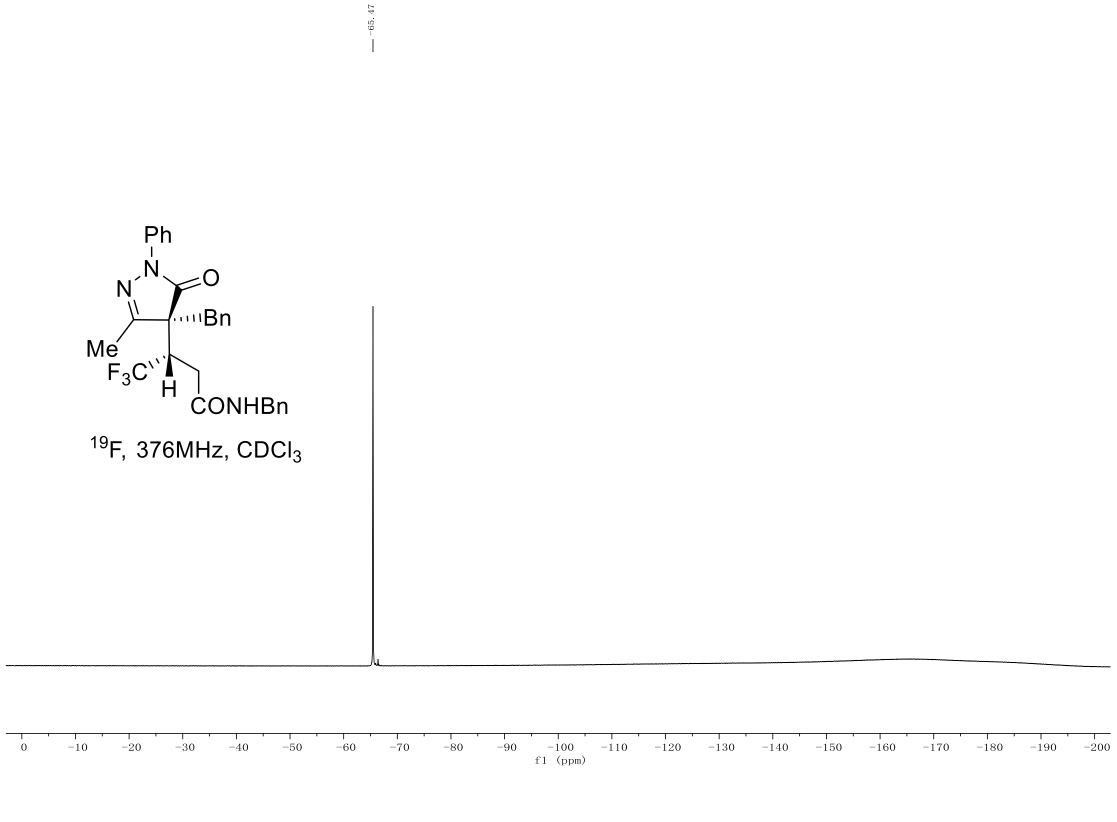

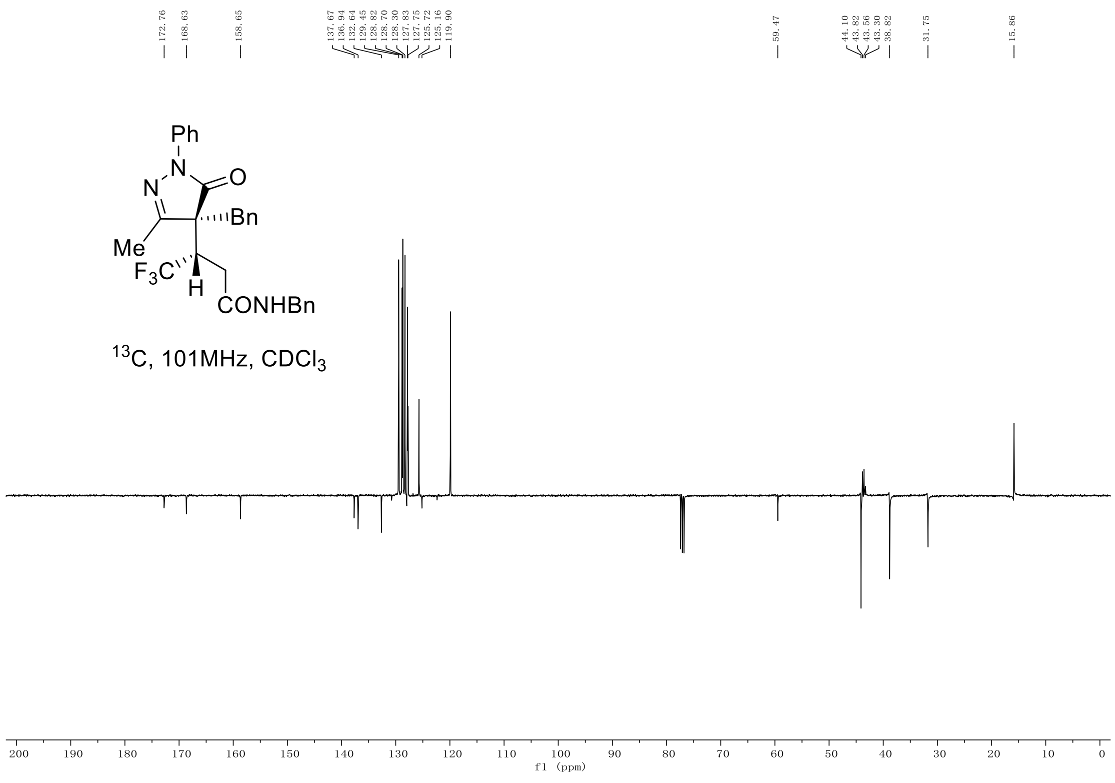

(*S*)-*N*-benzyl-3-((*R*)-4-benzyl-3-methyl-5-oxo-1-phenyl-4,5-dihydro-1*H*-pyrazol-4-yl)-4,4,4-trifluorobutanamide (29$_{\text{minor}}$)

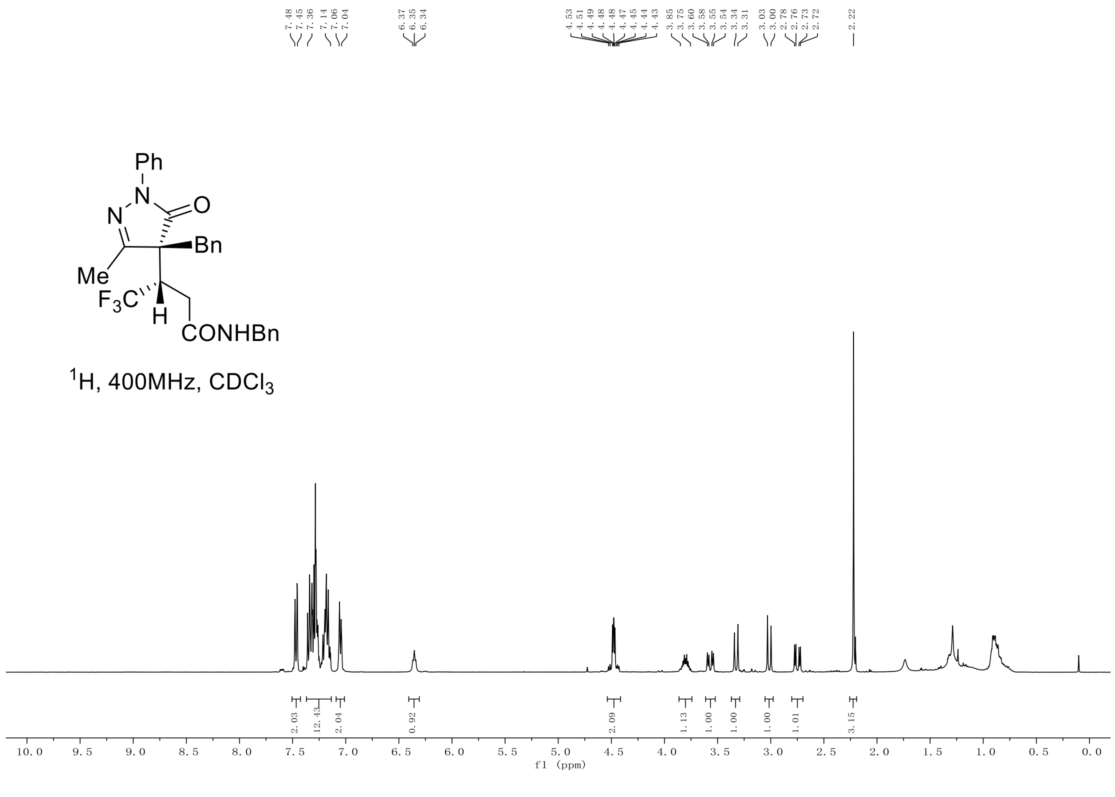

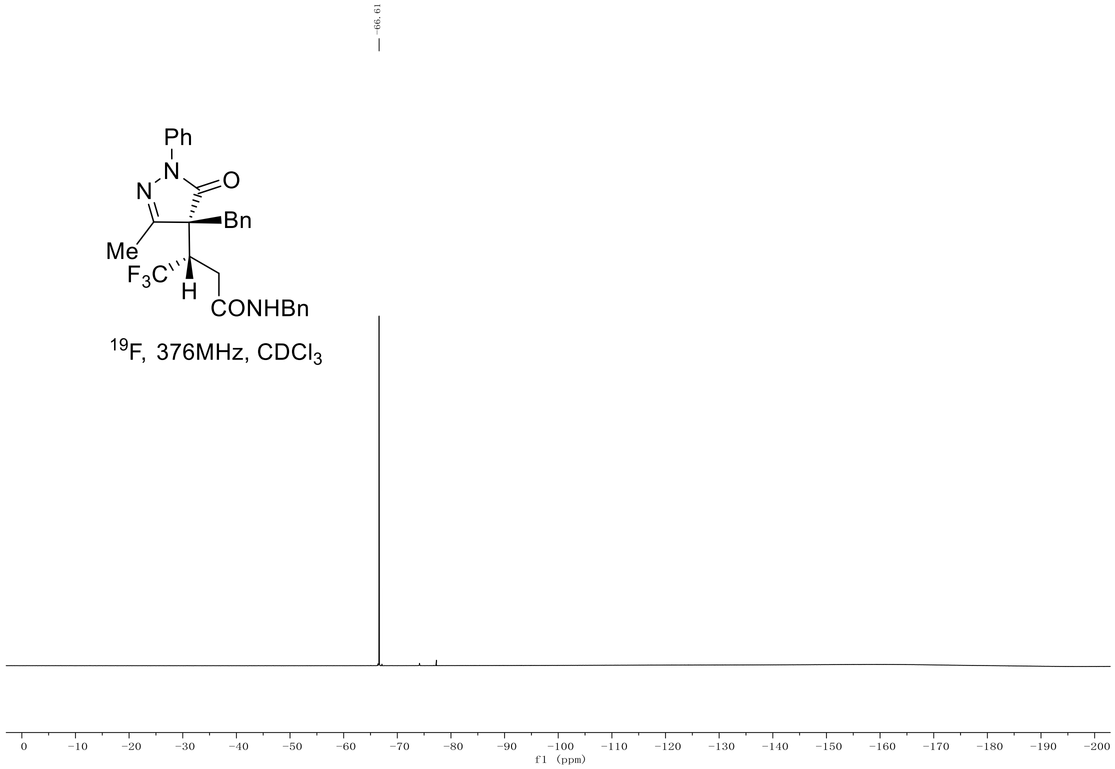

-66.61
Ph
N
N
O
Bn
Me
F3C
H
CONHBn
19F, 376MHz, CDCl3
f1 (ppm)

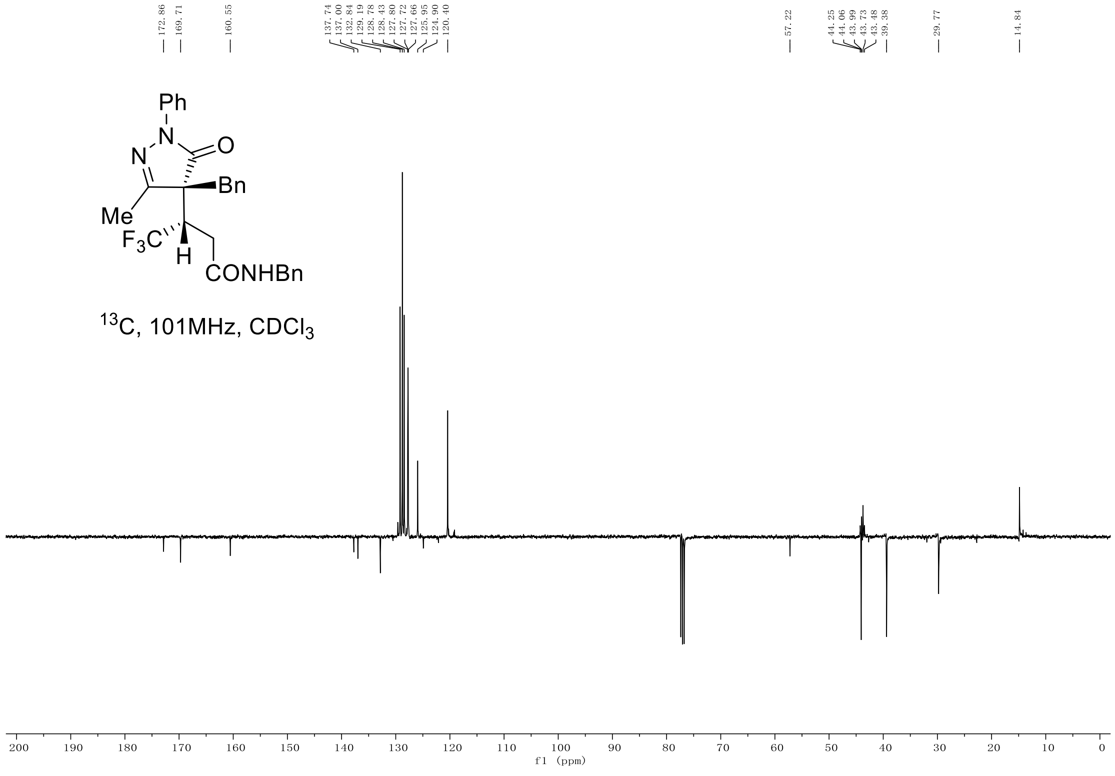

172.86
169.71
160.55
137.74
137.00
132.84
129.19
128.78
128.43
127.80
127.72
127.66
125.95
124.90
120.40
57.22
44.25
44.06
43.99
43.73
43.48
39.38
29.77
14.84
Ph
N
N
O
Bn
Me
F3C
H
CONHBn
13C, 101MHz, CDCl3
f1 (ppm)

(*S*)-*N*-benzyl-3-((*S*)-4-benzyl-5-oxo-1,3-diphenyl-4,5-dihydro-1*H*-pyrazol-4-yl)-4,4,4-trifluorobutanamide (30_{major})

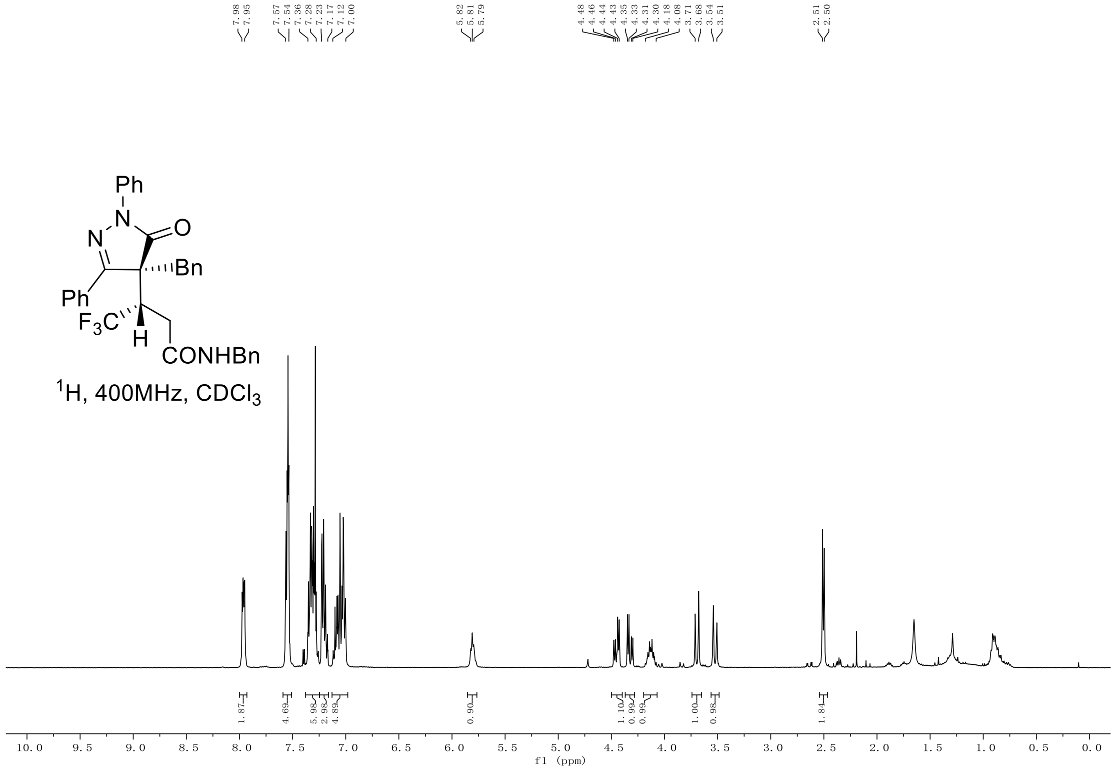

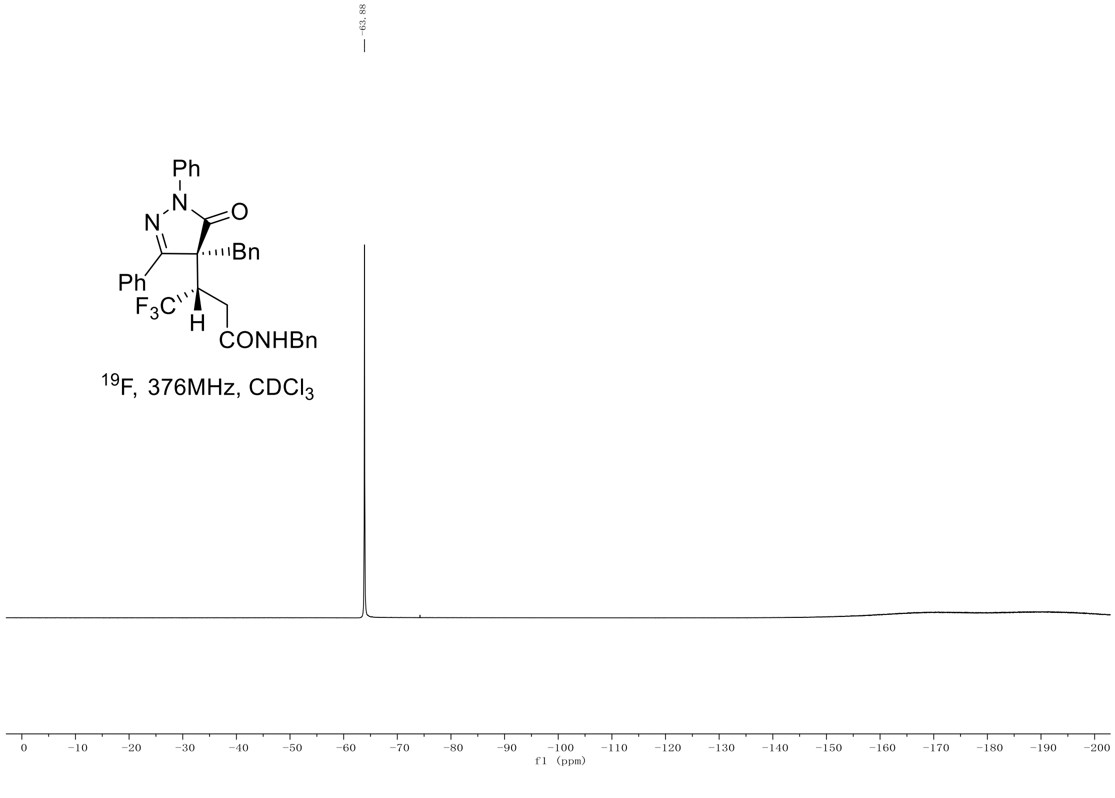

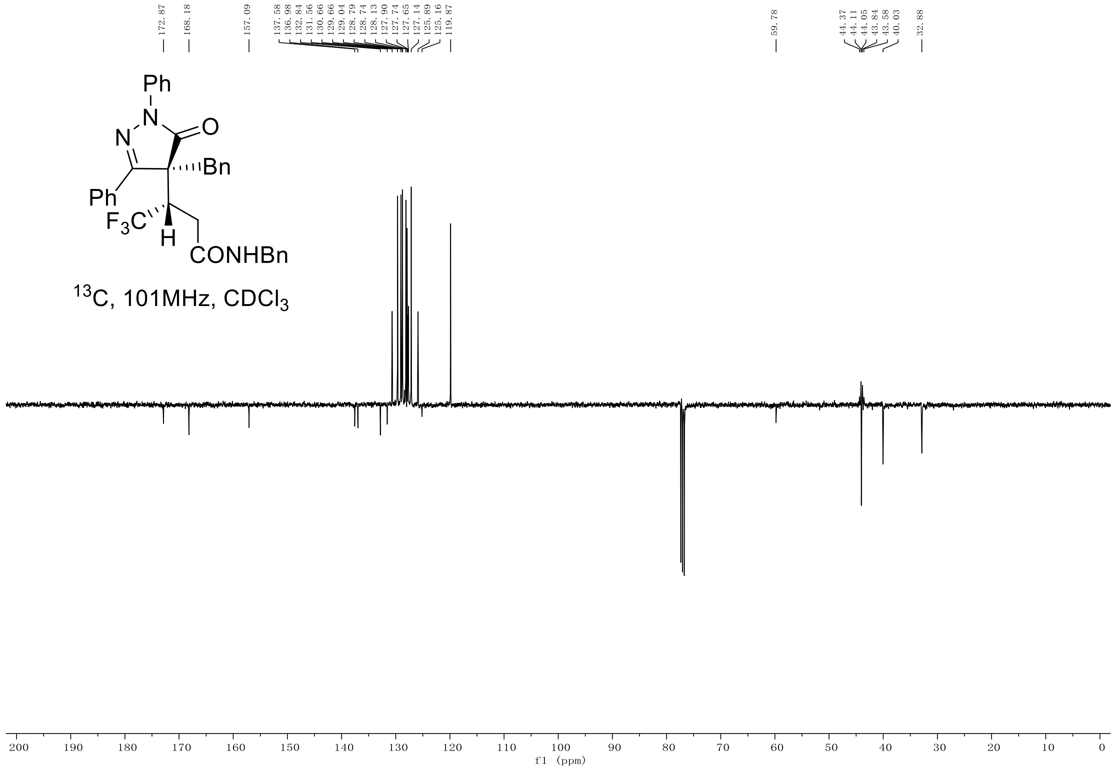

(*S*)-*N*-benzyl-3-((*R*)-4-benzyl-5-oxo-1,3-diphenyl-4,5-dihydro-1*H*-pyrazol-4-yl)-4,4,4-trifluorobutanamide (30_{minor})

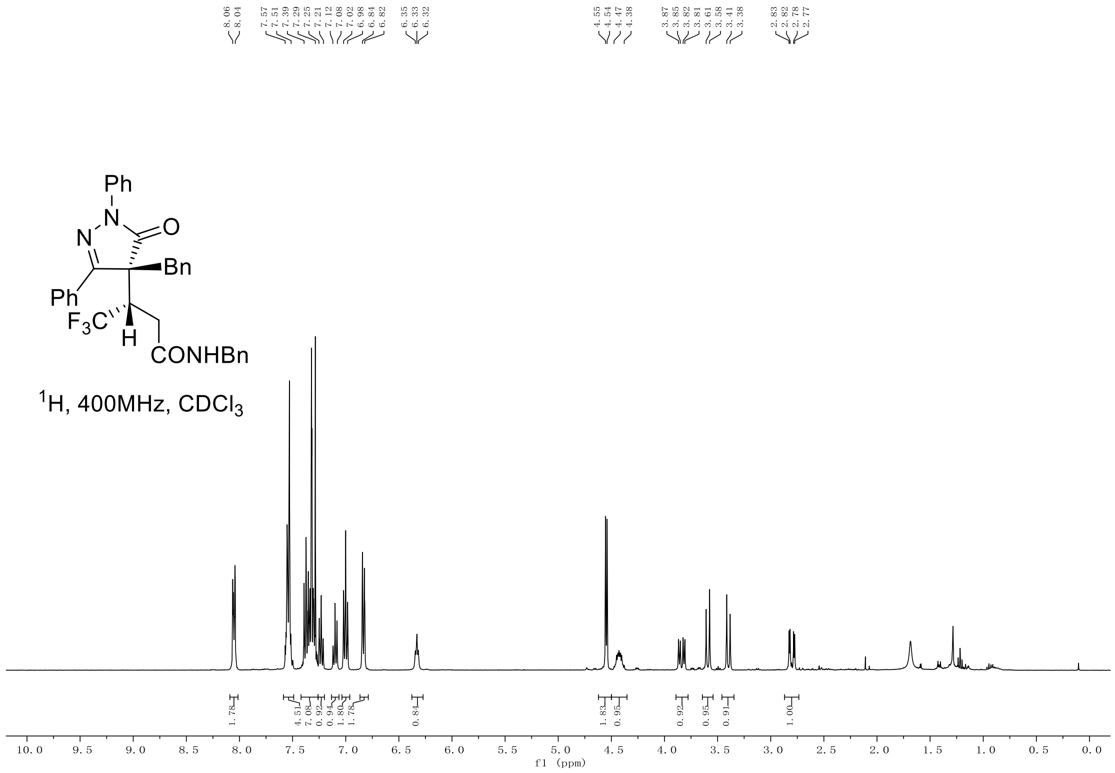

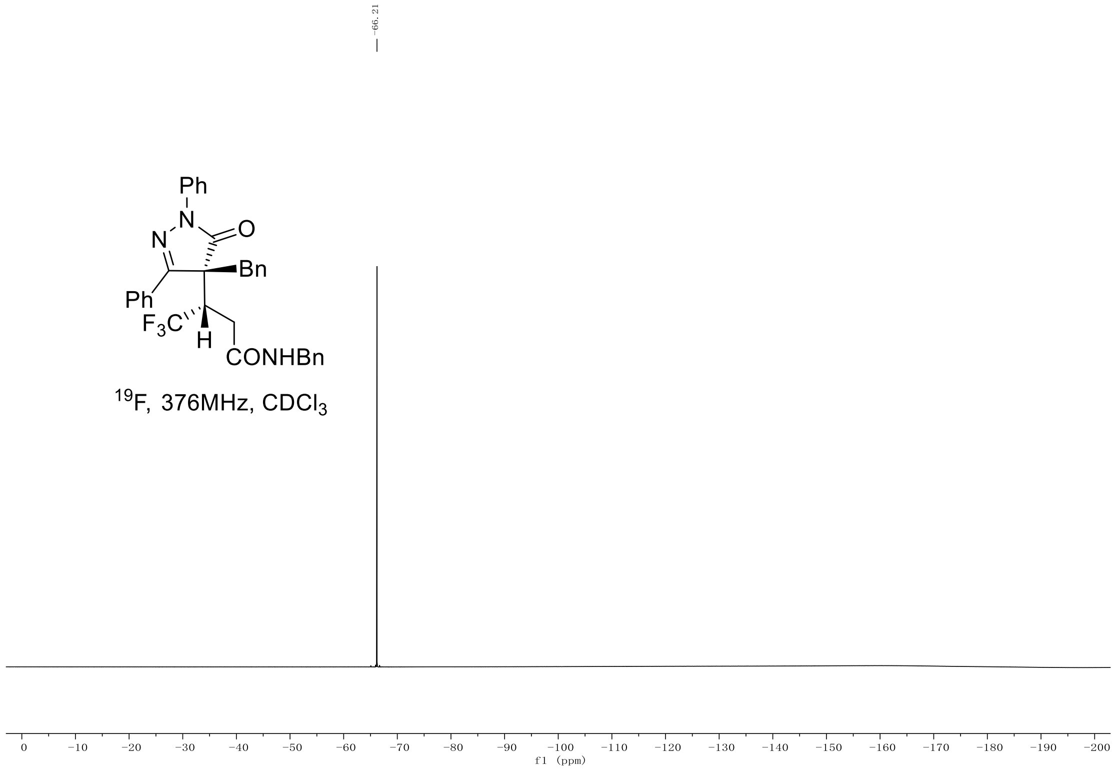

Ph
N
N
O
Bn
Ph
F3C
H
CONHBn
19F, 376MHz, CDCl3
-66.21
f1 (ppm)

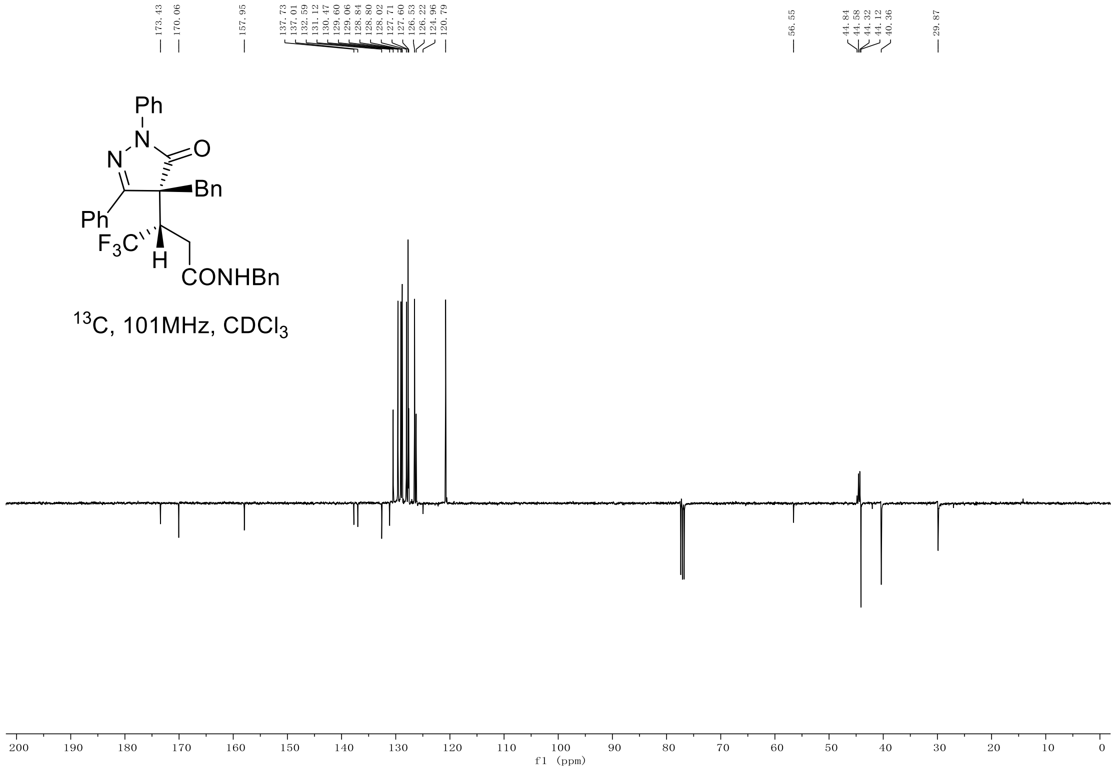

Ph
N
N
O
Bn
Ph
F3C
H
CONHBn
13C, 101MHz, CDCl3
173.43
170.06
157.95
137.73
137.01
132.59
131.12
130.47
129.60
129.06
128.84
128.80
128.02
127.71
127.60
126.53
126.22
124.96
120.79
56.55
44.84
44.58
44.32
44.12
40.36
29.87
f1 (ppm)

(*S*)-*N*-benzyl-4,4,4-trifluoro-3-((*S*)-3-methyl-4-(2-methylbenzyl)-5-oxo-1-phenyl-4,5-dihydro-1*H*-pyrazol-4-yl)butanamide (31_{major})

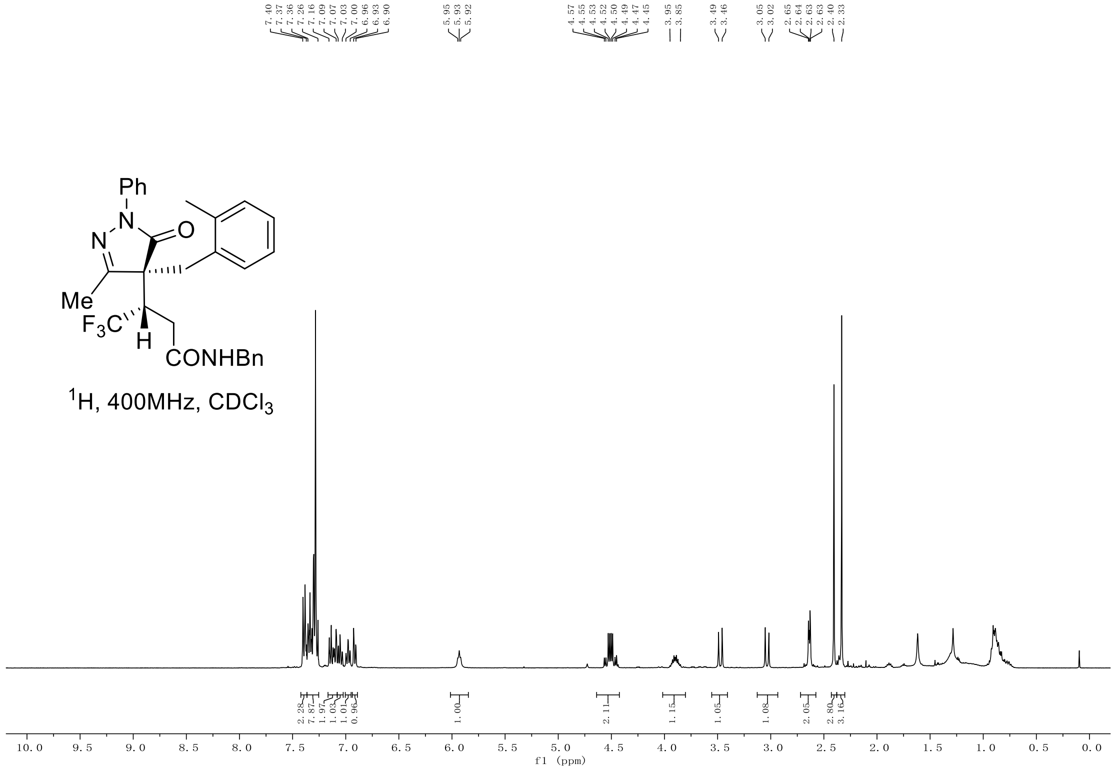

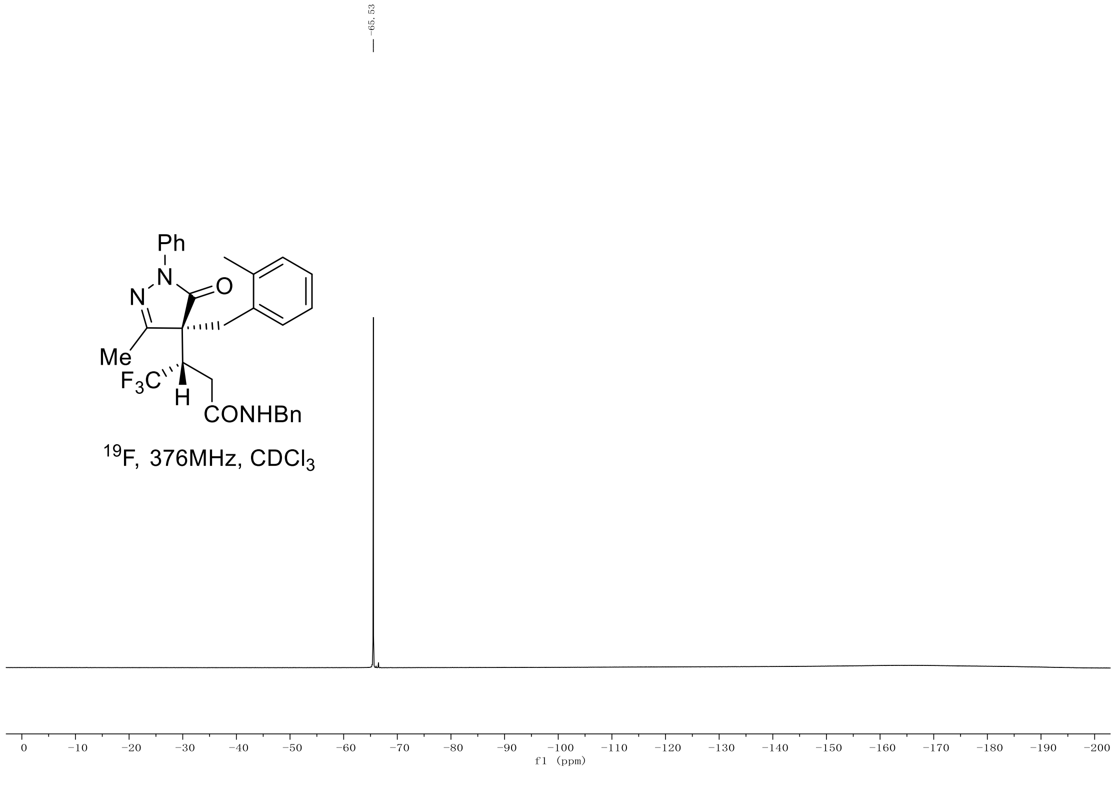

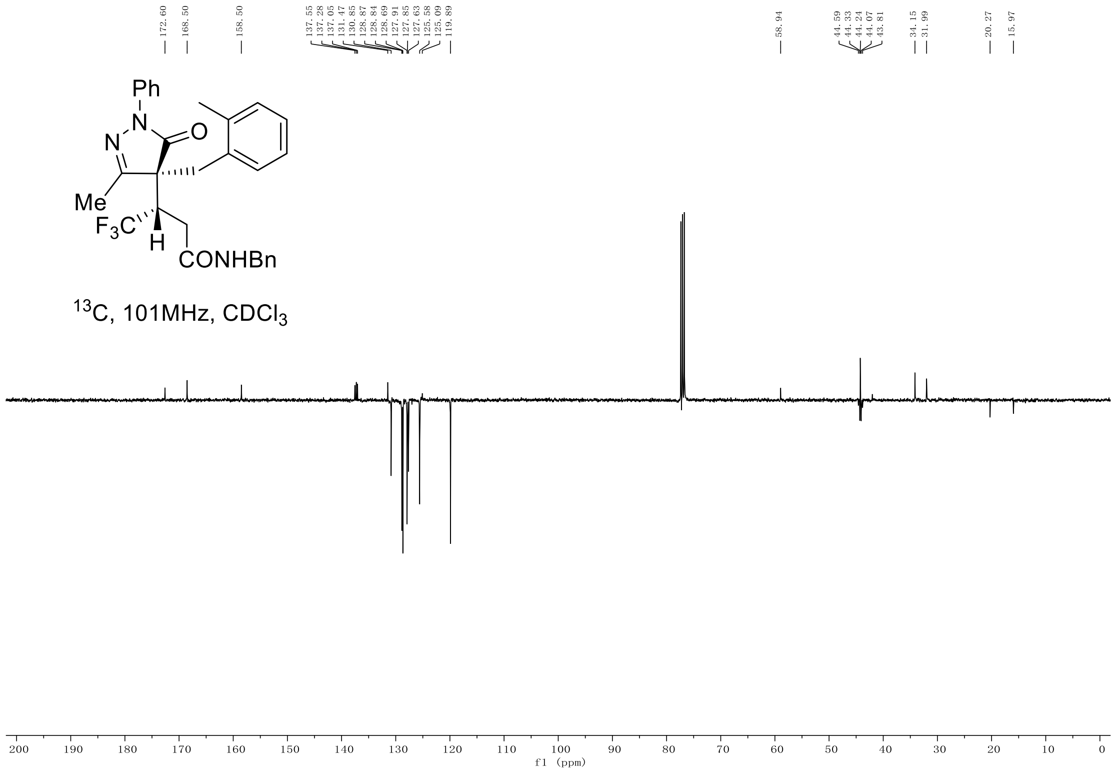

(*S*)-*N*-benzyl-4,4,4-trifluoro-3-((*R*)-3-methyl-4-(2-methylbenzyl)-5-oxo-1-phenyl-4,5-dihydro-1*H*-pyrazol-4-yl)butanamide (31$_{minor}$)

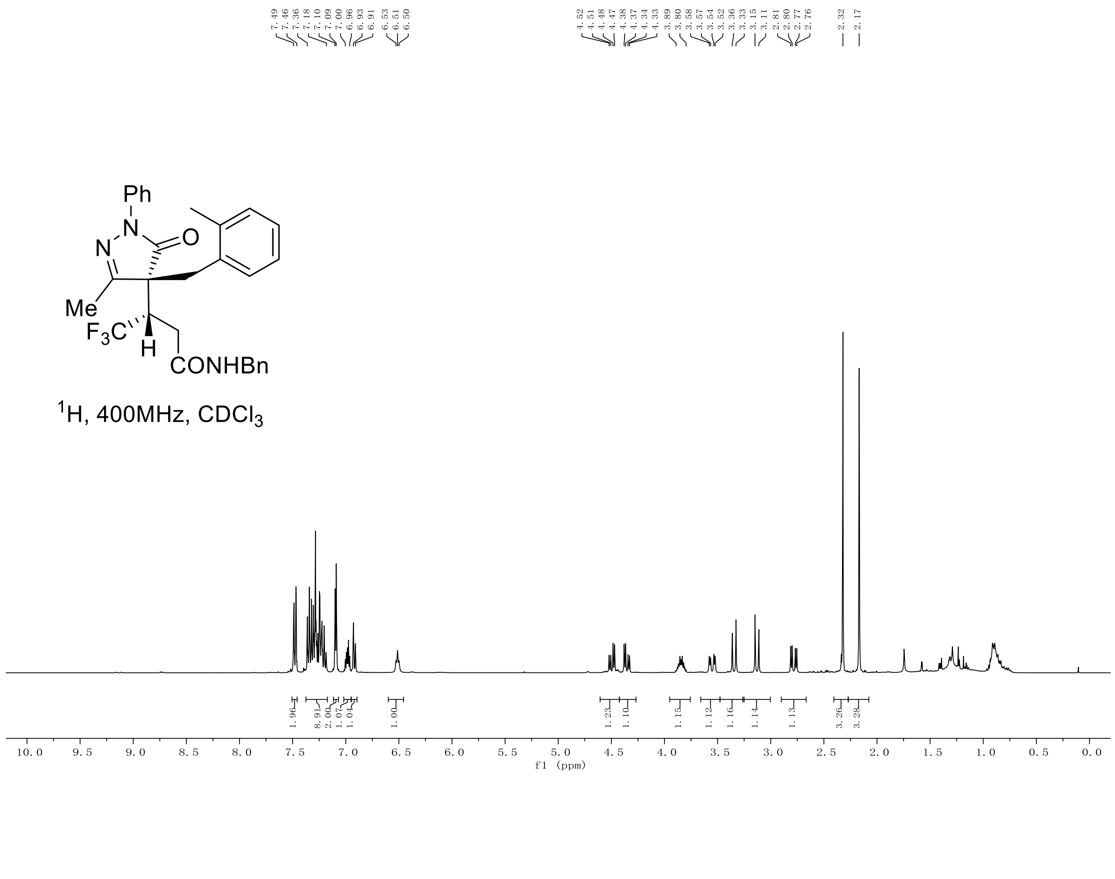

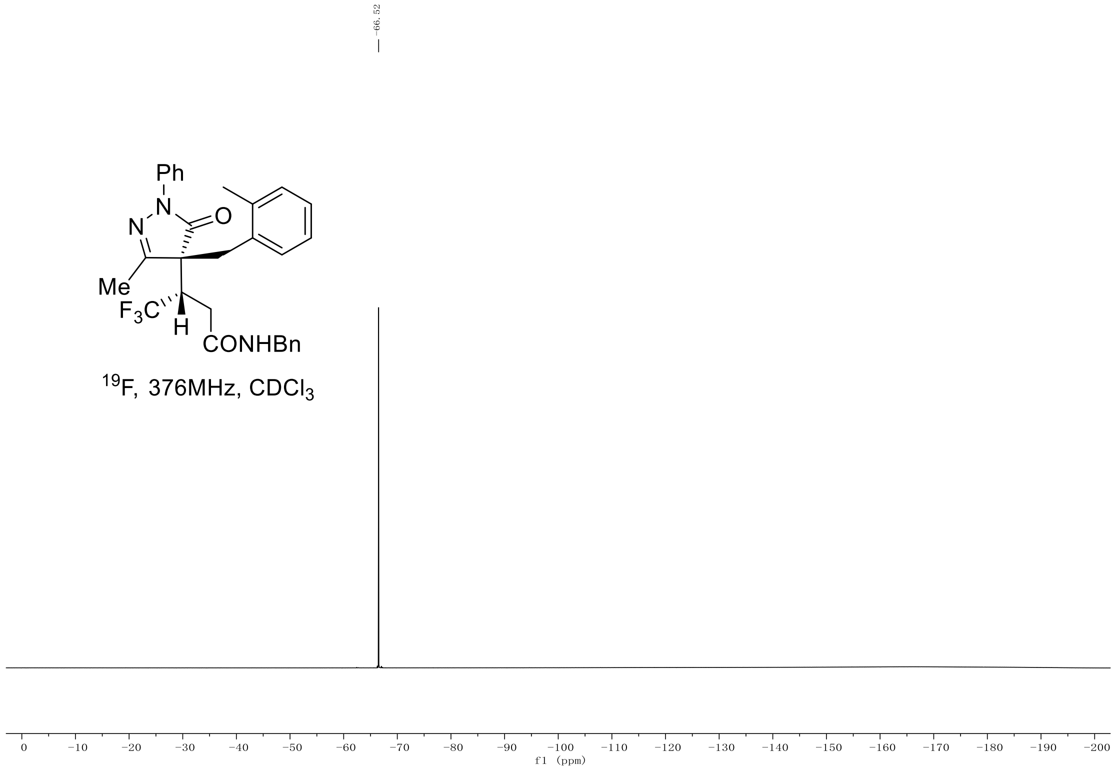
-66.52
Ph
N
N
O
Me
F_3C
H
CONHBn
^{19}F, 376MHz, $CDCl_3$
0 −10 −20 −30 −40 −50 −60 −70 −80 −90 −100 −110 −120 −130 −140 −150 −160 −170 −180 −190 −200
f1 (ppm)

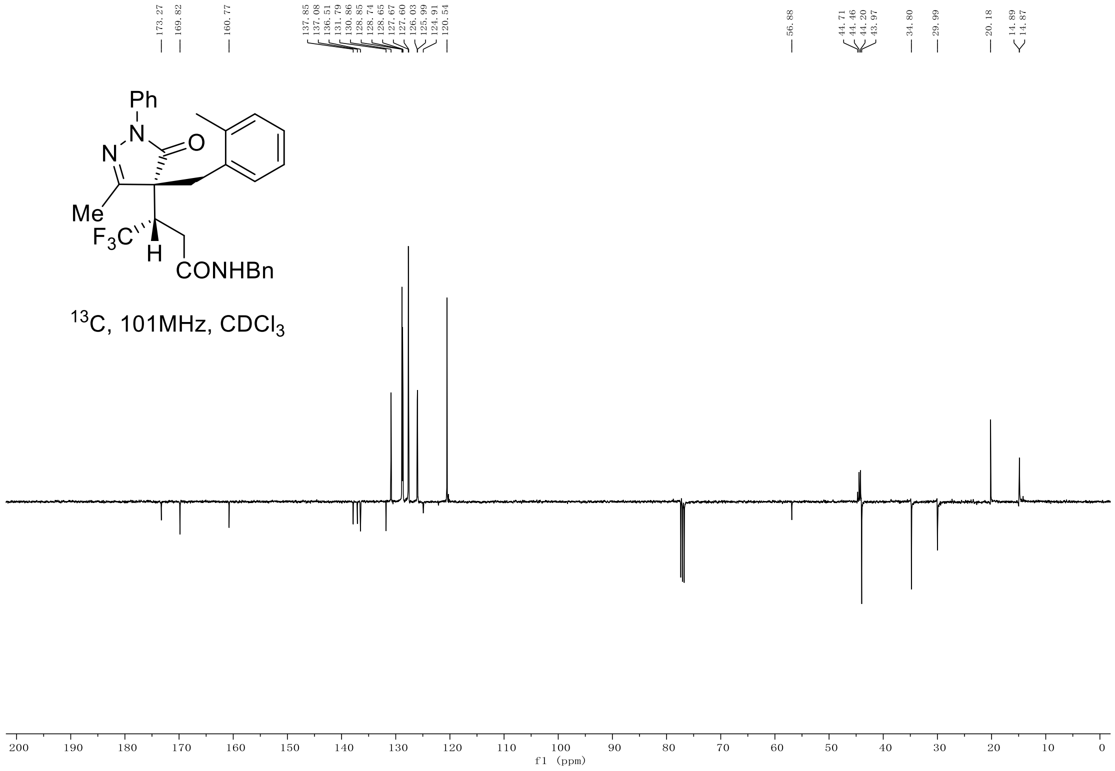
173.27
169.82
160.77
137.85
137.08
136.51
131.79
130.86
128.85
128.74
128.65
127.67
127.60
126.03
125.99
124.91
120.54
56.88
44.71
44.46
44.20
43.97
34.80
29.99
20.18
14.89
14.87
Ph
N
N
O
Me
F_3C
H
CONHBn
^{13}C, 101MHz, $CDCl_3$
200 190 180 170 160 150 140 130 120 110 100 90 80 70 60 50 40 30 20 10 0
f1 (ppm)

(*S*)-*N*-benzyl-4,4,4-trifluoro-3-((*S*)-3-methyl-4-(3-methylbenzyl)-5-oxo-1-phenyl-4,5-dihydro-1*H*-pyrazol-4-yl)butanamide (32$_{major}$)

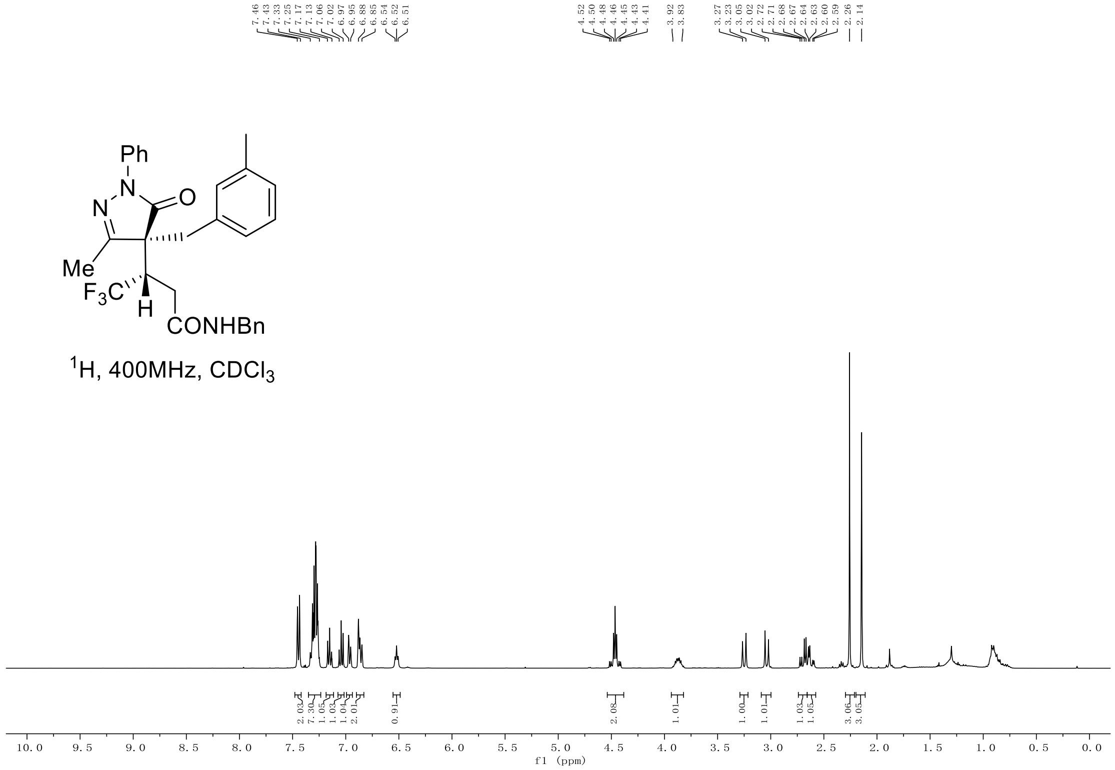

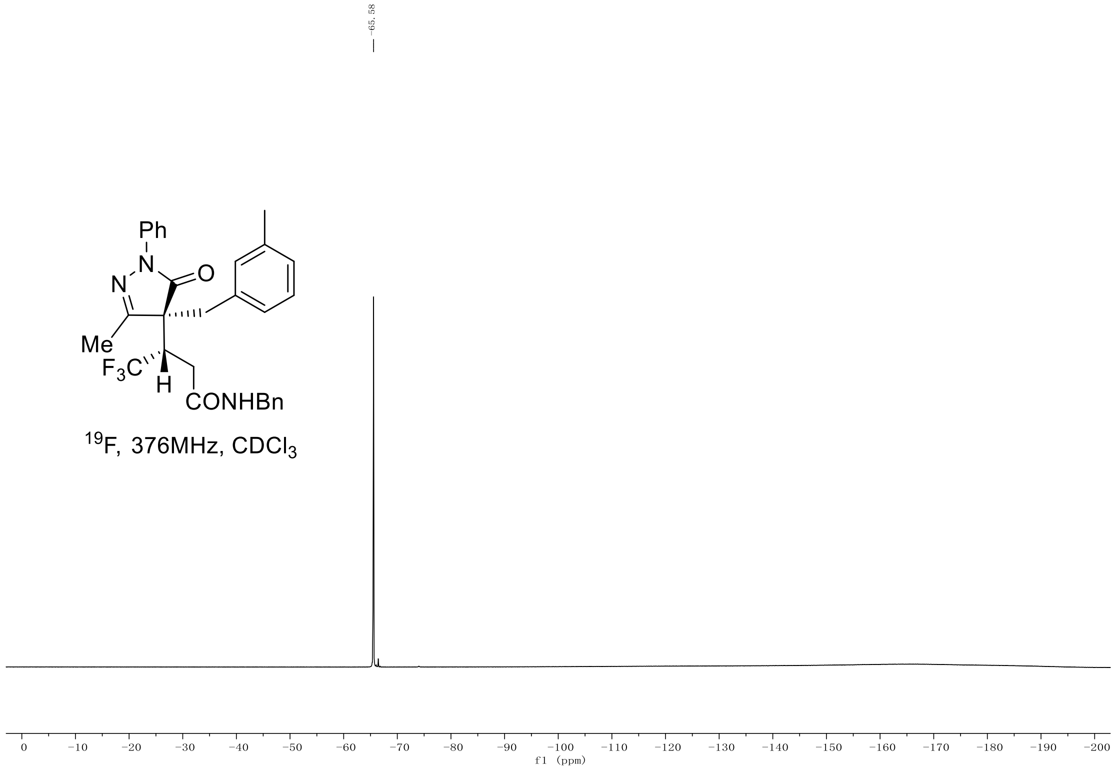

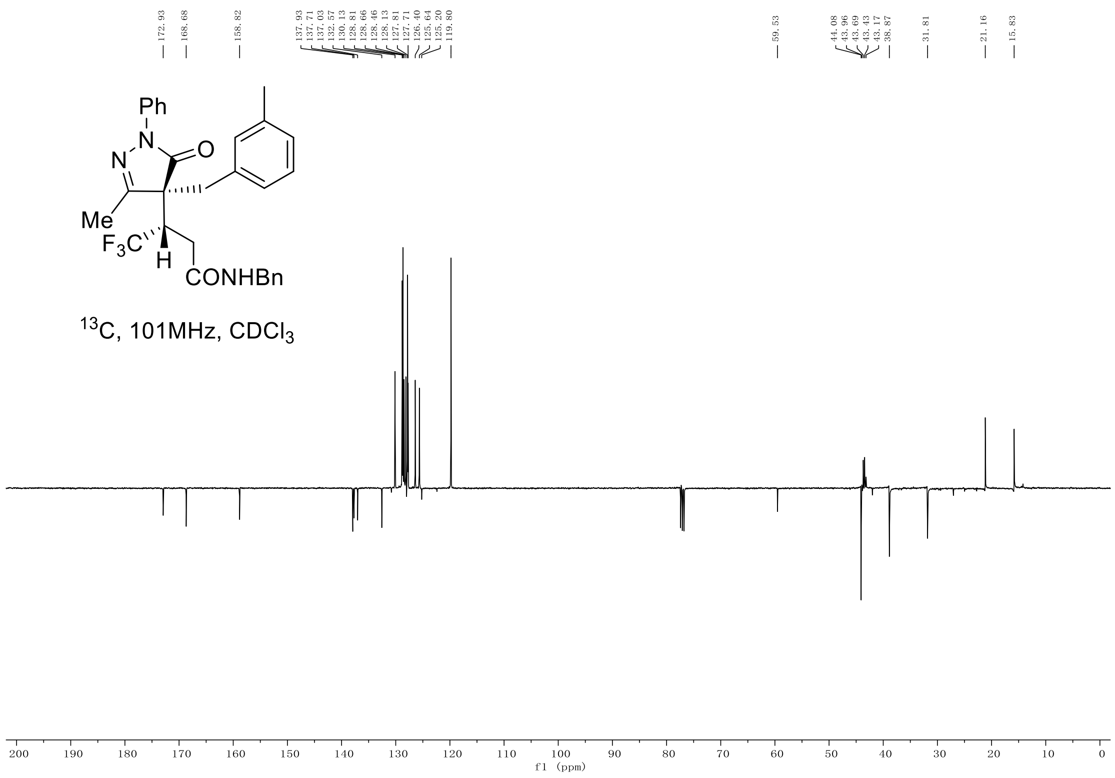

(*S*)-*N*-benzyl-4,4,4-trifluoro-3-((*R*)-3-methyl-4-(3-methylbenzyl)-5-oxo-1-phenyl-4,5-dihydro-1*H*-pyrazol-4-yl)butanamide (32_{minor})

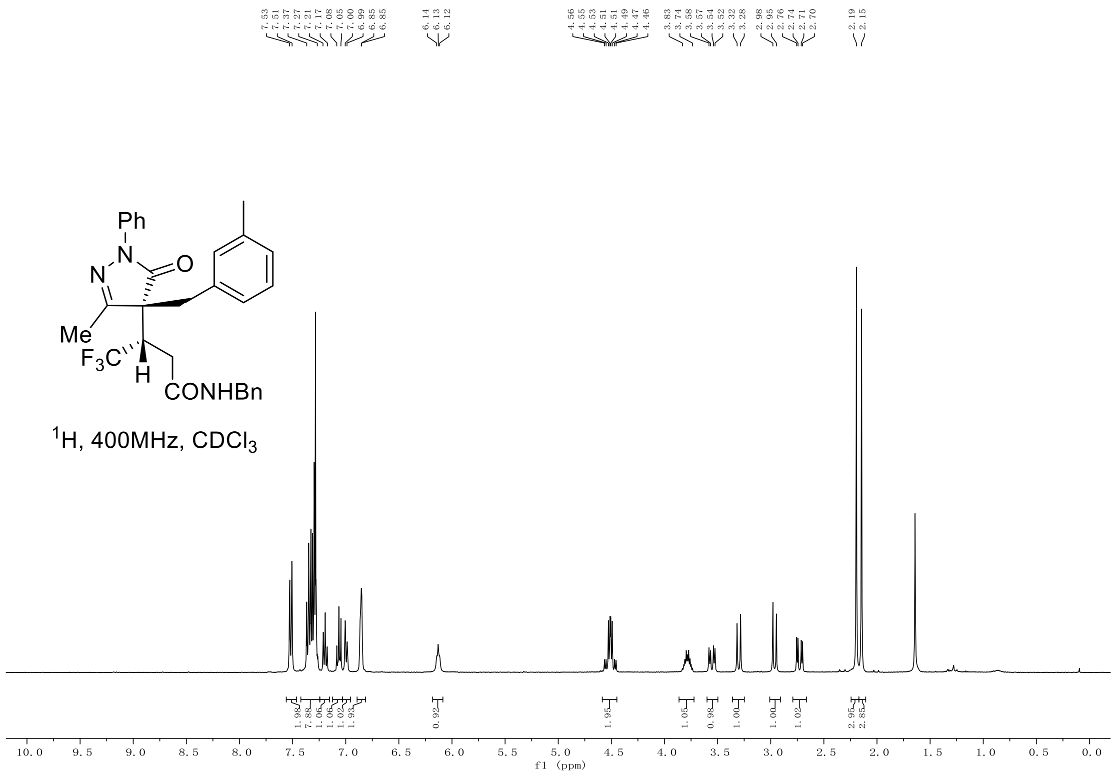

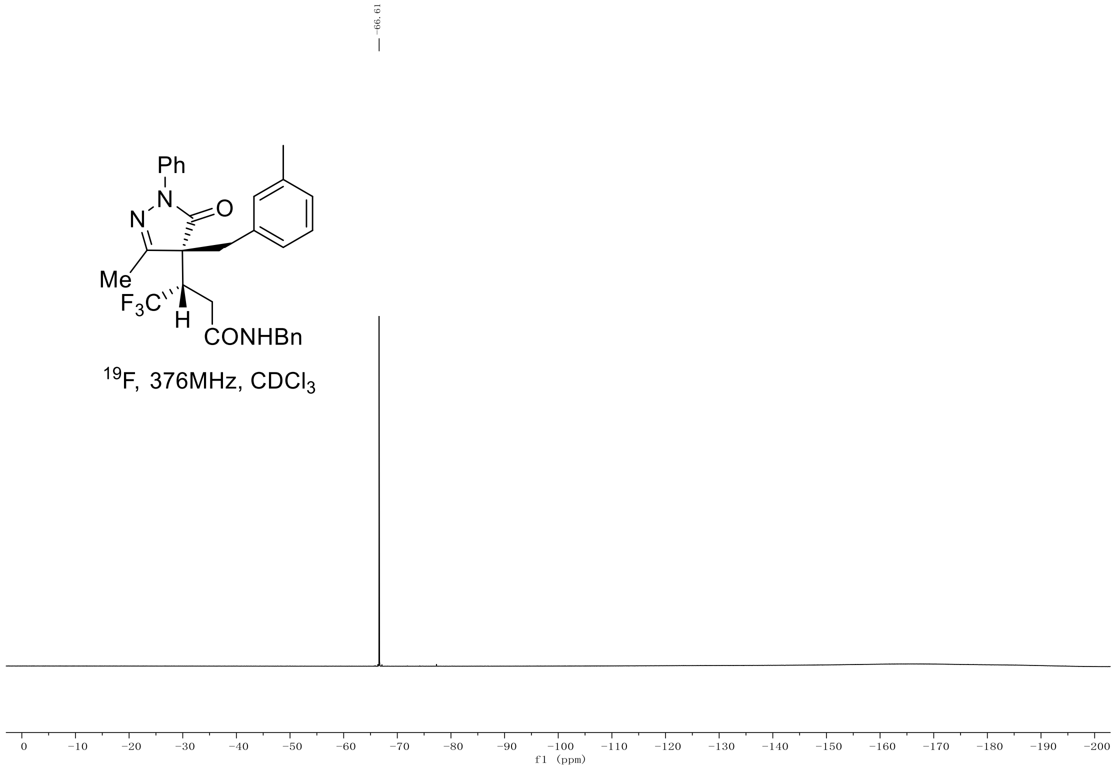

-66.61
Ph
N
N
O
Me
F3C
H
CONHBn
19F, 376MHz, CDCl3
0 -10 -20 -30 -40 -50 -60 -70 -80 -90 -100 -110 -120 -130 -140 -150 -160 -170 -180 -190 -200
f1 (ppm)

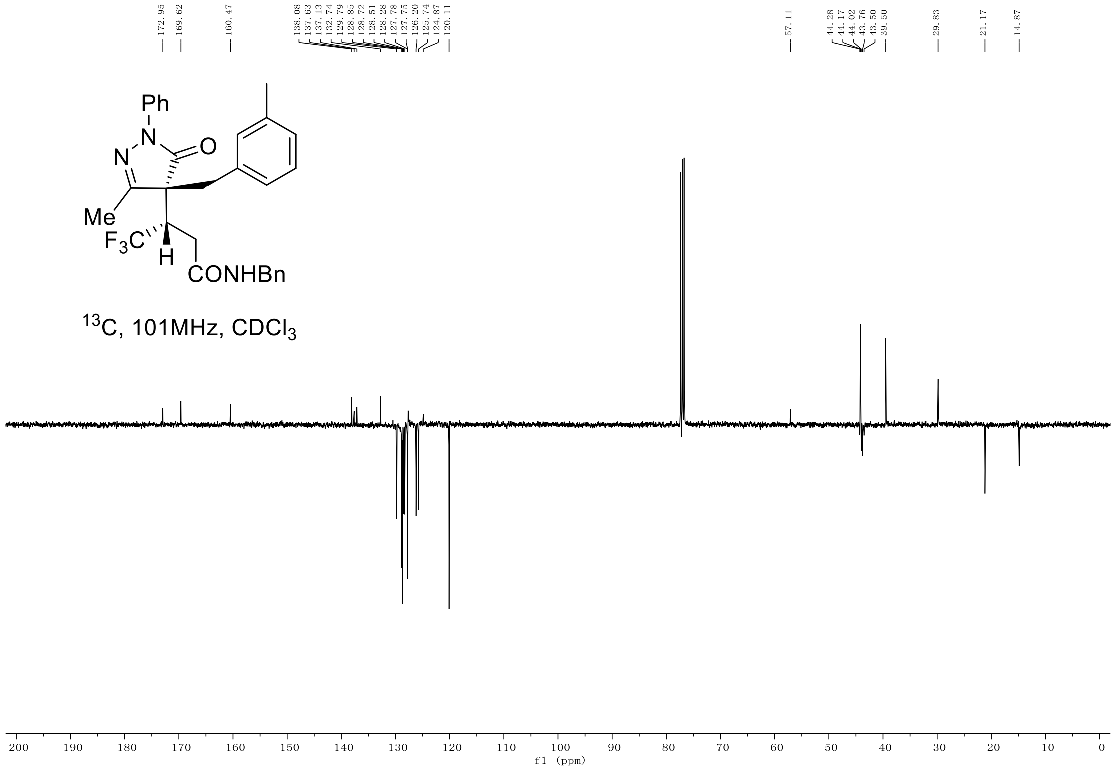

172.95
169.62
160.47
138.08
137.63
137.13
132.74
129.79
128.85
128.72
128.51
128.28
127.78
127.75
126.20
125.74
124.87
120.11
57.11
44.28
44.17
44.02
43.76
43.50
39.50
29.83
21.17
14.87
Ph
N
N
O
Me
F3C
H
CONHBn
13C, 101MHz, CDCl3
200 190 180 170 160 150 140 130 120 110 100 90 80 70 60 50 40 30 20 10 0
f1 (ppm)

(*S*)-*N*-benzyl-4,4,4-trifluoro-3-((*S*)-4-(4-chlorobenzyl)-3-methyl-5-oxo-1-phenyl-4,5-dihydro-1*H*-pyrazol-4-yl)butanamide (33$_{\mathrm{major}}$)

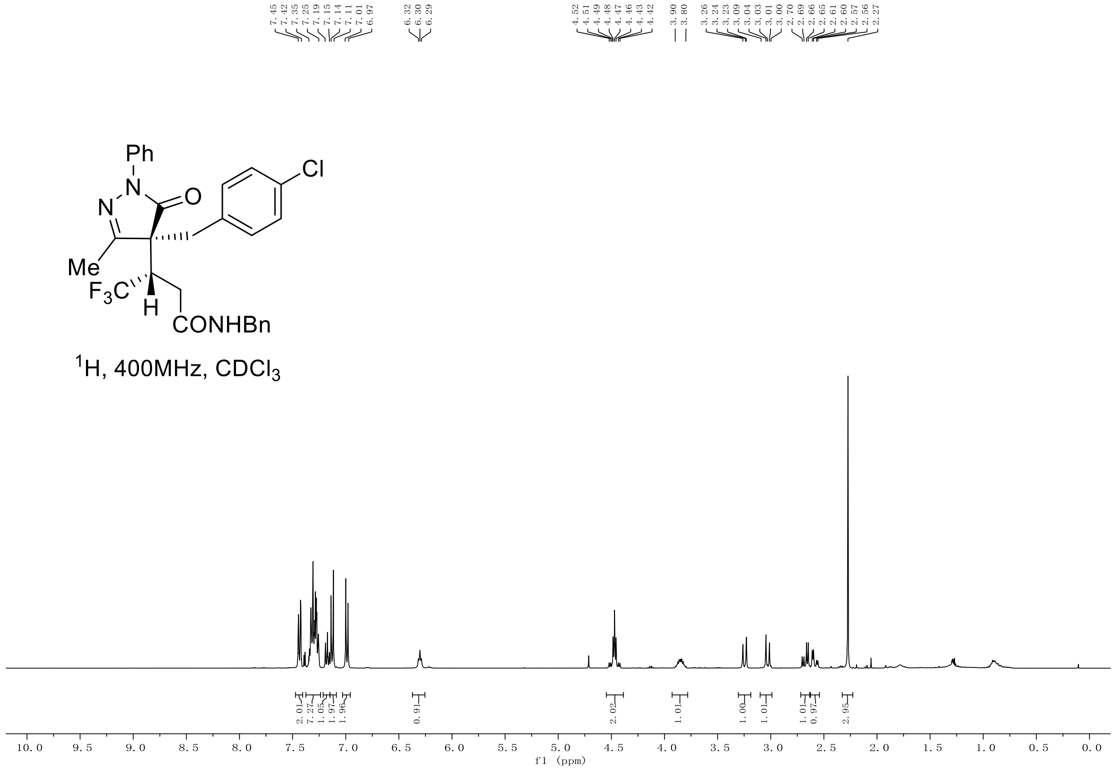

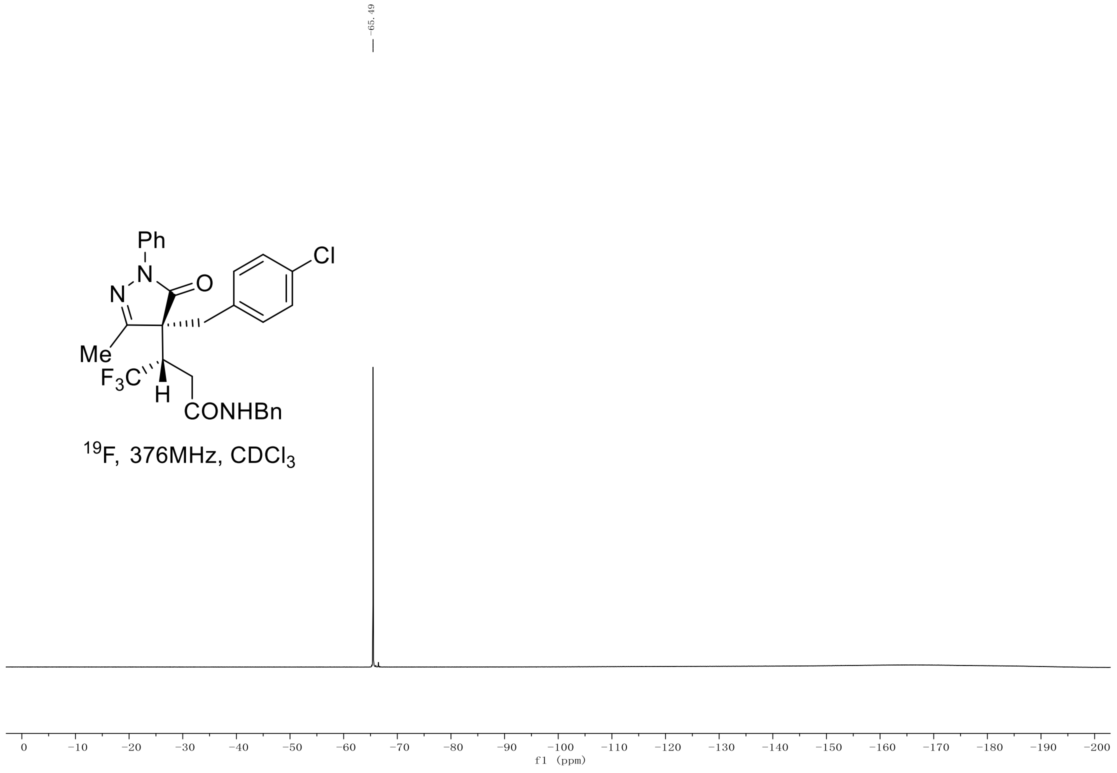

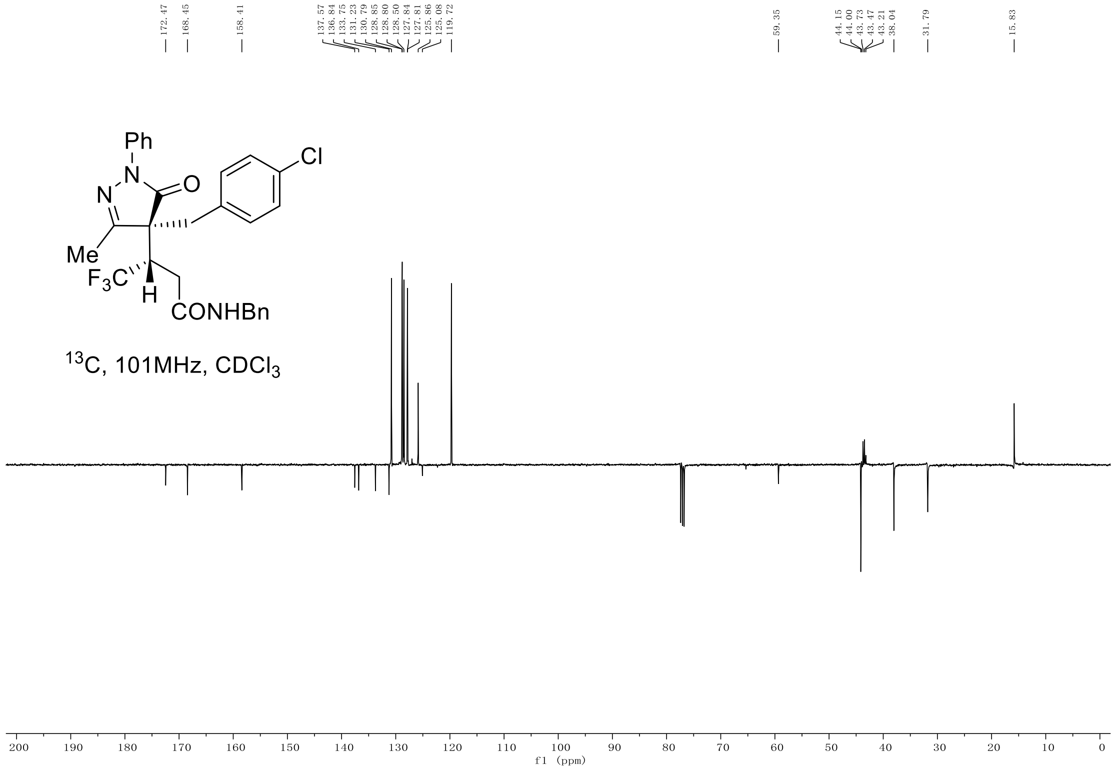

(*S*)-*N*-benzyl-4,4,4-trifluoro-3-((*R*)-4-(4-chlorobenzyl)-3-methyl-5-oxo-1-phenyl-4,5-dihydro-1*H*-pyrazol-4-yl)butanamide (33_{minor})

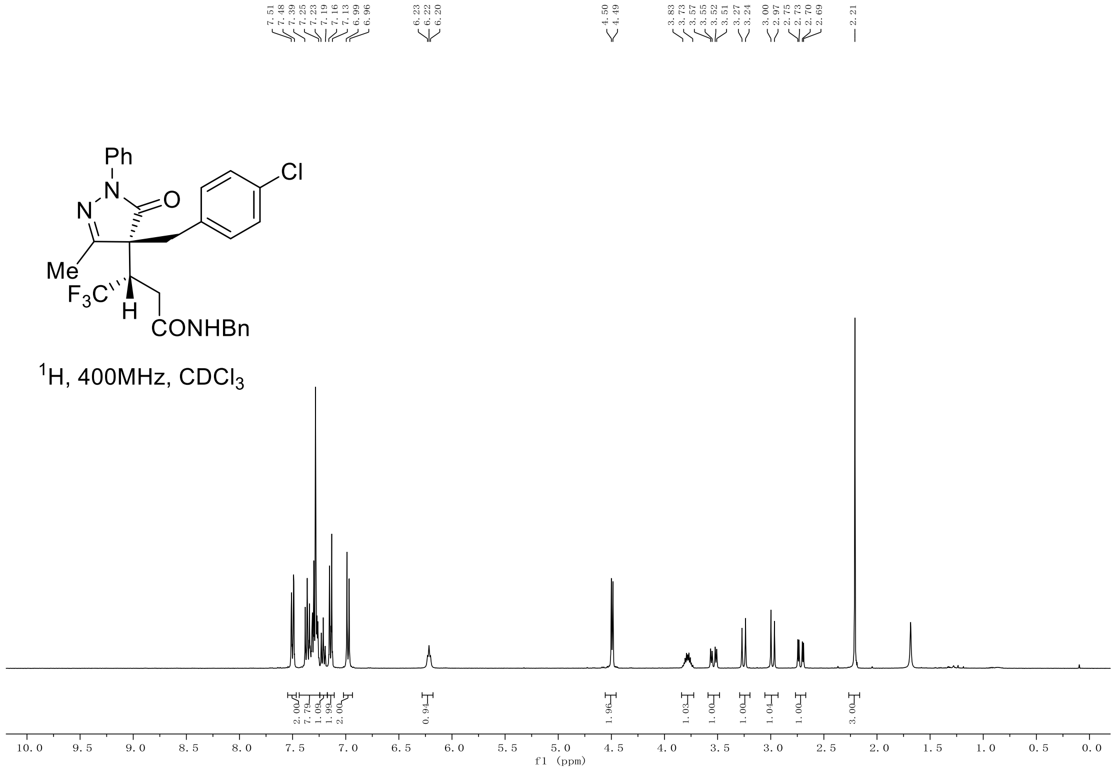

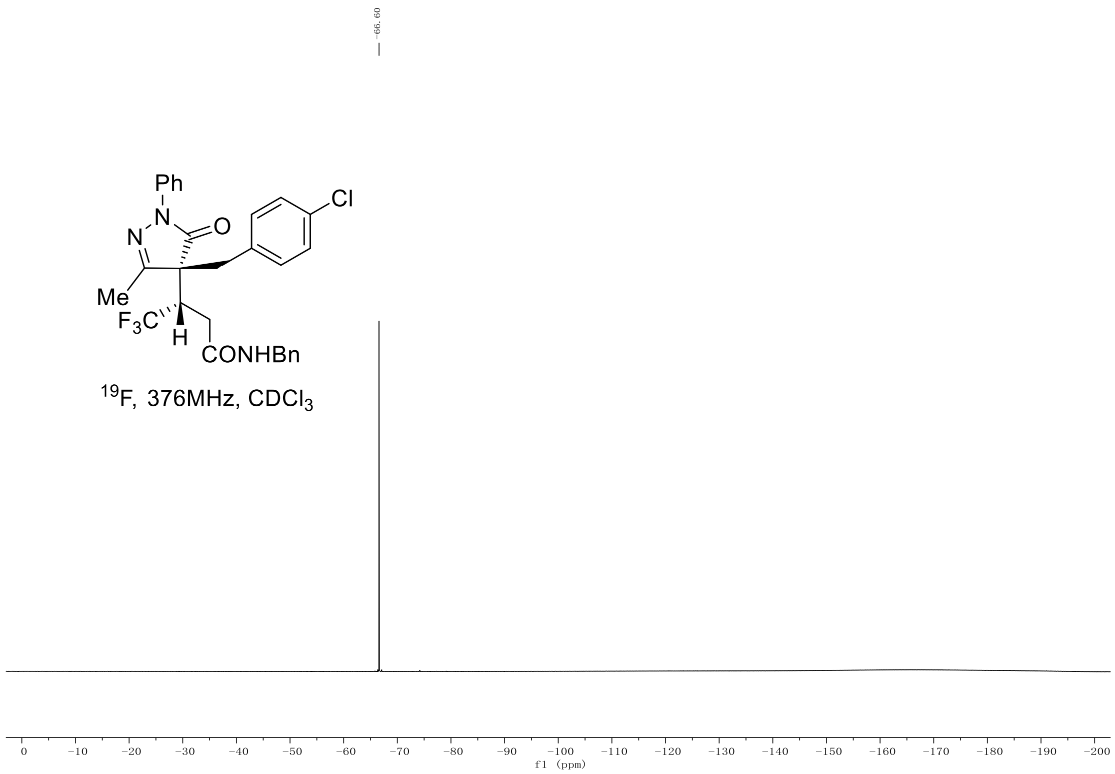
-66.60
Ph
N
N
O
Cl
Me
F3C
H
CONHBn
19F, 376MHz, CDCl3
f1 (ppm)

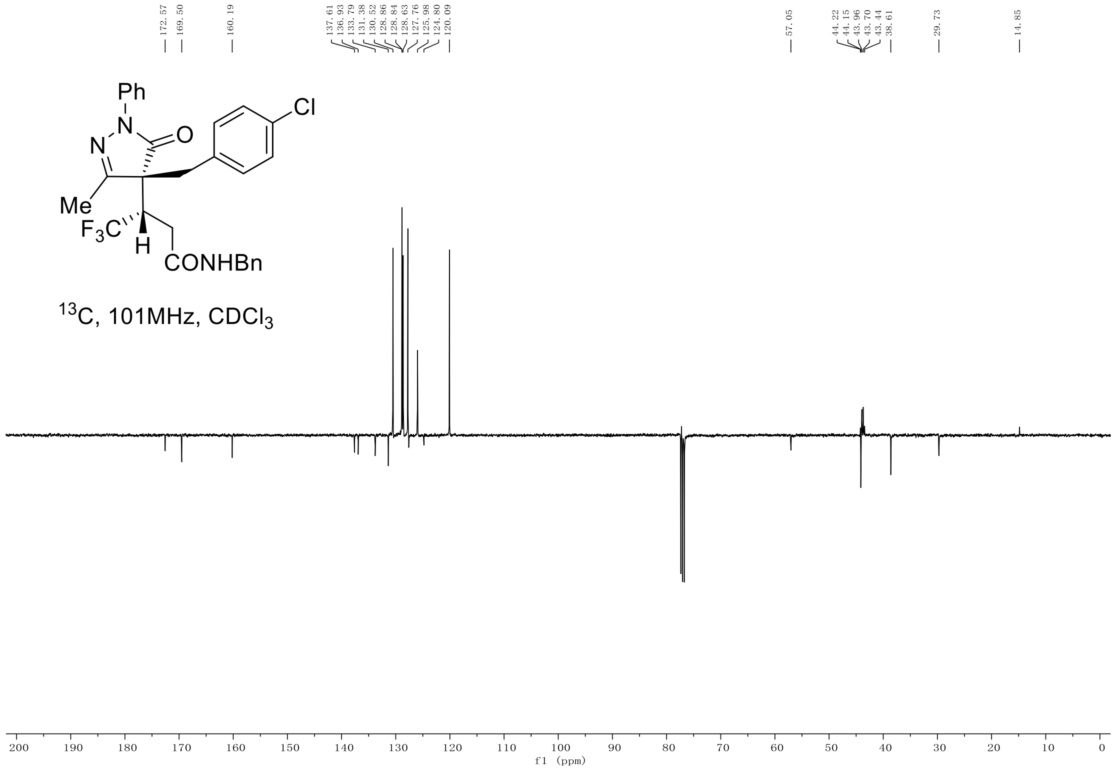
172.57
169.50
160.19
137.61
136.93
133.79
131.38
130.52
128.86
128.84
128.63
127.76
125.98
124.80
120.09
57.05
44.22
44.15
43.96
43.70
43.44
38.61
29.73
14.85
Ph
N
N
O
Cl
Me
F3C
H
CONHBn
13C, 101MHz, CDCl3
f1 (ppm)

(*S*)-*N*-benzyl-3-((*S*)-4-(4-cyanobenzyl)-3-methyl-5-oxo-1-phenyl-4,5-dihydro-1*H*-pyrazol-4-yl)-4,4,4-trifluorobutanamide (34_{major})

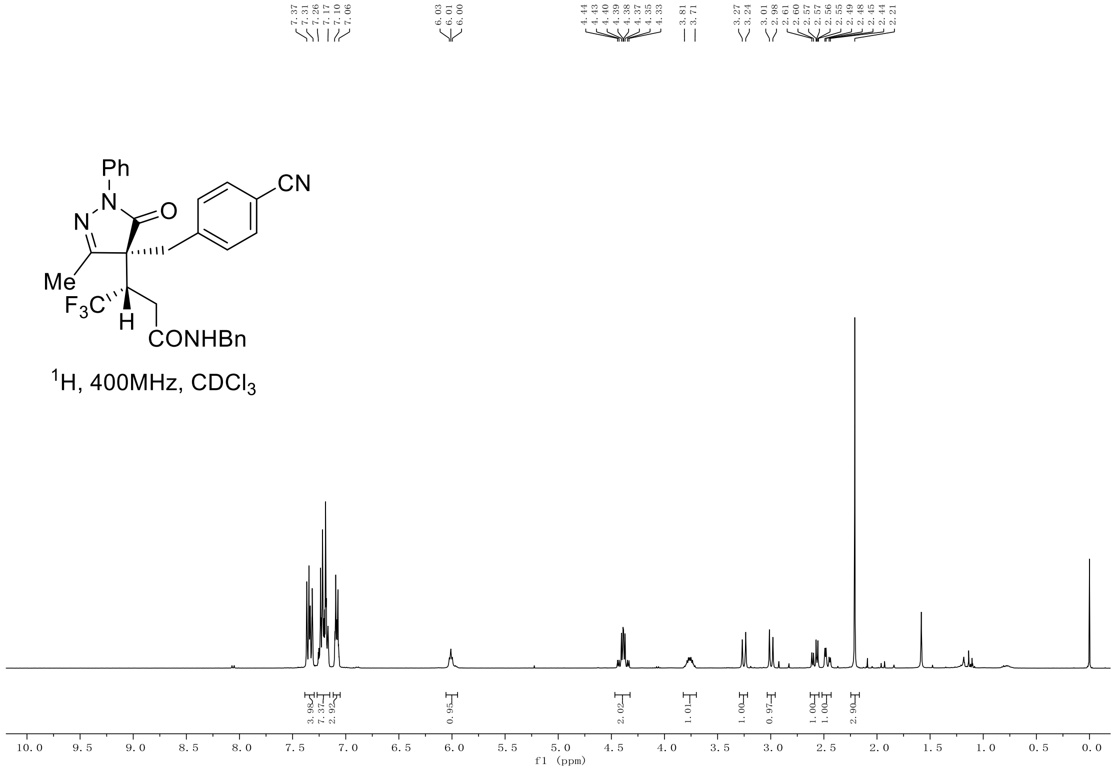

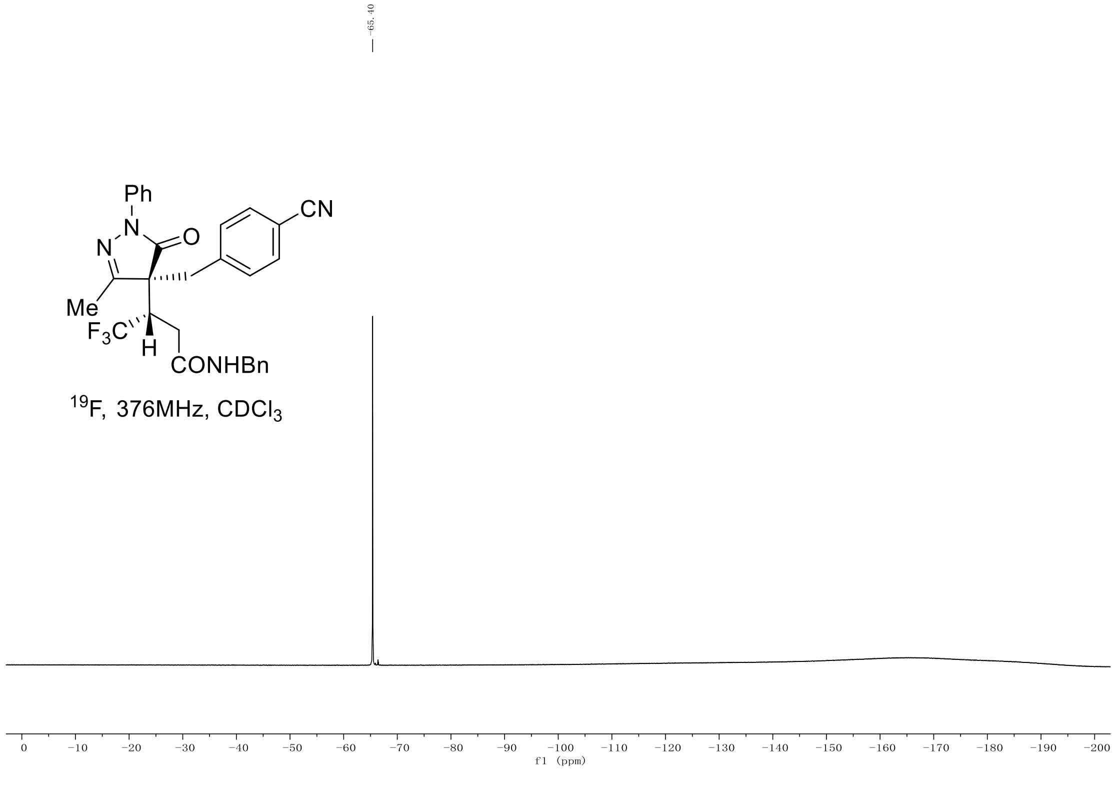

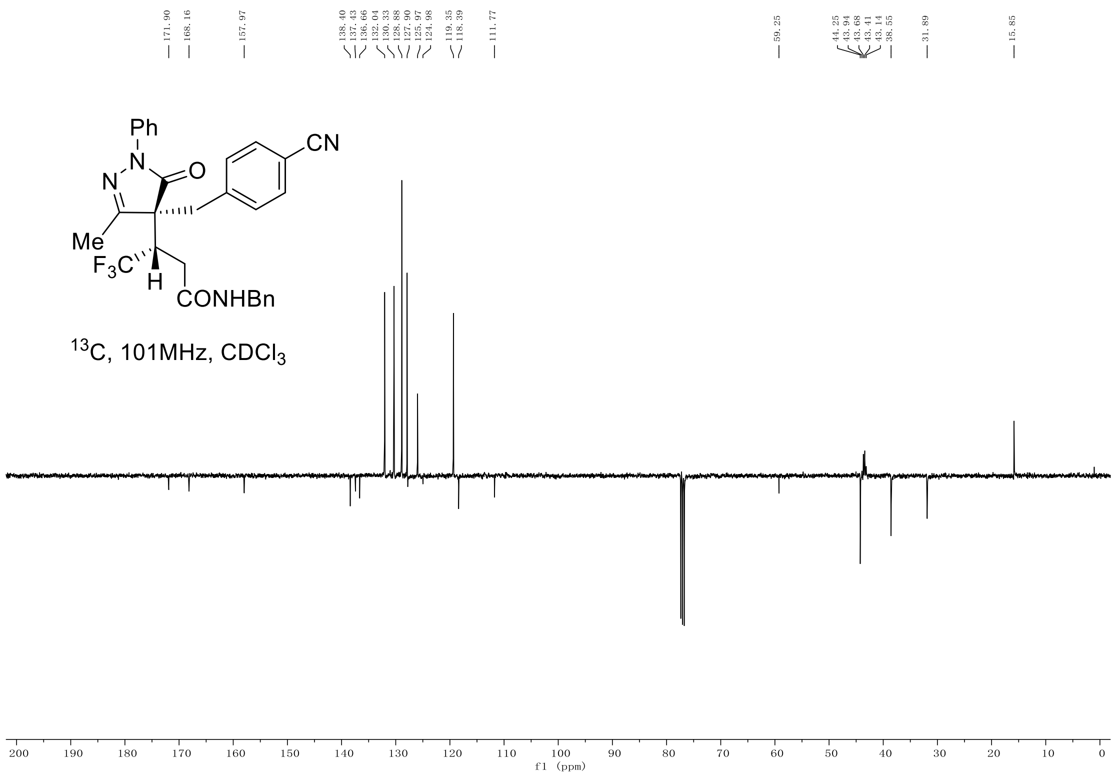

(*S*)-*N*-benzyl-3-((*R*)-4-(4-cyanobenzyl)-3-methyl-5-oxo-1-phenyl-4,5-dihydro-1*H*-pyrazol-4-yl)-4,4,4-trifluorobutanamide (34$_{\text{minor}}$)

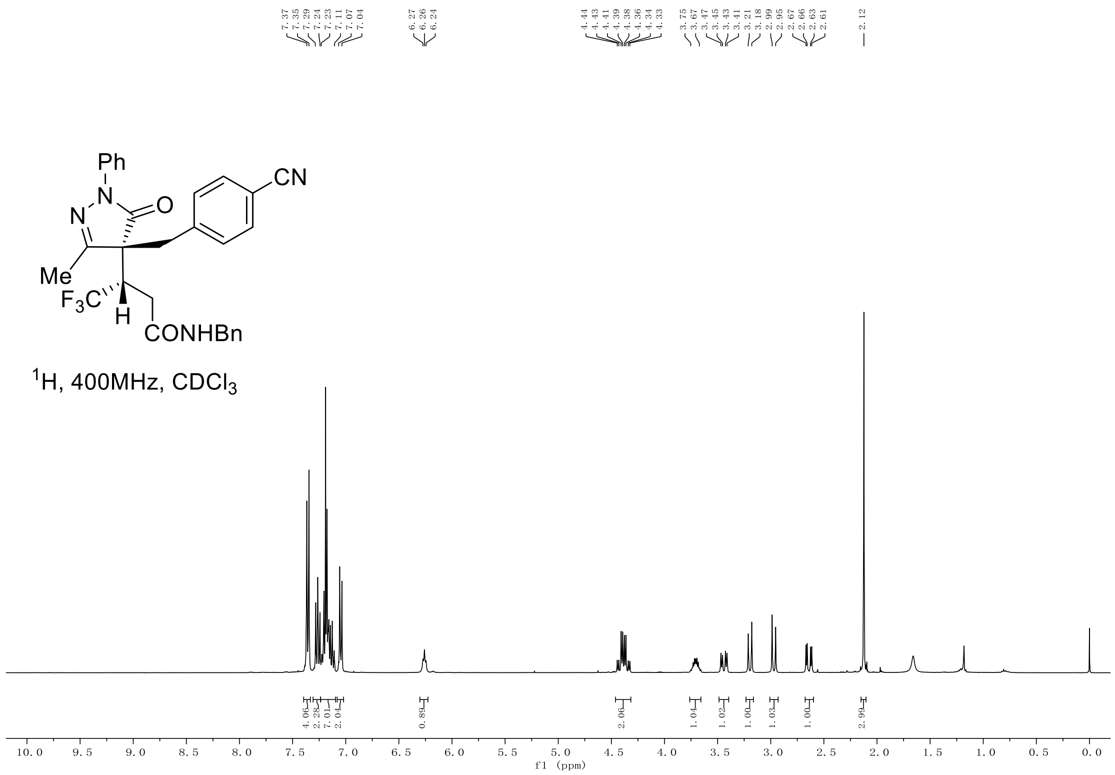

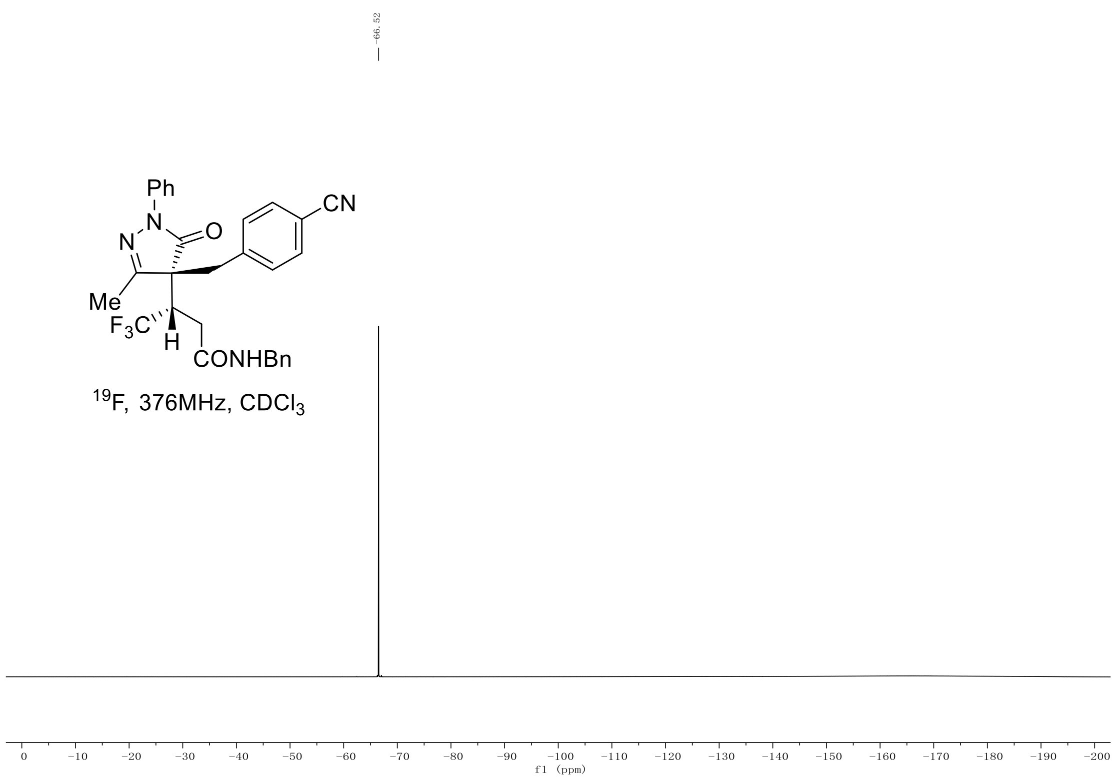

—66.52
Ph
N
N
O
CN
Me
F3C
H
CONHBn
19F, 376MHz, CDCl3
f1 (ppm)

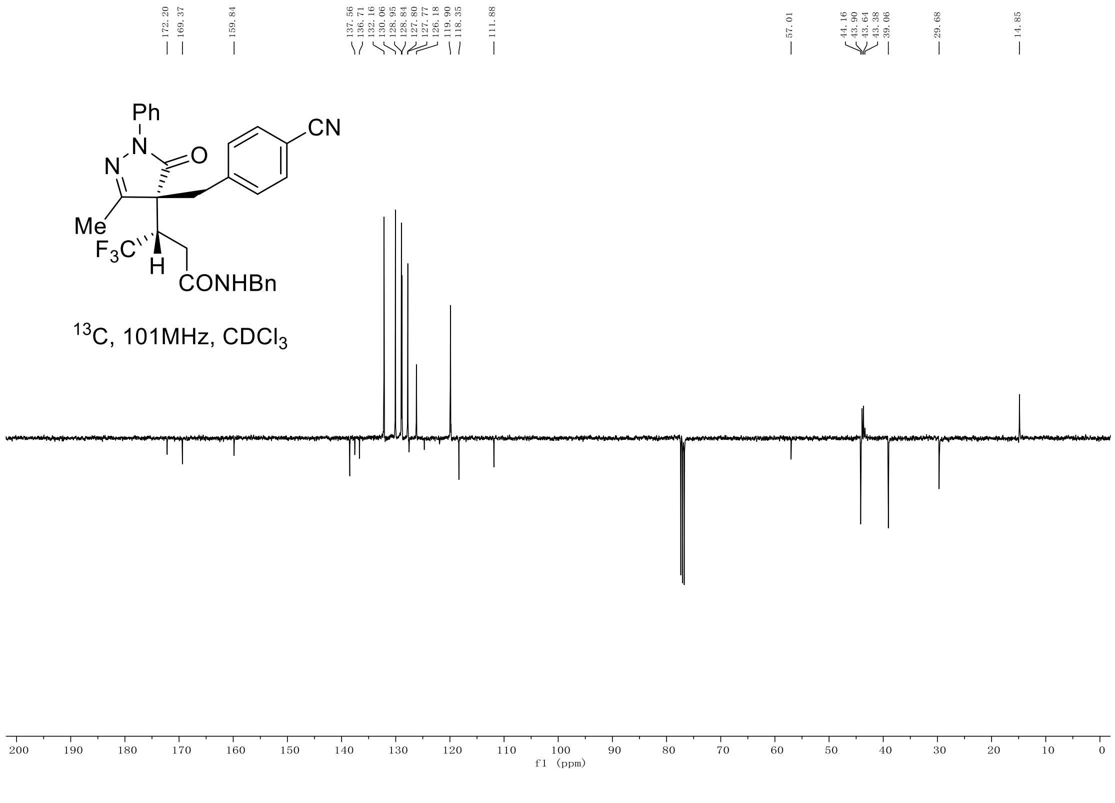

172.20
169.37
159.84
137.56
136.71
132.16
130.06
128.95
128.84
127.80
127.77
126.18
119.90
118.35
111.88
57.01
44.16
43.90
43.64
43.38
39.06
29.68
14.85
Ph
N
N
O
CN
Me
F3C
H
CONHBn
13C, 101MHz, CDCl3
f1 (ppm)

(*S*)-*N*-benzyl-4,4,4-trifluoro-3-((*R*)-3-methyl-5-oxo-1,4-diphenyl-4,5-dihydro-1*H*-pyrazol-4-yl)butanamide (35$_{major}$)

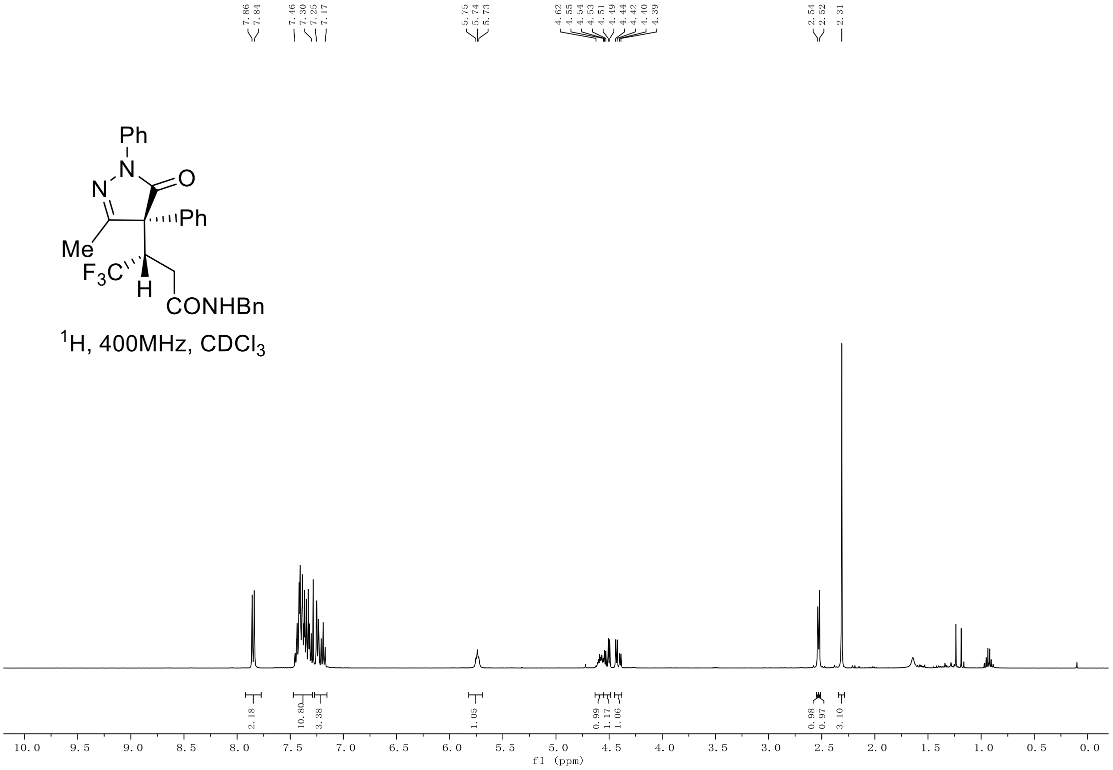

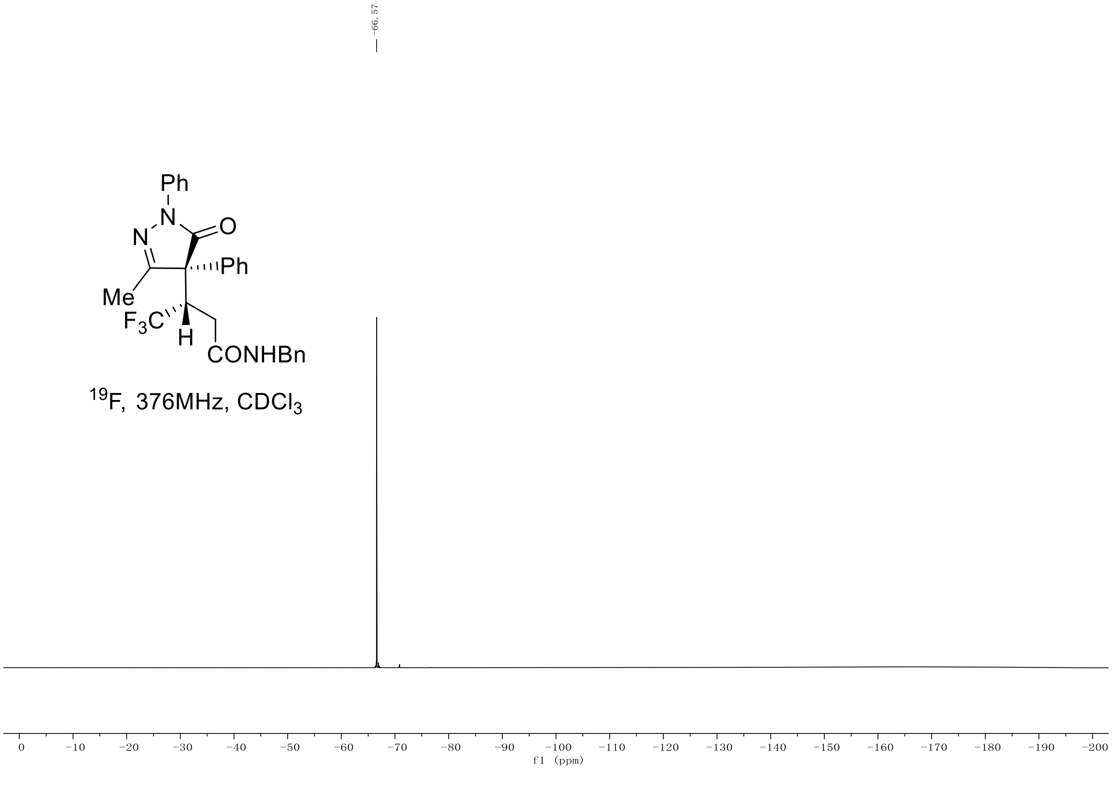

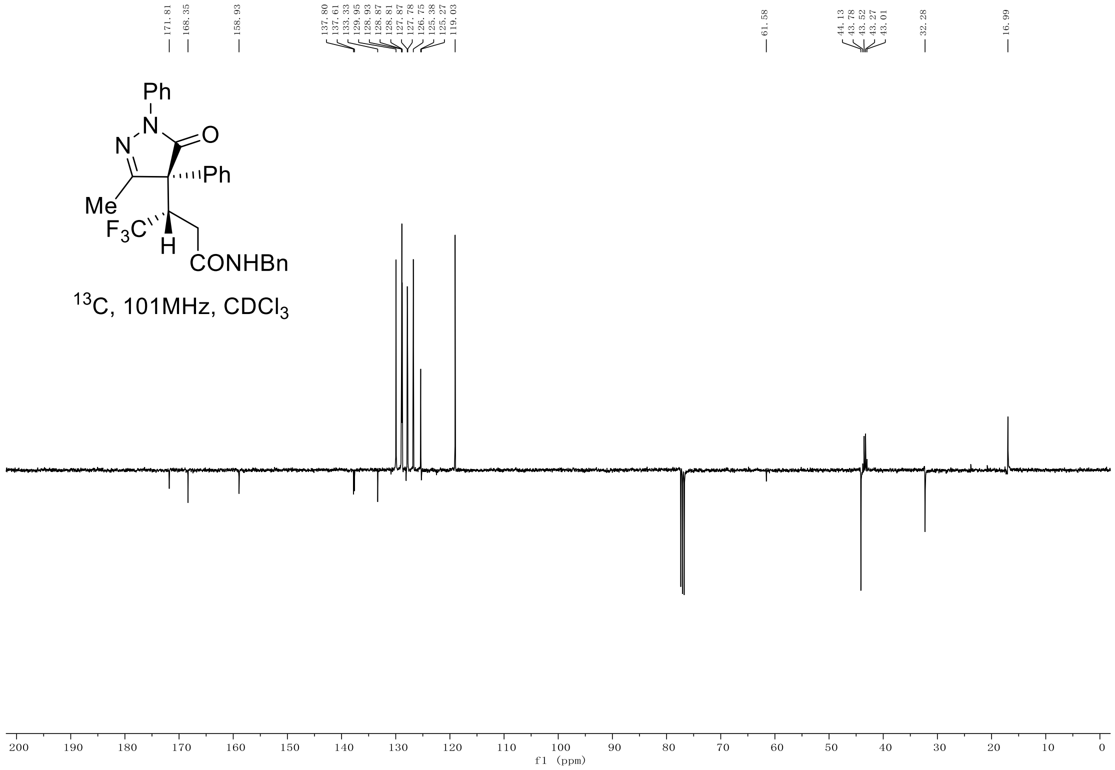

(*S*)-*N*-benzyl-4,4,4-trifluoro-3-((*S*)-3-methyl-5-oxo-1,4-diphenyl-4,5-dihydro-1*H*-pyrazol-4-yl)butanamide (35$_{minor}$)

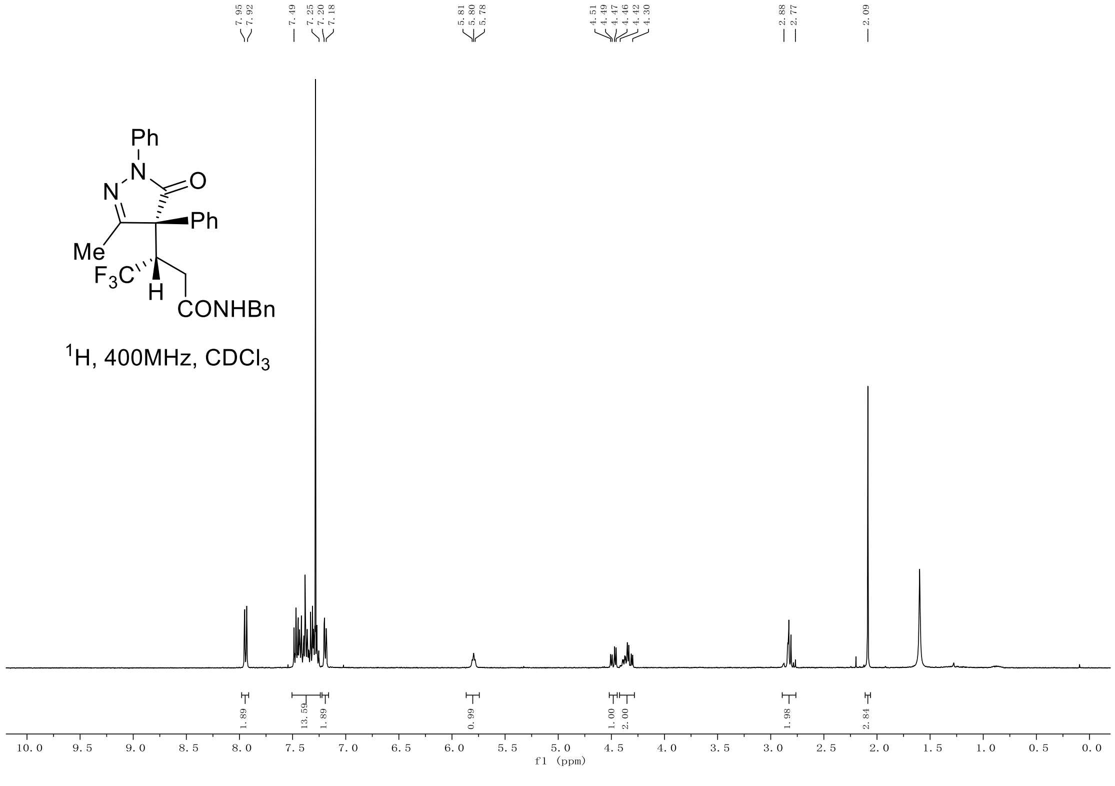

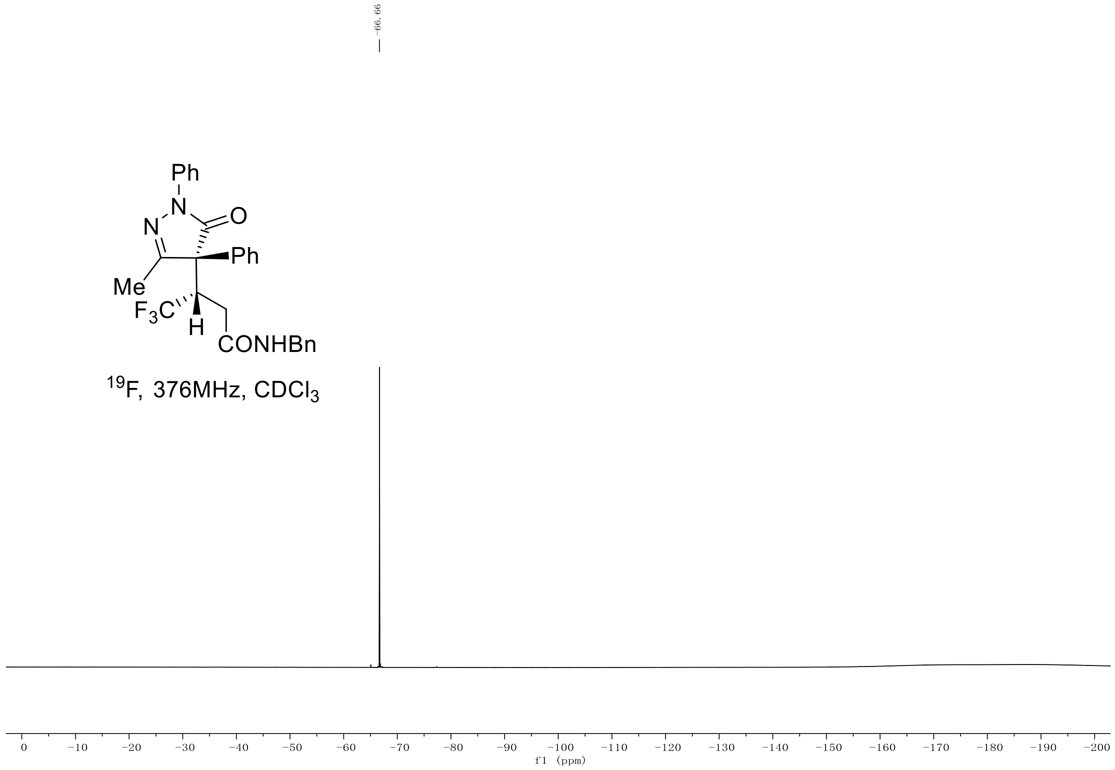

-66.66
Ph
N
N
O
Ph
Me
F3C
H
CONHBn
19F, 376MHz, CDCl3
f1 (ppm)

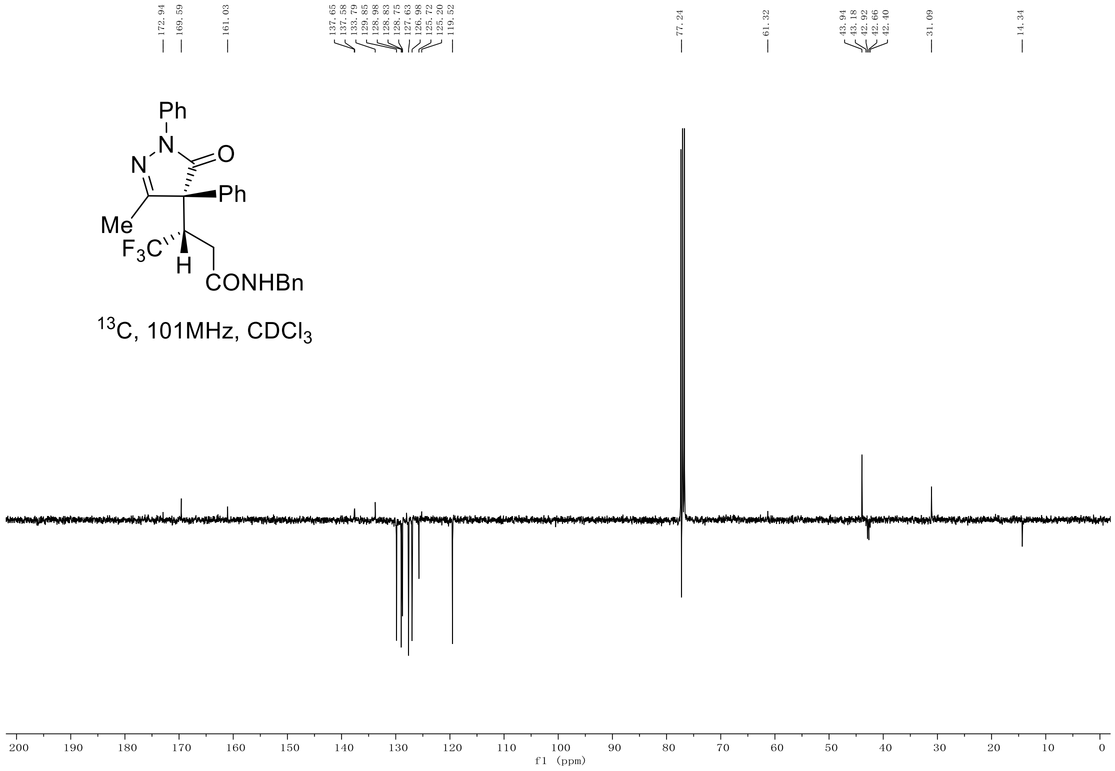

172.94
169.59
161.03
137.65
137.58
133.79
129.85
128.98
128.83
128.75
127.63
126.98
125.72
125.20
119.52
77.24
61.32
43.94
43.18
42.92
42.66
42.40
31.09
14.34
Ph
N
N
O
Ph
Me
F3C
H
CONHBn
13C, 101MHz, CDCl3
f1 (ppm)

***tert*-Butyl (*R*)-2-oxo-3-phenyl-3-((*S*)-1,1,1-trifluoro-4-(4-nitrophenoxy)-4-oxobutan-2-yl)indoline-1-carboxylate (13)**

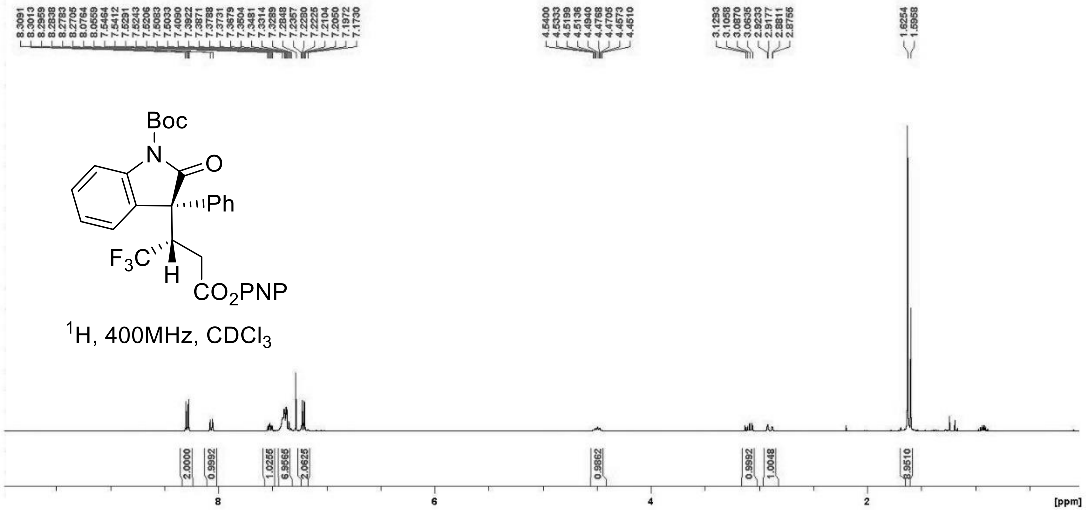

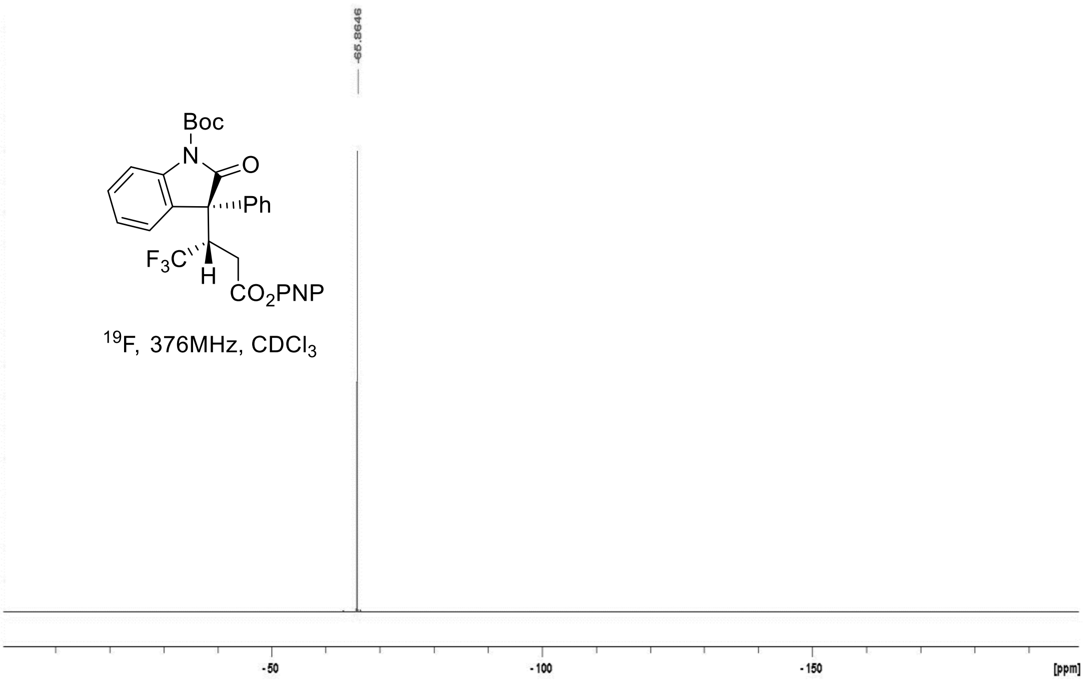

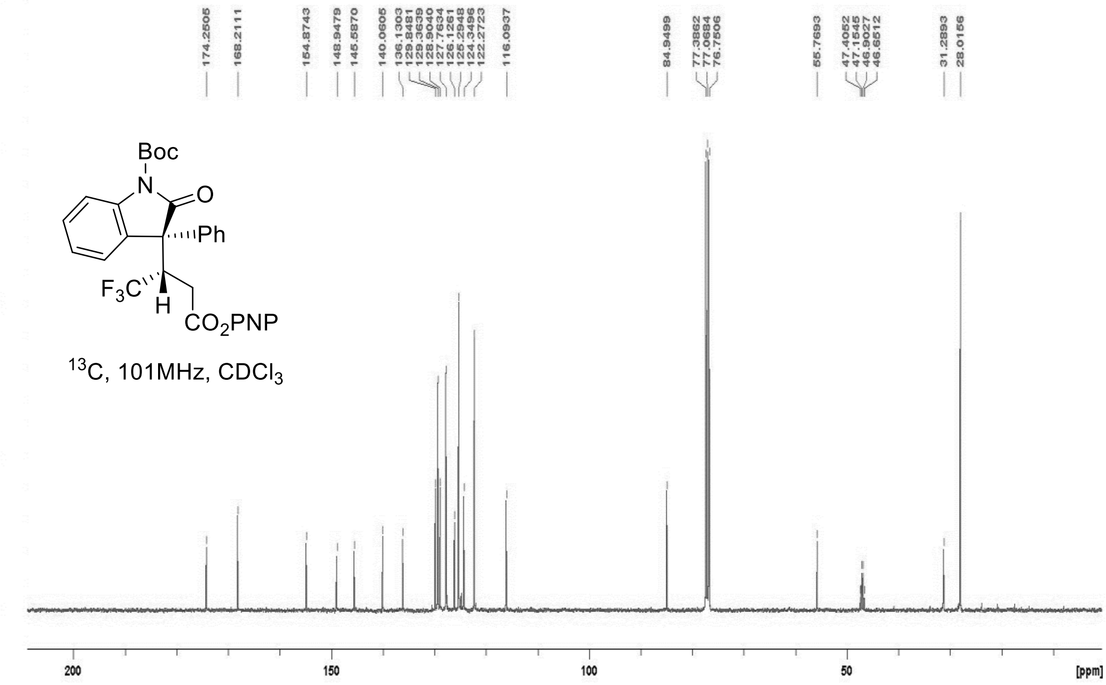

(*S*)-N-benzyl-4,4,4-trifluoro-3-((*R*)-1-methyl-2-oxo-3-phenylindolin-3-yl) butanamide (53)

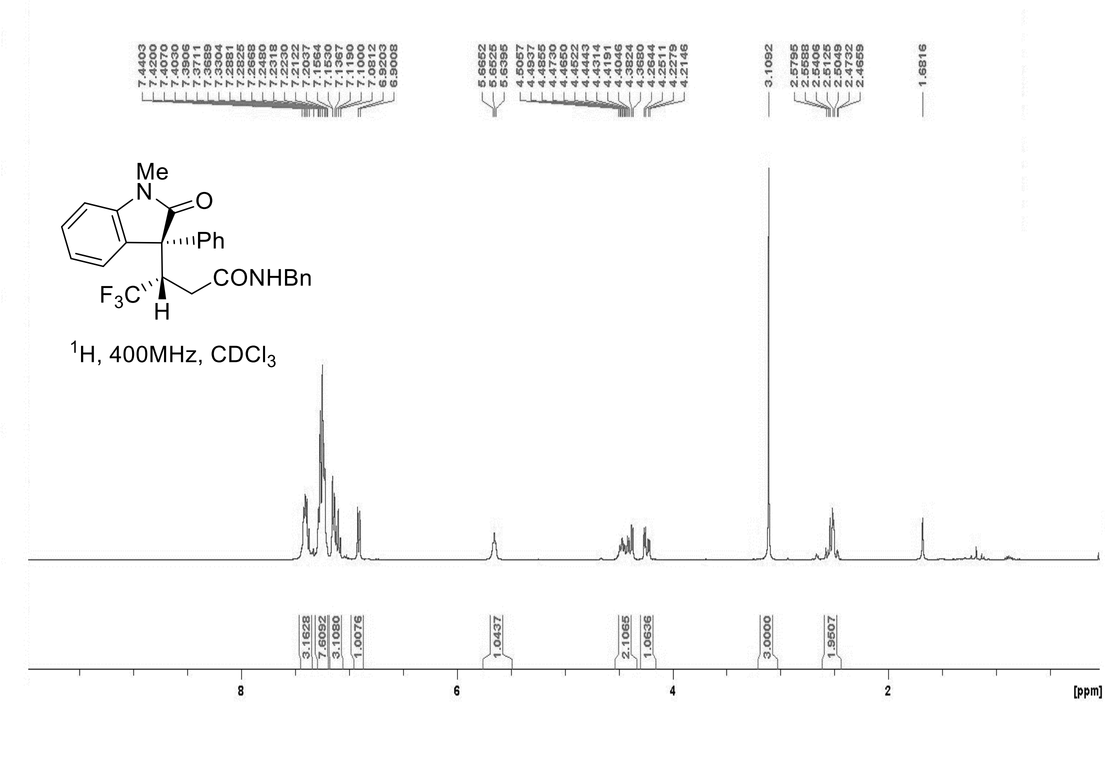

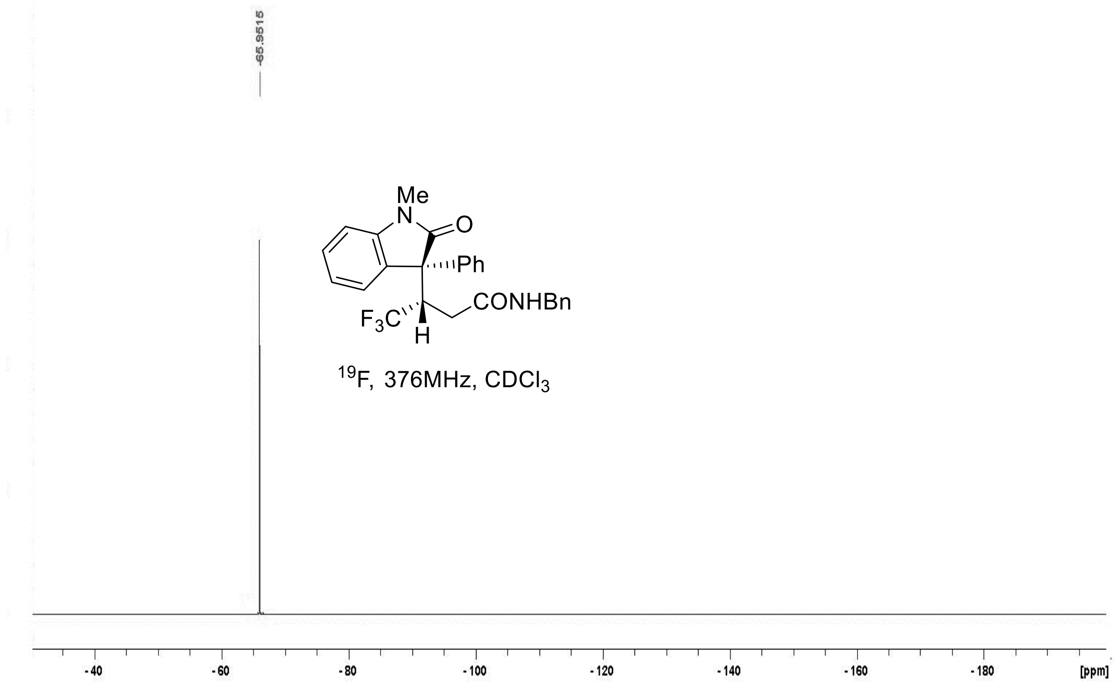

-65.9515
Me
N
O
Ph
CONHBn
F3C
H
19F, 376MHz, CDCl3
-40
-60
-80
-100
-120
-140
-160
-180
[ppm]

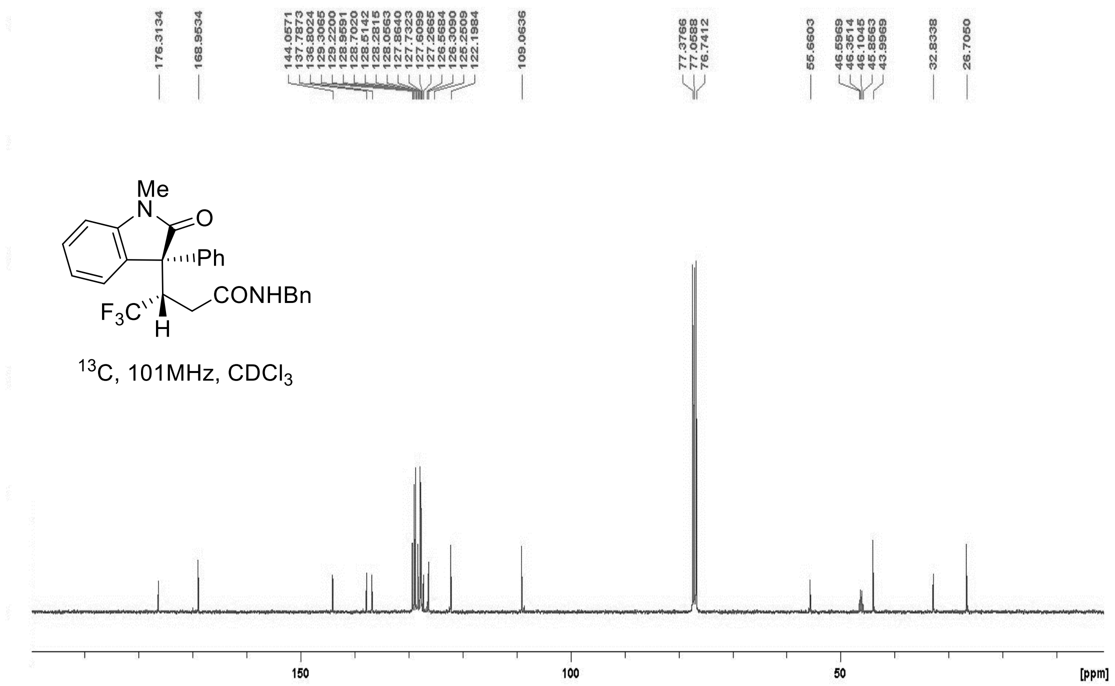

176.3134
168.9534
144.0571
137.7873
136.8024
129.3065
129.2200
128.9591
128.7020
128.5142
128.2815
128.0563
127.8640
127.7323
127.6099
127.2665
126.5684
126.3090
125.2859
122.1984
109.0636
77.3766
77.0588
76.7412
55.6603
46.5969
46.3514
46.1045
45.8563
43.9969
32.8338
26.7050
Me
N
O
Ph
CONHBn
F3C
H
13C, 101MHz, CDCl3
150
100
50
[ppm]

4-nitrophenyl (*S*)-3-((*R*)-1-benzyl-2-oxo-3-phenylindolin-3-yl)-4,4,4-trifluoro-Butanoate (54)

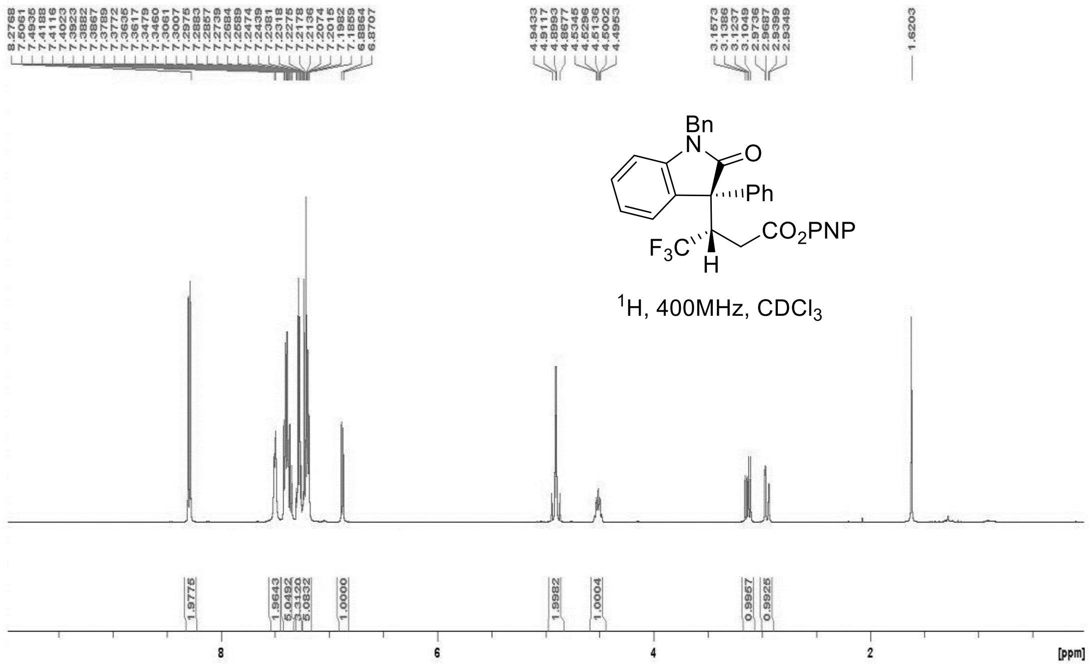

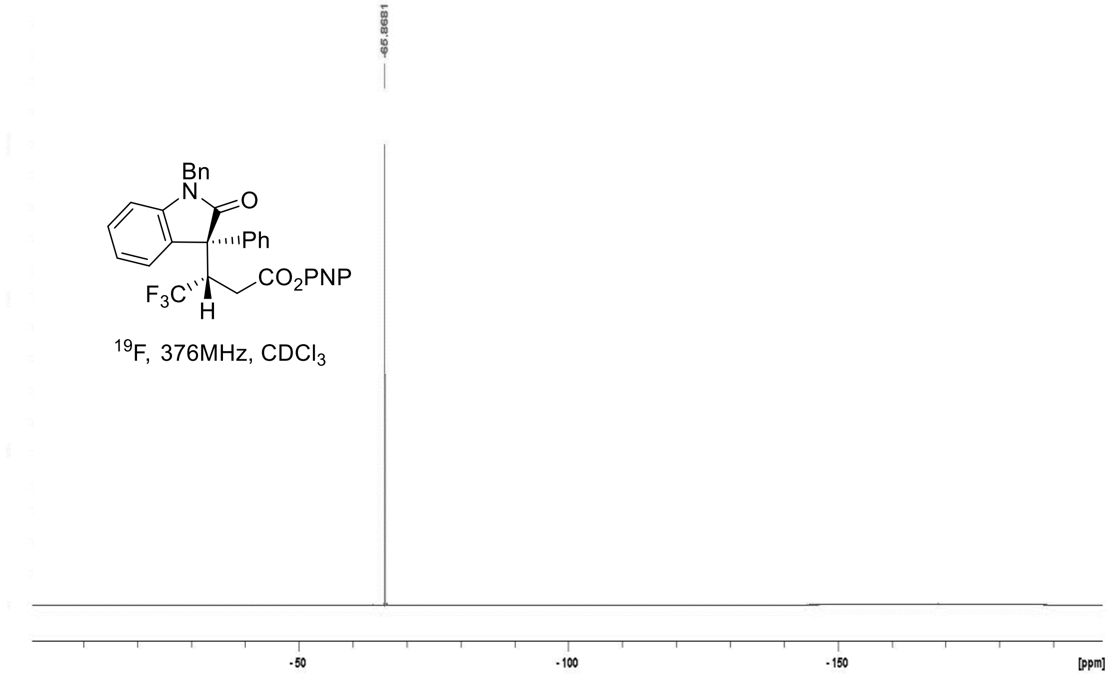

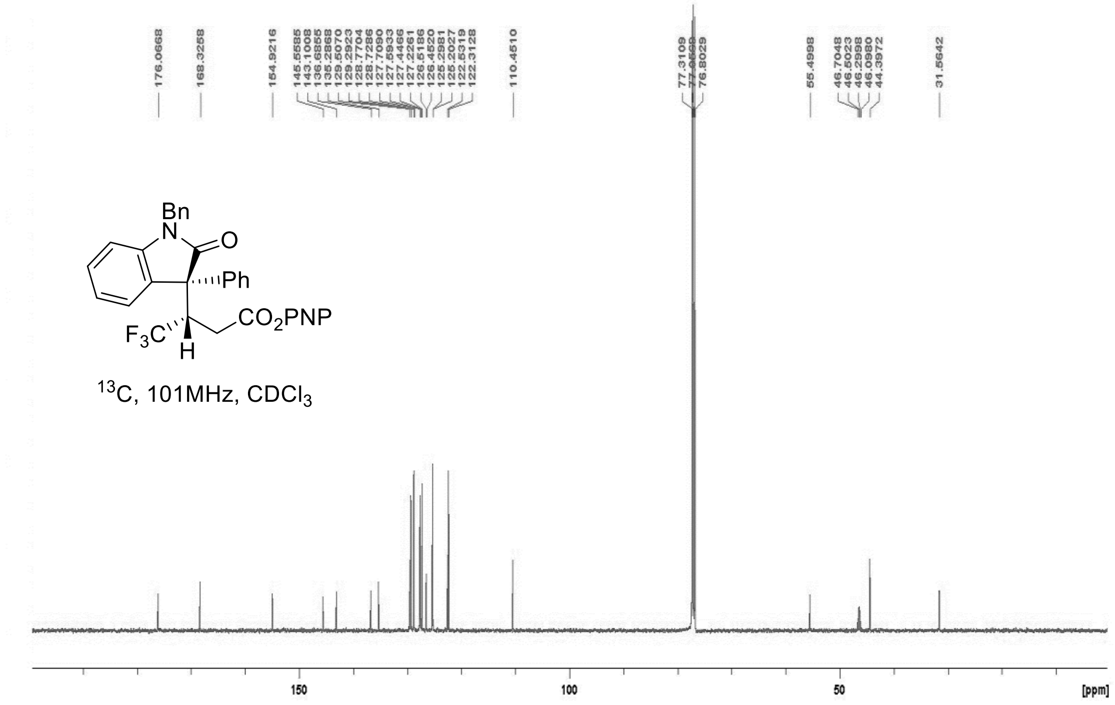

(*S*)-N-benzyl-3-((*R*)-1-benzyl-2-oxo-3-phenylindolin-3-yl)-4,4,4-trifluoro butanamide (55)

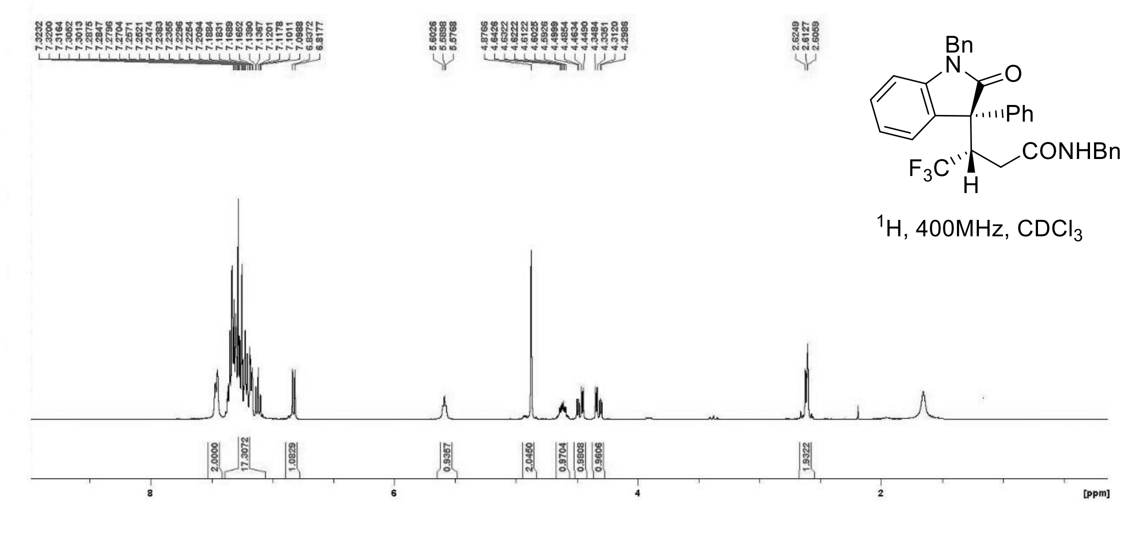

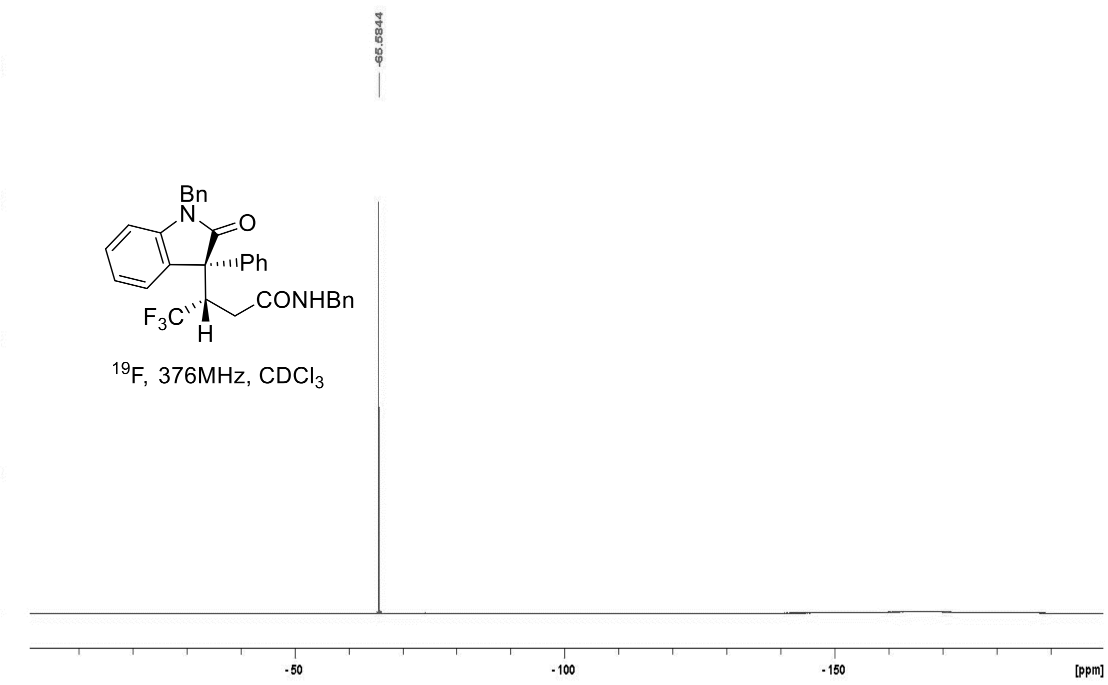
-65.5844
Bn
N
O
Ph
CONHBn
F3C
H
19F, 376MHz, CDCl3
-50
-100
-150
[ppm]

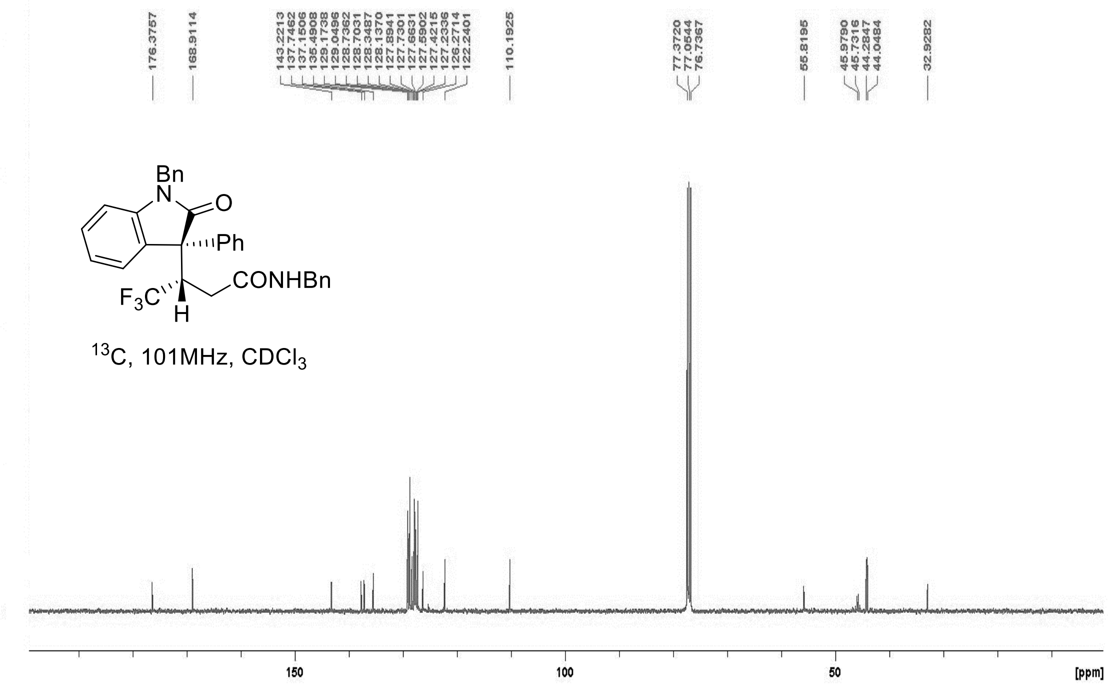
176.3757
168.9114
143.2213
137.7462
137.1506
135.4908
129.1738
129.0436
128.7362
128.7031
128.3487
128.1370
127.8941
127.7301
127.6631
127.5902
127.4215
127.2335
126.2714
122.2401
110.1925
77.3720
77.0544
76.7367
55.8195
45.9790
45.7316
44.2847
44.0484
32.9282
Bn
N
O
Ph
CONHBn
F3C
H
13C, 101MHz, CDCl3
150
100
50
[ppm]

(*R*)-1-benzyl-3-phenyl-3-((*S*)-1,1,1-trifluoro-4-oxo-4-(pyrrolidin-1-yl)butan-2-yl)indolin-2-one (56)

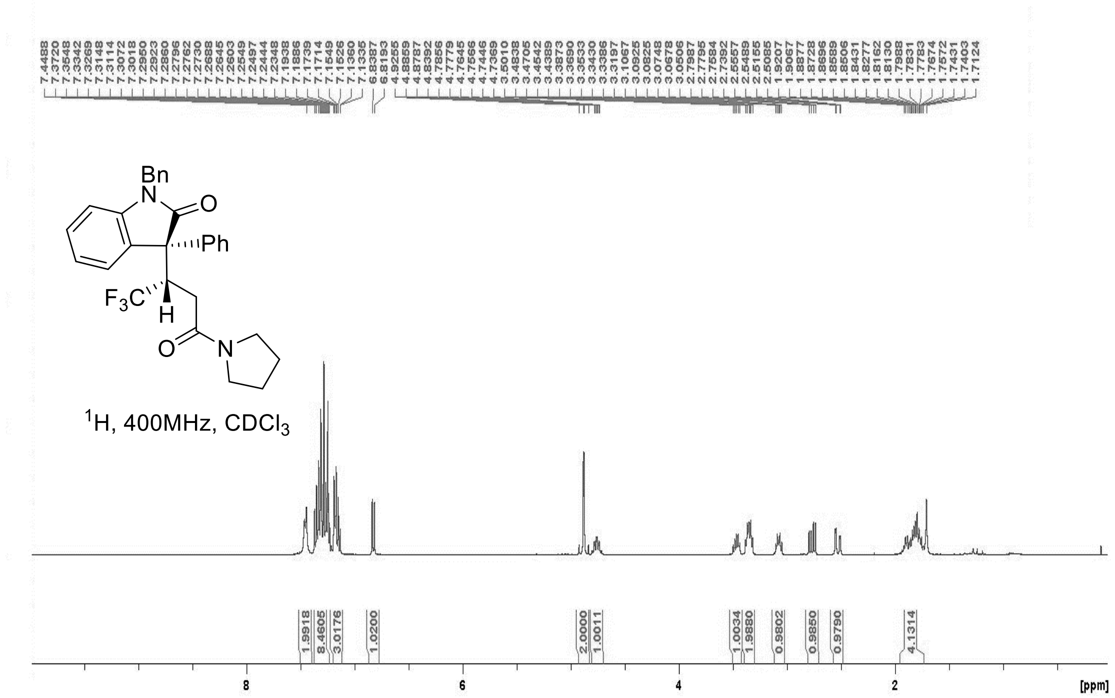

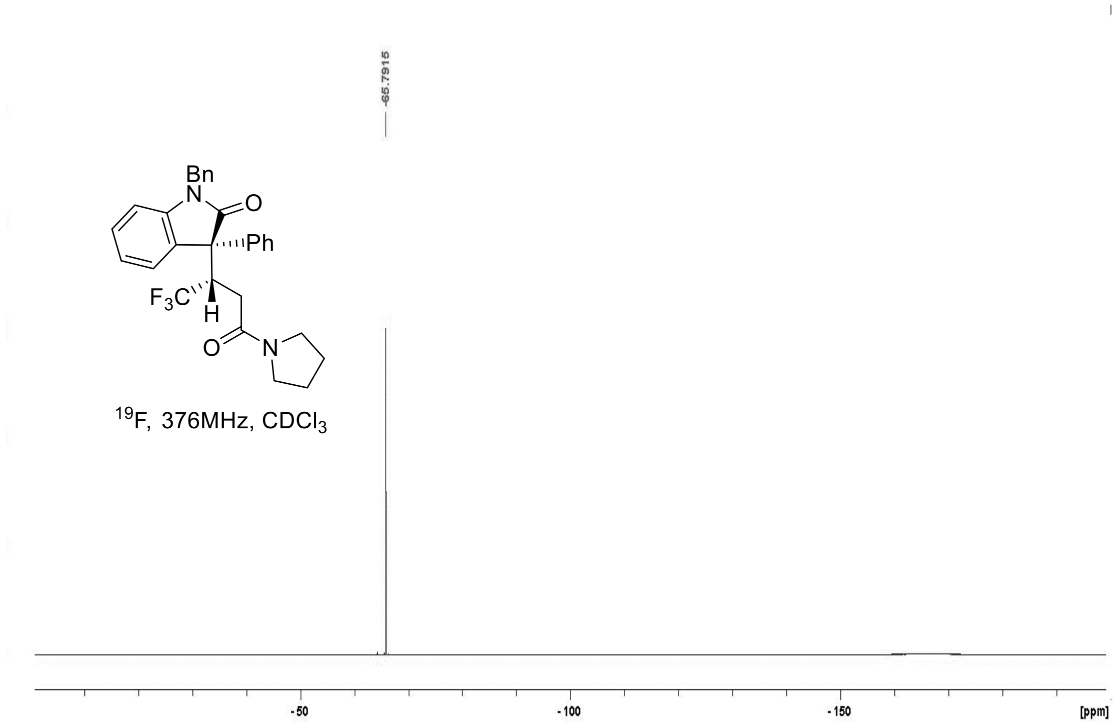

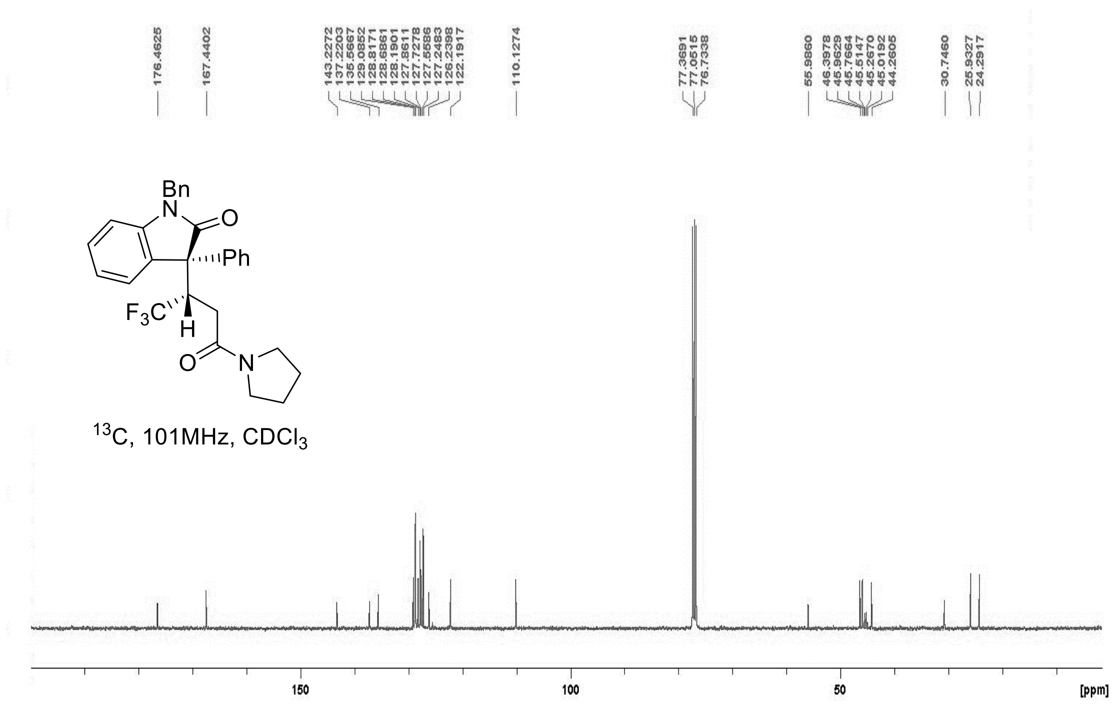

(*R*)-1-benzyl-3-phenyl-3-((*S*)-1,1,1-trifluoro-4-morpholino-4-oxobutan-2-yl) indolin-2-one (57)

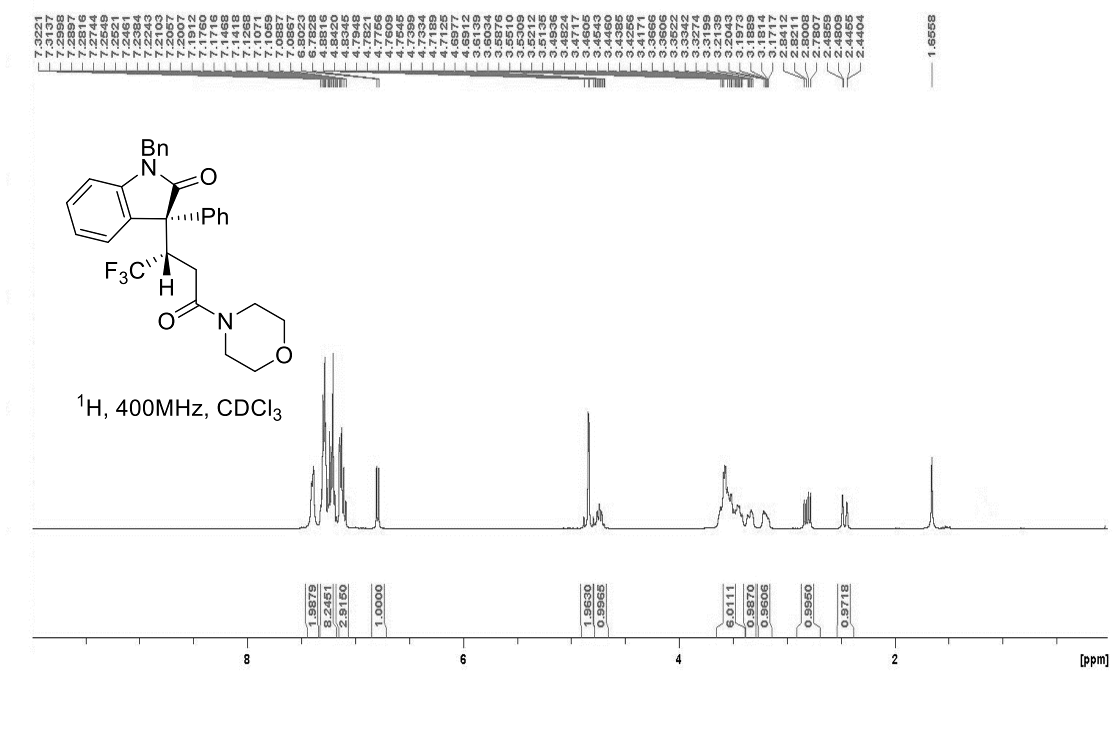

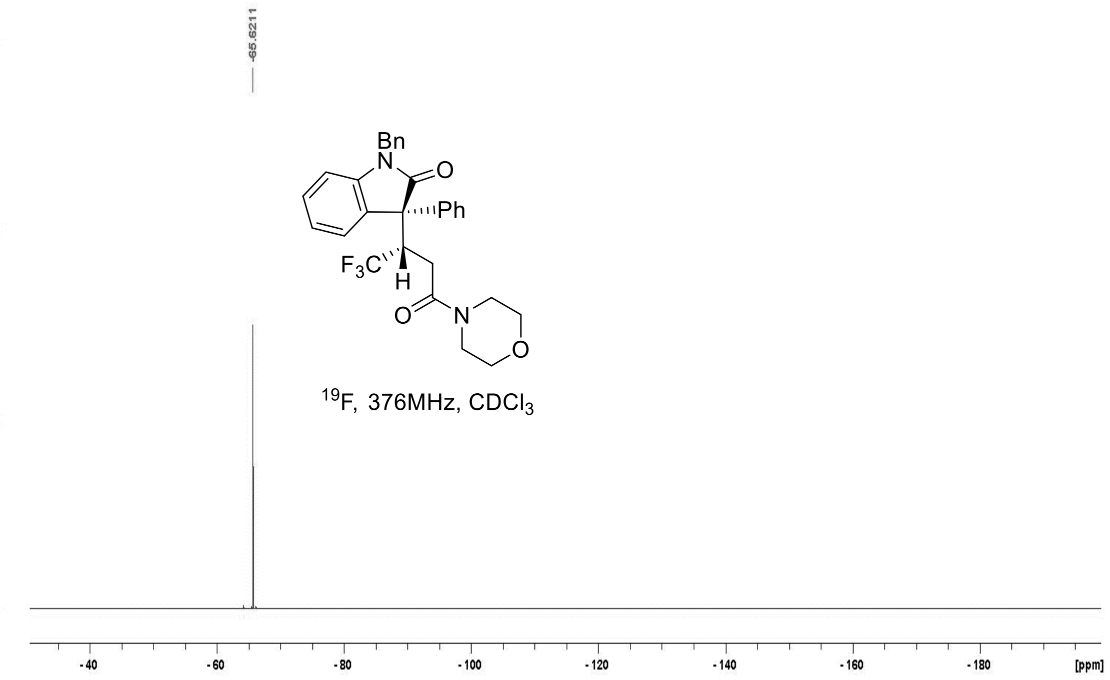

-65.6211
Bn
N
O
Ph
F3C
H
O
N
O
19F, 376MHz, CDCl3
-40
-60
-80
-100
-120
-140
-160
-180
[ppm]

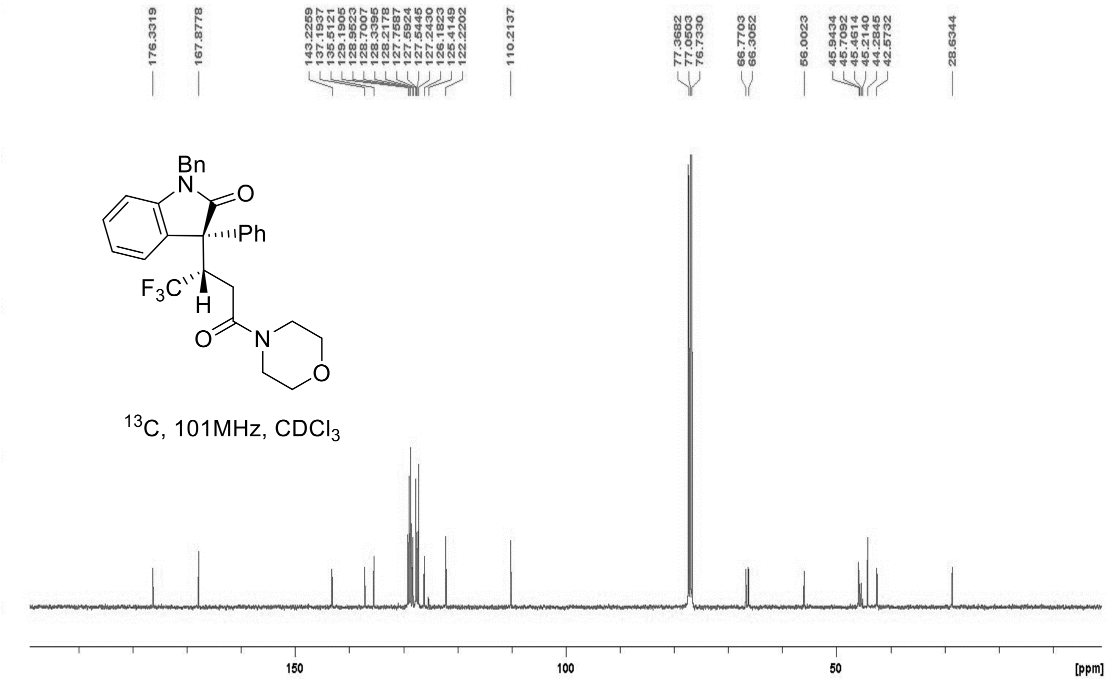

176.3319
167.8778
143.2259
137.1937
135.5121
129.1905
128.9523
128.7007
128.3395
128.2178
127.7587
127.5924
127.5445
127.2430
126.1823
125.4149
122.2202
110.2137
77.3682
77.0503
76.7330
66.7703
66.3052
56.0023
45.9434
45.7092
45.4614
45.2140
44.2845
42.5732
28.6344
Bn
N
O
Ph
F3C
H
O
N
O
13C, 101MHz, CDCl3
150
100
50
[ppm]

(*S*)-N-benzyl-3-((*R*)-1-benzyl-5-methoxy-2-oxo-3-phenylindolin-3-yl)-4,4,4-trifluorobutanamide (58)

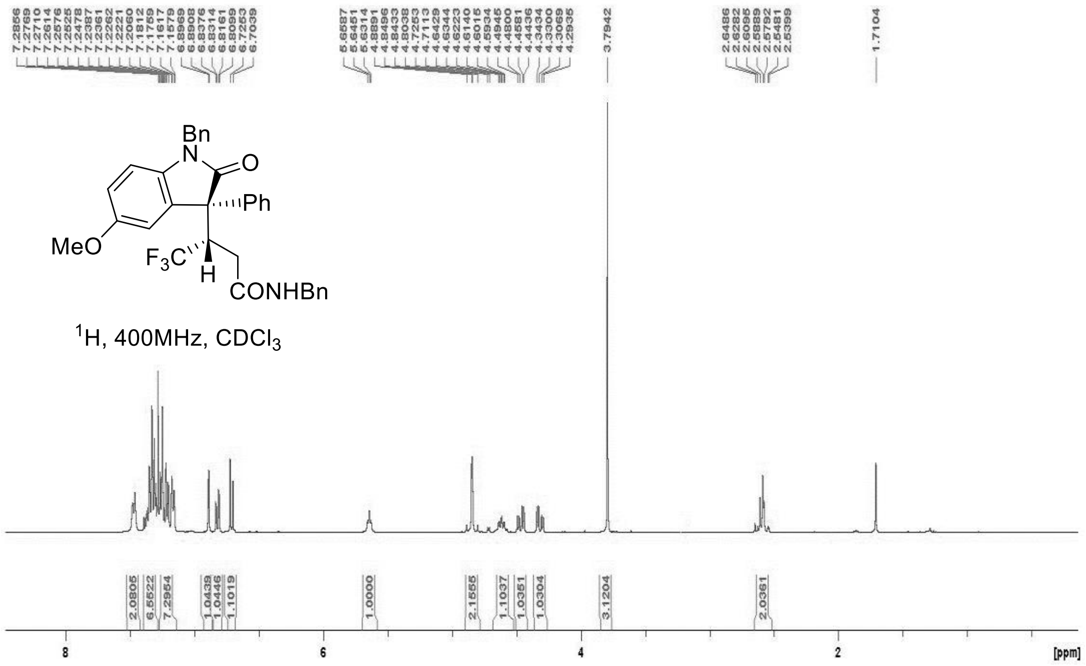

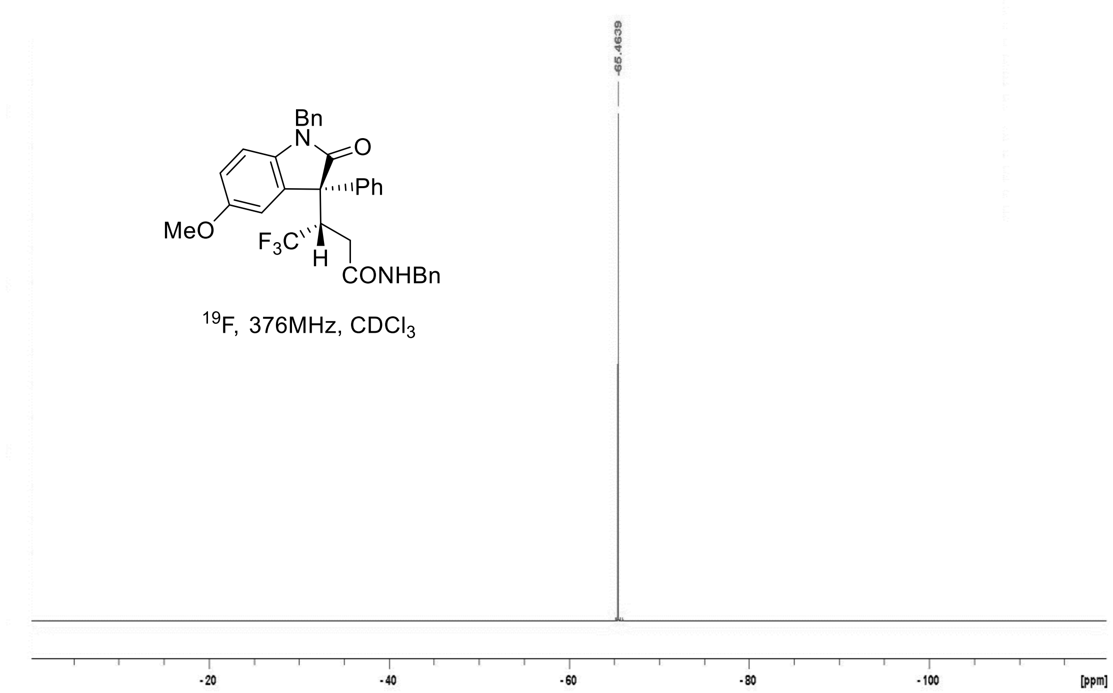

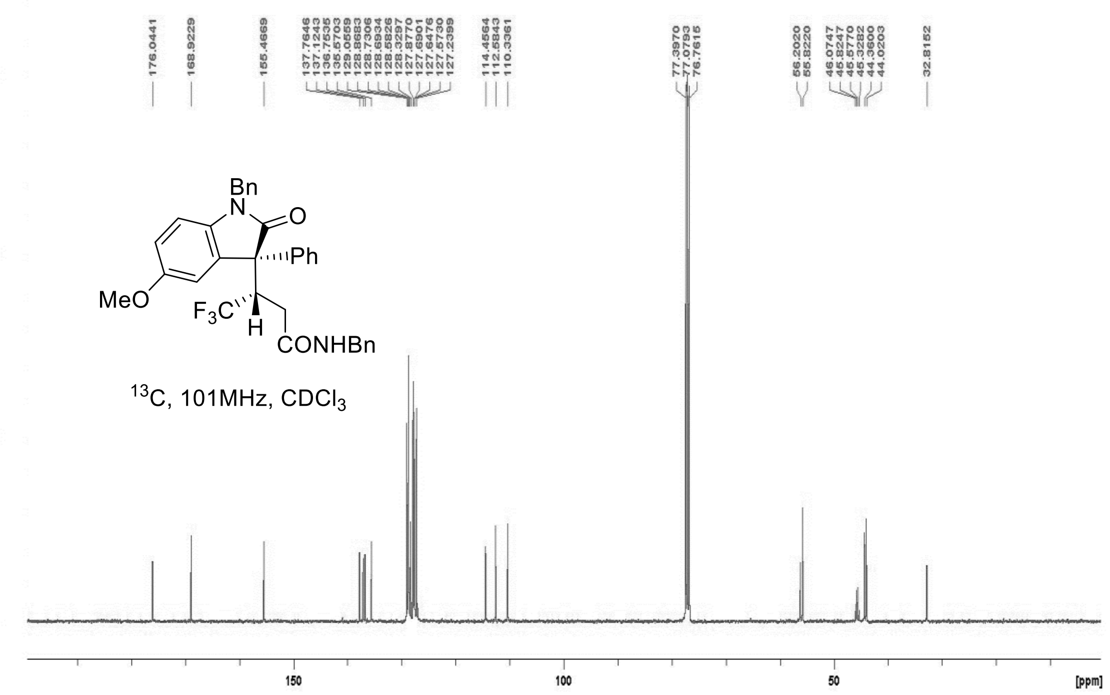

(*S*)-N-benzyl-3-((*R*)-1-benzyl-5-chloro-2-oxo-3-phenylindolin-3-yl)-4,4,4-trifluorobutanamide (59)

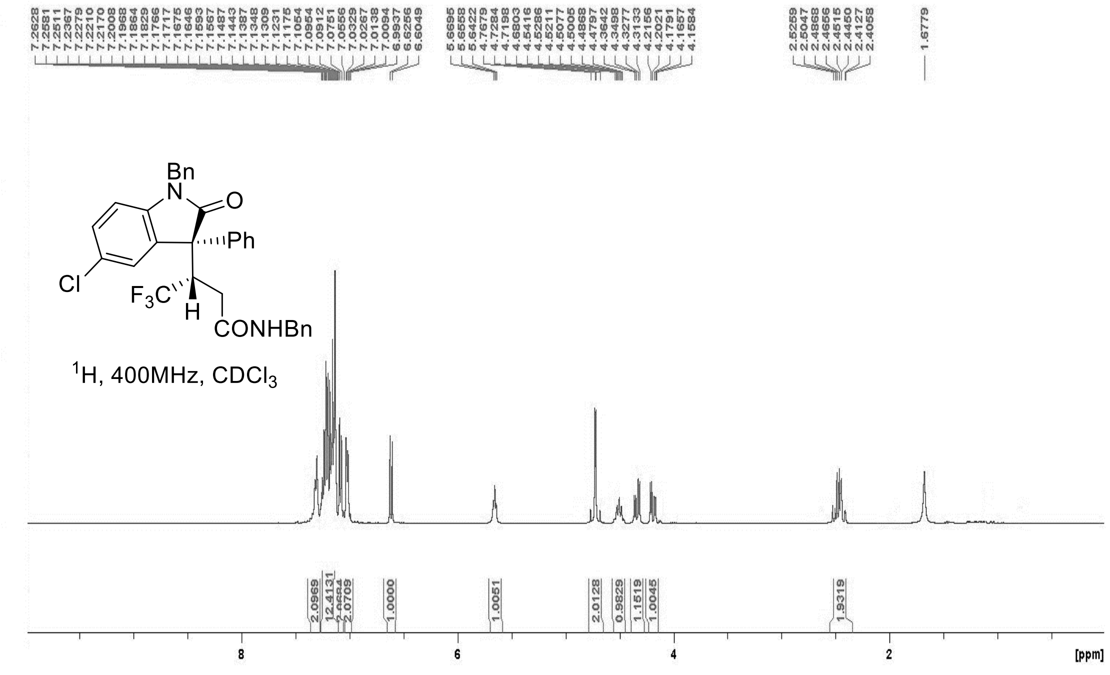

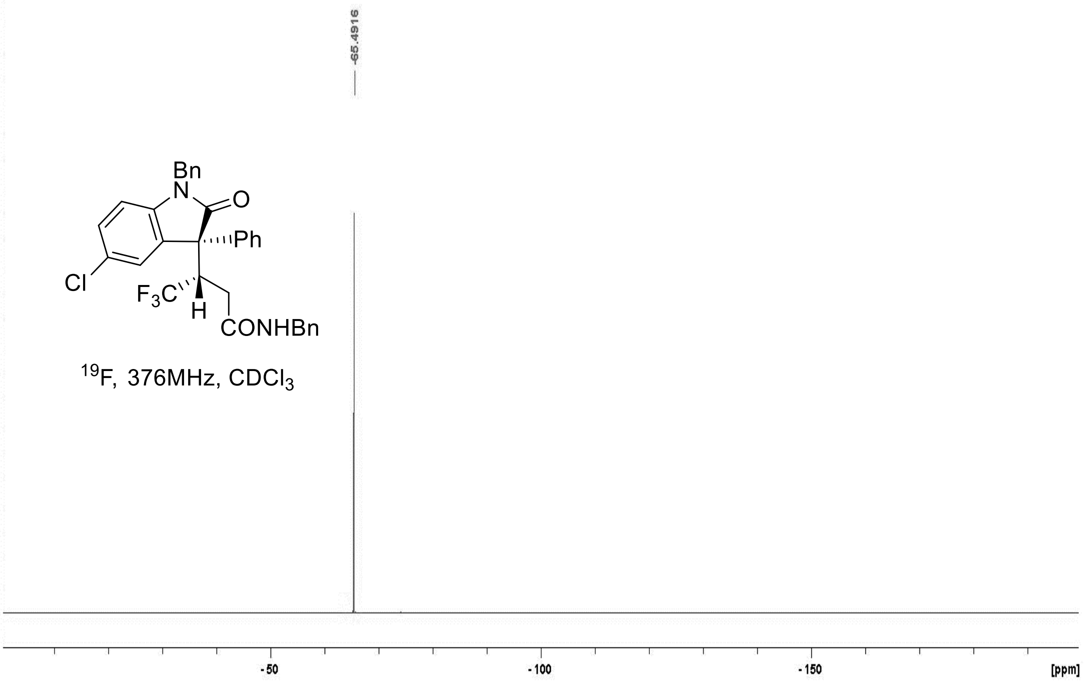

-65.4916
Bn
N
O
Ph
Cl
F3C
H
CONHBn
19F, 376MHz, CDCl3
-50
-100
-150
[ppm]

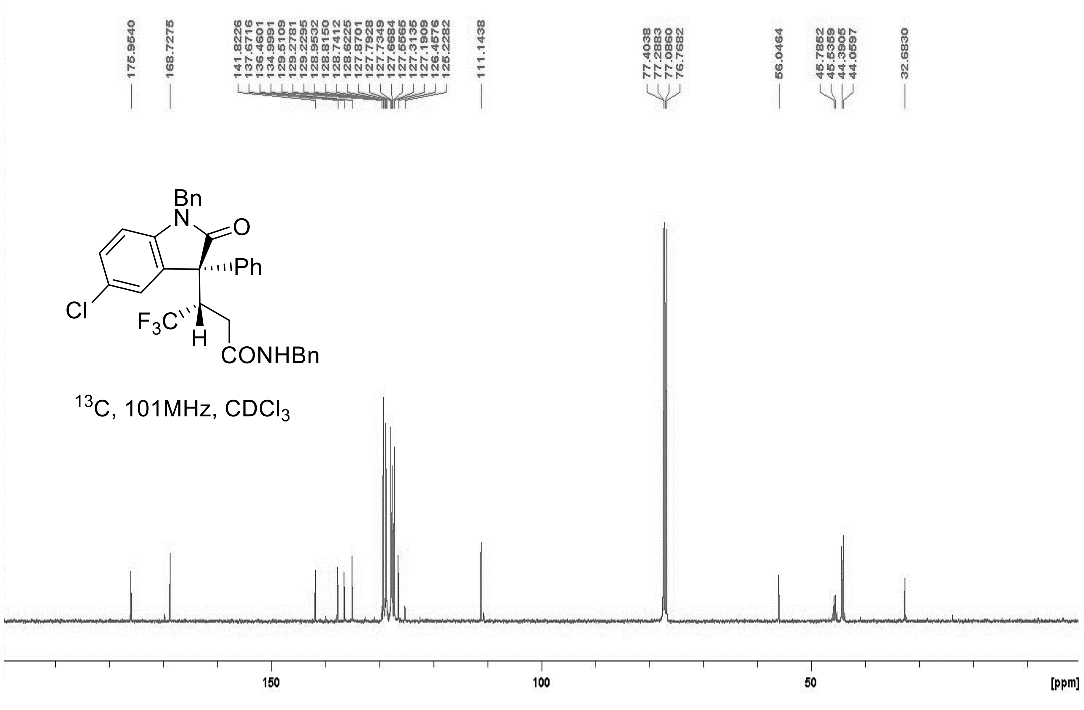

175.9540
168.7275
141.8226
137.6716
136.4601
134.9991
129.5109
129.2781
129.2295
128.9532
128.8150
128.7412
128.6225
127.8701
127.7928
127.7349
127.6684
127.5565
127.3135
127.1909
126.4576
125.2282
111.1438
77.4038
77.2883
77.0860
76.7682
56.0464
45.7852
45.5359
44.3905
44.0597
32.6830
Bn
N
O
Ph
Cl
F3C
H
CONHBn
13C, 101MHz, CDCl3
150
100
50
[ppm]

(*S*)-N-benzyl-3-((*R*)-1-benzyl-6-chloro-2-oxo-3-phenylindolin-3-yl)-4,4,4-trifluorobutanamide (60)

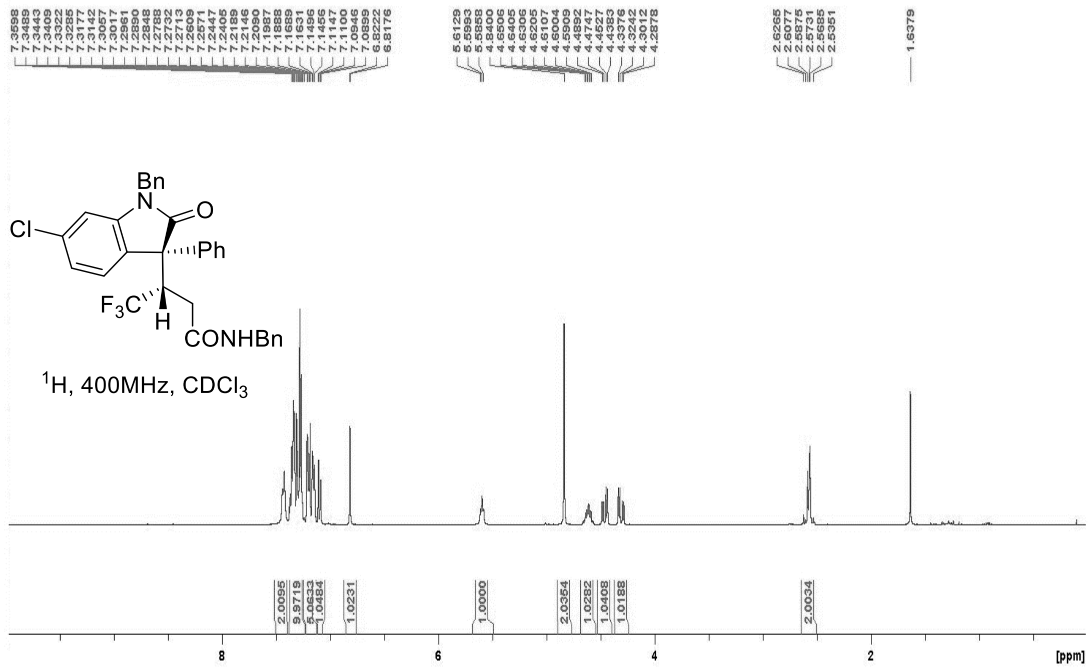

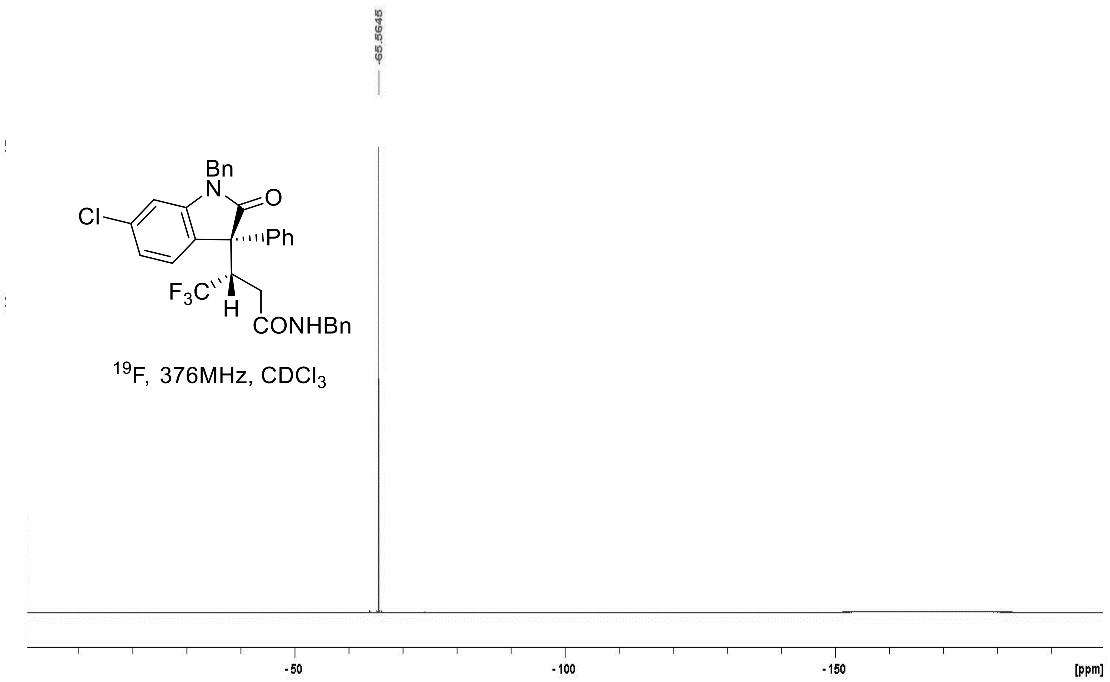

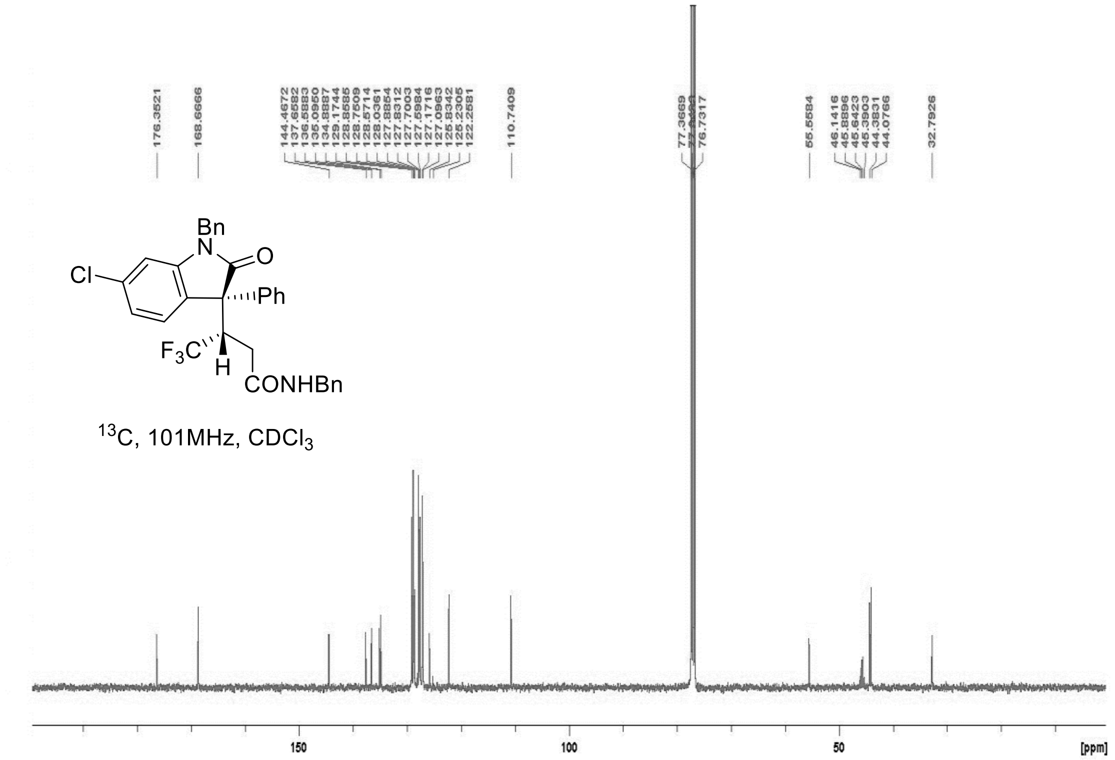

(*S*)-N-benzyl-3-((*R*)-1-benzyl-7-chloro-2-oxo-3-phenylindolin-3-yl)-4,4,4-trifluorobutanamide (61)

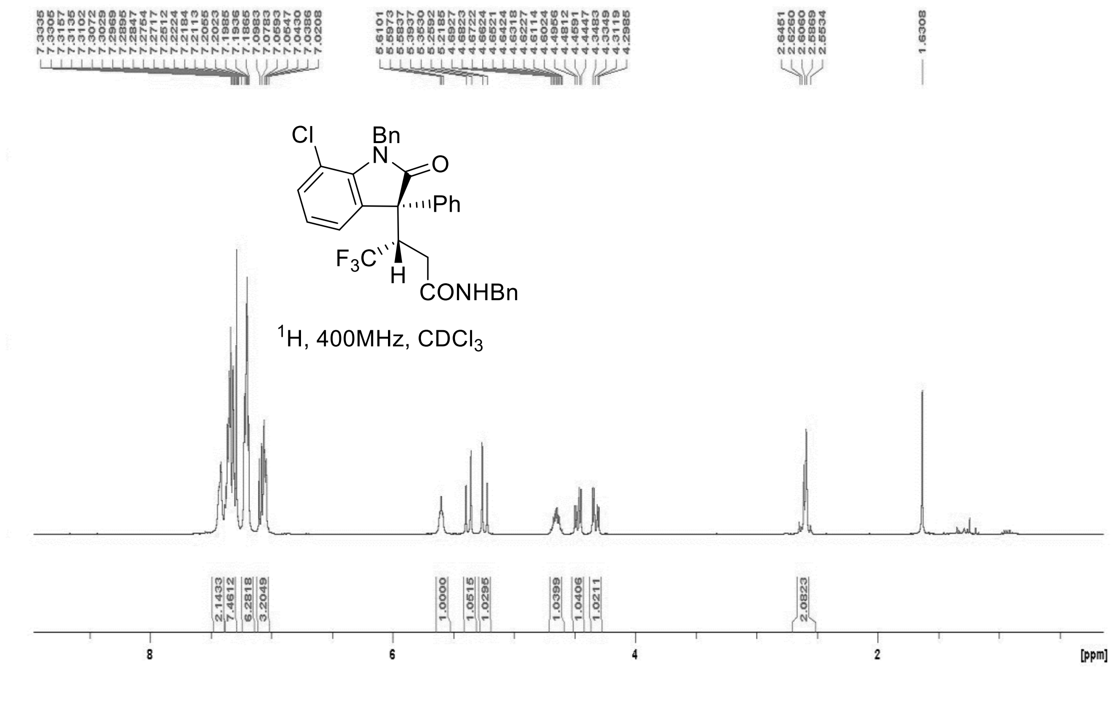

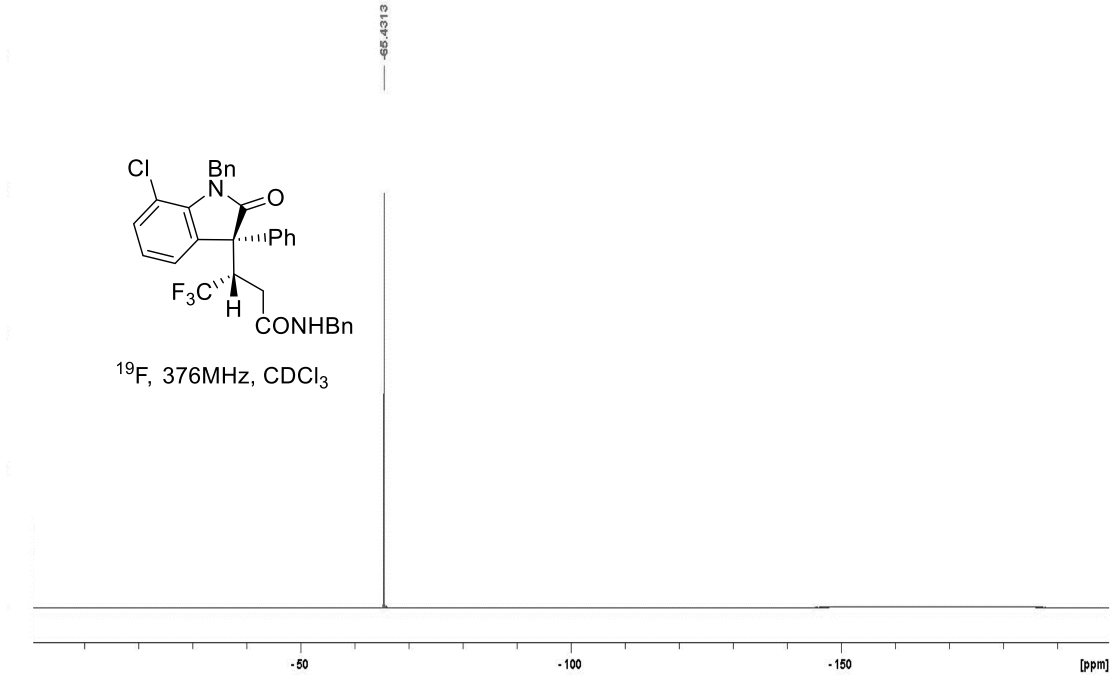
-65.4313
Cl
Bn
N
O
Ph
F3C
H
CONHBn
19F, 376MHz, CDCl3
-50
-100
-150
[ppm]

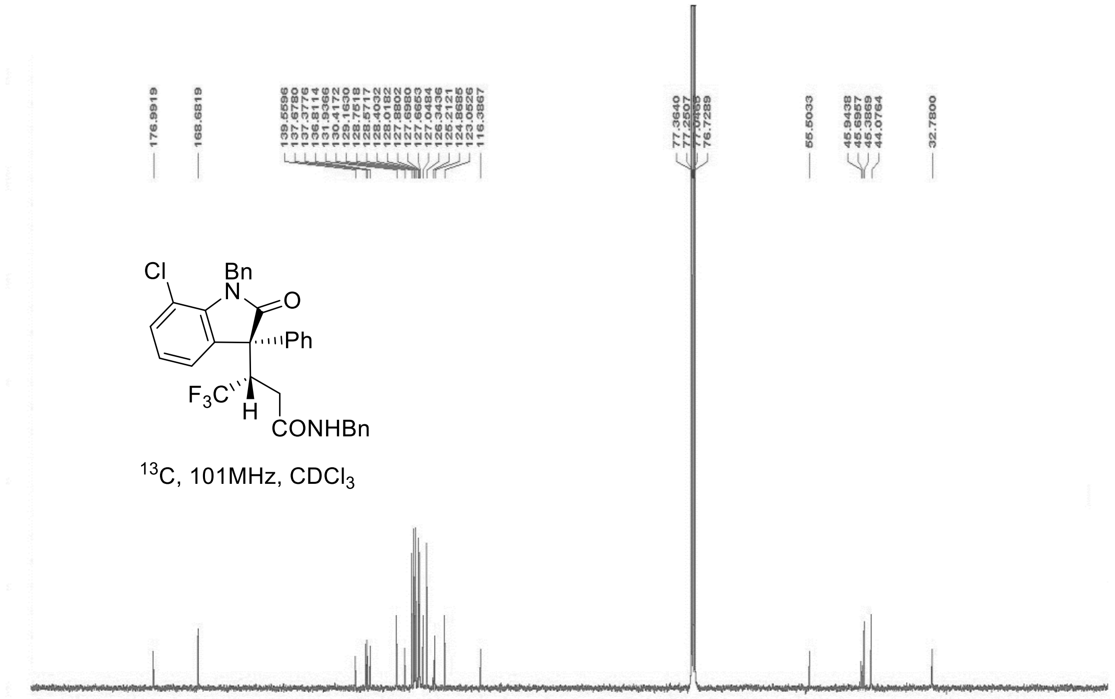
176.9919
168.6819
77.3640
55.5033
32.7800
Cl
Bn
N
O
Ph
F3C
H
CONHBn
13C, 101MHz, CDCl3
150
100
50
[ppm]

(*S*)-N-benzyl-3-((*R*)-1-benzyl-3-(4-methoxyphenyl)-2-oxoindolin-3-yl)-4,4,4-trifluorobutanamide (62)

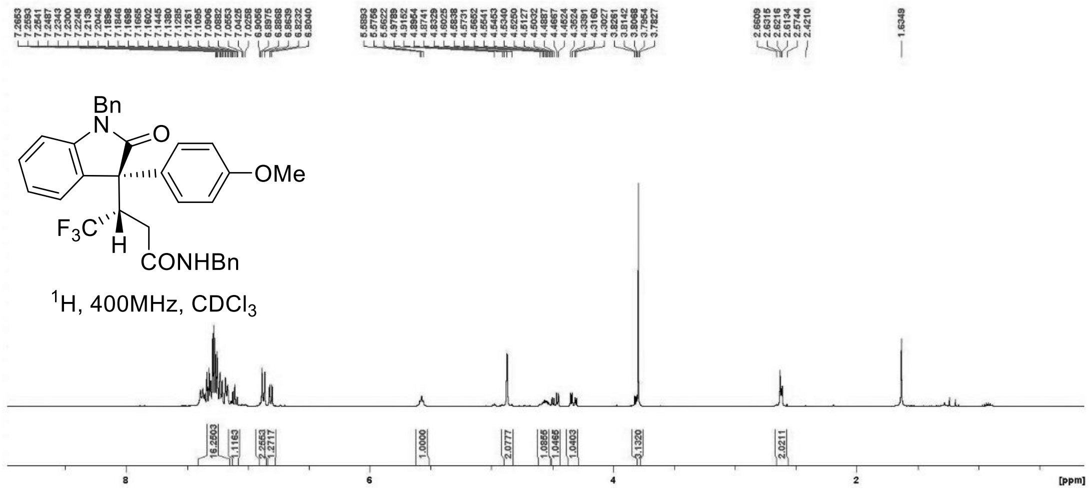

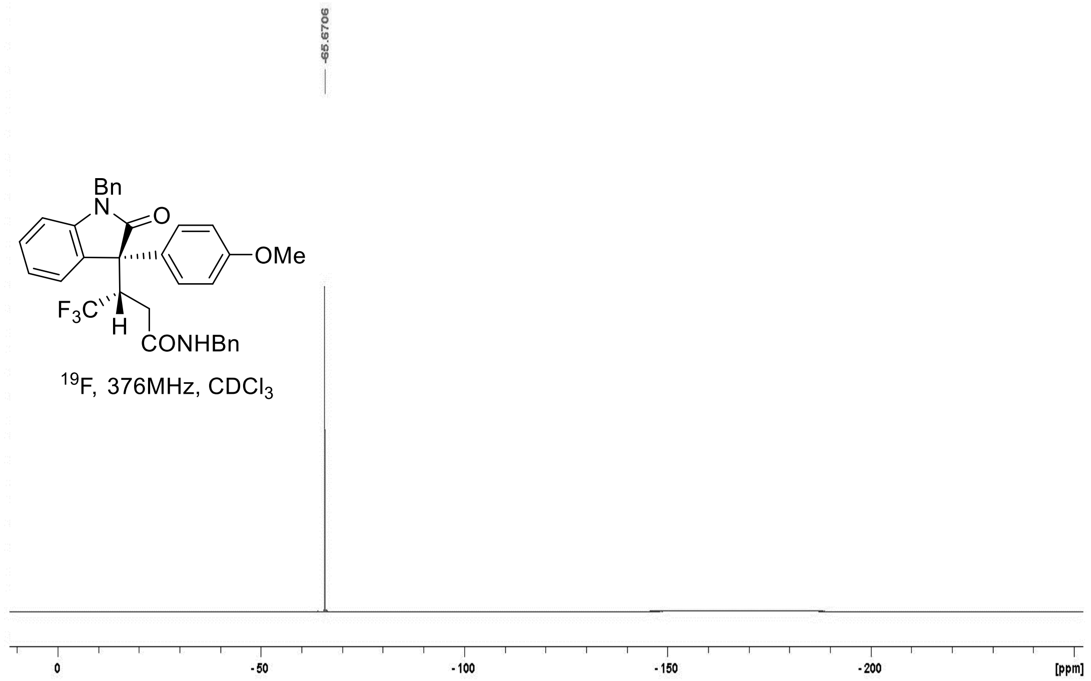

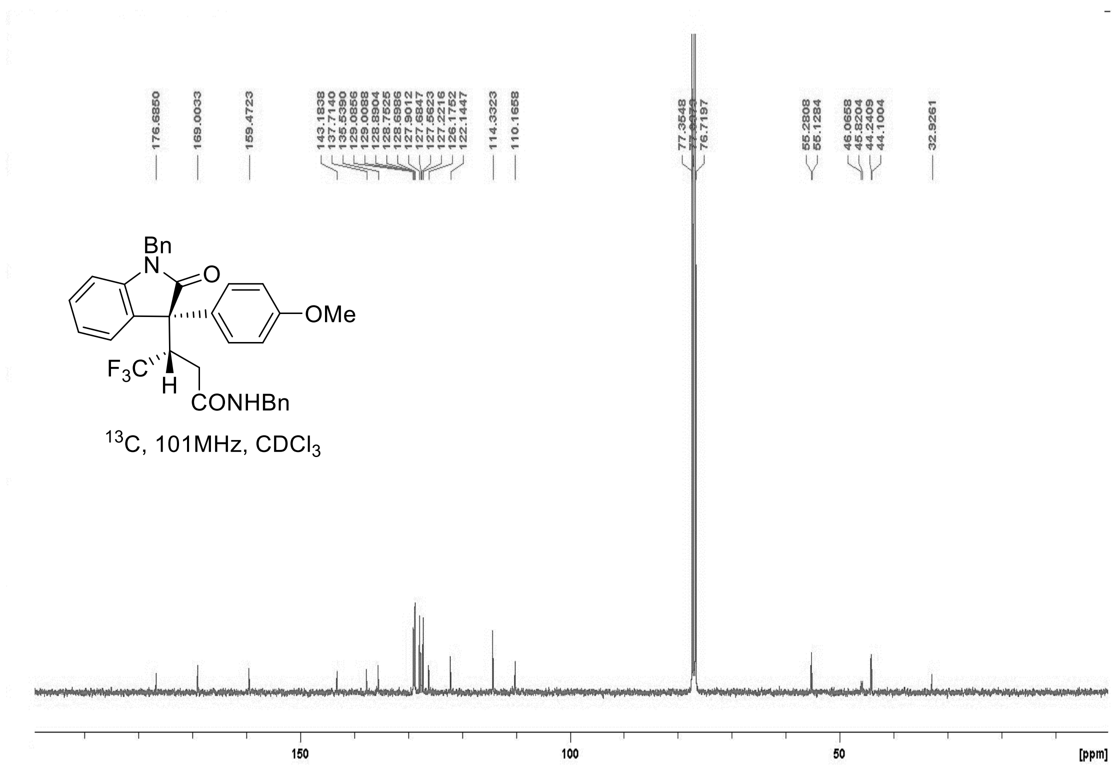

(*S*)-3-((*R*)-3-(benzo[d][1,3]dioxol-5-yl)-1-benzyl-2-oxoindolin-3-yl)-N-benzyl-4,4,4-trifluorobutanamide (63)

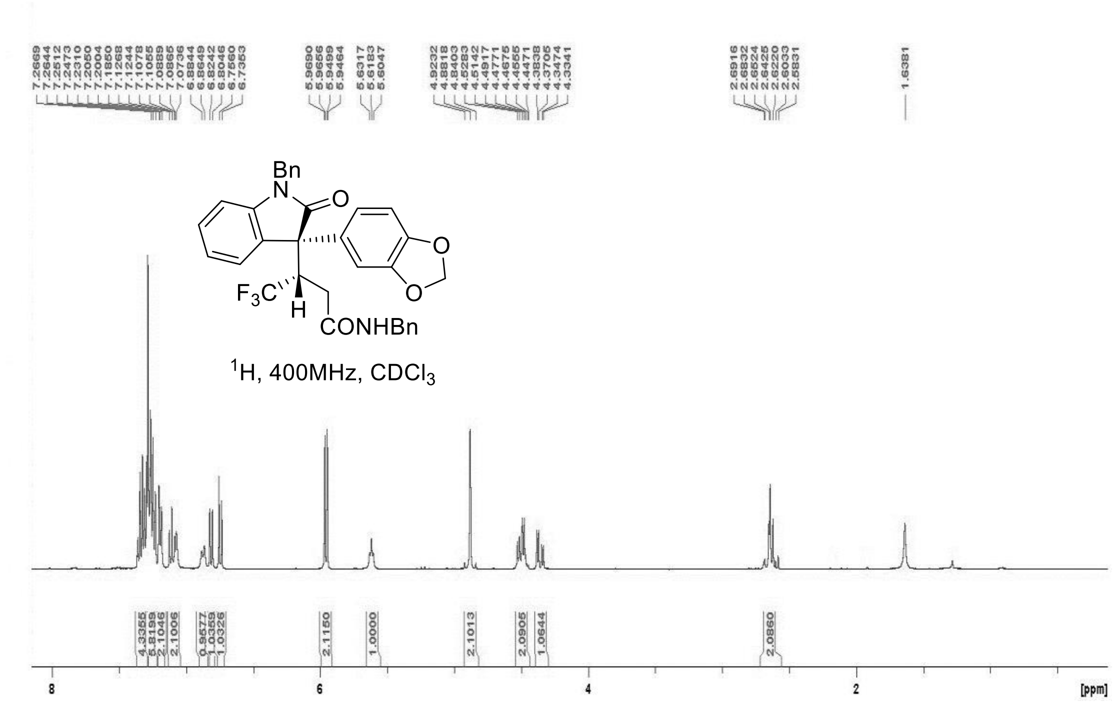

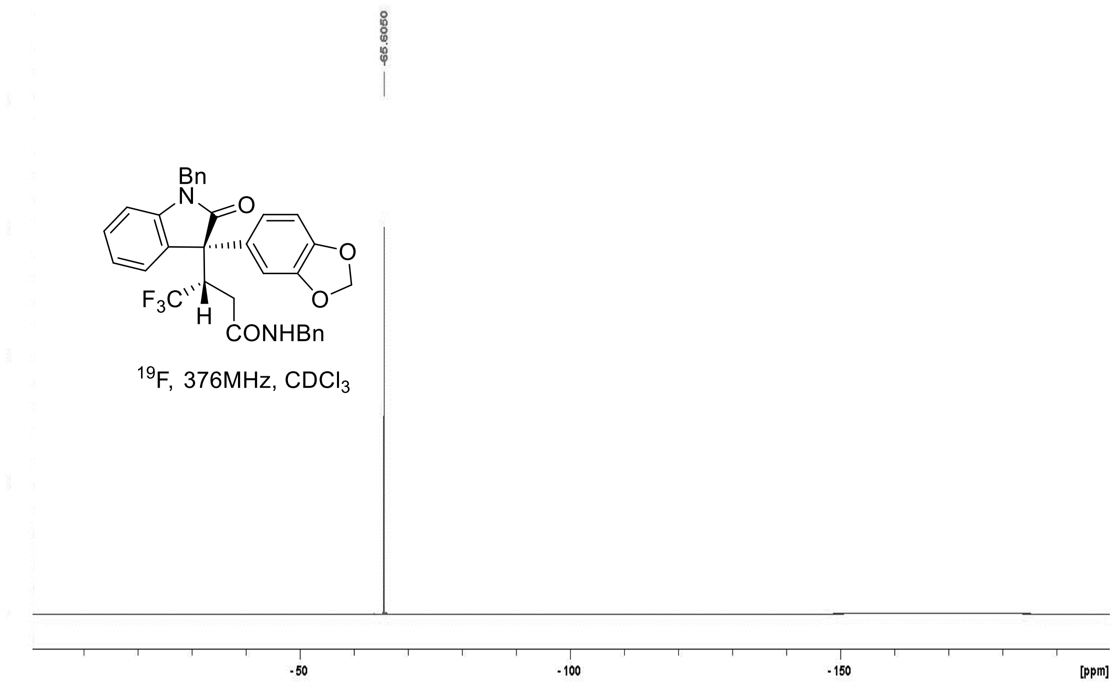
-65.6050
Bn
N
O
O
O
F_3C
H
CONHBn
^{19}F, 376MHz, $CDCl_3$
-50
-100
-150
[ppm]

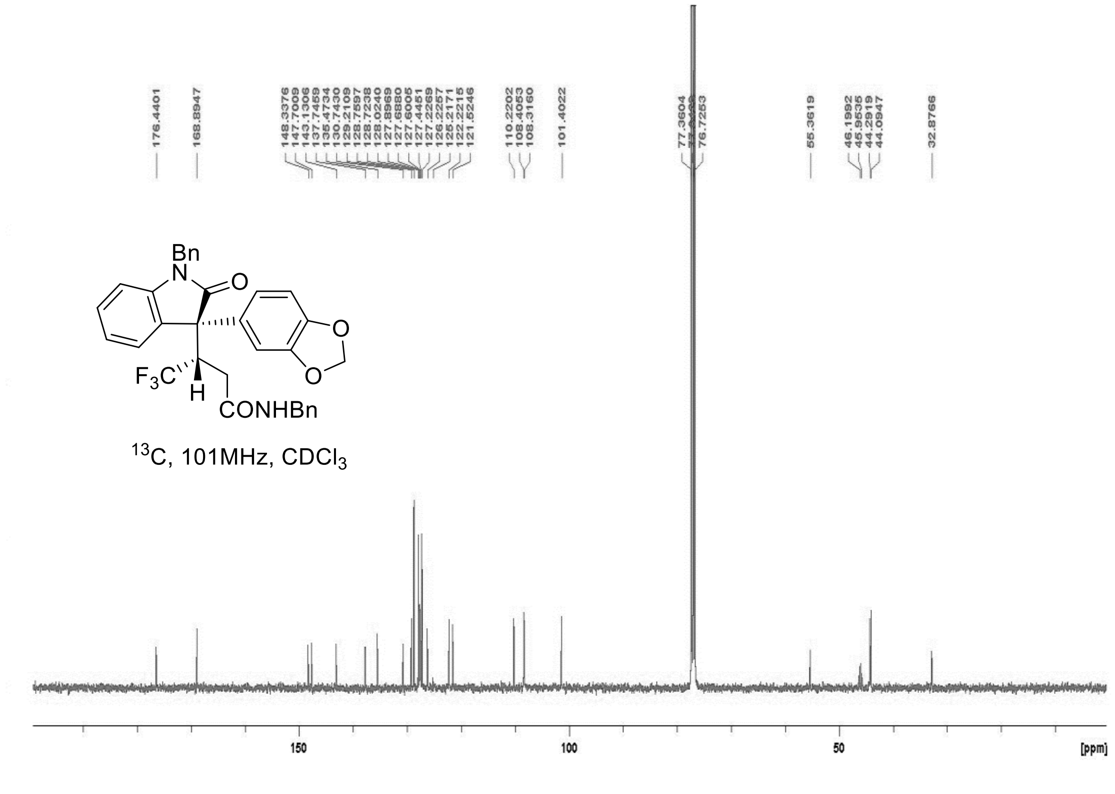
176.4401
168.8947
148.3376
147.7009
143.1306
137.7459
135.4734
130.7430
129.2109
128.7597
128.7238
128.0240
127.8969
127.6880
127.6005
127.4451
127.2269
126.2257
125.2171
122.2215
121.5246
110.2202
108.4053
108.3160
101.4022
77.3604
76.7253
55.3619
46.1992
45.9535
44.2919
44.0947
32.8766
Bn
N
O
O
O
F_3C
H
CONHBn
^{13}C, 101MHz, $CDCl_3$
150
100
50
[ppm]

(*S*)-N-benzyl-3-((*R*)-1-benzyl-3-(naphthalen-2-yl)-2-oxoindolin-3-yl)-4,4,4-trifluorobutanamide (64)

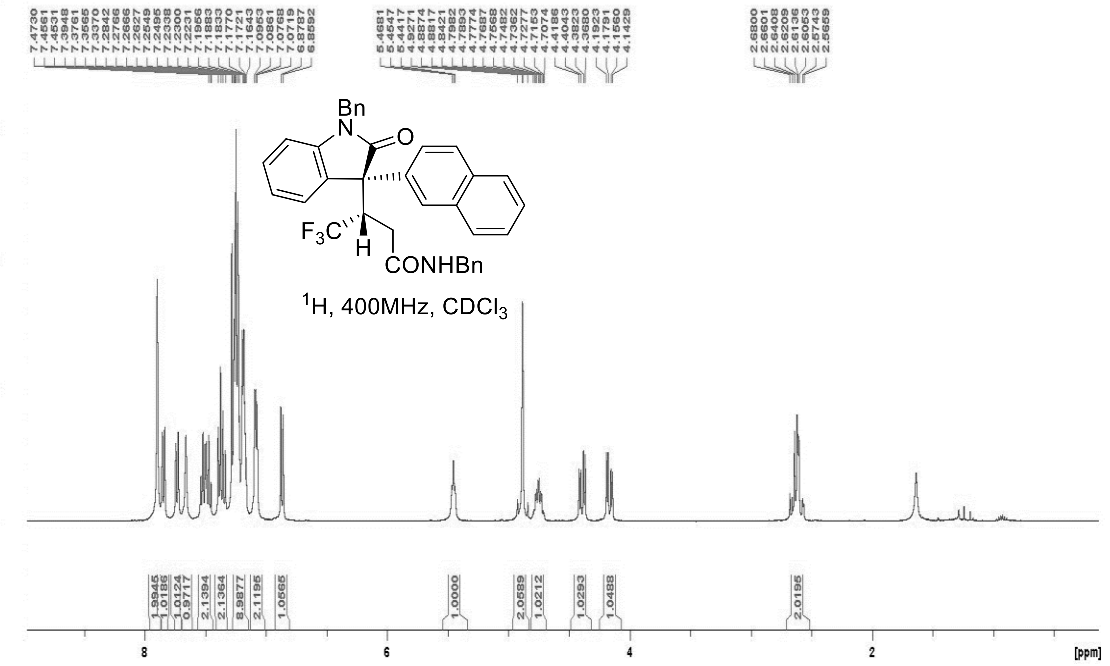

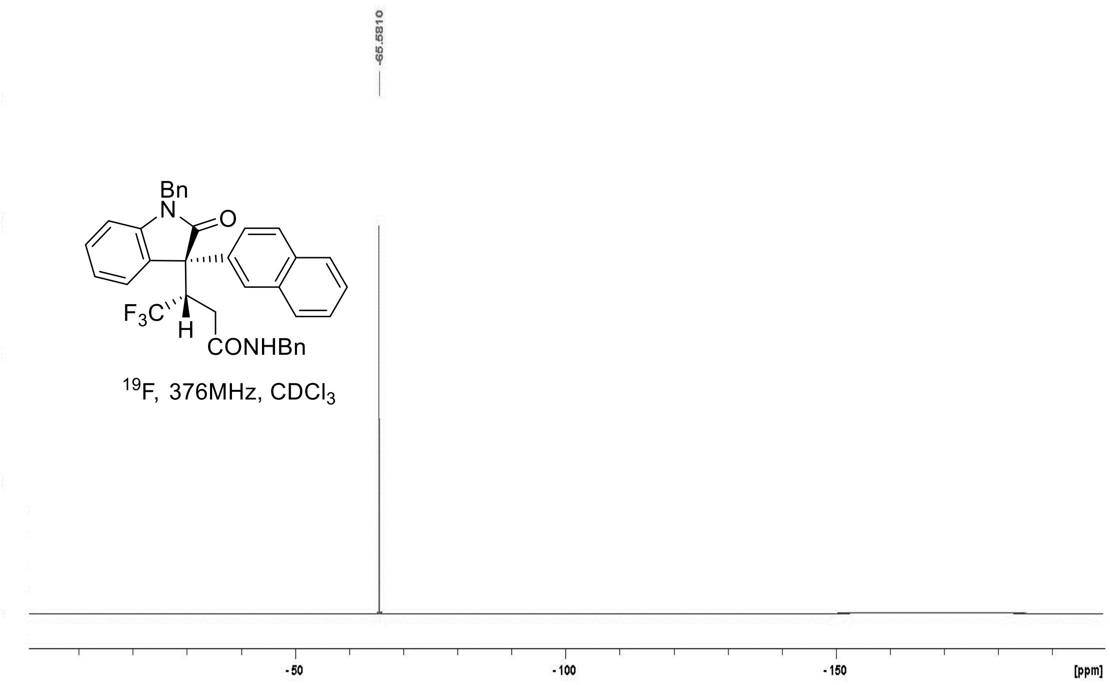

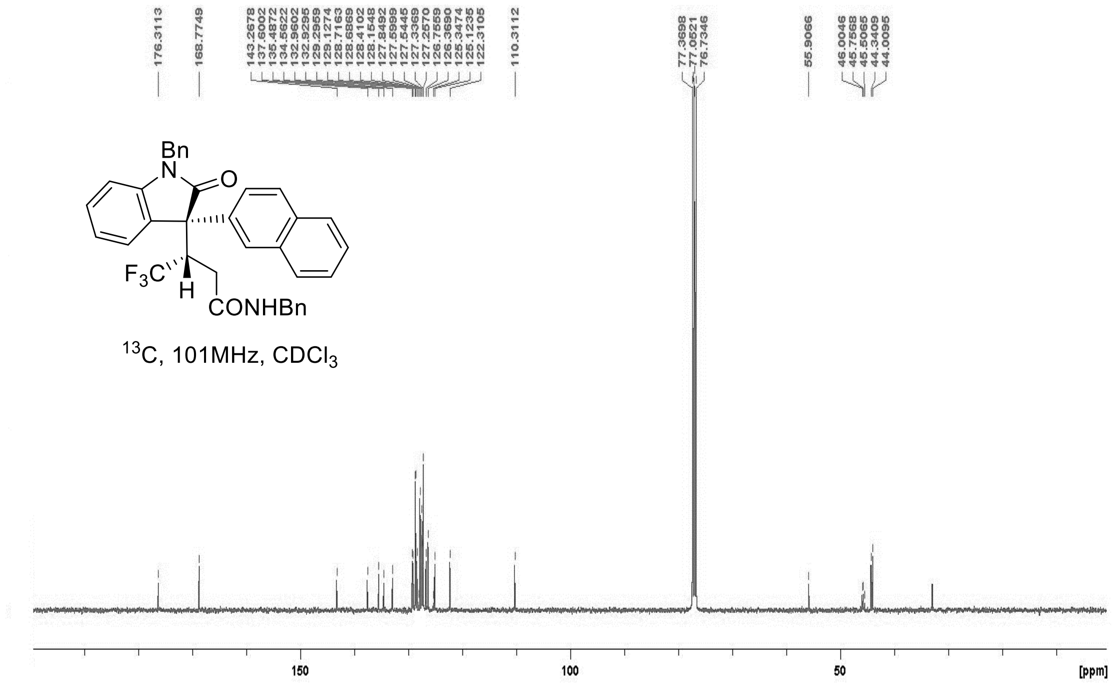

(*S*)-N-benzyl-3-((*R*)-1-benzyl-2-oxo-3-(4-(trifluoromethyl)phenyl)indolin-3-yl)-4,4,4-trifluorobutanamide (65)

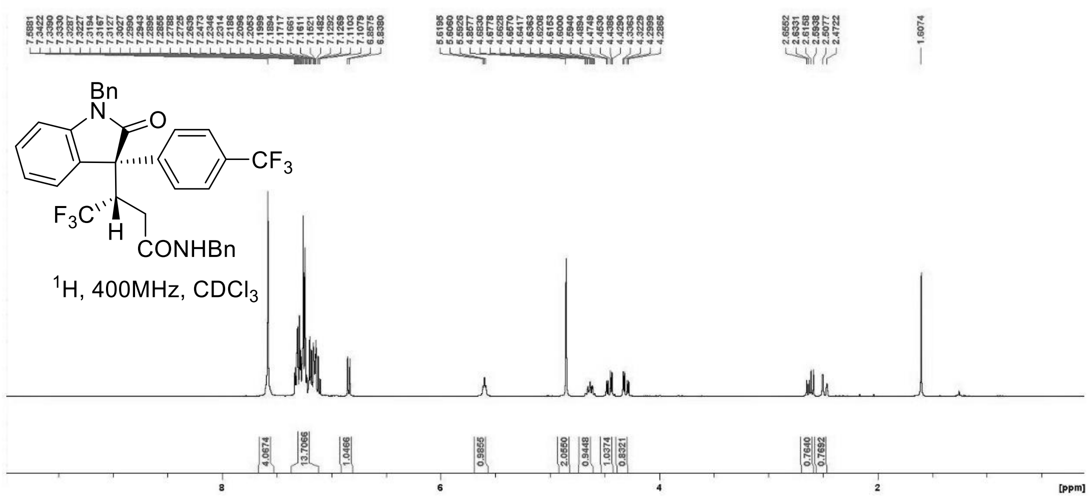

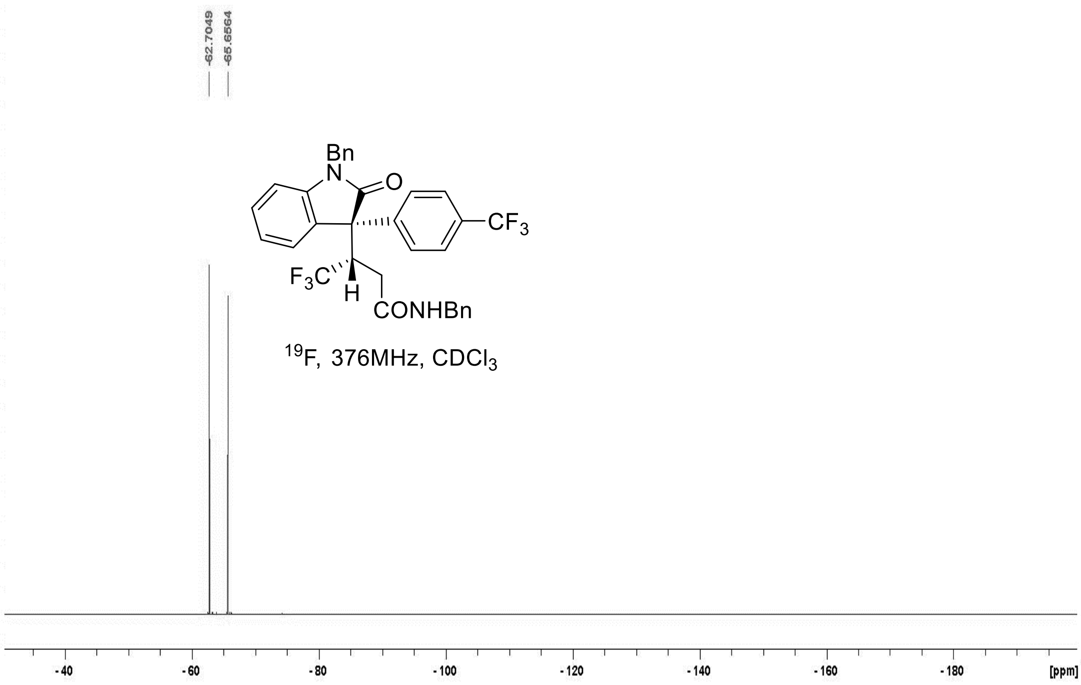

-62.7049
-65.6564
Bn
N
O
CF3
F3C
H
CONHBn
19F, 376MHz, CDCl3
-40
-60
-80
-100
-120
-140
-160
-180
[ppm]

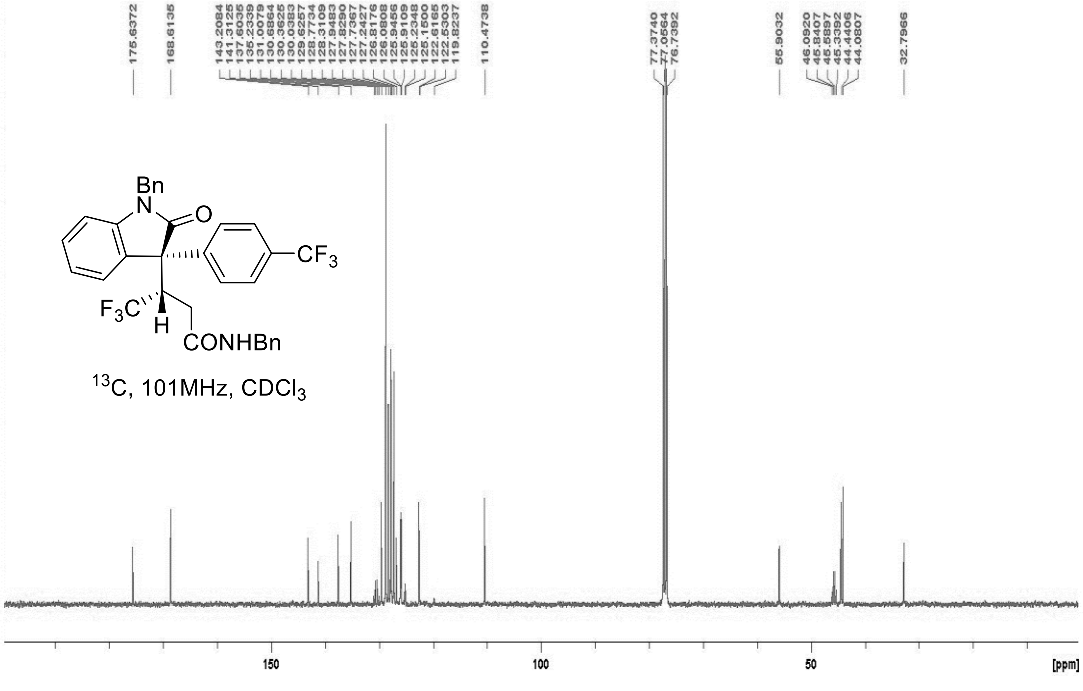

175.6372
168.6135
143.2084
141.3125
137.6035
135.2339
131.0079
130.6884
130.3625
130.0383
129.6257
128.7734
128.3109
127.9483
127.8290
127.7367
127.2427
126.8176
126.0808
125.9456
125.9109
125.2348
125.1500
122.6165
122.5303
119.8237
110.4738
77.3740
77.0564
76.7392
55.9032
46.0920
45.8407
45.5897
45.3392
44.4406
44.0807
32.7966
Bn
N
O
CF3
F3C
H
CONHBn
13C, 101MHz, CDCl3
150
100
50
[ppm]

(*S*)-N-benzyl-3-((*R*)-1-benzyl-3-(4-fluorophenyl)-2-oxoindolin-3-yl)-4,4,4-trifluorobutanamide (66)

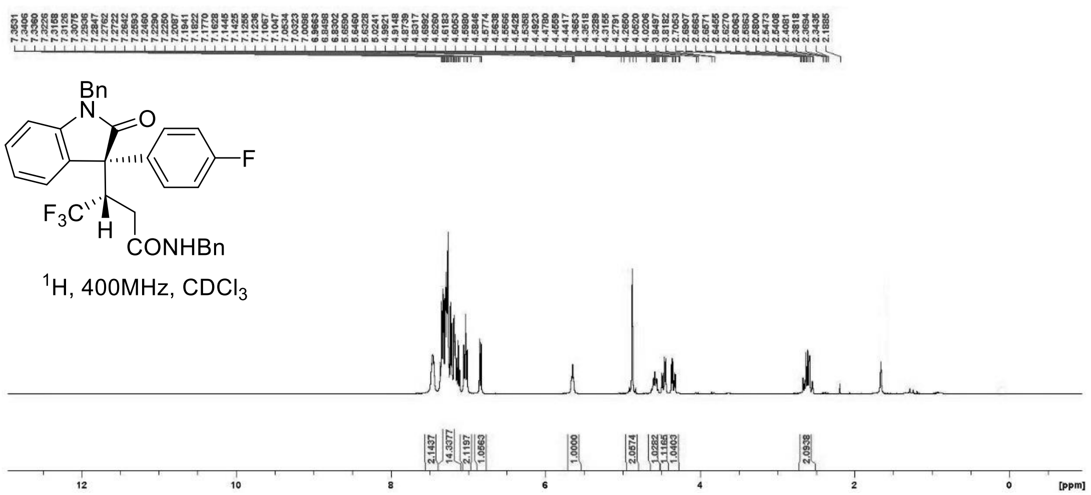

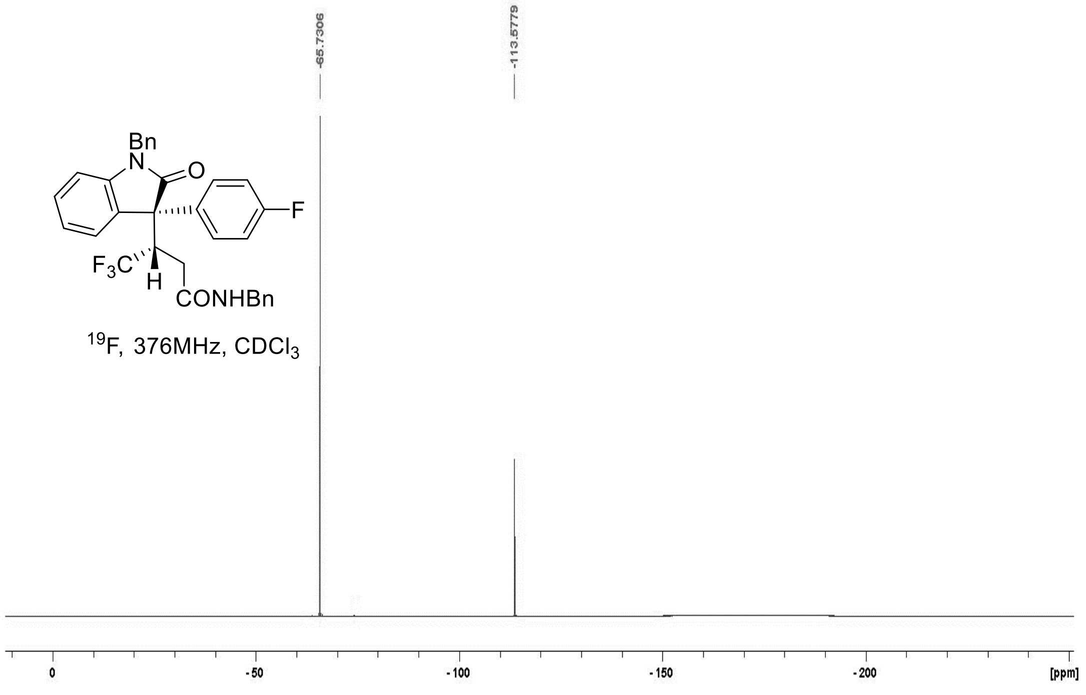

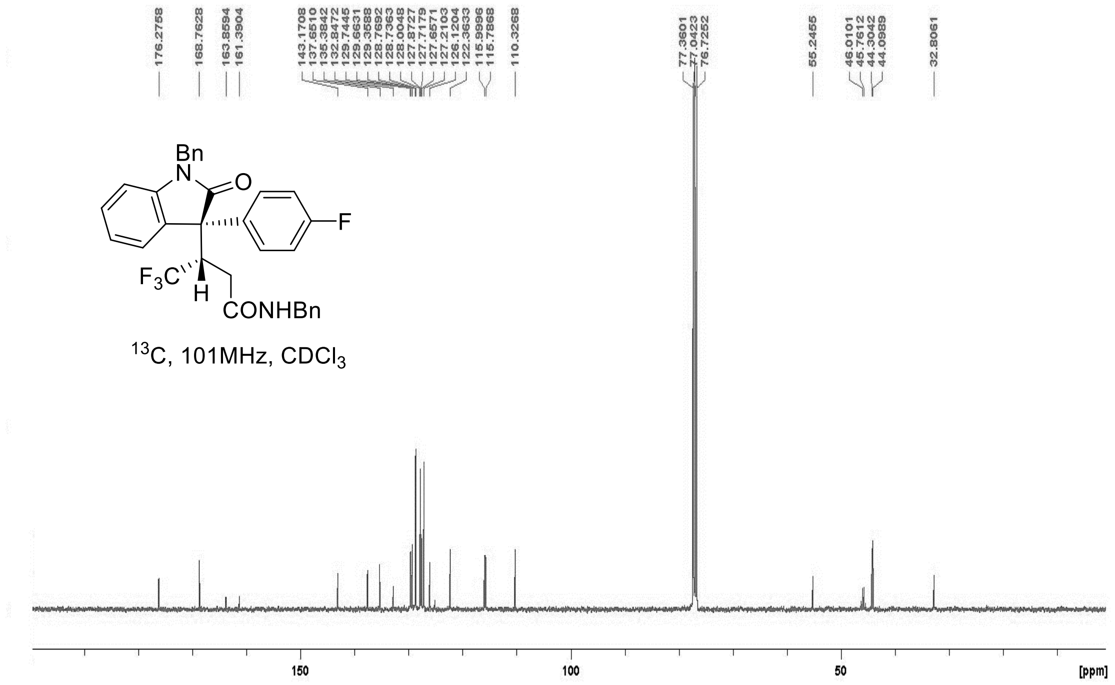

(*S*)-N-benzyl-3-((*R*)-1-benzyl-3-(3-fluorophenyl)-2-oxoindolin-3-yl)-4,4,4-trifluorobutanamide (67)

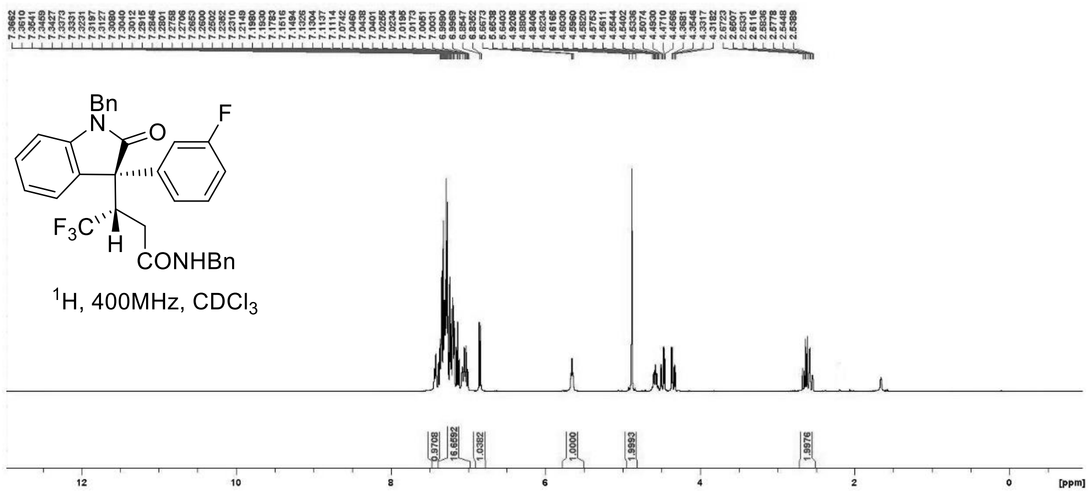

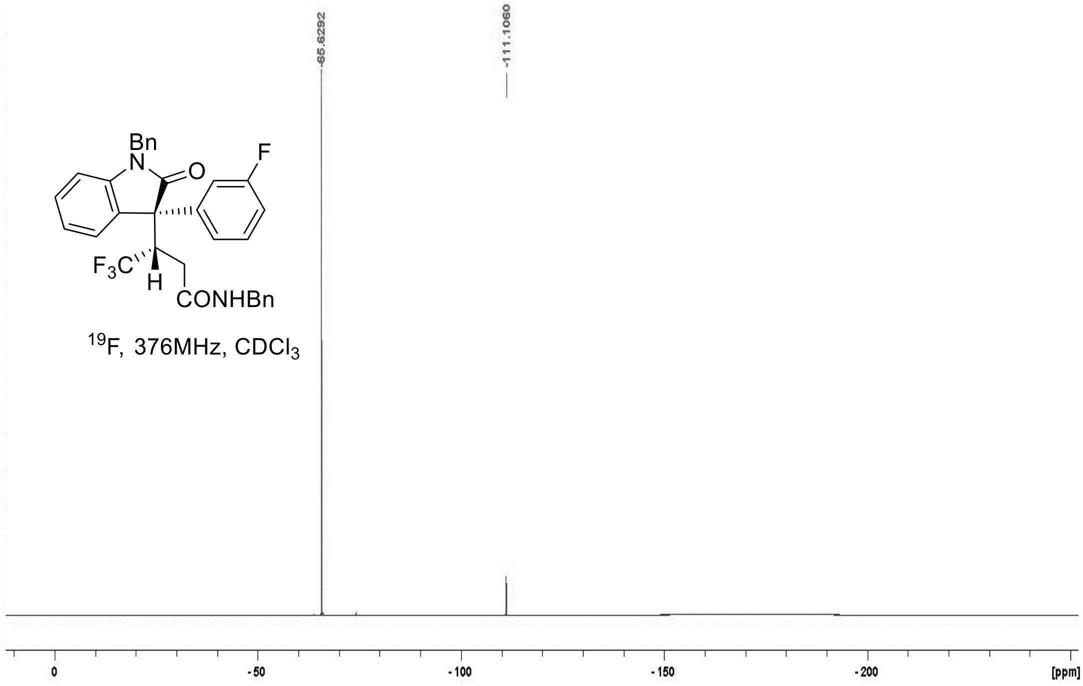

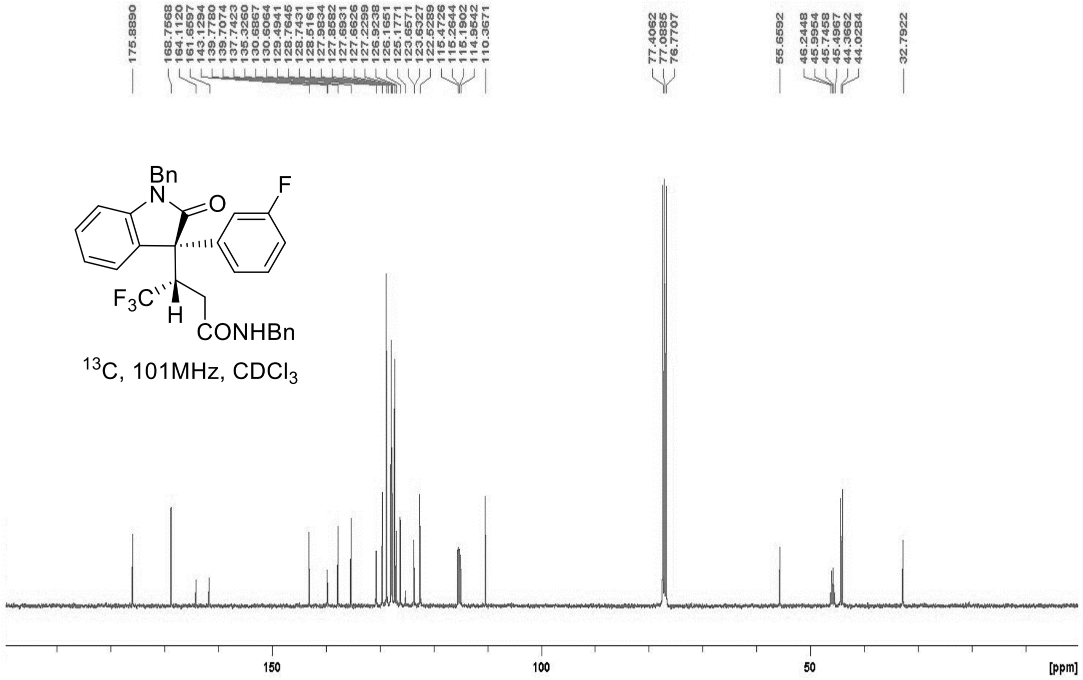

(*S*)-N-benzyl-3-((*R*)-1-benzyl-3-(3-methoxyphenyl)-2-oxoindolin-3-yl)-4,4,4-trifluorobutanamide (68)

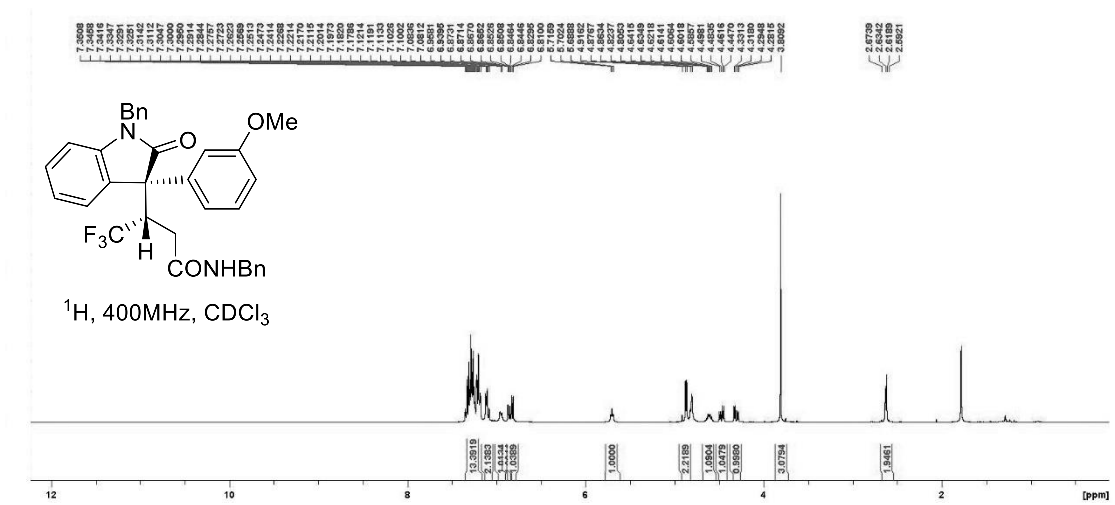

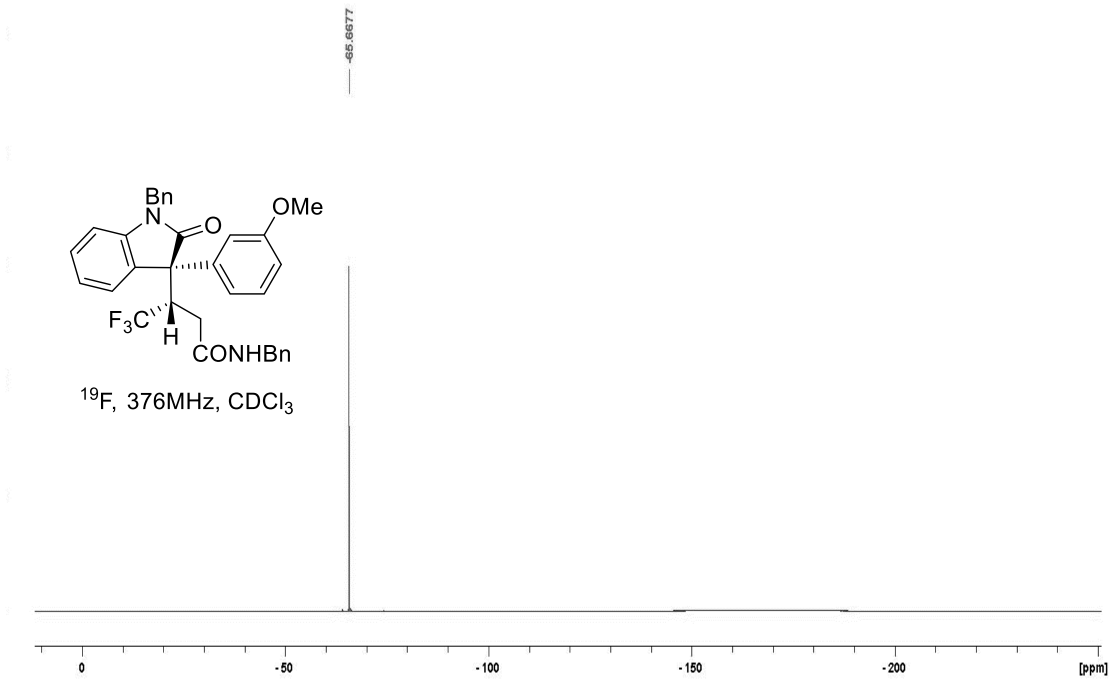

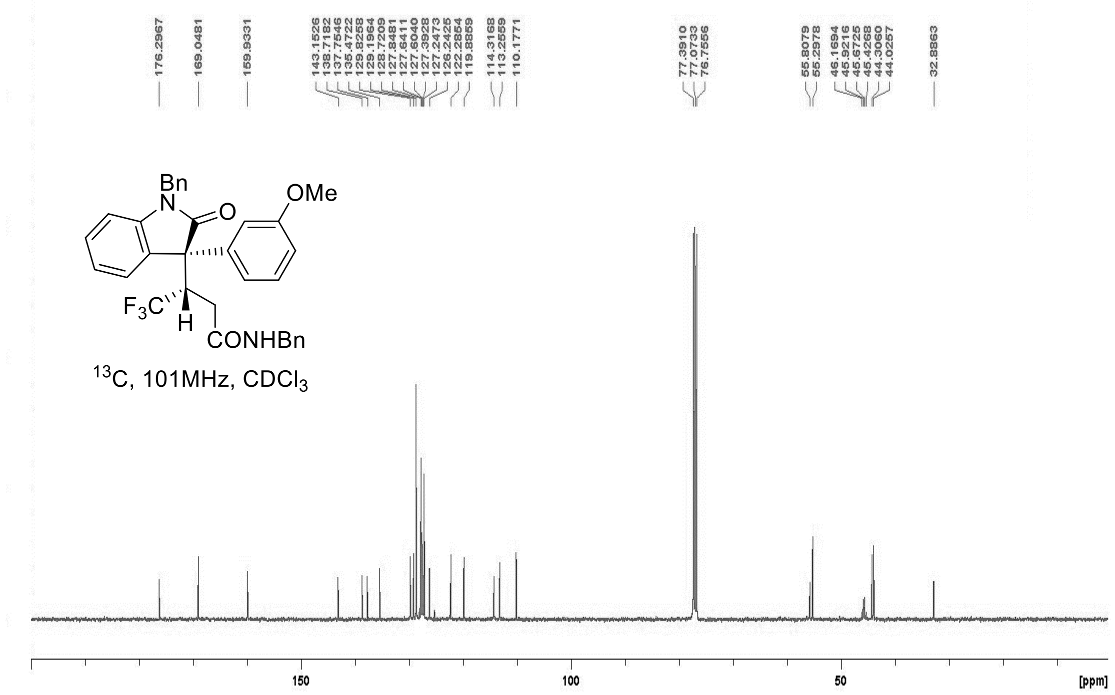

(*S*)-N-benzyl-3-((*R*)-1-benzyl-2-oxo-3-(thiophen-2-yl)indolin-3-yl)-4,4,4-trifluorobutanamide (69)

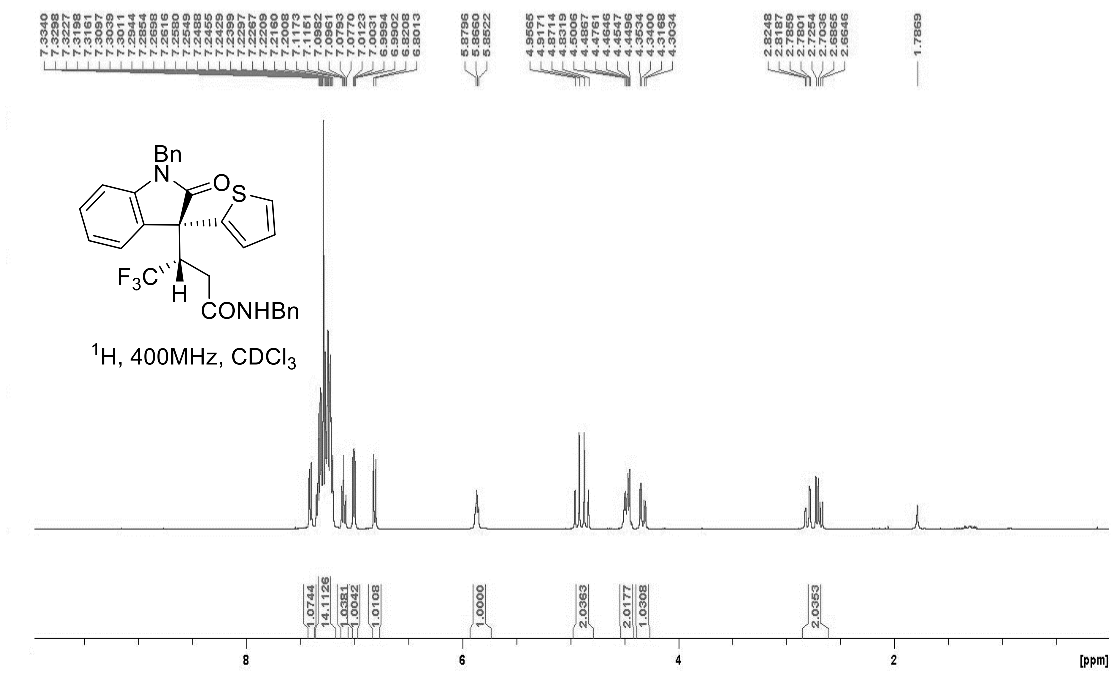

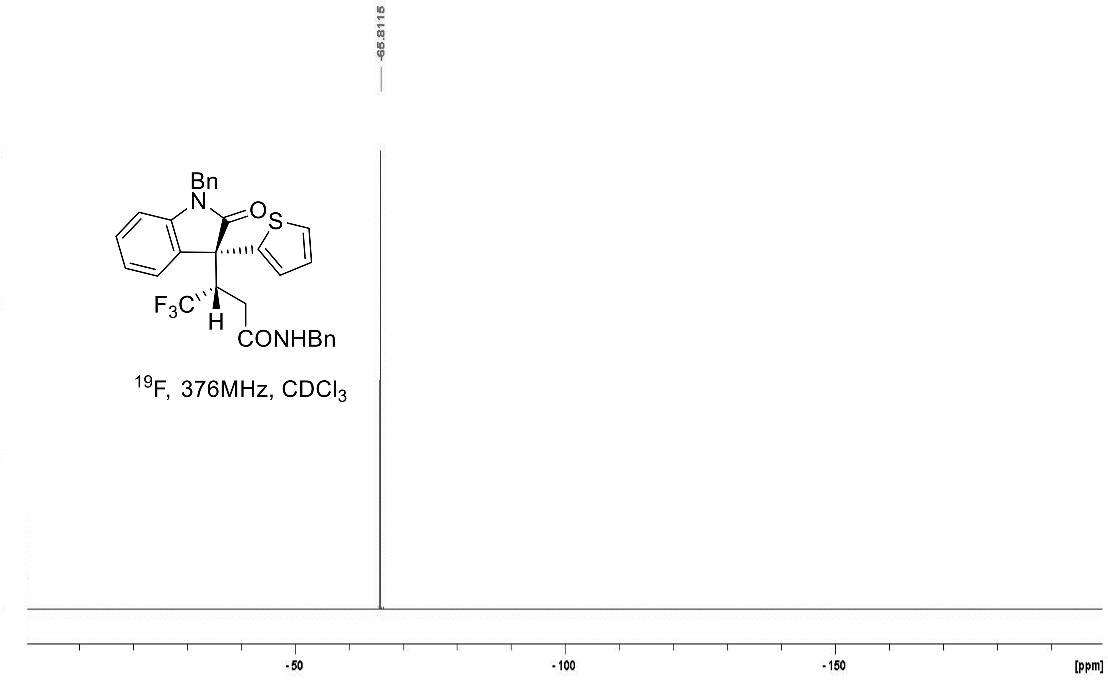
-65.8115
Bn
N
O
S
F3C
H
CONHBn
19F, 376MHz, CDCl3
-50
-100
-150
[ppm]

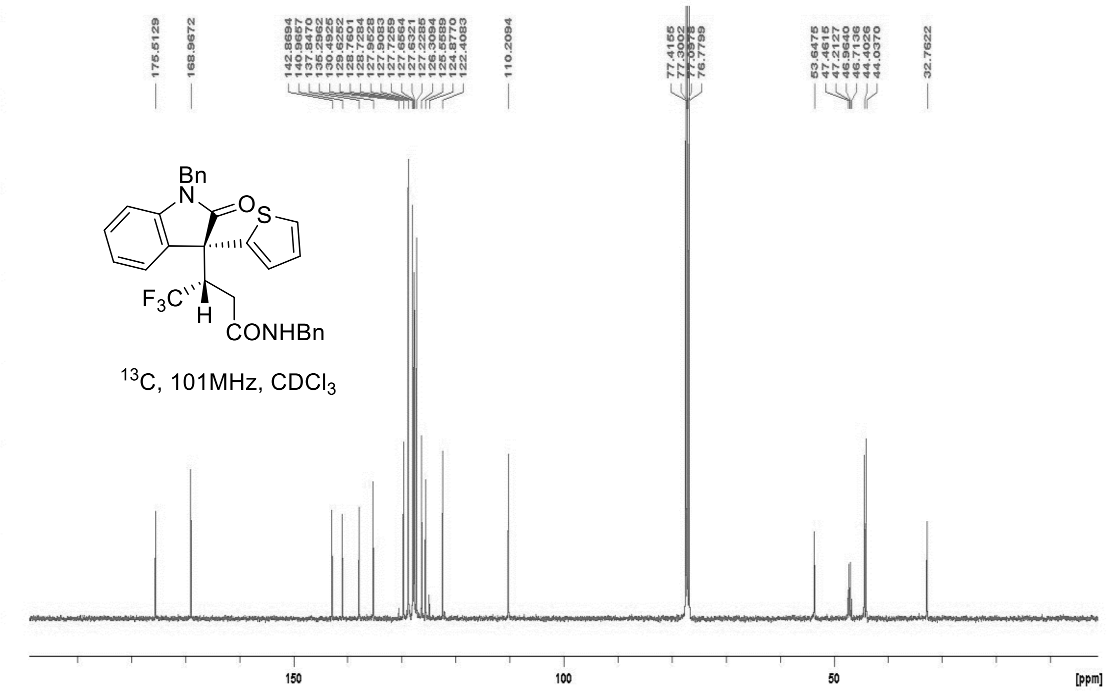
Bn
N
O
S
F3C
H
CONHBn
13C, 101MHz, CDCl3
150
100
50
[ppm]

(*S*)-N-benzyl-3-((*R*)-1-benzyl-2-oxo-3-phenylindolin-3-yl)-4,4-difluoro-Butanamide (76)

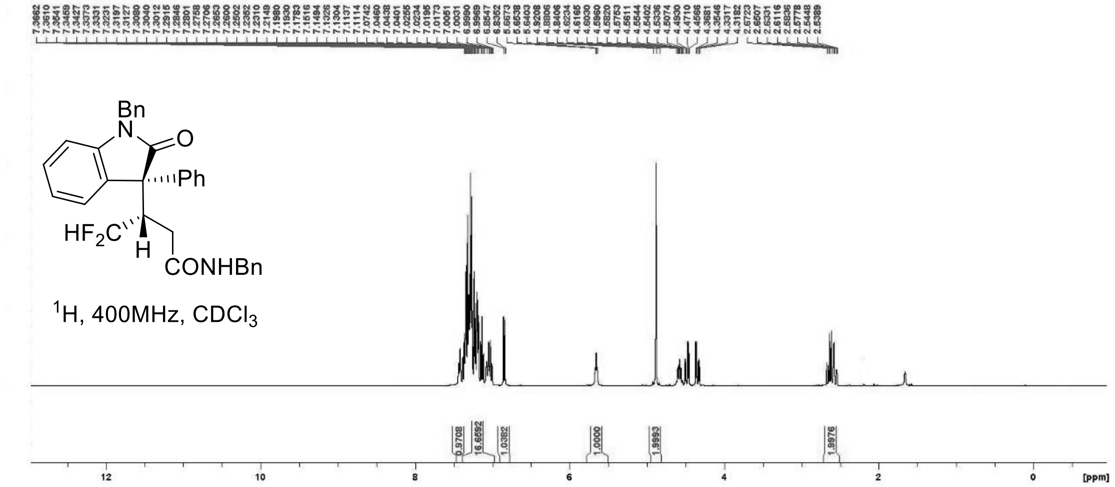

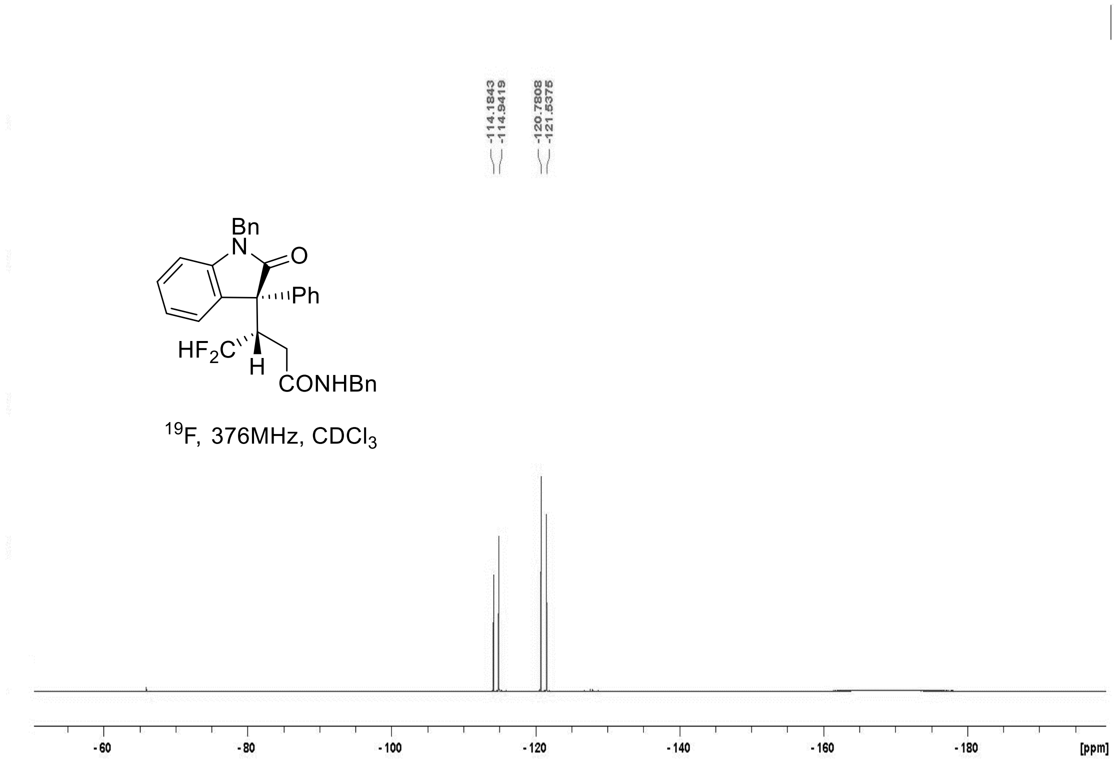

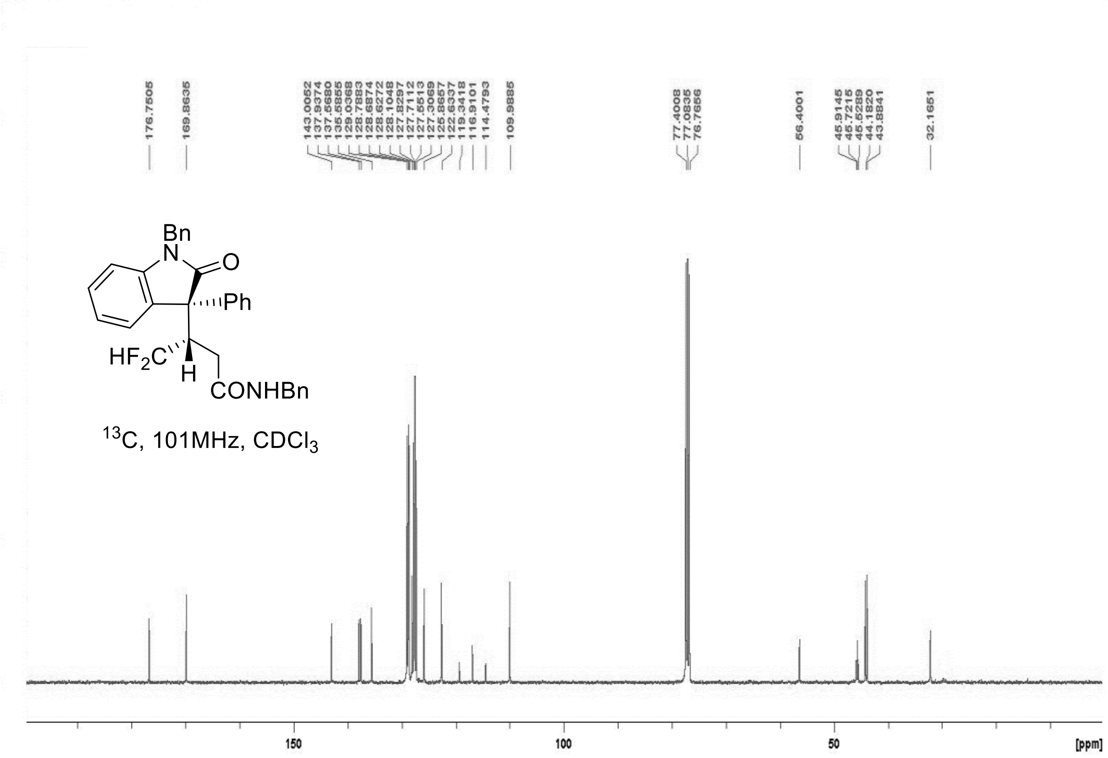

(*S*)-N-benzyl-3-((*R*)-1-benzyl-2-oxo-3-phenylindolin-3-yl)-4-chloro-4,4-difluorobutanamide (77)

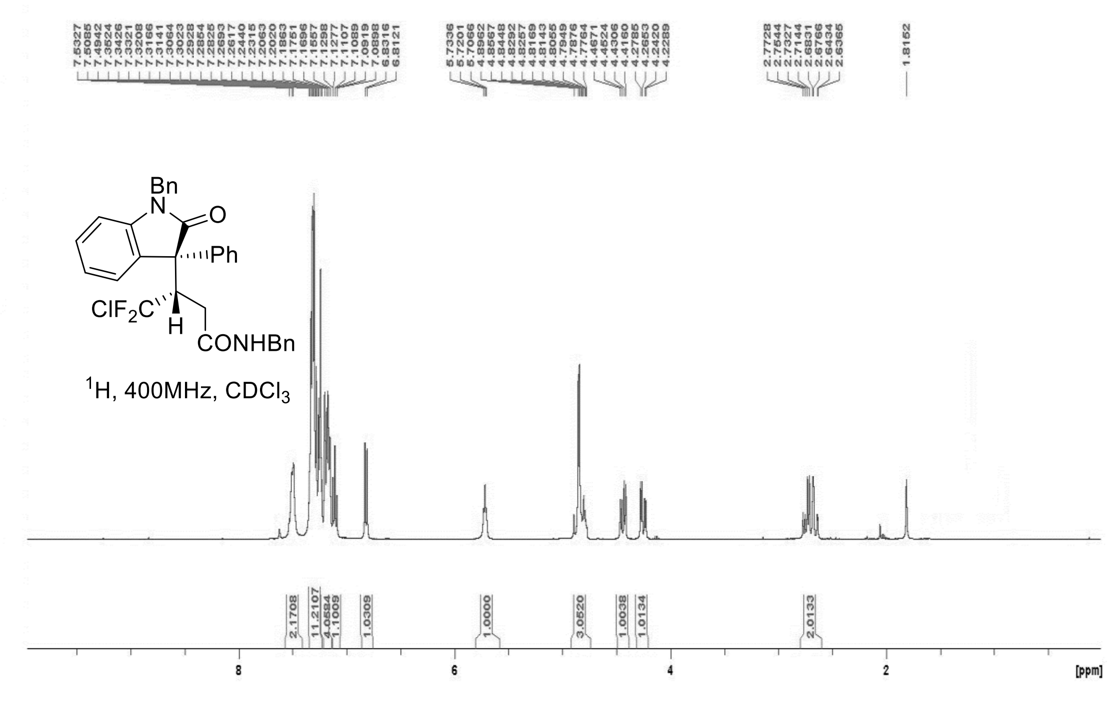

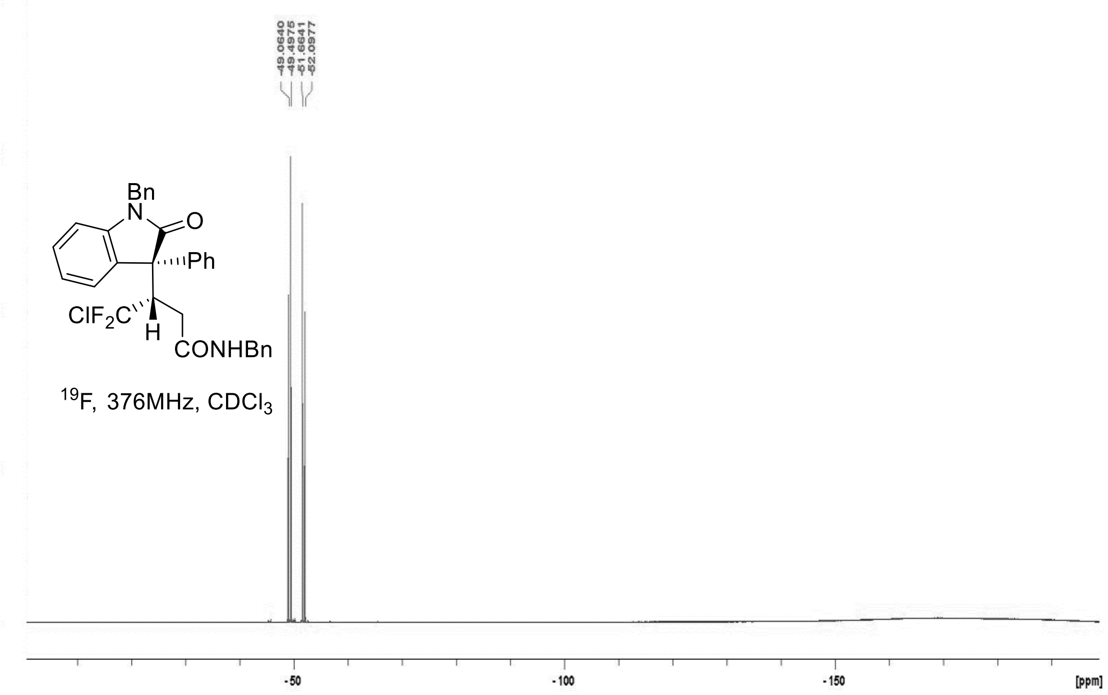
-49.0640
-49.4975
-51.6641
-52.0977
Bn
N
O
Ph
ClF_2C
H
CONHBn
^{19}F, 376MHz, $CDCl_3$
-50
-100
-150
[ppm]

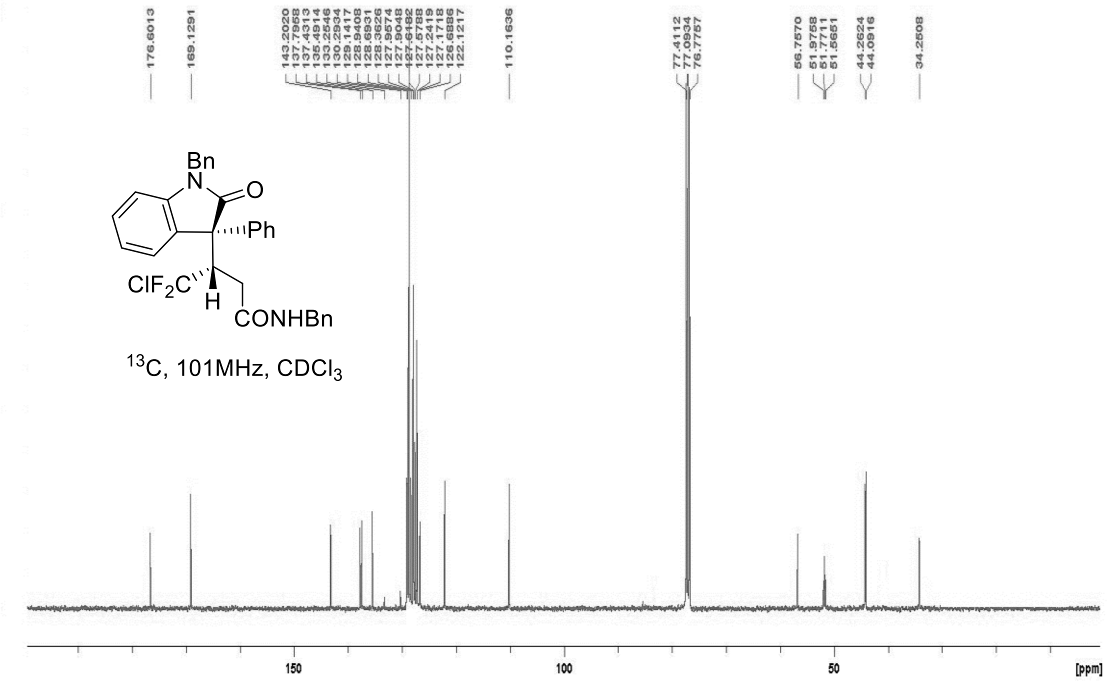
176.6013
169.1291
143.2020
137.7968
137.4313
135.4914
133.2546
130.2934
129.1417
128.9408
128.6931
128.3626
127.9574
127.9048
127.6182
127.5788
127.2419
127.1718
126.6886
122.1217
110.1636
77.4112
77.0934
76.7757
56.7570
51.9758
51.7711
51.5651
44.2624
44.0916
34.2508
Bn
N
O
Ph
ClF_2C
H
CONHBn
^{13}C, 101MHz, $CDCl_3$
150
100
50
[ppm]

(*S*)-N-benzyl-3-((*R*)-1-benzyl-2-oxo-3-phenylindolin-3-yl)-4-bromo-4,4-difluorobutanamide (78)

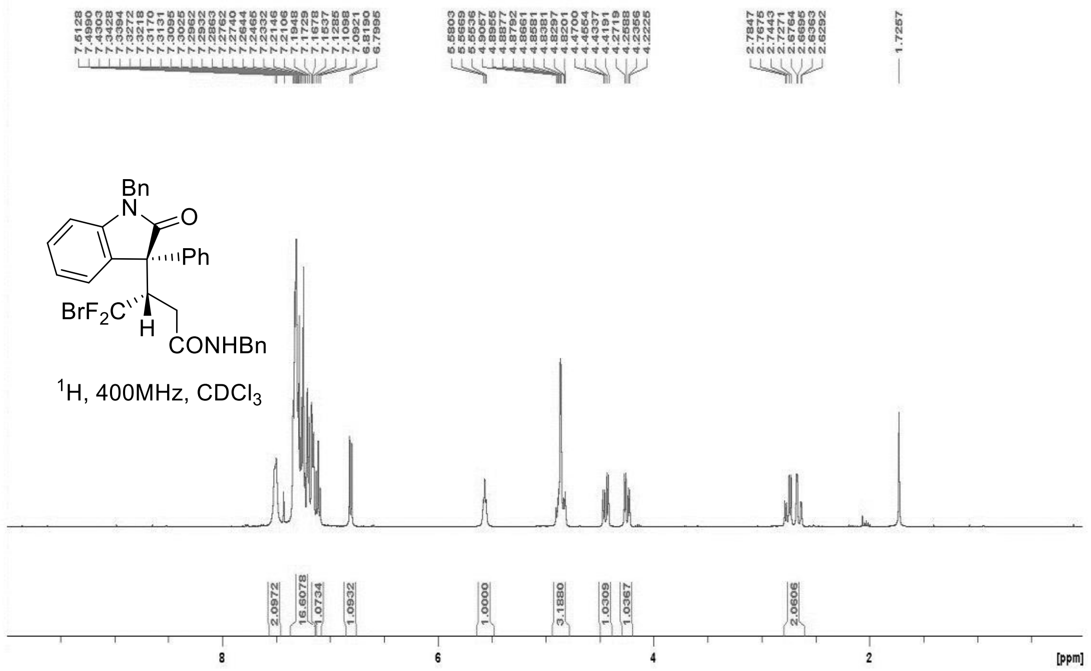

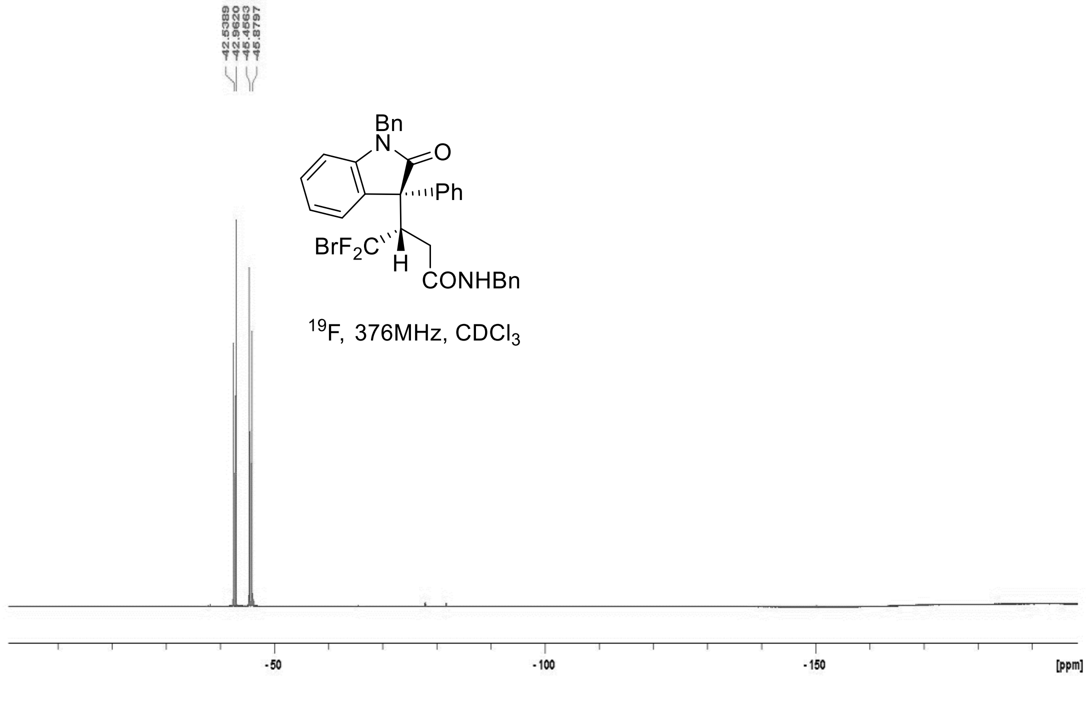

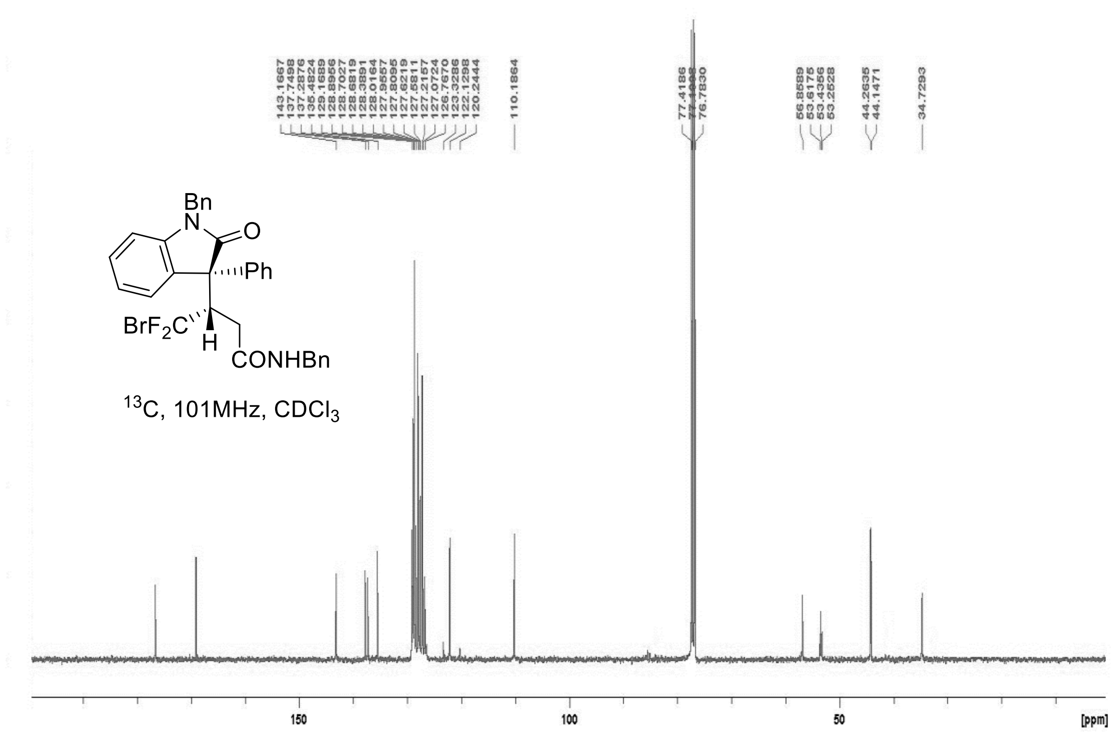

(*S*)-N-benzyl-3-((*R*)-1-benzyl-2-oxo-3-phenylindolin-3-yl)butanamide (79)

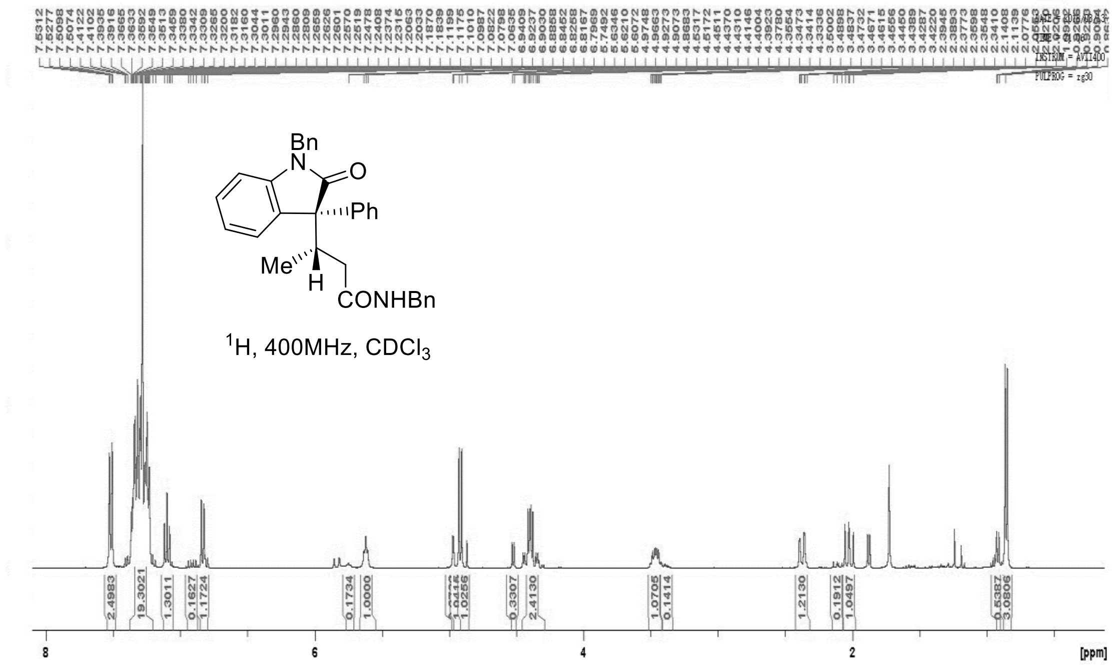

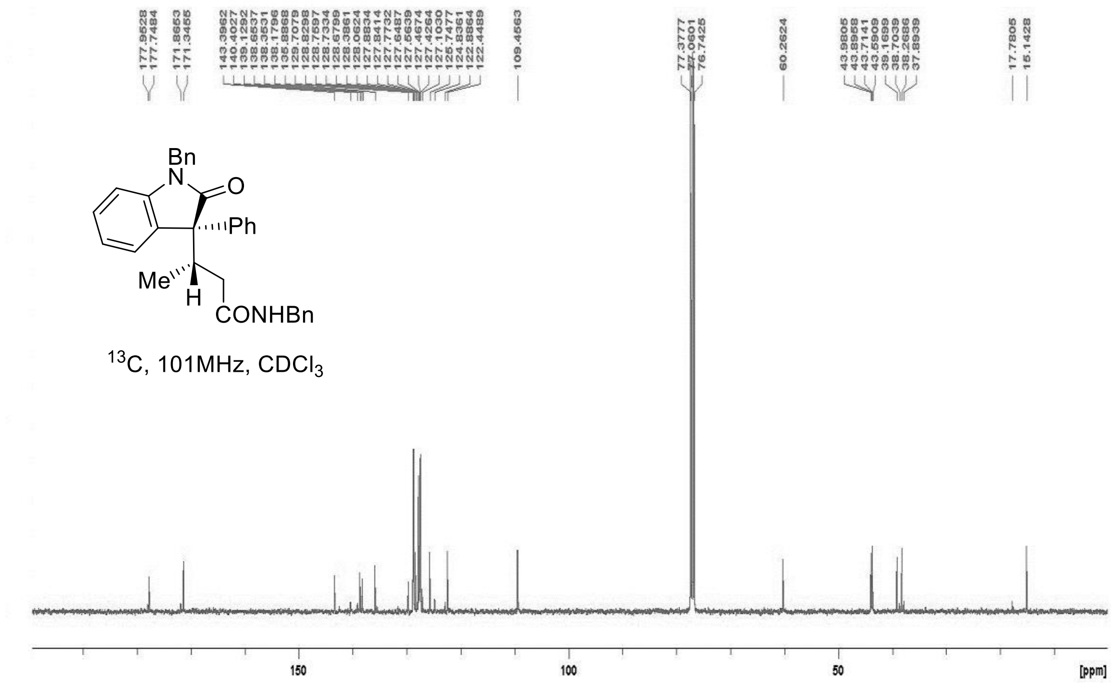

(*R*)-N-benzyl-3-((*R*)-1-benzyl-2-oxo-3-phenylindolin-3-yl)-3-phenylpropanamide (80$_{major}$) and (*S*)-N-benzyl-3-((*R*)-1-benzyl-2-oxo-3-phenylindolin-3-yl)-3-phenylpropanamide (80$_{minor}$)

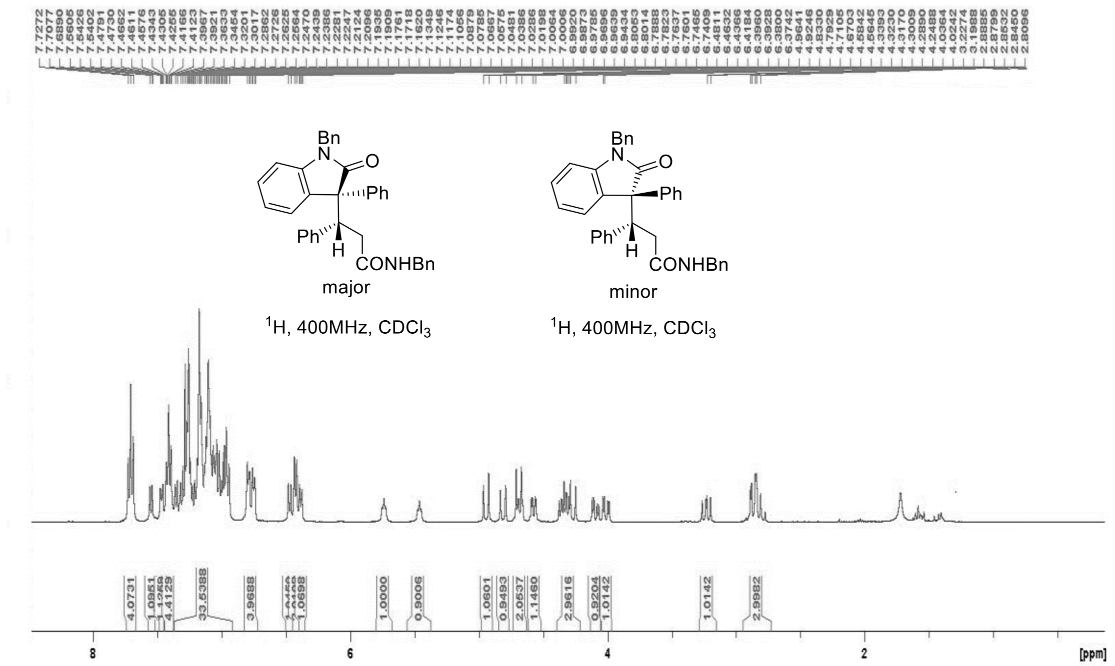

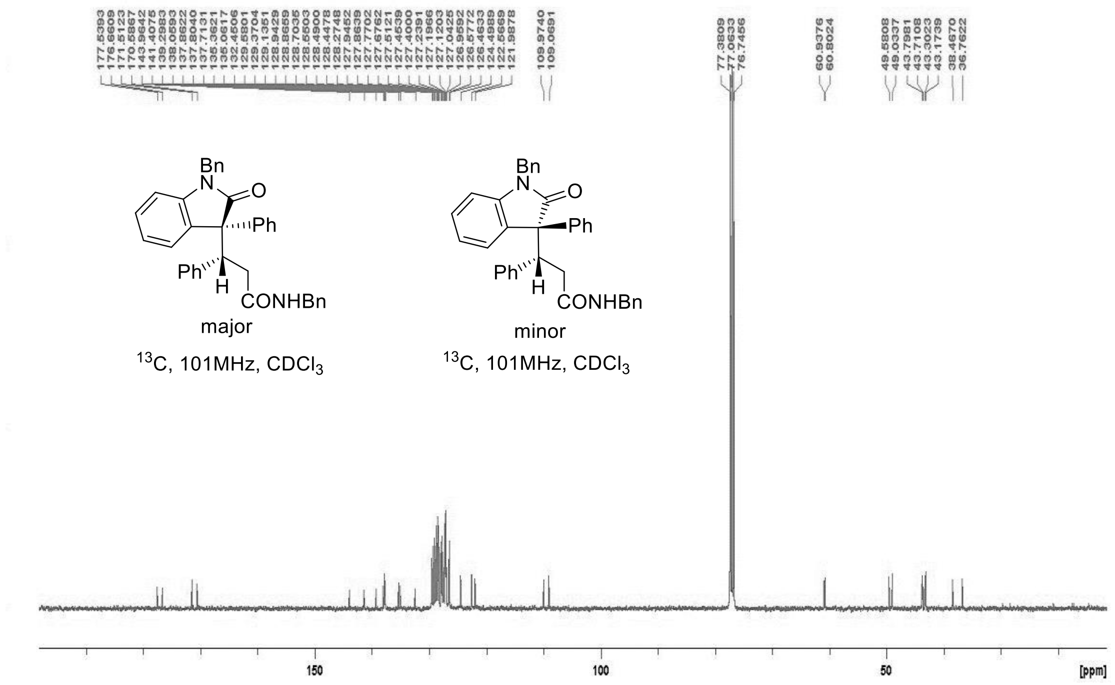

(*R*)-*N*-benzyl-3-((*R*)-1-benzyl-2-oxo-3-phenylindolin-3-yl)-3-(*p*-tolyl)propanamide (S3$_{major}$)

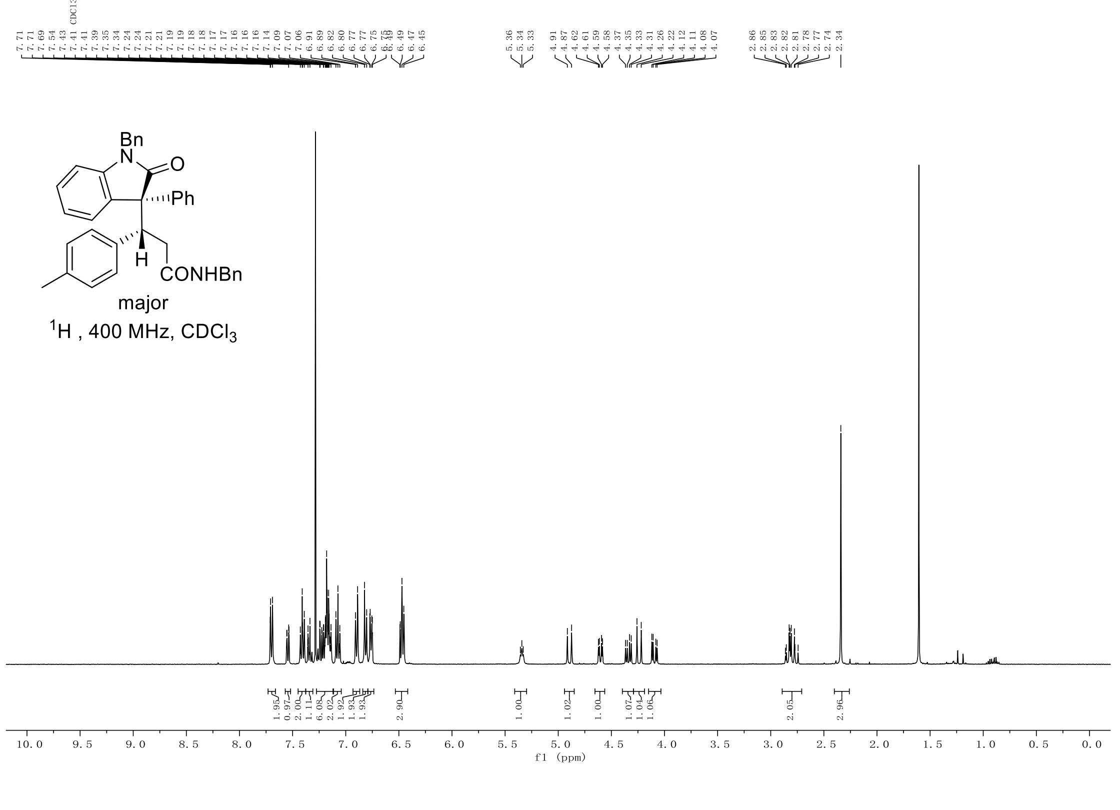

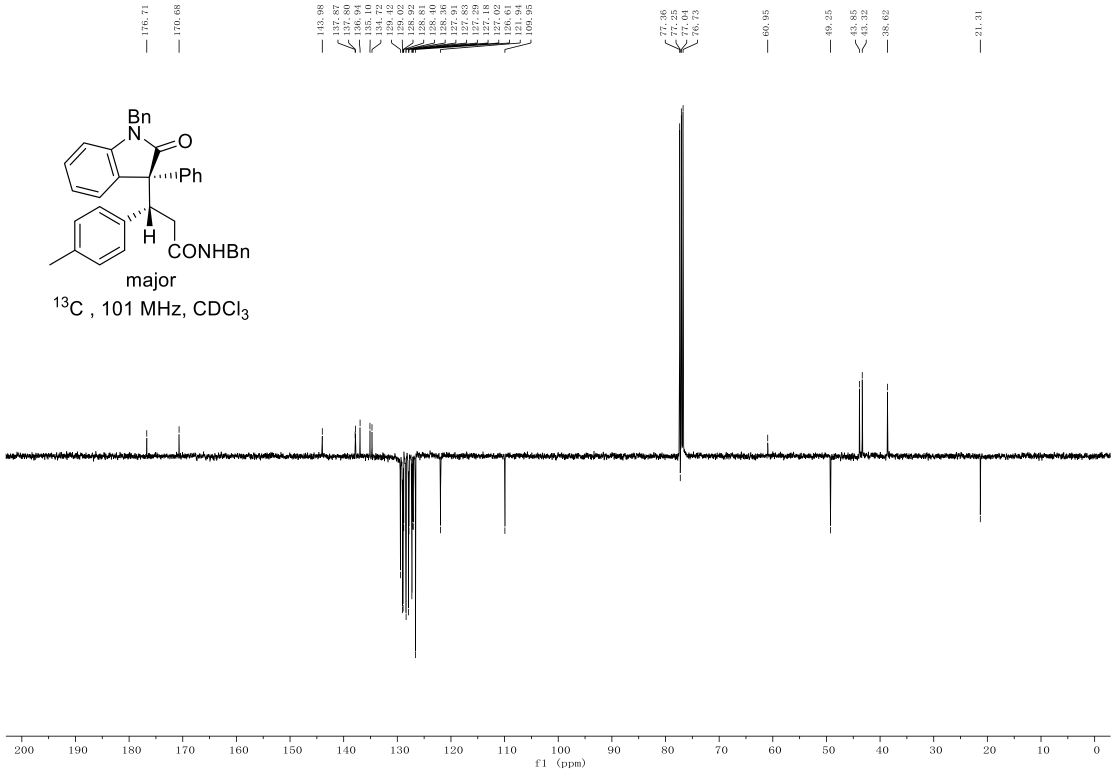

(*S*)-*N*-benzyl-3-((*R*)-1-benzyl-2-oxo-3-phenylindolin-3-yl)-3-(*p*-tolyl)propanamide (S3$_{minor}$)

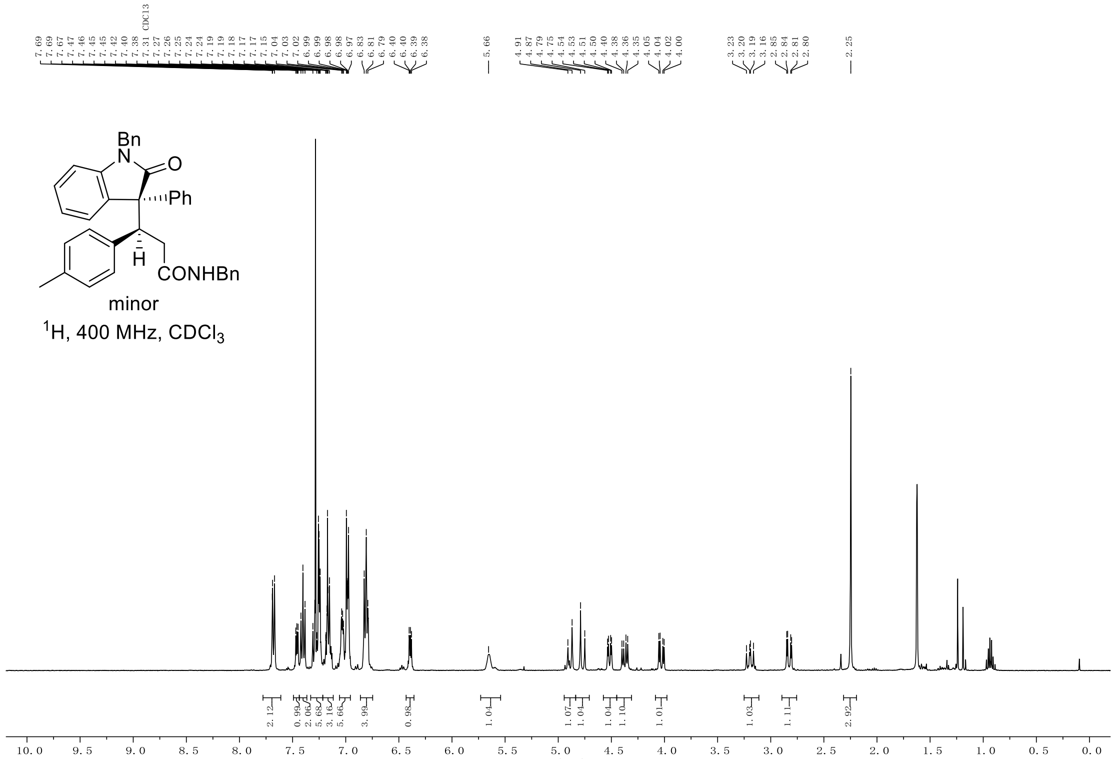

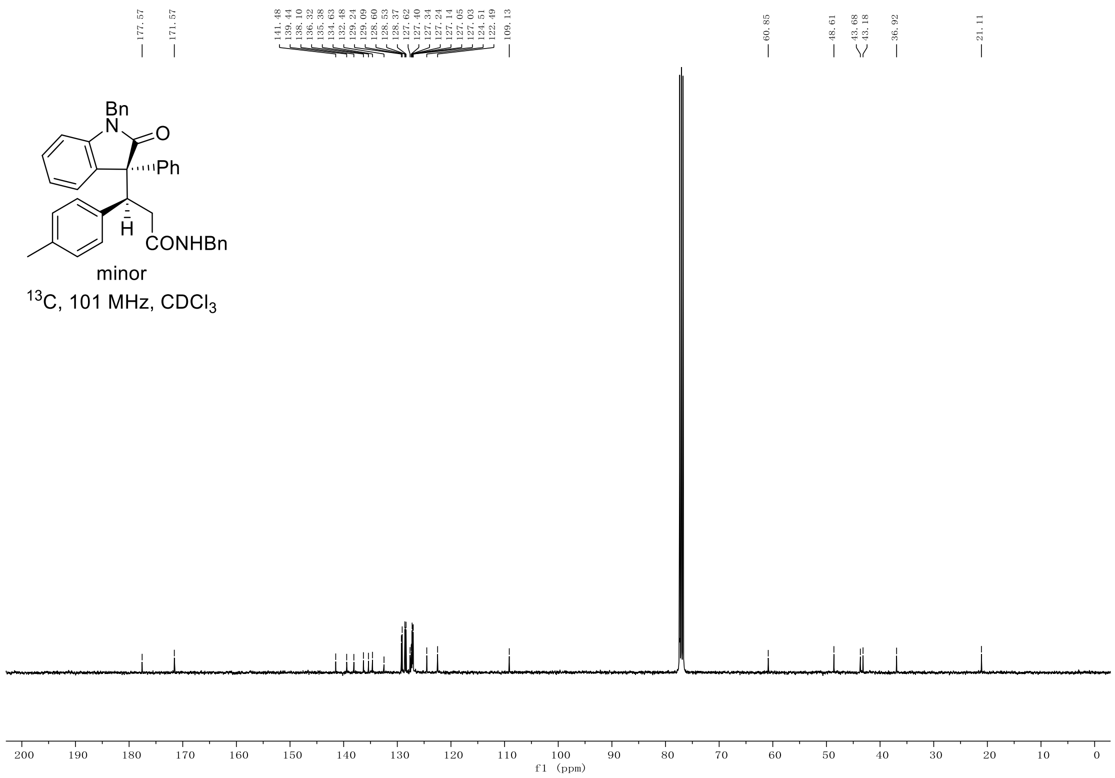

(*R*)-*N*-benzyl-3-((*R*)-1-benzyl-2-oxo-3-phenylindolin-3-yl)-3-(4-nitrophenyl)propanamide ($S4_{major}$)

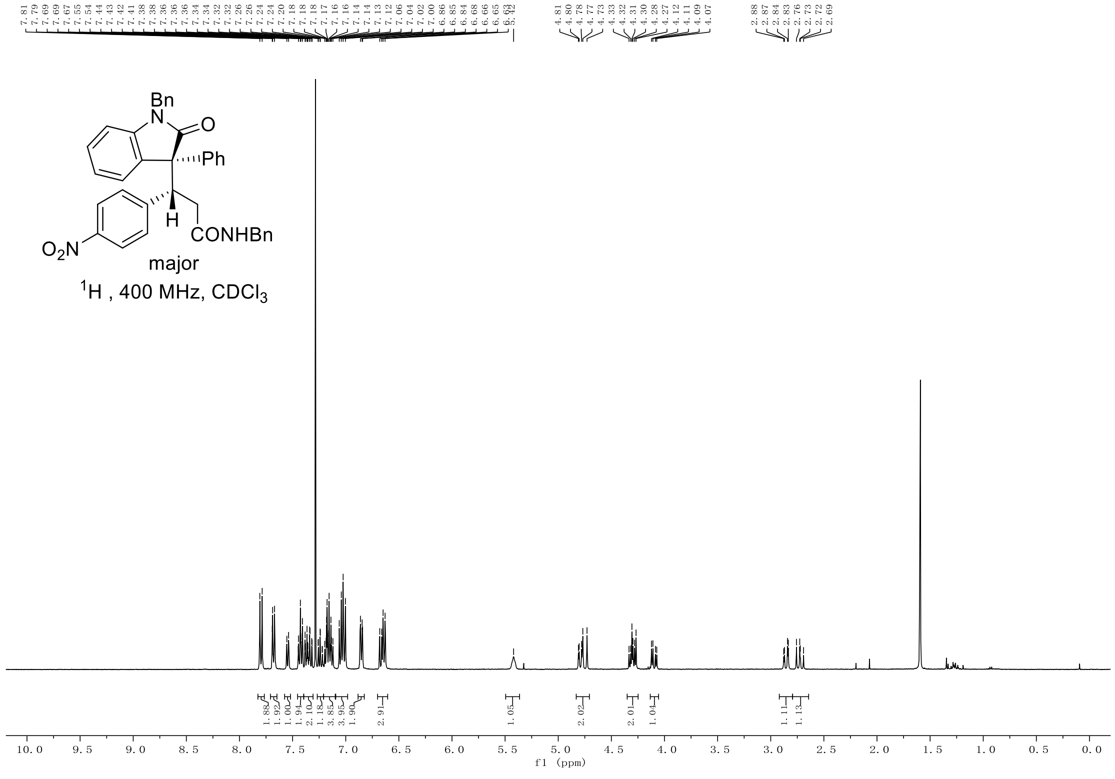

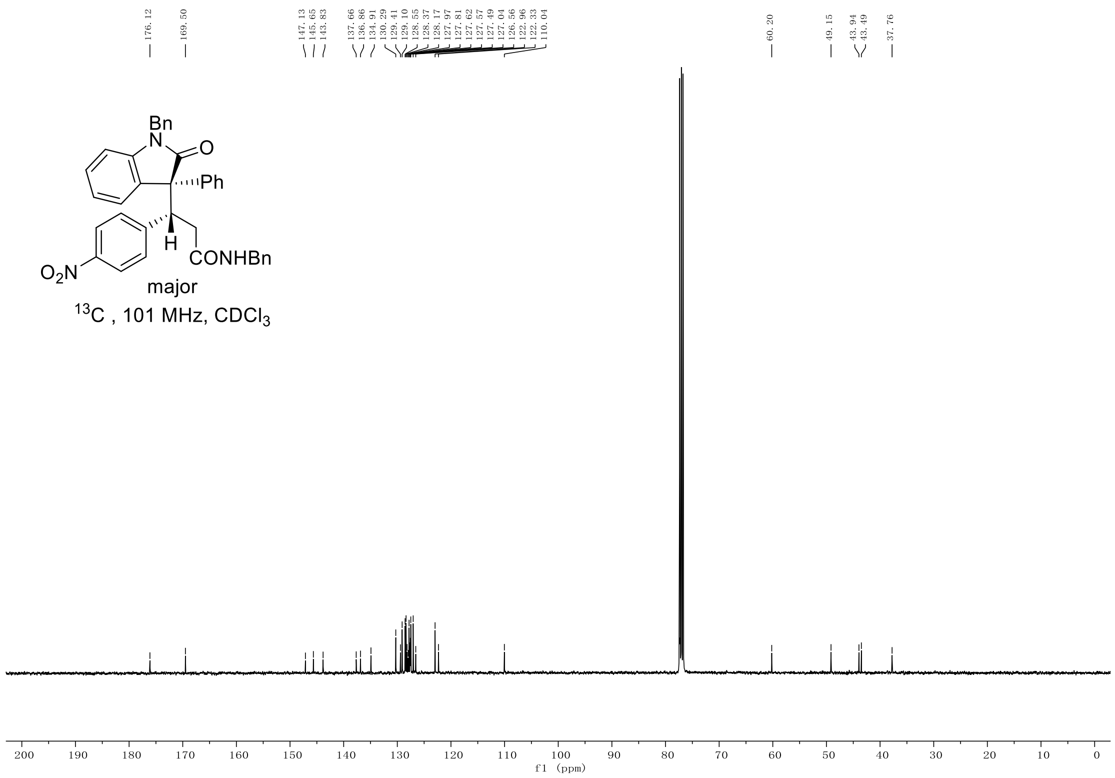

(*S*)-*N*-benzyl-3-((*R*)-1-benzyl-2-oxo-3-phenylindolin-3-yl)-3-(4-nitrophenyl)propanamide (S4$_{minor}$)

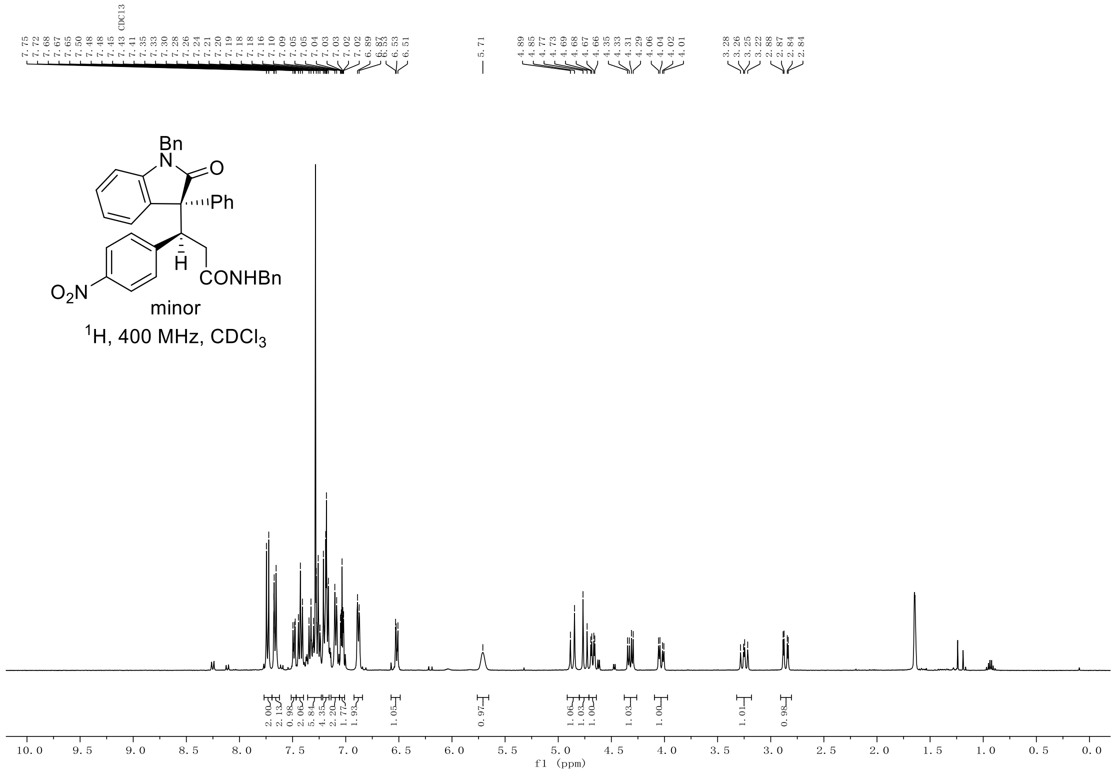

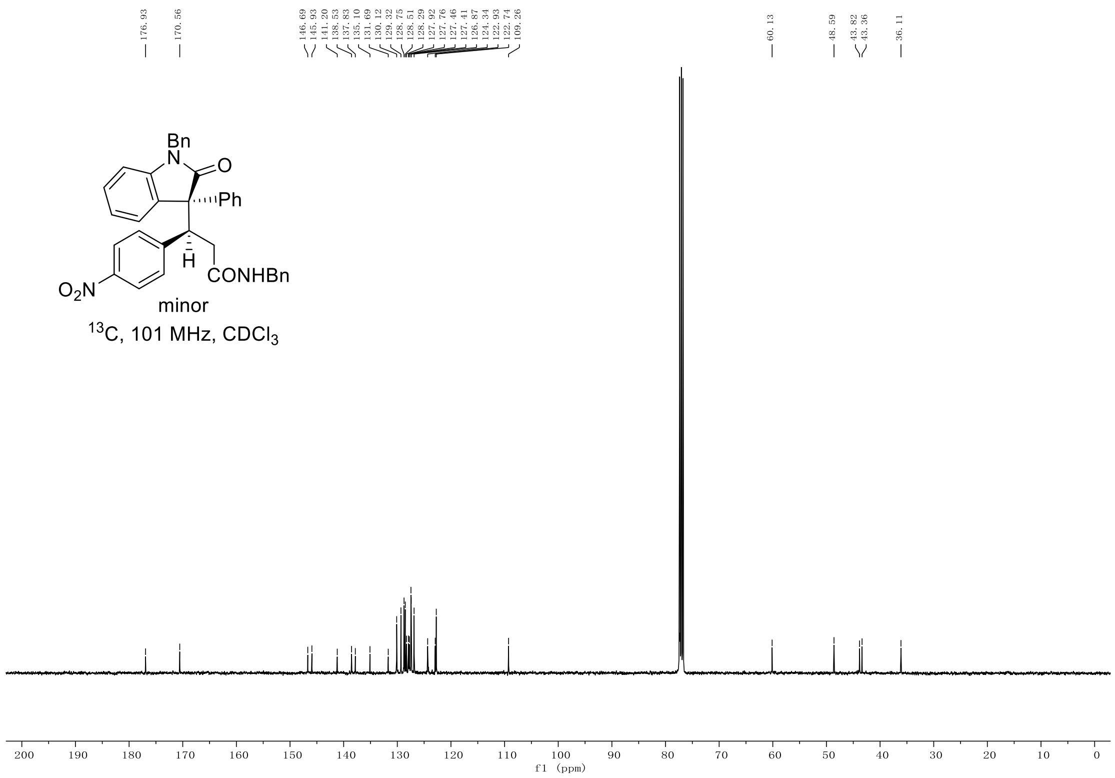

Ethyl (*S*)-2-((*R*)-1-benzyl-2-oxo-3-phenylindolin-3-yl)-4-(benzylamino)-4-oxo Butanoate (81_{major})

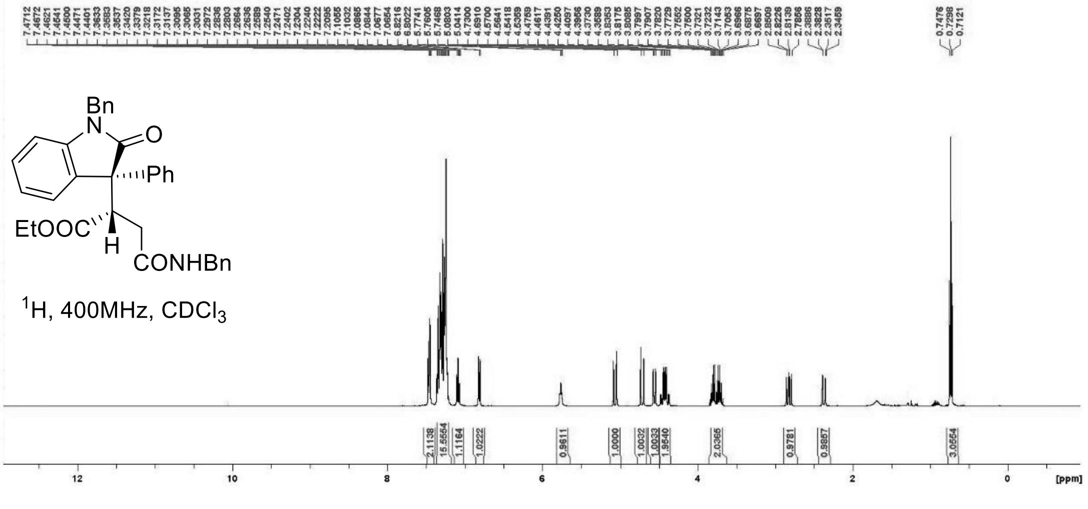

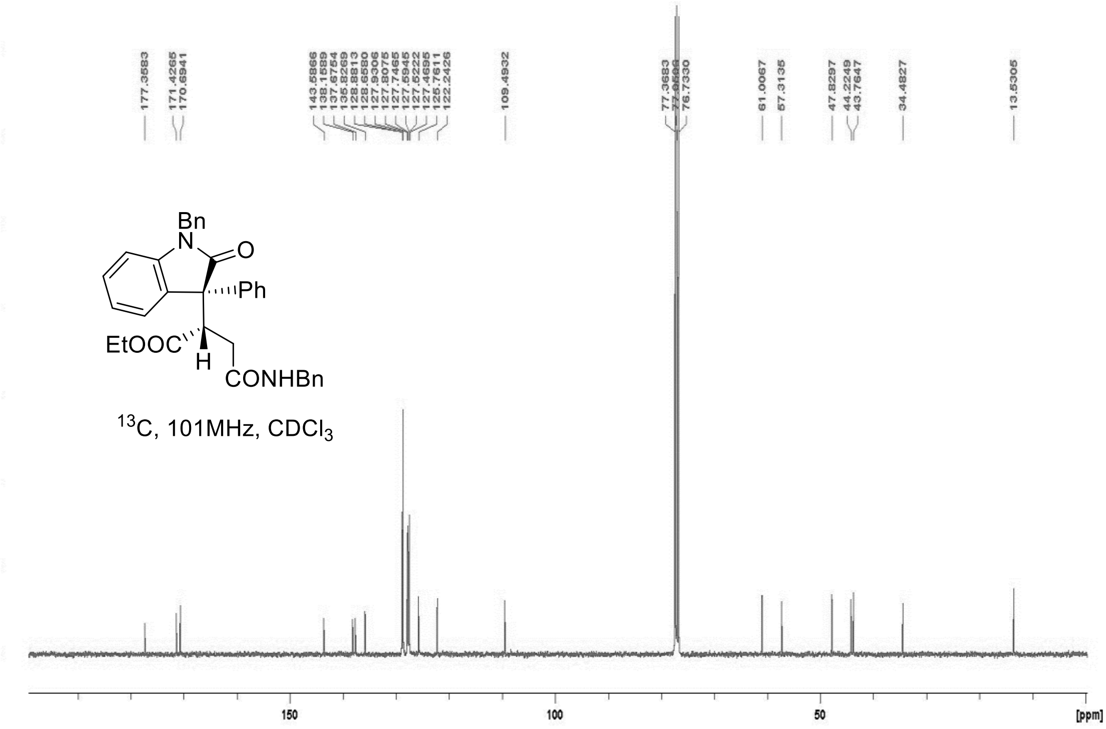

ethyl (*R*)-2-((*R*)-1-benzyl-2-oxo-3-phenylindolin-3-yl)-4-(benzylamino)-4-oxo butanoate (81$_{minor}$)

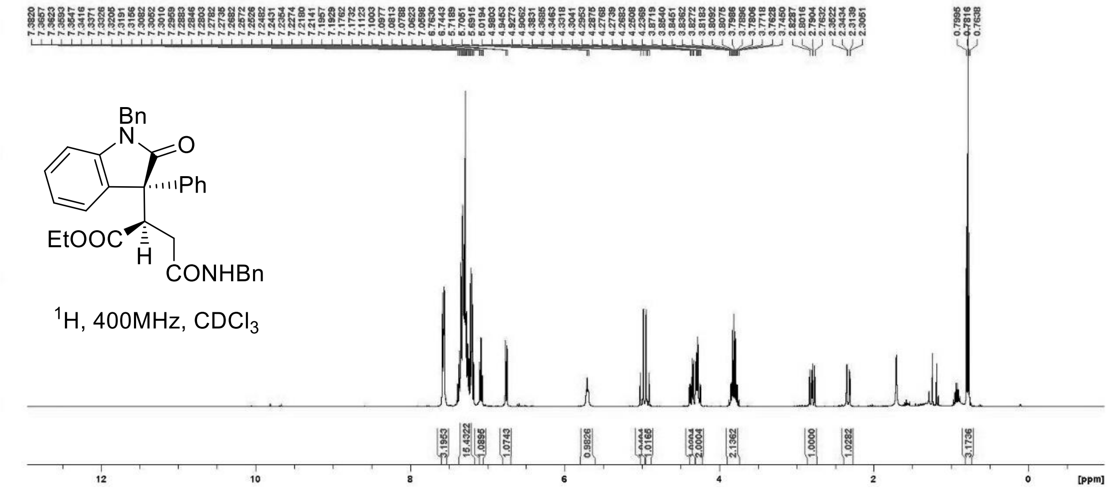

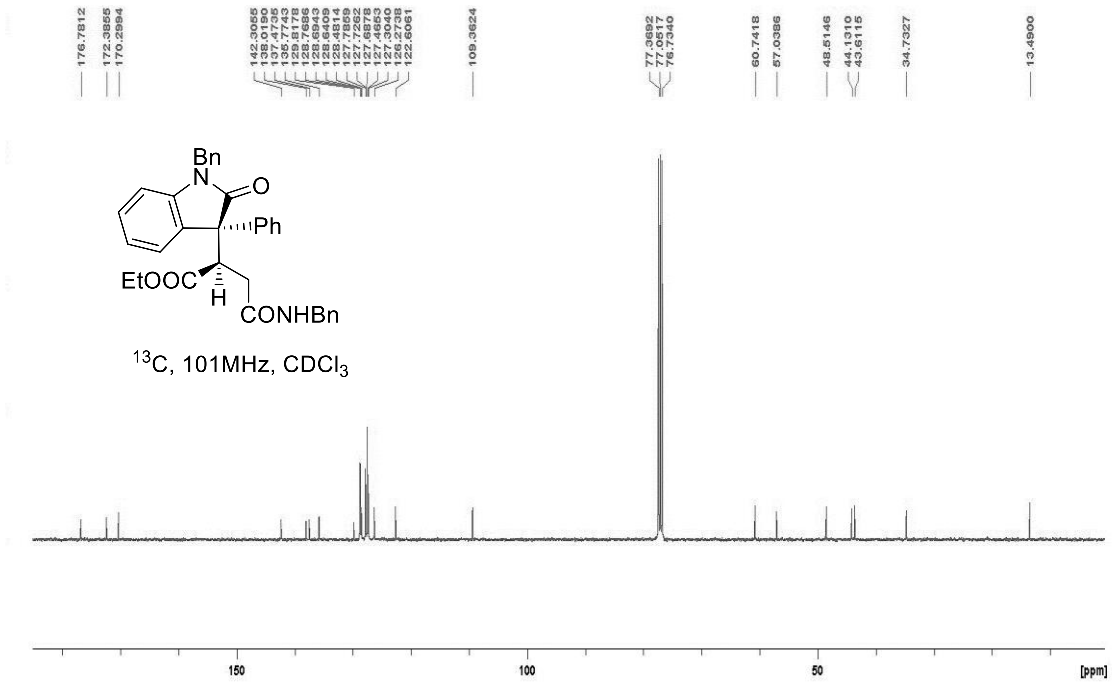
Bn
N
O
Ph
EtOOC
H
CONHBn
13C, 101MHz, CDCl3
176.7812
172.3855
170.2994
142.3055
138.0190
137.4735
135.7743
129.8178
128.7686
128.6943
128.6409
128.4814
127.7859
127.7262
127.6878
127.4653
127.3040
126.2738
122.6061
109.3624
77.3692
77.0517
76.7340
60.7418
57.0386
48.5146
44.1310
43.6115
34.7327
13.4900
150
100
50
[ppm]

Appendix IV: HPLC Spectra

HPLC Data for **12**$_{\mathbf{major}}$: Chiralcel OD-H (95:5 hexane : IPA, flow rate 1.00 mLmin^{-1}, 254 nm, 30 °C) t_R (minor): 31.3 min, t_R (major): 38.3 min, 96:4 er.

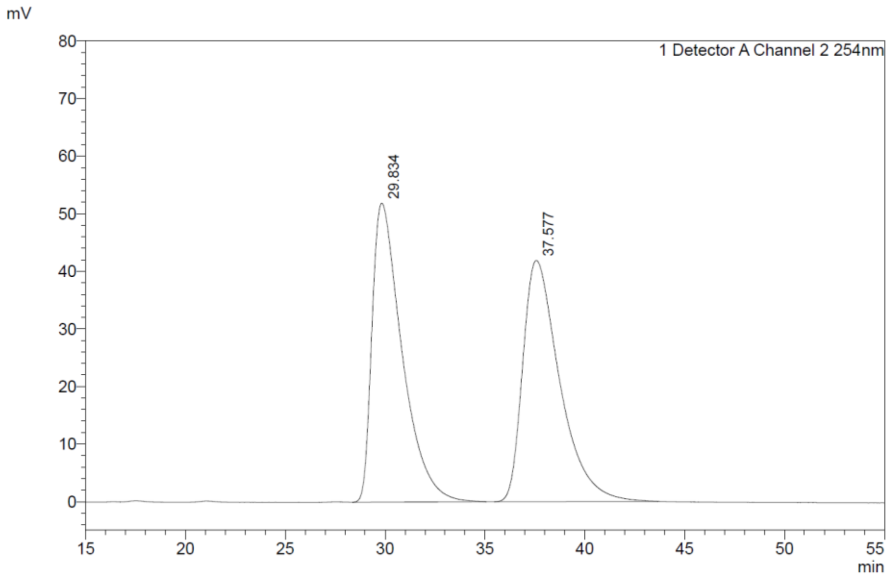

Detector A Channel 2 254nm

Peak#	Ret. Time	Area%
1	29.834	50.152
2	37.577	49.848
Total		100.000

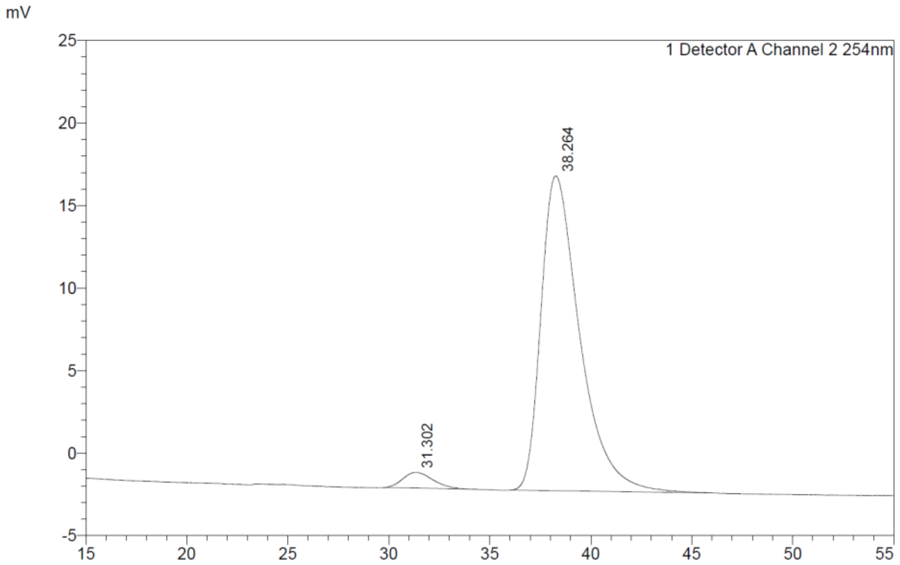

Detector A Channel 2 254nm

Peak#	Ret. Time	Area%
1	31.302	3.703
2	38.264	96.297
Total		100.000

HPLC Data for **12**$_{\mathbf{minor}}$: Chiralpak AD-H (95:5 hexane : IPA, flow rate 1.00 mLmin^{-1}, 254 nm, 30 °C) t_R (minor): 17.1 min, t_R (major): 23.1 min, 96:4 er.

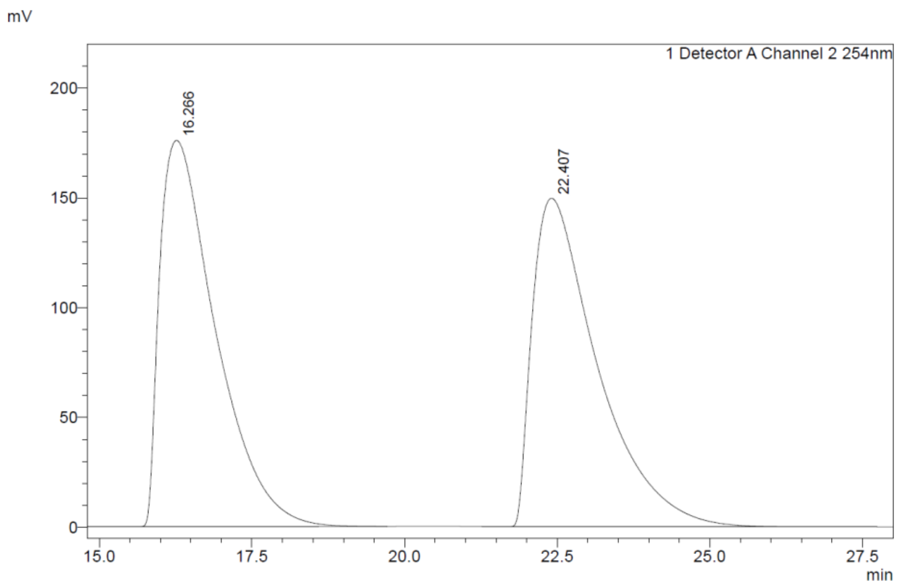

Detector A Channel 2 254nm

Peak#	Ret. Time	Area%
1	16.266	50.188
2	22.407	49.812
Total		100.000

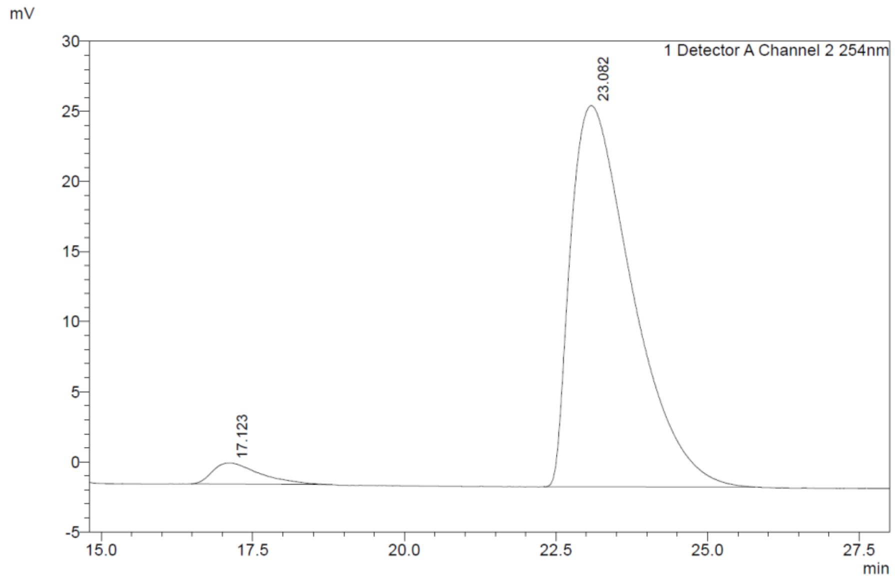

Detector A Channel 2 254nm

Peak#	Ret. Time	Area%
1	17.123	4.113
2	23.082	95.887
Total		100.000

HPLC Data for **25_{major}**: Chiralcel OJ-H (93:7 hexane : IPA, flow rate 1.00 mLmin^{-1}, 254 nm, 30 °C) t_R (minor): 15.8 min, t_R (major): 19.5 min, 92:8 er.

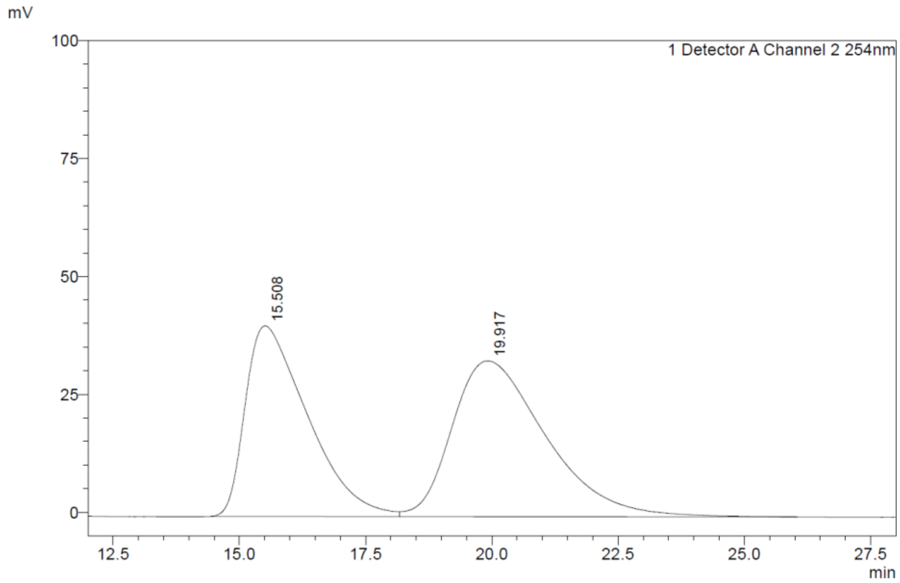

Detector A Channel 2 254nm

Peak#	Ret. Time	Area%
1	15.508	49.283
2	19.917	50.717
Total		100.000

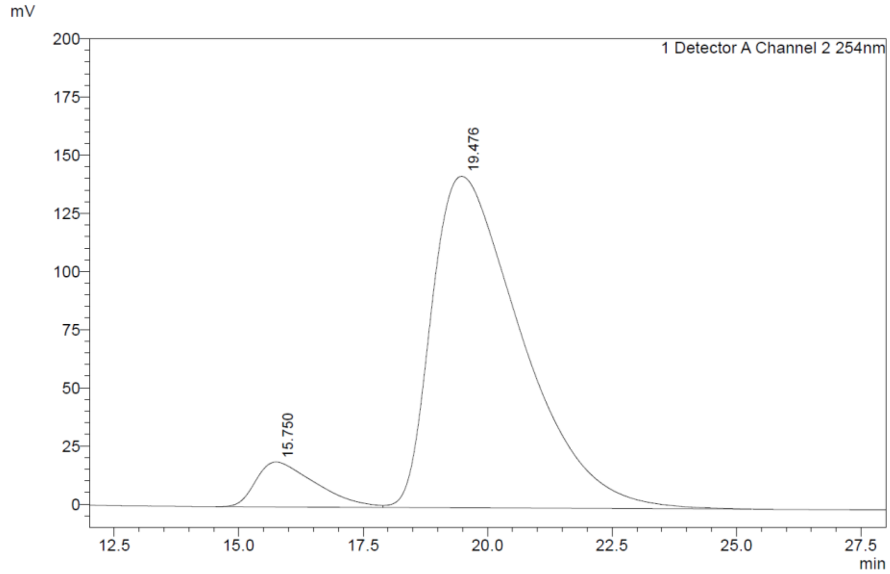

Detector A Channel 2 254nm

Peak#	Ret. Time	Area%
1	15.750	8.217
2	19.476	91.783
Total		100.000

HPLC Data for **25$_{minor}$**: Chiralpak AD-H (97:3 hexane : IPA, flow rate 1.00 mLmin^{-1}, 254 nm, 30 °C) t_R (minor): 22.6 min, t_R (major): 29.5 min, 96:4 er.

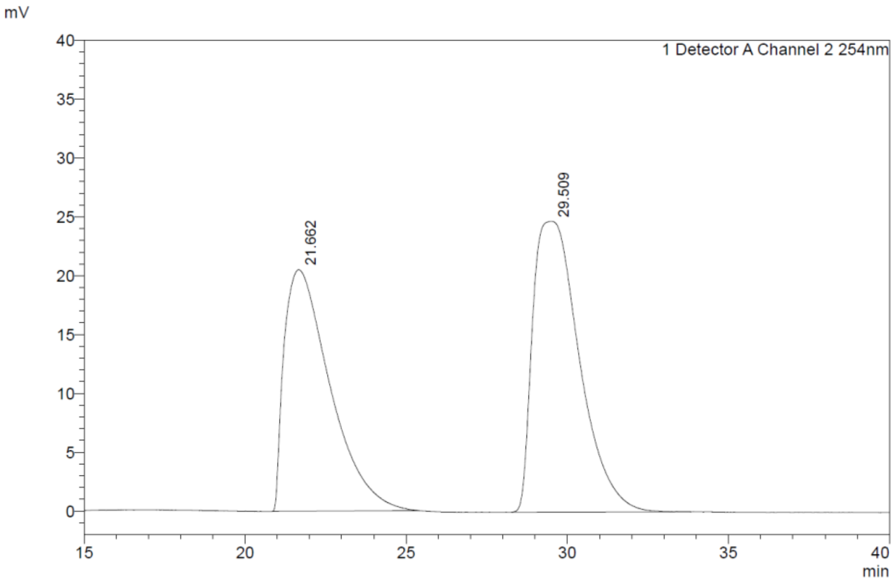

Detector A Channel 2 254nm

Peak#	Ret. Time	Area%
1	21.662	49.733
2	29.509	50.267
Total		100.000

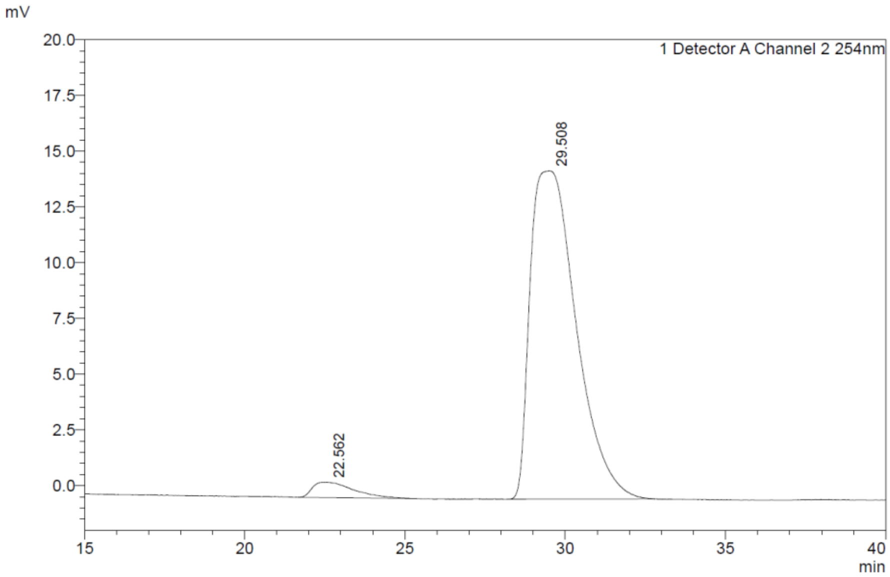

Detector A Channel 2 254nm

Peak#	Ret. Time	Area%
1	22.562	3.991
2	29.508	96.009
Total		100.000

HPLC Data for **26$_{major}$**: Chiralpak AD-H (90:10 hexane : IPA, flow rate 1.00 mLmin^{-1}, 254 nm, 30 °C) t_R (major): 16.3 min, t_R (minor): 41.8 min, 97:3 er.

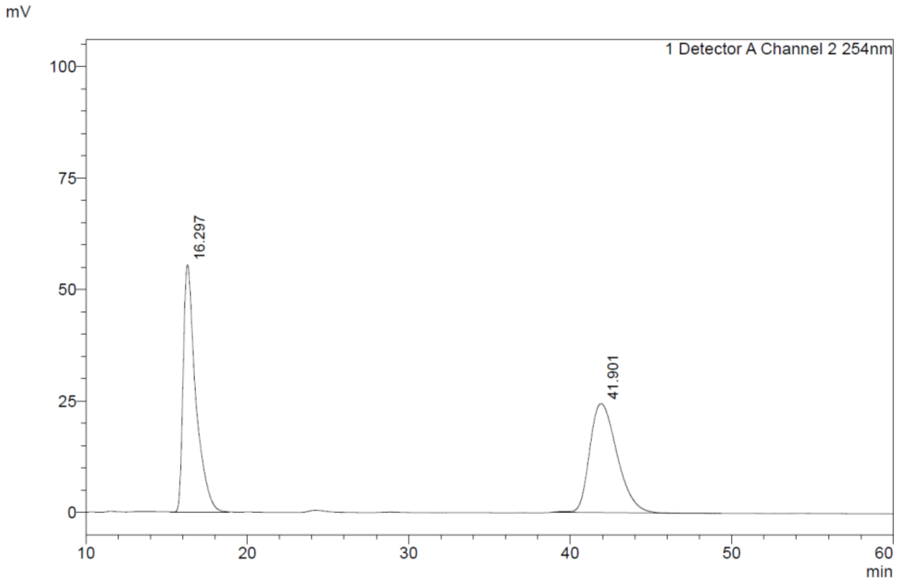

Detector A Channel 2 254nm

Peak#	Ret. Time	Area%
1	16.297	50.930
2	41.901	49.070
Total		100.000

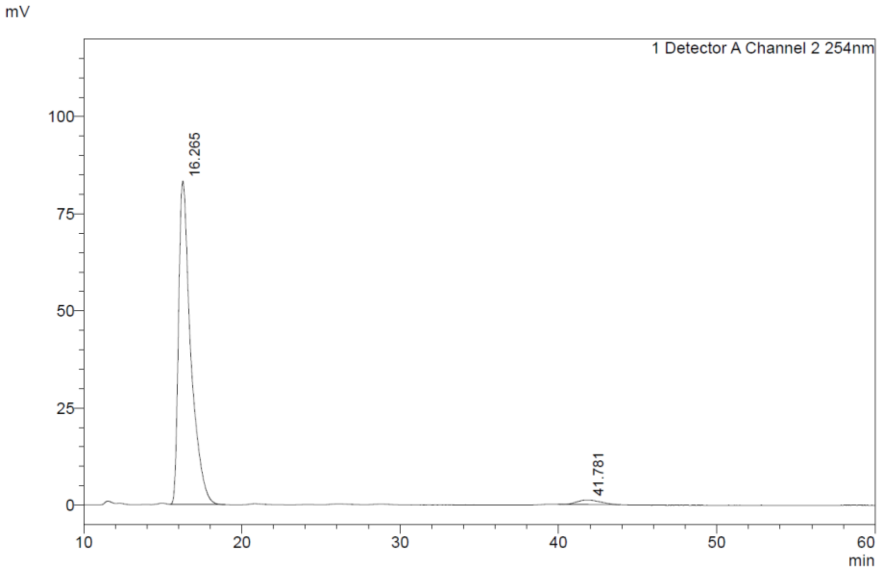

Detector A Channel 2 254nm

Peak#	Ret. Time	Area%
1	16.265	97.396
2	41.781	2.604
Total		100.000

HPLC Data for **26**$_{\mathbf{minor}}$: Chiralpak AD-H (93:7 hexane : IPA, flow rate 1.00 mLmin^{-1}, 254 nm, 30 °C) t_R (minor): 19.0 min, t_R (major): 39.7 min, 97:3 er.

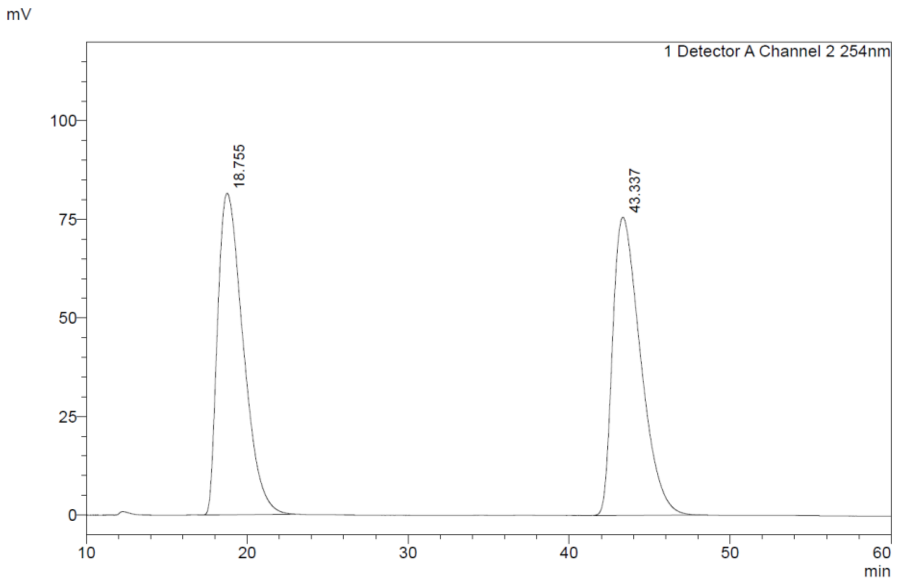

Detector A Channel 2 254nm

Peak#	Ret. Time	Area%
1	18.755	49.594
2	43.337	50.406
Total		100.000

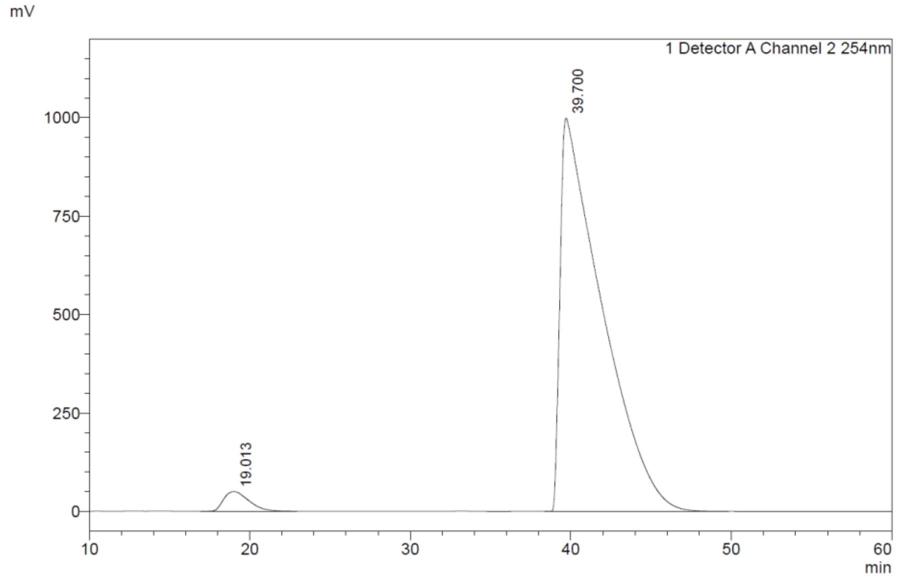

Detector A Channel 2 254nm

Peak#	Ret. Time	Area%
1	19.013	2.913
2	39.700	97.087
Total		100.000

HPLC Data for **27**$_{\mathbf{major}}$: Chiralpak AD-H (90:10 hexane : IPA, flow rate 1.00 mLmin^{-1}, 254 nm, 30 °C) t_R (major): 15.0 min, t_R (minor): 41.9 min, 96:4 er.

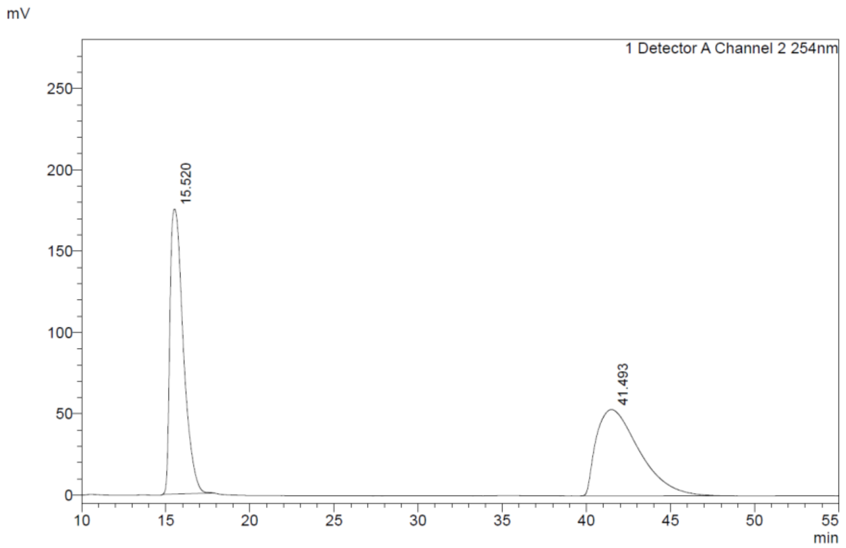

Detector A Channel 2 254nm

Peak#	Ret. Time	Area%
1	15.520	50.688
2	41.493	49.312
Total		100.000

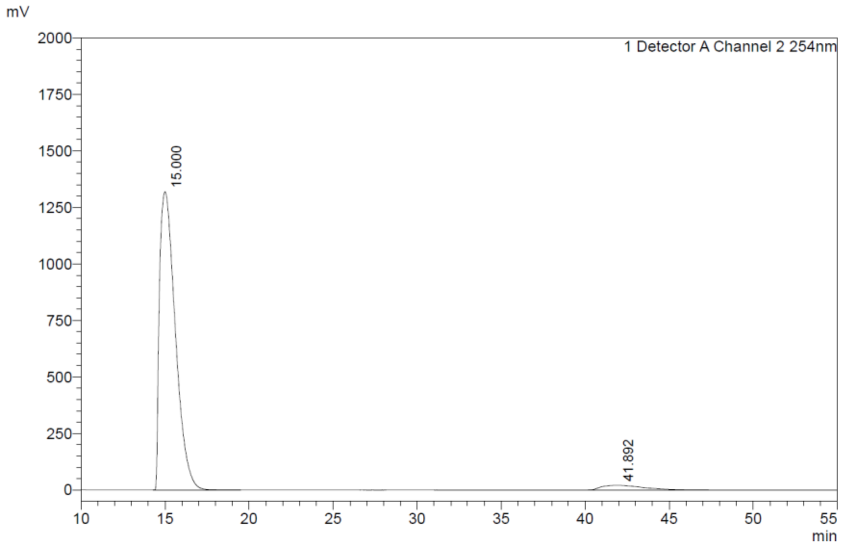

Detector A Channel 2 254nm

Peak#	Ret. Time	Area%
1	15.000	96.056
2	41.892	3.944
Total		100.000

HPLC Data for **27**$_{\mathbf{minor}}$: Chiralpak AD-H (97:3 hexane : IPA, flow rate 1.00 mLmin^{-1}, 254 nm, 30 °C) t_R (major): 30.9 min, t_R (minor): 36.4 min, 98:2 er.

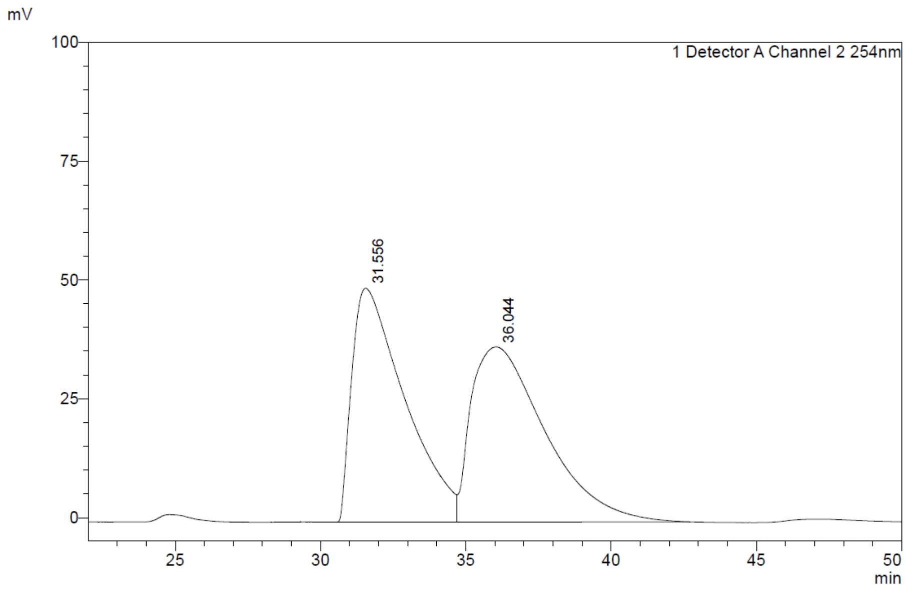

Detector A Channel 2 254nm

Peak#	Ret. Time	Area%
1	31.556	49.939
2	36.044	50.061
Total		100.000

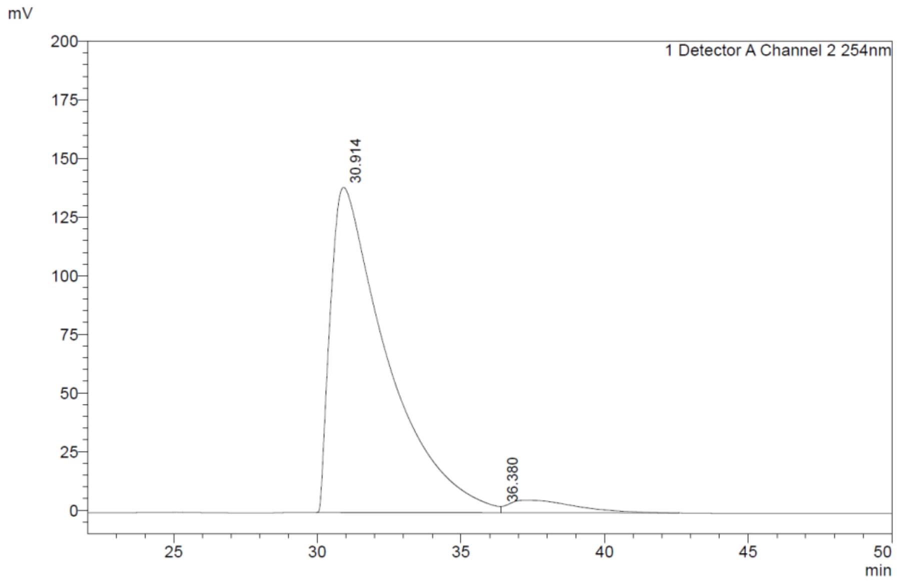

Detector A Channel 2 254nm

Peak#	Ret. Time	Area%
1	30.914	97.586
2	36.380	2.414
Total		100.000

HPLC Data for **28$_{major}$**: Chiralpak AD-H (90:10 hexane : IPA, flow rate 1.00 mLmin^{-1}, 254 nm, 30 °C) t_R (major): 16.1 min, t_R (minor): 21.9 min, 96:4 er.

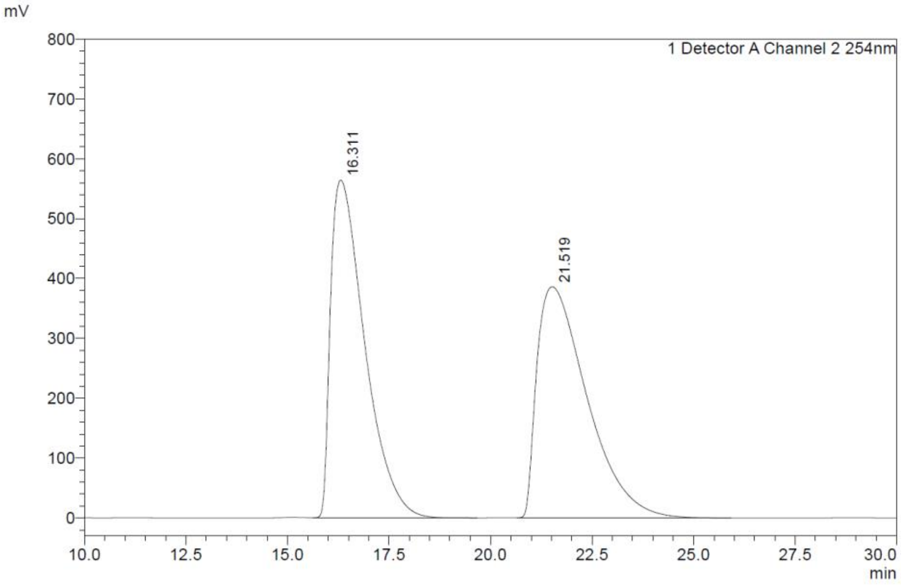

Detector A Channel 2 254nm

Peak#	Ret. Time	Area%
1	16.311	49.782
2	21.519	50.218
Total		100.000

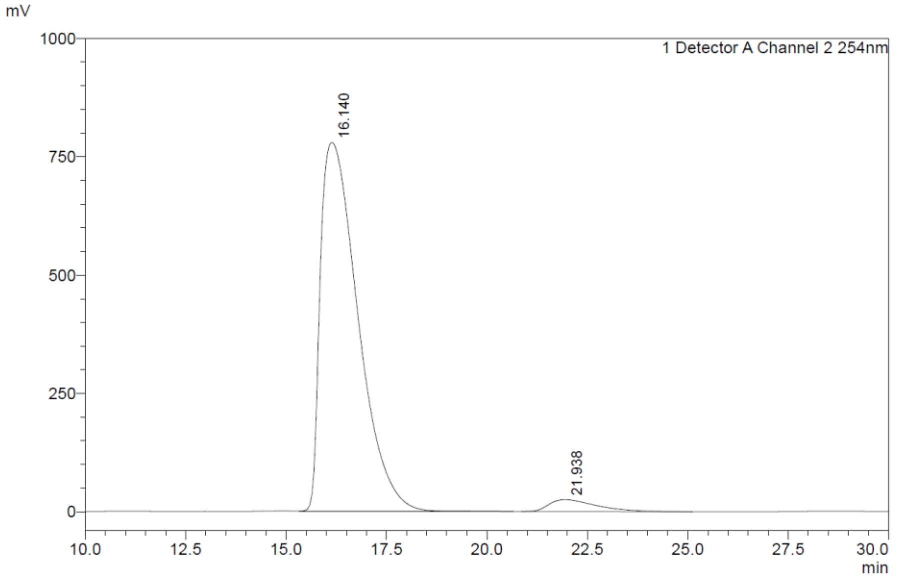

Detector A Channel 2 254nm

Peak#	Ret. Time	Area%
1	16.140	96.102
2	21.938	3.898
Total		100.000

HPLC Data for **28**$_{\mathbf{minor}}$: Chiralcel OD-H (97:3 hexane : IPA, flow rate 1.00 mLmin^{-1}, 254 nm, 30 °C) t_R (major): 15.2 min, t_R (minor): 24.7 min, 96:4 er.

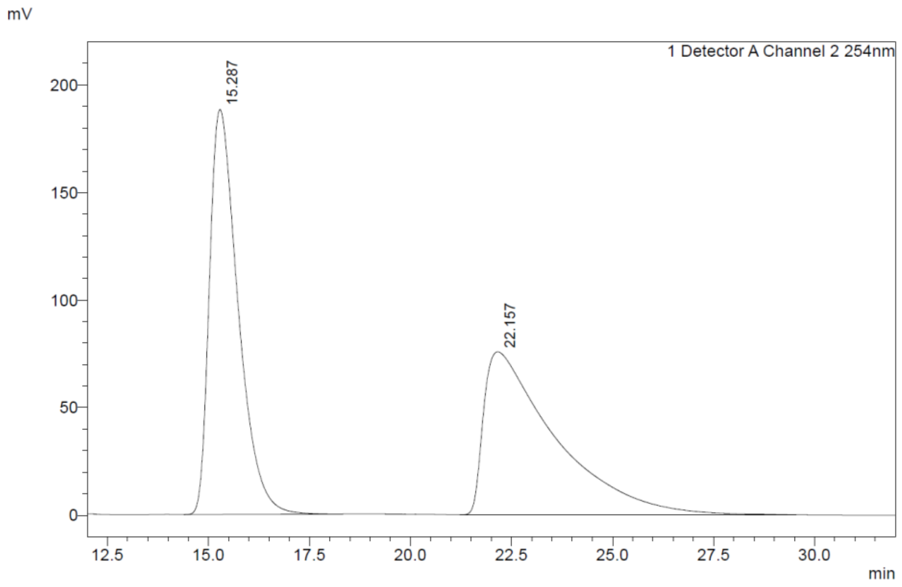

Detector A Channel 2 254nm

Peak#	Ret. Time	Area%
1	15.287	49.857
2	22.157	50.143
Total		100.000

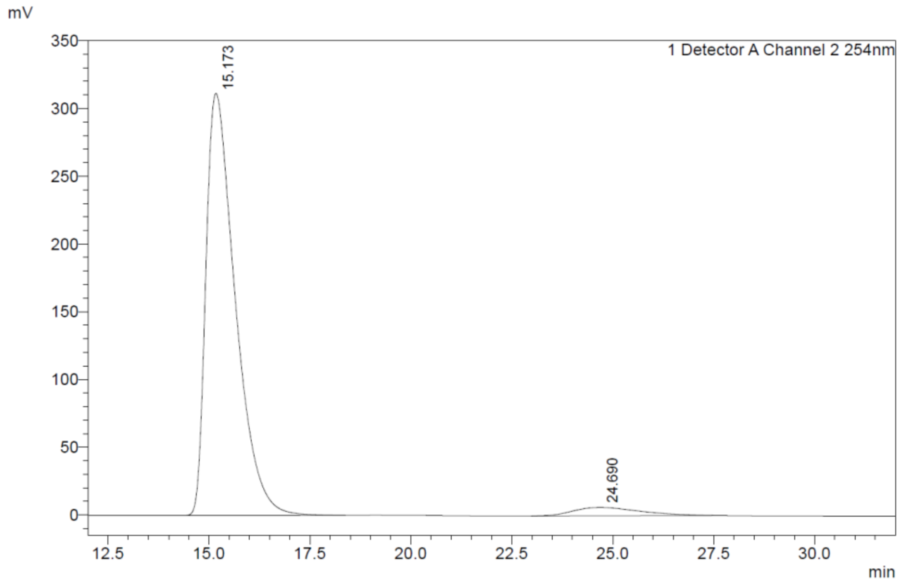

Detector A Channel 2 254nm

Peak#	Ret. Time	Area%
1	15.173	95.685
2	24.690	4.315
Total		100.000

HPLC Data for **29$_{major}$**: Chiralpak AD-H (85:15 hexane : IPA, flow rate 1.00 mLmin^{-1}, 254 nm, 30 °C) t_R (major): 11.7 min, t_R (minor): 21.2 min, 99:1 er.

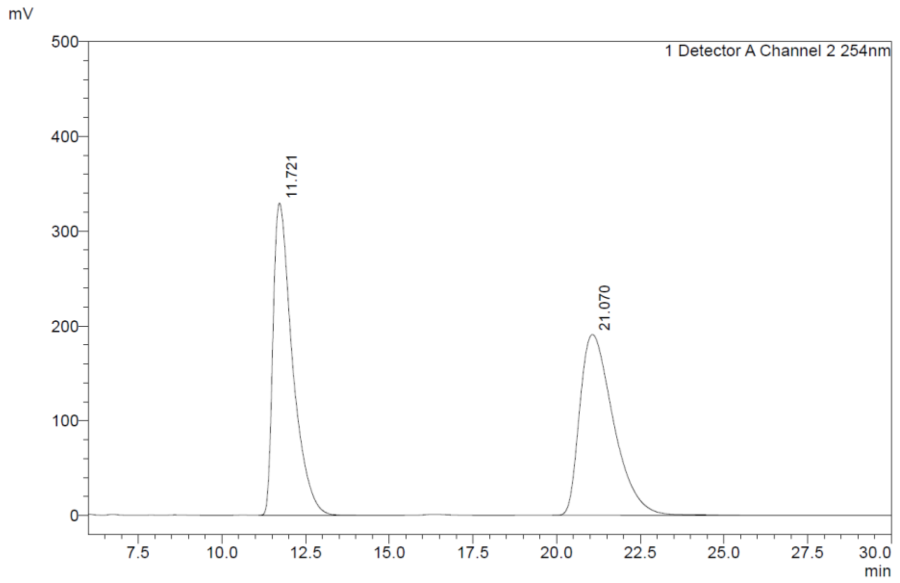

Detector A Channel 2 254nm

Peak#	Ret. Time	Area%
1	11.721	49.842
2	21.070	50.158
Total		100.000

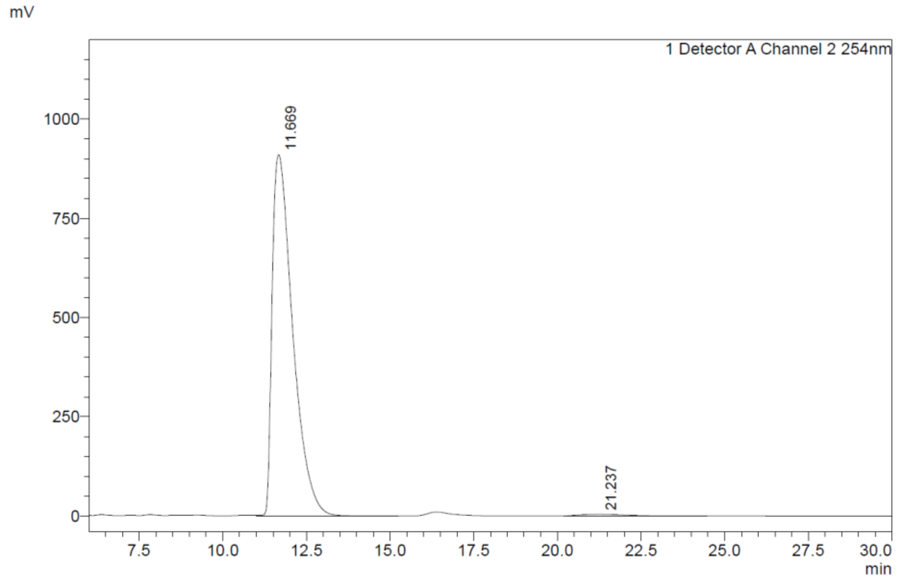

Detector A Channel 2 254nm

Peak#	Ret. Time	Area%
1	11.669	99.147
2	21.237	0.853
Total		100.000

HPLC Data for **29**$_{\mathbf{minor}}$: Chiralpak AD-H (90:10 hexane : IPA, flow rate 1.00 mLmin^{-1}, 254 nm, 30 °C) t_R (minor): 23.4 min, t_R (major): 28.4 min, 95:5 er.

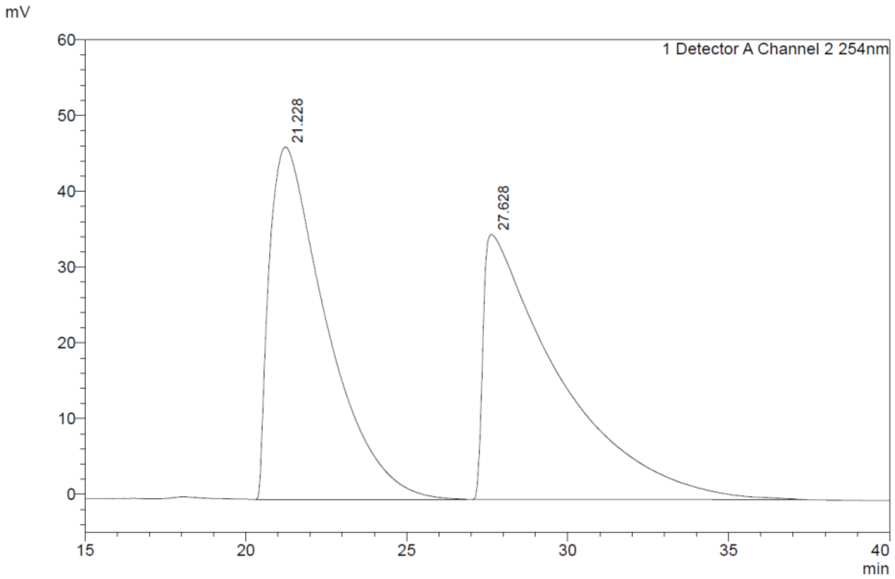

Detector A Channel 2 254nm

Peak#	Ret. Time	Area%
1	21.228	50.116
2	27.628	49.884
Total		100.000

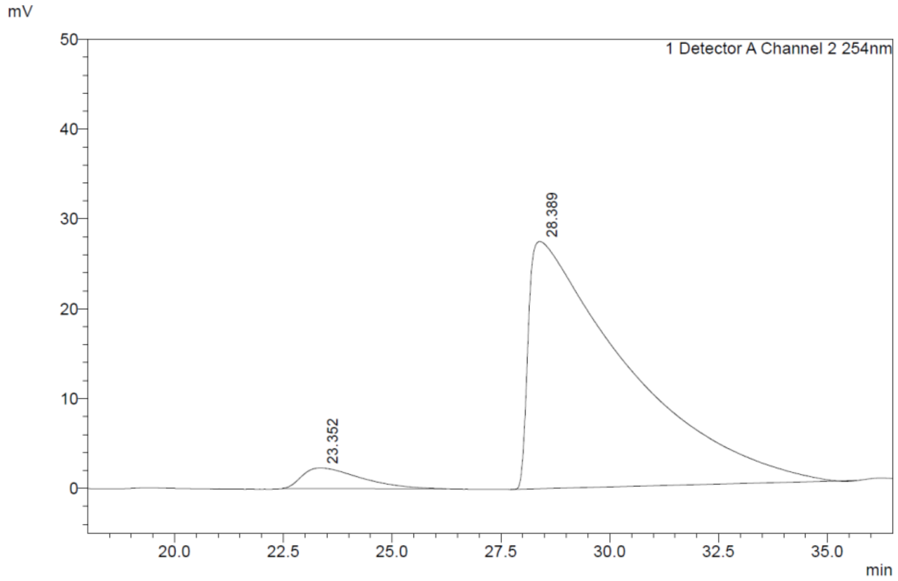

Detector A Channel 2 254nm

Peak#	Ret. Time	Area%
1	23.352	4.717
2	28.389	95.283
Total		100.000

HPLC Data for **30$_{major}$**: Chiralpak AD-H (85:15 hexane : IPA, flow rate 1.00 mLmin^{-1}, 254 nm, 30 °C) t_R (major): 10.3 min, t_R (minor): 24.3 min, 97:3 er.

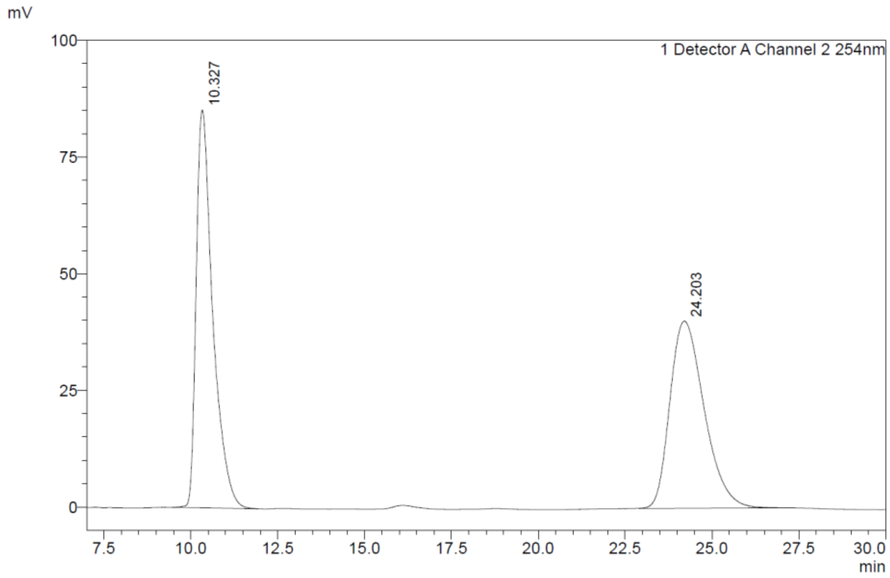

Detector A Channel 2 254nm

Peak#	Ret. Time	Area%
1	10.327	50.613
2	24.203	49.387
Total		100.000

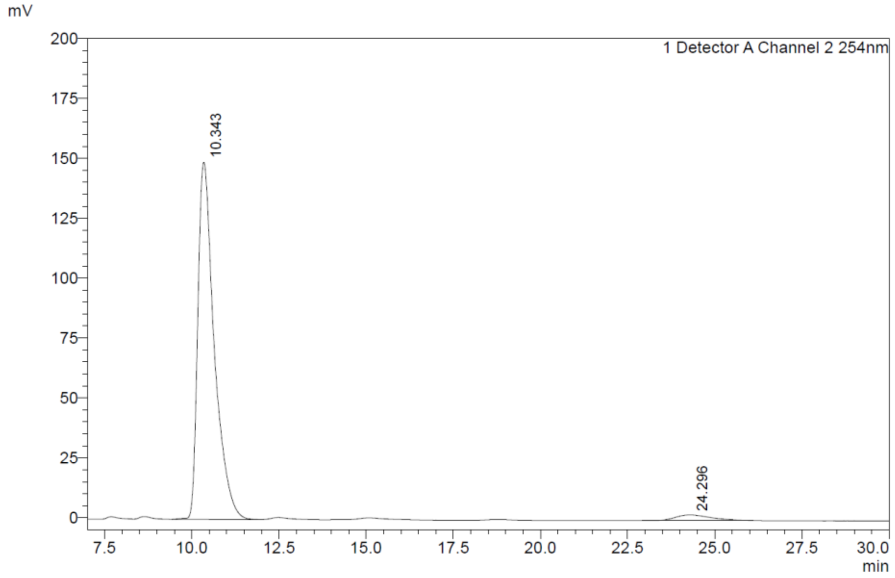

Detector A Channel 2 254nm

Peak#	Ret. Time	Area%
1	10.343	96.715
2	24.296	3.285
Total		100.000

HPLC Data for **30**$_{\textbf{minor}}$: Chiralcel OD-H (97:3 hexane : IPA, flow rate 1.00 mLmin^{-1}, 254 nm, 30 °C) t_R (major): 16.2 min, t_R (minor): 26.1 min, 97:3 er.

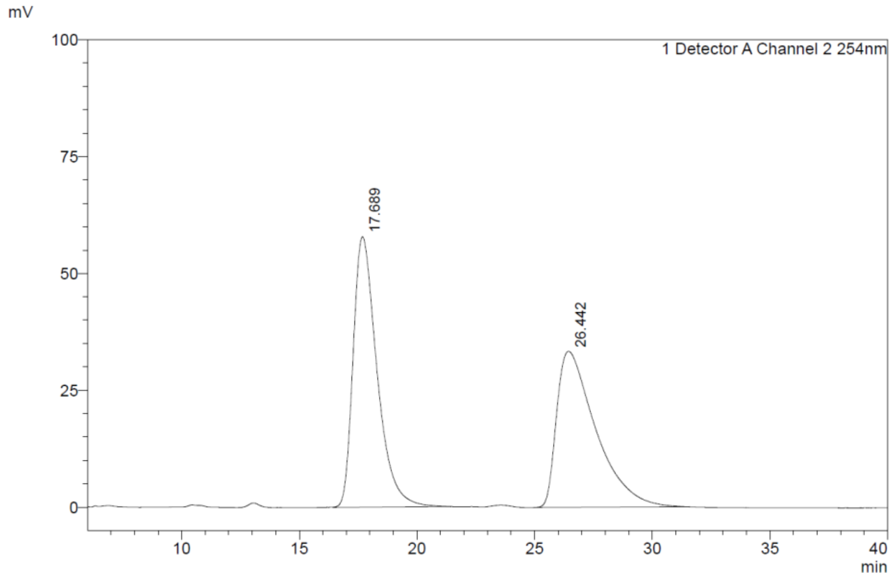

Detector A Channel 2 254nm

Peak#	Ret. Time	Area%
1	17.689	50.905
2	26.442	49.095
Total		100.000

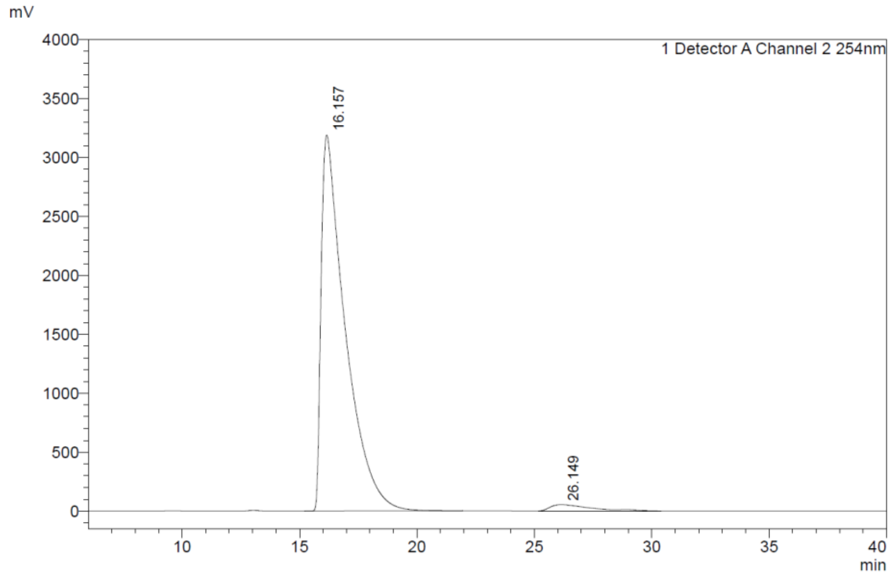

Detector A Channel 2 254nm

Peak#	Ret. Time	Area%
1	16.157	97.236
2	26.149	2.764
Total		100.000

HPLC Data for **31**$_{\mathbf{major}}$: Chiralcel AD-H (90:10 hexane : IPA, flow rate 1.00 mLmin^{-1}, 254 nm, 30 °C) t_R (major): 11.2 min, t_R (minor): 22.8 min, 99:1 er.

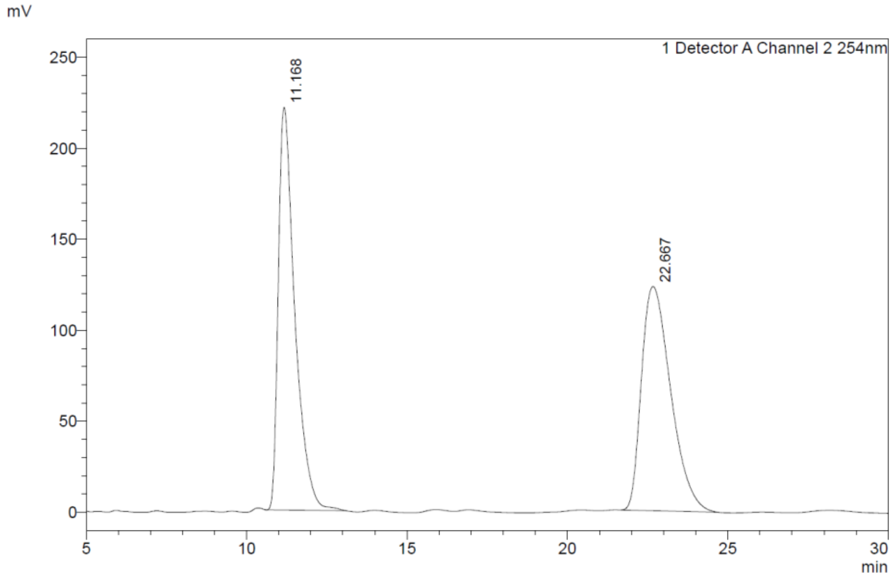

Detector A Channel 2 254nm

Peak#	Ret. Time	Area%
1	11.168	50.326
2	22.667	49.674
Total		100.000

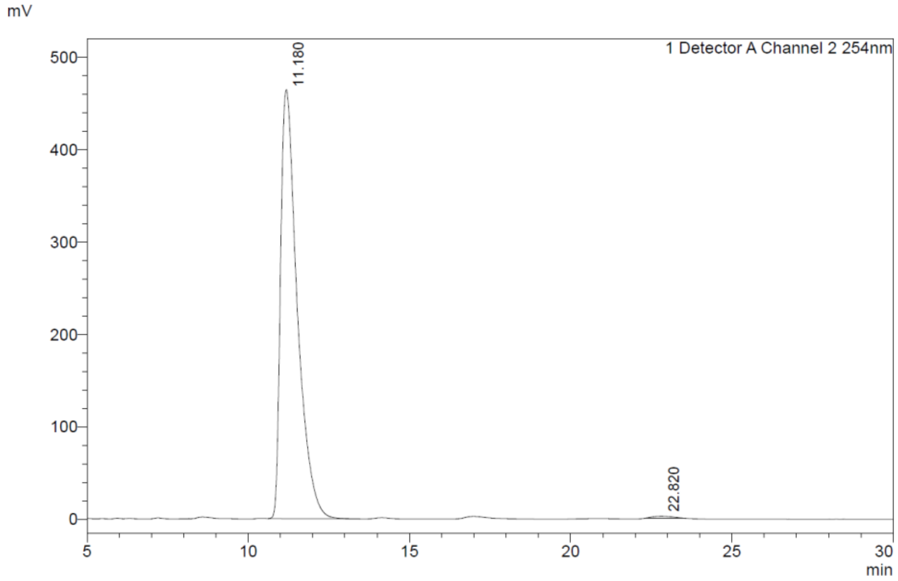

Detector A Channel 2 254nm

Peak#	Ret. Time	Area%
1	11.180	99.375
2	22.820	0.625
Total		100.000

HPLC Data for **31**$_{\mathbf{minor}}$: Chiralcel OD-H (95:5 hexane : IPA, flow rate 1.00 mLmin^{-1}, 254 nm, 30 °C) t_R (major): 11.8 min, t_R (minor): 20.8 min, 96:4 ee.

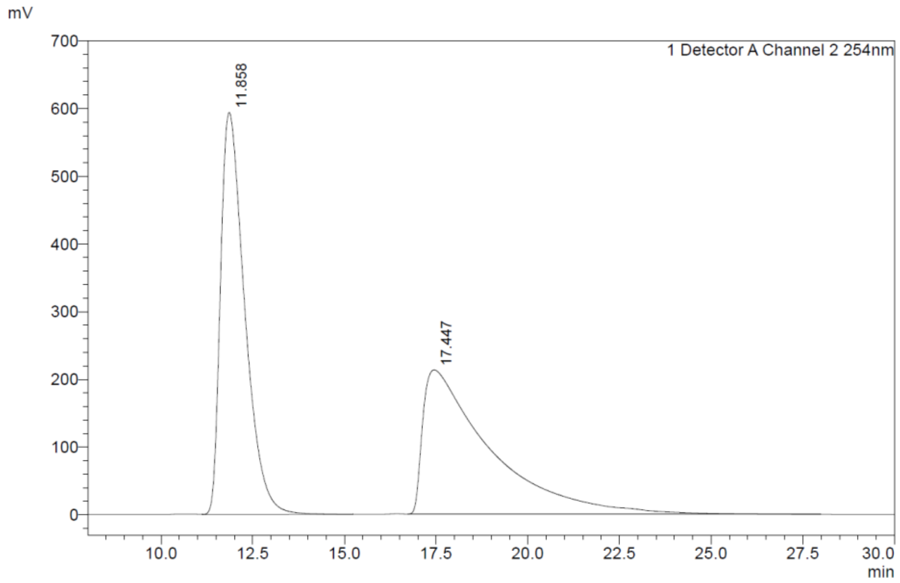

Detector A Channel 2 254nm

Peak#	Ret. Time	Area%
1	11.858	49.758
2	17.447	50.242
Total		100.000

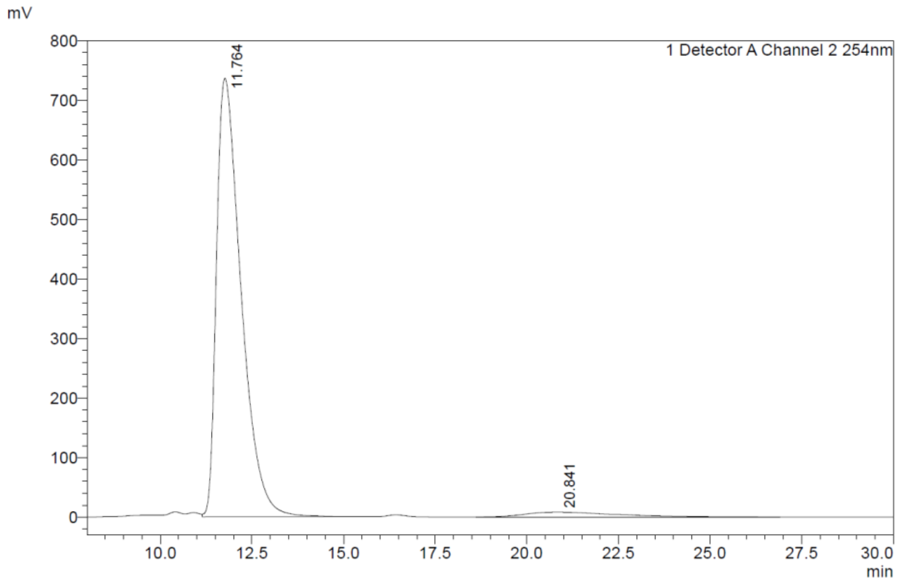

Detector A Channel 2 254nm

Peak#	Ret. Time	Area%
1	11.764	96.107
2	20.841	3.893
Total		100.000

HPLC Data for **32$_{major}$**: Chiralpak AD-H (90:10 hexane : IPA, flow rate 1.00 mLmin^{-1}, 254 nm, 30 °C) t_R (major): 15.8 min, t_R (minor): 21.4 min, 95:5 er.

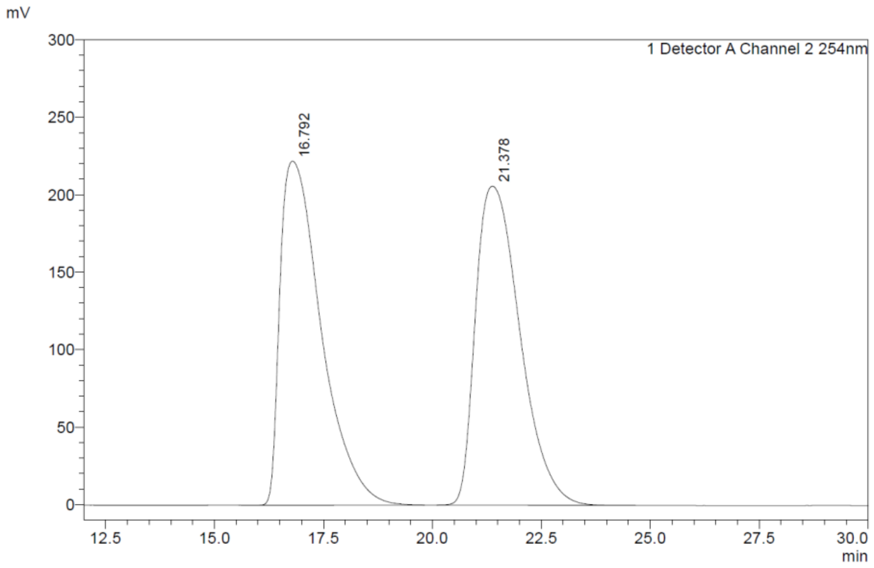

Detector A Channel 2 254nm

Peak#	Ret. Time	Area%
1	16.792	50.017
2	21.378	49.983
Total		100.000

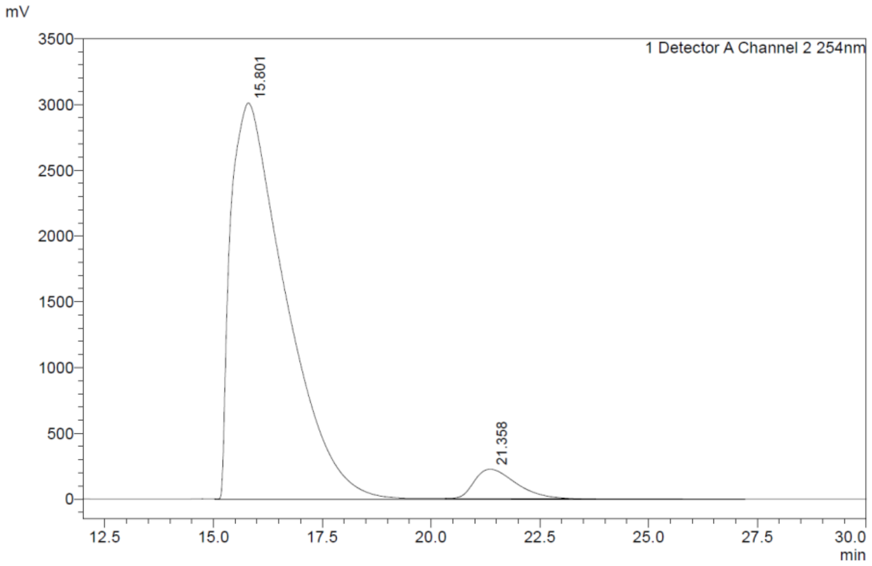

Detector A Channel 2 254nm

Peak#	Ret. Time	Area%
1	15.801	94.679
2	21.358	5.321
Total		100.000

HPLC Data for **32$_{minor}$**: Chiralpak AD-H (93:7 hexane : IPA, flow rate 1.00 mLmin^{-1}, 254 nm, 30 °C) t_R (minor): 22.3 min, t_R (major): 26.1 min, 96:4 er.

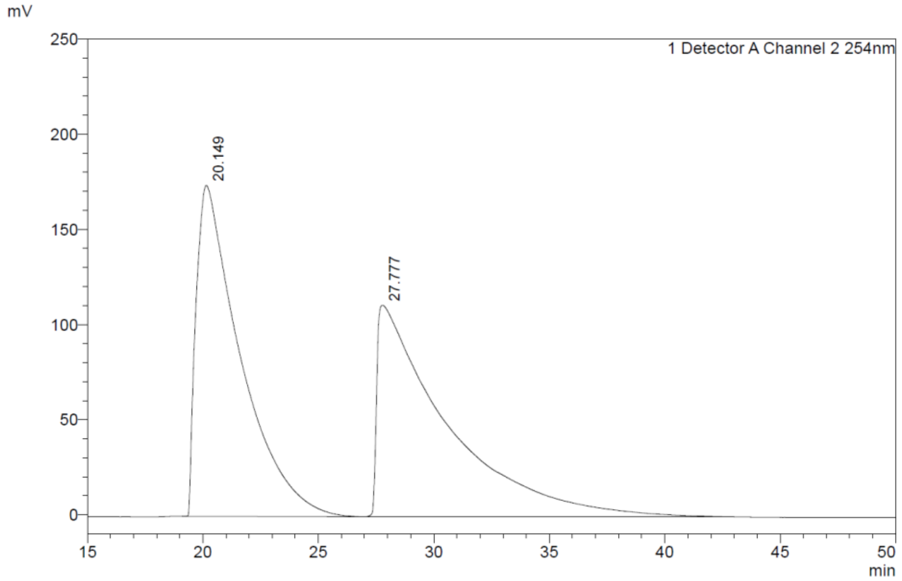

Detector A Channel 2 254nm

Peak#	Ret. Time	Area%
1	20.149	49.879
2	27.777	50.121
Total		100.000

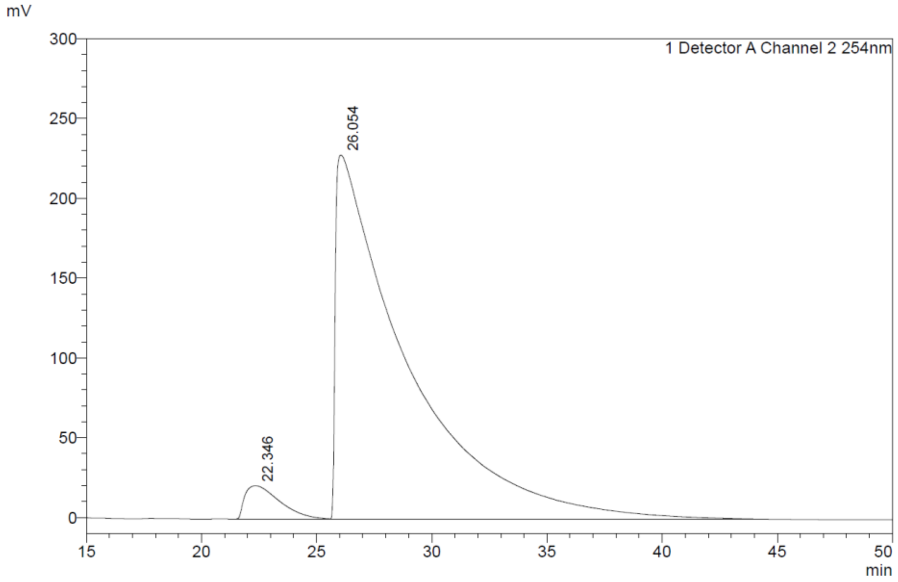

Detector A Channel 2 254nm

Peak#	Ret. Time	Area%
1	22.346	4.297
2	26.054	95.703
Total		100.000

HPLC Data for **33_{major}**: Chiralcel OD-H (90:10 hexane : IPA, flow rate 1.00 mLmin^{-1}, 254 nm, 30 °C) t_R (minor): 19.0 min, t_R (major): 34.1 min, 94:6 er.

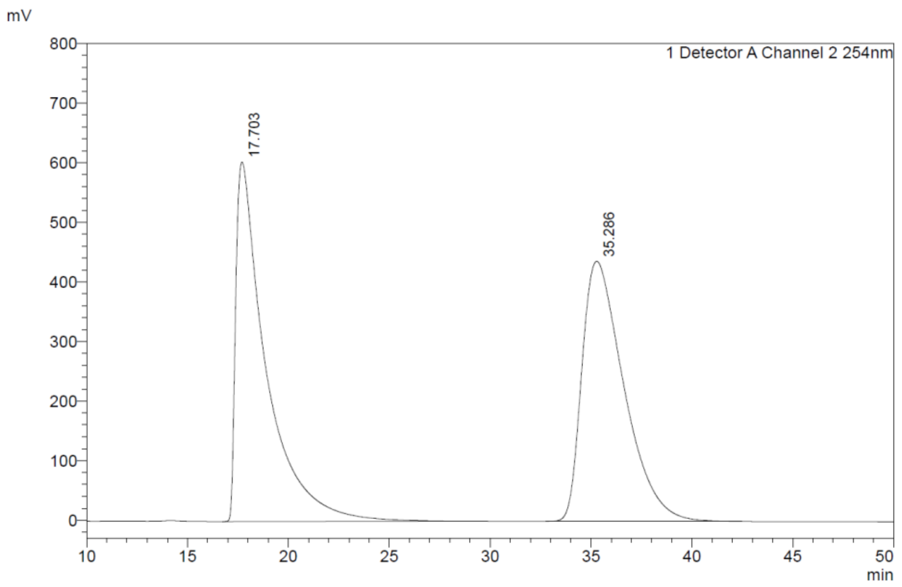

Detector A Channel 2 254nm

Peak#	Ret. Time	Area%
1	17.703	49.744
2	35.286	50.256
Total		100.000

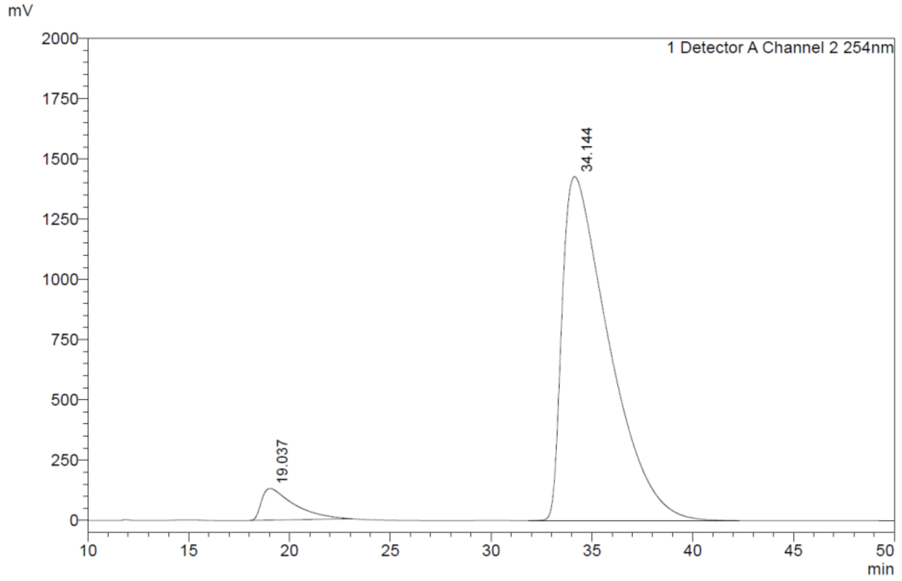

Detector A Channel 2 254nm

Peak#	Ret. Time	Area%
1	19.037	5.995
2	34.144	94.005
Total		100.000

HPLC Data for **33minor**: Chiralcel OD-H (97:3 hexane : IPA, flow rate 1.00 mLmin^{-1}, 254 nm, 30 °C) t_R (major): 23.6 min, t_R (minor): 47.2 min, 97:3 er.

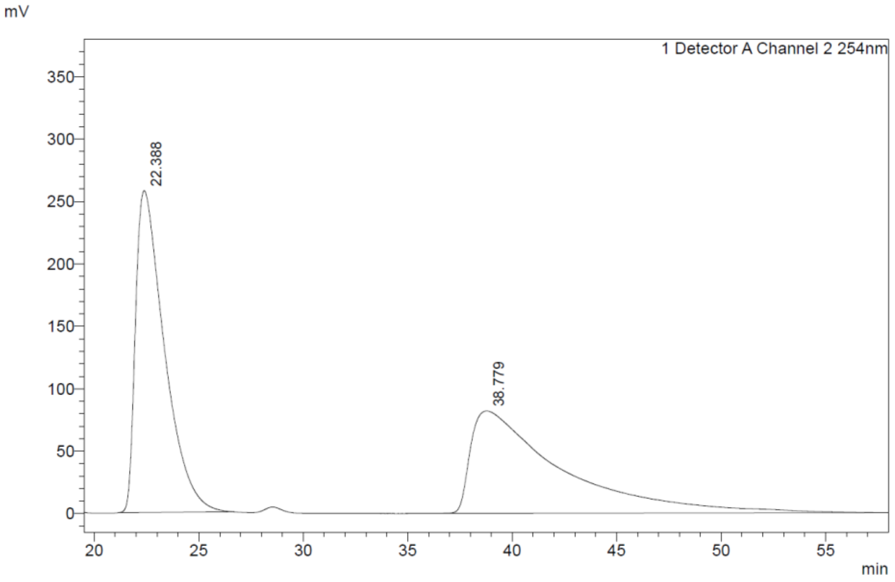

Detector A Channel 2 254nm

Peak#	Ret. Time	Area%
1	22.388	50.304
2	38.779	49.696
Total		100.000

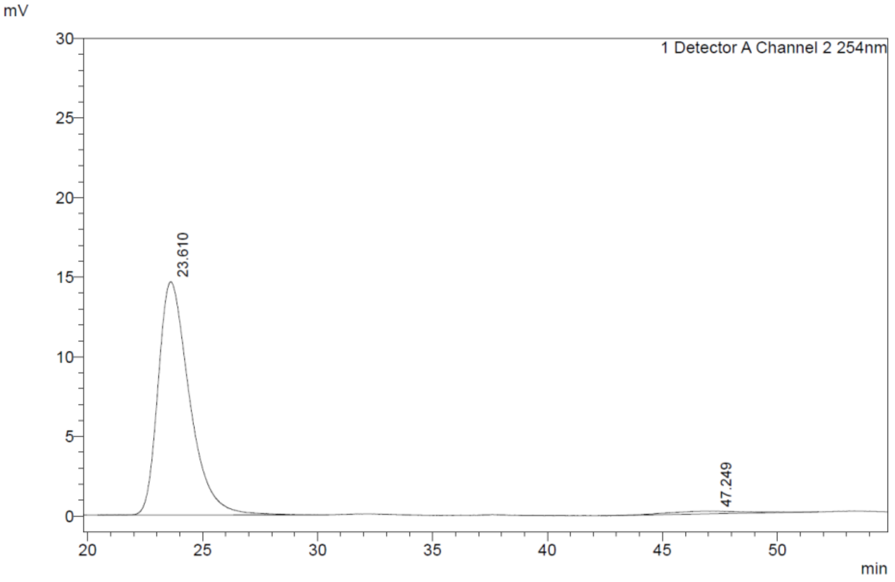

Detector A Channel 2 254nm

Peak#	Ret. Time	Area%
1	23.610	97.156
2	47.249	2.844
Total		100.000

HPLC Data for **34**$_{\mathbf{major}}$: Chiralcel OD-H (90:10 hexane : IPA, flow rate 1.00 mLmin^{-1}, 254 nm, 30 °C) t_R (minor): 41.3 min, t_R (major): 91.5 min, 92:8 er.

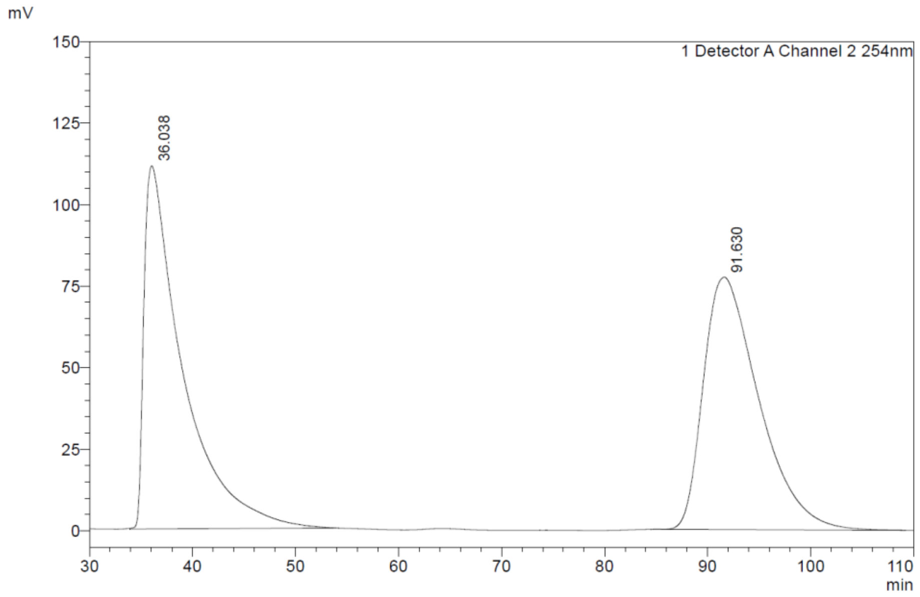

Detector A Channel 2 254nm

Peak#	Ret. Time	Area%
1	36.038	49.946
2	91.630	50.054
Total		100.000

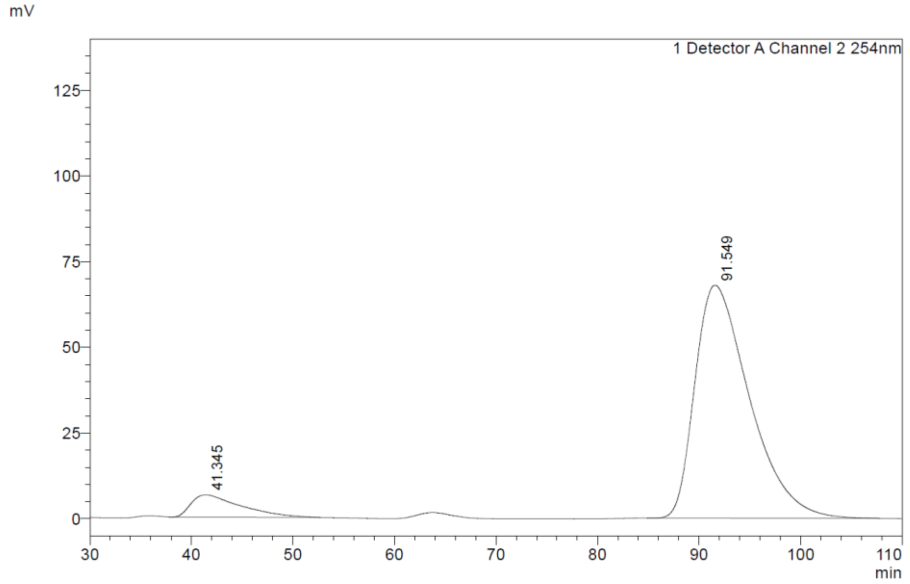

Detector A Channel 2 254nm

Peak#	Ret. Time	Area%
1	41.345	8.047
2	91.549	91.953
Total		100.000

HPLC Data for **34$_{\mathbf{minor}}$**: Chiralcel OD-H (90:10 hexane : IPA, flow rate 1.00 mLmin^{-1}, 254 nm, 30 °C) t_R (major): 14.1 min, t_R (minor): 20.4 min, 96:4 er.

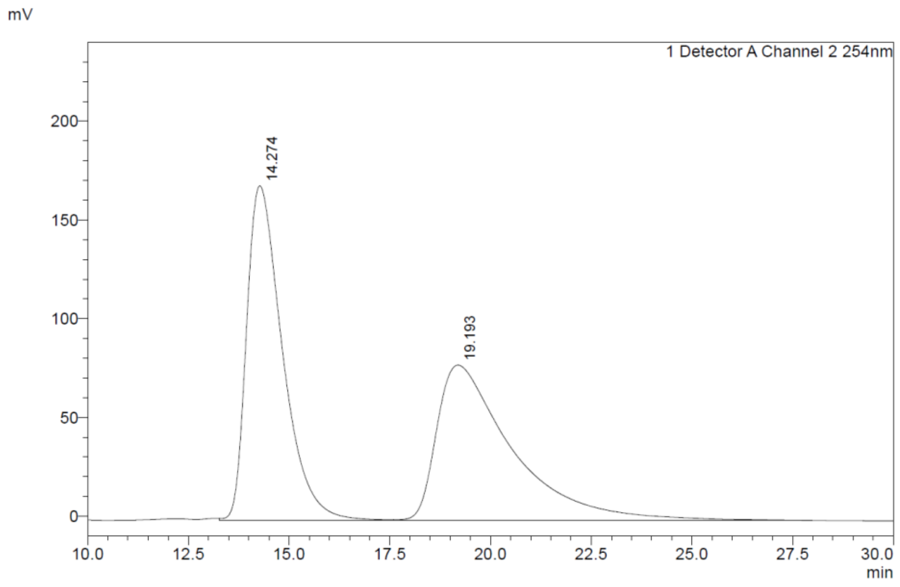

Detector A Channel 2 254nm

Peak#	Ret. Time	Area%
1	14.274	50.601
2	19.193	49.399
Total		100.000

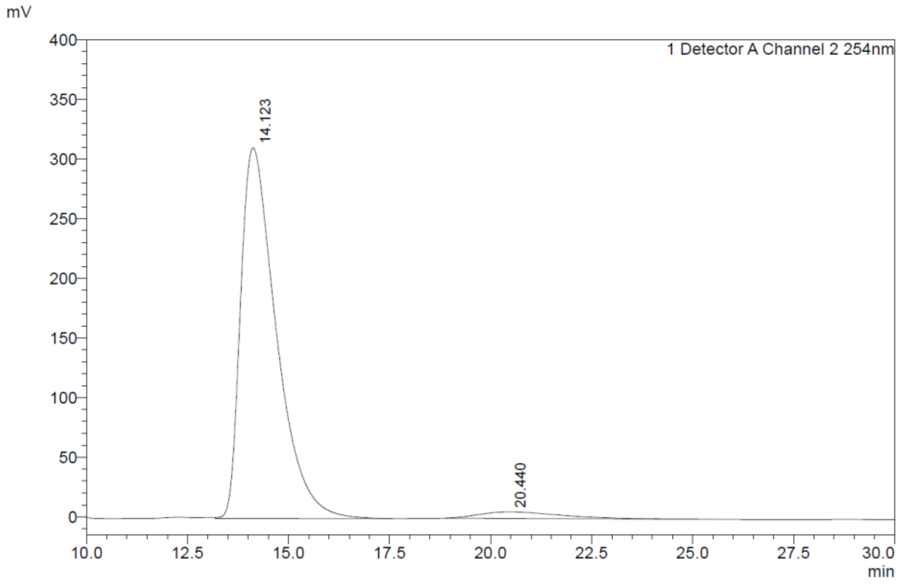

Detector A Channel 2 254nm

Peak#	Ret. Time	Area%
1	14.123	96.226
2	20.440	3.774
Total		100.000

HPLC Data for **35**$_{\mathbf{major}}$: Chiralcel OD-H (90:10 hexane : IPA, flow rate 1.00 mLmin^{-1}, 254 nm, 30 °C) t_R (major): 14.9 min, t_R (minor): 67.9 min, 95:5 er.

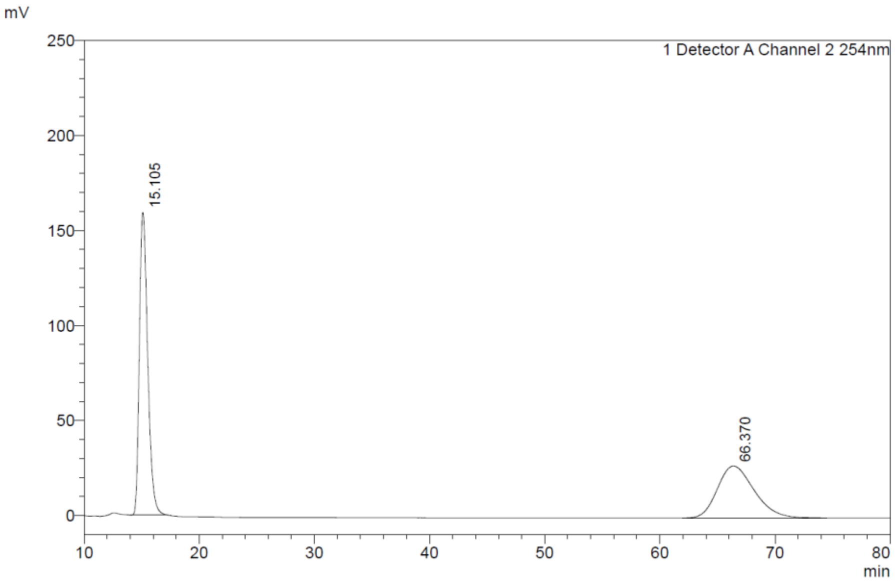

Detector A Channel 2 254nm

Peak#	Ret. Time	Area%
1	15.105	51.337
2	66.370	48.663
Total		100.000

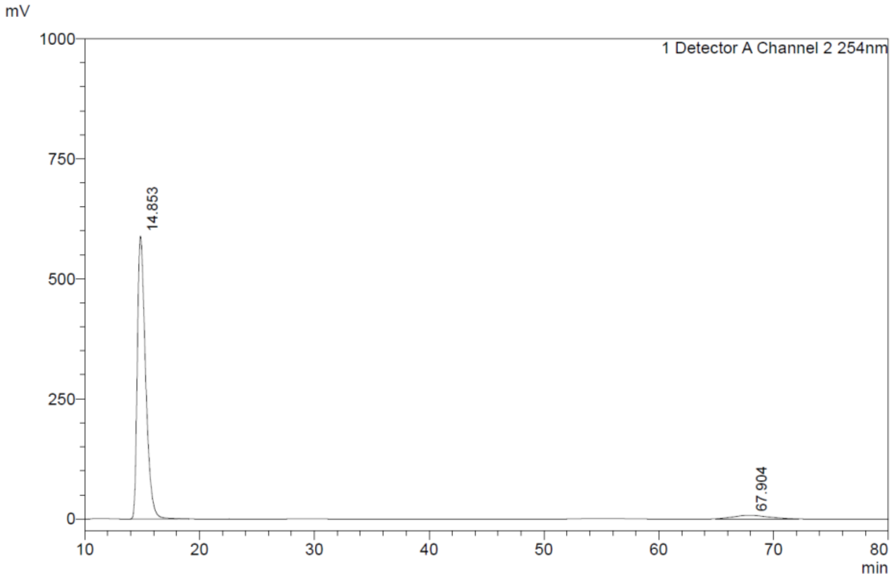

Detector A Channel 2 254nm

Peak#	Ret. Time	Area%
1	14.853	95.289
2	67.904	4.711
Total		100.000

HPLC Data for **35**$_{\mathbf{minor}}$: Chiralpak AD-H (95:5 hexane : IPA, flow rate 1.00 mLmin^{-1}, 254 nm, 30 °C) t_R (major): 29.0 min, t_R (minor): 51.1 min, 94:6 er.

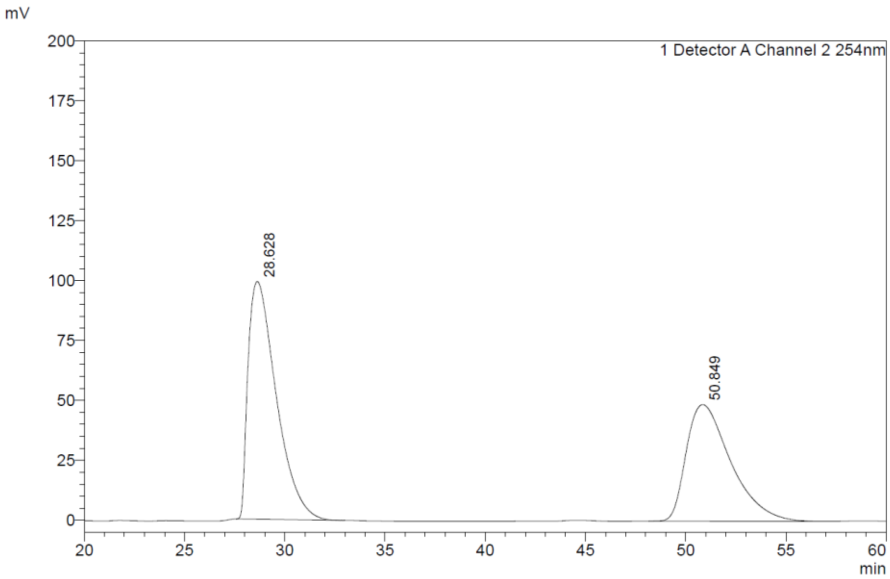

Detector A Channel 2 254nm

Peak#	Ret. Time	Area%
1	28.628	50.799
2	50.849	49.201
Total		100.000

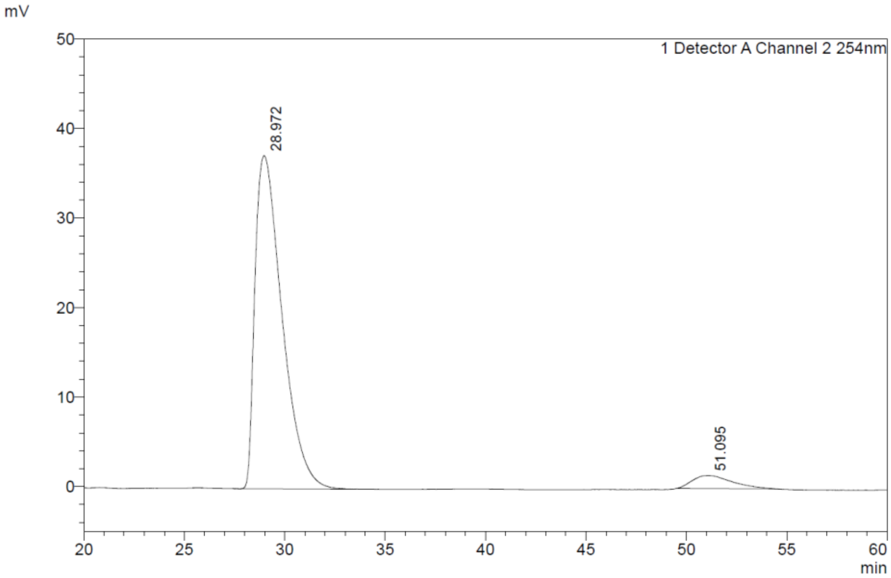

HPLC Data for **13**: Chiralcel OD-H (95:5 hexane : IPA, flow rate 1.00 mL.min^{-1}, 254 nm, 30 °C) t_R (major): 9.7 min, t_R (minor): 13.5 min, 98:2 er.

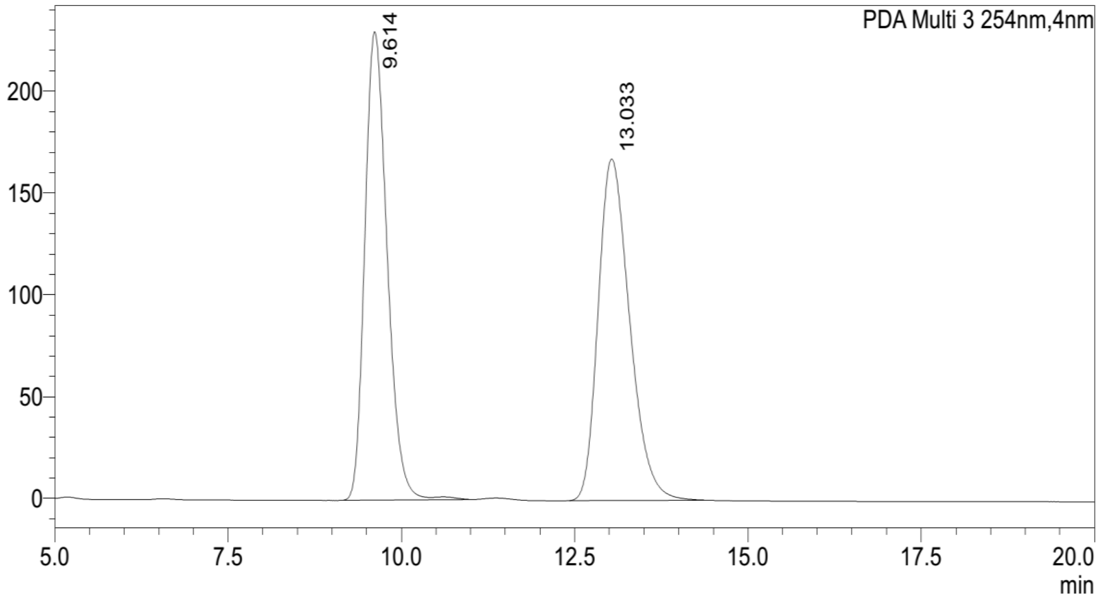

Peak#	Ret. Time	Area%
1	9.614	49.376
2	13.033	50.624
Total		100.000

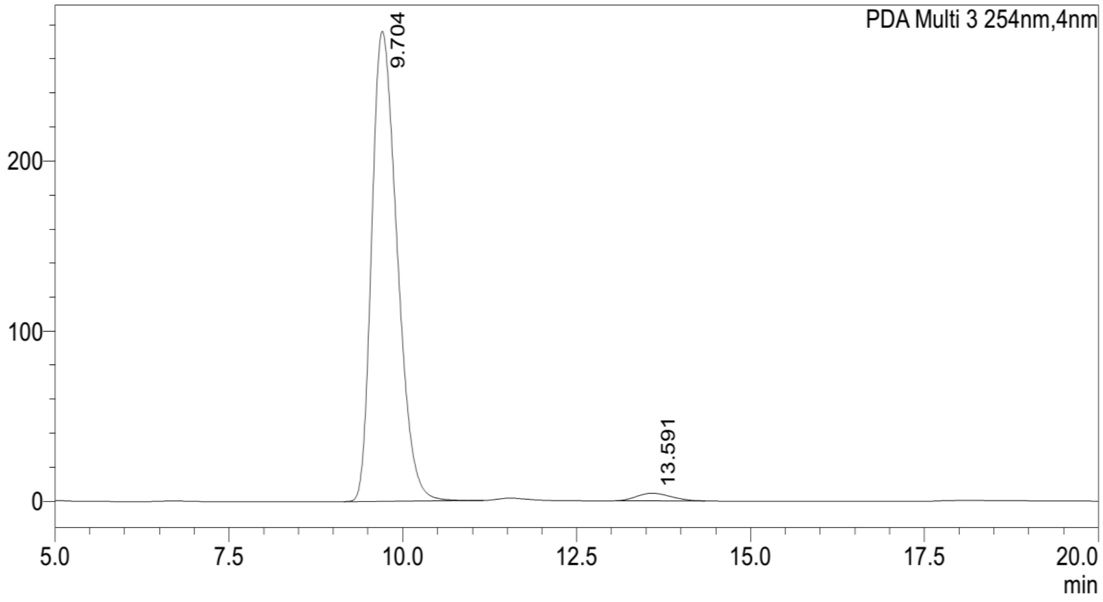

Peak#	Ret. Time	Area%
1	9.704	97.864
2	13.591	2.136
Total		100.000

HPLC Data for **53**: Chiralcel OD-H (93:7 hexane : IPA, flow rate 1.00 mL.min^{-1}, 254 nm, 30 °C) t_R (major): 26.1 min, t_R (minor): 39.4 min, 98:2 er.

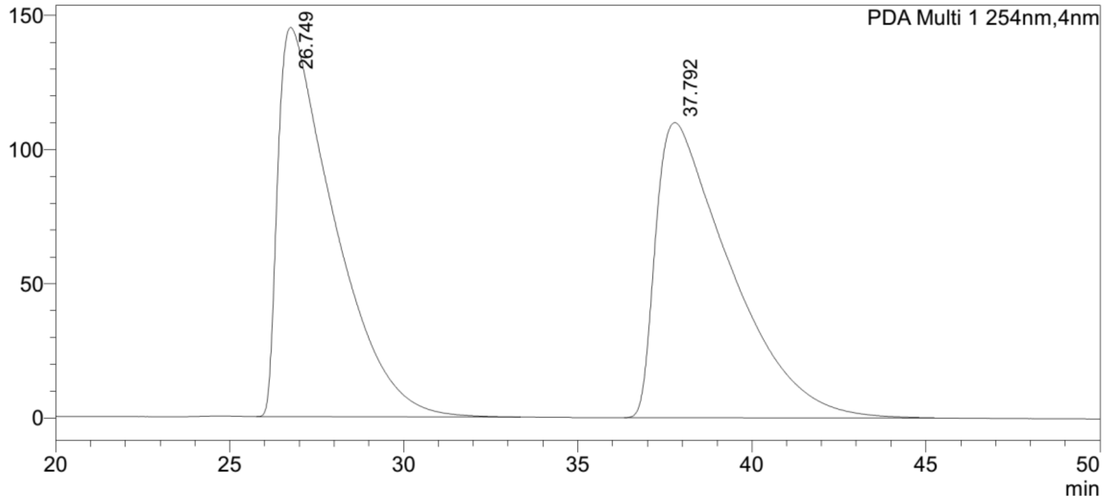

Peak#	Ret. Time	Area%
1	26.749	49.858
2	37.792	50.142
Total		100.000

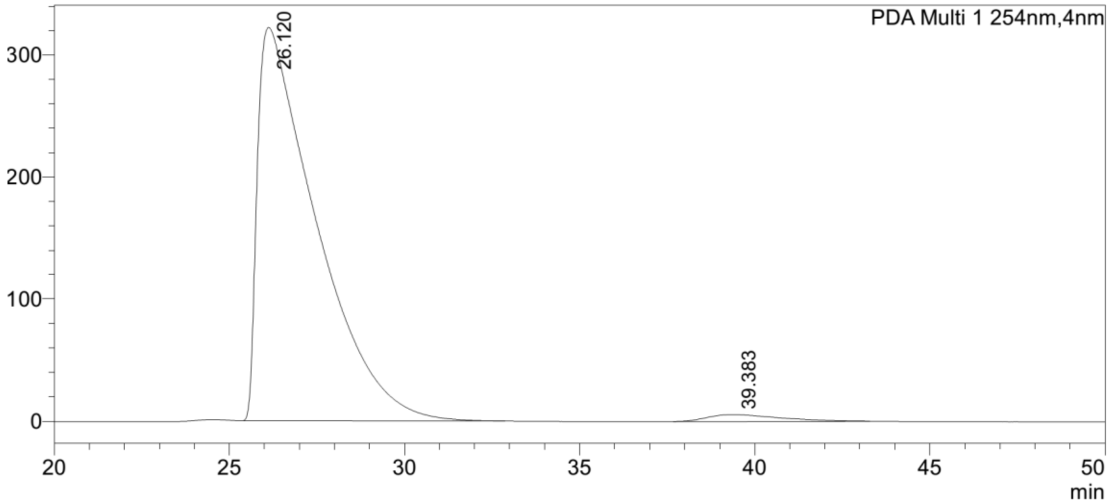

Peak#	Ret. Time	Area%
1	26.120	97.942
2	39.383	2.058
Total		100.000

HPLC Data for **54**: Chiralcel AS-H (96.5:3.5 hexane : IPA, flow rate 1.00 mL.min^{-1}, 254 nm, 30 °C) t_R (major): 20.2 min, t_R (minor): 26.6 min, 98:2 er.

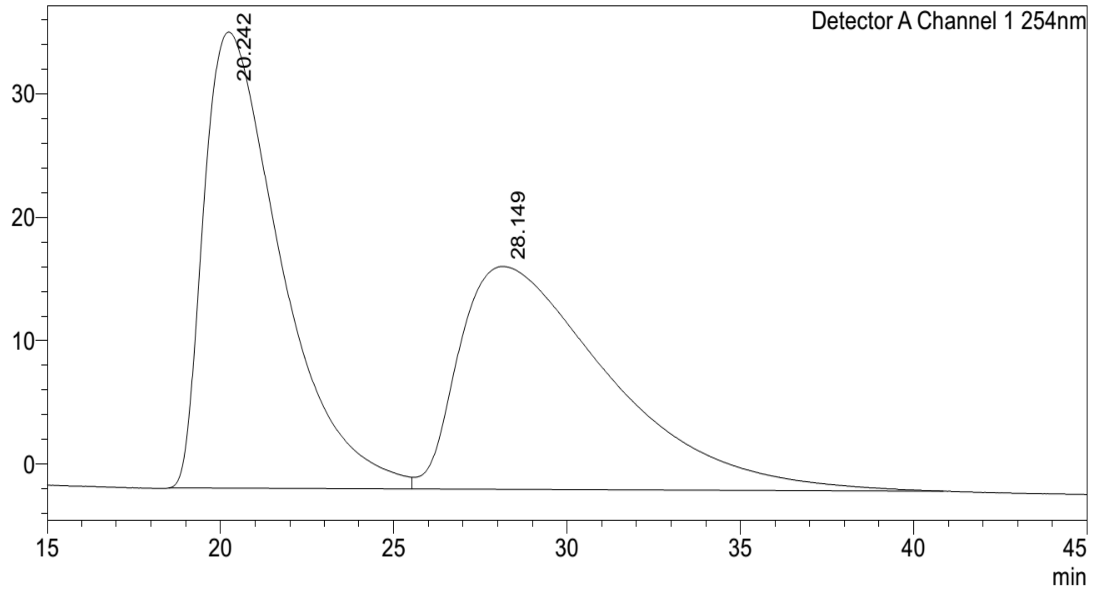

Peak#	Ret. Time	Area%
1	20.242	51.186
2	28.149	48.814
Total		100.000

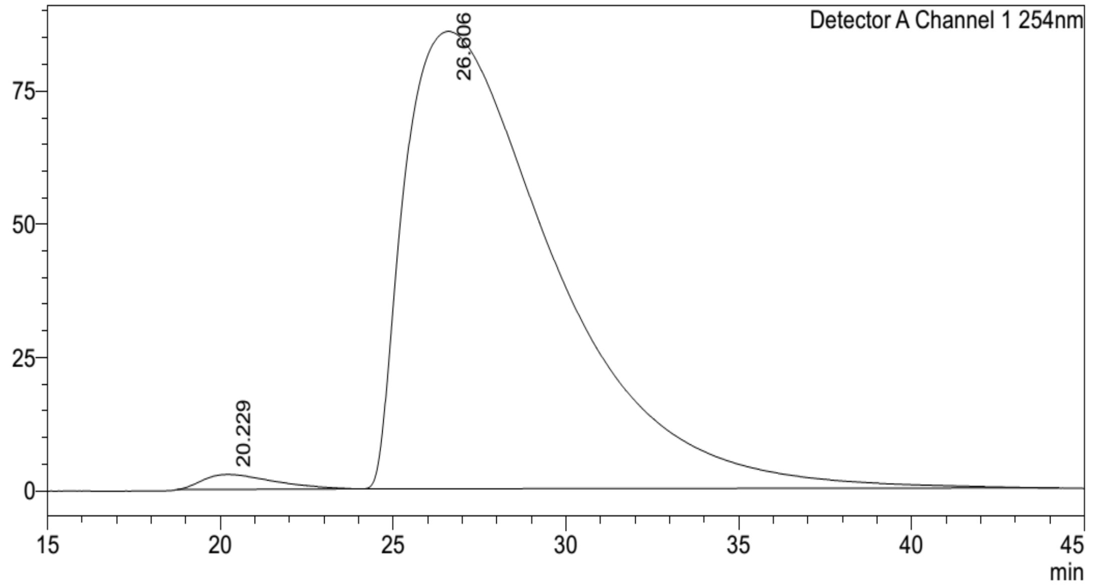

Peak#	Ret. Time	Area%
1	20.229	1.526
2	26.606	98.474
Total		100.000

HPLC Data for **55**: Chiralcel OD-H (93:7 hexane : IPA, flow rate 1.00 mL.min^{-1}, 254 nm, 30 °C) t_R (major): 34.5 min, t_R (minor): 48.7 min, >99:1 er.

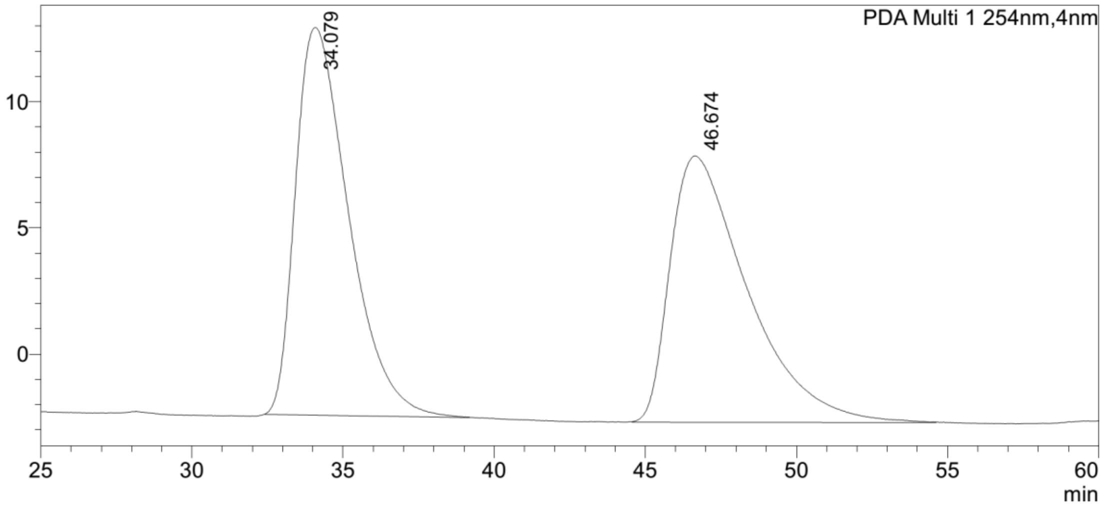

Peak#	Ret. Time	Area%
1	34.079	50.135
2	46.674	49.865
Total		100.000

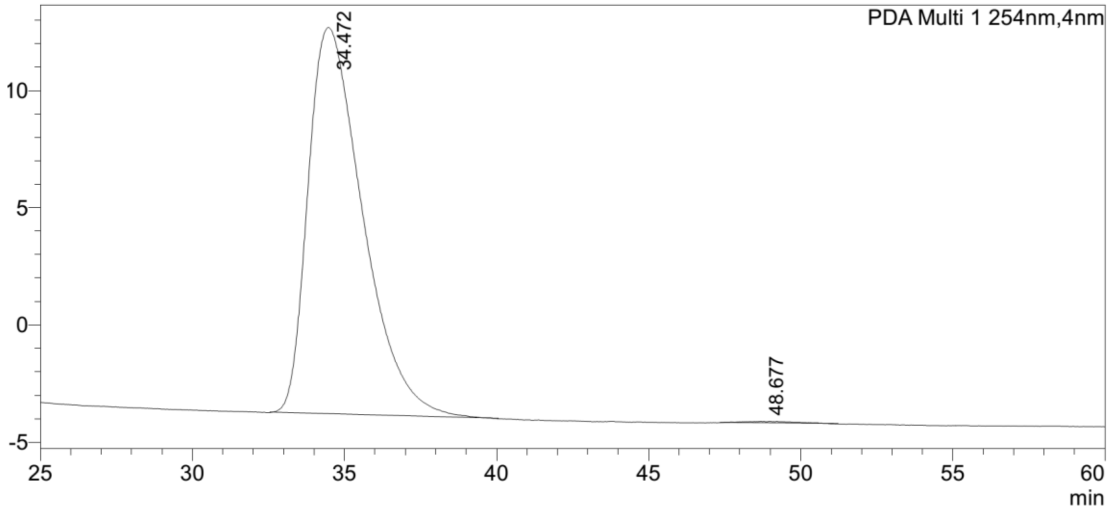

Peak#	Ret. Time	Area%
1	34.472	99.544
2	48.677	0.456
Total		100.000

HPLC Data for **56**: Chiralcel OD-H (90:10 hexane : IPA, flow rate 1.00 mL.min^{-1}, 254 nm, 30 °C) t_R (minor): 14.3 min, t_R (major): 22.2 min, 2:98 er.

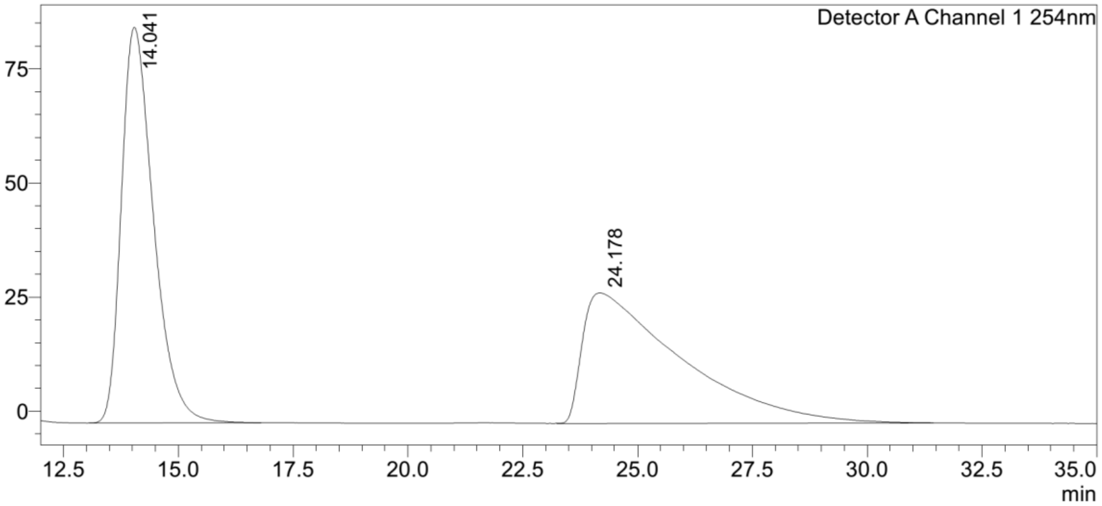

Peak#	Ret. Time	Area%
1	14.041	49.805
2	24.178	50.195
Total		100.000

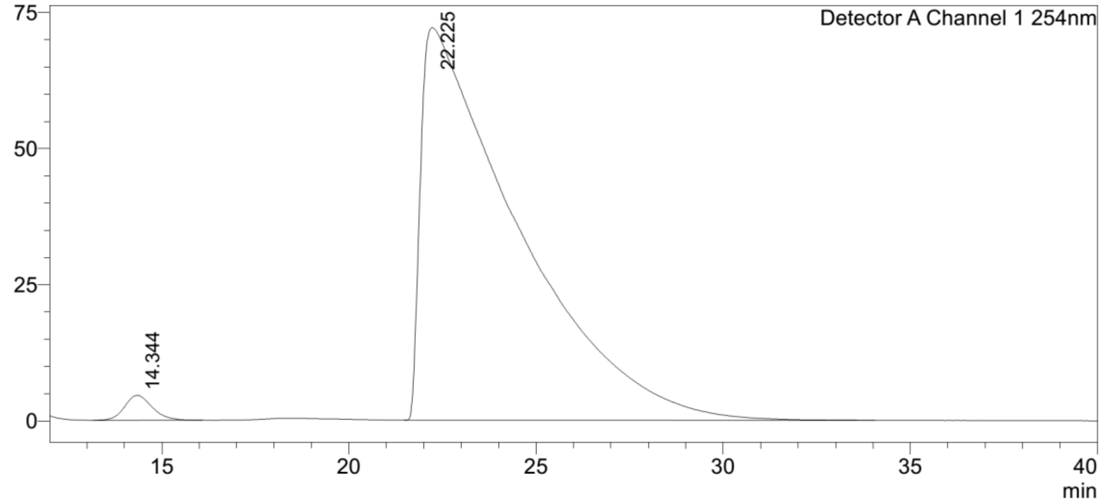

Peak#	Ret. Time	Area%
1	14.344	1.784
2	22.225	98.216
Total		100.000

HPLC Data for **57**: Chiralcel OD-H (93:7 hexane : IPA, flow rate 1.00 mL.min^{-1}, 254 nm, 30 °C) t_R (minor): 30.5 min, t_R (major): 34.3 min, 2:98 er.

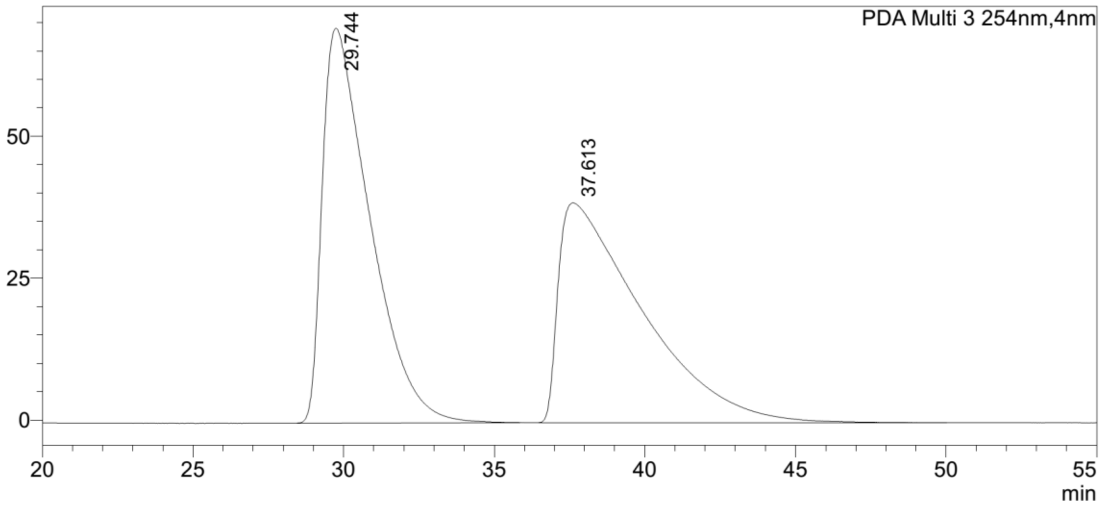

Peak#	Ret. Time	Area%
1	29.744	50.596
2	37.613	49.404
Total		100.000

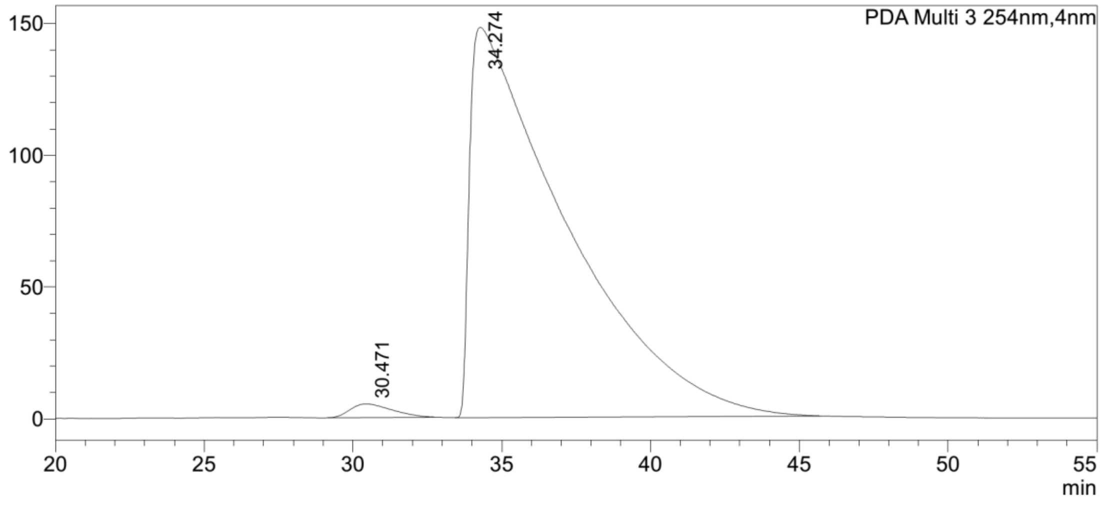

Peak#	Ret. Time	Area%
1	30.471	1.517
2	34.274	98.483
Total		100.000

HPLC Data for **58**: Chiralcel OD-H (90:10 hexane : IPA, flow rate 1.00 mL.min^{-1}, 254 nm, 30 °C) t_R (minor): 15.9 min, t_R (major): 19.5 min, 98:2 er.

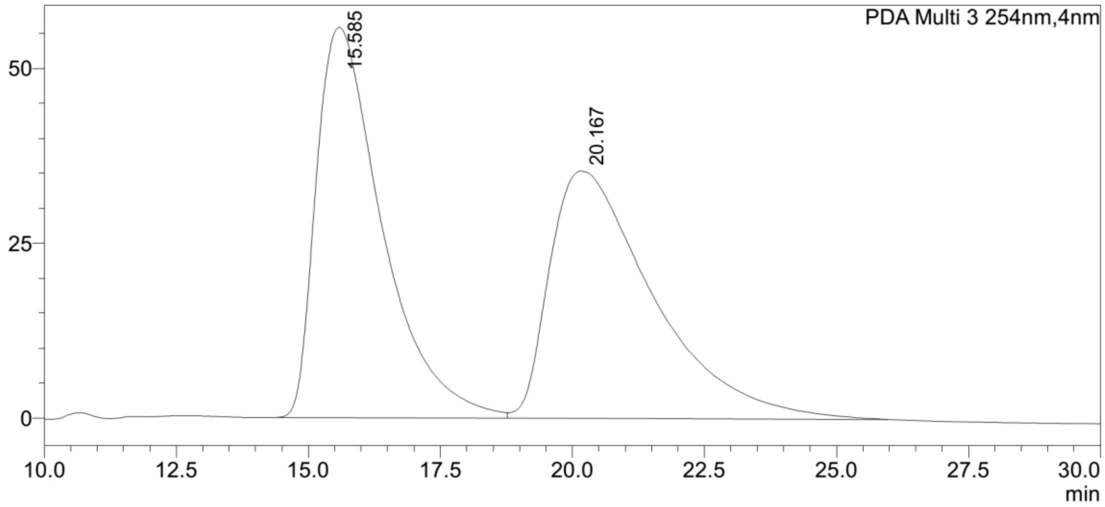

Peak#	Ret. Time	Area%
1	15.585	50.062
2	20.167	49.938
Total		100.000

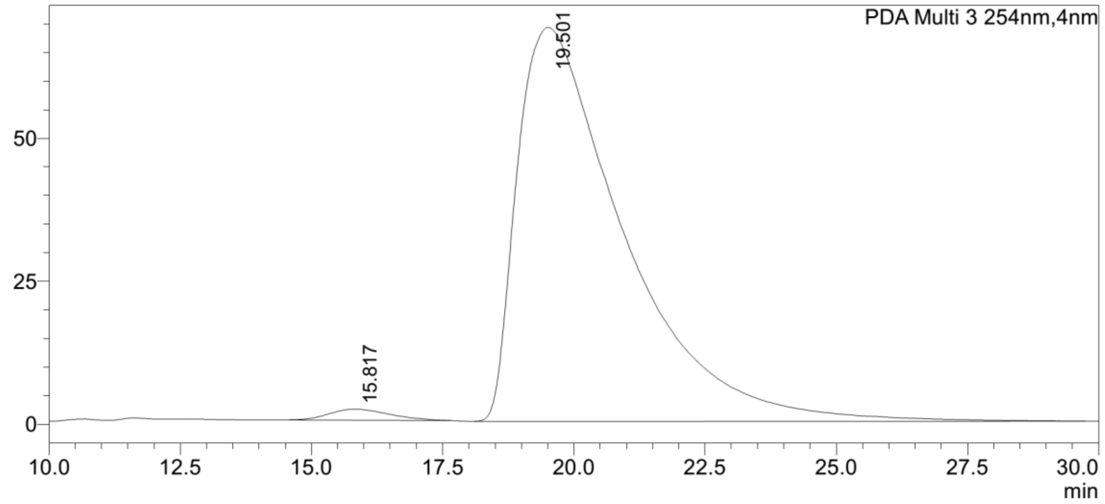

Peak#	Ret. Time	Area%
1	15.817	1.587
2	19.501	98.413
Total		100.000

HPLC Data for **59**: Chiralcel IC (93:7 hexane : IPA, flow rate 1.00 mL.min^{-1}, 254 nm, 50 °C) t_R (major): 52.7 min, t_R (minor): 67.8 min, 93:7 er.

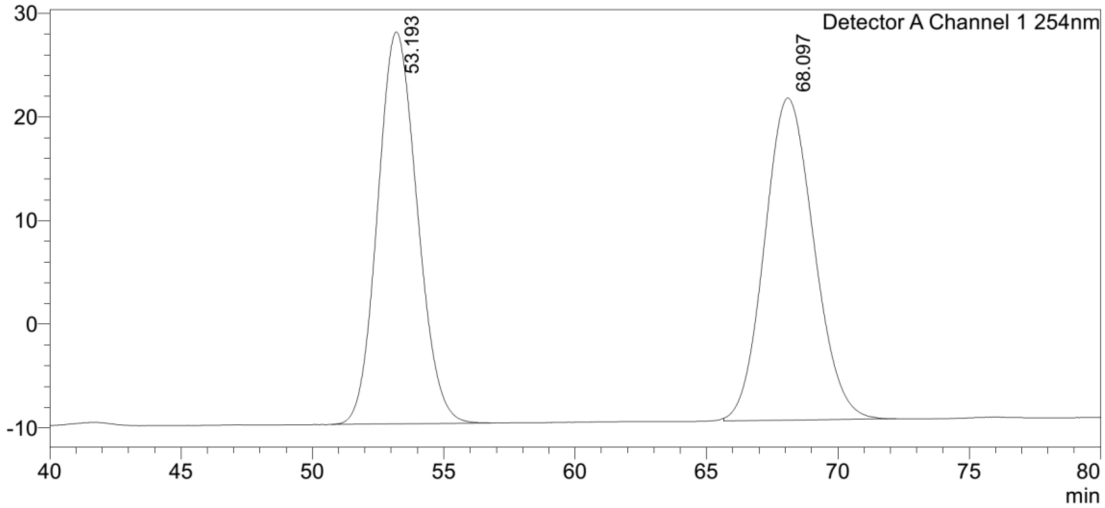

Peak#	Ret. Time	Area%
1	53.193	49.366
2	68.097	50.634
Total		100.000

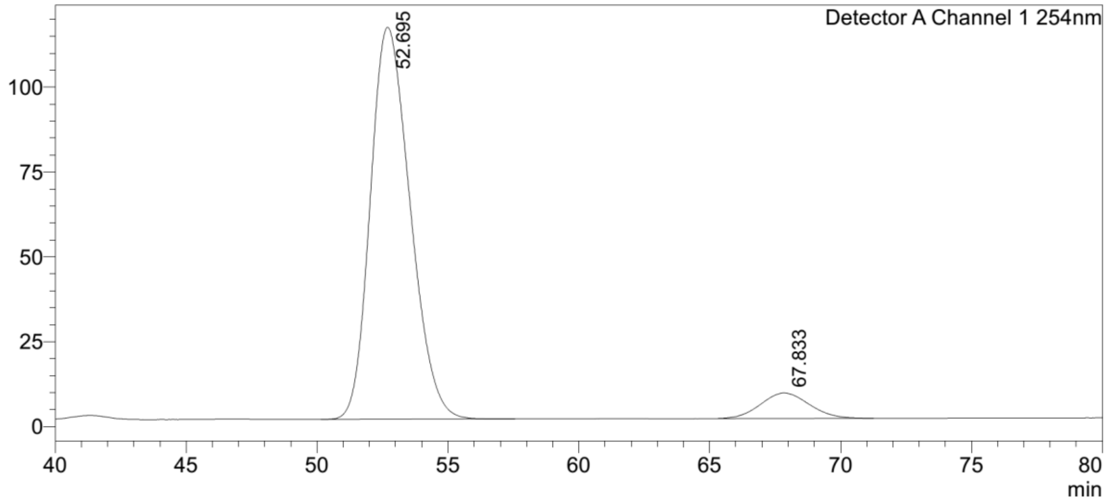

Peak#	Ret. Time	Area%
1	52.695	92.676
2	67.833	7.324
Total		100.000

HPLC Data for **60**: Chiralcel OD-H (93:7 hexane : IPA, flow rate 1.00 mL.min^{-1}, 254 nm, 30 ℃) t_R (major): 29.6 min, t_R (minor): 53.2 min, 95:5 er.

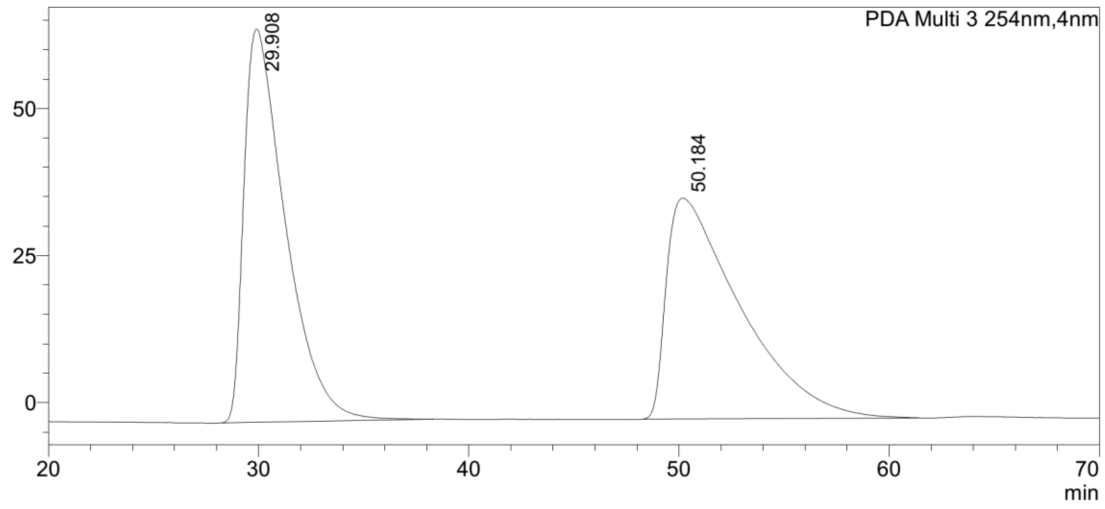

Peak#	Ret. Time	Area%
1	29.908	49.962
2	50.184	50.038
Total		100.000

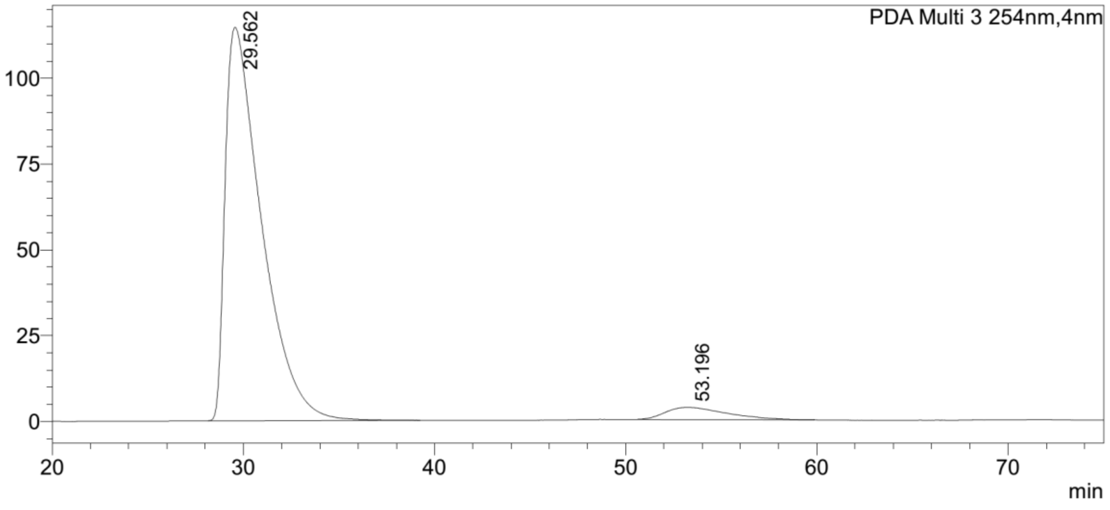

Peak#	Ret. Time	Area%
1	29.562	95.022
2	53.196	4.978
Total		100.000

HPLC Data for **61**: Chiralcel OD-H (90:10 hexane : IPA, flow rate 1.00 mL.min^{-1}, 254 nm, 30 °C) t_R (major): 15.0, t_R (minor): 34.1 min, 90:10 er.

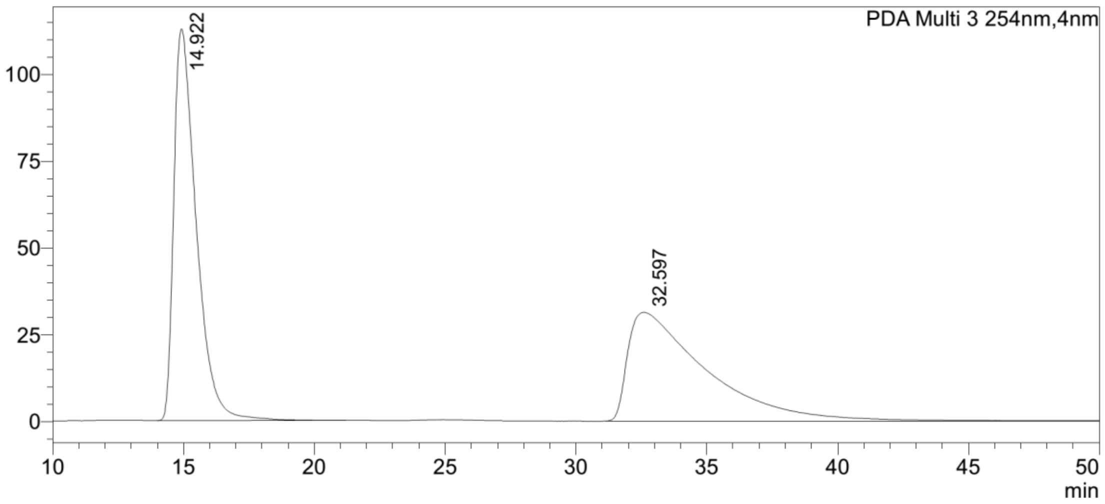

Peak#	Ret. Time	Area%
1	14.922	50.903
2	32.597	49.097
Total		100.000

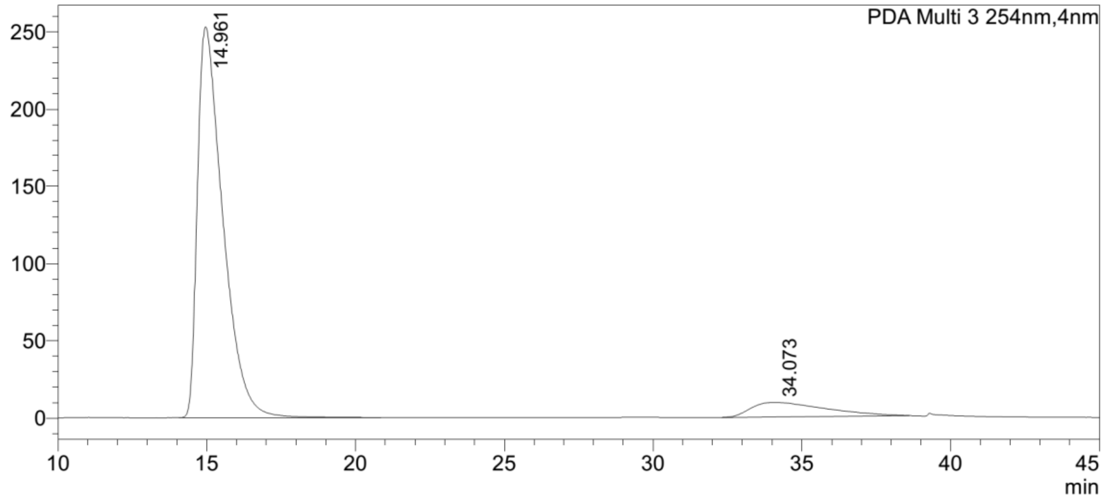

Peak#	Ret. Time	Area%
1	14.961	90.202
2	34.073	9.798
Total		100.000

HPLC Data for **62**: Chiralcel OD-H (90:10 hexane : IPA, flow rate 1.00 mL.min^{-1}, 254 nm, 30 °C) t_R (major): 20.2 min, t_R (minor): 27.7 min, >99:1 er.

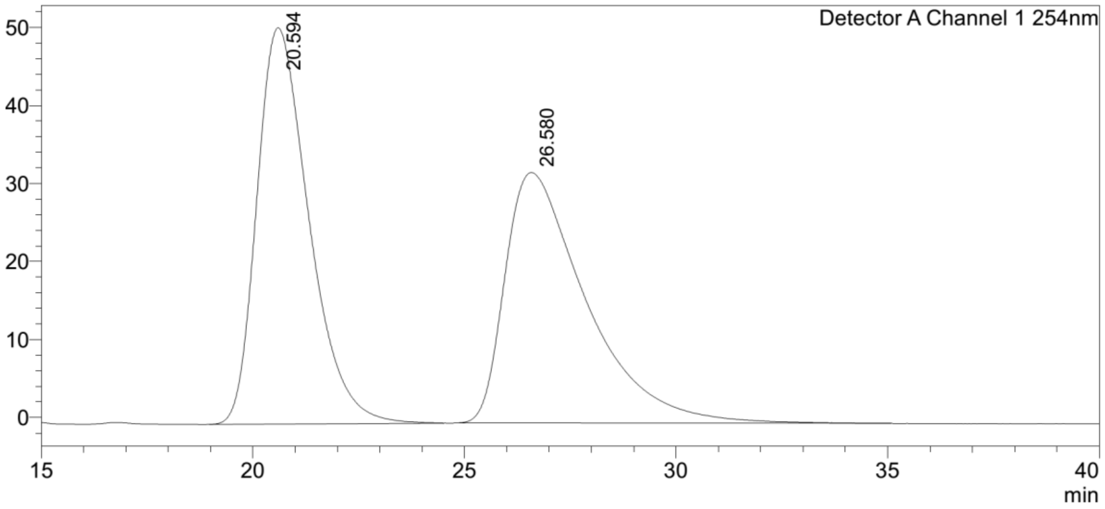

Peak#	Ret. Time	Area%
1	20.594	50.309
2	26.580	49.691
Total		100.000

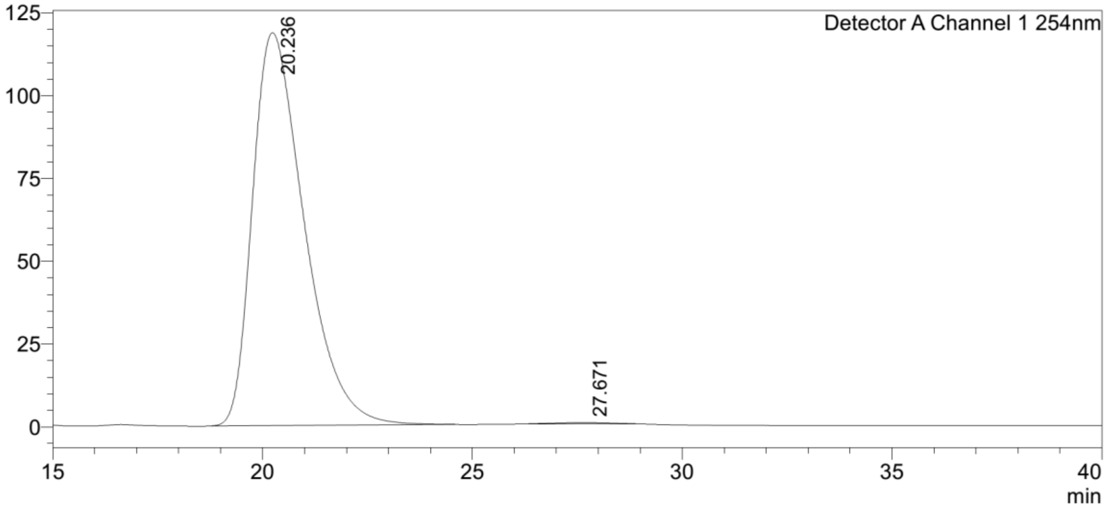

Peak#	Ret. Time	Area%
1	20.236	99.636
2	27.671	0.364
Total		100.000

HPLC Data for **63**: Chiralcel OD-H (90:10 hexane : IPA, flow rate 1.00 mL.min^{-1}, 254 nm, 30 °C) t_R (major): 30.2 min, t_R (minor): 52.1 min, 99:1 ee.

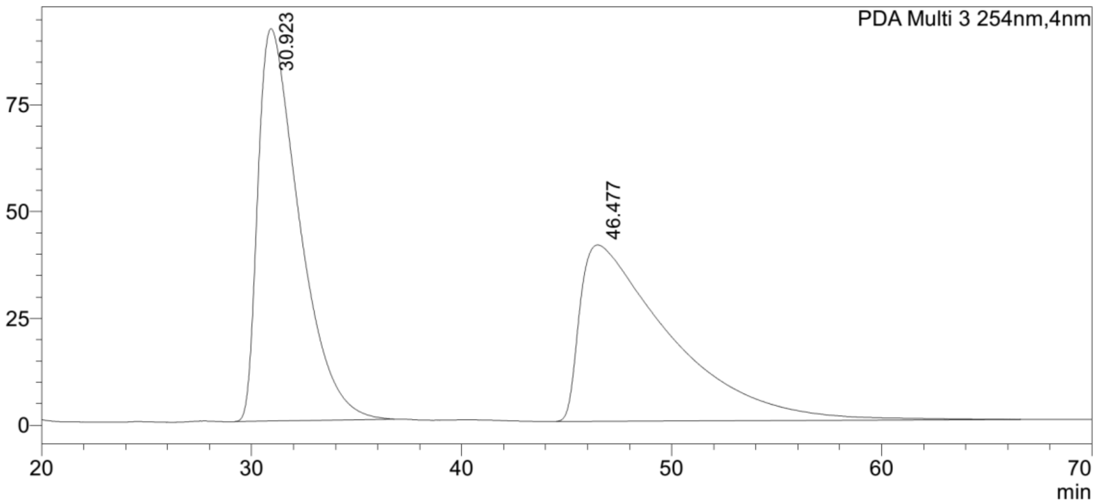

Peak#	Ret. Time	Area%
1	30.923	50.394
2	46.477	49.606
Total		100.000

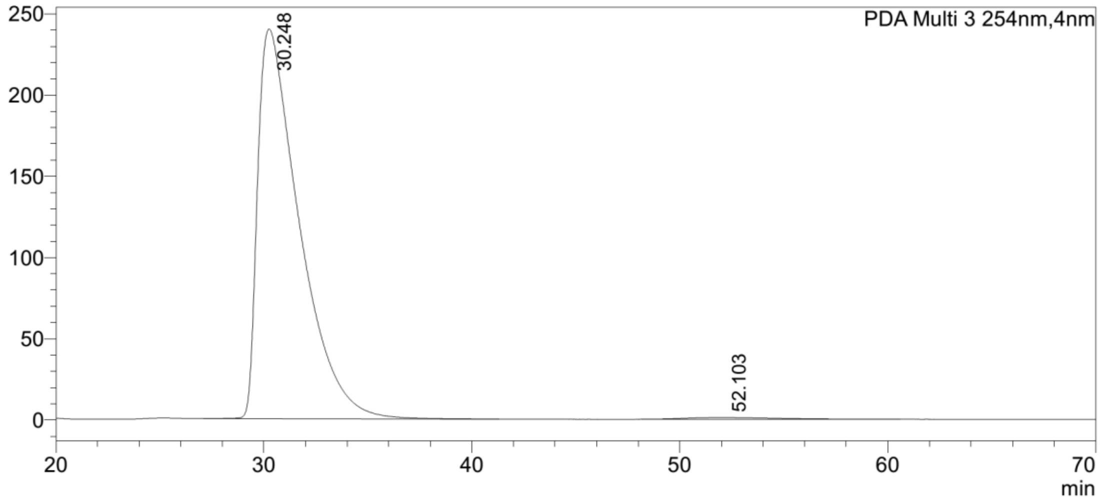

Peak#	Ret. Time	Area%
1	30.248	99.013
2	52.103	0.987
Total		100.000

HPLC Data for **64**: Chiralcel OD-H (90:10 hexane : IPA, flow rate 1.00 mL.min^{-1}, 254 nm, 30 °C) t_R (major): 22.2 min, t_R (minor): 46.6 min, 98:2 er.

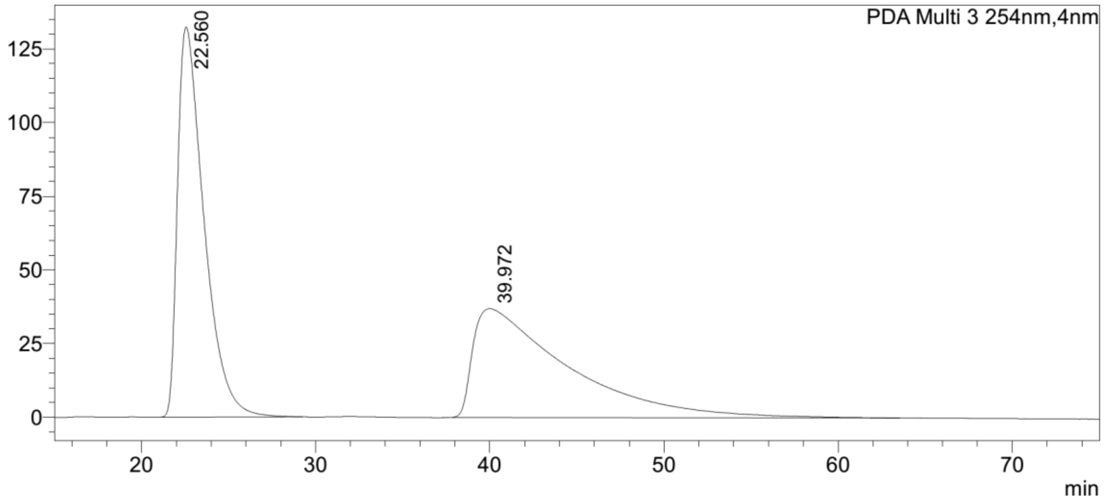

Peak#	Ret. Time	Area%
1	22.560	50.141
2	39.972	49.859
Total		100.000

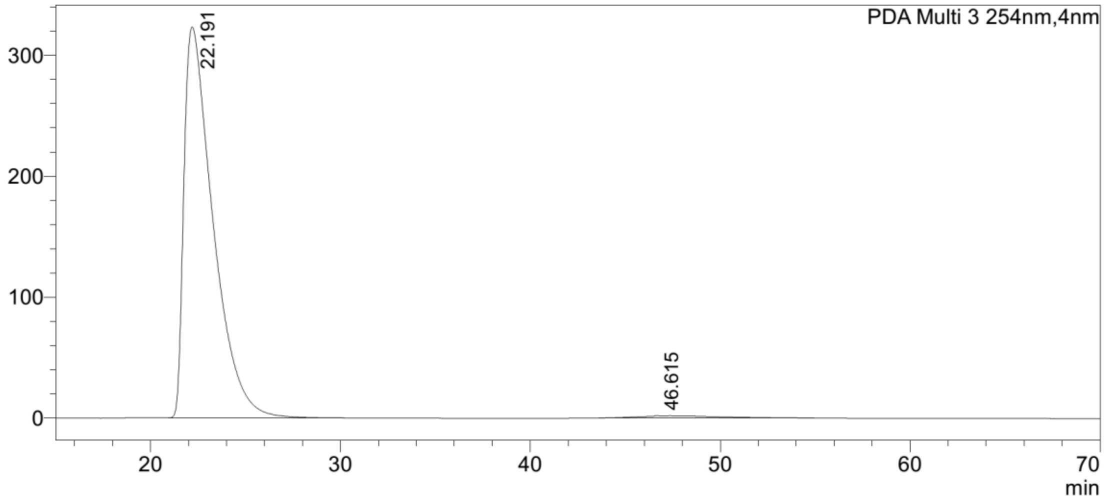

Peak#	Ret. Time	Area%
1	22.191	98.355
2	46.615	1.645
Total		100.000

HPLC Data for **65**: Chiralcel OD-H (95:5 hexane : IPA, flow rate 1.00 mL.min^{-1}, 254 nm, 30 °C) t_R (major): 136.9 min, t_R (minor): 201.3 min, 92:8 er.

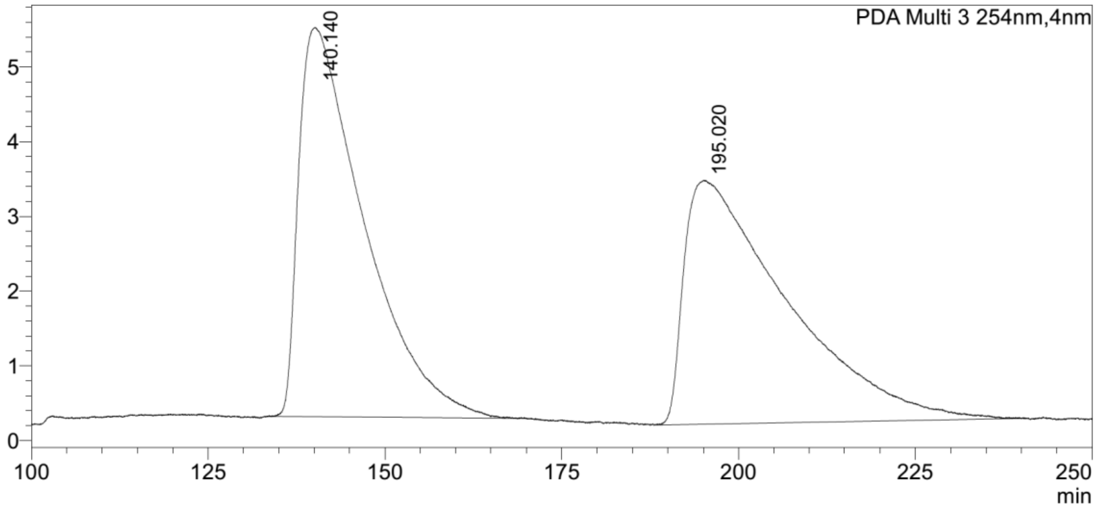

Peak#	Ret. Time	Area%
1	140.140	50.320
2	195.020	49.680
Total		100.000

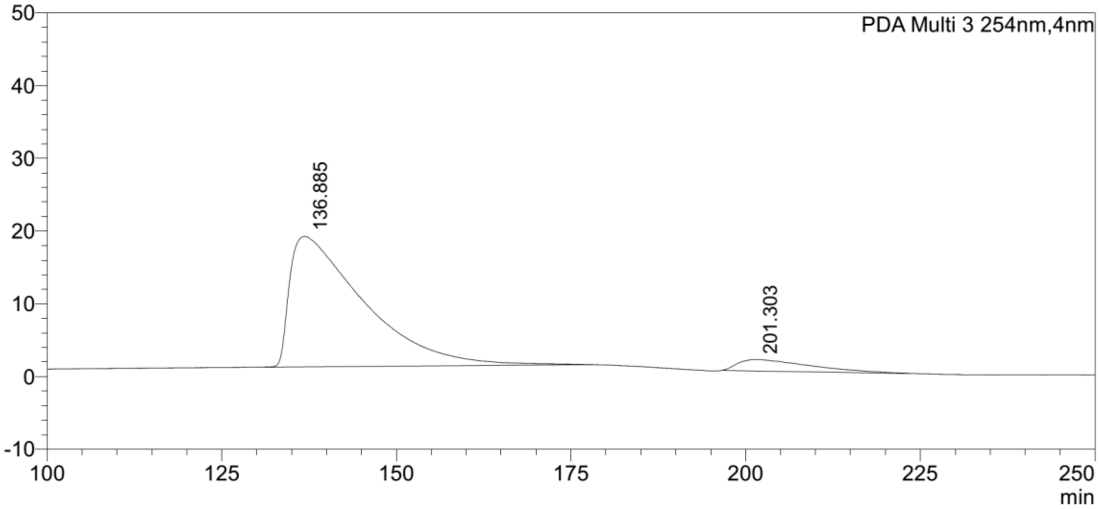

Peak#	Ret. Time	Area%
1	136.885	92.190
2	201.303	7.810
Total		100.000

HPLC Data for **66**: Chiralcel OD-H (95:5 hexane : IPA, flow rate 1.00 mL.min^{-1}, 254 nm, 30 ℃) t_R (major): 49.5 min, t_R (minor): 62.2 min, 98:2 er.

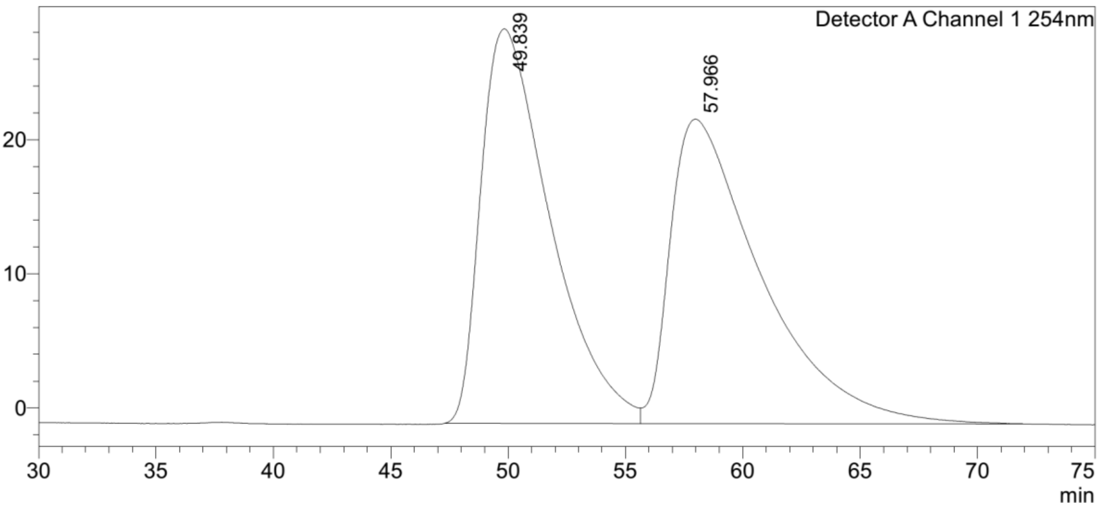

Peak#	Ret. Time	Area%
1	49.839	49.732
2	57.966	50.268
Total		100.000

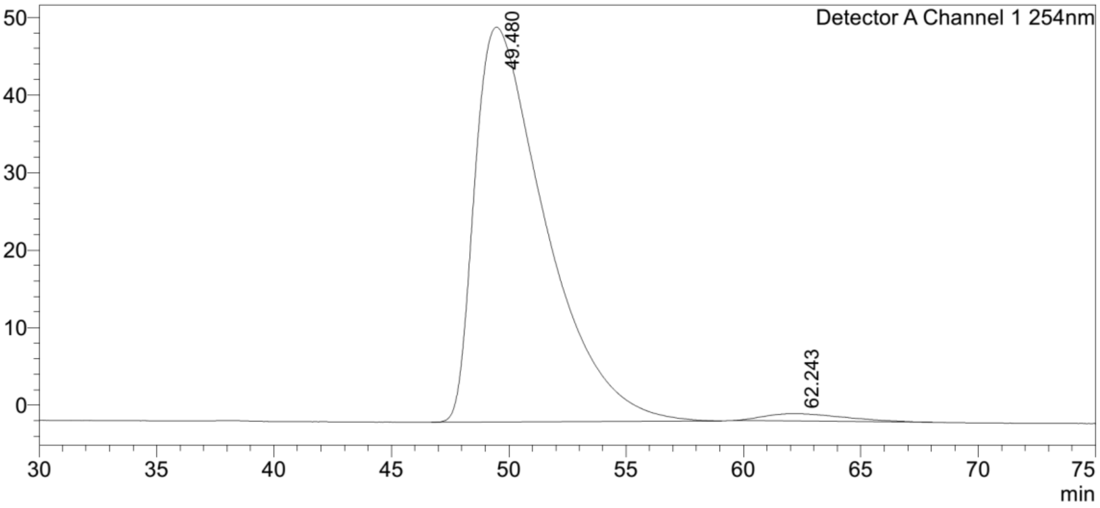

Peak#	Ret. Time	Area%
1	49.480	97.889
2	62.243	2.111
Total		100.000

HPLC Data for **67**: Chiralcel OD-H (90:10 hexane : IPA, flow rate 1.00 mL.min^{-1}, 254 nm, 30 °C) t_R (major): 19.2 min, t_R (minor): 26.1 min, 98:2 er.

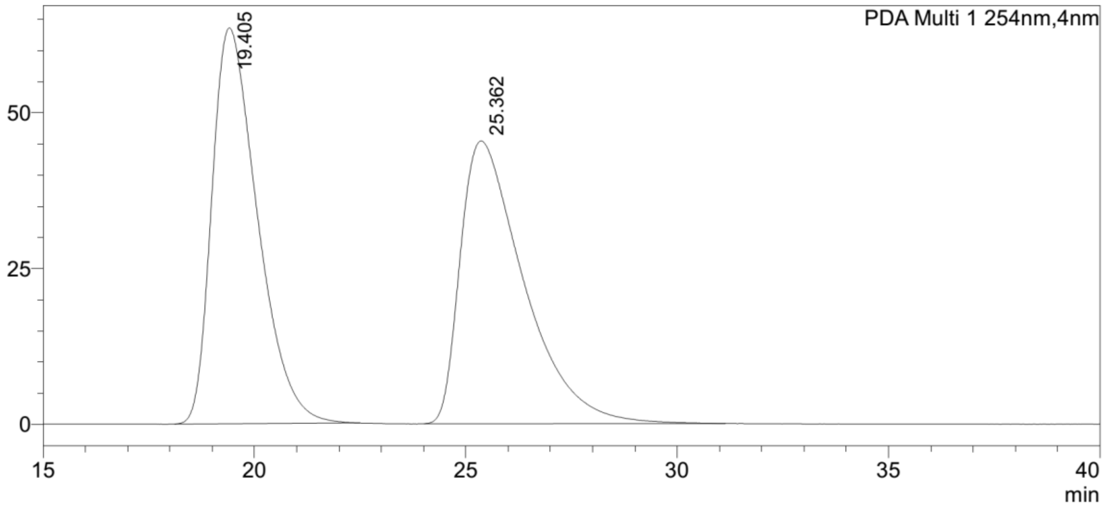

Peak#	Ret. Time	Area%
1	19.405	50.121
2	25.362	49.879
Total		100.000

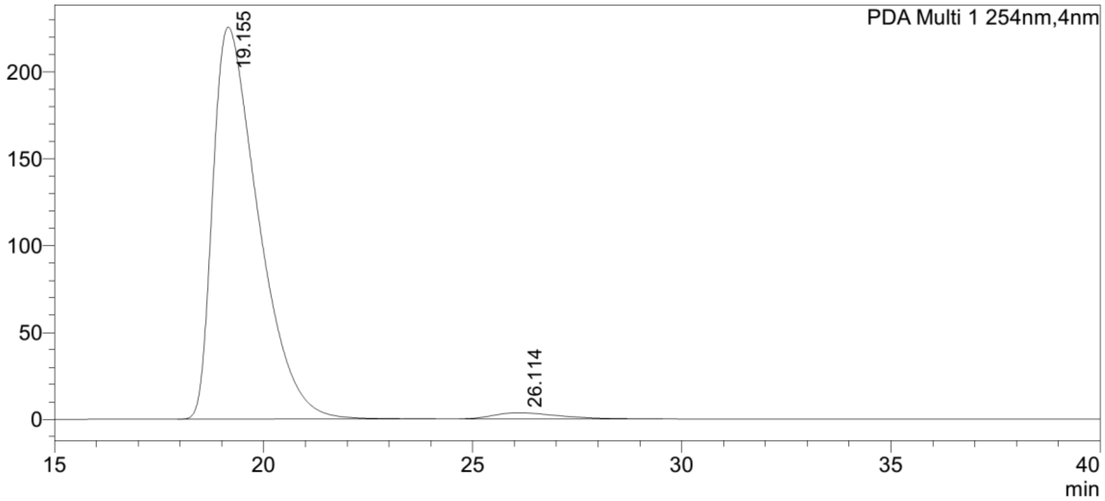

Peak#	Ret. Time	Area%
1	19.155	97.802
2	26.114	2.198
Total		100.000

HPLC Data for **68**: Chiralcel OD-H (90:10 hexane : IPA, flow rate 1.00 mL.min^{-1}, 254 nm, 30 °C) t_R (major): 20.2 min, t_R (minor): 27.5 min, >99:1 er.

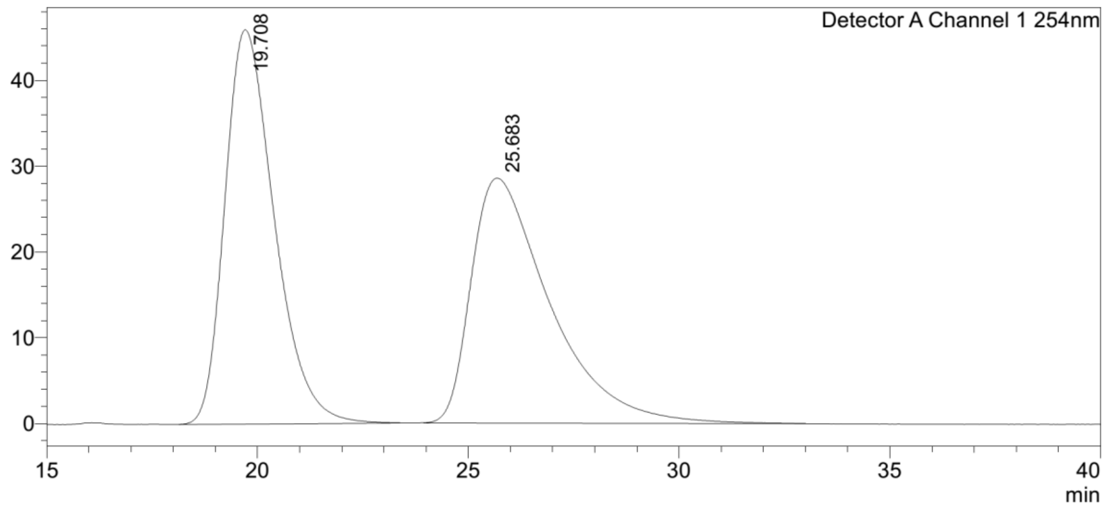

Peak#	Ret. Time	Area%
1	19.708	50.271
2	25.683	49.729
Total		100.000

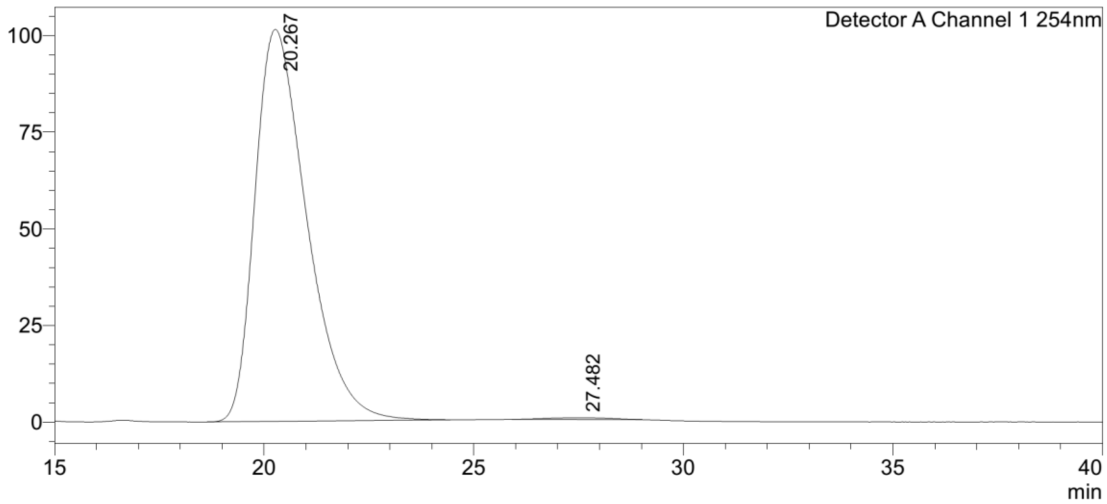

Peak#	Ret. Time	Area%
1	20.267	99.464
2	27.482	0.536
Total		100.000

HPLC Data for **69**: Chiralcel OD-H (93:7 hexane : IPA, flow rate 1.00 mL.min^{-1}, 254 nm, 30 °C) t_R (major): 45.2 min, t_R (minor): 57.4 min, 98:2 er.

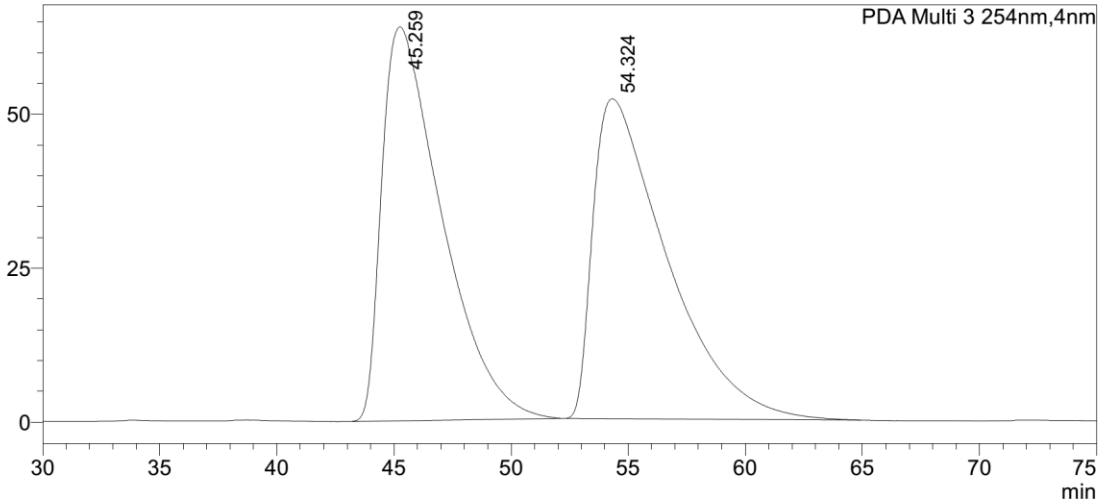

Peak#	Ret. Time	Area%
1	45.259	49.993
2	54.324	50.007
Total		100.000

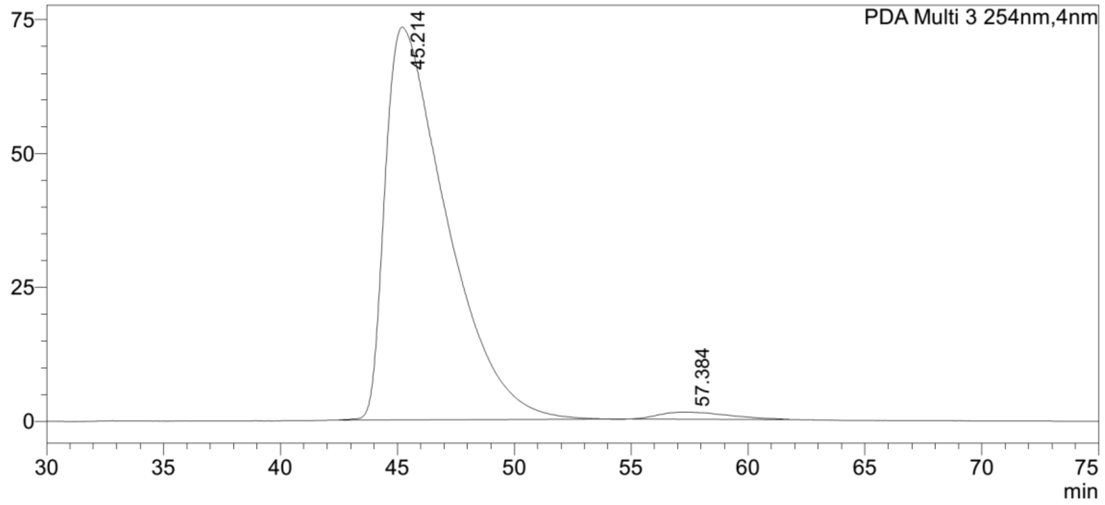

Peak#	Ret. Time	Area%
1	45.214	98.022
2	57.384	1.978
Total		100.000

HPLC Data for **76**: Chiralcel IC (80:20 hexane : IPA, flow rate 1.50 mL.min^{-1}, 254 nm, 40 °C) t_R (major): 34.6, t_R (minor): 41.7 min, 93:7 er.

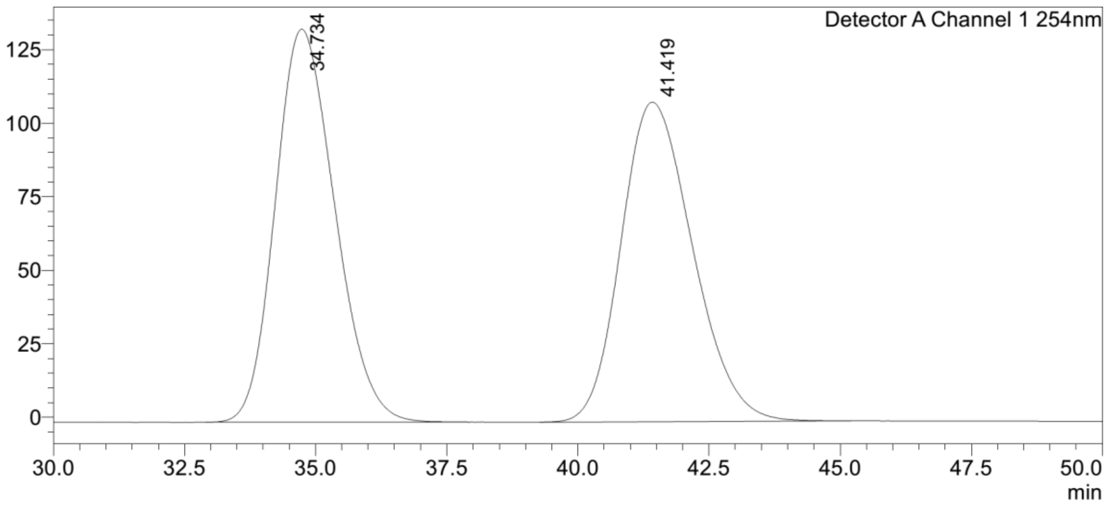

Peak#	Ret. Time	Area%
1	34.734	50.574
2	41.419	49.426
Total		100.000

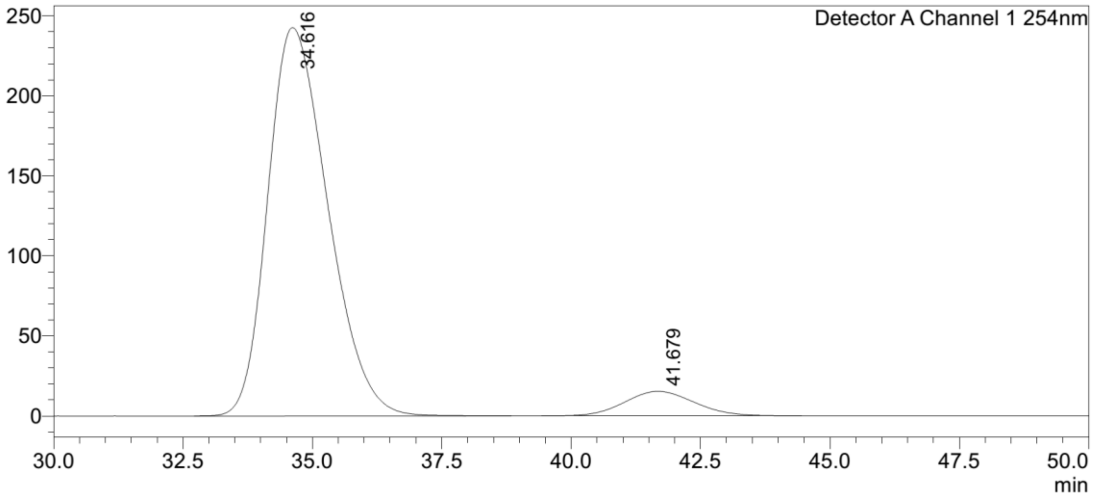

Peak#	Ret. Time	Area%
1	34.616	93.146
2	41.679	6.854
Total		100.000

HPLC Data for **77**: Chiralcel OD-H (93:7 hexane : IPA, flow rate 1.00 mL.min^{-1}, 254 nm, 30 °C) t_R (major): 34.4, t_R (minor): 47.4 min, 98:2 er.

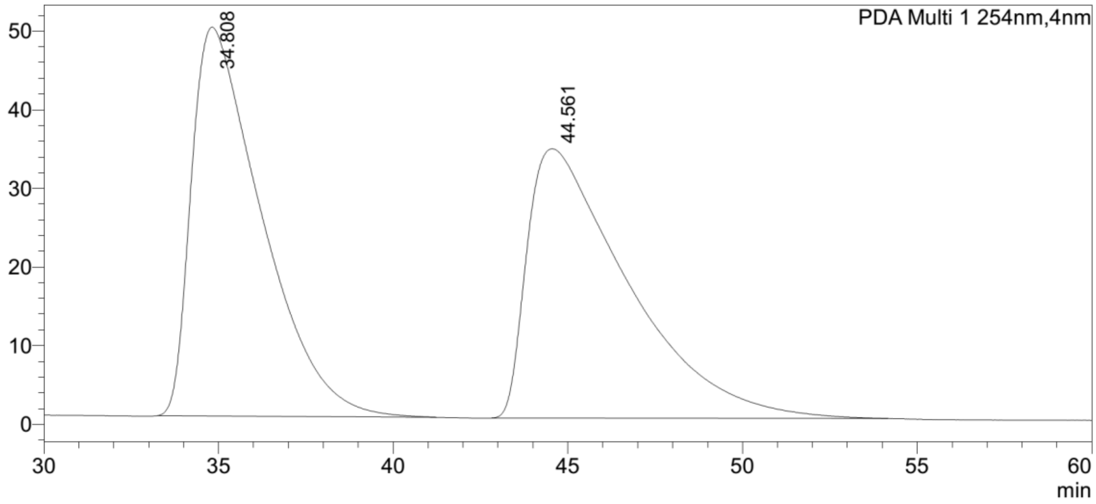

Peak#	Ret. Time	Area%
1	34.808	50.104
2	44.561	49.896
Total		100.000

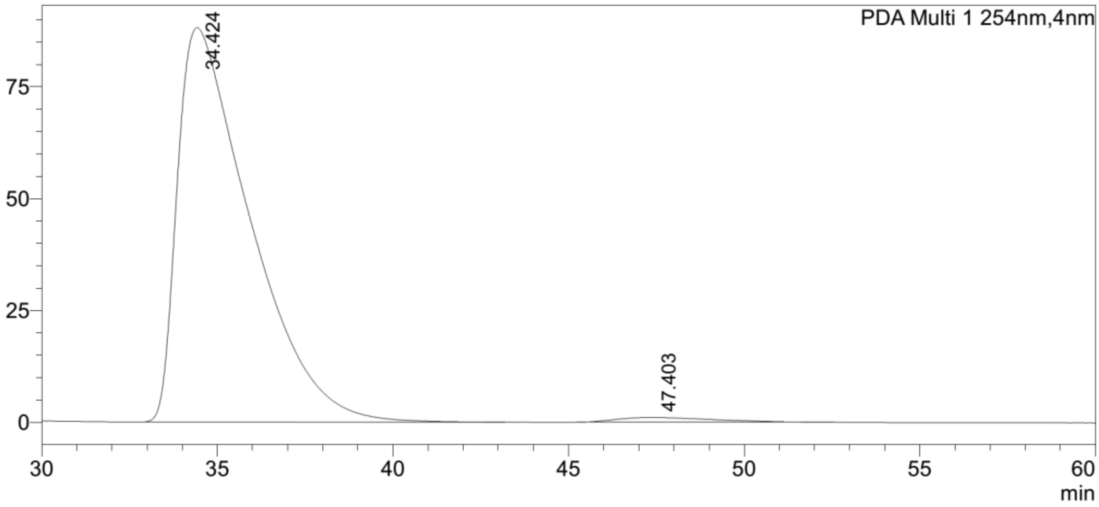

Peak#	Ret. Time	Area%
1	34.424	98.370
2	47.403	1.630
Total		100.000

HPLC Data for **78**: Chiralcel OD-H (93:7 hexane : IPA, flow rate 1.00 mL.min^{-1}, 254 nm, 30 °C) t_R (major): 35.7, t_R (minor): 47.5 min, 99:1 er.

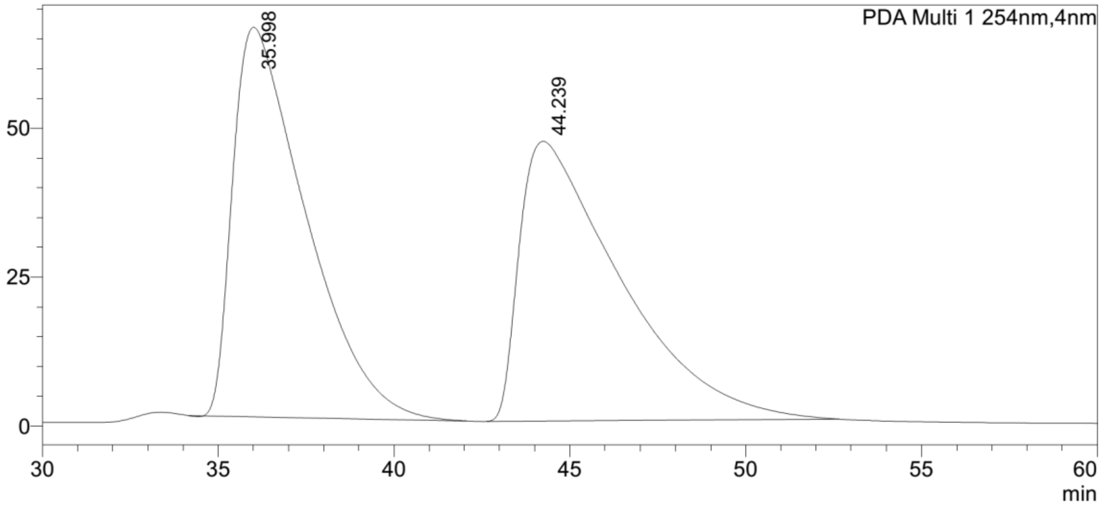

Peak#	Ret. Time	Area%
1	35.998	50.263
2	44.239	49.737
Total		100.000

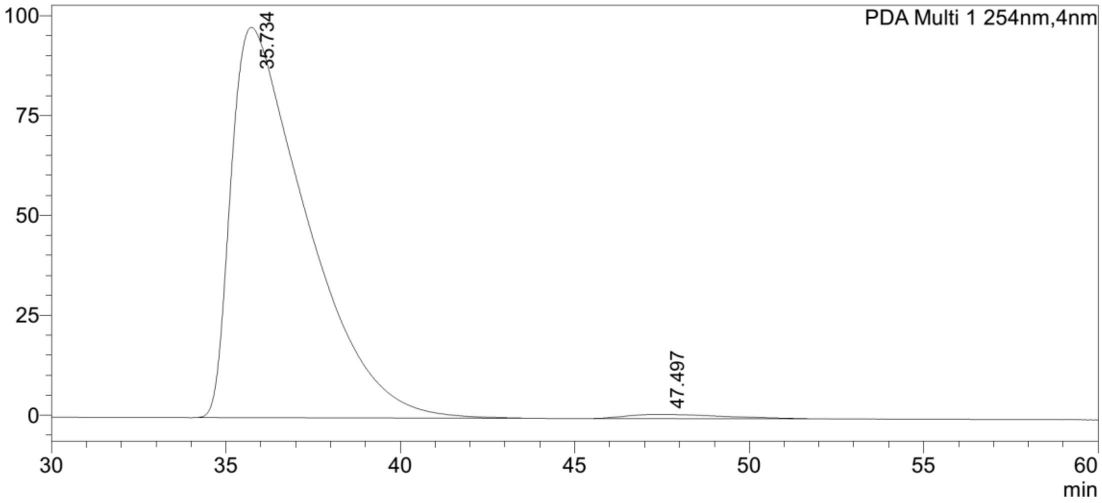

Peak#	Ret. Time	Area%
1	35.734	98.690
2	47.497	1.310
Total		100.000

HPLC Data for **79**: Chiralcel OD-H (93:7 hexane : IPA, flow rate 1.00 mL.min^{-1}, 254 nm, 30 °C) t_R (major): 51.8, t_R (minor): 66.3 min, 86:14 er.

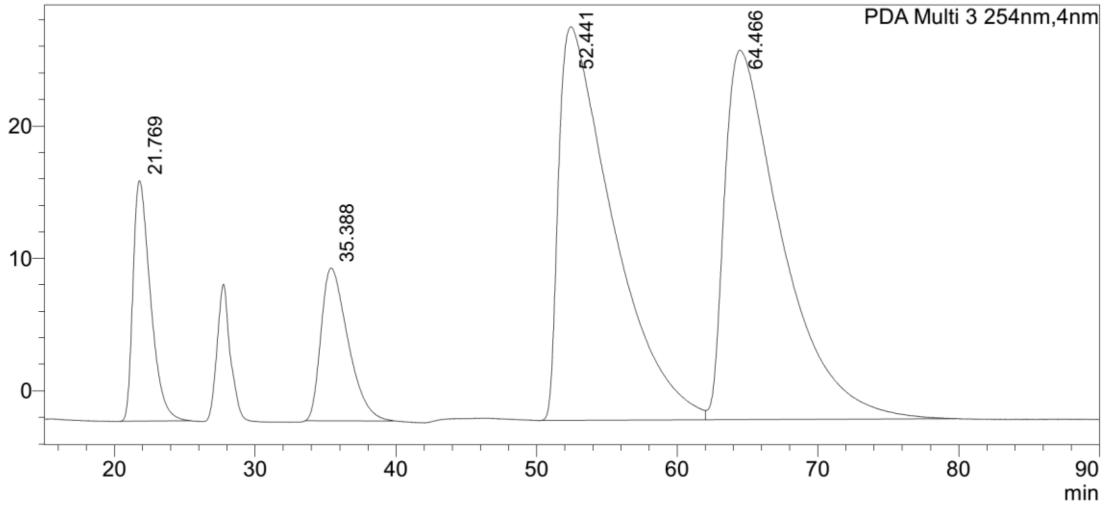

Peak#	Ret. Time	Area%
1	21.769	8.252
2	35.388	8.121
3	52.441	41.844
4	64.466	41.783
Total		100.000

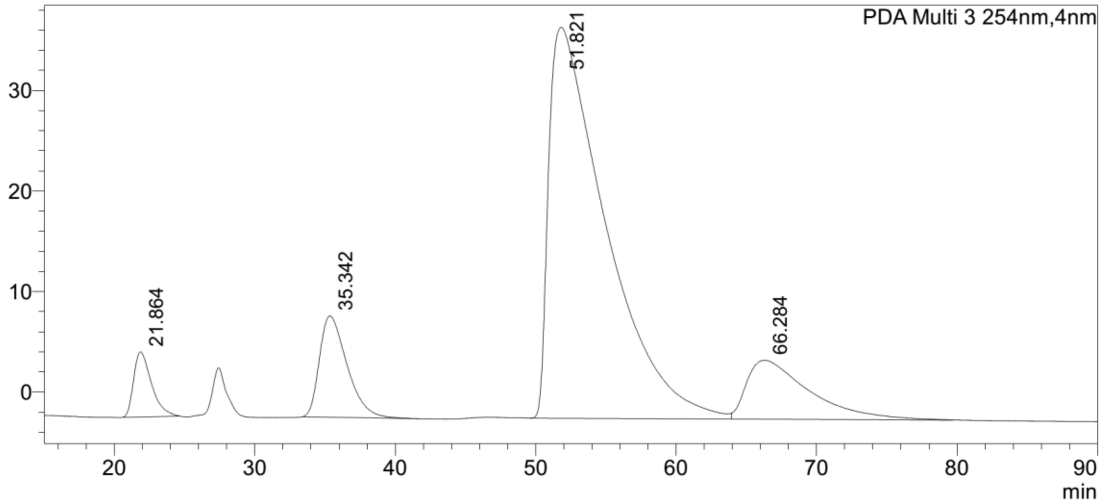

Peak#	Ret. Time	Area%
1	21.864	3.869
2	35.342	9.355
3	51.821	74.439
4	66.284	12.337
Total		100.000

HPLC Data for **80$_{major}$ and 80$_{minor}$**: Chiralcel IC (90:10 hexane : IPA, flow rate 0.50 mL.min^{-1}, 254 nm, 40 °C) t_R (minor): 49.1, t_R (major): 128.1 min, 99:1 er; t_R (major): 173.9 min, t_R (minor): 185.9, 79:21 er.

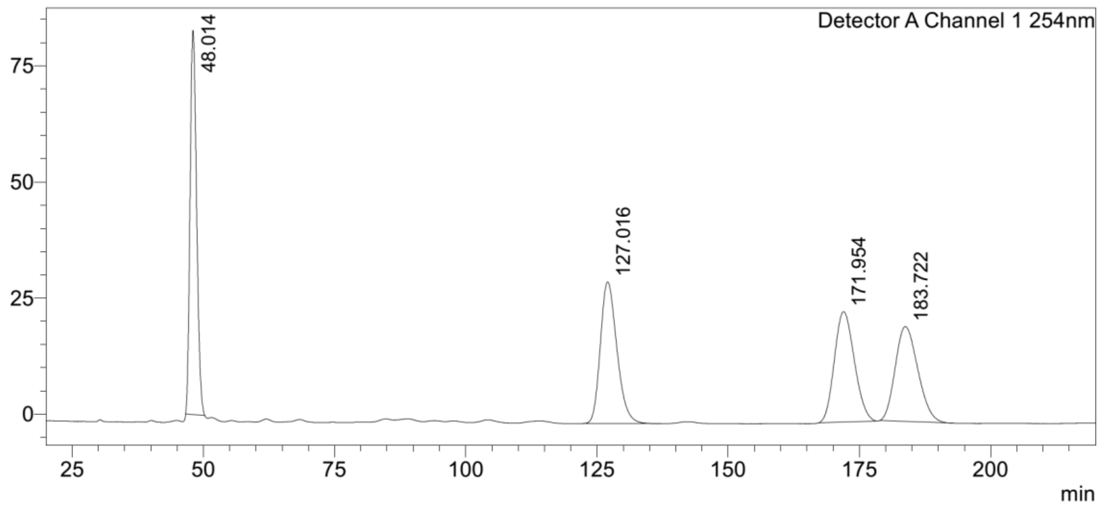

Peak#	Ret. Time	Area%
1	48.014	27.270
2	127.016	25.545
3	171.954	23.899
4	183.722	23.286
Total		100.000

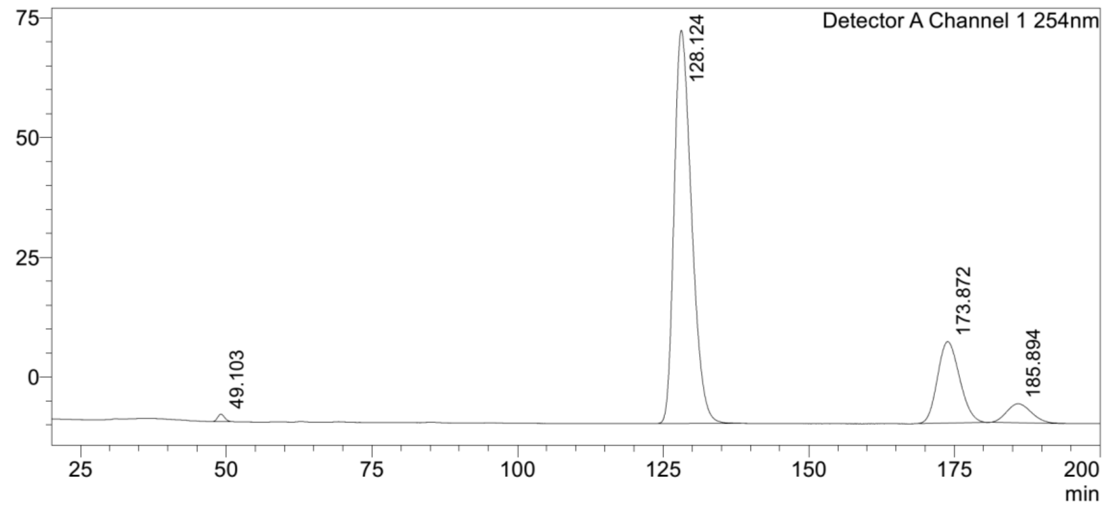

Peak#	Ret. Time	Area%
1	49.103	0.556
2	128.124	74.746
3	173.872	19.623
4	185.894	5.075
Total		100.000

HPLC Data for **81$_{major}$**: Chiralcel OD-H (85:15 hexane : IPA, flow rate 1.00 mL.min^{-1}, 254 nm, 30 °C) t_R (major): 23.5, t_R (minor): 31.1 min, 97:3 er.

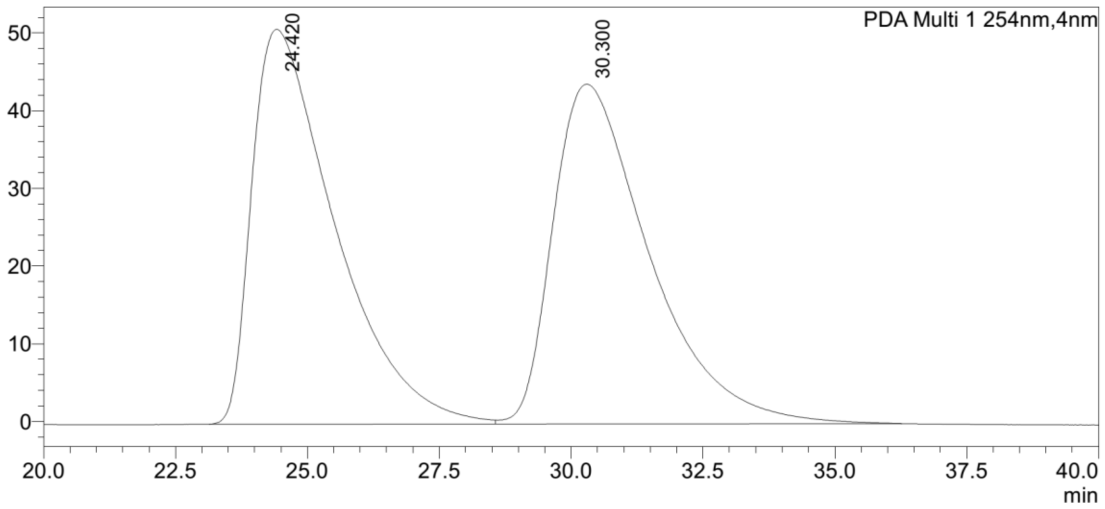

Peak#	Ret. Time	Area%
1	24.420	50.013
2	30.300	49.987
Total		100.000

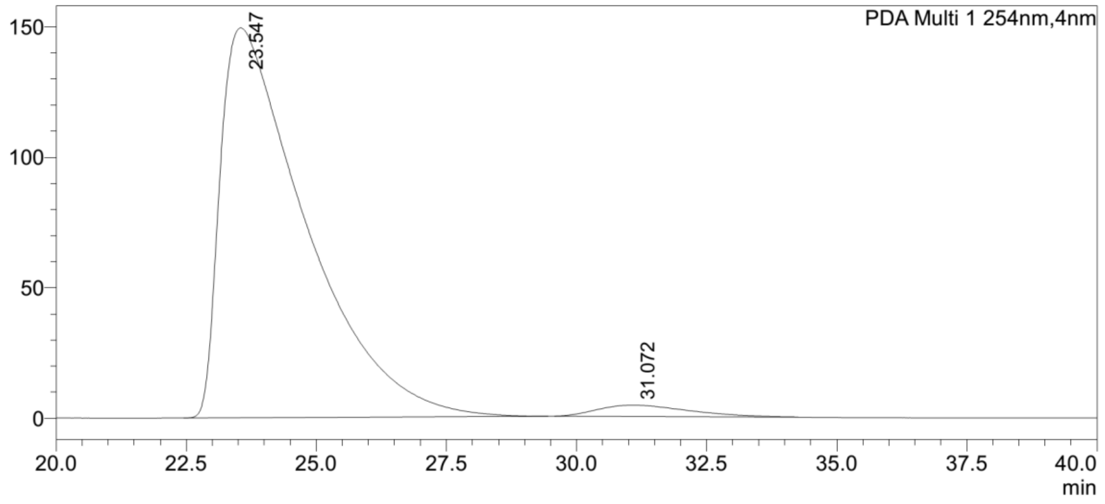

Peak#	Ret. Time	Area%
1	23.547	96.952
2	31.072	3.048
Total		100.000

HPLC Data for **81**$_{\mathbf{minor}}$: Chiralcel OD-H (85:15 hexane : IPA, flow rate 1.00 mL.min^{-1}, 254 nm, 30 °C) t_R (minor): 11.8 min , t_R (major): 14.7, 6:94 er.

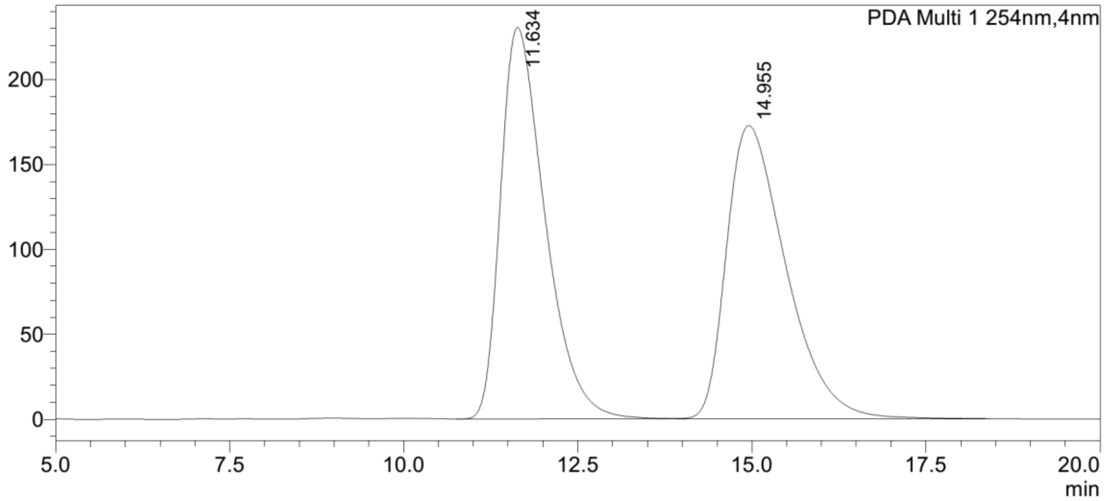

Peak#	Ret. Time	Area%
1	11.634	49.984
2	14.955	50.016
Total		100.000

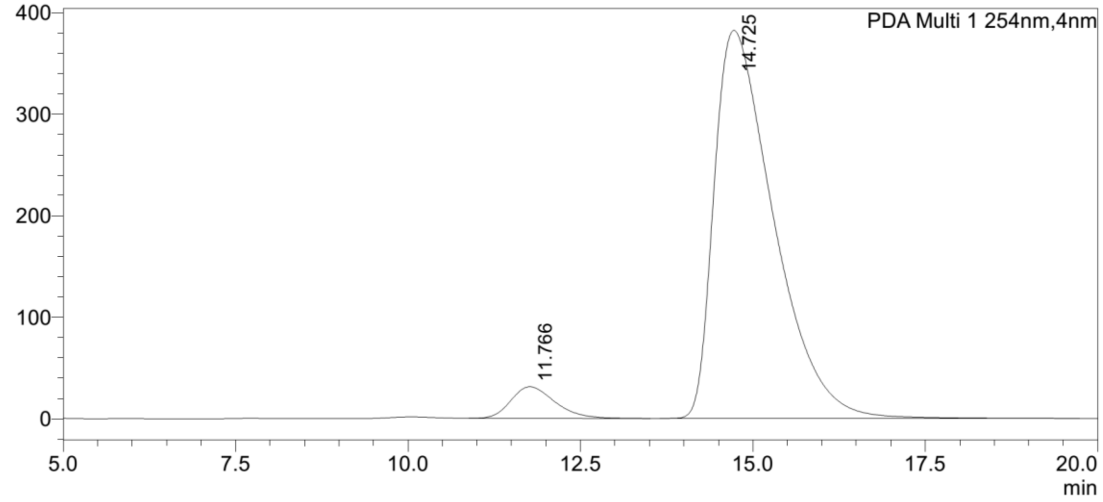

Peak#	Ret. Time	Area%
1	11.766	5.716
2	14.725	94.284
Total		100.000
